# Controlled Precursor Differentiation Enables Palladium-Catalyzed Divergent Carbonylation of Cyclobutenols

**DOI:** 10.1021/jacs.6c11571

**Published:** 2026-07-17

**Authors:** Yu-Kun Liu, Peng Yang, Hefei Yang, Jiajun Zhang, Xiao-Feng Wu

**Affiliations:** † 28392 Leibniz-Institut für Katalyse e.V., Albert-Einstein-Straße 29a, 18059 Rostock, Germany; ‡ Dalian National Laboratory for Clean Energy, Dalian Institute of Chemical Physics, Chinese Academy of Sciences 116023, Dalian, Liaoning, China

## Abstract

Controlling reaction selectivity is a central challenge in synthetic chemistry, particularly when multiple competing reactivity modes coexist within a single substrate. Existing strategies generally rely either on selectivity control during substrate activation or on downstream divergence from a common intermediate. However, these paradigms are less effective when distinct precursor states can independently evolve into distinct reaction manifolds. Herein, we introduce precursor differentiation as a distinct strategy for achieving divergent carbonylation. Through condition-controlled modulation of substrate evolution, a common cyclobutenol substrate can be selectively diverted into two distinct reactive precursors prior to catalytic engagement, thereby enabling access to two different carbonylation pathways. Under palladium catalysis, this strategy enables the highly selective synthesis of either hydroxyl-retained cyclobutanecarboxamides or cyclobutenamides from the same cyclobutenol platform. The method exhibits broad substrate scope (99 examples), consistently high selectivity (>20:1), and compatibility with pharmaceuticals and biologically relevant molecules. Furthermore, the resulting cyclobutenamide products serve as versatile platform intermediates for the selective synthesis of structurally distinct 3-azabicyclo[3.2.0]­heptane and 3-azabicyclo[3.1.1]­heptane frameworks. Mechanistic studies support a condition-controlled precursor differentiation process prior to carbonylation, providing a conceptual basis for achieving divergent carbonylation through selective control of precursor evolution.

## Graphical abstract

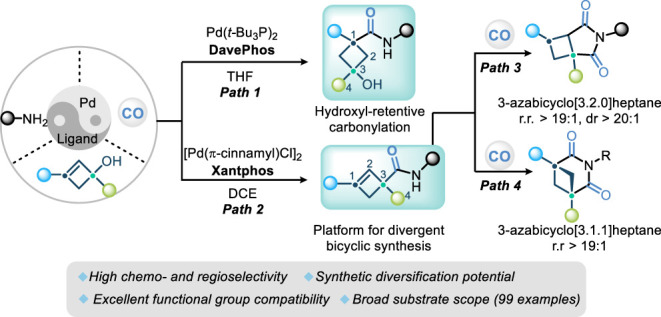

## Introduction

Four-membered carbocycles have attracted increasing attention in medicinal chemistry and act potential as valuable bioisosteric motifs.^1^ Among them, carbonyl-substituted four-membered carbocycles represent important structural motifs in biologically active molecules, natural products, and pharmaceutical agents (Figure 1a).^2^ Beyond their biological significance, four-membered carbocycles have also emerged as versatile platforms for molecular diversification owing to their unique structural features and diverse reactivity.^3^ In recent years, carbonylative transformations have become powerful tools for the functionalization of four-membered carbocycles, enabling efficient access to structurally diverse carbonyl-containing molecules.^4^ These transformations can be broadly classified into three categories: strain-release ring-opening carbonylation,^5^ nonring-opening carbonylative functionalization with retention of the four-membered scaffold,^6^ and carbonylative skeletal remodeling (Figure 1b).^7^ Collectively, these strategies have significantly expanded the synthetic utility of four-membered carbocycles. Nevertheless, owing to the substantial differences in reactivity among competing reaction modes available to strained four-membered systems, most reported transformations proceed through a single dominant reaction pathway. As a result, carbonylative strategies that enable divergent access to structurally differentiated four-membered frameworks from a common substrate platform remain scarce.

**Figure 1:**
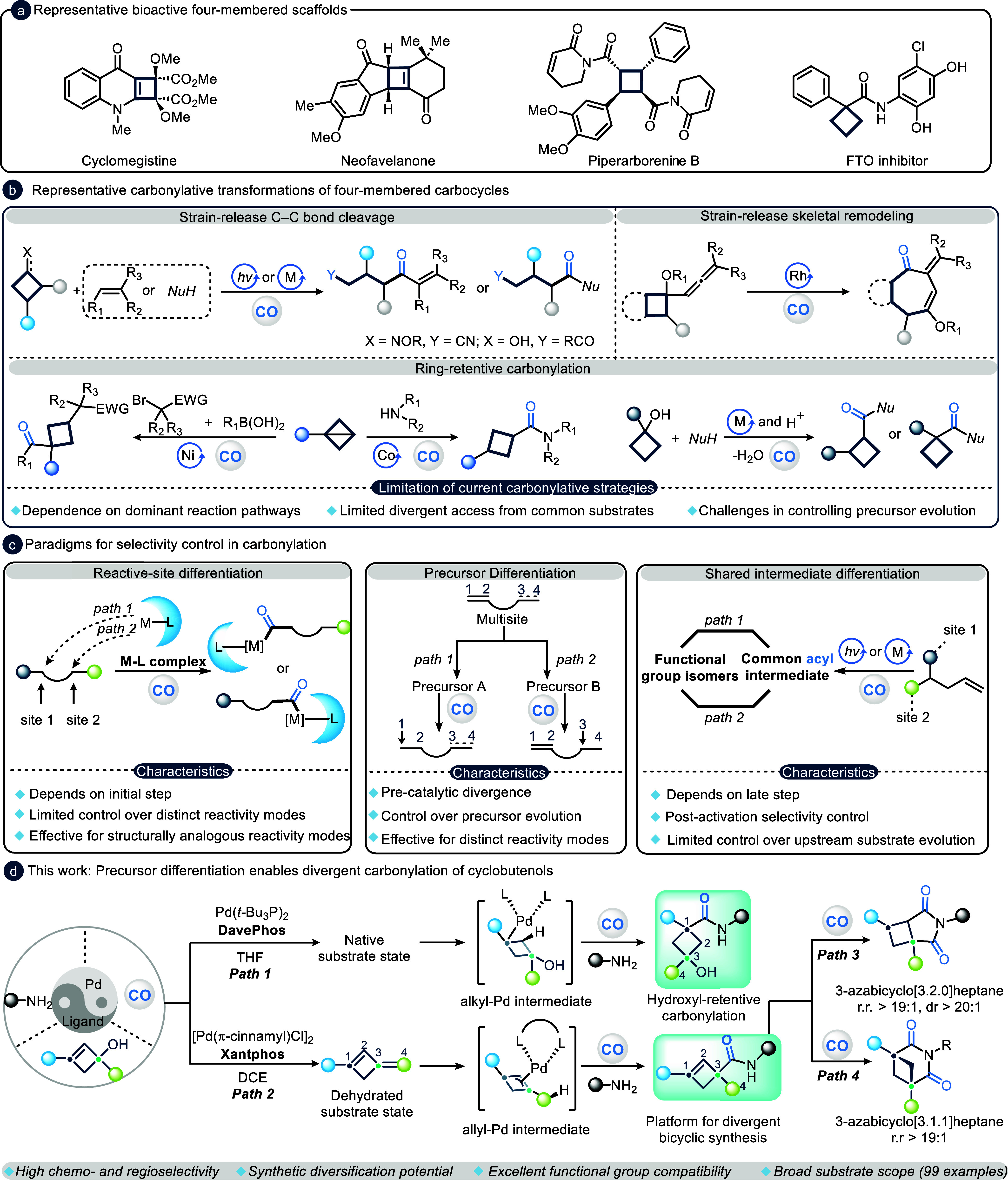
Carbonylative transformations of four-membered carbocycles and the design of precursor differentiation.

In this context, cyclobutenols represent a particularly attractive yet highly challenging substrate platform.^3c,8^ The coexistence of an alkene moiety, a hydroxyl group, and a highly strained four-membered framework within a compact molecular architecture endows cyclobutenols with diverse reactivity,^9^ while the inherent strain-driven reactivity of four-membered ring systems often favors rapid strain-release processes,^10^ rendering precise control over their reaction trajectories particularly challenging.^11^ Consistent with this reactivity bias, existing studies on cyclobutenol substrates have often exploited strain-release-driven skeletal reorganization,^12^ with comparatively few examples involving selective functionalization of the native scaffold.^13^ However, strategies that overcome this inherent reactivity bias to enable divergent synthesis from a common cyclobutenol platform remain essentially unexplored.

Against this backdrop, selective carbonylative transformations represent particularly attractive strategies for addressing this challenge, enabling rapid access to structurally diverse carbonyl-containing architectures.^14^ However, in most reported systems, reaction selectivity is generally achieved through two principal paradigms. The first relies on reactive-site differentiation during substrate activation, enabling selective discrimination between structurally analogous reactive sites, such as competing unsaturated bonds^15^ or multiple electrophilic positions (Figure 1c left),^16^ whereas the second proceeds through a shared carbonylative pathway, in which a common acyl-metal intermediate undergoes downstream divergence through tandem carbonylative transformations^17^ or chemoselective trapping by distinct nucleophilic functionalities within the substrate (Figure 1c right).^18^ By contrast, when multiple intrinsically distinct reactivity modes coexist within a multifunctional substrate, these established selectivity paradigms may prove inadequate for effective differentiation.^19^ We therefore questioned whether a common substrate could instead be deliberately diverted into fundamentally distinct reactive precursors prior to productive catalytic engagement, thereby enabling divergent carbonylative outcomes (Figure 1c mid).

Guided by this design principle, we herein report a condition-controlled palladium-catalyzed divergent carbonylation of cyclobutenols with diverse amines through precursor differentiation (Figure 1d). This strategy provides selective access to two structurally distinct carbonyl-containing four-membered scaffolds from a common cyclobutenol platform. The method exhibits excellent selectivity, broad substrate scope, and strong functional-group compatibility. Furthermore, the resulting cyclobutenamide products serve as versatile platform intermediates for the selective synthesis of structurally distinct aza-bicyclic frameworks.

## Methods

### General Procedure for the Synthesis of **3**

#### Condition A

A vial (4 mL) was charged with Pd­(*t*-Bu_3_P)_2_ (5 mol %), DavePhos (7 mol %), cyclobutenol (**1**, 2.0 equiv., 0.2 mmol), amine hydrochloride (**2**, 1.0 equiv., 0.1 mmol) and a stirring bar. The vial was closed by PTFE/white rubber septum (Wheaton 13 mm Septa) and phenolic cap and connected with atmosphere with a needle. The vial was evacuated under vacuum and recharged with argon for three times. After that, THF (2.0 mL) was added with a syringe under nitrogen atmosphere. Subsequently, the vial (or several vials) was placed in an alloy plate, which was transferred into a 300 mL autoclave of the 4560 series from Parr Instruments. After flushing the autoclave three times with CO, a pressure of 40 bar of CO was adjusted at ambient temperature. Then, the reaction was performed for 16 h at 90 °C. After the reaction was complete, the autoclave was cooled down with ice water to room temperature and the pressure was released carefully. The solution was concentrated in vacuo then purified by silica-gel column chromatography using pentane and ethyl acetate to afford the corresponding product **3.**

### General Procedure for the Synthesis of **4**

#### Condition B

A vial (8 mL) was charged with [Pd­(π-cinnamyl)­Cl]_2_ (5 mol %), XantPhos (15 mol %), cyclobutenol (**1**, 2 equiv., 0.2 mmol) amine hydrochloride (**2**, 1.0 equiv, 0.1 mmol) and a stirring bar. The vial was closed by PTFE/white rubber septum (Wheaton 13 mm Septa) and phenolic cap and connected with atmosphere with a needle. The vial was evacuated under vacuum and recharged with argon for three times. After that, DCE (3.5 mL), was added with a syringe under nitrogen atmosphere. Subsequently, the vial (or several vials) was placed in an alloy plate, which was transferred into a 300 mL autoclave of the 4560 series from Parr Instruments. After flushing the autoclave three times with CO, a pressure of 40 bar of CO was adjusted at ambient temperature. Then, the reaction was performed for 16 h at 80 °C. After the reaction was complete, the autoclave was cooled down with ice water to room temperature and the pressure was released carefully. The solution was concentrated in vacuo then purified by silica-gel column chromatography using pentane and ethyl acetate to afford the corresponding product **4**.

## Results and Discussion

We commenced our studies using cyclobutenol **1a** and aniline hydrochloride **2a** as model substrates under a CO atmosphere (Figure 2). Initial evaluation employing Pd­(*t*-Bu_3_P)_2_ and MePhos (**L1**) in THF at 90 °C under 40 bar of CO furnished the hydroxyl-retentive carbonylation product **3a** in 40% yield with excellent selectivity (>20:1) (entry 1), thereby demonstrating the feasibility of achieving selective hydroxyl-retentive carbonylation under the reaction conditions. Subsequent ligand evaluation revealed a pronounced influence of phosphine structure on reaction selectivity. Although ligands **L2**–**L4** delivered yields (entries 2–4) comparable to that obtained with MePhos (**L1**), a substantial erosion of selectivity was observed, highlighting the sensitivity of pathway selectivity to subtle structural modifications of the ligand framework. Gratifyingly, DavePhos (**L5**) significantly improved the reaction outcome, affording **3a** in 50% yield while maintaining excellent selectivity (entry 5). In contrast, JohnPhos (**L6**) exhibited comparable catalytic activity but diminished selectivity (entry 6), whereas the more sterically demanding ligand **L7** and the structurally related ligand **L8** resulted in lower yields and selectivities (entries 7–8). Consequently, DavePhos was identified as the optimal ligand for this transformation. With an effective ligand in hand, further optimization of the reaction parameters was undertaken. Increasing the loading of cyclobutenol **1a** to 2.0 equiv improved the yield of **3a** to 84% without compromising selectivity (entry 9). Subsequent adjustment of the reaction concentration and ligand loading ultimately furnished **3a** in 98% yield with outstanding selectivity (>20:1) (entry 10). Having established efficient conditions for the selective synthesis of **3a**, we next sought to access the complementary product **4a** selectively. To this end, ligand screening was reinitiated under a DCE/[Pd­(π-cinnamyl)­Cl]_2_ reaction system. To our delight, ligands **L9**–**L11** all promoted the desired transformation, with **L10** proving optimal and delivering **4a** in 49% yield with excellent selectivity (entries 11–13). Evaluation of additional ligand classes (**L12**–**L16**) failed to provide further improvement in either reactivity or selectivity (entry 14). Further optimization of the reaction temperature and substrate ratio (See Supporting Information for details) increased the yield of **4a** to 63% while maintaining excellent selectivity (entry 15). Finally, adjustment of the catalyst-to-ligand ratio furnished **4a** in 84% yield with a > 20:1 product ratio (entry 16).

**Figure 2:**
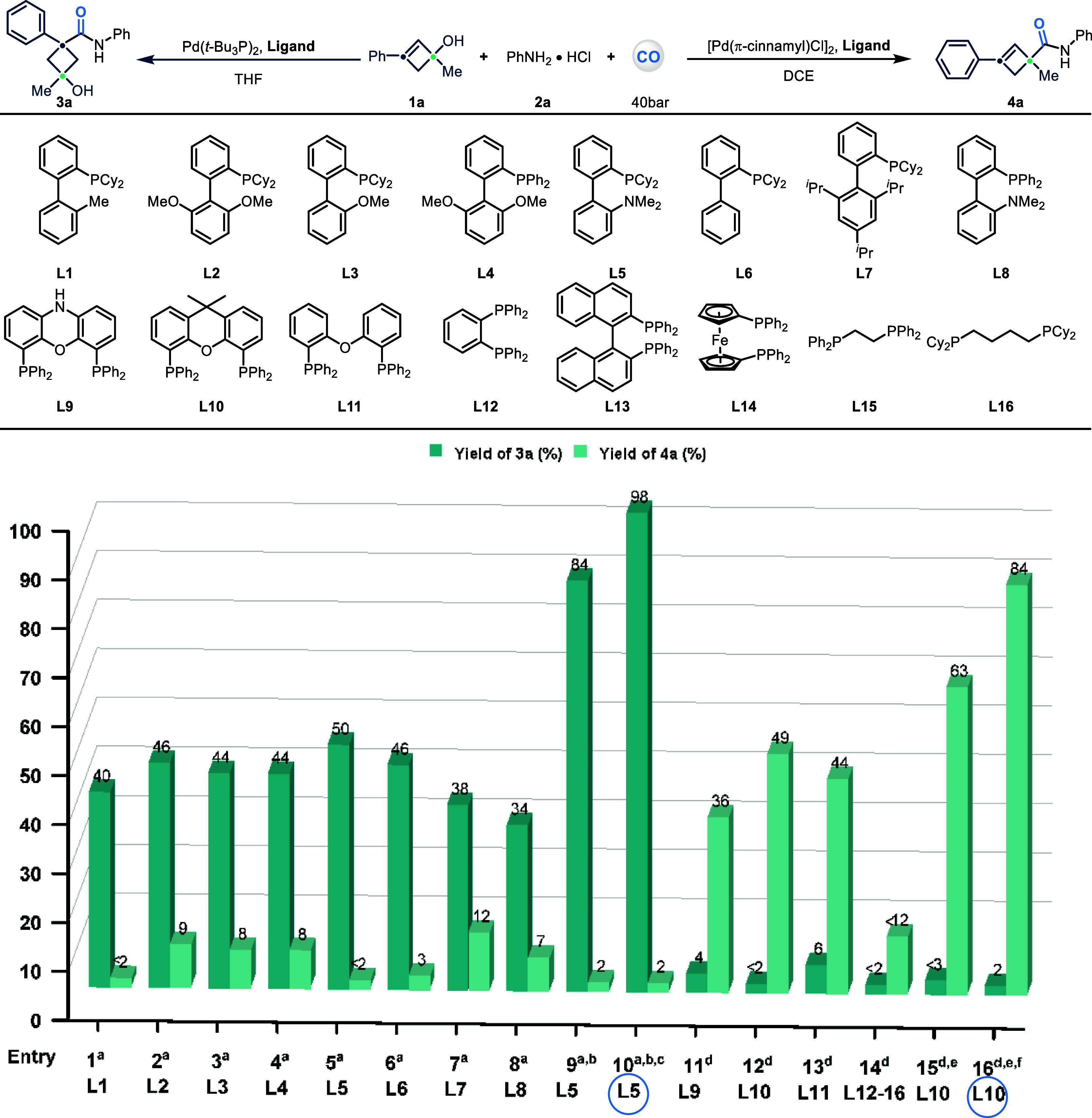
Reaction optimization for the precursor differentiation enables divergent carbonylation of cyclobutenols. [a] Reaction conditions: Pd­(*t*-Bu_3_P)_2_ (5 mol %), **Ligand** (10 mol %), **1a** (0.12 mmol), **2a** (0.1 mmol), CO (40 bar), THF (1.0 mL) at 90 °C for 16 h. [b] **1a** (0.2 mmol). [c] THF (2.0 mL), **L5** (7 mol %). [d] [Pd­(π-cinnamyl)­Cl]_2_ (2.5 mol %), **Ligand** (5 mol %), **1a** (0.12 mmol), **2a** (0.1 mmol), CO (40 bar), DCE (1.0 mL) at 60 °C for 16 h. [e] **1a** (0.2 mmol), 80 °C [f] DCE (3.5 mL), [Pd­(π-cinnamyl)­Cl]_2_ (5 mol %), **L10** (15 mol %).

With the optimized conditions in hand, we next investigated the substrate scope of the hydroxyl-retentive carbonylation process (Table 1, left). Notably, excellent chemo-selectivity was maintained throughout the entire substrate scope, with all examined substrates furnishing hydroxyl-retained products **3** preferentially over the corresponding dehydrative carbonylation products **4** (**3**:**4** > 20:1). Using aniline hydrochloride **2a** as the coupling partner, the desired product **3a** was obtained in 96% isolated yield. The structure of **3a** was confirmed by single-crystal X-ray diffraction analysis (CCDC 2554488). We first examined the steric effects of arylamine substrates. Ortho-, meta-, and para-methyl-substituted aniline hydrochlorides all participated in the reaction, delivering the corresponding products **3b**–**3d** in excellent yields, indicating that the reaction was largely insensitive to the substitution pattern on the aromatic ring. Subsequently, the electronic effects of the arylamine component were evaluated. Both electron-rich and electron-deficient anilines proved to be competent substrates. In particular, the *para*-methoxy-substituted substrate afforded product **3e** in 98% yield. Halogen-substituted substrates bearing F, Cl, and Br groups were also well tolerated, providing the desired products **3f**–**3h** in good yields. Moreover, a variety of synthetically useful functionalities, including *tert*-butyl, acetyl, and acetoxy groups, were readily accommodated, furnishing products **3i**–**3k** in excellent yields. Importantly, fluorinated substituents such as trifluoromethyl, trifluoromethoxy, and trifluoromethylthio were all compatible with the catalytic system, affording the corresponding products **3l**–**3n** in good yields. A benzyloxy-substituted substrate was likewise successfully converted to product **3o**, further demonstrating the broad functional-group tolerance of this transformation. Moreover, 3-aminothiophene hydrochloride proved to be a viable substrate, delivering heteroaryl product **3p** in 63% yield. To further probe positional effects, *ortho*- and *meta*-substituted aniline derivatives were examined. Substrates bearing *ortho*-F, *ortho*-methylthio, *ortho*-difluoromethoxy, and *meta*-methoxy substituents all underwent the reaction efficiently to furnish products **3q**–**3t** in good to excellent yields, suggesting that steric hindrance exerts only a minor influence on the reaction outcome. The protocol was also applicable to more structurally complex arylamines. Various polysubstituted anilines smoothly delivered products **3u**–**3w** in high yields. In addition, fused aromatic and heteroaromatic amines were well tolerated. Naphthyl, benzodioxole, difluorobenzodioxole, and coumarin-containing substrates all afforded the desired products **3x**–**3ab** in synthetically useful yields Despite the broad substrate scope observed for aromatic amines, aliphatic amine hydrochlorides proved unreactive under the stand conditions, and the corresponding products **3ac** and **3ad** were not observed.

**Table 1:**
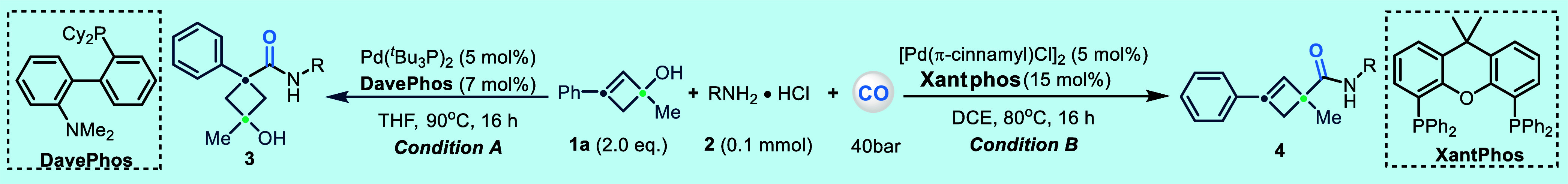

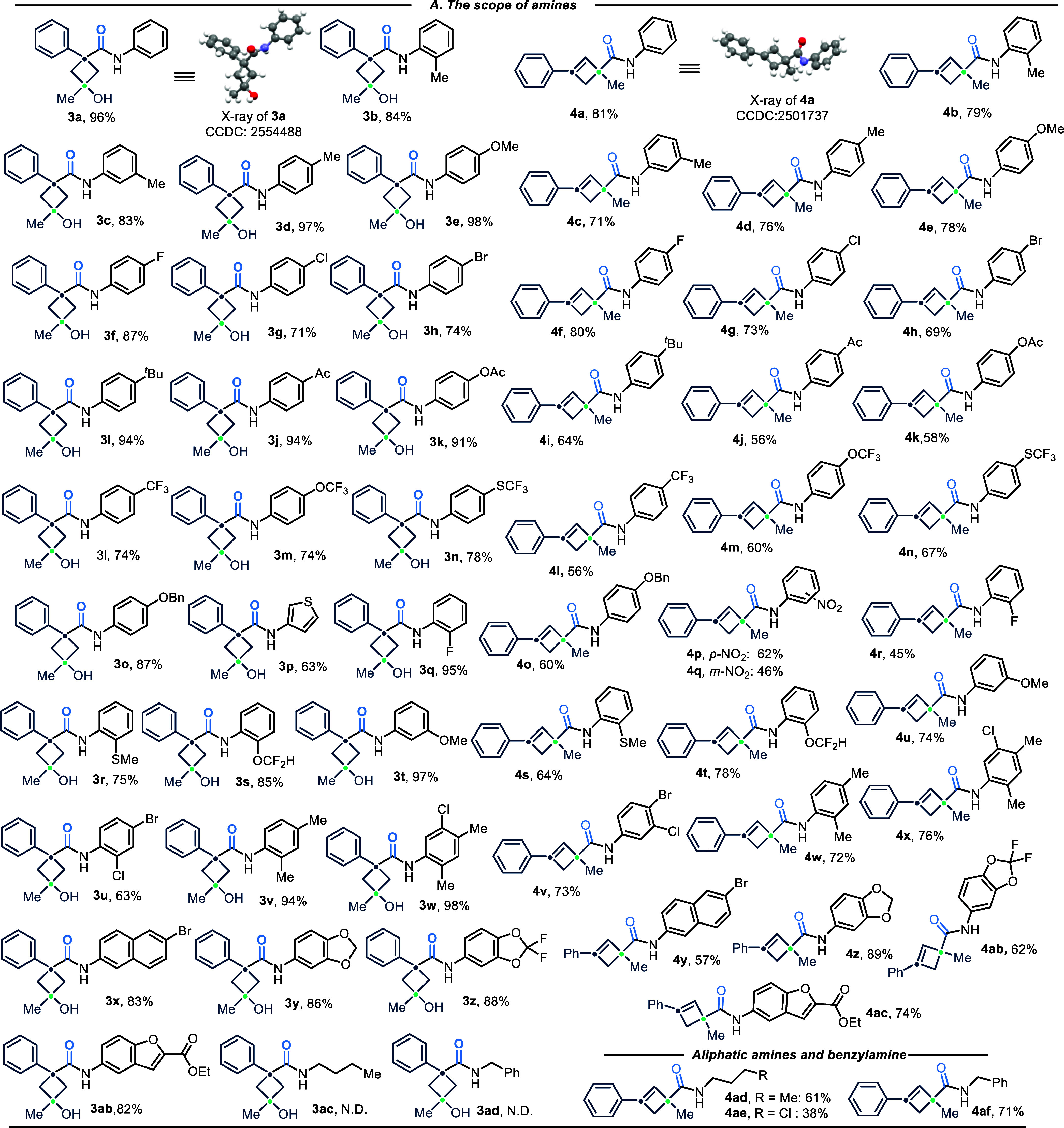
Aniline Scope in the Precursor-Differentiated Divergent Carbonylation^a^

a Reaction conditions: Condition: A Pd­(*t*-Bu_3_P)_2_ (5 mol %), **DavePhos** (7 mol %), **1a** (0.2 mmol), **2** (0.1 mmol), CO (40 bar), THF (2.0 mL) at 90 °C for 16 h. [b] Condition B: [Pd­(π-cinnamyl)­Cl]_2_ (5 mol %), **XantPhos** (15 mol %), **1a** (0.2 mmol), **2** (0.1 mmol), CO (40 bar), DCE (3.5 mL) at 80 °C for 16 h. [c] All yields were isolated yields. For all examples shown, product ratios of **3**:**4** (or **4**:**3**) were greater than 20:1.

Having established a hydroxyl-retentive carbonylation manifold under Condition A, we next explored the scope of the complementary dehydrative carbonylation process under Condition B (Table 1, right). Using aniline hydrochloride **2a** as the model substrate, the desired cyclobutenamide product **4a** was isolated in 81% yield. The structure of **4a** was confirmed by single-crystal X-ray diffraction analysis (CCDC 2501737). Gratifyingly, the dehydrative carbonylation pathway also proceeded with uniformly high chemoselectivity across all substrates examined (**4**:**3** > 20:1). The reaction proved tolerant to a broad range of amine hydrochlorides. Initial evaluation of methyl-substituted substrates revealed little positional effect, as *ortho*-, *meta*-, and *para*-methyl anilines all afforded the corresponding products **4b**–**4d** in comparable yields. Electronic variation on the aromatic ring was likewise well accommodated. Electron-rich substrates bearing methoxy and *tert*-butyl groups delivered products **4e** and **4i**, while halogen-substituted substrates bearing F, Cl, and Br substituents furnished products **4f**–**4h** in good yields. Aniline derivatives containing acetyl and acetoxy substituents also participated effectively, providing products **4j** and **4k**. The protocol exhibited broad functional-group compatibility. Aniline derivatives bearing trifluoromethyl, trifluoromethoxy, or trifluoromethylthio substituents were all competent substrates, affording products **4l**–**4n** in moderate to good yields. Likewise, a benzyloxy-substituted substrate underwent the transformation to furnish product **4o**. Notably, strongly electron-withdrawing nitro-substituted anilines also proved viable coupling partners, delivering products **4p** and **4q**. Variation of the substitution pattern was further examined through a collection of *ortho*- and *meta*-substituted aniline derivatives. Substrates bearing *ortho*-F, *ortho*-methylthio, and *ortho*-difluoromethoxy groups, as well as a *meta*-methoxy-substituted substrate, all underwent efficient conversion to provide products **4r**–**4u**. The transformation was also applicable to polysubstituted arylamines, furnishing products **4v**–**4x** in consistently good yields. The synthetic utility of the method was further demonstrated by its compatibility with more structurally elaborate amine substrates. Naphthyl and various benzo-fused heterocyclic motifs, including benzodioxole, difluorobenzodioxole, and coumarin derivatives, were readily incorporated to furnish cyclobutenamide products **4y**–**4ac** in synthetically useful yields. In contrast to the hydroxyl-retentive manifold under Condition A, the present dehydrative carbonylation manifold also accommodated aliphatic amines. Both alkyl amines and benzylamine successfully participated in the reaction, affording products **4ad**–**4af** in 38–71% yields. These promising results with aliphatic amines are benefited from the increased acidic conditions compared with Condition A which failed with **3ac** and **3ad**.

To further evaluate the substrate scope of the condition-controlled divergent carbonylation process, a series of aryl- and nonaryl-substituted cyclobutenols were prepared from the corresponding alkynes through cyclobutenone intermediate and then examined under both catalytic systems (Table 2). Encouragingly, excellent chemoselectivities were observed throughout the substrate scope, with product ratios greater than 20:1 in all successful examples. We first investigated the influence of the electronic properties of the aryl substituent on the reaction outcome. Both electron-rich and electron-deficient cyclobutenols were well tolerated under the reaction conditions. Cyclobutenols containing aryl groups substituted with electron-donating functionalities such as methyl, methoxy, and *tert*-butyl groups underwent the transformation, affording the hydroxyl-retained cyclobutanecarboxamide products **3ae**, **3af**, and **3ai** in 75–80% yields. Under Condition B, the corresponding cyclobutenamide products **4ag**, **4ah**, and **4ak** were likewise obtained in 60–80% yields. Similarly, cyclobutenols with fluoro- and chloro-substituted aryl groups were readily accommodated by both catalytic systems. Products **3ag** and **3ah** were obtained in 89% and 92% yields, and the corresponding cyclobutenamide products **4ai** and **4aj** were formed under Condition B. These halogenated products also provide opportunities for further synthetic elaboration. Notably, a cyclobutenol featuring a trifluoromethyl-substituted aryl group proved to be a competent substrate under both catalytic systems, affording products **3aj** and **4al** in 58% and 40% yields, respectively, indicating that the electronic nature of the aryl substituent exerts only a limited influence on the reaction efficiency. The scope was further extended to fused aromatic and heteroaromatic cyclobutenols. A naphthyl-substituted cyclobutenol delivered products **3ak** and **4am** in 81% and 73% yields. Comparable reactivity was observed for a *meta*-fluorophenyl-substituted cyclobutanol, which afforded products **3al** and **4an** in 72% and 77% yields. An *ortho*-methyl-substituted substrate also reacted to afford product **4ao** in 83% yield, demonstrating the good tolerance of the catalytic system toward *ortho*-substitution. In addition, thiophene- and benzothiophene-substituted cyclobutenols participated in both catalytic manifolds, furnishing products **3am** and **3an** under Condition A and products **4ap** and **4aq** under Condition B in moderate to excellent yields. These results further highlight the broad heteroarene compatibility of this divergent transformation. We next investigated the influence of substitution on the cyclobutenol framework itself. Beyond aryl-substituted substrates, alkyl-substituted cyclobutenols also participated effectively in both reaction manifolds, furnishing products **3ao** and **4ar**. Furthermore, replacement of the bridgehead methyl group with *n*-butyl or *n*-hexyl substituents were well tolerated, providing products **3ap**, **3aq**, **4as**, and **4at**. In contrast, when the bridgehead substituent was changed to a phenyl group or hydrogen atom, neither catalytic system was able to efficiently deliver the corresponding products, indicating that substitution at the bridgehead position is crucial for maintaining the reactivity of the cyclobutenol substrate.

**Table 2:**
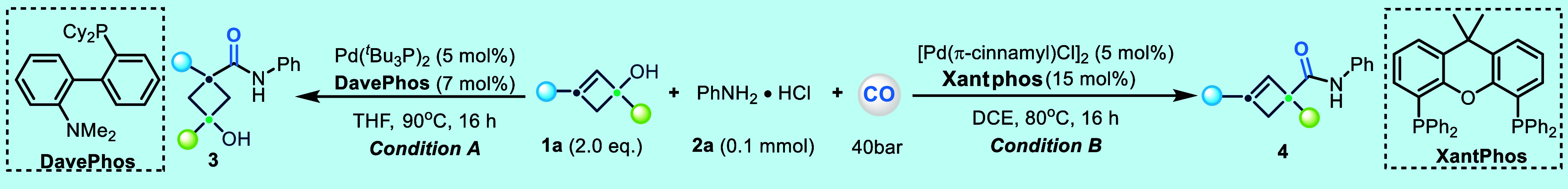

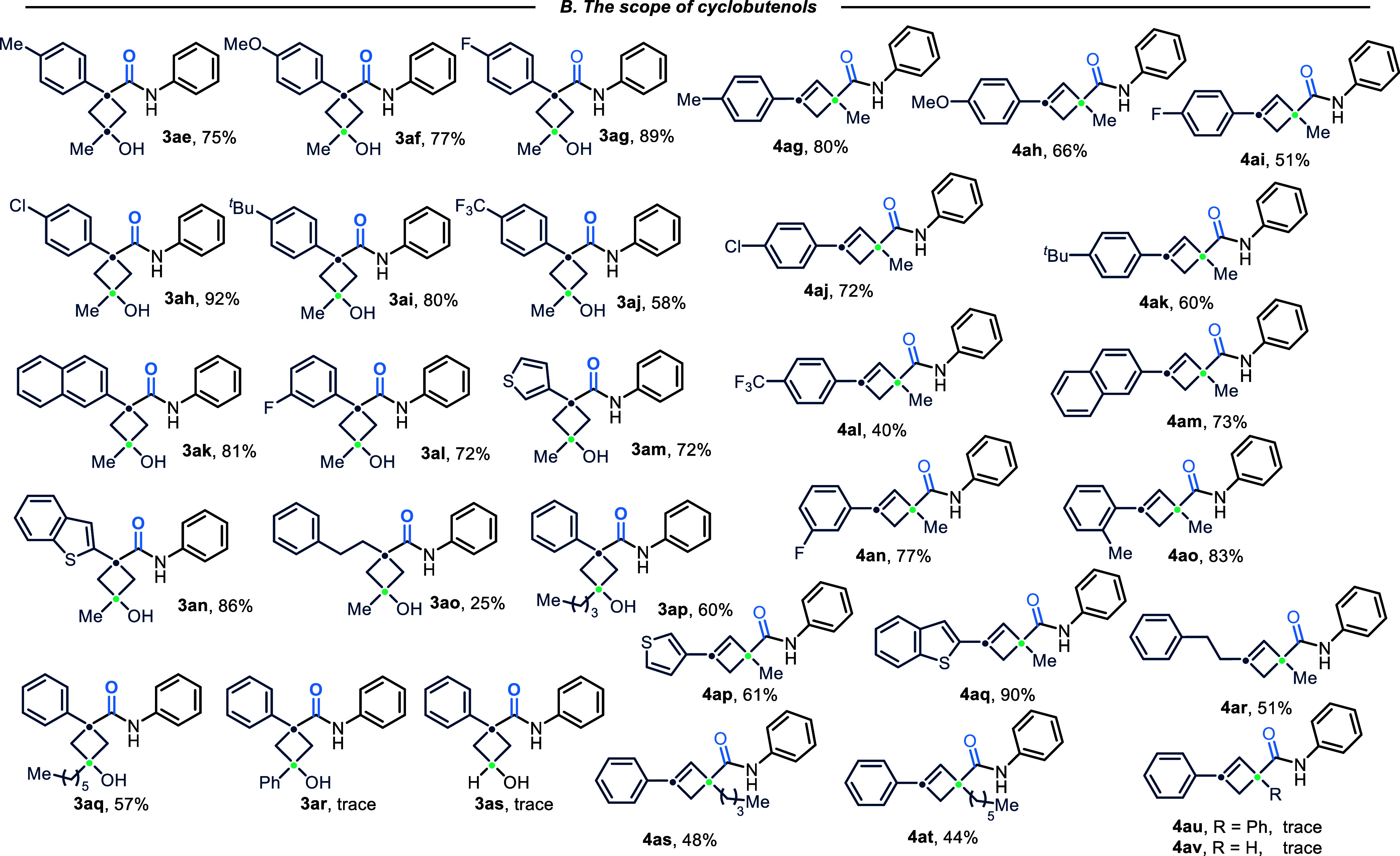
Cyclobutenols Scope in the Precursor-Differentiated Divergent Carbonylation^a^

a Reaction conditions: Condition A: Pd­(*t*-Bu_3_P)_2_ (5 mol %), **DavePhos** (7 mol %), **1** (0.2 mmol), **2a** (0.1 mmol), CO (40 bar), THF (2.0 mL) at 90 °C for 16 h.[b] Condition B [Pd­(π-cinnamyl)­Cl]_2_ (5 mol %), **XantPhos**(15 mol %), **1** (0.2 mmol), **2a** (0.1 mmol), CO (40 bar), DCE (3.5 mL) at 80 °C for 16 h. [c] All yields were isolated yields. For all examples shown, product ratios of **3**:**4** (or **4**:**3**) were greater than 20:1.

To make this divergent carbonylation strategy more attractive, we explored its applicability to the late-stage diversification of pharmaceuticals and bioactive molecules (Table 3). A variety of marketed drug-derived substrates were readily accommodated under both catalytic systems. Derivatives of Naproxen, Ibuprofen, Gemfibrozil, and Loxoprofen all underwent the transformation under Condition A, affording the corresponding hydroxyl-retained cyclobutanecarboxamide products **3at**–**3aw** in moderate to good yields. In addition, a menthol-derived substrate was readily converted to the desired product **3ax** in 85% yield. These substrates also performed well under Condition B, furnishing the corresponding cyclobutenamide products **4aw**–**4bb** in 70–83% yields while maintaining excellent chemoselectivity throughout. Furthermore, amino acid derivatives were likewise compatible with the catalytic system. Substrates derived from l-valine, l-phenylalanine, l-alanine, and l-phenylglycine participated in the reaction, affording products **4bc**–**4bf** in 64–70% yields. Collectively, these results underscore the utility of this divergent carbonylation strategy for the rapid diversification of structurally complex and biologically relevant molecules.

**Table 3:**
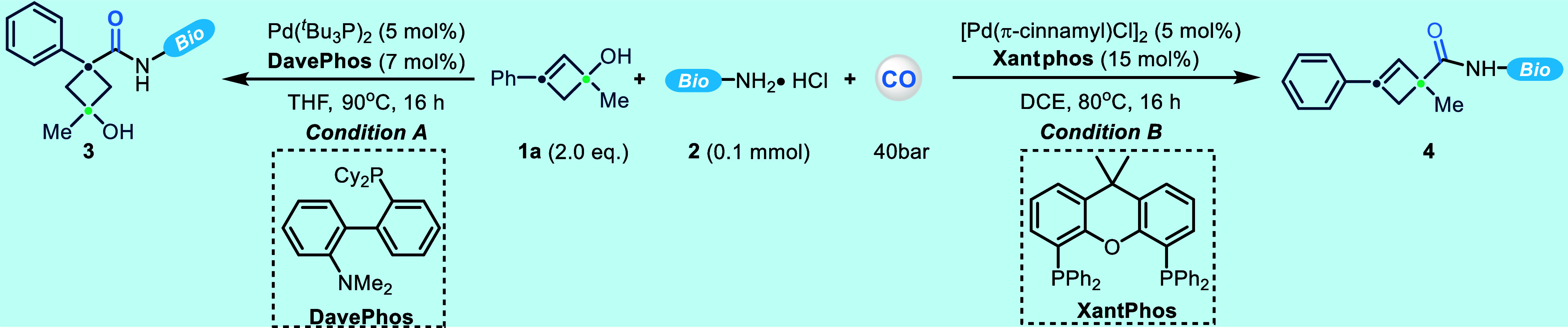

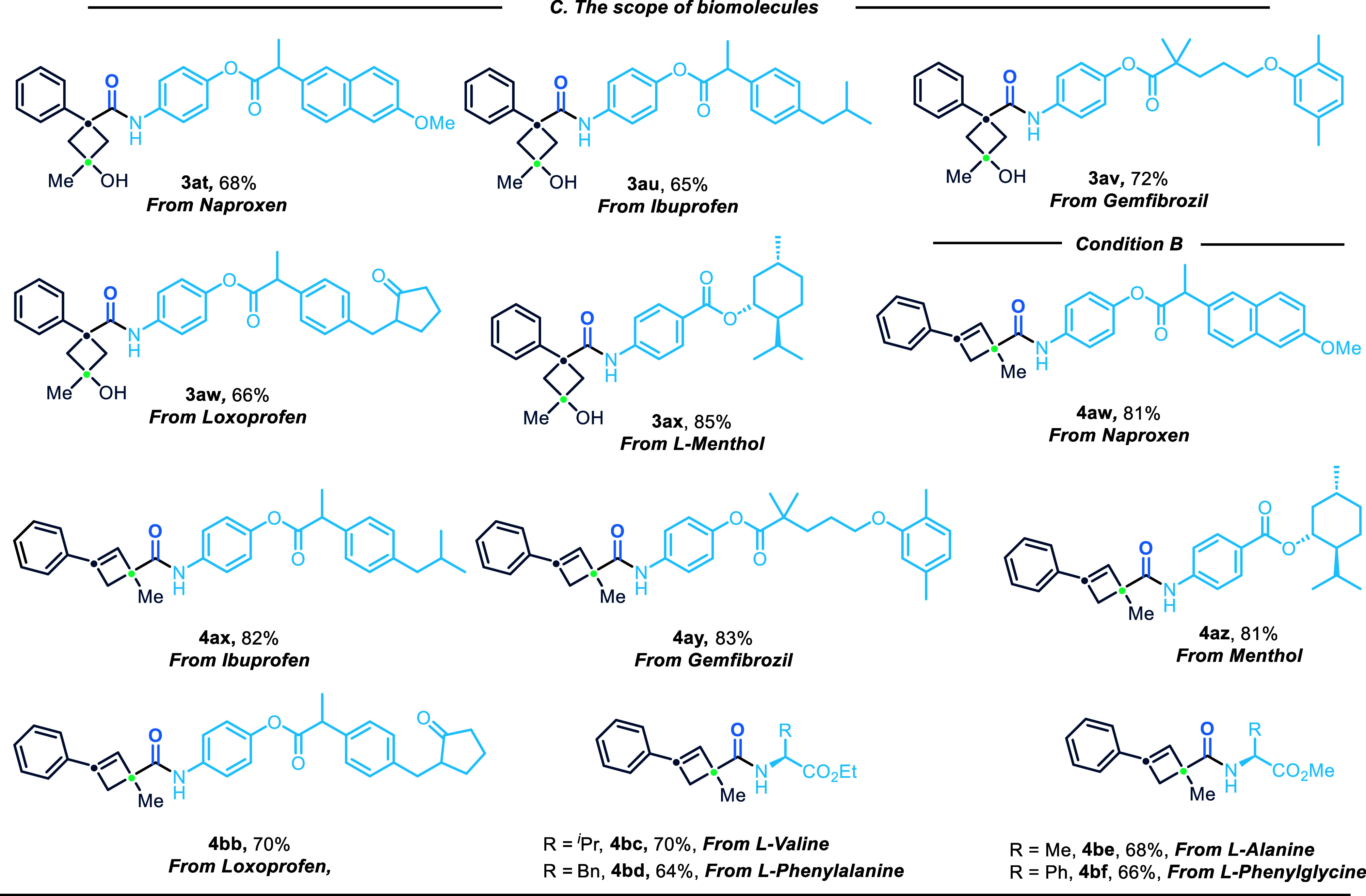
Biomolecule Scope in the Precursor-Differentiated Divergent Carbonylation^a^

a Reaction conditions: Condition A: Pd­(*t*-Bu_3_P)_2_ (5 mol %), **DavePhos** (7 mol %), **1a** (0.2 mmol), **2** (0.1 mmol), CO (40 bar), THF (2.0 mL) at 90 °C for 16 h.[b] Condition B [Pd­(π-cinnamyl)­Cl]_2_ (5 mol %), **XantPhos**(15 mol %), **1a** (0.2 mmol), **2** (0.1 mmol), CO (40 bar), DCE (3.5 mL) at 80 °C for 16 h. [c] All yields were isolated yields. For all examples shown, product ratios of **3**:**4** (or **4**:**3**) were greater than 20:1.

To further demonstrate the practical utility of this divergent carbonylation strategy, large-scale experiments were first conducted under both sets of reaction conditions (Figure 3a). The desired products **3a** and **4a** were obtained in 87% and 73% yields, respectively, while maintaining excellent chemoselectivity, highlighting the scalability and robustness of the protocol. Subsequently, the synthetic utility of product **3a** was explored through several downstream transformations (Figure 3b). Reduction of the amide carbonyl group with LiAlH_4_ provided the corresponding amine derivative **5** in 57% yield. In addition, treatment of **3a** with TFAA and DIPEA enabled efficient conversion of the hydroxyl group into the corresponding trifluoroacetate derivative **6**, further demonstrating the versatility of this scaffold for subsequent functionalization.

**Figure 3:**
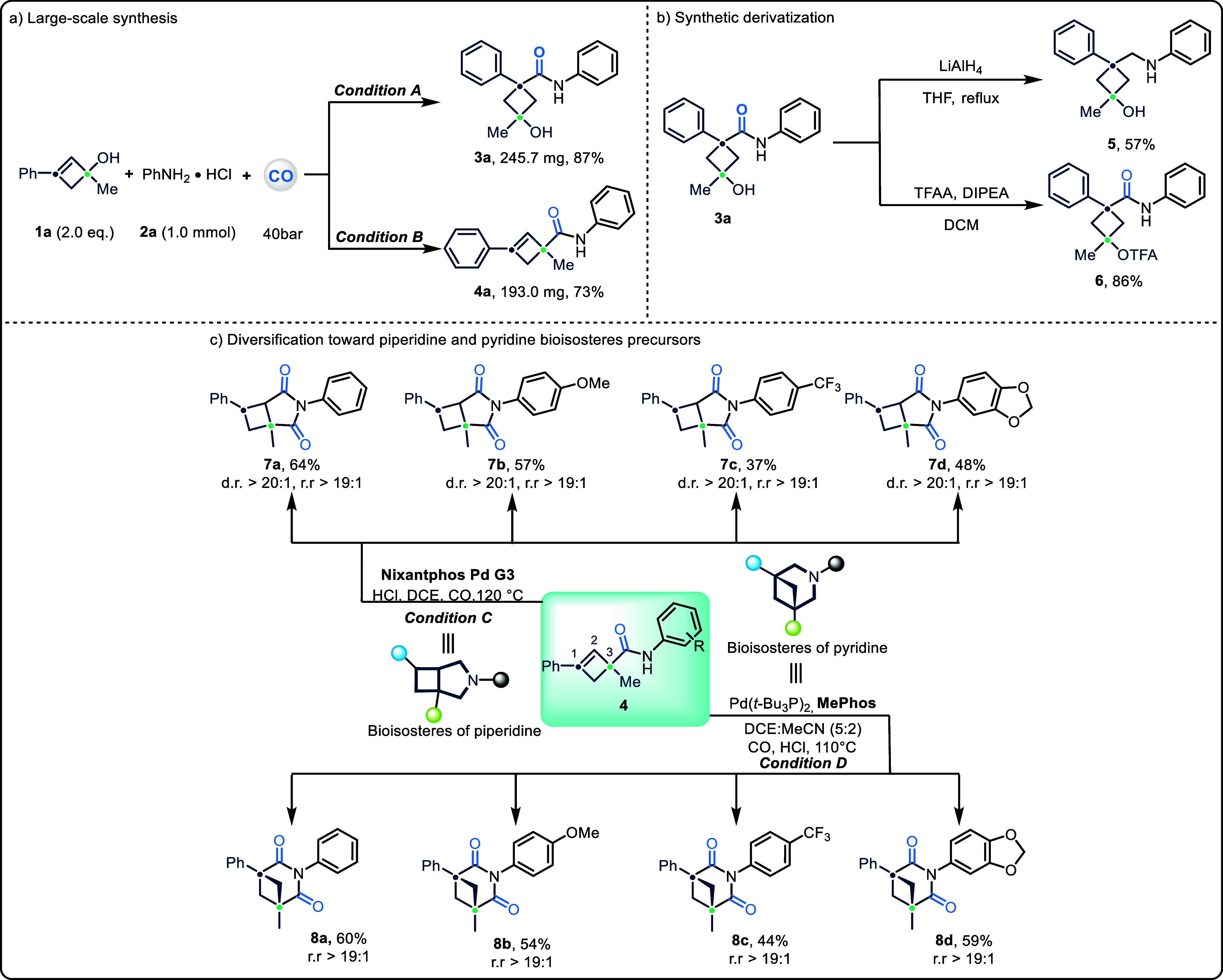
Synthetic applications.

Considering that cyclobutenamide **4** contains both an amide moiety and a strained cyclobutene framework, we envisioned that it could serve not only as the terminal product of the divergent carbonylation process but also as a versatile platform intermediate for the construction of structurally diverse aza-bicyclic scaffolds. Guided by this hypothesis, a series of downstream transformations were investigated (Figure 3c).^19d^ Under NiXantphos Pd G3 catalysis, platform intermediate **4** underwent a 2,3-cyclocarbonylation reaction to furnish a range of 3-azabicyclo[3.2.0]­heptane scaffolds **7a**–**7d** with excellent regioselectivity and diastereoselectivity. In contrast, under Pd­(*t*-Bu_3_P)_2_/MePhos catalysis, the same intermediate selectively participated in a 1,3-cyclocarbonylation process, providing 3-azabicyclo[3.1.1]­heptane scaffolds **8a**–**8d** with equally high regioselectivity. Notably, both classes of saturated aza-bicyclic frameworks can serve as valuable precursors to piperidine^20^ and pyridine^21^ bioisosteres. Collectively, these results demonstrate that the condition-controlled strategy not only enables divergent carbonylation of cyclobutenols but also allows the resulting platform intermediate to be directed toward two distinct classes of high-value aza-bicyclic scaffolds, thereby significantly expanding the potential utility of this methodology in medicinally relevant scaffold construction and molecular diversification.

To gain further insight into the mechanism of the condition-controlled divergent carbonylation process, a series of deuterium-labeling, intermediate verification, control, and Hammett studies were conducted (Figure 4). We first sought to identify the origin of the hydrogen atom incorporated into product **3** (Figure 4a). When deuterated aniline hydrochloride **2a**–**d** was employed under Condition A, only trace deuterium incorporation was observed in product **3a**-*d* **1**, indicating that aniline hydrochloride is not the primary hydrogen source. Considering that trace amounts of water may be present in the reaction system, D_2_O was subsequently introduced into the reaction mixture. Increasing the amount of D_2_O led to progressively higher deuterium incorporation at the C2 position of the product. Furthermore, when deuterated aniline hydrochloride and 10 equiv of D_2_O were employed simultaneously, substantially higher deuterium incorporation was observed compared with the experiment using deuterated aniline hydrochloride alone. These results indicate that trace water might serves, at least in part, as the hydrogen source in the formation of product **3**. However, the possibility that rapid D^+^ and H^+^ exchange between the aniline and D_2_O can not be fully excluded. We next examined the reaction pathway operating under Condition B (Figure 4b). When deuterated aniline hydrochloride **2a**-***d*** was subjected to the standard reaction conditions, significant deuterium incorporation was observed at the C4 position of product **4a**-*d* **1**. This result supports the intermediacy of conjugated diene **9** generated through dehydration of cyclobutenol **1a** prior to carbonylation.

**Figure 4:**
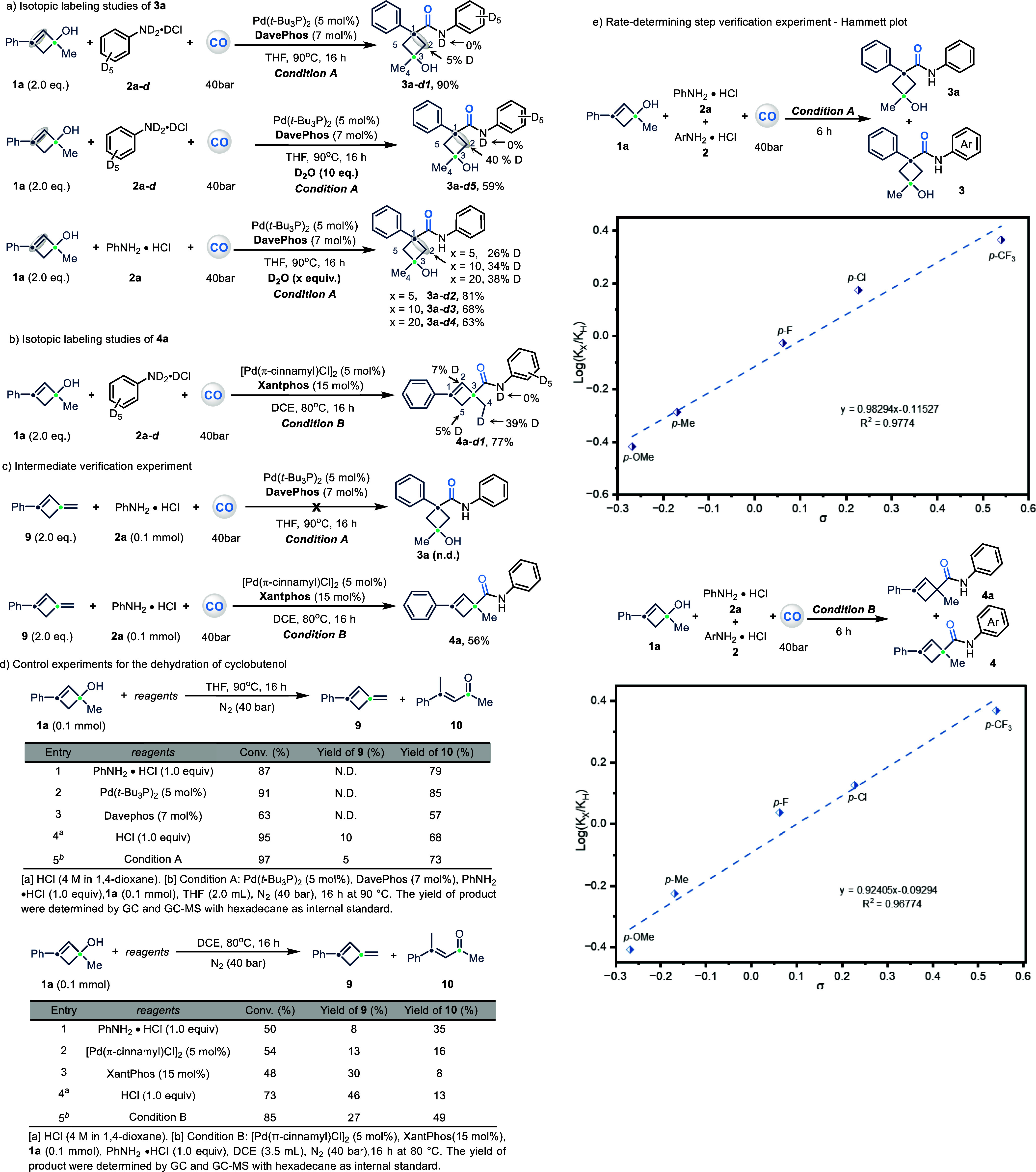
Mechanistic investigations.

To further verify the proposed intermediates, conjugated diene **9** was synthesized and subjected to both catalytic systems (Figure 4c). Under Condition A, no formation of product **3a** was detected. In contrast, diene **9** furnished product **4a** in 56% yield under Condition B. These results identify diene **9** as an intermediate in the formation of product **4**, but not product **3**, indicating that the two pathways diverge prior to carbonylation. Notably, product 3a remained intact upon treatment with TFA, HCl, or *p*-TsOH, with no detectable dehydration or ring-opening observed (see Supporting Information), demonstrating the unexpected stability of the hydroxyl-retained cyclobutanecarboxamide scaffold under acidic conditions. Next, a series of dehydration control experiments were carried out to investigate the formation of reaction precursors under the two sets of reaction conditions (Figure 4d). In THF, regardless of whether aniline hydrochloride, the catalytic system, or HCl was added, cyclobutenol **1a** was predominantly converted into ring-opened product **10**, while only trace amounts of diene **9** were detected. In contrast, when DCE was employed as the solvent, formation of diene **9** was readily observed in all control experiments, accompanied by varying amounts of ring-opened product **10**. Together, these findings suggest that the divergent outcomes arise from the formation of distinct reaction precursors under the two reaction conditions.

To gain further insight into the rate-determining step, Hammett studies were performed using a series of *para*-substituted aniline hydrochlorides under both catalytic systems (Figure 4e). Positive linear correlations between log­(K_X_/K_H_) and σ were observed, giving reaction constants of ρ = 0.98 and ρ = 0.92 for Conditions A and B. Electron-deficient aniline hydrochlorides reacted faster than their electron-rich counterparts in both cases. These results are consistent with ammonolysis of the corresponding acyl intermediates being the likely rate-determining step. The similar ρ values obtained under the two catalytic systems further imply similar electronic demands for the rate-determining steps in both pathways.

Based on the mechanistic studies described above and relevant literature precedents,^15m,15p,22^ a plausible mechanism is proposed to account for the condition-controlled precursor-differentiated carbonylation process (Figure 5). In catalytic cycle A, cyclobutenol **1a** directly enters the catalytic cycle without prior dehydration, while Pd–H species **A** is generated in situ in the presence of the palladium catalyst, ligand, and acid. Regioselective hydropalladation of cyclobutenol **1a** by Pd–H species **A** affords **Int B**, which subsequently undergoes CO insertion to generate acyl-palladium **Int C**. Nucleophilic attack of aniline on **Int C** then furnishes the hydroxyl-retained product **3a**, accompanied by regeneration of the active palladium catalyst. Importantly, besides ligand, the hydroxyl group might also coordinate to the palladium during the carbonylation process and further contribute to the high diastereoselectivity. In contrast, catalytic cycle B proceeds through a distinct precursor. Under Condition B, cyclobutenol **1a** first undergoes dehydration to form conjugated diene intermediate **9**, while Pd–H species **D** is generated in situ under the reaction conditions. Migratory insertion of diene **9** into the Pd–H bond affords a π-allyl palladium intermediate, which exists as two interconverting regioisomeric forms, **Int E** and **Int E′**. Among them, **Int E′** is more favorable for the subsequent CO insertion step, leading to the formation of acyl–palladium complex **Int F**. Finally nucleophilic attack of aniline on **Int F** delivers cyclobutenamide product **4a** and regenerates Pd–H species **D**, thereby completing catalytic cycle B.

**Figure 5:**
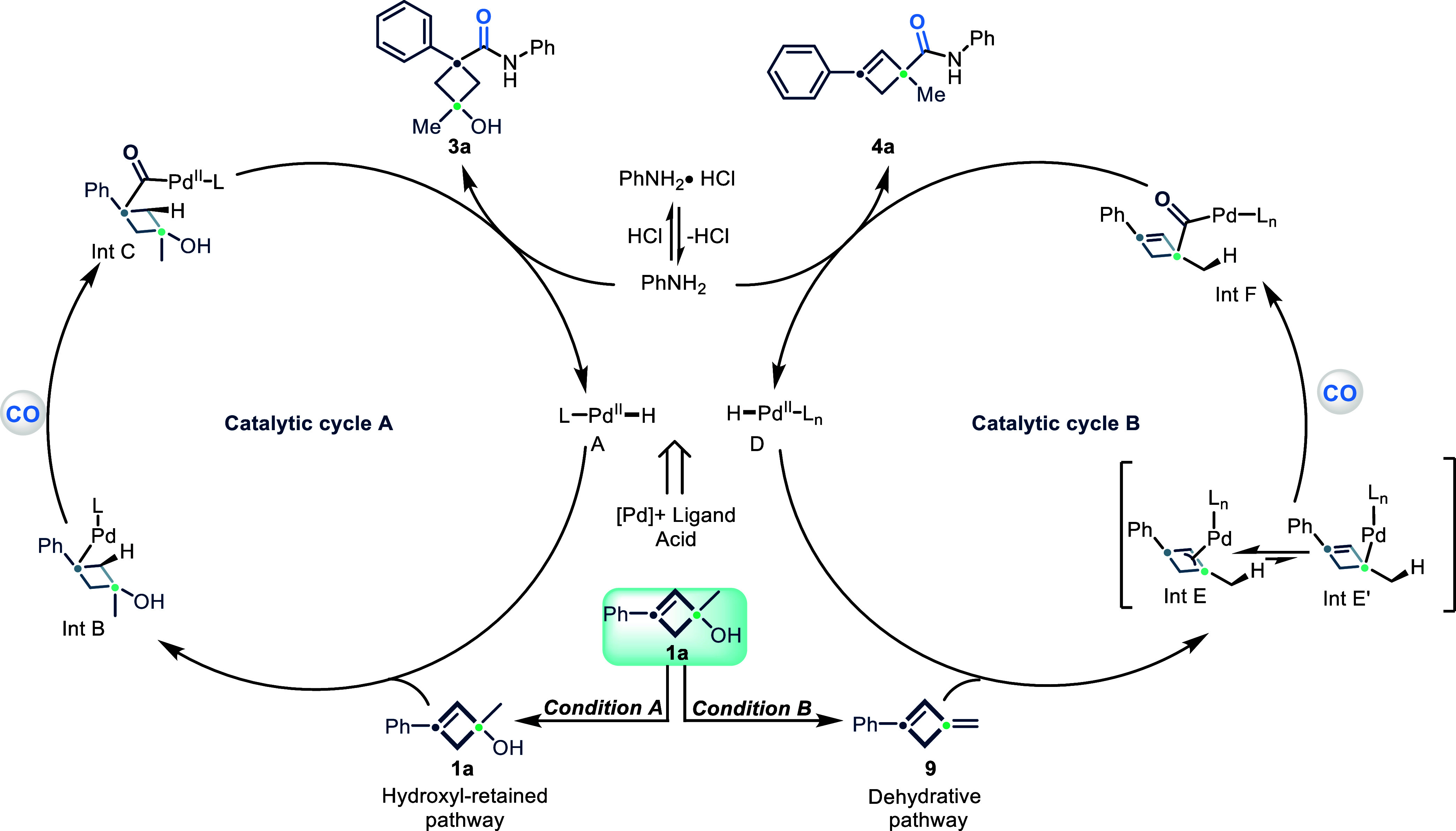
Proposed mechanism.

## Conclusions

In summary, this study establishes a condition-controlled strategy for the divergent carbonylation of cyclobutenols through precursor differentiation. By directing a common substrate into distinct reaction precursors prior to carbonylation, two structurally distinct classes of four-membered-ring amides can be accessed with high selectivity. The broad substrate scope and compatibility with pharmaceutical and biologically relevant molecules further highlight the synthetic utility of this transformation. The resulting cyclobutenamide products also serve as versatile platform intermediates for the selective synthesis of two different aza-bicyclic frameworks. Mechanistic studies support a condition-dependent precursor differentiation process as the origin of the divergent reactivity. Beyond the present system, this strategy may provide a useful perspective for the design of divergent catalytic transformations through precursor differentiation.

## Supplementary Material



## Notes

General comments, general procedure, optimization details, analytic data and NMR spectra (PDF)
