# Supplementary material for: Controlled Precursor Differentiation Enables Palladium-Catalyzed Divergent Carbonylation of Cyclobutenols
Source: J Am Chem Soc. 2026 Jul 17;148(29):31546–58. doi: 10.1021/jacs.6c11571 (PMC13426269; doi:10.1021/jacs.6c11571)
Supplement: Supplementary file 1 [file ja6c11571_si_001.pdf]

Supporting Information

Controlled Precursor Differentiation Enables Palladium-Catalyzed Divergent Carbonylation of Cyclobutenols

Yu-Kun Liu[a], Peng Yang[a], Hefei Yang[,b], Jiajun Zhang[a,b], Xiao-Feng Wu[a,b*]

[a]Leibniz-Institut fur Katalyse e. V., Albert-Einstein-Straße 29a, 18059 Rostock, Germany.
[b]Dalian National Laboratory for Clean Energy, Dalian Institute of Chemical Physics, Chinese Academy of Sciences, Dalian, 116023, Liaoning, China. E-mail: xwu2020@ dicp.ac.cn

Contents

1.General information 2
2. Optimization of Reaction Conditions........ 3
  2.1 Optimization of reaction conditions of **3a**........ 3
  2.2 Optimization of reaction conditions of **4a**........ 5
3. General Procedure for the Preparation of Cyclobutenols and Methylenecycloalkene 8
  3.1 List of tertiary cyclobutenols 8
  3.2 Typical procedure of tertiary cyclobutenols........ 8
  3.3 Typical procedure of **1q** 9
  3.3 Typical procedure of **9** 9
4. General Procedure for the Synthesis of **3** and **4** 10
  4.1 General procedure for the synthesis of **3** 10
  4.2 General procedure for the synthesis of **4** 10
5. Synthetic Applications........ 11
6. Mechanistic Studies........ 12
  6.1 Isotopic labeling studies 12
    6.1.1 Isotopic labeling studies of **3a** 12
    6.1.2 Isotopic labeling studies of **4a** 13
  6.2 Intermediate verification experiment 16
  6.3 Control experiments for the dehydration of cyclobutanol 17
  6.4 Hammett plot analysis 18
  6.5 Stability of **3a** under acidic conditions 20
7. Spectroscopic Data of Products 21
8. NMR Spectra of the Products 47
9. X-ray Crystallographic Data 181
10. Reference........183

1.General information

Reagents and solvents: Unless otherwise noted, reagents were ordered from *Sigma-Aldrich, TCI, ABCR, Alfa Aesar* or *BLD pharm*, and used without purification. Pure solvents was available from *Thermo Fisher*, and degassed (3 times) under argon atmosphere, then store under standard Schlenk technique (anhydrous and under inert atmosphere).

Purification: Analytical thin layer chromatography was performed using *MACHEREY-NAGEL Gmbn & Co. KG* silica gel plates (Silica gel 60 UV_{254}). Visualization was by ultraviolet fluorescence (λ = 254 nm) and/or staining with potassium permanganate ($KMnO_4$). The products were isolated from the reaction mixture by column chromatography on silica gel 60, 0.063-0.2 mm, 70-230 mesh (Merck). Gradient flash chromatography was conducted eluting with PE/EA, PE refers to pentane and EA refers to ethyl acetate, they were listed as volume/volume ratios.

Data collection: GC analysis was performed on an Agilent HP-7890A instrument with FID detector and HP-5 capillary column (polydimethylsiloxane with 5% phenyl groups, 30 m, 0.32 mm i.d., 0.25 µm film thickness) using argon as carrier gas. Electron impact (EI) mass spectra were recorded on AMD 402 mass spectrometer (70 eV). The data are given as mass units per charge (m/z). High resolution mass spectra (HRMS) were recorded on Agilent 6210. NMR spectra were recorded on Bruker Avance 300 and Bruker ARX 400 spectrometers. Multiplets were assigned as s(singlet), d (doublet), t (triplet), q (quartet), dd (doublet of doublet), m (multiplet) and br (broad). s (singlet). Chemical shifts (ppm) are given relative to solvent: references for $CDCl_3$ were 7.26 ppm (^{1}H NMR) and 77.00 ppm (^{13}C NMR). All measurements were carried out at room temperature unless otherwise stated.

NOTE: Because of the high toxicity of carbon monoxide, all the reactions should be performed in an autoclave. The laboratory should be well-equipped with a CO detector and alarm system.

2. Optimization of Reaction Conditions

2.1 Optimization of reaction conditions of **3a**

Table S1. Optimization of solvent

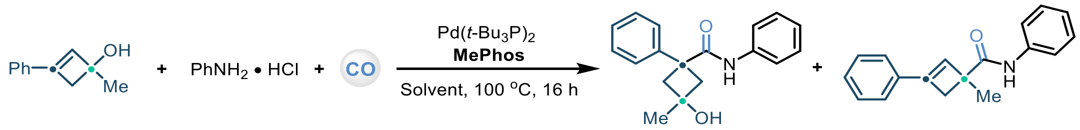

1a **2a** 40 bar **3a** **4a**

Entry	Solvent	Yield of **3a**[b] (%)	**3a**:**4a**
1	DCM	7	2:1
2	DCE	11	10:1
3	$CHCl_3$	4	1:1
4	THF	32	8:1
5[c]	THF	43	> 20:1
6[d]	THF	37	16:1
7	MeCN	trace	/
8	Toluene	20	12:1
9	Anisole	18	5:1
10	1,4-dioxane	29	6:1
11	PhCl	18	6:1
12	DME	26	4:1

[a] Pd(*t*-Bu$_3$P)$_2$ (5 mol%), **MePhos** (10 mol%), **1a** (0.12 mmol), **2a** (0.1 mmol), CO (40 bar), Solvent (1.0 mL) at 100 °C for 16 h. [b] The yield and **3a**:**4a** ratio were determined by GC with n-hexadecane as internal standard. [c] 90 °C. [d] 80 °C.

Table S2. Optimization of ligand

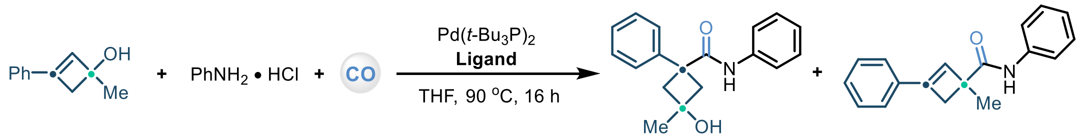

1a **2a** 40 bar **3a** **4a**

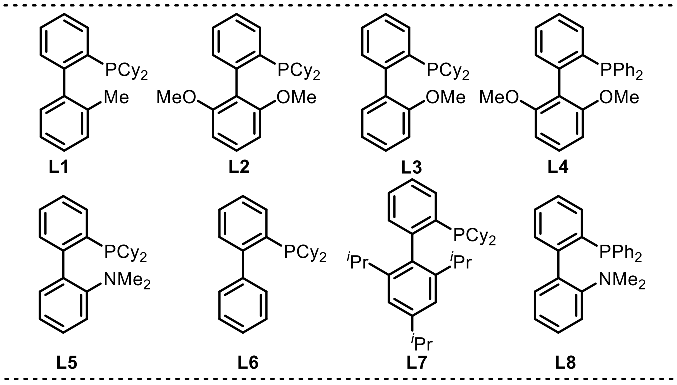

Entry	**Ligand**	Yield of **3a**[b] (%)	**3a**:**4a**
1	**L1**	40	> 20:1
2	**L2**	46	5:1
3	**L3**	44	5:1
4	**L4**	44	5:1
5	**L5**	50	> 20:1
6	**L6**	46	17:1
7	**L7**	38	3:1

8	**L8**	34	5:1

[a] $Pd(t\text{-}Bu_3P)_2$ (5 mol%), **Ligand** (10 mol%), **1a** (0.12 mmol), **2a** (0.1 mmol), CO (40 bar), THF (1.0 mL) at 90 °C for 16 h. [b] The yield and **3a**:**4a** ratio were determined by GC with n-hexadecane as internal standard.

Table S3. Optimization of catalyst

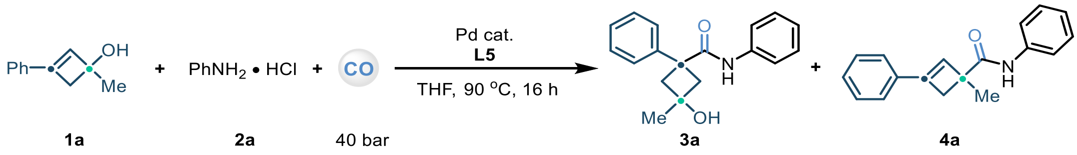

Entry	Pd cat.	Yield of **3a**[b] (%)	**3a**:**4a**
1	$Pd(OAc)_2$	20	7:1
2	$Pd(CH_3CN)Cl_2$	21	2:1
3	$PdCl_2$	18	1:1
4	$Pd(TFA)_2$	24	2:1
5	$Pd(COD)_2Cl_2$	23	2:1
6[c]	$[Pd(\pi\text{-cinnamyl})Cl]_2$	29	3:1
7	$Pd(PPh_3)_4$	26	2:1
8	$Pd_2(dBa)_3$	34	2:1
9	$Pd(t\text{-}Bu_3P)_2$	50	> 20:1

[a] Pd cat. (5 mol%), **L5** (10 mol%), **1a** (0.12 mmol), **2a** (0.1 mmol), CO (40 bar), THF (1.0 mL) at 90 °C for 16 h. [b] The yield and **3a**:**4a** ratio were determined by GC with n-hexadecane as internal standard. [c] 2.5 mol%

Table S4. Optimization the amount of **1a**

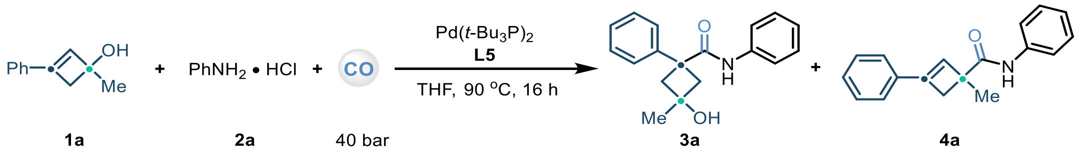

Entry	**1a** (mmol)	**2a** (mmol)	Yield of **3a**[b] (%)	**3a**:**4a**
1	0.12	0.1	50	> 20:1
2	0.15	0.1	57	> 20:1
3	0.2	0.1	84	> 20:1
4	0.25	0.1	68	> 20:1
5	0.1	0.12	49	> 20:1
6	0.1	0.15	42	> 20:1
7	0.1	0.2	39	17:1

[a] $Pd(t\text{-}Bu_3P)_2$ (5 mol%), **L5** (10 mol%), **1a**, **2a**, CO (40 bar), THF (1.0 mL) at 90 °C for 16 h. [b] The yield and **3a**:**4a** ratio were determined by GC with n-hexadecane as internal standard.

Table S5. Optimization of concentration

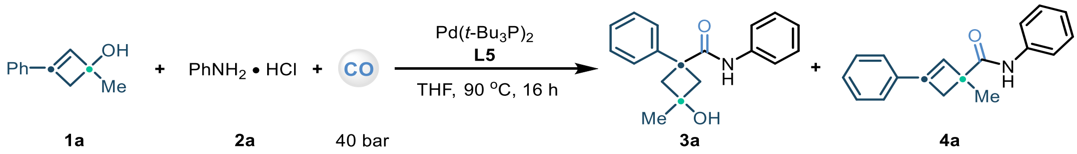

Entry	THF (mL)	Yield of **3a**[b] (%)	**3a**:**4a**
1	0.5	64	> 20:1
2	0.7	70	> 20:1
3	1.0	84	> 20:1
4	1.5	87	> 20:1
5	2.0	93	> 20:1

6	2.5	88	> 20:1

[a] Pd(t-Bu$_3$P)$_2$ (5 mol%), **L5** (10 mol%), **1a** (0.2 mmol), **2a** (0.1 mmol), CO (40 bar), THF at 90 °C for 16 h. [b] The yield and **3a**:**4a** ratio were determined by GC with n-hexadecane as internal standard.

Table S6. Optimization of the catalyst-ligand ratio

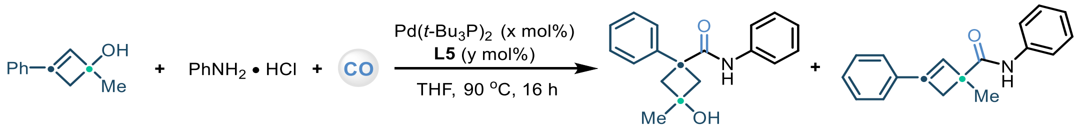

1a **2a** 40 bar **3a** **4a**

Entry	x (mol%)	y (mol%)	Yield of **3a**[b] (%)	**3a**:**4a**
1	2	4	53	> 20:1
2	5	10	93	> 20:1
3	5	7	98 (96)	> 20:1
4	5	5	95	> 20:1
5	5	3	85	> 20:1
6	5	w/o **L5**	49	> 20:1

[a] Pd(t-Bu$_3$P)$_2$ (x mol%), **L5** (y mol%), **1a** (0.2 mmol), **2a** (0.1 mmol), CO (40 bar), THF at 90 °C for 16 h. [b] The yield and **3a**:**4a** ratio were determined by GC with n-hexadecane as internal standard. Isolation yields in parentheses.

2.2 Optimization of reaction conditions of 4a

Table S1. Optimization of ligand

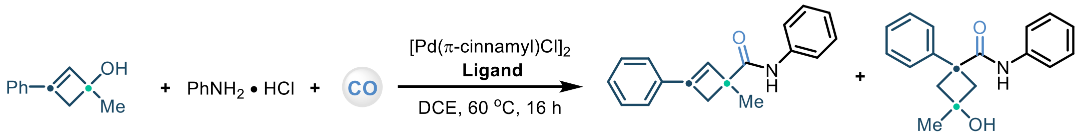

1a **2a** 40 bar **4a** **3a**

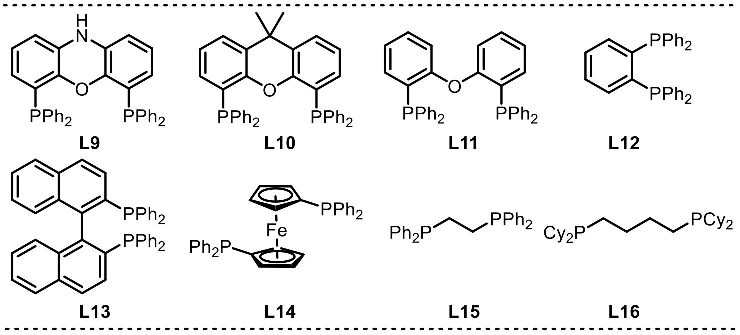

Entry	Ligand	Yield of **4a**[b] (%)	**4a**:**3a**
1	**L9**	36	9:1
2	**L10**	49 (44)	> 20:1
3	**L11**	44	7:1
4	**L12**	8	/
5	**L13**	9	/
6	**L14**	6	/
7	**L15**	9	3:1
8	**L16**	12	6:1

[a] [Pd(π-cinnamyl)Cl]$_2$ (2.5 mol%), **Ligand** (5 mol%), **1a** (0.12 mmol), **2a** (0.1 mmol), CO (40 bar), DCE (1.0 mL) at 60 °C for 16 h. [b] The yield and **4a**:**3a** ratio were determined by GC with n-hexadecane as internal standard. Isolation yields in parentheses.

Table S2. Optimization of catalyst

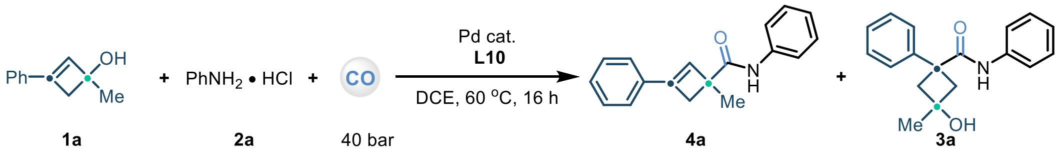

Entry	Pd cat.	Yield of **4a**[c] (%)	**4a**:**3a**
1[b]	[Pd(π-cinnamyl)Cl]$_2$	49 (44)	> 20:1
2	Pd(TFA)$_2$	25	> 20:1
3	PdCl$_2$	trace	/
4	Pd(OAc)$_2$	16	8:1
5	Pd(MeCN)$_2$Cl$_2$	trace	/
6	Pd(dBa)$_2$	13	2:1
7	Pd(acac)$_2$	16	4:1
8	Pd(*t*-Bu$_3$P)$_2$	trace	/
9	Pd(PPh$_3$)P$_4$	trace	/

[a] [Pd(π-cinnamyl)Cl]$_2$ (2.5 mol%) or Pd cat. (5 mol%), **Ligand** (5 mol%), **1a** (0.12 mmol), **2a** (0.1 mmol), CO (40 bar), DCE (1.0 mL) at 60 °C for 16 h. [b] [Pd(π-cinnamyl)Cl]$_2$ (2.5 mol%). [c] The yield and **4a**:**3a** ratio were determined by GC with n-hexadecane as internal standard. Isolation yields in parentheses.

Table S3. Optimization of the amount of **1a** and temperature

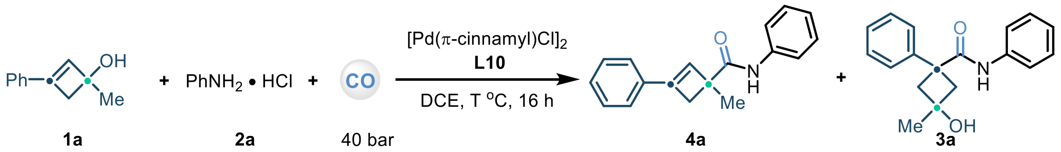

Entry	**1a** (mmol)	**2a** (mmol)	Temperature (°C)	Yield of **4a**[b] (%)	**4a**:**3a**
1	0.12	0.1	60	49 (44)	> 20:1
2	0.15	0.1	60	55	> 20:1
3	0.2	0.1	60	57	> 20:1
4	0.2	0.1	80	63 (62)	> 20:1
5	0.2	0.1	90	59	> 20:1
6	0.2	0.1	100	46	16:1
7	0.25	0.1	60	53	> 20:1
8	0.1	0.12	60	40	> 20:1
9	0.1	0.15	60	29	15:1
10	0.1	0.2	60	35	12:1

[a] [Pd(π-cinnamyl)Cl]$_2$ (2.5 mol%), **L10** (5 mol%), **1a**, **2a**, CO (40 bar), DCE (1.0 mL) at T °C for 16 h. [b] The yield and **4a**:**3a** ratio were determined by GC with n-hexadecane as internal standard. Isolation yields in parentheses.

Table S4. Optimization of concentration

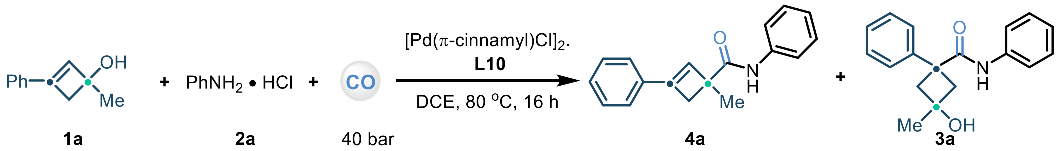

Entry	DCE (mL)	Yield of **4a**[b] (%)	**4a**:**3a**
1	0.5	36	9:1
2	0.7	54	> 20:1
3	1.0	63 (62)	> 20:1
4	1.5	64	> 20:1
5	2.0	72 (70)	> 20:1

6	2.5	74	> 20:1
7	3.0	75	> 20:1
8	3.5	77	> 20:1
9	4.0	75	> 20:1

[a]] [Pd(π-cinnamyl)Cl]$_2$ (2.5 mol%), **L10** (5 mol%), **1a** (0.2 mmol), **2a** (0.1 mmol), CO (40 bar), DCE at 80 °C for 16 h. [b] The yield and **4a**:**3a** ratio were determined by GC with n-hexadecane as internal standard. Isolation yields in parentheses.

Table S5. Optimization of the catalyst-ligand ratio, time and pressure

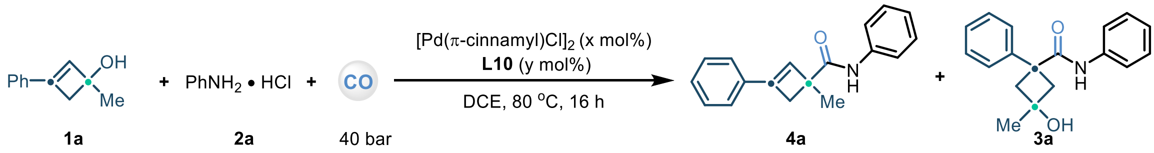

Entry	x (mol%)	y (mol%)	Yield of **4a**[b] (%)	**4a**:**3a**
1	1.5	3	51	17:1
2	2.5	5	77	> 20:1
3	5	10	80	> 20:1
4	5	12	82	> 20:1
5	5	15	84 (81)	> 20:1
6[c]	5	15	65	> 20:1
5[d]	5	15	71	> 20:1
8[e]	5	15	75	> 20:1
6	5	20	83	> 20:1
7	7.5	15	79	> 20:1
8	10	20	74	> 20:1

[a] [Pd(π-cinnamyl)Cl]$_2$ (x mol%), **L10** (y mol%), **1a** (0.2 mmol), **2a** (0.1 mmol), CO (40 bar), DCE (3.5 mL) at 80 °C for 16 h. [b] The yield and **4a**:**3a** ratio were determined by GC with n-hexadecane as internal standard. Isolation yields in parentheses. [c] 20 h. [d] 30 bar. [e] 14 h.

3. General Procedure for the Preparation of Cyclobutenols and Methylenecycloalkene

3.1 List of tertiary cyclobutenols

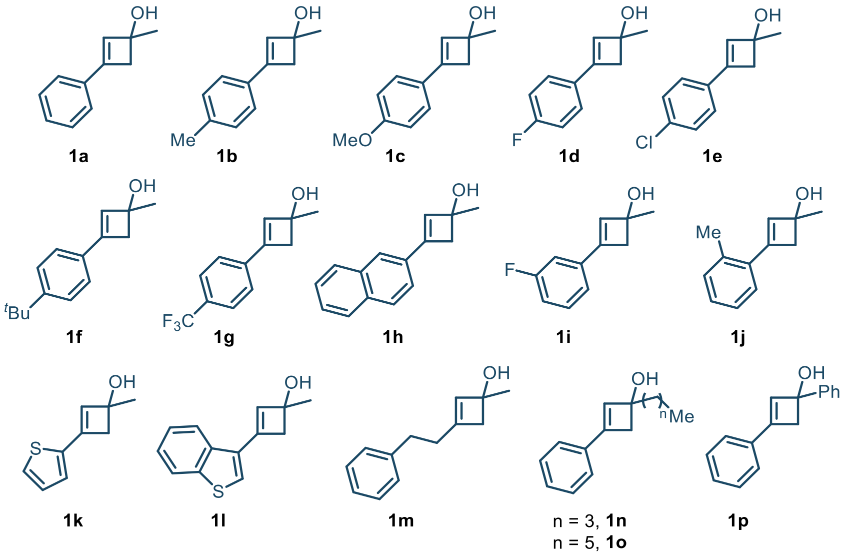

All alkenyl cyclobutanols **1a-1o** were prepared according to the reported procedure and their characterization data match the reported data.[1]

3.2 Typical procedure of tertiary cyclobutenols

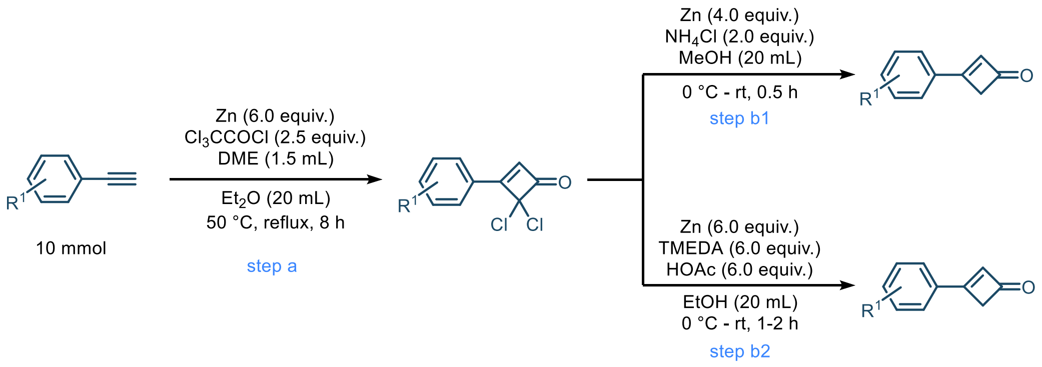

To a flame-dried 100 mL three-necked flask equipped with a magnetic stir bar were charged zinc dust (3.9 g, 60 mmol), Et_2O (20 mL), DME (1.4 g, 1.5 mL, 15 mmol), Cl_3CCOCl (4.5 g, 2.8 mL, 25 mmol), and the corresponding alkyne (10.0 mmol) under a nitrogen atmosphere. The flask was sealed and stirred at 45 °C for 8 h. After cooling to room temperature, the reaction mixture was filtered through Celite and rinsed with Et_2O. The combined organic layers were washed successively with 0.5 M HCl, 5% NaOH solution, and brine, dried over $MgSO_4$, filtered, and concentrated. The crude product was purified by column chromatography on silica gel (pentane/ ethyl acetate = 30:1 → 20:1) to afford the dichlorocyclobutenone (step a).

To a mixture of zinc dust (2.6 g, 40 mmol), NH_4Cl (1.1 g, 20 mmol), and MeOH (5 mL) at 0 °C was added the indicated dichlorocyclobutenone (10 mmol) in MeOH (15 mL) dropwise. The reaction mixture was stirred at 0 °C for 20 min and then at room temperature for 10 min until full conversion was indicated by TLC. The mixture was filtered through Celite and concentrated. The residue was washed with saturated aqueous $NaHCO_3$ and brine, extracted with DCM, dried over $MgSO_4$, concentrated, and purified by column chromatography (PE/EA = 20:1 → 15:1) to give the corresponding cyclobutenones. Precursors **1a**–**1d**, **1i**, and **1n**–**1o** were synthesized by this method (step b1).

To a mixture of zinc dust (3.9 g, 60 mmol), TMEDA (7.0 g, 9.0 mL, 60 mmol), and EtOH (10 mL) at 0 °C was added HOAc (3.6 g, 3.4 mL, 60 mmol) dropwise. The indicated dichlorocyclobutenone (10 mmol) in EtOH (10 mL) was added dropwise via an addition funnel over 0.5 h. The reaction mixture was stirred at 0 °C for 20 min and then at room temperature for 1–2 h until full conversion was observed by TLC. The mixture was filtered through Celite and

concentrated. The residue was washed with 0.5 M HCl and extracted with MeOtBu. The combined organic layers were washed with saturated aqueous $NaHCO_3$ and brine, dried over $MgSO_4$, concentrated, and purified by column chromatography (pentane/ ethyl acetate =15:1 → 10:1) to afford the cyclobutenones. Precursors **1e**-**1h**, and **1j**-**1m** were obtained using this method (step b2).

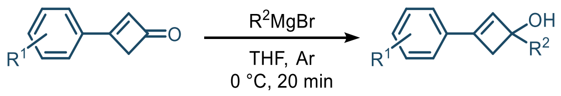

Cyclobutenonols **1a**–**1p** were prepared according to the following procedure. To a solution of the corresponding cyclobutenone (10 mmol) in THF (30 mL) at 0 °C under a nitrogen atmosphere was added the appropriate Grignard reagent (MeMgBr: 3.0 M in Et_2O, 3.5 mL; $^{n}C_4H_9MgBr$, $^{n}C_6H_{13}MgBr$, or PhMgBr: 1.0 M in THF, 15 mL). The reaction mixture was stirred at 0 °C for 20 min and then quenched with saturated aqueous NH_4Cl. The organic layer was washed with brine, extracted with EA, dried over $MgSO_4$, filtered, and concentrated. The residue was purified by silica gel column chromatography (pentane/ ethyl acetate = 10:1 → 5:1) to afford the corresponding cyclobutenonols.

3.3 Typical procedure of **1q**

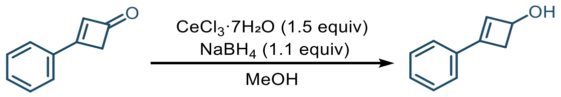

Cyclobutenone **1q** were prepared according to a known procedure.[2] To a solution of 3-phenylcyclobutenone (5 mmol) and $CeCl_3{\cdot}7H_2O$ (7.5 mmol) in MeOH (15 mL) at room temperature was slowly added $NaBH_4$ (5.5 mmol) over 15 min. The reaction mixture was stirred at the same temperature for 5 min, then quenched with brine (30 mL) and extracted with Et_2O (3 × 30 mL). The combined organic layers were dried over $MgSO_4$, filtered, and concentrated under reduced pressure. The residue was purified by silica gel column chromatography (pentane/ ethyl acetate = 10:1 → 5:1) to afford the product **1p** (532.9 mg, 73% yield, pale yellow solid.).

3.3 Typical procedure of **9**

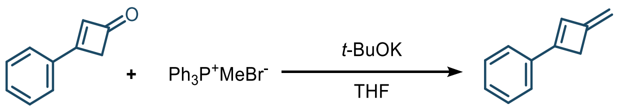

9

Methylenecycloalkenes **9** were prepared using conditions modified from a reported procedure.[3] A solution of methyl methyltriphenylphosphonium bromide (11.8 g, 33.0 mmol) and *t*-BuOK (3.74 g, 30.0 mmol) in THF (30 mL) was stirred at 0°C for 1 h before a solution of 3-phenylcyclobutenone (1.44 g, 10.0mmol) in THF (10 mL) was added. The reaction mixture was then stirred at 0 °C for 1 h. The reaction was quenched with H_2O and extracted with DCM. The combined organic phase was dried over Na_2SO_4, filtered, concentrated, and the residue was purified by column chromatography (pentane) to afford the pure product **9** (752.6 mg, 53% yield, colorless oil).

4. General Procedure for the Synthesis of **3** and **4**

4.1 General procedure for the synthesis of **3**

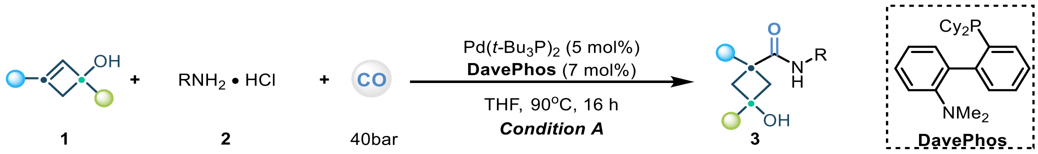

Condition A: A vial (4 mL) was charged with Pd(t-Bu$_3$P)$_2$ (5 mol%), DavePhos (7 mol%), cyclobutenol (**1**, 2.0 equiv, 0.2 mmol), amine hydrochloride (**2**, 1.0 equiv., 0.1 mmol) and a stirring bar. The vial was closed by PTFE/white rubber septum (Wheaton 13 mm Septa) and phenolic cap and connected with atmosphere with a needle. The vial was evacuated under vacuum and recharged with argon for three times. After that, THF (2.0 mL) was added with a syringe under nitrogen atmosphere. Subsequently, the vial (or several vials) was placed in an alloy plate, which was transferred into a 300 mL autoclave of the 4560 series from Parr Instruments. After flushing the autoclave three times with CO, a pressure of 40 bar of CO was adjusted at ambient temperature. Then, the reaction was performed for 16 h at 90 °C. After the reaction was complete, the autoclave was cooled down with ice water to room temperature and the pressure was released carefully. The solution was concentrated in vacuo then purified by silica-gel column chromatography using pentane and ethyl acetate to afford the corresponding product **3.**

4.2 General procedure for the synthesis of **4**

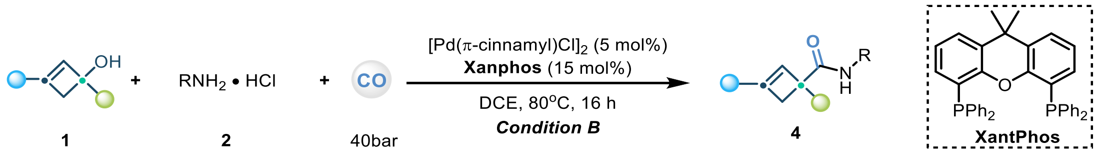

***Condition B**:* A vial (8 mL) was charged with [Pd(π-cinnamyl)Cl]$_2$ (5 mol%), XantPhos(15 mol%), ,cyclobutenol (**1**, 2 equiv., 0.2 mmol) amine hydrochloride (**2**, 1.0 equiv, 0.1 mmol) and a stirring bar. The vial was closed by PTFE/white rubber septum (Wheaton 13 mm Septa) and phenolic cap and connected with atmosphere with a needle. The vial was evacuated under vacuum and recharged with argon for three times. After that, DCE (3.5 mL), was added with a syringe under nitrogen atmosphere. Subsequently, the vial (or several vials) was placed in an alloy plate, which was transferred into a 300 mL autoclave of the 4560 series from Parr Instruments. After flushing the autoclave three times with CO, a pressure of 40 bar of CO was adjusted at ambient temperature. Then, the reaction was performed for 16 h at 80 °C. After the reaction was complete, the autoclave was cooled down with ice water to room temperature and the pressure was released carefully. The solution was concentrated in vacuo then purified by silica-gel column chromatography using pentane and ethyl acetate to afford the corresponding product **4**.

5. Synthetic Applications

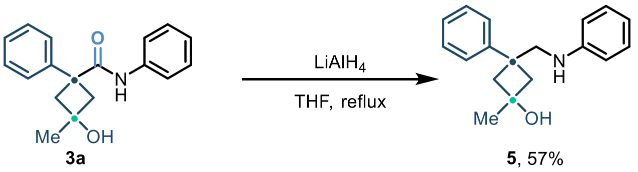

In an oven-dried 10 mL Schlenk tube, $LiAlH_4$ (22.7 mg, 3.0 equiv, 0.6 mmol) was added to a solution of **3a** (56.2 mg, 1.0 equiv, 0.2 mmol) in THF (2.0 mL) at 0 °C. The reaction mixture was then heated to reflux and stirred for 8 h. After cooling to room temperature, the reaction was quenched with water, and the mixture was adjusted to basic conditions by the addition of NaOH (4 M). The resulting mixture was extracted with ethyl acetate (10 mL × 3), and the combined organic layers were concentrated under reduced pressure. The crude product was purified by column chromatography (pentane/ ethyl acetate = 5:1) to remove impurities, affording compound **5** (30.1 mg, 57% yield) as a pale yellow solid.

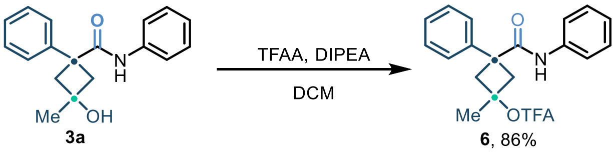

In an oven-dried 10 mL Schlenk tube equipped with a magnetic stir bar were added **3a** (28.1 mg, 0.1 mmol, 1.0 equiv), DIPEA (28.5 mg, 39 μL, 2.2 equiv), and DCM (1.0 mL). The reaction tube was then sealed and cooled to 0 °C. TFAA (42.0 mg, 28 μL, 2.0 equiv) was added, and the reaction mixture was stirred at 0°C for 5 h. The reaction mixture was then diluted with DCM (10 mL) and quenched with water (10 mL). The aqueous layer was extracted with DCM (5 mL × 2). The combined organic layers were washed with 1 M HCl (10 mL), 1 M NaOH (10 mL), and brine (10 mL), dried over anhydrous Na_2SO_4, and concentrated under reduced pressure. The crude product was purified by column chromatography (pentane/ ethyl acetate = 20:1) to remove impurities, affording compound **6** (30.5 mg, 81% yield) as a white solid.

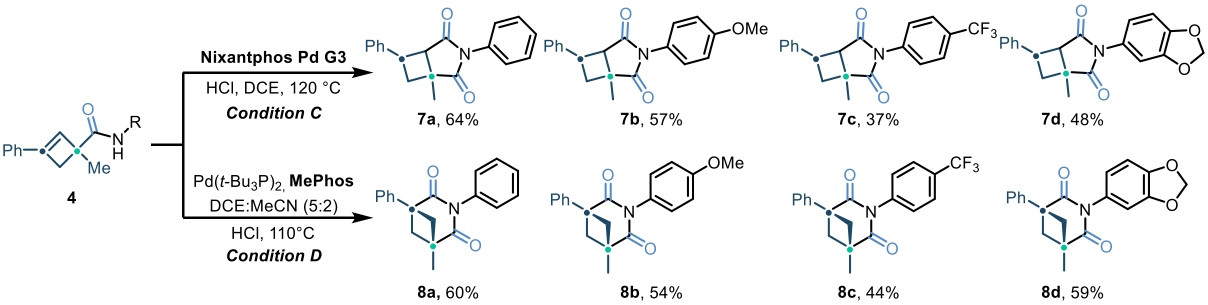

Condition C: A vial (4 mL) was charged with NiXantphos Pd G3 (5 mol%), **4** (1.0 equiv., 0.2 mmol) and a stirring bar. The vial was closed by PTFE/white rubber septum (Wheaton 13 mm Septa) and phenolic cap and connected with atmosphere with a needle. The vial was evacuated under vacuum and recharged with argon for three times. After that, DCE (0.7 mL) and 2 equiv. HCl (4 M in 1,4-dioxane, 100 μL), were added with a syringe under nitrogen atmosphere. Subsequently, the vial (or several vials) was placed in an alloy plate, which was transferred into a 300 mL autoclave of the 4560 series from Parr Instruments. After flushing the autoclave three times with CO, a pressure of 40 bar of CO was adjusted at ambient temperature. Then, the reaction was performed for 24 h at 120 °C. After the reaction was complete, the autoclave was cooled down with ice water to room temperature and the pressure was released carefully. The solution was concentrated in vacuo then purified by silica-gel column chromatography using pentane and ethyl acetate to afford the corresponding product **7.**

Condition D: A vial (4 mL) was charged with Pd(t-$Bu_3P)_2$ (5 mol%), MePhos (15 mol%), **4** (1 equiv., 0.2 mmol), and a stirring bar. The vial was closed by PTFE/white rubber septum (Wheaton 13 mm Septa) and phenolic cap and connected with atmosphere with a needle. The vial was evacuated under vacuum and recharged with argon for three times. After that, DCE (1.0 mL), MeCN (0.4 mL) and 2 equiv. HCl (4 M in 1,4-dioxane, 100 μL), were added with a syringe under nitrogen atmosphere. Subsequently, the vial (or several vials) was placed in an alloy plate, which was transferred into a 300 mL autoclave of the 4560 series from Parr Instruments. After flushing the autoclave three times with CO, a pressure of 50 bar of CO was adjusted at ambient temperature. Then, the reaction was performed for 24 h at 110 °C. After the reaction was complete, the autoclave was cooled down with ice water to room temperature and the pressure was released carefully. The solution was concentrated in vacuo then purified by silica-gel column chromatography using pentane and ethyl acetate to afford the corresponding product **8**.

6. Mechanistic Studies

6.1 Isotopic labeling studies

6.1.1 Isotopic labeling studies of **3a**

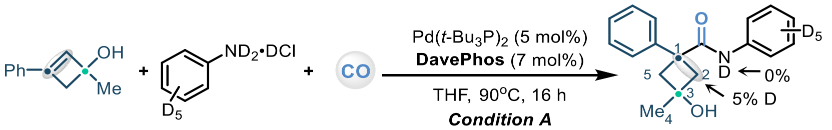

1a (2.0 eq.) **2a-*d*** 40bar **3a-*d1***, 90%

A vial (4 mL) was charged with Pd(t-Bu$_3$P)$_2$ (5 mol%), DavePhos (7 mol%), cyclobutenol (**1**, 2.0 equiv, 0.2 mmol), aniline hydrochloride-*d8* (**2a-*d***, 1.0 equiv., 0.1 mmol) and a stirring bar. The vial was closed by PTFE/white rubber septum (Wheaton 13 mm Septa) and phenolic cap and connected with atmosphere with a needle. The vial was evacuated under vacuum and recharged with argon for three times. After that, THF (2.0 mL) was added with a syringe under nitrogen atmosphere. Subsequently, the vial (or several vials) was placed in an alloy plate, which was transferred into a 300 mL autoclave of the 4560 series from Parr Instruments. After flushing the autoclave three times with CO, a pressure of 40 bar of CO was adjusted at ambient temperature. Then, the reaction was performed for 16 h at 90 °C. After the reaction was complete, the autoclave was cooled down with ice water to room temperature and the pressure was released carefully. The solution was concentrated in vacuo then purified by silica-gel column chromatography using pentane and ethyl acetate to afford the corresponding product **3a-*d1*.**

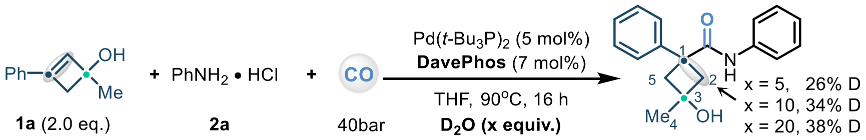

1a (2.0 eq.) **2a** 40bar

x = 5, **3a-*d2***, 81%
x = 10, **3a-*d3***, 68%
x = 20, **3a-*d4***, 63%

A vial (4 mL) was charged with Pd(t-Bu$_3$P)$_2$ (5 mol%), DavePhos (7 mol%), cyclobutenol (**1**, 2.0 equiv, 0.2 mmol), aniline hydrochloride (**2a,** 1.0 equiv., 0.1 mmol) and a stirring bar. The vial was closed by PTFE/white rubber septum (Wheaton 13 mm Septa) and phenolic cap and connected with atmosphere with a needle. The vial was evacuated under vacuum and recharged with argon for three times. After that, THF (2.0 mL) and D_2O (x equiv) were added with a syringe under nitrogen atmosphere. Subsequently, the vial (or several vials) was placed in an alloy plate, which was transferred into a 300 mL autoclave of the 4560 series from Parr Instruments. After flushing the autoclave three times with CO, a pressure of 40 bar of CO was adjusted at ambient temperature. Then, the reaction was performed for 16 h at 90 °C. After the reaction was complete, the autoclave was cooled down with ice water to room temperature and the pressure was released carefully. The solution was concentrated in vacuo then purified by silica-gel column chromatography using pentane and ethyl acetate to afford the corresponding product **3a-*d2-4*.**

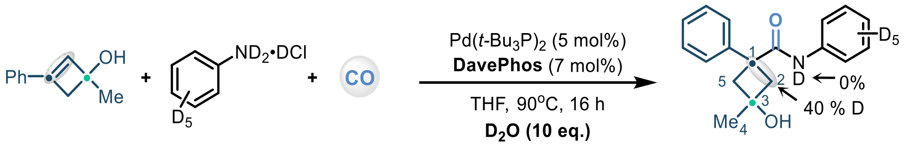

1a (2.0 eq.) **2a-*d*** 40bar **3a-*d5***, 59%

A vial (4 mL) was charged with Pd(t-Bu$_3$P)$_2$ (5 mol%), DavePhos (7 mol%), aniline hydrochloride-*d8* (**2a,** 1.0 equiv., 0.1 mmol), cyclobutenol (**1**, 2.0 equiv, 0.2 mmol) and a stirring bar. The vial was closed by PTFE/white rubber septum (Wheaton 13 mm Septa) and phenolic cap and connected with atmosphere with a needle. The vial was evacuated under vacuum and recharged with argon for three times. After that, THF (2.0 mL) and D_2O (10 equiv) were added with a syringe under nitrogen atmosphere. Subsequently, the vial (or several vials) was placed in an alloy plate, which was transferred into a 300 mL autoclave of the 4560 series from Parr Instruments. After flushing the autoclave three times with CO, a pressure of 40 bar of CO was adjusted at ambient temperature. Then, the reaction was performed for 16 h at 90 °C. After the reaction was complete, the autoclave was cooled down with ice water to room temperature and the pressure was released carefully. The solution was concentrated in vacuo then purified by silica-gel column chromatography using pentane and ethyl acetate to afford the corresponding product **3a-*d5*.**

6.1.2 Isotopic labeling studies of **4a**

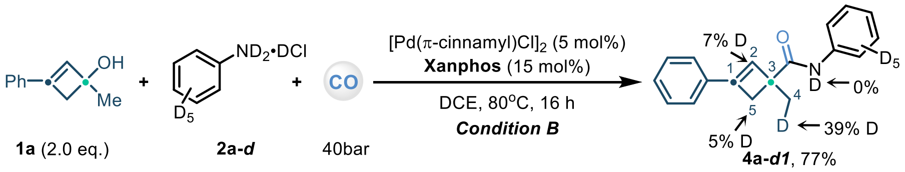

A vial (8 mL) was charged with [Pd(π-cinnamyl)Cl]$_2$ (5 mol%), XantPhos(15 mol%), cyclobutenol (**2**, 2 equiv., 0.2 mmol), amine hydrochloride-d8 (**2**, 1.0 equiv., 0.1 mmol) and a stirring bar. The vial was closed by PTFE/white rubber septum (Wheaton 13 mm Septa) and phenolic cap and connected with atmosphere with a needle. The vial was evacuated under vacuum and recharged with argon for three times. After that, DCE (3.5 mL), was added with a syringe under nitrogen atmosphere. Subsequently, the vial (or several vials) was placed in an alloy plate, which was transferred into a 300 mL autoclave of the 4560 series from Parr Instruments. After flushing the autoclave three times with CO, a pressure of 40 bar of CO was adjusted at ambient temperature. Then, the reaction was performed for 16 h at 80 °C. After the reaction was complete, the autoclave was cooled down with ice water to room temperature and the pressure was released carefully. The solution was concentrated in vacuo then purified by silica-gel column chromatography using pentane and ethyl acetate to afford the corresponding product ***4a-d1***.

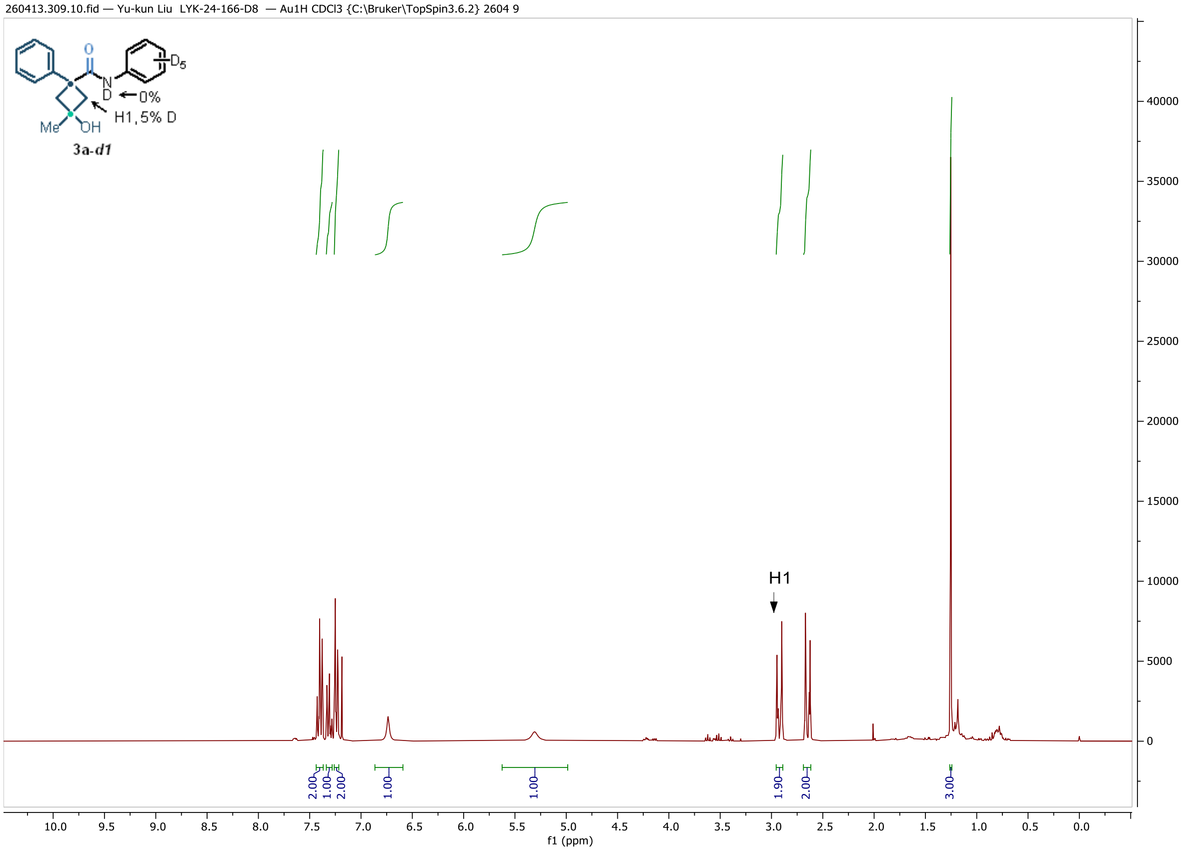

260422.f315.10.fid — Yu-kun Liu LYK-24-168 — Au1H CDCl3 {C:\Bruker\TopSpin3.6.2} 2604 15

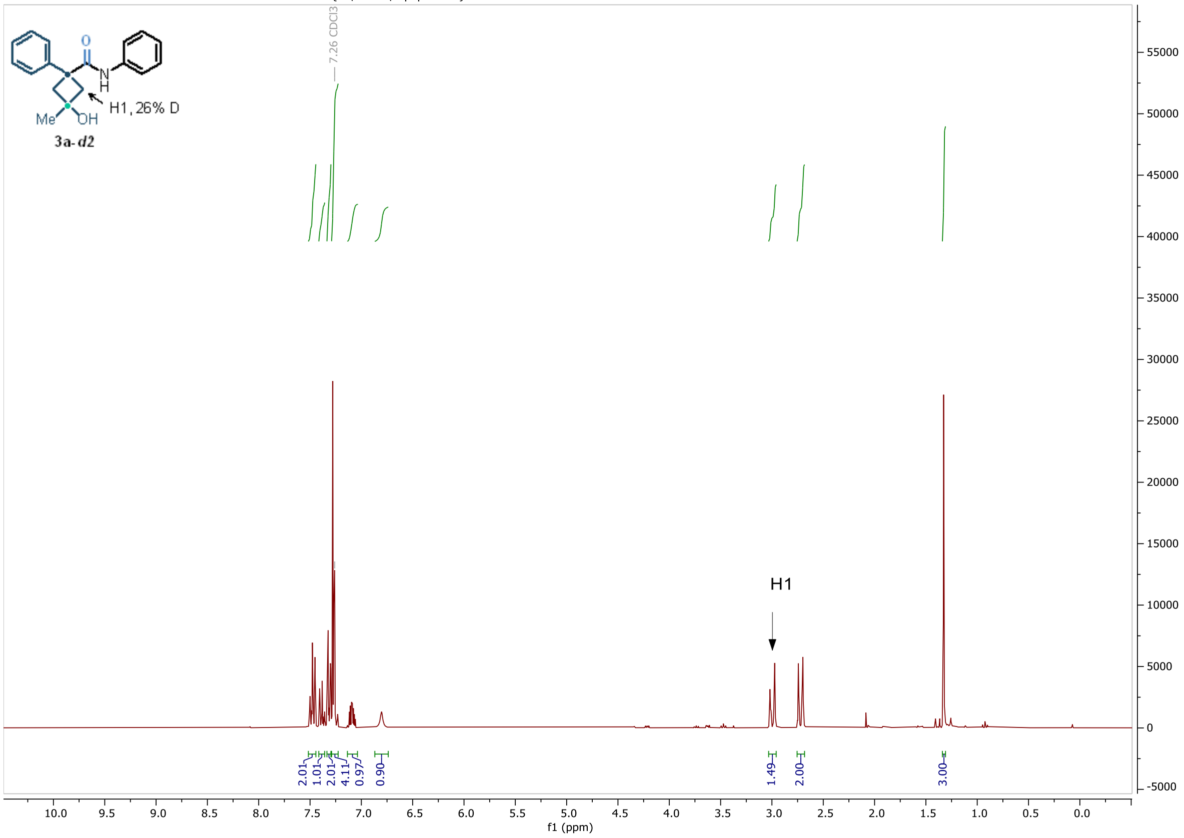

260508.306.10.fid — Yu-kun Liu LYK-24-177 — Au1H CDCl3 {C:\Bruker\TopSpin3.6.2} 2605 6

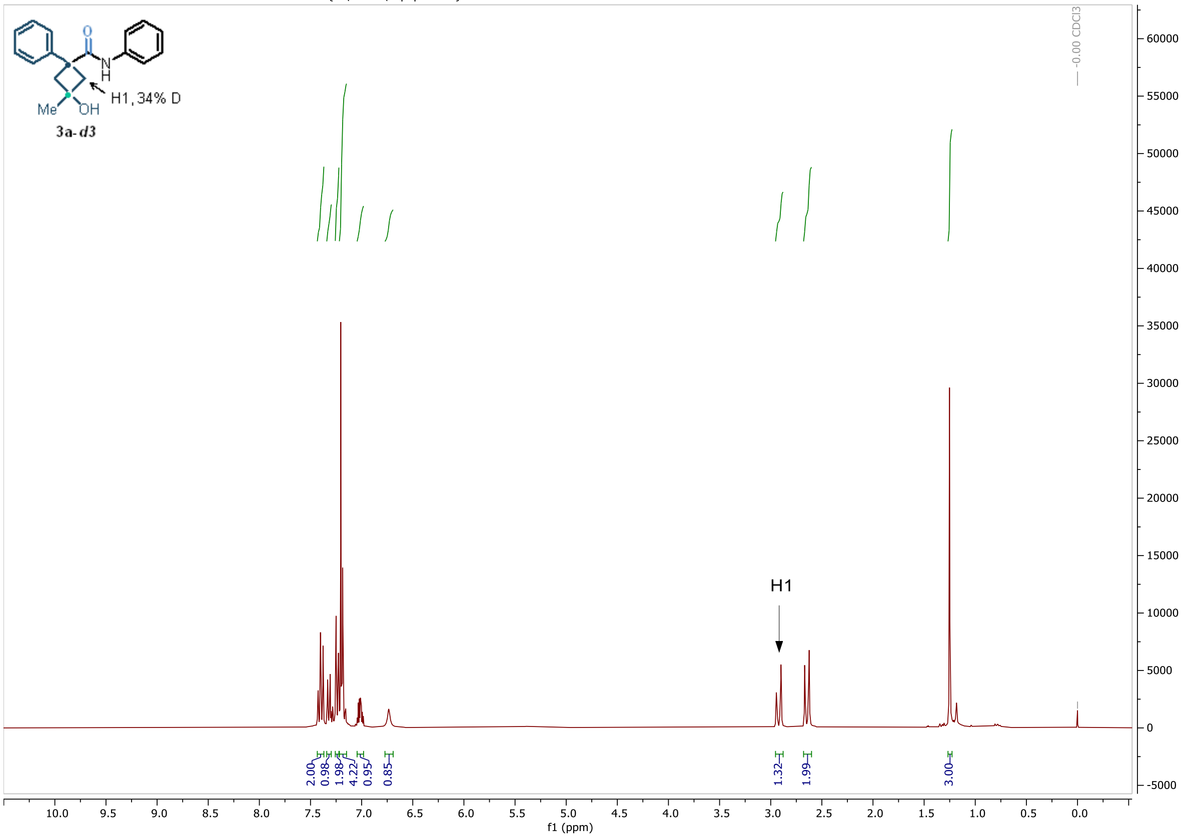

260508.307.10.fid — Yu-kun Liu LYK-24-178 — Au1H CDCl3 {C:\Bruker\TopSpin3.6.2} 2605 7

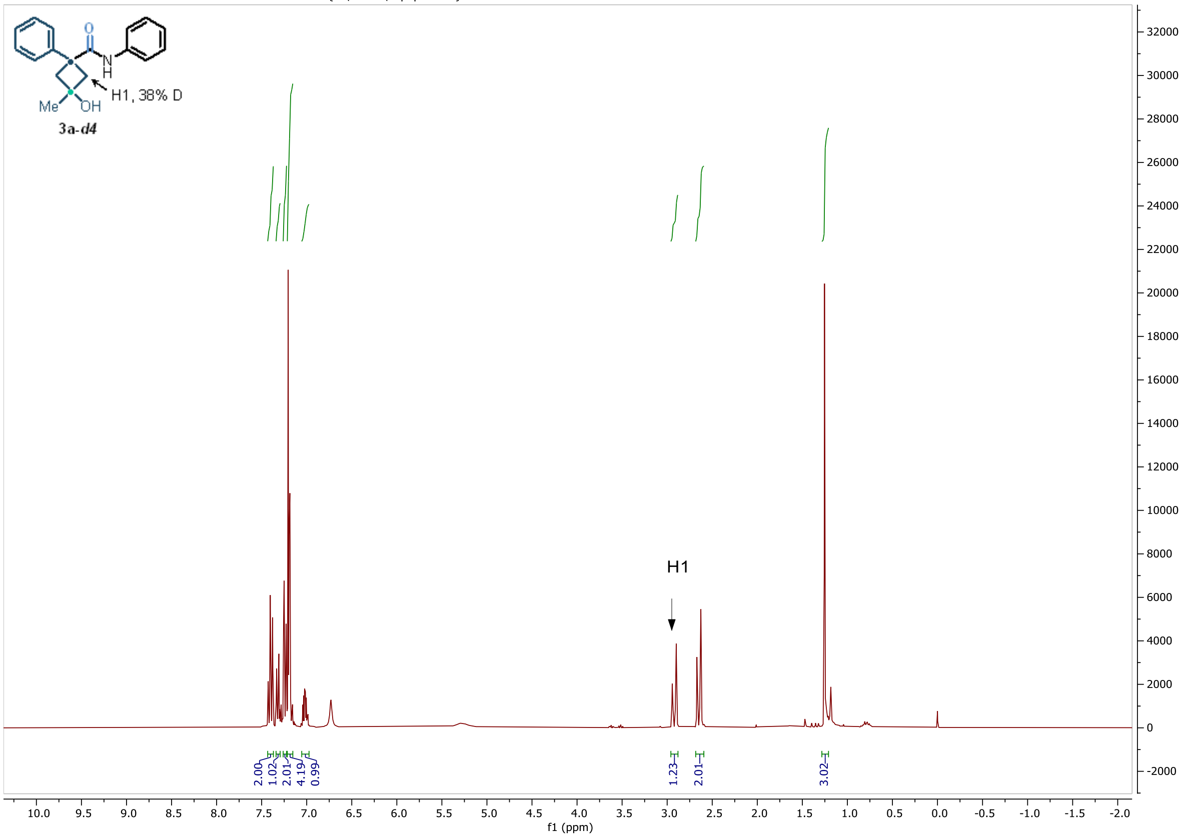

260512.310.10.fid — Yu-kun Liu LYK-24-1-180 — Au1H CDCl3 {C:\Bruker\TopSpin3.6.2} 2605 10

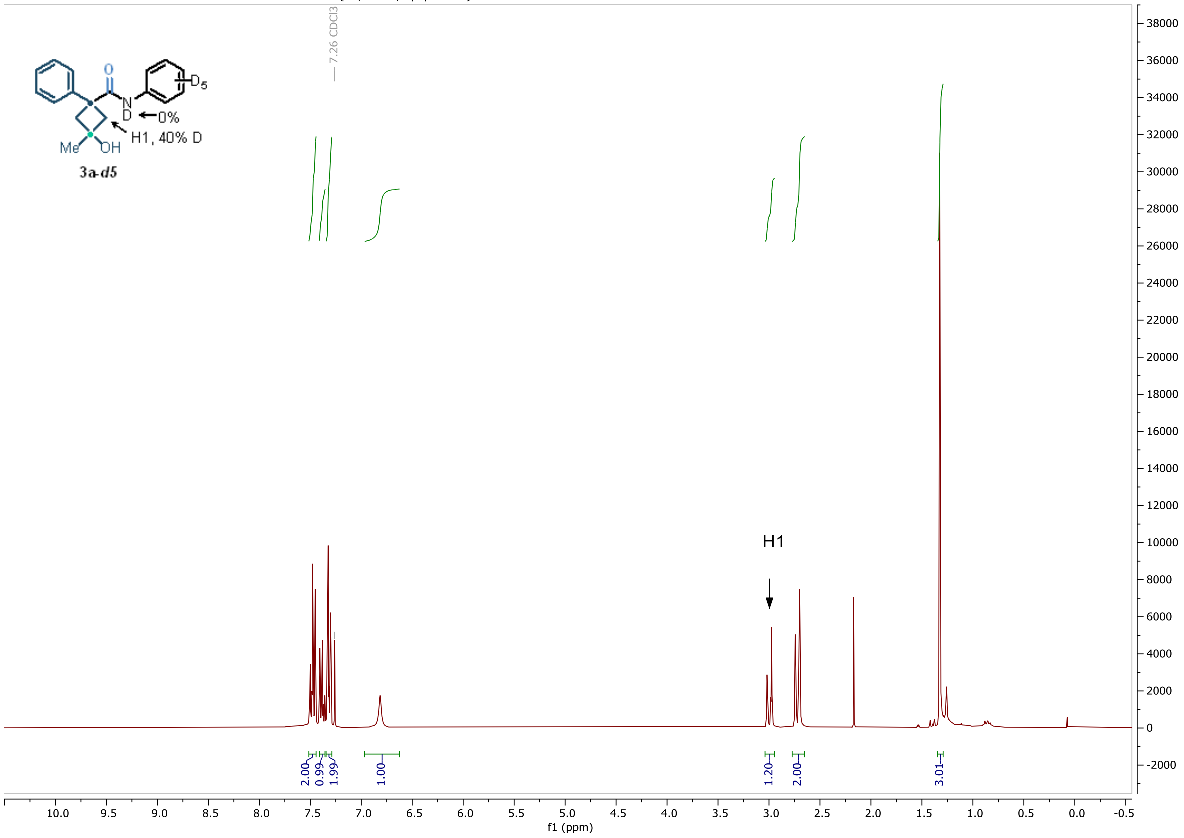

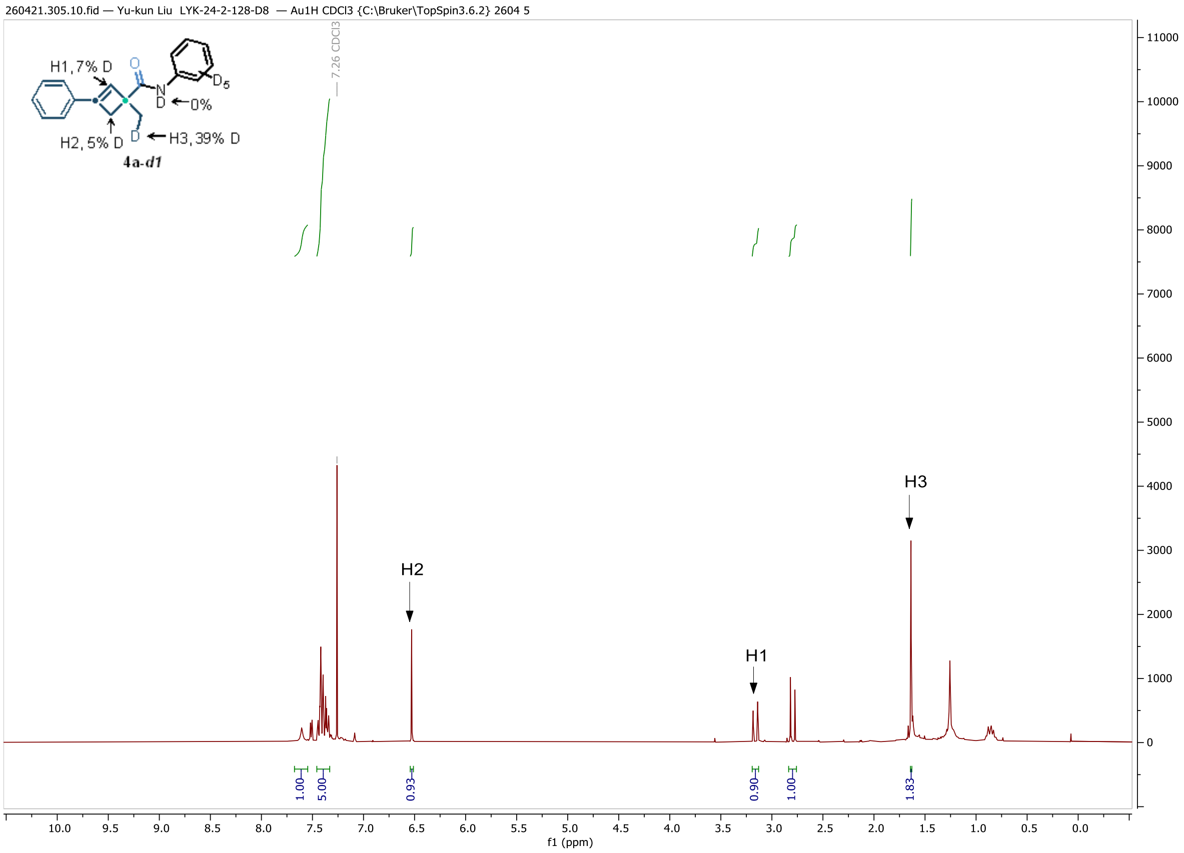

6.2 Intermediate verification experiment

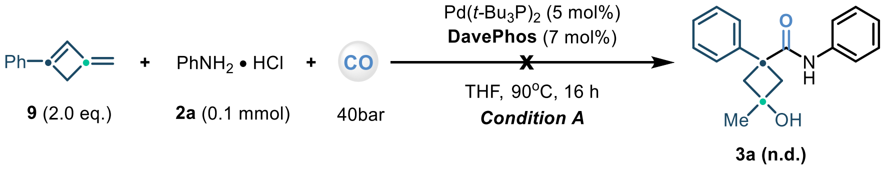

Condition A: A vial (4 mL) was charged with Pd(*t*-Bu$_3$P)$_2$ (5 mol%), DavePhos (7 mol%), amine hydrochloride (**2a**, 1.0 equiv., 0.1 mmol) and a stirring bar. The vial was closed by PTFE/white rubber septum (Wheaton 13 mm Septa) and phenolic cap and connected with atmosphere with a needle. The vial was evacuated under vacuum and recharged with argon for three times. After that, methylenecycloalkenes **9** (2.0 equiv, 0.2 mmol) and THF (2.0 mL) were added with a syringe under nitrogen atmosphere. Subsequently, the vial (or several vials) was placed in an alloy plate, which was transferred into a 300 mL autoclave of the 4560 series from Parr Instruments. After flushing the autoclave three times with CO, a pressure of 40 bar of CO was adjusted at ambient temperature. Then, the reaction was performed for 16 h at 90 °C. After the reaction was complete, the autoclave was cooled down with ice water to room temperature and the pressure was released carefully. The reaction mixture was concentrated in vacuo, and analysis of the crude mixture by GC-MS and ^{1}H NMR spectroscopy indicated that product **3a** was not formed.

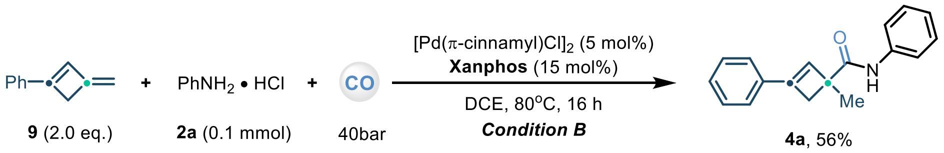

Condition B: A vial (8 mL) was charged with [Pd(π-cinnamyl)Cl]$_2$ (5 mol%), XantPhos(15 mol%), amine hydrochloride (**2a**, 1.0 equiv, 0.1 mmol) and a stirring bar. The vial was closed by PTFE/white rubber septum (Wheaton 13 mm Septa) and phenolic cap and connected with atmosphere with a needle. The vial was evacuated under vacuum and recharged with argon for three times. After that, methylenecycloalkenes **9** (2.0 equiv, 0.2 mmol) and DCE (3.5 mL) were added with a syringe under nitrogen atmosphere. Subsequently, the vial (or several vials) was placed in an alloy plate, which was transferred into a 300 mL autoclave of the 4560 series from Parr Instruments. After flushing the autoclave three times with CO, a pressure of 40 bar of CO was adjusted at ambient temperature. Then, the reaction was performed for 16 h at 80 °C. After the reaction was complete, the autoclave was cooled

down with ice water to room temperature and the pressure was released carefully. The solution was concentrated in vacuo then purified by silica-gel column chromatography using pentane and ethyl acetate to afford the corresponding product **4a**.

6.3 Control experiments for the dehydration of cyclobutanol

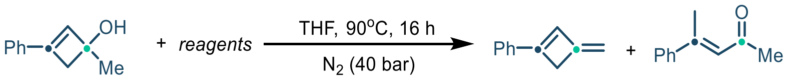

1a (0.1 mmol) **9** **10**

A vial (4 mL) equipped with a magnetic stirring bar was charged with cyclobutenol **1a** (1.0 equiv, 0.1 mmol) and the *corresponding reagent*. The vial was sealed with a PTFE/white rubber septum (Wheaton 13 mm septa) and a phenolic cap and connected to the atmosphere through a needle. The vial was evacuated and backfilled with argon three times. THF (2.0 mL) was then added via syringe under a nitrogen atmosphere. Subsequently, the vial (or several vials) was placed in an alloy plate, which was transferred into a 300 mL autoclave (Parr Instruments, Series 4560). After the autoclave was flushed three times with N_2, it was pressurized to 40 bar with N_2 at ambient temperature. The reaction mixture was then stirred at 90 °C for 16 h. After completion of the reaction, the autoclave was cooled to room temperature using an ice-water bath, and the pressure was carefully released. The yields of **9** and **10** were determined by GC analysis using n-hexadecane as an internal standard, and the identities of the products were confirmed by GC-MS and NMR analysis. See following table.

Entry	*reagents*	Conv.	Yield of **7**	Yield of **8**
1	$PhNH_2$ HCl (1 equiv)	87%	N.D.	79%
2	Pd(*t*-$Bu_3P)_2$ (5 mol%)	91%	N.D.	85%
3	Davephos (7 mol%)	63%	N.D.	57%
4	HCl (1 equiv, 4 M in 1,4-dioxane)	> 95%	10%	68%
5	Standard Conditions	> 95%	5%	73%

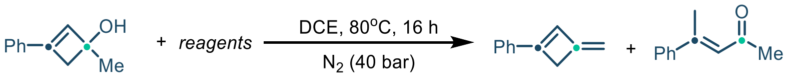

1a **9** **10**

A vial (4 mL) equipped with a magnetic stirring bar was charged with cyclobutenol **1a** (1.0 equiv, 0.1 mmol) and the *corresponding reagent*. The vial was sealed with a PTFE/white rubber septum (Wheaton 13 mm septa) and a phenolic cap and connected to the atmosphere through a needle. The vial was evacuated and backfilled with argon three times. DCE (3.5 mL) was then added via syringe under a nitrogen atmosphere. Subsequently, the vial (or several vials) was placed in an alloy plate, which was transferred into a 300 mL autoclave (Parr Instruments, Series 4560). After the autoclave was flushed three times with N_2, it was pressurized to 40 bar with N_2 at ambient temperature. The reaction mixture was then stirred at 90 °C for 16 h. After completion of the reaction, the autoclave was cooled to room temperature using an ice-water bath, and the pressure was carefully released. The yields of **9** and **10** were determined by GC analysis using n-hexadecane as an internal standard, and the identities of the products were confirmed by GC-MS and NMR analysis. See following table.

Entry	*reagents*	Conv.	Yield of **7** (%)	Yield of **8** (ratio)
1	$PhNH_2$ HCl (1 equiv)	50%	8%	35%
2	[Pd(π-cinnamyl)Cl]$_2$ (5 mol%)	54%	13%	16%
3	Xantphos (15 mol%)	48%	30%	8%
4	HCl (1 equiv, 4 M in 1,4-dioxane)	73%	46%	13%
5	Standard Conditions	85%	27%	49%

6.4 Hammett plot analysis

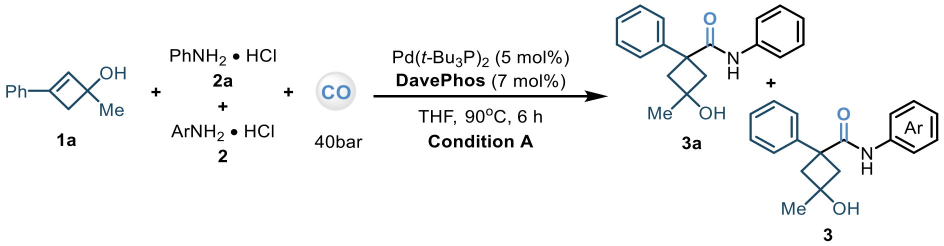

A 4 mL screw-cap vial was charged with $Pd(t\text{-}Bu_3P)_2$(5 mol%), DavePhos (7 mol%), cyclobutanol (**1a**, 0.1 mmol, 1.0 equiv), $PhNH_2$·HCl (**2a**, 0.1 mmol, 1.0 equiv), $ArNH_2$·HCl (**2**, 0.1 mmol, 1.0 equiv), and an oven-dried magnetic stirring bar. The vial was sealed with a PTFE septum and cap and connected to the atmosphere via a needle. After THF (2.0 mL) was added via syringe under an argon atmosphere, the vial was placed in an alloy plate and transferred into a 300 mL Parr 4560 autoclave under argon. The autoclave was purged with CO three times and then pressurized to 40 atm CO at room temperature. The reaction mixture was heated to 90 °C for 6 h with stirring. After cooling to room temperature, the crude reaction mixture was analyzed directly by GC and GC-MS analysis to determine the product distribution (see table below).

$ArNH_2$	σ	**3a**:**3** (ration of yield)	K_X/K_H	$Log(K_X/K_H)$
p-OMe	-0.268	72.3%/27.7%	0.383	-0.417
p-Me	-0.17	65.9%/34.1%	0.517	-0.287
p-F	0.062	51.5%/48.5%	0.942	-0.026
p-Cl	0.227	40.1%/59.9%	1.493	0.174
p-CF_3	0.54	30.2%/69.8%	2.311	0.364

Figure S1 Hammett plot. The ratio of the yield of **3** and **3a** were determined by GC and GC-MS.

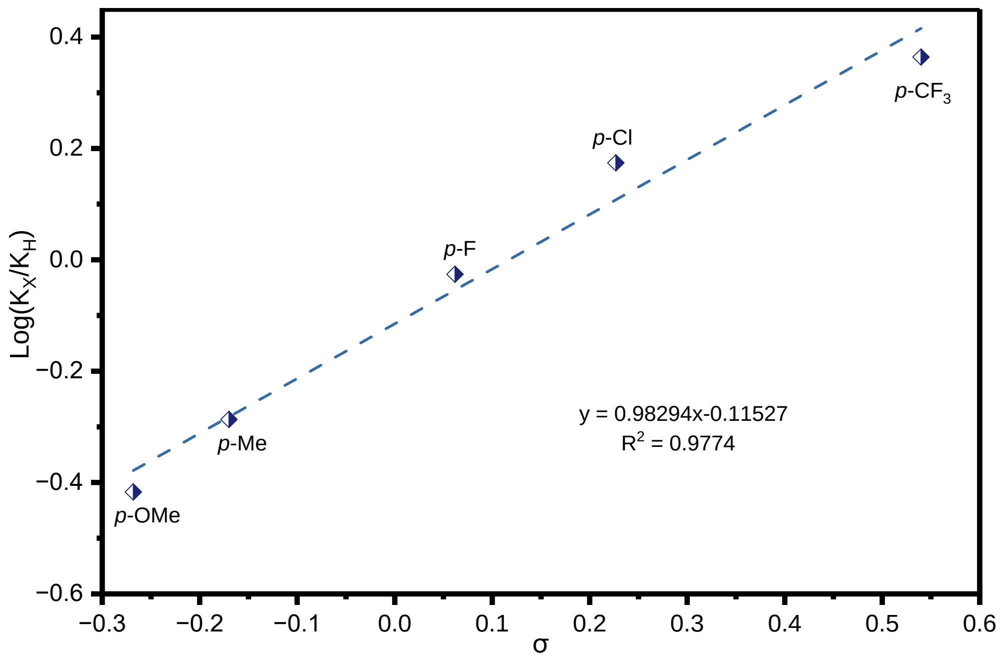

Figure S2. Hammett plot of $Log(K_X/K_H)$ versus σp under condition A.

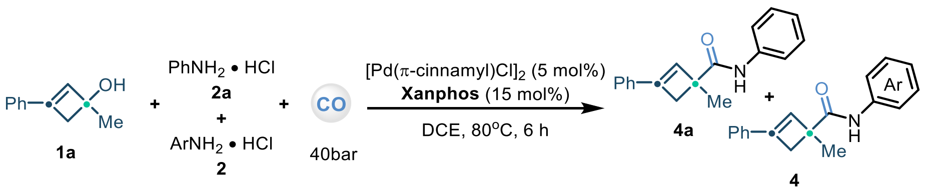

A vial (8 mL) was charged with [Pd(π-cinnamyl)Cl]2 (5 mol%), XantPhos(15 mol%), ,cyclobutenol (**1**, 1.0 equiv., 0.2 mmol) $PhNH_2$·HCl (**2a**, 0.1 mmol, 1.0 equiv), $ArNH_2$·HCl (**2**, 0.1 mmol, 1.0 equiv) and a stirring bar. The vial was sealed with a PTFE septum and cap and connected to the atmosphere via a needle. After DCE (3.5 mL) was added via syringe under an argon atmosphere, the vial was placed in an alloy plate and transferred into a 300 mL Parr 4560 autoclave under argon. The autoclave was purged with CO three times and then pressurized to 40 atm CO at room temperature. The reaction mixture was heated to 80 °C for 6 h with stirring. After cooling to room temperature, the crude reaction mixture was analyzed directly by GC and GC-MS analysis to determine the product distribution (see table below).

$ArNH_2$	σ	4a:4 (ration of yield)	K_X/K_H	$Log(K_X/K_H)$
p-OMe	-0.268	71.9%/28.1%	0.391	-0.408
p-Me	-0.17	62.7%/37.3%	0.594	-0.226
p-F	0.062	47.9%/52.1%	1.088	0.0366
p-Cl	0.227	42.8%/57.2%	1.336	0.126
p-CF_3	0.54	30.0%/70.0%	2.333	0.368

Figure S3 Hammett plot. The ratio of the yield of **4** and **4a** were determined by GC and GC-MS.

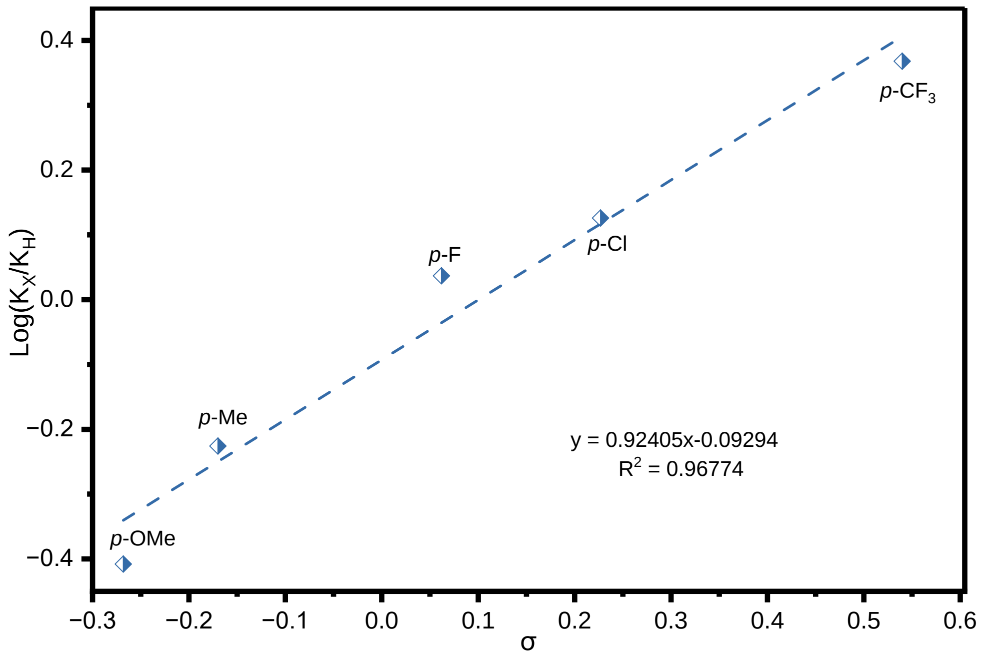

Figure S2. Hammett plot of $Log(K_X/K_H)$ versus σp under condition B.

6.5 Stability of **3a** under acidic conditions

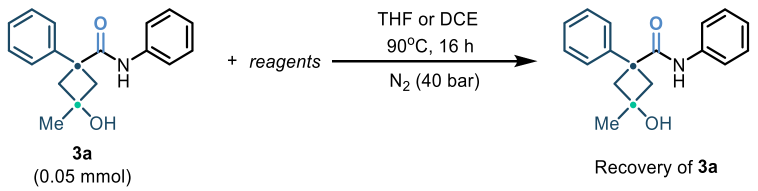

A vial (4 mL) equipped with a magnetic stirring bar was charged with **3a** (1.0 equiv, 0.05 mmol) and the *corresponding acid*. The vial was sealed with a PTFE/white rubber septum (Wheaton 13 mm septa) and a phenolic cap and connected to the atmosphere through a needle. The vial was evacuated and backfilled with argon three times. THF or DCE (1.0 mL) was then added via syringe under a nitrogen atmosphere. Subsequently, the vial (or several vials) was placed in an alloy plate, which was transferred into a 300 mL autoclave (Parr Instruments, Series 4560). After the autoclave was flushed three times with N_2, it was pressurized to 40 bar with N_2 at ambient temperature. The reaction mixture was then stirred at 90 °C for 16 h. After completion of the reaction, the autoclave was cooled to room temperature using an ice-water bath, and the pressure was carefully released. The recovery **3a** were determined by GC analysis using n-hexadecane as an internal standard.

Entry	Solvent	*reagents*	Recovery of **3a**
1	THF	PTSA H_2O (1 equiv)	> 98%
2	THF	$PhNH_2$ HCl (1 equiv)	90%
3	THF	TFA (1 equiv)	92%
4	THF	HCl (1 equiv, 4 M in 1,4-dioxane)	93%
5	THF	Condition A	92%
6	DCE	HCl (1 equiv, 4 M in 1,4-dioxane)	93%
7	DCE	HCl (2 equiv, 4 M in 1,4-dioxane)	94%
8	DCE	HCl (3 equiv, 4 M in 1,4-dioxane)	94%

7. Spectroscopic Data of Products

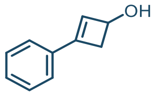

3-phenylcyclobut-2-en-1-ol (1q)

532.9 mg, 73% yield, pale yellow solid. Eluent: pentane/ethyl acetate = 10:1, R_f = 0.2.

^{1}H NMR (400 MHz, CDCl$_3$) δ 7.42 – 7.28 (m, 5H), 6.38 (s, 1H), 4.79 (d, *J* = 3.9 Hz, 1H), 3.19 (dd, *J* = 13.0, 4.0 Hz, 1H), 2.60 (d, *J* = 12.9 Hz, 1H).

^{13}C NMR (101 MHz, CDCl$_3$) δ 147.3, 133.9, 129.4, 128.8, 128.5, 125.5, 67.5, 40.6.

HRMS (**ESI**) (m/z): $[M + Na]^+$ Calcd for $C_{10}H_{10}ONa$ 169.0624; Found: 169.0627.

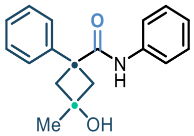

3-hydroxy-3-methyl-N,1-diphenylcyclobutane-1-carboxamide (3a)

27.6 mg, 98% yield, white solid. Eluent: pentane/ethyl acetate = 4:1, R_f = 0.2.

^{1}H NMR (300 MHz, CDCl$_3$) δ 7.52 – 7.45 (m, 2H), 7.42 – 7.35 (m, 1H), 7.32 (dd, *J* = 7.0, 1.8 Hz, 2H), 7.29 – 7.22 (m, 4H), 7.14 – 7.05 (m, 1H), 6.84 (br, 1H), 3.10 – 2.94 (m, 2H), 2.84 – 2.59 (m, 2H), 1.33 (s, 3H).

^{13}C NMR (75 MHz, CDCl$_3$) δ 177.0, 143.3, 137.2, 129.4, 129.0, 127.9, 127.3, 125.0, 120.3, 69.3, 47.9, 46.8, 28.7.

HRMS (ESI-TOF): m/z calcd. for $[M + Na]^+$ $C_{18}H_{19}NO_2Na^+$ 304.1308; Found: 304.1308.

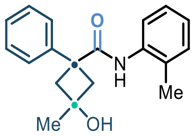

3-hydroxy-3-methyl-1-phenyl-N-(o-tolyl)cyclobutane-1-carboxamide (3b)

24.8 mg, 84% yield, white solid. Eluent: pentane/ethyl acetate = 4:1, R_f = 0.2.

^{1}H NMR (300 MHz, CDCl$_3$) δ 7.88 (d, *J* = 8.0 Hz, 1H), 7.55 – 7.43 (m, 2H), 7.43 – 7.31 (m, 3H), 7.23 – 7.15 (m, 1H), 7.10 – 6.97 (m, 2H), 6.75 (br, 1H), 3.27 – 2.90 (m, 2H), 2.83 – 2.60 (m, 2H), 1.73 (s, 3H), 1.34 (s, 3H).

^{13}C NMR (75 MHz, CDCl$_3$) δ 177.2, 143.6, 135.4, 130.5, 129.4, 128.5, 127.9, 127.4, 127.0, 125.5, 122.1, 69.4, 47.9, 47.0, 28.7, 17.0.

HRMS (ESI-TOF): m/z calcd. for $[M + Na]^+$ $C_{19}H_{21}NO_2Na^+$ 318.1464; Found: 318.1473.

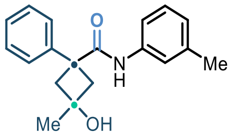

3-hydroxy-3-methyl-1-phenyl-N-(m-tolyl)cyclobutane-1-carboxamide (3c)

24.4 mg, 83% yield, white solid. Eluent: pentane/ethyl acetate = 4:1, R_f = 0.2.

^{1}H NMR (300 MHz, CDCl$_3$) δ 7.51 – 7.43 (m, 2H), 7.42 – 7.35 (m, 1H), 7.33 – 7.28 (m, 2H), 7.24 – 7.09 (m, 2H), 7.08 – 7.00 (m, 1H), 6.96 – 6.85 (m, 1H), 6.76 (br, 1H), 3.06 – 2.93 (m, 2H), 2.79 – 2.64 (m, 2H), 2.29 (d, *J* = 0.7 Hz, 3H), 1.33 (s, 3H).

^{13}C NMR (75 MHz, CDCl$_3$) δ 177.0, 143.4, 139.1, 137.1, 129.5, 128.9, 127.9, 127.4, 125.9, 121.0, 117.5, 69.4, 48.0, 46.9, 21.5.

HRMS (ESI-TOF): m/z calcd. for $[M + Na]^+$ $C_{19}H_{21}NO_2Na^+$ 318.1464; Found: 318.1469.

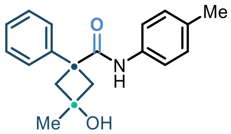

3-hydroxy-3-methyl-1-phenyl-N-(p-tolyl)cyclobutane-1-carboxamide (3d)

28.3 mg, 95% yield, white solid. Eluent: pentane/ethyl acetate = 3:1, R_f = 0.2.

^{1}H NMR (300 MHz, CDCl3) δ 7.54 – 7.43 (m, 2H), 7.42 – 7.34 (m, 1H), 7.32 – 7.27 (m, 2H), 7.21 – 7.12 (m, 2H), 7.08 – 6.98 (m, 2H), 6.75 (br, 1H), 3.14 – 2.94 (m, 2H), 2.86 – 2.59 (m, 2H), 2.28 (s, 3H), 1.32 (s, 3H).

^{13}C NMR (75 MHz, CDCl$_3$) δ 177.0, 143.5, 134.8, 134.6, 129.5, 129.4, 127.8, 127.4, 120.5, 69.4, 48.0, 46.8, 28.7, 21.0.
HRMS (ESI-TOF): m/z calcd. for $[M + Na]^+$ $C_{19}H_{21}NO_2Na^+$ 318.1464; Found: 318.1473.

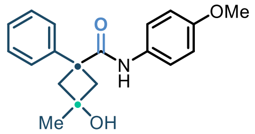

3-hydroxy-N-(4-methoxyphenyl)-3-methyl-1-phenylcyclobutane-1-carboxamide (3e)
30.6 mg, 98% yield, white solid. Eluent: pentane/ethyl acetate = 2:1, R_f = 0.3.
^{1}H NMR (300 MHz, CDCl$_3$) δ 7.49 – 7.42 (m, 2H), 7.41 – 7.36 (m, 1H), 7.33 – 7.28 (m, 2H), 7.21 – 7.14 (m, 2H), 6.83 – 6.75 (m, 2H), 6.71 (br, 1H), 4.25 (br, 1H), 3.75 (s, 3H), 3.03 – 2.90 (m, 2H), 2.78 – 2.63 (m, 2H), 1.32 (s, 3H).
^{13}C NMR (75 MHz, CDCl$_3$) δ 177.0, 156.9, 143.4, 130.1, 129.3, 127.7, 127.3, 122.3, 114.1, 69.3, 55.5, 47.9, 46.6, 28.6.
HRMS (ESI-TOF): m/z calcd. for $[M + Na]^+$ $C_{19}H_{21}NO_3Na^+$ 334.1414; Found: 334.1417.

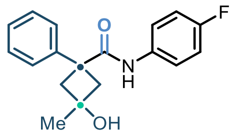

N-(4-fluorophenyl)-3-hydroxy-3-methyl-1-phenylcyclobutane-1-carboxamide (3f)
26.0 mg, 87% yield, white solid. Eluent: pentane/ethyl acetate = 3:1, R_f = 0.2.
^{1}H NMR (300 MHz, CDCl$_3$) δ 7.46 – 7.39 (m, 4H), 7.37 – 7.30 (m, 3H), 7.28 – 7.22 (m, 2H), 6.92 (br, 1H), 3.07 – 2.83 (m, 2H), 2.77 – 2.55 (m, 2H), 1.25 (s, 3H).
^{13}C NMR (75 MHz, CDCl$_3$) δ 177.2, 142.9, 140.4, 129.6, 128.1, 127.4, 126.9, 126.3 (q, J = 3.8 Hz), 122.3, 119.8, 69.4, 48.0, 46.9, 28.7.
^{19}F NMR (282 MHz, CDCl$_3$) δ -62.2 (s).
HRMS (ESI-TOF): m/z calcd. for $[M + Na]^+$ $C_{18}H_{18}FNO_2Na^+$ 322.1214; Found: 322.1216.

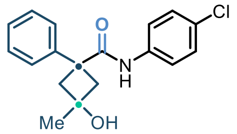

N-(4-chlorophenyl)-3-hydroxy-3-methyl-1-phenylcyclobutane-1-carboxamide (3g)
22.4 mg, 71% yield, white solid. Eluent: pentane/ethyl acetate = 2:1, R_f = 0.3.
^{1}H NMR (400 MHz, CDCl$_3$) δ 7.43 – 7.37 (m, 2H), 7.34 – 7.29 (m, 1H), 7.26 – 7.21 (m, 2H), 7.20 – 7.12 (m, 4H), 6.75 (br, 1H), 3.05 – 2.78 (m, 2H), 2.79 – 2.46 (m, 2H), 1.25 (s, 3H).
^{13}C NMR (101 MHz, CDCl$_3$) δ 177.0, 143.1, 135.8, 130.1, 129.5, 129.1, 128.0, 127.4, 121.5, 69.4, 48.0, 46.8, 28.7.
HRMS (ESI-TOF): m/z calcd. for $[M + Na]^+$ $C_{18}H_{18}ClNO_2Na^+$ 338.0918; Found: 338.0919.

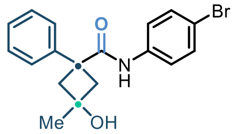

N-(4-bromophenyl)-3-hydroxy-3-methyl-1-phenylcyclobutane-1-carboxamide (3h)
26.5 mg, 74% yield, white solid. Eluent: pentane/ethyl acetate = 2:1, R_f = 0.3.
^{1}H NMR (400 MHz, CDCl$_3$) δ 7.51 – 7.44 (m, 2H), 7.42 – 7.34 (m, 3H), 7.33 – 7.28 (m, 2H), 7.21 – 7.16 (m, 2H), 6.82 (br, 1H), 3.05 – 2.91 (m, 2H), 2.82 – 2.66 (m, 2H), 1.32 (s, 3H).
^{13}C NMR (101 MHz, CDCl$_3$) δ 177.0, 143.1, 136.4, 132.0, 129.5, 128.0, 127.4, 121.8, 117.7, 69.4, 48.0, 46.8, 28.7.
HRMS (ESI-TOF): m/z calcd. for $[M + Na]^+$ $C_{18}H_{18}BrNO_2Na^+$ 382.0413; Found: 382.0415.

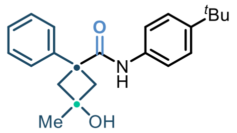

N-(4-(tert-butyl)phenyl)-3-hydroxy-3-methyl-1-phenylcyclobutane-1-carboxamide (3i)

31.7 mg, 94% yield, white solid. Eluent: pentane/ethyl acetate = 4:1, R_f = 0.2.

^{1}H NMR (300 MHz, $CDCl_3$) δ 7.50 – 7.43 (m, 2H), 7.40 – 7.34 (m, 1H), 7.33 – 7.26 (m, 4H), 7.22 – 7.16 (m, 2H), 6.75 (br, 1H), 3.10 – 2.90 (m, 2H), 2.83 – 2.61 (m, 2H), 1.33 (s, 3H), 1.26 (s, 9H).

^{13}C NMR (75 MHz, $CDCl_3$) δ 177.1, 148.3, 143.5, 134.5, 129.4, 127.9, 127.4, 125.9, 120.4, 69.4, 48.0, 46.8, 34.5, 31.4, 28.7.

HRMS (ESI-TOF): m/z calcd. for $[M + Na]^+$ $C_{22}H_{27}NO_2Na^+$ 360.1934; Found: 360.1931.

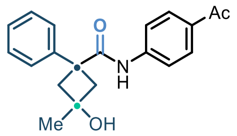

N-(4-acetylphenyl)-3-hydroxy-3-methyl-1-phenylcyclobutane-1-carboxamide (3j)

30.4 mg, 94% yield, white solid. Eluent: pentane/ethyl acetate = 2:1, R_f = 0.3.

^{1}H NMR (300 MHz, $CDCl_3$) δ 7.90 – 7.82 (m, 2H), 7.53 – 7.44 (m, 2H), 7.43 – 7.37 (m, 3H), 7.34 – 7.29 (m, 2H), 7.06 (br, 1H), 3.10 – 2.95 (m, 2H), 2.85 – 2.68 (m, 2H), 2.54 (s, 3H), 1.32 (s, 3H).

^{13}C NMR (75 MHz, $CDCl_3$) δ 196.9, 177.1, 142.8, 141.6, 133.4, 129.7, 129.6, 128.1, 127.3, 119.3, 69.4, 47.9, 46.9, 28.7, 26.5 (d, *J* = 1.8 Hz).

HRMS (ESI-TOF): m/z calcd. for $[M + Na]^+$ $C_{20}H_{21}NO_3Na^+$ 346.1413; Found: 346.1418.

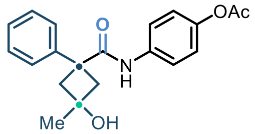

4-(3-hydroxy-3-methyl-1-phenylcyclobutane-1-carboxamido)phenyl acetate (3k)

30.8 mg, 91% yield, white solid. Eluent: pentane/ethyl acetate = 4:3, R_f = 0.2.

^{1}H NMR (400 MHz, $CDCl_3$) δ 7.93 (d, J = 8.7 Hz, 2H), 7.52 – 7.45 (m, 2H), 7.42 – 7.35 (m, 3H), 7.34 – 7.29 (m, 2H), 7.03 (br, 1H), 3.87 (s, 3H), 3.04 – 2.96 (m, 2H), 2.76 – 2.70 (m, 2H), 1.31 (s, 3H).

^{13}C NMR (101 MHz, $CDCl_3$) δ 177.0, 166.5, 142.8, 141.4, 130.8, 129.6, 128.1, 127.4, 126.2, 119.2, 69.3, 52.2, 47.9, 46.9, 28.7.

HRMS (ESI-TOF): m/z calcd. for $[M + Na]^+$ $C_{20}H_{21}NO_4Na^+$ 362.1363; Found: 363.1368.

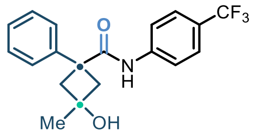

3-hydroxy-3-methyl-1-phenyl-N-(4-(trifluoromethyl)phenyl)cyclobutane-1-carboxamide (3l)

25.7 mg, 74% yield, white solid. Eluent: pentane/ethyl acetate = 3:1, R_f = 0.2.

^{1}H NMR (300 MHz, $CDCl_3$) δ 7.53 – 7.46 (m, 2H), 7.44 – 7.37 (m, 1H), 7.36 – 7.30 (m, 2H), 7.30 – 7.23 (m, 2H), 7.02 – 6.93 (m, 2H), 6.80 (br, 1H), 3.16 – 2.87 (m, 2H), 2.84 – 2.54 (m, 2H), 1.35 (s, 3H).

^{13}C NMR (75 MHz, $CDCl_3$) δ 177.1, 159.9 (d, *J* = 244.7 Hz), 143.3, 133.2 (d, *J* = 2.8 Hz), 129.5, 128.0, 127.4, 122.3 (d, *J* = 8.0 Hz), 115.8 (d, *J* = 22.7 Hz), 69.4, 48.0, 46.8, 28.7.

^{19}F NMR (282 MHz, $CDCl_3$) δ -116.92 – -117.08 (m).

HRMS (ESI-TOF): m/z calcd. for $[M + Na]^+$ $C_{19}H_{18}F_3NO2Na^+$ 372.1182; Found: 372.1181.

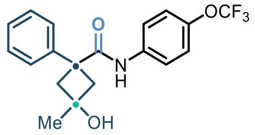

3-hydroxy-3-methyl-1-phenyl-N-(4-(trifluoromethoxy)phenyl)cyclobutane-1-carboxamide (3m)

27.0 mg, 74% yield, white solid. Eluent: pentane/ethyl acetate = 2:1, R_f = 0.3.

^{1}H NMR (300 MHz, $CDCl_3$) δ 7.53 – 7.44 (m, 2H), 7.43 – 7.36 (m, 1H), 7.35 – 7.29 (m, 4H), 7.16 – 7.06 (m, 2H), 6.86 (br, 1H), 5.12 (s, 1H), 3.15 – 2.86 (m, 2H), 2.82 – 2.64 (m, 2H), 1.32 (s, 3H).

^{13}C NMR (75 MHz, $CDCl_3$) δ 177.2, 145.9, 143.1, 135.9, 129.6, 128.1, 127.4, 121.8, 121.5, 69.4, 48.0, 46.8, 28.7.

^{19}F NMR (282 MHz, $CDCl_3$) δ -58.19 (s).

HRMS (ESI-TOF): m/z calcd. for $[M + Na]^+$ $C_{19}H_{18}F_3NO3Na^+$ 388.1131; Found: 388.1137.

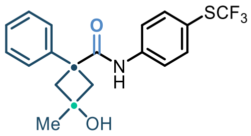

3-hydroxy-3-methyl-1-phenyl-N-(4-((trifluoromethyl)thio)phenyl)cyclobutane-1-carboxamide (3n)
29.7 mg, 78% yield, white solid. Eluent: pentane/ethyl acetate = 4:1, R_f = 0.3.
^{1}H NMR (300 MHz, CDCl$_3$) δ 7.56 – 7.45 (m, 4H), 7.43 – 7.35 (m, 3H), 7.34 – 7.29 (m, 2H), 6.97 (br, 1H), 3.04 – 2.91 (m, 2H), 2.79 – 2.67 (m, 2H), 1.32 (s, 3H).
^{13}C NMR (75 MHz, CDCl$_3$) δ 177.1, 142.8, 139.9, 137.5, 129.6, 128.1, 127.5, 127.3, 120.6, 119.6 (d, *J* = 2.3 Hz), 69.4, 47.9, 46.9, 28.7.
^{19}F NMR (282 MHz, CDCl$_3$) δ -43.28 (s).
HRMS (ESI-TOF): m/z calcd. for $[M + Na]^+$ $C_{19}H_{18}F_3NO_2SNa^+$ 404.0902; Found: 404.0906.

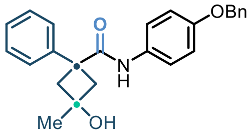

N-(4-(benzyloxy)phenyl)-3-hydroxy-3-methyl-1-phenylcyclobutane-1-carboxamide (3o)
33.5 mg, 87% yield, white solid. Eluent: pentane/ethyl acetate = 3:1, R_f = 0.2.
^{1}H NMR (300 MHz, CDCl$_3$) δ 7.50 – 7.43 (m, 2H), 7.41 – 7.36 (m, 4H), 7.36 – 7.28 (m, 4H), 7.22 – 7.15 (m, 2H), 6.96 – 6.83 (m, 2H), 6.70 (br, 1H), 5.02 (s, 2H), 3.03 – 2.91 (m, 2H), 2.83 – 2.57 (m, 2H), 1.33 (s, 3H).
^{13}C NMR (75 MHz, CDCl$_3$) δ 177.1, 156.1, 143.5, 136.9, 130.5, 129.4, 128.7, 128.1, 127.8, 127.5, 127.4, 122.3, 115.3, 70.4, 69.4, 48.0, 46.7, 28.7.
HRMS (ESI-TOF): m/z calcd. for $[M + Na]^+$ $C_{25}H_{25}NO_3Na^+$ 410.1726; Found: 410.1722.

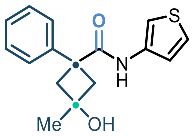

3-hydroxy-3-methyl-1-phenyl-N-(thiophen-3-yl)cyclobutane-1-carboxamide (3p)
18.0 mg, 63% yield, white solid. Eluent: pentane/ethyl acetate = 3:1, R_f = 0.2.
^{1}H NMR (300 MHz, CDCl$_3$) δ 7.54 – 7.43 (m, 3H), 7.41 – 7.35 (m, 1H), 7.33 – 7.27 (m, 2H), 7.15 (dd, *J* = 5.2, 3.2 Hz, 1H), 7.01 (br, 1H), 6.72 (dd, *J* = 5.2, 1.4 Hz, 1H), 3.14 – 2.95 (m, 2H), 2.84 – 2.40 (m, 2H), 1.32 (s, 3H).
^{13}C NMR (75 MHz, CDCl$_3$) δ 176.3, 143.3, 135.1, 129.5, 127.9, 127.4, 124.8, 121.3, 111.2, 69.4, 48.1, 46.5, 28.7.
HRMS (ESI-TOF): m/z calcd. for $[M + Na]^+$ $C_{16}H_{17}NO_2SNa^+$ 310.0872; Found: 310.0877.

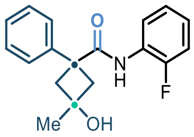

N-(2-fluorophenyl)-3-hydroxy-3-methyl-1-phenylcyclobutane-1-carboxamide (3q)
28.3 mg, 95% yield, white solid. Eluent: pentane/ethyl acetate = 3:1, R_f = 0.2.
^{1}H NMR (300 MHz, CDCl$_3$) δ 8.25 (td, *J* = 8.0, 1.8 Hz, 1H), 7.53 – 7.44 (m, 2H), 7.42 – 7.38 (m, 1H), 7.35 – 7.30 (m, 2H), 7.15 (br, 1H), 7.13 – 7.07 (m, 1H), 7.04 – 6.92 (m, 2H), 3.11 – 2.82 (m, 2H), 2.90 – 2.57 (m, 2H), 1.33 (s, 3H).
^{13}C NMR (75 MHz, CDCl$_3$) δ 177.1, 152.8 (d, *J* = 244.2 Hz), 142.9, 129.5, 128.0, 127.3, 126.0 (d, *J* = 10.0 Hz), 125.1 (d, *J* = 7.7 Hz), 124.6 (d, *J* = 3.8 Hz), 121.8, 114.9 (d, *J* = 19.0 Hz), 69.4, 48.0, 47.0, 28.7.
^{19}F NMR (282 MHz, CDCl$_3$) δ -131.29 – -131.60 (m).
HRMS (ESI-TOF): m/z calcd. for $[M + Na]^+$ $C_{18}H_{18}FNO_2Na^+$ 322.1214; Found: 322.1217.

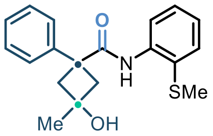

3-hydroxy-3-methyl-N-(2-(methylthio)phenyl)-1-phenylcyclobutane-1-carboxamide (3r)
24.6 mg, 75% yield, white solid. Eluent: pentane/ethyl acetate = 5:1, R_f = 0.2.
^{1}H NMR (300 MHz, CDCl$_3$) δ 8.28 (dd, *J* = 8.2, 1.4 Hz, 1H), 8.11 (br, 1H), 7.45 – 7.36 (m, 2H), 7.34 – 7.25 (m, 4H), 7.24 – 7.16 (m, 1H), 6.94 (td, *J* = 7.6, 1.4 Hz, 1H), 5.18 (br, 1H), 3.06 – 2.89 (m, 2H), 2.79 – 2.51 (m, 2H), 1.86 (s, 3H), 1.26 (s, 3H).
^{13}C NMR (75 MHz, CDCl$_3$) δ 177.2, 143.3, 138.4, 133.6, 129.4, 129.2, 127.8, 127.5, 125.9, 124.9, 120.4, 69.4, 48.0, 47.2, 28.7, 18.9.
HRMS (ESI-TOF): m/z calcd. for $[M + Na]^+$ $C_{19}H_{21}NO_2SNa^+$ 350.1185; Found: 350.1188.

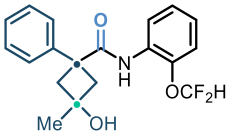

N-(2-(difluoromethoxy)phenyl)-3-hydroxy-3-methyl-1-phenylcyclobutane-1-carboxamide (3s)
29.4 mg, 85% yield, white solid. Eluent: pentane/ethyl acetate = 5:1, R_f = 0.2.
^{1}H NMR (300 MHz, CDCl$_3$) δ 8.37 (dd, *J* = 8.2, 1.5 Hz, 1H), 7.52 – 7.44 (m, 2H), 7.43 – 7.35 (m, 2H), 7.34 – 7.28 (m, 2H), 7.23 – 7.15 (m, 1H), 7.07 – 7.01 (m, 1H), 7.01 – 6.97 (m, 1H), 6.13 (t, *J* = 73.3 Hz, 1H), 5.22 (br, 1H), 3.06 – 2.95 (m, 2H), 2.84 – 2.64 (m, 2H), 1.33 (s, 3H).
^{13}C NMR (75 MHz, CDCl$_3$) δ 177.1, 142.9, 140.3 – 140.2 (t, *J* = 3.0 Hz), 129.7 (d, *J* = 33.7 Hz), 127.9, 127.4, 126.4, 124.7, 121.3, 119.1 (d, *J* = 21.8 Hz), 115.8, 112.3, 69.4, 47.9, 47.1, 28.7.
^{19}F NMR (282 MHz, CDCl$_3$) δ -80.50 (s, 1F), -80.76 (s, 1F).
HRMS (ESI-TOF): m/z calcd. for $[M + Na]^+$ $C_{19}H_{19}F_2NO_3Na^+$ 370.1225; Found: 370.1221.

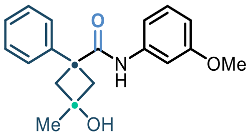

3-hydroxy-N-(3-methoxyphenyl)-3-methyl-1-phenylcyclobutane-1-carboxamide (3t)
30.1 mg, 97% yield, white solid. Eluent: pentane/ethyl acetate = 3:1, R_f = 0.2.
^{1}H NMR (300 MHz, CDCl$_3$) δ 7.51 – 7.43 (m, 2H), 7.42 – 7.34 (m, 1H), 7.33 – 7.28 (m, 2H), 7.21 – 6.98 (m, 2H), 6.80 (br, 1H), 6.73 – 6.53 (m, 2H), 3.77 (s, 3H), 3.12 – 2.91 (m, 2H), 2.80 – 2.65 (m, 2H), 1.32 (s, 3H).
^{13}C NMR (75 MHz, CDCl$_3$) δ 177.0, 160.2, 143.3, 138.5, 129.7, 129.5, 127.9, 127.4, 112.4, 110.7, 106.1, 69.4, 55.5, 48.0, 46.9, 28.7.
HRMS (ESI-TOF): m/z calcd. for $[M + Na]^+$ $C_{19}H_{21}NO_3Na^+$ 334.1413; Found: 334.1414.

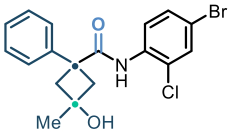

N-(4-bromo-2-chlorophenyl)-3-hydroxy-3-methyl-1-phenylcyclobutane-1-carboxamide (3u)
24.6 mg, 63% yield, white solid. Eluent: pentane/ethyl acetate = 3:1, R_f = 0.2.
^{1}H NMR (300 MHz, CDCl$_3$) δ 7.53 (d, *J* = 2.5 Hz, 1H), 7.51 – 7.44 (m, 3H), 7.43 – 7.39 (m, 1H), 7.32 – 7.28 (m, 2H), 7.05 (dd, *J* = 8.7, 2.5 Hz, 1H), 6.85 (br, 1H), 3.02 – 2.94 (m, 2H), 2.76 – 2.68 (m, 2H), 1.32 (s, 3H).
^{13}C NMR (75 MHz, CDCl$_3$) δ 177.0, 142.8, 137.5, 134.9, 133.8, 129.6, 128.2, 127.3, 121.7, 119.6, 117.5, 69.4, 48.0, 46.8, 28.7.
HRMS (ESI-TOF): m/z calcd. for $[M + Na]^+$ $C_{18}H_{17}BrClNO_2Na^+$ 416.0023; Found: 416.0017.

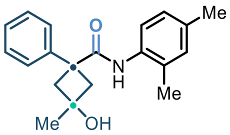

N-(2,4-dimethylphenyl)-3-hydroxy-3-methyl-1-phenylcyclobutane-1-carboxamide (3v)
28.9 mg, 94% yield, white solid. Eluent: pentane/ethyl acetate = 5:1, R_f = 0.2.

^{1}H NMR (300 MHz, CDCl$_3$) δ 7.66 (d, *J* = 8.2 Hz, 1H), 7.51 – 7.43 (m, 2H), 7.41 – 7.30 (m, 3H), 7.03 – 6.97 (m, 1H), 6.91 – 6.85 (m, 1H), 6.66 (br, 1H), 5.69 (br, 1H), 3.04 – 2.95 (m, 2H), 2.78 – 2.68 (m, 2H), 2.24 (s, 3H), 1.72 (s, 3H), 1.34 (s, 3H).
^{13}C NMR (101 MHz, CDCl$_3$) δ 177.2, 143.8, 135.3, 132.7, 131.2, 129.4, 128.9, 128.5, 127.9, 127.4, 122.4, 69.4, 47.9, 46.9, 28.7, 21.0, 17.0.
HRMS (ESI-TOF): m/z calcd. for $[M + Na]^+$ $C_{20}H_{23}NO_2Na^+$ 332.1621; Found: 332.1624.

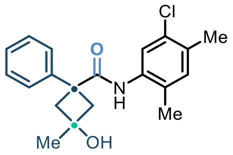

N-(5-chloro-2,4-dimethylphenyl)-3-hydroxy-3-methyl-1-phenylcyclobutane-1-carboxamide (3w)
33.7 mg, 98% yield, white solid. Eluent: pentane/ethyl acetate = 3:1, R_f = 0.2.
^{1}H NMR (300 MHz, CDCl$_3$) δ 8.36 (s, 1H), 7.49 – 7.43 (m, 2H), 7.41 – 7.36 (m, 1H), 7.34 (br, 1H), 7.31 – 7.27 (m, 2H), 6.35 (s, 1H), 3.82 (s, 3H), 3.56 (s, 3H), 2.96 (d, *J* = 13.7 Hz, 2H), 2.71 (d, *J* = 13.6 Hz, 2H), 1.32 (s, 3H).
^{13}C NMR (75 MHz, CDCl$_3$) δ 176.5, 151.7, 148.1, 143.3, 129.2, 127.6, 127.5, 121.3, 121.1, 113.9, 96.7, 69.3, 56.7, 56.2, 47.9, 47.0, 28.7.
HRMS (ESI-TOF): m/z calcd. for $[M + Na]^+$ $C_{20}H_{22}ClNO_2Na^+$ 366.1231; Found: 366.1232.

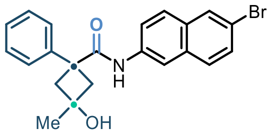

N-(6-bromonaphthalen-2-yl)-3-hydroxy-3-methyl-1-phenylcyclobutane-1-carboxamide (3x)
34.2 mg, 83% yield, white solid. Eluent: pentane/ethyl acetate = 3:1, R_f = 0.2.
^{1}H NMR (300 MHz, CDCl$_3$) δ 8.05 (d, *J* = 2.1 Hz, 1H), 7.89 (d, *J* = 2.0 Hz, 1H), 7.69 – 7.58 (m, 2H), 7.55 – 7.47 (m, 3H), 7.46 – 7.38 (m, 1H), 7.37 – 7.32 (m, 2H), 7.14 (dd, *J* = 8.8, 2.2 Hz, 1H), 6.99 (br, 1H), 3.32 – 2.94 (m, 2H), 2.84 – 2.64 (m, 2H), 1.34 (s, 3H).
^{13}C NMR (75 MHz, CDCl$_3$) δ 177.3, 143.2, 135.1, 132.2, 131.9, 130.1, 129.7, 129.6, 129.4, 128.0, 127.9, 127.4, 121.0, 119.3, 117.1, 69.4, 48.1, 46.9, 28.8.
HRMS (ESI-TOF): m/z calcd. for $[M + Na]^+$ $C_{22}H_{20}BrNO_2Na^+$ 432.0569; Found: 432.0570.

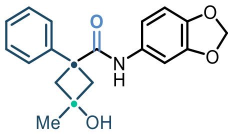

N-(benzo[d][1,3]dioxol-5-yl)-3-hydroxy-3-methyl-1-phenylcyclobutane-1-carboxamide (3y)
28.0 mg, 86% yield, white solid. Eluent: pentane/ethyl acetate = 3:1, R_f = 0.2.
^{1}H NMR (300 MHz, CDCl$_3$) δ 7.52 – 7.43 (m, 2H), 7.41 – 7.33 (m, 1H), 7.32 – 7.27 (m, 2H), 7.03 (d, *J* = 2.1 Hz, 1H), 6.70 (br, 1H), 6.66 (d, *J* = 8.3 Hz, 1H), 6.51 (dd, *J* = 8.3, 2.1 Hz, 1H), 5.91 (s, 2H), 3.11 – 2.88 (m, 2H), 2.78 – 2.61 (m, 2H), 1.32 (s, 3H).
^{13}C NMR (75 MHz, CDCl$_3$) δ 177.0, 147.9, 144.9, 143.4, 131.4, 129.4, 127.9, 127.4, 113.8, 108.0, 103.2, 101.5, 69.3, 48.0, 46.7, 28.7.
HRMS (ESI-TOF): m/z calcd. for $[M + Na]^+$ $C_{17}H_{19}NO_4Na^+$ 324.1206; Found: 324.1213.

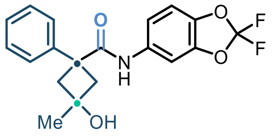

N-(2,2-difluorobenzo[d][1,3]dioxol-5-yl)-3-hydroxy-3-methyl-1-phenylcyclobutane-1-carboxamide (3z)
31.8 mg, 88% yield, white solid. Eluent: pentane/ethyl acetate = 2:1, R_f = 0.3.
^{1}H NMR (300 MHz, CDCl$_3$) δ 7.52 – 7.43 (m, 3H), 7.42 – 7.35 (m, 1H), 7.32 – 7.28 (m, 2H), 6.93 – 6.86 (m, 1H), 6.84 (br, 1H), 6.68 (dd, *J* = 8.6, 2.2 Hz, 1H), 5.09 (br, 1H), 3.10 – 2.88 (m, 2H), 2.80 – 2.62 (m, 2H), 1.32 (s, 3H).
^{13}C NMR (75 MHz, CDCl$_3$) δ 177.13, 143.96, 142.98, 140.68, 133.39, 131.86, 129.58, 128.08, 127.37, 115.25, 109.27, 103.59, 69.37, 47.99, 46.76, 28.72.
^{19}F NMR (282 MHz, CDCl$_3$) δ -50.02 (s).
HRMS (ESI-TOF): m/z calcd. for $[M + Na]^+$ $C_{17}H_{17}F_2NO_4Na^+$ 360.1018; Found: 306.1023.

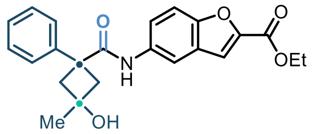

ethyl 5-(3-hydroxy-3-methyl-1-phenylcyclobutane-1-carboxamido)benzofuran-2-carboxylate (3ab)
32.4 mg, 82% yield, white solid. Eluent: pentane/ethyl acetate = 5:1, R_f = 0.2.
^{1}H NMR (300 MHz, CDCl$_3$) δ 7.90 (d, *J* = 2.2 Hz, 1H), 7.52 – 7.45 (m, 2H), 7.44 – 7.36 (m, 3H), 7.35 – 7.30 (m, 2H), 7.06 (dd, *J* = 9.0, 2.2 Hz, 1H), 6.91 (br, 1H), 5.31 (br, 1H), 4.43 (q, *J* = 7.1 Hz, 2H), 3.08 – 2.96 (m, 2H), 2.85 – 2.62 (m, 2H), 1.41 (t, *J* = 7.1 Hz, 3H), 1.33 (s, 3H).
^{13}C NMR (75 MHz, CDCl$_3$) δ 177.3, 159.5, 153.0, 146.8, 143.3, 133.3, 129.5, 128.0, 127.5, 127.4, 121.3, 114.5, 113.8, 112.6, 69.4, 61.8, 48.1, 46.8, 28.8, 14.4.
HRMS (ESI-TOF): m/z calcd. for $[M + Na]^+$ $C_{23}H_{23}NO_5Na^+$ 416.1468; Found: 416.1471.

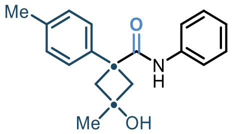

3-hydroxy-3-methyl-N-phenyl-1-(p-tolyl)cyclobutane-1-carboxamide (3ae)
22.0 mg, 75% yield, white solid. Eluent: pentane/ethyl acetate = 4:1, R_f = 0.2.
^{1}H NMR (300 MHz, CDCl$_3$) δ 7.33 – 7.23 (m, 6H), 7.22 – 7.16 (m, 2H), 7.12 – 7.05 (m, 1H), 6.86 (br, 1H), 3.02 – 2.93 (m, 2H), 2.74 – 2.65 (m, 2H), 2.41 (d, *J* = 0.7 Hz, 3H), 1.32 (s, 3H).
^{13}C NMR (75 MHz, CDCl$_3$) δ 177.3, 140.3, 137.7, 137.3, 130.1, 129.1, 127.3, 125.0, 120.4, 69.4, 48.0, 46.5, 28.7, 21.3.
HRMS (ESI-TOF): m/z calcd. for $[M + Na]^+$ $C_{19}H_{21}NO_2Na^+$ 318.1464; Found: 318.1470.

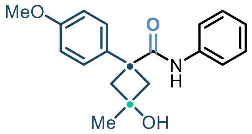

3-hydroxy-1-(4-methoxyphenyl)-3-methyl-N-phenylcyclobutane-1-carboxamide (3af)
24.0 mg, 77% yield, white solid. Eluent: pentane/ethyl acetate = 3:1, R_f = 0.2.
^{1}H NMR (300 MHz, CDCl$_3$) δ 7.33 – 7.25 (m, 4H), 7.25 – 7.19 (m, 2H), 7.12 – 7.05 (m, 1H), 7.02 – 6.96 (m, 2H), 6.86 (br, 1H), 5.38 (br, 1H), 3.86 (s, 3H), 3.07 – 2.89 (m, 2H), 2.74 – 2.60 (m, 2H), 1.32 (s, 3H).
^{13}C NMR (75 MHz, CDCl$_3$) δ 177.5, 159.1, 137.3, 135.2, 129.1, 128.6, 125.1, 120.4, 114.8, 69.3, 55.5, 48.1, 46.2, 28.8.
HRMS (ESI-TOF): m/z calcd. for $[M + Na]^+$ $C_{19}H_{21}NO_3Na^+$ 334.1413; Found: 334.1412.

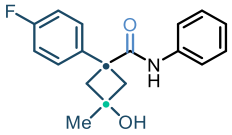

1-(4-fluorophenyl)-3-hydroxy-3-methyl-N-phenylcyclobutane-1-carboxamide (3ag)
26.7 mg, 89% yield, white solid. Eluent: pentane/ethyl acetate = 3:1, R_f = 0.2.
^{1}H NMR (300 MHz, CDCl$_3$) δ 7.32 – 7.28 (m, 4H), 7.28 – 7.24 (m, 2H), 7.20 – 7.13 (m, 2H), 7.12 – 7.06 (m, 1H), 6.80 (s, 1H), 5.25 (s, 1H), 3.08 – 2.88 (m, 2H), 2.75 – 2.62 (m, 2H), 1.32 (s, 3H).
^{13}C NMR (75 MHz, CDCl$_3$) δ 176.7, 162.2 (d, *J* = 248.0 Hz), 139.2 (d, *J* = 3.4 Hz), 137.1, 129.2 (d, *J* = 8.1 Hz), 129.1, 125.2, 120.4, 116.4 (d, *J* = 21.6 Hz), 69.2, 48.1, 46.3, 28.8.
^{19}F NMR (282 MHz, CDCl$_3$) δ -113.68 – -113.81 (m).
HRMS (ESI-TOF): m/z calcd. for $[M + Na]^+$ $C_{18}H_{18}FNO_2Na^+$ 322.1214; Found: 322.1219.

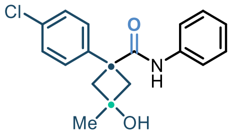

1-(4-chlorophenyl)-3-hydroxy-3-methyl-N-phenylcyclobutane-1-carboxamide (3ah)
29.1 mg, 92% yield, white solid. Eluent: pentane/ethyl acetate = 2:1, R_f = 0.2.

^{1}H NMR (300 MHz, CDCl$_3$) δ 7.51 – 7.43 (m, 2H), 7.38 – 7.33 (m, 1H), 7.32 – 7.30 (m, 2H), 7.30 – 7.25 (m, 3H), 7.17 – 7.07 (m, 1H), 6.82 (br, 1H), 3.15 – 2.91 (m, 2H), 2.77 – 2.62 (m, 2H), 1.33 (s, 3H).
^{13}C NMR (75 MHz, CDCl$_3$) δ 176.3, 141.8, 137.1, 133.9, 129.7, 129.1 (d, *J* = 2.9 Hz), 128.9, 125.2, 120.4, 69.2, 48.1, 46.4, 28.8.
HRMS (ESI-TOF): m/z calcd. for $[M + Na]^+$ $C_{18}H_{18}ClNO_2Na^+$ 338.0918; Found: 338.0922.

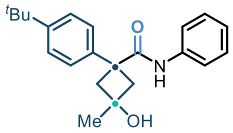

1-(4-(tert-butyl)phenyl)-3-hydroxy-3-methyl-N-phenylcyclobutane-1-carboxamide (3ai)
27.0 mg, 92% yield, white solid. Eluent: pentane/ethyl acetate = 3:1, R_f = 0.2.
^{1}H NMR (300 MHz, CDCl$_3$) δ 7.50 – 7.45 (m, 2H), 7.30 – 7.26 (m, 4H), 7.25 – 7.19 (m, 2H), 7.14 – 7.04 (m, 1H), 6.85 (br, 1H), 3.00 – 2.92 (m, 2H), 2.76 – 2.65 (m, 2H), 1.37 (s, 9H), 1.32 (s, 3H).
^{13}C NMR (75 MHz, CDCl$_3$) δ 177.4, 150.9, 140.2, 137.3, 129.1, 127.1, 126.3, 125.1, 120.5, 69.4, 48.0, 46.5, 34.8, 31.4, 28.7.
HRMS (ESI-TOF): m/z calcd. for $[M + Na]^+$ $C_{22}H_{27}NO_2Na^+$ 360.1934; Found: 360.1932.

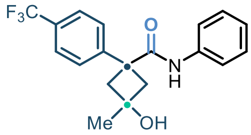

3-hydroxy-3-methyl-N-phenyl-1-(4-(trifluoromethyl)phenyl)cyclobutane-1-carboxamide (3aj)
20.4 mg, 58% yield, white solid. Eluent: pentane/ethyl acetate = 5:2, R_f = 0.2.
^{1}H NMR (300 MHz, CDCl$_3$) δ 7.74 (d, *J* = 8.0 Hz, 2H), 7.46 (d, *J* = 8.0 Hz, 2H), 7.32 – 7.27 (m, 4H), 7.16 – 7.05 (m, 1H), 6.78 (br, 1H), 3.11 – 2.95 (m, 2H), 2.81 – 2.61 (m, 2H), 1.33 (s, 3H).
^{13}C NMR (75 MHz, CDCl$_3$) δ 175.8, 147.4, 137.0, 129.2, 127.9, 126.8, 126.4 (q, *J* = 3.3 Hz), 125.4, 120.5, 119.9, 69.4, 48.1, 46.9, 28.8.
^{19}F NMR (282 MHz, CDCl$_3$) δ -62.60 (s).
HRMS (ESI-TOF): m/z calcd. for $[M + Na]^+$ $C_{19}H_{18}F_3NO_2Na^+$ 372.1182; Found: 372.1185.

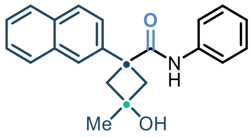

3-hydroxy-3-methyl-1-(naphthalen-2-yl)-N-phenylcyclobutane-1-carboxamide (3ak)
26.9 mg, 81% yield, white solid. Eluent: pentane/ethyl acetate = 3:1, R_f = 0.2.
^{1}H NMR (300 MHz, CDCl$_3$) δ 8.00 – 7.86 (m, 3H), 7.81 (d, *J* = 1.9 Hz, 1H), 7.64 – 7.53 (m, 2H), 7.38 (dd, *J* = 8.5, 1.9 Hz, 1H), 7.29 – 7.18 (m, 4H), 7.12 – 7.03 (m, 1H), 6.90 (br, 1H), 3.13 – 3.03 (m, 2H), 2.89 – 2.75 (m, 2H), 1.35 (s, 3H).
^{13}C NMR (75 MHz, CDCl$_3$) δ 176.9, 140.5, 137.2, 133.4, 132.6, 129.6, 129.0, 128.1, 128.0, 127.2, 126.9, 126.2, 125.2, 125.1, 120.4, 69.4, 48.0, 47.0, 28.8.
HRMS (ESI-TOF): m/z calcd. for $[M + Na]^+$ $C_{22}H_{21}NO_2Na^+$ 354.1464; Found: 354.1469.

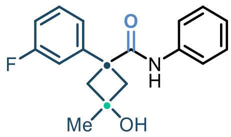

1-(3-fluorophenyl)-3-hydroxy-3-methyl-N-phenylcyclobutane-1-carboxamide (3al)
21.4 mg, 72% yield, white solid. Eluent: pentane/ethyl acetate = 3:1, R_f = 0.2.
^{1}H NMR (300 MHz, CDCl$_3$) δ 7.45 (td, *J* = 8.0, 5.9 Hz, 1H), 7.33 – 7.24 (m, 4H), 7.14 – 7.06 (m, 3H), 7.06 – 7.00 (m, 1H), 6.83 (br, 1H), 3.05 – 2.94 (m, 2H), 2.74 – 2.63 (m, 2H), 1.33 (s, 3H).
^{13}C NMR (75 MHz, CDCl$_3$) δ 176.1, 163.3 (d, *J* = 248.9 Hz), 145.9 (d, *J* = 6.6 Hz), 137.1, 131.2 (d, *J* = 8.3 Hz), 129.1, 125.2, 123.1 (d, *J* = 3.0 Hz), 120.4, 115.0 (d, *J* = 20.9 Hz), 114.7 (d, *J* = 21.6 Hz), 69.2, 48.0, 46.7 (d, *J* = 1.7 Hz), 28.8.
^{19}F NMR (282 MHz, CDCl$_3$) δ -110.47 – -110.72 (m).
HRMS (ESI-TOF): m/z calcd. for $[M + Na]^+$ $C_{18}H_{18}FNO_2Na^+$ 322.1214; Found: 322.1214.

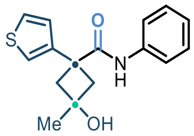

3-hydroxy-3-methyl-N-phenyl-1-(thiophen-3-yl)cyclobutane-1-carboxamide (3am)
20.8 mg, 72% yield, white solid. Eluent: pentane/ethyl acetate = 3:1, R_f = 0.2.
^{1}H NMR (300 MHz, CDCl$_3$) δ 7.47 (dd, *J* = 5.0, 2.9 Hz, 1H), 7.41 – 7.34 (m, 1H), 7.33 – 7.29 (m, 2H), 7.28 (h, *J* = 1.2 Hz, 1H), 7.23 (dd, *J* = 2.9, 1.4 Hz, 1H), 7.13 – 7.05 (m, 1H), 7.00 (dd, *J* = 5.0, 1.4 Hz, 2H), 3.03 – 2.86 (m, 2H), 2.74 – 2.61 (m, 2H), 1.32 (s, 3H).
^{13}C NMR (75 MHz, CDCl$_3$) δ 176.1, 144.5, 137.3, 129.1, 128.2, 127.0, 125.0, 122.7, 120.3, 69.5, 48.2, 43.5, 28.5.
HRMS (ESI-TOF): m/z calcd. for $[M + Na]^+$ $C_{16}H_{17}NO_2SNa^+$ 310.0872; Found: 310.0879.

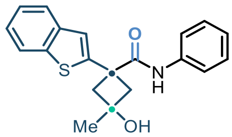

1-(benzo[b]thiophen-2-yl)-3-hydroxy-3-methyl-N-phenylcyclobutane-1-carboxamide (3an)
29.1 mg, 86% yield, white solid. Eluent: pentane/ethyl acetate = 3:1, R_f = 0.2.
^{1}H NMR (300 MHz, CDCl$_3$) δ 7.97 – 7.87 (m, 1H), 7.59 – 7.52 (m, 1H), 7.46 – 7.34 (m, 3H), 7.26 – 7.20 (m, 4H), 7.11 – 7.05 (m, 1H), 7.03 (br, 1H), 3.26 – 3.07 (m, 2H), 3.00 – 2.66 (m, 2H), 1.36 (s, 3H).
^{13}C NMR (75 MHz, CDCl$_3$) δ 175.8, 141.2, 138.0, 137.1, 137.0, 129.1, 125.4, 125.2, 125.0, 124.4, 123.4, 122.9, 120.7, 70.1, 47.4, 43.7, 28.8.
HRMS (ESI-TOF): m/z calcd. for $[M + Na]^+$ $C_{20}H_{19}NO_2SNa^+$ 360.1028; Found: 360.1031.

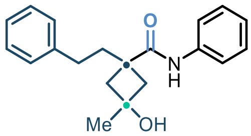

3-hydroxy-3-methyl-1-phenethyl-N-phenylcyclobutane-1-carboxamide (3ao)
7.7 mg, 25% yield, 95% purity, white solid. Eluent: Dichloromethane/ethyl acetate = 20:1, R_f = 0.3.
^{1}H NMR (300 MHz, CDCl$_3$) δ 7.76 (br, 1H), 7.54 – 7.49 (m, 2H), 7.36 – 7.29 (m, 3H), 7.24 – 7.15 (m, 4H), 7.13 – 7.06 (m, 1H), 2.74 – 2.62 (m, 2H), 2.64 – 2.60 (m, 2H), 2.41 – 2.37 (m, 2H), 2.17 (s, 3H), 1.84 – 1.68 (m, 2H).
^{13}C NMR (75 MHz, CDCl$_3$) δ 170.4, 141.8, 138.1, 129.1, 128.6, 128.5, 126.1, 124.3, 119.9, 47.6, 42.1, 36.4, 33.5, 32.2, 30.9.
HRMS (ESI-TOF): m/z calcd. for $[M + Na]^+$ $C_{20}H_{23}NO_2Na^+$ 332.1621; Found: 332.1621.

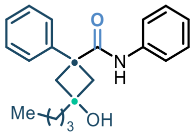

3-butyl-3-hydroxy-N,1-diphenylcyclobutane-1-carboxamide (3ap)
19.0 mg, 60% yield, white solid. Eluent: pentane/ethyl acetate = 8:1, R_f = 0.2.
^{1}H NMR (400 MHz, CDCl$_3$) δ 7.81 – 7.77 (m, 0.6H), 7.60 – 7.57 (m, 0.7H), 7.48 – 7.44 (m, 2H), 7.41 – 7.37 (m, 1H), 7.33 – 7.29 (m, 2H), 7.28 – 7.27 (m, 2H), 7.18 – 7.13 (m, 0.4H), 7.12 – 7.05 (m, 1H), 6.82 (br, 1H), 6.55 (t, *J* = 1.5 Hz, 0.3H), 4.82 (d, *J* = 1.5 Hz, 1H, -OH), 2.98 – 2.85 (m, 2H), 2.80 – 2.51 (m, 2H), 1.61 – 1.47 (m, 2H), 1.40 – 1.33 (m, 2H), 1.31 – 1.24 (m, 2H), 0.87 (t, *J* = 7.2 Hz, 3H).
^{13}C NMR (101 MHz, CDCl$_3$) δ 177.2, 143.6, 137.3, 130.7, 129.4, 129.3, 129.1, 127.9, 127.4, 126.0, 125.1, 124.2, 121.6, 120.3, 118.9, 71.5, 52.9, 47.3, 41.7, 25.5, 23.1, 14.2.
(The observed splitting and non-integer integrations in both **^{1}H** and **^{13}C NMR** spectra are attributed to the presence of rotamers resulting from restricted rotation at room temperature.)
HRMS (ESI-TOF): m/z calcd. for $[M + Na]^+$ $C_{21}H_{25}NO_2Na^+$ 346.1777; Found: 346.1783.

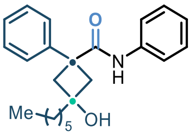

3-hexyl-3-hydroxy-N,1-diphenylcyclobutane-1-carboxamide (3aq)
19.1 mg, 57% yield, white solid. Eluent: pentane/ethyl acetate = 8:1, R_f = 0.2.
^{1}H NMR (300 MHz, CDCl$_3$) δ 7.84 – 7.75 (m, 0.6H), 7.63 – 7.55 (m, 0.7H), 7.50 – 7.43 (m, 2H), 7.42 – 7.37 (m, 1H), 7.33 – 7.29 (m, 2H), 7.27 (d, J = 2.7 Hz, 2H), 7.18 – 7.12 (m, 0.4H), 7.12 – 7.05 (m, 1H), 6.81 (br, 1H), 6.55 (t, J = 1.5 Hz, 0.3H), 4.82 (d, J = 1.5 Hz, 1H, -OH), 2.95 – 2.87 (m, 2H), 2.73 – 2.64 (m, 2H), 1.57 – 1.48 (m, 2H), 1.41 – 1.32 (m, 2H), 1.29 – 1.21 (m, 6H), 0.89 – 0.83 (m, 3H).
^{13}C NMR (75 MHz, CDCl$_3$) δ 177.2, 143.6, 137.3, 129.3, 129.1, 127.9, 127.4, 126.1, 125.1, 124.2, 120.3, 118.9, 71.6, 47.4, 46.9, 42.1, 32.0, 29.7, 23.3, 22.7, 14.2.
(The observed splitting and non-integer integrations in both **^{1}H** and **^{13}C NMR** spectra are attributed to the presence of rotamers resulting from restricted rotation at room temperature.)
HRMS (ESI-TOF): m/z calcd. for $[M + Na]^+$ $C_{23}H_{29}NO_2Na^+$ 374.2090; Found: 374.2098.

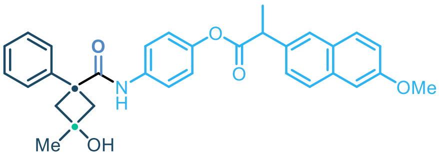

4-(3-hydroxy-3-methyl-1-phenylcyclobutane-1-carboxamido)phenyl (R)-2-(6-methoxynaphthalen-2-yl)propanoate (3at)
34.5 mg, 68% yield, white solid. Eluent: pentane/ethyl acetate = 2:1, R_f = 0.2.
^{1}H NMR (300 MHz, CDCl$_3$) δ 7.76 – 7.70 (m, 3H), 7.50 – 7.42 (m, 3H), 7.40 – 7.34 (m, 1H), 7.31 – 7.22 (m, 4H), 7.20 – 7.10 (m, 2H), 6.91 – 6.85 (m, 2H), 6.80 (br, 1H), 4.06 (q, J = 7.1 Hz, 1H), 3.92 (s, 3H), 3.05 – 2.91 (m, 2H), 2.79 – 2.63 (m, 2H), 1.66 (d, J = 7.2 Hz, 3H), 1.31 (s, 3H).
^{13}C NMR (75 MHz, CDCl$_3$) δ 176.9, 173.2, 157.9, 147.6, 143.2, 135.1, 134.9, 133.9, 129.5, 129.4, 129.1, 127.9, 127.5, 127.3, 126.2, 126.2, 122.0, 121.1, 119.2, 105.7, 69.3, 55.4, 48.0, 46.8, 45.6, 28.7, 18.6.
HRMS (ESI-TOF): m/z calcd. for $[M + Na]^+$ $C_{32}H_{31}NO_5Na^+$ 532.2094; Found: 532.2099.

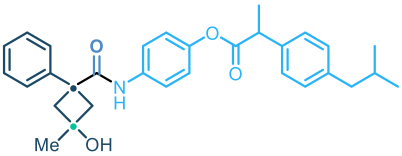

4-(3-hydroxy-3-methyl-1-phenylcyclobutane-1-carboxamido)phenyl 2-(4-isobutylphenyl)propanoate (3au)
31.7 mg, 65% yield, white solid. Eluent: pentane/ethyl acetate = 4:1, R_f = 0.2.
^{1}H NMR (300 MHz, CDCl$_3$) δ 7.50 – 7.44 (m, 2H), 7.41 – 7.34 (m, 1H), 7.32 – 7.30 (m, 1H), 7.29 – 7.23 (m, 5H), 7.16 – 7.09 (m, 2H), 6.93 – 6.85 (m, 2H), 6.80 (br, 1H), 3.89 (q, J = 7.1 Hz, 1H), 3.07 – 2.90 (m, 2H), 2.80 – 2.63 (m, 2H), 2.46 (d, J = 7.2 Hz, 2H), 1.86 (hept, J = 6.6 Hz, 1H), 1.57 (d, J = 7.1 Hz, 3H), 1.32 (s, 3H), 0.90 (d, J = 6.6 Hz, 6H).
^{13}C NMR (75 MHz, CDCl$_3$) δ 177.0, 173.3, 147.7, 143.2, 141.0, 137.2, 134.8, 129.6, 129.5, 127.9, 127.4, 127.3, 122.0, 121.1, 69.4, 48.0, 46.8, 45.3, 45.2, 30.3, 28.7, 22.5, 18.6.
HRMS (ESI-TOF): m/z calcd. for $[M + Na]^+$ $C_{31}H_{35}NO_4Na^+$ 508.2458; Found: 508.2455.

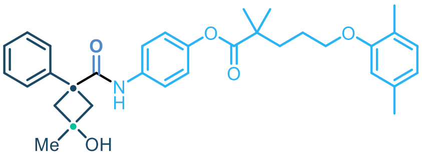

4-(3-hydroxy-3-methyl-1-phenylcyclobutane-1-carboxamido)phenyl 5-(2,5-dimethylphenoxy)-2,2-dimethylpentanoate (3av)
38.2 mg, 72% yield, white solid. Eluent: pentane/ethyl acetate = 4:1, R_f = 0.2.
^{1}H NMR (300 MHz, CDCl$_3$) δ 7.52 – 7.45 (m, 2H), 7.43 – 7.36 (m, 1H), 7.35 – 7.27 (m, 4H), 7.00 (d, J = 7.4 Hz, 1H), 6.96 – 6.90 (m, 2H), 6.84 (br, 1H), 6.71 – 6.59 (m, 2H), 4.02 – 3.92 (m, 2H), 3.07 – 2.93 (m, 2H), 2.83 – 2.62 (m, 2H), 2.30 (d, J = 0.8 Hz, 3H), 2.16 (s, 3H), 1.94 – 1.79 (m, 4H), 1.34 (d, J = 4.7 Hz, 9H).
^{13}C NMR (75 MHz, CDCl$_3$) δ 176.9, 176.4, 157.0, 147.8, 143.2, 136.6, 134.8, 130.4, 129.5, 127.9, 127.4, 123.7, 122.1, 121.2, 120.9, 112.0, 69.4, 67.8, 48.0, 46.8, 42.5, 37.2, 28.7, 25.4, 25.2, 21.5, 15.9.

HRMS (ESI-TOF): m/z calcd. for $[M + Na]^+$ $C_{33}H_{39}NO_5Na^+$ 552.2720; Found: 552.2728.

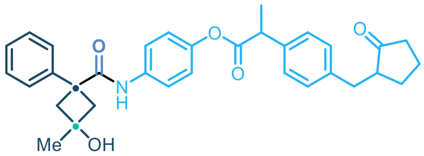

4-(3-hydroxy-3-methyl-1-phenylcyclobutane-1-carboxamido)phenyl 2-(4-((2-oxocyclopentyl)methyl)-phenyl)pro-panoate (3aw)

34.8 mg, 66% yield, white solid. Eluent: pentane/ethyl acetate = 3:2, R_f = 0.2.

^{1}H NMR (300 MHz, $CDCl_3$) δ 7.51 – 7.42 (m, 2H), 7.40 – 7.33 (m, 2H), 7.31 – 7.27 (m, 2H), 7.27 – 7.25 (m, 2H), 7.24 – 7.22 (m, 1H), 7.18 – 7.08 (m, 2H), 6.91 – 6.86 (m, 2H), 6.82 (br, 1H), 5.23 (br, 1H), 3.89 (q, *J* = 7.1 Hz, 1H), 3.13 (dd, *J* = 13.7, 4.1 Hz, 1H), 3.01 – 2.93 (m, 2H), 2.75 – 2.67 (m, 2H), 2.52 (dd, *J* = 13.8, 9.5 Hz, 1H), 2.39 – 2.27 (m, 2H), 2.17 – 2.03 (m, 3H), 1.99 – 1.90 (m, 1H), 1.81 – 1.66 (m, 1H), 1.56 (d, *J* = 7.1 Hz, 3H), 1.31 (s, 3H).

^{13}C NMR (75 MHz, $CDCl_3$) δ 220.3, 176.9, 173.1, 147.6, 143.2, 139.3, 137.9, 134.8, 129.5, 129.4, 127.9, 127.7, 127.3, 121.9, 121.1, 69.3, 51.1, 47.9, 46.8, 45.3, 38.3, 35.3, 29.3, 28.7, 20.6, 18.6.

HRMS (ESI-TOF): m/z calcd. for $[M + Na]^+$ $C_{33}H_{35}NO_5Na^+$ 548.2407; Found: 548.2417.

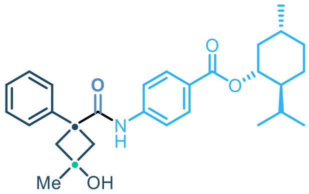

(1R,2S,5R)-2-isopropyl-5-methylcyclohexyl 4-(3-hydroxy-3-methyl-1-phenylcyclobutane-1-carboxamido)-benzoate (3ax)

39.4 mg, 85% yield, white solid. Eluent: pentane/ethyl acetate = 4:1, R_f = 0.2.

^{1}H NMR (300 MHz, $CDCl_3$) δ 8.00 – 7.86 (m, 2H), 7.55 – 7.44 (m, 2H), 7.43 – 7.29 (m, 5H), 7.02 (br, 1H), 4.87 (td, *J* = 10.8, 4.4 Hz, 1H), 3.07 – 2.94 (m, 2H), 2.78 – 2.68 (m, 2H), 2.12 – 2.05 (m, 1H), 1.95 – 1.82 (m, 1H), 1.76 – 1.65 (m, 2H), 1.61 – 1.44 (m, 2H), 1.32 (s, 3H), 1.16 – 1.00 (m, 2H), 0.89 (t, *J* = 7.1 Hz, 7H), 0.76 (d, *J* = 6.9 Hz, 3H).

^{13}C NMR (75 MHz, $CDCl_3$) δ 177.0, 165.5, 142.9, 141.2, 130.8, 129.6, 128.0, 127.3, 127.0, 119.2, 74.9, 69.3, 47.9, 47.3, 46.9, 41.1, 34.4, 31.5, 28.7, 26.7, 23.8, 22.1, 20.8, 16.7.

HRMS (ESI-TOF): m/z calcd. for $[M + Na]^+$ $C_{29}H_{37}NO_4Na^+$ 486.2614; Found: 486.2623.

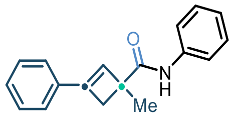

1-methyl-N,3-diphenylcyclobut-2-ene-1-carboxamide (4a)

21.4 mg, 81% yield, white solid. Eluent: pentane/ethyl acetate = 15:1, Rf = 0.2.

^{1}H NMR (300 MHz, $CDCl_3$) δ 7.63 (br, 1H), 7.55 – 7.49 (m, 2H), 7.46 – 7.34 (m, 5H), 7.33 – 7.27 (m, 2H), 7.13 – 7.04 (m, 1H), 6.53 (s, 1H), 3.17 (d, *J* = 13.4 Hz, 2H), 2.80 (d, *J* = 13.3 Hz, 2H), 1.64 (s, 3H).

^{13}C NMR (75 MHz, $CDCl_3$) δ 149.4, 138.1, 133.6, 129.4, 129.1, 129.0, 128.7, 125.1, 124.3, 119.9, 48.7, 42.0, 21.6.

HRMS (ESI-TOF): m/z calcd. for $[M + Na]^+$ $C_{18}H_{17}NONa^+$ 286.1202; Found: 286.1204.

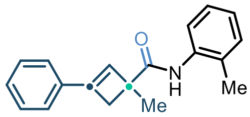

1-methyl-3-phenyl-N-(o-tolyl)cyclobut-2-ene-1-carboxamide (4b)

21.8 mg, 79% yield, white solid. Eluent: pentane/ethyl acetate = 15:1, Rf = 0.2.

^{1}H NMR (300 MHz, $CDCl_3$) δ 7.93 (d, *J* = 8.1 Hz, 1H), 7.60 (br, 1H), 7.45 – 7.34 (m, 5H), 7.25 – 7.11 (m, 2H), 7.04 (td, *J* = 7.4, 1.4 Hz, 1H), 6.55 (s, 1H), 3.18 (d, *J* = 13.3 Hz, 1H), 2.83 (d, *J* = 13.3 Hz, 1H), 2.17 (s, 3H), 1.65 (s, 3H).

^{13}C NMR (75 MHz, $CDCl_3$) δ 173.8, 149.6, 136.0, 133.6, 130.5, 129.4, 129.0, 128.7, 128.4, 127.0, 125.0, 124.9, 122.4, 48.8, 42.3, 21.4, 17.6.

HRMS (ESI-TOF): m/z calcd. for $[M + H]^+$ $C_{19}H_{19}NONa^+$ 300.1359; Found: 300.1368.

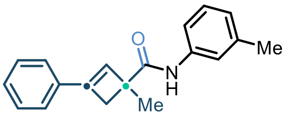

1-methyl-3-phenyl-N-(m-tolyl)cyclobut-2-ene-1-carboxamide (4c)
19.6 mg, 71% yield, white solid. Eluent: pentane/ethyl acetate = 15:1, Rf = 0.2.
^{1}H NMR (300 MHz, $CDCl_3$) δ 7.58 (br, 1H), 7.45 – 7.35 (m, 5H), 7.35 – 7.24 (m, 2H), 7.22 – 7.12 (m, 1H), 6.90 (d, *J* = 7.5 Hz, 1H), 6.52 (d, *J* = 1.0 Hz, 1H), 3.15 (d, *J* = 13.4 Hz, 1H), 2.79 (d, *J* = 13.3 Hz, 1H), 2.32 (s, 3H), 1.64 (s, 3H).
^{13}C NMR (75 MHz, $CDCl_3$) δ 173.9, 149.4, 139.0, 138.0, 133.6, 129.4, 129.0, 128.9, 128.7, 125.1, 125.1, 120.5, 116.9, 48.7, 42.1, 21.6, 21.6.
HRMS (ESI-TOF): m/z calcd. for $[M + H]^+$ $C_{19}H_{19}NONa^+$ 300.1359; Found: 300.1364.

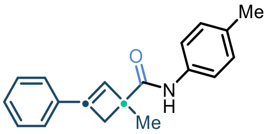

1-methyl-3-phenyl-N-(p-tolyl)cyclobut-2-ene-1-carboxamide (4d)
21.0 mg, 76% yield, white solid. Eluent: pentane/ethyl acetate = 10:1, Rf = 0.3.
^{1}H NMR (300 MHz, $CDCl_3$) δ 7.56 (br, 1H), 7.44 – 7.41 (m, 3H), 7.40 – 7.31 (m, 4H), 7.15 – 7.08 (m, 2H), 6.52 (s, 1H), 3.15 (d, *J* = 13.3 Hz, 1H), 2.78 (d, *J* = 13.3 Hz, 1H), 2.30 (s, 3H), 1.63 (s, 3H).
^{13}C NMR (75 MHz, $CDCl_3$) δ 173.8, 149.3, 135.5, 133.9, 133.6, 129.6, 129.5, 129.0, 128.7, 125.0, 120.0, 48.6, 42.0, 21.7, 21.0.
HRMS (ESI-TOF): m/z calcd. for $[M + Na]^+$ $C_{19}H_{19}NONa^+$ 300.1359; Found: 300.1365.

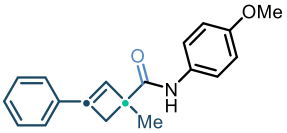

N-(4-methoxyphenyl)-1-methyl-3-phenylcyclobut-2-ene-1-carboxamide (4e)
23.0 mg, 78% yield, white solid. Eluent: pentane/ethyl acetate = 10:1, Rf = 0.2.
^{1}H NMR (300 MHz, $CDCl_3$) δ 7.52 (br, 1H), 7.45 – 7.39 (m, 5H), 7.38 – 7.33 (m, 2H), 6.88 – 6.81 (m, 2H), 6.52 (s, 1H), 3.78 (s, 3H), 3.15 (d, *J* = 13.3 Hz, 1H), 2.78 (d, *J* = 13.3 Hz, 1H), 1.63 (s, 3H).
^{13}C NMR (75 MHz, $CDCl_3$) δ 173.7, 156.4, 149.2, 133.6, 131.2, 129.5, 129.0, 128.7, 125.0, 121.8, 114.2, 55.6, 48.5, 42.1, 21.7.
HRMS (ESI-TOF): m/z calcd. for $[M + Na]^+$ $C_{19}H_{19}NO_2Na^+$ 316.1308; Found: 316.1309.

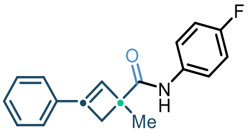

N-(4-fluorophenyl)-1-methyl-3-phenylcyclobut-2-ene-1-carboxamide (4f)
22.6 mg, 80% yield, white solid. Eluent: pentane/ethyl acetate = 20:1, Rf = 0.2.
^{1}H NMR (300 MHz, $CDCl_3$) δ 7.61 (br, 1H), 7.51 – 7.45 (m, 2H), 7.43 – 7.41 (m, 2H), 7.40 – 7.31 (m, 3H), 7.04 – 6.95 (m, 2H), 6.52 (s, 1H), 3.15 (d, *J* = 13.3 Hz, 1H), 2.80 (d, *J* = 13.3 Hz, 1H), 1.63 (s, 3H).
^{13}C NMR (75 MHz, $CDCl_3$) δ 173.9, 159.4 (d, *J* = 243.2 Hz), 149.5, 133.8 (d, *J* = 43.8 Hz), 129.2 (d, *J* = 12.8 Hz), 128.7, 125.1, 121.8 (d, *J* = 7.9 Hz), 115.7 (d, *J* = 22.5 Hz), 48.5, 42.1, 21.6.
^{19}F NMR (282 MHz, $CDCl_3$) δ -118.10 – -118.33 (m).
HRMS (ESI-TOF): m/z calcd. for $[M + Na]^+$ $C_{18}H_{16}FNONa^+$ 304.1108; Found: 304.1113.

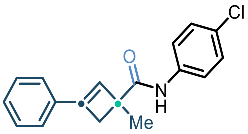

N-(4-chlorophenyl)-1-methyl-3-phenylcyclobut-2-ene-1-carboxamide (4g)
21.7 mg, 73% yield, white solid. Eluent: pentane/ethyl acetate = 15:1, Rf = 0.2.
^{1}H NMR (400 MHz, $CDCl_3$) δ 7.63 (br, 1H), 7.50 – 7.46 (m, 2H), 7.44 – 7.34 (m, 5H), 7.29 – 7.24 (m, 2H), 6.51 (s, 1H), 3.15 (d, *J* = 13.4 Hz, 1H), 2.80 (d, *J* = 13.3 Hz, 1H), 1.63 (s, 3H).

^{13}C NMR (101 MHz, CDCl$_3$) δ 173.9, 149.6, 136.7, 133.5, 129.2, 129.2, 129.1, 129.1, 128.7, 125.1, 121.1, 48.6, 42.1, 21.5.
HRMS (ESI-TOF): m/z calcd. for $[M + Na]^+$ $C_{18}H_{16}ClNONa^+$ 320.0812; Found: 320.0816.

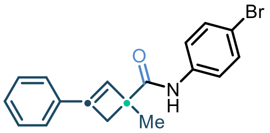

N-(4-bromophenyl)-1-methyl-3-phenylcyclobut-2-ene-1-carboxamide (4h)
23.1 mg, 69% yield, white solid. Eluent: pentane/ethyl acetate = 15:1, Rf = 0.2.
^{1}H NMR (400 MHz, CDCl$_3$) δ 7.63 (br, 1H), 7.45 – 7.41 (m, 6H), 7.40 – 7.35 (m, 3H), 6.51 (s, 1H), 3.14 (d, J = 13.3 Hz, 1H), 2.79 (d, J = 13.3 Hz, 1H), 1.63 (s, 3H).
^{13}C NMR (101 MHz, CDCl$_3$) δ 173.96, 149.58, 137.18, 133.45, 132.01, 129.16, 129.14, 128.73, 125.07, 121.47, 116.80, 48.63, 42.03, 21.50.
HRMS (ESI-TOF): m/z calcd. for $[M + Na]^+$ $C_{18}H_{16}BrNONa^+$ 364.0307; Found: 364.0313.

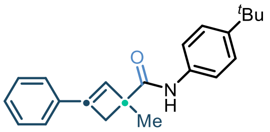

N-(4-(tert-butyl)phenyl)-1-methyl-3-phenylcyclobut-2-ene-1-carboxamide (4i)
19.6 mg, 64% yield, white solid. Eluent: pentane/ethyl acetate = 10:1, Rf = 0.3.
^{1}H NMR (300 MHz, CDCl$_3$) δ 7.57 (br, 1H), 7.48 – 7.39 (m, 5H), 7.38 – 7.28 (m, 4H), 6.53 (s, 1H), 3.15 (d, J = 13.3 Hz, 1H), 2.79 (d, J = 13.3 Hz, 1H), 1.63 (s, 3H), 1.30 (s, 9H).
^{13}C NMR (75 MHz, CDCl$_3$) δ 173.8, 149.3, 147.3, 135.5, 133.6, 129.5, 129.0, 128.7, 125.9, 125.0, 119.7, 48.6, 42.1, 34.5, 31.5, 21.7.
HRMS (ESI-TOF): m/z calcd. for $[M + H]^+$ $C_{22}H_{25}NONa^+$ 342.1828; Found: 342.1831.

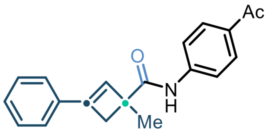

N-(4-acetylphenyl)-1-methyl-3-phenylcyclobut-2-ene-1-carboxamide (4j)
17.2 mg, 56% yield, white solid. Eluent: pentane/ethyl acetate = 7:1, Rf = 0.2.
^{1}H NMR (300 MHz, CDCl$_3$) δ 7.96 – 7.89 (m, 2H), 7.85 (br, 1H), 7.64 (2, 2H), 7.46 – 7.34 (m, 5H), 6.53 (s, 1H), 3.17 (d, J = 13.4 Hz, 1H), 2.81 (d, J = 13.4 Hz, 1H), 2.56 (s, 3H), 1.64 (s, 3H).
^{13}C NMR (75 MHz, CDCl$_3$) δ 197.1, 174.2, 149.7, 142.5, 133.4, 132.9, 129.8, 129.2, 129.1, 128.7, 125.1, 119.0, 48.8, 42.0, 26.6, 21.5.
HRMS (ESI-TOF): m/z calcd. for $[M + Na]^+$ $C_{20}H_{19}NO_2Na^+$ 328.1308; Found: 328.1312.

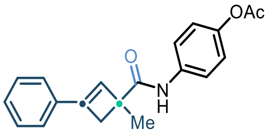

4-(1-methyl-3-phenylcyclobut-2-ene-1-carboxamido)phenyl acetate (4k)
18.6 mg, 58% yield, white solid. Eluent: pentane/ethyl acetate = 7:1, Rf = 0.3.
^{1}H NMR (300 MHz, CDCl$_3$) δ 8.03 – 7.95 (m, 2H), 7.81 (br, 1H), 7.64 – 7.58 (m, 2H), 7.46 – 7.32 (m, 5H), 6.52 (s, 1H), 3.89 (s, 3H), 3.17 (d, J = 13.4 Hz, 1H), 2.81 (d, J = 13.4 Hz, 1H), 1.64 (s, 3H).
^{13}C NMR (75 MHz, CDCl$_3$) δ 174.2, 166.7, 149.7, 142.3, 133.4, 130.9, 129.2, 129.1, 128.7, 125.6, 125.1, 118.9, 52.1, 48.8, 42.0, 21.4.
HRMS (ESI-TOF): m/z calcd. for $[M + Na]^+$ $C_{20}H_{19}NO_3Na^+$ 344.1257; Found: 344.1259.

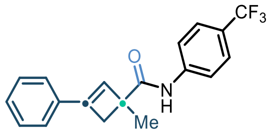

1-methyl-3-phenyl-N-(4-(trifluoromethyl)phenyl)cyclobut-2-ene-1-carboxamide (4l)
18.6 mg, 56% yield, white solid. Eluent: pentane/ethyl acetate = 15:1, Rf = 0.2.
^{1}H NMR (300 MHz, CDCl$_3$) δ 7.78 (br, 1H), 7.69 – 7.60 (m, 2H), 7.58 – 7.52 (m, 2H), 7.47 – 7.33 (m, 5H), 6.53 (s, 1H), 3.17 (d, *J* = 13.4 Hz, 1H), 2.82 (d, *J* = 13.4 Hz, 1H), 1.64 (s, 3H).
^{13}C NMR (75 MHz, CDCl$_3$) δ 174.2, 149.8, 141.2, 133.4, 129.2, 129.1, 128.8, 126.3 (q, *J* = 3.8 Hz), 125.1, 119.4, 48.7, 42.1, 21.4.
^{19}F NMR (282 MHz, CDCl$_3$) δ -62.07 (s).
HRMS (ESI-TOF): m/z calcd. for $[M + Na]^+$ $C_{19}H_{16}F3NONa^+$ 354.1076; Found: 354.1082.

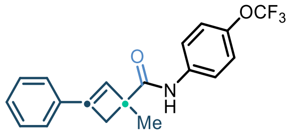

1-methyl-3-phenyl-N-(4-(trifluoromethoxy)phenyl)cyclobut-2-ene-1-carboxamide (4m)
20.7 mg, 60% yield, white solid. Eluent: pentane/ethyl acetate = 10:1, Rf = 0.3.
^{1}H NMR (300 MHz, CDCl$_3$) δ 7.60 (br, 1H), 7.51 – 7.44 (m, 2H), 7.38 – 7.27 (m, 5H), 7.13 – 7.03 (m, 2H), 6.44 (s, 1H), 3.07 (d, *J* = 13.4 Hz, 1H), 2.73 (d, *J* = 13.4 Hz, 1H), 1.56 (s, 3H).
^{13}C NMR (75 MHz, CDCl$_3$) δ 174.0, 149.6, 145.3, 136.8, 133.5, 129.2 (d, *J* = 2.0 Hz), 128.7, 125.1, 121.8, 121.1, 48.6, 42.1, 21.5.
^{19}F NMR (282 MHz, CDCl$_3$) δ -58.14 (s).
HRMS (ESI-TOF): m/z calcd. for $[M + Na]^+$ $C_{19}H_{16}F_3NO_2Na^+$ 370.1025; Found: 370.1031.

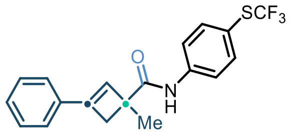

1-methyl-3-phenyl-N-(4-((trifluoromethyl)thio)phenyl)cyclobut-2-ene-1-carboxamide (4n)
24.4 mg, 67% yield, white solid. Eluent: pentane/ethyl acetate = 15:1, Rf = 0.2.
^{1}H NMR (300 MHz, CDCl$_3$) δ 7.76 (br, 1H), 7.66 – 7.55 (m, 4H), 7.48 – 7.33 (m, 5H), 6.52 (s, 1H), 3.16 (d, *J* = 13.4 Hz, 1H), 2.81 (d, *J* = 13.4 Hz, 1H), 1.64 (s, 3H).
^{13}C NMR (75 MHz, CDCl$_3$) δ 174.2, 149.8, 140.7, 137.6, 133.4, 131.7, 129.2, 129.0, 128.8, 125.1, 120.3, 118.7 (d, *J* = 2.1 Hz), 48.7, 42.0, 21.4.
^{19}F NMR (282 MHz, CDCl$_3$) δ -43.35 (s).
HRMS (ESI-TOF): m/z calcd. for $[M + H]^+$ $C_{19}H_{17}F_3NOSNa^+$ 364.0978; Found: 364.0985.

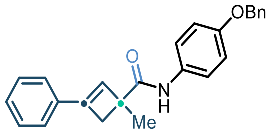

N-(4-(benzyloxy)phenyl)-1-methyl-3-phenylcyclobut-2-ene-1-carboxamide (4o)
22.0 mg, 60% yield, white solid. Eluent: pentane/ethyl acetate = 10:1, Rf = 0.3.
^{1}H NMR (300 MHz, CDCl$_3$) δ 7.54 (br, 1H), 7.46 – 7.39 (m, 8H), 7.39 – 7.31 (m, 4H), 6.96 – 6.88 (m, 2H), 6.52 (s, 1H), 5.04 (s, 2H), 3.15 (d, *J* = 13.3 Hz, 1H), 2.79 (d, *J* = 13.3 Hz, 1H), 1.63 (s, 3H).
^{13}C NMR (75 MHz, CDCl$_3$) δ 173.7, 155.6, 149.2, 137.1, 133.6, 131.5, 129.5, 129.0, 128.7, 128.1, 127.6, 125.0, 121.7, 115.3, 70.4, 48.5, 42.1, 21.7.
HRMS (ESI-TOF): m/z calcd. for $[M + H]^+$ $C_{25}H_{23}NO_2Na^+$ 392.1621; Found: 392.1624.

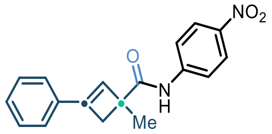

1-methyl-N-(4-nitrophenyl)-3-phenylcyclobut-2-ene-1-carboxamide (4p)
12.6 mg, 62% yield, yellow oil. Eluent: pentane/ethyl acetate = 10:1, Rf = 0.3.

^{1}H NMR (300 MHz, CDCl$_3$) δ 8.23 – 8.15 (m, 2H), 7.96 (s, 1H), 7.77 – 7.67 (m, 2H), 7.45 – 7.35 (m, 5H), 6.52 (s, 1H), 3.17 (d, J = 13.5 Hz, 1H), 2.84 (d, J = 13.5 Hz, 1H), 1.92 – 1.26 (m, 3H).
^{13}C NMR (75 MHz, CDCl$_3$) δ 174.4, 150.1, 143.9, 143.6, 133.2, 129.4, 128.8, 128.8, 125.2, 125.1, 119.2, 48.8, 42.0, 21.3.
HRMS (ESI-TOF): m/z calcd. for [M + H]$^+$ $C_{18}H_{16}N_2O_3Na^+$ 309.1234; Found: 309.1240.

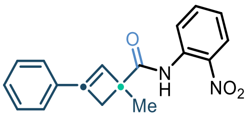

1-methyl-N-(2-nitrophenyl)-3-phenylcyclobut-2-ene-1-carboxamide (4q)
14.2 mg, 46% yield, yellow oil. Eluent: pentane/ethyl acetate = 15:1, Rf = 0.2.
^{1}H NMR (300 MHz, CDCl$_3$) δ 10.93 (br, 1H), 8.85 (dd, J = 8.6, 1.4 Hz, 1H), 8.21 (dd, J = 8.5, 1.6 Hz, 1H), 7.65 (ddd, J = 8.6, 7.2, 1.6 Hz, 1H), 7.53 – 7.43 (m, 2H), 7.42 – 7.30 (m, 3H), 7.16 (ddd, J = 8.5, 7.2, 1.4 Hz, 1H).
^{13}C NMR (75 MHz, CDCl$_3$) δ 175.4, 149.4, 136.2, 136.1, 135.4, 133.6, 129.0, 129.0, 128.7, 125.9, 125.2, 123.1, 122.1, 49.4, 41.8, 21.6.
HRMS (ESI-TOF): m/z calcd. for [M + Na]$^+$ $C_{18}H_{16}N_2O_3Na^+$ 331.1053; Found: 331.1063.

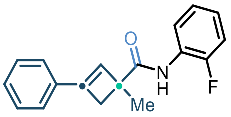

N-(2-fluorophenyl)-1-methyl-3-phenylcyclobut-2-ene-1-carboxamide (4r)
12.6 mg, 45% yield, white solid. Eluent: pentane/ethyl acetate = 10:1, Rf = 0.4.
^{1}H NMR (300 MHz, CDCl$_3$) δ 8.35 (td, J = 7.9, 1.5 Hz, 1H), 7.89 (br, 1H), 7.46 – 7.30 (m, 5H), 7.17 – 7.08 (m, 1H), 7.07 – 7.00 (m, 2H), 6.53 (s, 1H), 3.18 (d, J = 13.3 Hz, 1H), 2.81 (d, J = 13.3 Hz, 1H), 1.65 (s, 3H).
^{13}C NMR (75 MHz, CDCl$_3$) δ 174.1, 154.2, 151.0, 149.5, 133.6, 129.0, 129.0, 128.7, 125.1, 124.7 (d, J = 3.7 Hz), 124.3 (d, J = 7.7 Hz), 121.7, 114.8 (d, J = 19.2 Hz), 48.9, 42.0, 21.7.
^{19}F NMR (282 MHz, CDCl$_3$) δ -131.6 – -131.7 (m).
HRMS (ESI-TOF): m/z calcd. for [M + H]$^+$ $C_{18}H_{16}FNONa^+$ 304.1108; Found: 304.1114.

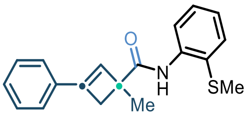

1-methyl-N-(2-(methylthio)phenyl)-3-phenylcyclobut-2-ene-1-carboxamide (4s)
19.8 mg, 64% yield, white solid. Eluent: pentane/ethyl acetate = 20:1, Rf = 0.3.
^{1}H NMR (300 MHz, CDCl$_3$) δ 9.02 (br, 1H), 8.51 – 8.19 (m, 1H), 7.48 – 7.42 (m, 3H), 7.41 – 7.33 (m, 3H), 7.32 – 7.27 (m, 1H), 7.03 (td, J = 7.6, 1.4 Hz, 1H), 6.58 (s, 1H), 3.19 (d, J = 13.3 Hz, 1H), 2.84 (d, J = 13.3 Hz, 1H), 2.20 (s, 3H), 1.66 (s, 3H).
^{13}C NMR (75 MHz, CDCl$_3$) δ 174.1, 149.1, 138.9, 133.7, 133.4, 129.5, 129.3, 128.9, 128.6, 125.2, 125.0, 124.1, 120.2, 49.2, 42.1, 21.5, 18.7.
HRMS (ESI-TOF): m/z calcd. for [M + Na]$^+$ $C_{19}H_{19}NOSNa^+$ 332.1079; Found: 332.1077.

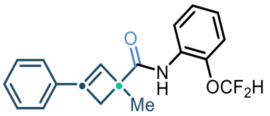

N-(2-(difluoromethoxy)phenyl)-1-methyl-3-phenylcyclobut-2-ene-1-carboxamide (4t)
25.6 mg, 78% yield, white solid. Eluent: pentane/ethyl acetate = 10:1, Rf = 0.3.
^{1}H NMR (300 MHz, CDCl$_3$) δ 8.43 (dd, J = 8.3, 1.3 Hz, 1H), 8.23 (br, 1H), 7.46 – 7.32 (m, 5H), 7.26 – 7.19 (m, 1H), 7.10 – 7.01 (m, 2H), 6.52 (s, 1H), 6.36 (t, J = 73.5 Hz, 1H), 3.16 (d, J = 13.3 Hz, 1H), 2.82 (d, J = 13.3 Hz, 1H), 1.64 (s, 3H).
^{13}C NMR (75 MHz, CDCl$_3$) δ 174.1, 149.5, 140.3, 133.6, 130.7, 129.1, 129.0, 128.6, 126.6, 125.0, 124.0, 121.3, 119.2, 116.6 (t, J = 261.0 Hz), 49.0, 42.0, 21.3.
^{19}F NMR (282 MHz, CDCl$_3$) δ -79.56(s, 1F), -79.82(s, 1F).
HRMS (ESI-TOF): m/z calcd. for [M + Na]$^+$ $C_{19}H_{17}F_2NO_2Na^+$ 352.1119; Found: 352.1122.

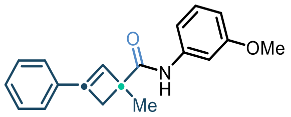

N-(3-methoxyphenyl)-1-methyl-3-phenylcyclobut-2-ene-1-carboxamide (4u)
21.6 mg, 74% yield, white solid. Eluent: pentane/ethyl acetate = 8:1, Rf = 0.3.
^{1}H NMR (300 MHz, $CDCl_3$) δ 7.61 (br, 1H), 7.46 – 7.40 (m, 3H), 7.39 – 7.33 (m, 3H), 7.18 (t, *J* = 8.1 Hz, 1H), 6.93 (ddd, *J* = 8.0, 2.0, 0.9 Hz, 1H), 6.65 (ddd, *J* = 8.3, 2.5, 0.9 Hz, 1H), 6.52 (s, 1H), 3.80 (s, 3H), 3.16 (d, *J* = 13.3 Hz, 1H), 2.79 (d, *J* = 13.4 Hz, 1H), 1.63 (s, 3H).
^{13}C NMR (75 MHz, $CDCl_3$) δ 174.0, 160.3, 149.5, 139.4, 133.6, 129.7, 129.3, 129.1, 128.7, 125.1, 111.8, 110.6, 105.2, 55.5, 48.7, 42.1, 21.6.
HRMS (ESI-TOF): m/z calcd. for $[M + Na]^+$ $C_{19}H_{19}NO_2Na^+$ 316.1308; Found: 316.1307.

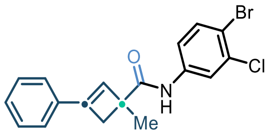

N-(4-bromo-3-chlorophenyl)-1-methyl-3-phenylcyclobut-2-ene-1-carboxamide (4v)
27.4 mg, 73% yield, white solid. Eluent: pentane/ethyl acetate = 10:1, Rf = 0.2.
^{1}H NMR (300 MHz, $CDCl_3$) δ 7.78 (d, *J* = 2.5 Hz, 1H), 7.66 (br, 1H), 7.50 (d, *J* = 8.7 Hz, 1H), 7.45 – 7.35 (m, 5H), 7.29 (dd, *J* = 8.7, 2.5 Hz, 1H), 6.49 (s, 1H), 3.14 (d, *J* = 13.4 Hz, 1H), 2.80 (d, *J* = 13.4 Hz, 1H), 1.62 (s, 3H).
^{13}C NMR (75 MHz, $CDCl_3$) δ 174.1, 149.8, 138.3, 134.8, 133.8, 133.3, 129.2, 129.0, 128.7, 125.1, 121.5, 119.3, 116.7, 48.6, 42.0, 21.4.
HRMS (ESI-TOF): m/z calcd. for $[M + H]^+$ $C_{18}H_{15}BrClNONa^+$ 397.9918; Found: 397.9922.

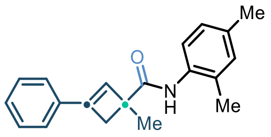

N-(2,4-dimethylphenyl)-1-methyl-3-phenylcyclobut-2-ene-1-carboxamide (4w)
21.0 mg, 72% yield, white solid. Eluent: pentane/ethyl acetate = 10:1, Rf = 0.3.
^{1}H NMR (300 MHz, $CDCl_3$) δ 7.47 – 7.34 (m, 5H), 7.20 (br, 1H), 7.09 – 7.02 (m, 3H), 6.58 (s, 1H), 3.25 (d, *J* = 13.2 Hz, 1H), 2.82 (d, *J* = 13.2 Hz, 1H), 2.21 (s, 6H), 1.66 (s, 3H).
^{13}C NMR (75 MHz, $CDCl_3$) δ 173.9, 148.8, 135.5, 133.9, 133.7, 129.8, 128.9, 128.7, 128.3, 127.3, 125.0, 48.4, 42.1, 22.0, 18.5.
HRMS (ESI-TOF): m/z calcd. for $[M + H]^+$ $C_{20}H_{21}NONa^+$ 314.1515; Found: 314.1514.

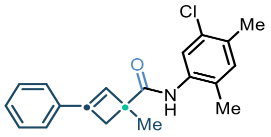

N-(5-chloro-2,4-dimethylphenyl)-1-methyl-3-phenylcyclobut-2-ene-1-carboxamide (4x)
24.7 mg, 76% yield, white solid. Eluent: pentane/ethyl acetate = 5:1, Rf = 0.3.
^{1}H NMR (300 MHz, $CDCl_3$) δ 8.45 (s, 1H), 8.06 (br, 1H), 7.45 – 7.32 (m, 5H), 6.51 (s, 1H), 6.48 (s, 1H), 3.87 (s, 3H), 3.81 (s, 3H), 3.14 (d, *J* = 13.2 Hz, 1H), 2.78 (d, *J* = 13.2 Hz, 1H), 1.62 (s, 3H).
^{13}C NMR (75 MHz, $CDCl_3$) δ 173.6, 151.3, 149.0, 147.8, 133.8, 129.3, 128.8, 128.6, 125.0, 121.8, 121.6, 114.0, 96.7, 56.8, 56.3, 48.9, 42.0, 21.8.
HRMS (ESI-TOF): m/z calcd. for $[M + H]^+$ $C_{20}H_{20}ClNONa^+$ 348.1126; Found: 348.1128.

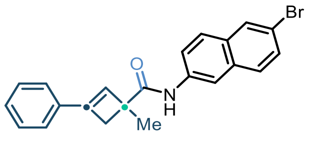

N-(6-bromonaphthalen-2-yl)-1-methyl-3-phenylcyclobut-2-ene-1-carboxamide (4y)
22.3 mg, 57% yield, white solid. Eluent: pentane/ethyl acetate = 10:1, Rf = 0.3.

^{1}H NMR (300 MHz, CDCl3) δ 8.27 (d, J = 2.1 Hz, 1H), 7.93 (d, J = 1.9 Hz, 1H), 7.83 (br, 1H), 7.67 (t, J = 9.0 Hz, 2H), 7.53 (dd, J = 8.7, 2.0 Hz, 1H), 7.50 – 7.38 (m, 6H), 6.59 (s, 1H), 3.23 (d, J = 13.4 Hz, 1H), 2.86 (d, J = 13.4 Hz, 1H), 1.70 (s, 3H).
^{13}C NMR (75 MHz, CDCl3) δ 174.2, 149.6, 135.9, 133.5, 132.5, 131.6, 130.0, 129.7, 129.4, 129.2, 129.1, 128.7, 127.9, 125.1, 120.9, 118.8, 116.5, 48.7, 42.1, 21.6.
HRMS (ESI-TOF): m/z calcd. for $[M + H]^+$ $C_{22}H_{18}BrNONa^+$ 414.0464; Found: 414.0467.

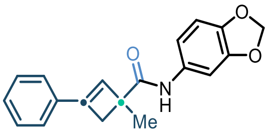

N-(benzo[d][1,3]dioxol-5-yl)-1-methyl-3-phenylcyclobut-2-ene-1-carboxamide (4z)
27.2 mg, 89% yield, white solid. Eluent: pentane/ethyl acetate = 10:1, Rf = 0.3.
^{1}H NMR (300 MHz, $CDCl_3$) δ 7.53 (br, 1H), 7.47 – 7.36 (m, 5H), 7.27 (d, *J* = 2.1 Hz, 1H), 6.82 – 6.65 (m, 2H), 6.50 (s, 1H), 5.93 (s, 2H), 3.14 (d, *J* = 13.3 Hz, 1H), 2.78 (d, *J* = 13.3 Hz, 1H), 1.62 (d, *J* = 0.4 Hz, 3H).
^{13}C NMR (75 MHz, $CDCl_3$) δ 173.8, 149.3, 147.9, 144.3, 133.6, 132.4, 129.3, 129.0, 128.7, 125.0, 113.1, 108.1, 102.9, 101.3, 48.5, 42.0, 21.6.
HRMS (ESI-TOF): m/z calcd. for $[M + H]^+$ $C_{19}H_{17}NO_3Na^+$ 330.1101; Found: 330.1108.

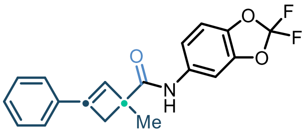

N-(2,2-difluorobenzo[d][1,3]dioxol-5-yl)-1-methyl-3-phenylcyclobut-2-ene-1-carboxamide (4ab)
21.1 mg, 62% yield, white solid. Eluent: pentane/ethyl acetate = 15:1, Rf = 0.2.
^{1}H NMR (400 MHz, $CDCl_3$) δ 7.68 – 7.61 (m, 2H), 7.45 – 7.35 (m, 5H), 6.94 (d, *J* = 1.3 Hz, 2H), 6.51 (s, 1H), 3.14 (d, *J* = 13.4 Hz, 1H), 2.80 (d, *J* = 13.4 Hz, 1H), 1.63 (s, 3H).
^{13}C NMR (101 MHz, $CDCl_3$) δ 174.0, 149.7, 144.0, 140.2, 134.2, 133.4, 131.9, 129.2, 129.0, 128.8, 125.1, 114.7, 109.3, 103.3, 48.5, 42.1, 21.5.
^{19}F NMR (376 MHz, $CDCl_3$) δ -50.00 (s).
HRMS (ESI-TOF): m/z calcd. for $[M + Na]^+$ $C_{19}H_{15}F_2NO_3Na^+$ 366.0912; Found: 366.0922.

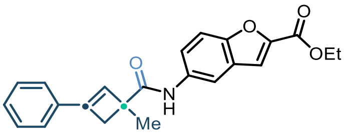

ethyl 5-(1-methyl-3-phenylcyclobut-2-ene-1-carboxamido)benzofuran-2-carboxylate (4ac)
27.8 mg, 74% yield, white solid. Eluent: pentane/ethyl acetate = 10:1, Rf = 0.3.
^{1}H NMR (400 MHz, $CDCl_3$) δ 8.10 (d, *J* = 2.2 Hz, 1H), 7.76 (s, 1H), 7.48 (d, *J* = 8.9 Hz, 1H), 7.45 (d, *J* = 1.0 Hz, 1H), 7.44 – 7.38 (m, 3H), 7.37 – 7.32 (m, 2H), 6.54 (s, 1H), 4.43 (q, *J* = 7.1 Hz, 2H), 3.18 (d, *J* = 13.4 Hz, 1H), 2.81 (d, *J* = 13.3 Hz, 1H), 1.65 (s, 3H), 1.42 (t, *J* = 7.1 Hz, 3H).
^{13}C NMR (101 MHz, $CDCl_3$) δ 174.1, 159.6, 152.7, 149.5, 146.6, 134.2, 133.5, 129.2, 129.1, 128.7, 127.5, 125.0, 121.1, 114.0, 113.9, 112.5, 61.7, 48.6, 42.1, 21.6, 14.5.
HRMS (ESI-TOF): m/z calcd. for $[M + Na]^+$ $C_{23}H_{21}NO_4Na^+$ 398.1363; Found: 398.1370.

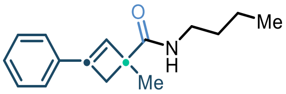

N-butyl-1-methyl-3-phenylcyclobut-2-ene-1-carboxamide (4ad)
14.8 mg, 61% yield, white solid. Eluent: pentane/ethyl acetate = 8:1, Rf = 0.3.
^{1}H NMR (300 MHz, $CDCl_3$) δ 7.39 – 7.30 (m, 5H), 6.40 (d, *J* = 4.0 Hz, 1H), 5.84 (br, 1H), 3.33 – 3.19 (m, 2H), 3.00 (d, *J* = 13.2 Hz, 1H), 2.67 (dd, *J* = 15.7, 13.0 Hz, 1H), 1.56 (d, *J* = 10.7 Hz, 3H), 1.50 – 1.41 (m, 2H), 1.37 – 1.25 (m, 2H), 0.91 (t, *J* = 7.3 Hz, 3H).
^{13}C NMR (75 MHz, $CDCl_3$) δ 175.8, 148.4, 133.8, 129.8, 128.7, 128.6, 124.9, 47.9, 42.0, 39.5, 31.9, 21.9, 20.2, 13.9.
HRMS (ESI-TOF): m/z calcd. for $[M + H]^+$ $C_{16}H_{21}NONa^+$ 266.1515; Found: 266.1517.

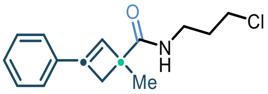

N-(3-chloropropyl)-1-methyl-3-phenylcyclobut-2-ene-1-carboxamide (4ae)
10.0 mg, 38% yield, white solid. Eluent: pentane/ethyl acetate = 5:1, Rf = 0.3.
^{1}H NMR (300 MHz, $CDCl_3$) δ 7.40 – 7.31 (m, 5H), 6.40 (s, 1H), 6.06 (br, 1H), 3.56 (t, *J* = 6.3 Hz, 2H), 3.41 (qd, *J* = 6.6, 2.6 Hz, 2H), 3.00 (d, *J* = 13.2 Hz, 1H), 2.70 (d, *J* = 13.2 Hz, 1H), 1.99 (p, *J* = 6.5 Hz, 2H), 1.54 (s, 3H).
^{13}C NMR (75 MHz, $CDCl_3$) δ 176.1, 148.5, 133.7, 129.6, 128.8, 128.6, 124.9, 47.8, 41.9, 37.4, 32.3, 21.8.
HRMS (ESI-TOF): m/z calcd. for $[M + H]^+$ $C_{15}H_{18}ClNONa^+$ 286.0969; Found: 286.0974.

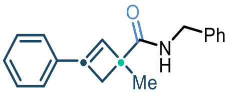

N-benzyl-1-methyl-3-phenylcyclobut-2-ene-1-carboxamide (4af)
19.8 mg, 71% yield, white solid. Eluent: pentane/ethyl acetate = 10:1, Rf = 0.3.
^{1}H NMR (300 MHz, $CDCl_3$) δ 7.39 – 7.34 (m, 4H), 7.33 – 7.30 (m, 2H), 7.30 – 7.23 (m, 4H), 6.41 (s, 1H), 6.17 (br, 1H), 4.60 – 4.33 (m, 2H), 3.06 (d, *J* = 13.2 Hz, 1H), 2.73 (d, *J* = 13.1 Hz, 1H), 1.59 (s, 3H).
^{13}C NMR (75 MHz, $CDCl_3$) δ 175.6, 148.4, 138.6, 133.7, 129.6, 128.8, 128.8, 128.6, 127.8, 127.6, 124.9, 47.9, 43.7, 41.9, 22.0.
HRMS (ESI-TOF): m/z calcd. for $[M + H]^+$ $C_{19}H_{19}NONa^+$ 300.1359; Found: 300.1364.

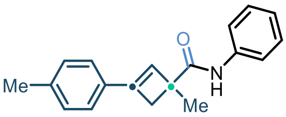

1-methyl-N-phenyl-3-(p-tolyl)cyclobut-2-ene-1-carboxamide (4ag)
22.1 mg, 80% yield, white solid. Eluent: pentane/ethyl acetate = 15:1, Rf = 0.2.
^{1}H NMR (300 MHz, $CDCl_3$) δ 7.65 (br, 1H), 7.56 – 7.49 (m, 2H), 7.35 – 7.27 (m, 4H), 7.23 – 7.17 (m, 2H), 7.13 – 7.04 (m, 1H), 6.46 (s, 1H), 3.14 (d, *J* = 13.3 Hz, 1H), 2.77 (d, *J* = 13.3 Hz, 1H), 2.39 (s, 3H), 1.63 (s, 3H).
^{13}C NMR (75 MHz, $CDCl_3$) δ 174.1, 149.4, 139.1, 138.1, 130.9, 129.4, 129.1, 128.2, 125.0, 124.2, 119.9, 48.6, 42.1, 21.6, 21.6.
HRMS (ESI-TOF): m/z calcd. for $[M + H]^+$ $C_{19}H_{19}NONa^+$ 300.1359.; Found: 300.1364.

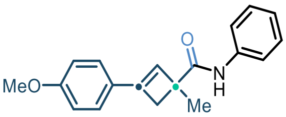

3-(4-methoxyphenyl)-1-methyl-N-phenylcyclobut-2-ene-1-carboxamide (4ah)
19.4 mg, 66% yield, white solid. Eluent: pentane/ethyl acetate = 8:1, Rf = 0.2.
^{1}H NMR (300 MHz, $CDCl_3$) δ 7.66 (br, 1H), 7.55 – 7.49 (m, 2H), 7.39 – 7.34 (m, 2H), 7.32 – 7.25 (m, 2H), 7.14 – 7.02 (m, 1H), 6.93 – 6.89 (m, 2H), 6.36 (s, 1H), 3.84 (s, 3H), 3.12 (d, *J* = 13.3 Hz, 1H), 2.76 (d, *J* = 13.3 Hz, 1H), 1.62 (s, 3H).
^{13}C NMR (75 MHz, $CDCl_3$) δ 174.2, 160.3, 149.0, 138.2, 129.1, 128.0, 126.7, 126.6, 124.2, 119.9, 114.1, 55.5, 48.4 (d, *J* = 1.9 Hz), 42.1, 21.7.
HRMS (ESI-TOF): m/z calcd. for $[M + H]^+$ $C_{19}H_{19}NO_2Na^+$ 316.1308.; Found: 316.1312.

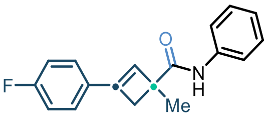

3-(4-fluorophenyl)-1-methyl-N-phenylcyclobut-2-ene-1-carboxamide (4ai)
14.2 mg, 51% yield, white solid. Eluent: pentane/ethyl acetate = 10:1, Rf = 0.3.
^{1}H NMR (300 MHz, $CDCl_3$) δ 7.59 (br, 1H), 7.56 – 7.49 (m, 2H), 7.43 – 7.35 (m, 2H), 7.34 – 7.27 (m, 2H), 7.13 – 7.03 (m, 3H), 6.46 (s, 1H), 3.15 (d, *J* = 13.3 Hz, 1H), 2.77 (d, *J* = 13.3 Hz, 1H), 1.63 (s, 3H).
^{13}C NMR (75 MHz, $CDCl_3$) δ 173.8, 163.1 (d, *J* = 249.0 Hz), 148.2, 138.0, 130.0 (d, *J* = 3.3 Hz), 129.1, 128.8 (d, *J* = 2.4 Hz), 126.9 (d, *J* = 8.4 Hz), 124.4, 119.9, 115.8 (d, *J* = 21.9 Hz), 48.6, 42.0, 21.7.
^{19}F NMR (282 MHz, $CDCl_3$) δ -111.54 – -111.69 (m).
HRMS (ESI-TOF): m/z calcd. for $[M + Na]^+$ $C_{18}H_{16}FNONa^+$ 304.1108; Found: 304.1113.

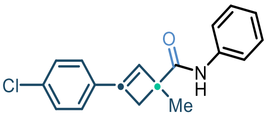

3-(4-chlorophenyl)-1-methyl-N-phenylcyclobut-2-ene-1-carboxamide (4aj)
22.1 mg, 80% yield, white solid. Eluent: pentane/ethyl acetate = 10:1, Rf = 0.2.
^{1}H NMR (300 MHz, $CDCl_3$) δ 7.57 (br, 1H), 7.55 – 7.50 (m, 2H), 7.38 – 7.33 (m, 4H), 7.32 – 7.27 (m, 2H), 7.13 – 7.06 (m, 1H), 6.52 (s, 1H), 3.15 (d, *J* = 13.3 Hz, 1H), 2.76 (d, *J* = 13.3 Hz, 1H), 1.63 (s, 3H).
^{13}C NMR (75 MHz, $CDCl_3$) δ 173.6, 148.1, 138.0, 134.8, 132.1, 130.0, 129.1, 128.9, 126.4, 124.4, 119.9, 48.7, 41.9, 21.7.
HRMS (ESI-TOF): m/z calcd. for $[M + H]^+$ $C_{18}H_{16}ClNONa^+$ 320.0812.; Found: 320.0818.

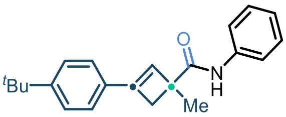

3-(4-(tert-butyl)phenyl)-1-methyl-N-phenylcyclobut-2-ene-1-carboxamide (4ak)
19.0 mg, 60% yield, white solid. Eluent: pentane/ethyl acetate = 10:1, Rf = 0.2.
^{1}H NMR (300 MHz, $CDCl_3$) δ 7.66 (br, 1H), 7.55 – 7.48 (m, 2H), 7.46 – 7.38 (m, 4H), 7.34 – 7.27 (m, 2H), 7.13 – 7.04 (m, 1H), 6.48 (s, 1H), 3.14 (d, *J* = 13.3 Hz, 1H), 2.78 (d, *J* = 13.3 Hz, 1H), 1.63 (s, 3H), 1.34 (s, 9H).
^{13}C NMR (75 MHz, $CDCl_3$) δ 174.1, 152.4, 149.4, 138.1, 130.9, 129.1, 128.5, 125.6, 124.9, 124.2, 119.8, 48.6, 42.1, 34.9, 31.4, 21.6.
HRMS (ESI-TOF): m/z calcd. for $[M + H]^+$ $C_{22}H_{25}NONa^+$ 342.1828.; Found: 342.1826.

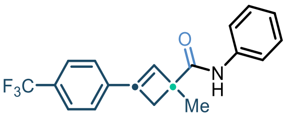

1-methyl-N-phenyl-3-(4-(trifluoromethyl)phenyl)cyclobut-2-ene-1-carboxamide (4al)
13.4 mg, 40% yield, white solid. Eluent: pentane/ethyl acetate = 10:1, Rf = 0.2.
^{1}H NMR (300 MHz, $CDCl_3$) δ 7.69 – 7.60 (m, 2H), 7.55 – 7.48 (m, 5H), 7.38 – 7.28 (m, 2H), 7.16 – 7.05 (m, 1H), 6.66 (s, 1H), 3.20 (d, *J* = 13.3 Hz, 1H), 2.81 (d, *J* = 13.3 Hz, 1H), 1.66 (s, 3H).
^{13}C NMR (75 MHz, $CDCl_3$) δ 173.3, 147.9, 137.9, 136.7, 132.3, 129.1, 125.7 (q, *J* = 3.8 Hz), 125.3, 124.5, 120.0, 49.0, 41.9, 21.8.
^{19}F NMR (282 MHz, $CDCl_3$) δ -62.64.
HRMS (ESI-TOF): m/z calcd. for $[M + Na]^+$ $C_{19}H_{16}F_3NONa^+$ 354.1076; Found: 354.1084.

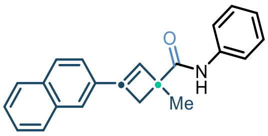

1-methyl-3-(naphthalen-2-yl)-N-phenylcyclobut-2-ene-1-carboxamide (4am)
22.9 mg, 73% yield, white solid. Eluent: pentane/ethyl acetate = 10:1, Rf = 0.2.
^{1}H NMR (300 MHz, $CDCl_3$) δ 7.88 – 7.80 (m, 3H), 7.75 (br, 1H), 7.69 – 7.59 (m, 2H), 7.57 – 7.46 (m, 4H), 7.36 – 7.27 (m, 2H), 7.19 – 7.00 (m, 1H), 6.63 (s, 1H), 3.28 (d, *J* = 13.3 Hz, 1H), 2.91 (d, *J* = 13.2 Hz, 1H), 1.68 (s, 3H).
^{13}C NMR (75 MHz, $CDCl_3$) δ 173.9, 149.4, 138.1, 133.6, 133.3, 131.0, 130.0, 129.1, 128.4, 128.0, 126.8, 124.5, 124.3, 122.5, 119.9, 48.8, 42.1, 21.7.
HRMS (ESI-TOF): m/z calcd. for $[M + Na]^+$ $C_{22}H_{19}NONa^+$ 336.1359; Found: 336.1361.

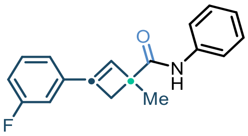

3-(3-fluorophenyl)-1-methyl-N-phenylcyclobut-2-ene-1-carboxamide (4an)
21.7 mg, 77% yield, white solid. Eluent: pentane/ethyl acetate = 20:1, Rf = 0.2.
^{1}H NMR (300 MHz, $CDCl_3$) δ 7.59 – 7.49 (m, 3H), 7.38 – 7.27 (m, 3H), 7.18 (dt, *J* = 7.7, 1.2 Hz, 1H), 7.13 – 7.06 (m, 2H), 7.06 – 6.99 (m, 1H), 6.56 (s, 1H), 3.15 (d, *J* = 13.3 Hz, 1H), 2.77 (d, *J* = 13.3 Hz, 1H), 1.64 (s, 3H).

^{13}C NMR (75 MHz, CDCl$_3$) δ 173.5, 163.1 (d, *J* = 246.6 Hz), 148.2, 138.0, 135.7 (d, *J* = 7.5 Hz), 130.9, 130.3 (d, *J* = 8.3 Hz), 129.1, 124.4, 120.8 (d, *J* = 3.0 Hz), 119.9, 115.9 (d, *J* = 21.5 Hz), 111.9 (d, *J* = 21.7 Hz), 48.8, 41.9, 21.7.
^{19}F NMR (282 MHz, CDCl$_3$) δ -112.61 – -113.06 (m).
HRMS (ESI-TOF): m/z calcd. for $[M + Na]^+$ $C_{18}H_{16}FNONa^+$ 304.1108; Found: 304.1115.

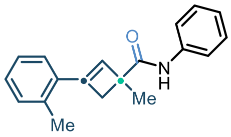

1-methyl-N-phenyl-3-(o-tolyl)cyclobut-2-ene-1-carboxamide (4ao)
23.0 mg, 83% yield, white solid. Eluent: pentane/ethyl acetate = 15:1, Rf = 0.2.
^{1}H NMR (300 MHz, CDCl$_3$) δ 7.68 (br, 1H), 7.57 – 7.49 (m, 2H), 7.36 – 7.31 (m, 2H), 7.28 – 7.24 (m, 4H), 7.13 – 7.06 (m, 1H), 6.43 (s, 1H), 3.27 (d, *J* = 13.2 Hz, 1H), 2.88 (d, *J* = 13.2 Hz, 1H), 2.50 (d, *J* = 0.5 Hz, 3H), 1.66 (s, 3H).
^{13}C NMR (75 MHz, CDCl$_3$) δ 173.9, 149.2, 138.1, 137.3, 133.3, 132.2, 131.0, 129.1, 128.8, 127.1, 126.2, 124.3, 119.9, 48.9, 43.6, 22.1, 21.8.
HRMS (ESI-TOF): m/z calcd. for $[M + H]^+$ $C_{19}H_{19}NONa^+$ 300.1359.; Found: 300.1364.

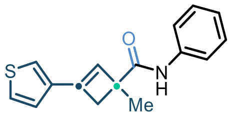

1-methyl-N-phenyl-3-(thiophen-3-yl)cyclobut-2-ene-1-carboxamide (4ap)
16.4 mg, 61% yield, white solid. Eluent: pentane/ethyl acetate = 10:1, Rf = 0.2.
^{1}H NMR (300 MHz, CDCl$_3$) δ 7.64 (br, 1H), 7.56 – 7.50 (m, 2H), 7.37 – 7.32 (m, 1H), 7.31 – 7.26 (m, 3H), 7.23 (dd, *J* = 4.9, 1.3 Hz, 1H), 7.13 – 7.05 (m, 1H), 6.28 (s, 1H), 3.13 (d, *J* = 13.2 Hz, 1H), 2.77 (d, *J* = 13.2 Hz, 1H), 1.63 (s, 3H).
^{13}C NMR (75 MHz, CDCl$_3$) δ 173.9, 144.6, 138.1, 136.7, 129.1, 128.1, 126.7, 125.0, 124.3, 122.9, 119.9, 49.6, 42.8, 21.6.
HRMS (ESI-TOF): m/z calcd. for $[M + H]^+$ $C_{16}H_{15}NOSNa^+$ 292.0766.; Found: 292.0772.

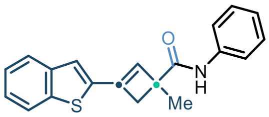

3-(benzo[b]thiophen-2-yl)-1-methyl-N-phenylcyclobut-2-ene-1-carboxamide (4aq)
28.6 mg, 90% yield, white solid. Eluent: pentane/ethyl acetate = 10:1, Rf = 0.2.
^{1}H NMR (300 MHz, CDCl$_3$) δ 8.10 – 8.02 (m, 1H), 7.90 (ddd, *J* = 7.7, 1.4, 0.7 Hz, 1H), 7.72 (br, 1H), 7.56 – 7.50 (m, 2H), 7.50 – 7.45 (m, 1H), 7.43 – 7.39 (m, 2H), 7.34 – 7.27 (m, 2H), 7.13 – 7.06 (m, 1H), 6.59 (s, 1H), 3.31 (d, *J* = 13.1 Hz, 1H), 2.94 (d, *J* = 13.1 Hz, 1H), 2.07 – 1.37 (m, 3H).
^{13}C NMR (75 MHz, CDCl$_3$) δ 173.8, 143.8, 140.6, 138.1, 137.0, 131.1, 129.1, 129.0, 126.2, 125.0, 125.0, 124.3, 123.2, 123.1, 119.9, 50.5, 43.6, 21.8.
HRMS (ESI-TOF): m/z calcd. for $[M + H]^+$ $C_{20}H_{17}NOSNa^+$ 342.0923.; Found: 342.0929.

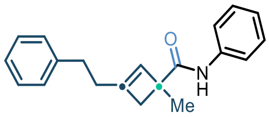

1-methyl-3-phenethyl-N-phenylcyclobut-2-ene-1-carboxamide (4ar)
14.9 mg, 51% yield, white solid. Eluent: pentane/ethyl acetate = 10:1, Rf = 0.2.
^{1}H NMR (300 MHz, CDCl$_3$) δ 7.43 – 7.38 (m, 2H), 7.32 (d, *J* = 1.9 Hz, 1H), 7.30 – 7.28 (m, 2H), 7.28 – 7.26 (m, 1H), 7.24 – 7.20 (m, 3H), 7.11 – 7.05 (m, 1H), 5.94 (t, *J* = 1.5 Hz, 1H), 2.88 – 2.81 (m, 2H), 2.74 (d, *J* = 13.5 Hz, 1H), 2.54 – 2.45 (m, 2H), 2.40 (d, *J* = 13.5 Hz, 1H), 2.17 (s, 3H).
^{13}C NMR (75 MHz, CDCl$_3$) δ 174.3, 153.7, 141.3, 138.1, 131.9, 129.0, 128.7, 128.4, 126.3, 124.1, 119.9, 49.1, 44.6, 32.7, 32.5, 21.3.
HRMS (ESI-TOF): m/z calcd. for $[M + Na]^+$ $C_{20}H_{21}NONa^+$ 314.1515; Found: 314.1522.

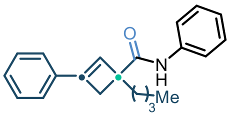

1-butyl-N,3-diphenylcyclobut-2-ene-1-carboxamide (4as)
14.7 mg, 48% yield, white solid. Eluent: pentane/ethyl acetate = 30:1, Rf = 0.2.
^{1}H NMR (300 MHz, CDCl$_3$) δ 7.57 – 7.51 (m, 3H), 7.44 – 7.41 (m, 2H), 7.40 – 7.38 (m, 1H), 7.37 – 7.33 (m, 2H), 7.32 – 7.27 (m, 2H), 7.14 – 7.04 (m, 1H), 6.62 (s, 1H), 3.09 (d, *J* = 13.4 Hz, 1H), 2.81 (d, *J* = 13.4 Hz, 1H), 2.42 – 2.28 (m, 1H), 1.75 – 1.60 (m, 1H), 1.48 – 1.31 (m, 4H), 0.95 – 0.88 (m, 3H).
^{13}C NMR (75 MHz, CDCl$_3$) δ 173.5, 150.2, 138.1, 133.6, 129.1, 129.0, 128.7, 127.5, 125.0, 124.3, 119.9, 52.8, 40.8, 36.1, 28.5, 23.3, 14.2.
HRMS (ESI-TOF): m/z calcd. for [M + Na]$^+$ $C_{21}H_{24}NO^+$ 306.1853; Found: 306.1848.

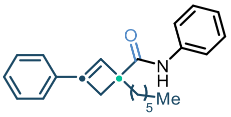

1-hexyl-N,3-diphenylcyclobut-2-ene-1-carboxamide (4at)
14.6 mg, 44% yield, white solid. Eluent: pentane/ethyl acetate = 30:1, Rf = 0.2.
^{1}H NMR (300 MHz, CDCl$_3$) δ 7.55 – 7.50 (m, 2H), 7.46 – 7.38 (m, 4H), 7.37 – 7.28 (m, 4H), 7.13 – 7.05 (m, 1H), 6.61 (s, 1H), 3.09 (d, *J* = 13.4 Hz, 1H), 2.81 (d, *J* = 13.3 Hz, 1H), 2.40 – 2.26 (m, 1H), 1.74 – 1.60 (m, 1H), 1.45 – 1.16 (m, 8H), 1.01 – 0.82 (m, 3H).
^{13}C NMR (75 MHz, CDCl$_3$) δ 173.5, 150.2, 138.1, 133.6, 129.1, 129.0, 128.7, 127.5, 125.0, 124.3, 119.9, 52.9, 40.8, 36.4, 31.9, 29.9, 26.3, 22.8, 14.2.
HRMS (ESI-TOF): m/z calcd. for [M + Na]$^+$ $C_{23}H_{28}NO^+$ 334.2166; Found: 334.2160.

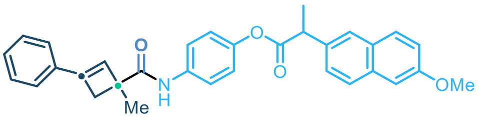

4-(1-methyl-3-phenylcyclobut-2-ene-1-carboxamido)phenyl (2R)-2-(6-methoxynaphthalen-2-yl)propanoate (4aw)
39.7 mg, 81% yield, white solid. Eluent: pentane/ethyl acetate = 10:1, Rf = 0.2.
^{1}H NMR (300 MHz, CDCl$_3$) δ 7.81 – 7.69 (m, 3H), 7.61 (br, 1H), 7.52 – 7.45 (m, 3H), 7.44 – 7.32 (m, 5H), 7.20 – 7.10 (m, 2H), 6.97 – 6.89 (m, 2H), 6.49 (s, 1H), 4.07 (q, *J* = 7.1 Hz, 1H), 3.92 (s, 3H), 3.13 (d, *J* = 13.3 Hz, 1H), 2.77 (d, *J* = 13.3 Hz, 1H), 1.68 (d, *J* = 7.2 Hz, 3H), 1.61 (s, 3H).
^{13}C NMR (75 MHz, CDCl$_3$) δ 173.8, 173.4, 157.9, 149.4, 147.0, 135.7, 135.2, 133.9, 133.5, 129.5, 129.3, 129.1, 129.0, 128.7, 127.5, 126.3, 126.2, 125.1, 121.9, 120.7, 119.2, 105.7, 55.5, 48.6, 45.7, 42.0, 21.6, 18.7.
HRMS (ESI-TOF): m/z calcd. for [M + Na]$^+$ $C_{29}H_3NO_3Na^+$ 466.2353; Found: 466.2357.

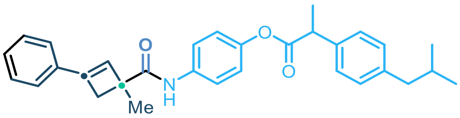

4-(1-methyl-3-phenylcyclobut-2-ene-1-carboxamido)phenyl 2-(4-isobutylphenyl)propanoate (4ax)
38.4 mg, 82% yield, white solid. Eluent: pentane/ethyl acetate = 10:1, Rf = 0.2.
^{1}H NMR (300 MHz, CDCl$_3$) δ 7.63 (br, 1H), 7.52 – 7.46 (m, 2H), 7.44 – 7.33 (m, 5H), 7.32 – 7.25 (m, 2H), 7.17 – 7.12 (m, 2H), 6.99 – 6.90 (m, 2H), 6.50 (s, 1H), 3.91 (q, *J* = 7.1 Hz, 1H), 3.14 (d, *J* = 13.3 Hz, 1H), 2.78 (d, *J* = 13.3 Hz, 1H), 2.47 (d, *J* = 7.2 Hz, 2H), 1.87 (dp, *J* = 13.6, 6.7 Hz, 1H), 1.71 – 1.41 (m, 6H), 0.91 (d, *J* = 6.6 Hz, 6H).
^{13}C NMR (75 MHz, CDCl$_3$) δ 173.8, 173.4, 149.4, 147.1, 140.9, 137.3, 135.7, 133.5, 129.6, 129.3, 129.0, 128.7, 127.3, 125.1, 121.9, 120.7, 48.6, 45.3, 45.2, 42.0, 30.3, 22.5, 21.6, 18.7.
HRMS (ESI-TOF): m/z calcd. for [M + H]$^+$ $C_{31}H_{33}NO_3Na^+$ 490.2352.; Found: 490.2357.

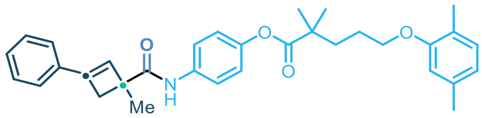

4-(1-methyl-3-phenylcyclobut-2-ene-1-carboxamido)phenyl 5-(2,5-dimethylphenoxy)-2,2-dimethyl-pentanoate (4ay)
42.6 mg, 83% yield, white solid. Eluent: pentane/ethyl acetate = 10:1, Rf = 0.2.
^{1}H NMR (300 MHz, CDCl$_3$) δ 7.65 (br, 1H), 7.57 – 7.50 (m, 2H), 7.46 – 7.38 (m, 4H), 7.37 – 7.34 (m, 1H), 7.04 – 6.94 (m, 3H), 6.73 – 6.59 (m, 2H), 6.52 (s, 1H), 4.13 – 3.79 (m, 2H), 3.16 (d, *J* = 13.3 Hz, 1H), 2.80 (d, *J* = 13.3 Hz, 1H), 2.31 (d, *J* = 0.8 Hz, 3H), 2.18 (s, 3H), 1.88 (d, *J* = 3.0 Hz, 4H), 1.64 (s, 3H), 1.36 (s, 6H).
^{13}C NMR (75 MHz, CDCl$_3$) δ 176.5, 173.8, 157.0, 149.4, 147.2, 136.6, 135.7, 133.5, 130.5, 129.3, 129.0, 128.7, 125.1, 123.7, 122.0, 120.9, 120.8, 112.1, 67.9, 48.6, 42.5, 42.0, 37.3, 25.4, 25.3, 21.6, 21.5, 15.9.
HRMS (ESI-TOF): m/z calcd. for [M + H]$^+$ $C_{33}H_{37}NO_4Na^+$ 534.2615.; Found: 534.2618.

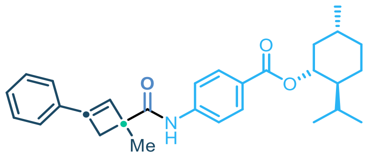

2-isopropyl-5-methylcyclohexyl 4-(1-methyl-3-phenylcyclobut-2-ene-1-carboxamido)benzoate (4az)
36.1 mg, 81% yield, white solid. Eluent: pentane/ethyl acetate = 10:1, Rf = 0.2.
^{1}H NMR (300 MHz, CDCl$_3$) δ 8.03 – 7.94 (m, 2H), 7.83 (br, 1H), 7.65 – 7.56 (m, 2H), 7.43 – 7.33 (m, 5H), 6.53 (s, 1H), 4.91 (td, *J* = 10.8, 4.4 Hz, 1H), 3.17 (d, *J* = 13.4 Hz, 1H), 2.81 (d, *J* = 13.4 Hz, 1H), 2.17 – 2.07 (m, 1H), 2.00 – 1.88 (m, 1H), 1.77 – 1.68 (m, 2H), 1.64 (s, 3H), 1.60 – 1.49 (m, 2H), 1.19 – 1.02 (m, 2H), 0.91 (dd, *J* = 6.8, 3.5 Hz, 7H), 0.78 (d, *J* = 6.9 Hz, 3H).
^{13}C NMR (75 MHz, CDCl$_3$) δ 174.1, 165.7, 149.6, 142.1, 133.4, 130.8, 129.1, 128.7, 128.7, 126.3, 125.1, 118.9, 74.8, 48.8, 47.4, 42.0, 41.1, 34.4, 31.6, 26.6, 23.8, 22.2, 21.5, 20.9, 16.7.
HRMS (ESI-TOF): m/z calcd. for [M + H]$^+$ $C_{29}H_{35}NO_3Na^+$ 468.2509.; Found: 468.2507.

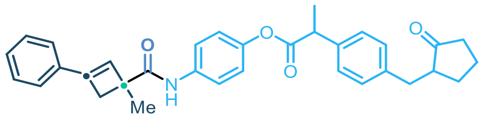

4-(1-methyl-3-phenylcyclobut-2-ene-1-carboxamido)phenyl 2-(4-((2-oxocyclopentyl)methyl)phenyl)-propanoate (4bb)
34.8 mg, 71% yield, white solid. Eluent: pentane/ethyl acetate = 3:2, Rf = 0.2.
^{1}H NMR (300 MHz, CDCl$_3$) δ 7.62 (br, 1H), 7.53 – 7.46 (m, 2H), 7.44 – 7.33 (m, 5H), 7.31 – 7.27 (m, 2H), 7.19 – 7.13 (m, 2H), 6.97 – 6.90 (m, 2H), 6.50 (s, 1H), 3.91 (q, *J* = 7.1 Hz, 1H), 3.14 (dd, *J* = 13.6, 4.3 Hz, 2H), 2.78 (d, *J* = 13.3 Hz, 1H), 2.53 (dd, *J* = 13.8, 9.5 Hz, 1H), 2.45 – 2.25 (m, 2H), 2.22 – 2.04 (m, 3H), 1.96 (dddd, *J* = 12.3, 6.2, 4.5, 2.6 Hz, 1H), 1.80 – 1.68 (m, 1H), 1.62 (s, 3H), 1.58 (d, *J* = 7.2 Hz, 3H).
^{13}C NMR (75 MHz, CDCl$_3$) δ 220.3, 173.8, 173.3, 149.5, 147.0, 139.3, 138.0, 135.7, 133.5, 129.4, 129.3, 129.1, 128.7, 127.7, 125.1, 121.9, 120.7, 51.1, 48.6, 45.3, 42.1, 38.3, 35.3, 29.4, 21.6, 20.7, 18.7.
HRMS (ESI-TOF): m/z calcd. for [M + Na]$^+$ $C_{33}H_{33}NO_4Na^+$ 530.2302; Found: 530.2299.

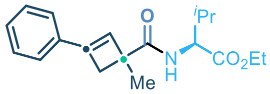

ethyl (1-methyl-3-phenylcyclobut-2-ene-1-carbonyl)valinate (4bc)
22.1 mg, 70% yield, white solid. Eluent: pentane/ethyl acetate = 7:1, Rf = 0.2.
^{1}H NMR (300 MHz, CDCl$_3$) δ 7.42 – 7.30 (m, 5H), 6.45 (d, *J* = 7.4 Hz, 1H), 6.30 (t, *J* = 10.4 Hz, 1H), 4.53 (ddd, *J* = 14.0, 8.8, 5.0 Hz, 1H), 4.27 – 4.06 (m, 2H), 3.07 (dd, *J* = 19.2, 13.1 Hz, 1H), 2.71 (dd, *J* = 13.1, 10.5 Hz, 1H), 2.25 – 2.08 (m, 1H), 1.55 (d, *J* = 3.6 Hz, 3H), 1.26 (dt, *J* = 12.7, 7.1 Hz, 3H), 0.98 – 0.82 (m, 6H).
^{13}C NMR (75 MHz, CDCl$_3$) δ 175.8, 175.6, 172.4, 172.2, 148.5, 148.3, 133.8, 129.5, 129.5, 128.7, 128.7, 128.6, 128.6, 125.0, 125.0, 61.3, 61.3, 57.1, 56.8, 47.9, 47.9, 42.1, 41.5, 31.6, 31.3, 22.0, 21.8, 19.1, 18.0, 17.9, 14.3, 14.3.
(Duplicated ^{13}C resonances and minor duplication of certain ^{1}H signals are attributed to slowly interconverting amide rotamers; no diastereomeric mixture was detected by ^{1}H NMR)
HRMS (ESI-TOF): m/z calcd. for [M + H]$^+$ $C_{19}H_{25}NO_3Na^+$ 338.1726; Found: 338.1729.

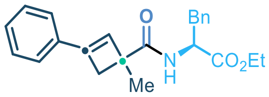

ethyl (1-methyl-3-phenylcyclobut-2-ene-1-carbonyl)phenylalaninate (4bd)
23.1 mg, 64% yield, white solid. Eluent: pentane/ethyl acetate = 7:1, Rf = 0.2.
^{1}H NMR (300 MHz, CDCl$_3$) δ 7.42 – 7.28 (m, 5H), 7.26 – 7.19 (m, 2H), 7.13 – 7.06 (m, 2H), 7.05 – 6.99 (m, 1H), 6.33 (d, *J* = 16.4 Hz, 1H), 6.23 (d, *J* = 8.0 Hz, 1H), 4.84 (ddt, *J* = 21.7, 7.8, 6.2 Hz, 1H), 4.16 (dq, *J* = 10.8, 7.1 Hz, 2H), 3.23 – 2.96 (m, 2H), 2.64 (dd, *J* = 13.1, 7.4 Hz, 1H), 1.50 (d, *J* = 2.9 Hz, 3H), 1.23 (dt, *J* = 13.7, 7.1 Hz, 3H).
^{13}C NMR (75 MHz, CDCl$_3$) δ 175.4, 175.2, 171.9, 171.7, 148.5, 148.3, 136.1, 133.8, 133.7, 129.5, 129.4, 129.2, 128.8, 128.7, 128.6, 128.6, 128.5, 127.2, 127.1, 125.0, 125.0, 61.6, 61.5, 53.1, 52.8, 47.7, 47.7, 41.9, 41.4, 38.2, 37.8, 21.7, 21.4, 14.3, 14.2.
(Duplicated ^{13}C resonances and minor duplication of certain ^{1}H signals are attributed to slowly interconverting amide rotamers; no diastereomeric mixture was detected by ^{1}H NMR)
HRMS (ESI-TOF): m/z calcd. for $[M + H]^+$ $C_{23}H_{25}NO_3Na^+$ 386.1726; Found: 386.1733.

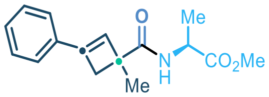

methyl (1-methyl-3-phenylcyclobut-2-ene-1-carbonyl)alaninate (4de)
18.5 mg, 68% yield, white solid. Eluent: pentane/ethyl acetate = 8:1, Rf = 0.2.
^{1}H NMR (300 MHz, CDCl$_3$) δ 7.43 – 7.29 (m, 5H), 6.44 (d, *J* = 9.7 Hz, 1H), 6.40 – 6.27 (m, 1H), 4.67 – 4.49 (m, 1H), 3.73 (d, *J* = 8.8 Hz, 3H), 3.06 (dd, *J* = 25.2, 13.2 Hz, 1H), 2.70 (dd, *J* = 13.1, 4.9 Hz, 1H), 1.54 (d, *J* = 0.6 Hz, 3H), 1.39 (t, *J* = 7.3 Hz, 3H).
^{13}C NMR (75 MHz, CDCl$_3$) δ 175.6, 175.3, 173.8, 173.7, 148.5, 148.3, 133.8, 129.5, 129.5, 128.8, 128.7, 128.6, 128.6, 125.0, 125.0, 52.6, 52.5, 48.1, 48.1, 47.6, 47.6, 41.8, 41.6, 21.8, 21.7, 18.5, 18.4.
(Duplicated ^{13}C resonances and minor duplication of certain ^{1}H signals are attributed to slowly interconverting amide rotamers; no diastereomeric mixture was detected by ^{1}H NMR)
HRMS (ESI-TOF): m/z calcd. for $[M + H]^+$ $C_{16}H_{19}NO_3Na^+$ 296.1257; Found: 296.1260.

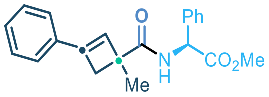

methyl 2-(1-methyl-3-phenylcyclobut-2-ene-1-carboxamido)-2-phenylacetate (4bf)
22.0 mg, 66% yield, white solid. Eluent: pentane/ethyl acetate = 7:1, Rf = 0.2.
^{1}H NMR (300 MHz, CDCl$_3$) δ 7.42 – 7.35 (m, 5H), 7.34 – 7.28 (m, 5H), 6.78 (dd, *J* = 30.3, 7.2 Hz, 1H), 6.45 (d, *J* = 19.1 Hz, 1H), 5.57 (dd, *J* = 7.2, 5.4 Hz, 1H), 3.72 (d, *J* = 5.2 Hz, 3H), 3.07 (dd, *J* = 58.2, 13.2 Hz, 1H), 2.69 (t, *J* = 13.3 Hz, 1H), 1.55 (d, *J* = 4.0 Hz, 3H).
^{13}C NMR (75 MHz, CDCl$_3$) δ 175.3, 175.1, 171.6, 171.6, 148.6, 148.5, 136.7, 136.5, 133.8, 133.7, 129.4, 129.3, 129.2, 129.1, 128.8, 128.7, 128.6, 127.5, 127.3, 125.1, 125.0, 56.6, 56.5, 52.9, 52.8, 47.6, 47.6, 41.7, 41.6, 21.8, 21.7.
(Duplicated ^{13}C resonances and minor duplication of certain ^{1}H signals are attributed to slowly interconverting amide rotamers; no diastereomeric mixture was detected by ^{1}H NMR)
HRMS (ESI-TOF): m/z calcd. for $[M + H]^+$ $C_{21}H_{21}NO_3Na^+$ 358.1413.; Found: 358.1417.

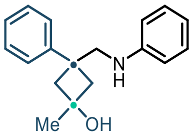

1-methyl-3-phenyl-3-((phenylamino)methyl)cyclobutan-1-ol (5)
30.1 mg, 57% yield, pale yellow solid. Eluent: pentane/ethyl acetate = 6:1, Rf = 0.2.
^{1}H NMR (300 MHz, CDCl$_3$) δ 7.33 – 7.25 (m, 2H), 7.20 – 7.13 (m, 3H), 7.09 – 7.01 (m, 2H), 6.64 – 6.56 (m, 1H), 6.54 – 6.47 (m, 2H), 3.32 (s, 2H), 2.59 – 2.25 (m, 4H), 1.16 (s, 3H).
^{13}C NMR (75 MHz, CDCl$_3$) δ 148.7, 146.8, 129.3, 128.6, 126.6, 126.3, 117.7, 113.3, 69.6, 56.4, 46.4, 38.9, 29.9.

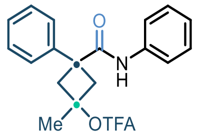

1-methyl-3-phenyl-3-(phenylcarbamoyl)cyclobutyl 2,2,2-trifluoroacetate (6)
30.5 mg, 86% yield, white solid. Eluent: pentane/ethyl acetate = 20:1, Rf = 0.2.
^{1}H NMR (300 MHz, CDCl$_3$) δ 7.53 – 7.37 (m, 5H), 7.32 – 7.21 (m, 4H), 7.10 – 7.02 (m, 1H), 6.71 (br, 1H), 3.56 – 3.43 (m, 2H), 2.98 – 2.89 (m, 2H), 1.42 (s, 3H).
^{13}C NMR (75 MHz, CDCl$_3$) δ 172.2, 140.5, 137.6, 129.8, 129.1, 128.2, 127.3, 124.7, 119.8, 80.3, 46.6, 44.5, 23.9.
^{19}F NMR (282 MHz, CDCl$_3$) δ -75.40 (s).
HRMS (ESI-TOF): m/z calcd. for $[M + H]^+$ $C_{20}H_{18}F_3NO_3Na^+$ 400.1131.; Found: 400.1137.

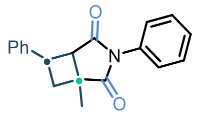

1-methyl-3,6-diphenyl-3-azabicyclo[3.2.0]heptane-2,4-dione (7a)
18.5 mg, 64% yield, white solid. Eluent: pentane/ethyl acetate = 10:1, R_f = 0.3, r.r.> 19:1, d.r. > 20:1.
^{1}H NMR (300 MHz, CDCl$_3$) δ 7.55 – 7.48 (m, 2H), 7.46 – 7.42 (m, 1H), 7.40 (dd, *J* = 2.3, 1.3 Hz, 2H), 7.38 – 7.28 (m, 5H), 3.70 (td, *J* = 8.7, 5.4 Hz, 1H), 3.26 (d, *J* = 5.5 Hz, 1H), 2.97 (dddd, *J* = 12.7, 9.3, 3.5, 0.9 Hz, 1H), 2.50 (dd, *J* = 12.8, 8.2 Hz, 0H), 1.57 (s, 1H).
^{13}C NMR (75 MHz, CDCl$_3$) δ 181.4, 177.2, 142.2, 132.3, 129.3, 129.0, 128.8, 127.2, 126.6, 126.6, 52.0, 41.7, 39.5, 36.9, 21.0.
HRMS (ESI-TOF): m/z calcd. for $[M + Na]^+$ $C_{19}H_{17}NO_2Na^+$ 314.1151; Found: 314.1151.

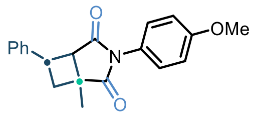

3-(4-methoxyphenyl)-1-methyl-6-phenyl-3-azabicyclo[3.2.0]heptane-2,4-dione (7b)
18.4 mg, 57% yield, white solid. Eluent: pentane/ethyl acetate = 8:1, R_f = 0.3, r.r.> 19:1, d.r. > 20:1.
^{1}H NMR (300 MHz, CDCl$_3$) δ 7.45 – 7.39 (m, 2H), 7.37 – 7.33 (m, 2H), 7.33 – 7.27 (m, 3H), 7.07 – 6.99 (m, 2H), 3.87 (s, 3H), 3.70 (td, *J* = 8.7, 5.4 Hz, 1H), 3.26 (d, *J* = 5.5 Hz, 1H), 2.98 (dddd, *J* = 12.8, 9.3, 3.5, 0.9 Hz, 1H), 2.51 (dd, *J* = 12.8, 8.2 Hz, 1H), 1.58 (s, 3H).
^{13}C NMR (75 MHz, CDCl$_3$) δ 181.6, 177.5, 159.6, 142.2, 128.9, 127.8, 127.2, 126.6, 124.9, 114.6, 55.6, 52.0, 41.6, 39.5, 36.8, 21.0.
HRMS (ESI-TOF): m/z calcd. for $[M + Na]^+$ $C_{20}H_{19}NO_3Na^+$ 344.1257; Found: 344.1260.

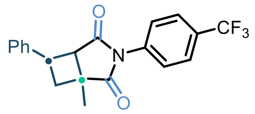

1-methyl-6-phenyl-3-(4-(trifluoromethyl)phenyl)-3-azabicyclo[3.2.0]heptane-2,4-dione (7c)
13.3 mg, 37% yield, white solid. Eluent: pentane/ethyl acetate = 20:1, R_f = 0.3, r.r.> 19:1, d.r. > 20:1.
^{1}H NMR (300 MHz, CDCl$_3$) δ 7.82 – 7.73 (m, 2H), 7.61 – 7.53 (m, 2H), 7.44 – 7.37 (m, 2H), 7.35 – 7.29 (m, 3H), 3.71 (td, *J* = 8.8, 5.5 Hz, 1H), 3.28 (dd, *J* = 5.6, 0.8 Hz, 1H), 2.98 (dddd, *J* = 12.8, 9.3, 3.5, 0.9 Hz, 1H), 2.53 (dd, *J* = 12.8, 8.3 Hz, 1H), 1.58 (s, 3H).
^{13}C NMR (75 MHz, CDCl$_3$) δ 180.9, 176.7, 141.9, 135.4, 130.8, 130.4, 129.0, 127.3, 126.8, 126.5, 126.4 (q, *J* = 3.8 Hz), 52.0, 41.8, 39.5, 36.9, 21.0.
^{19}F NMR (282 MHz, CDCl$_3$) δ -62.68 (s).
HRMS (ESI-TOF): m/z calcd. for $[M + Na]^+$ $C_{20}H_{16}F_3NO_2Na^+$ 382.1025; Found: 382.1031.

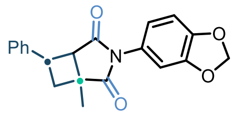

3-(benzo[d][1,3]dioxol-5-yl)-1-methyl-6-phenyl-3-azabicyclo[3.2.0]heptane-2,4-dione (7d)
16.2 mg, 48% yield, white solid. Eluent: pentane/ethyl acetate = 6:1, R_f = 0.2, r.r.> 19:1, d.r. > 20:1.

¹H NMR (300 MHz, CDCl₃) δ 7.46 – 7.36 (m, 2H), 7.34 – 7.27 (m, 3H), 6.98 – 6.88 (m, 1H), 6.85 – 6.77 (m, 2H), 6.03 (s, 2H), 3.66 (td, *J* = 8.8, 5.5 Hz, 1H), 3.23 (d, *J* = 5.5 Hz, 1H), 2.95 (dddd, *J* = 12.8, 9.3, 3.5, 0.9 Hz, 1H), 2.48 (dd, *J* = 12.8, 8.2 Hz, 1H), 1.55 (s, 3H).
^{13}C NMR (75 MHz, $CDCl_3$) δ 181.5, 177.3, 148.3, 148.0, 142.1, 129.0, 127.2, 126.6, 125.8, 120.5, 108.6, 107.8, 102.0, 51.9, 41.7, 39.5, 36.9, 21.0.
HRMS (ESI-TOF): m/z calcd. for $[M + Na]^+$ $C_{20}H_{17}NO_4Na^+$ 358.1049; Found: 358.1053.

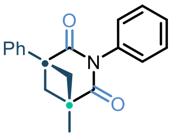

1-methyl-3,5-diphenyl-3-azabicyclo[3.1.1]heptane-2,4-dione (8a)
17.6 mg, 60% yield, white solid. Eluent: pentane/ethyl acetate = 10:1, R_f = 0.2, r.r.> 19:1.
^{1}H NMR (300 MHz, $CDCl_3$) δ 7.50 – 7.36 (m, 5H), 7.35 – 7.32 (m, 1H), 7.24 – 7.14 (m, 4H), 3.09 – 3.00 (m, 2H), 2.69 – 2.58 (m, 2H), 1.47 (s, 3H).
^{13}C NMR (75 MHz, $CDCl_3$) δ 177.4, 176.3, 139.2, 133.8, 129.2, 128.6, 128.5, 128.5, 127.6, 126.6, 50.7, 44.6, 43.1, 19.8.
HRMS (ESI-TOF): m/z calcd. for $[M + Na]^+$ $C_{19}H_{17}NO_2Na^+$ 314.1151; Found: 314.1151.

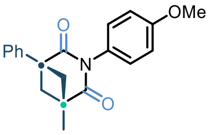

3-(4-methoxyphenyl)-1-methyl-5-phenyl-3-azabicyclo[3.1.1]heptane-2,4-dione (8b)
17.2 mg, 54% yield, white solid. Eluent: pentane/ethyl acetate = 5:1, R_f = 0.2, r.r.> 19:1.
^{1}H NMR (300 MHz, $CDCl_3$) δ 7.43 – 7.36 (m, 2H), 7.35 – 7.28 (m, 1H), 7.19 – 7.08 (m, 4H), 7.00 – 6.91 (m, 2H), 3.82 (s, 3H), 3.07 – 2.98 (m, 2H), 2.67 – 2.56 (m, 2H), 1.46 (s, 3H).
^{13}C NMR (75 MHz, $CDCl_3$) δ 177.7, 176.5, 159.5, 139.3, 129.5, 128.6, 127.6, 126.6, 126.4, 114.5, 55.6, 50.7, 44.6, 43.1, 19.8.
HRMS (ESI-TOF): m/z calcd. for $[M + Na]^+$ $C_{20}H_{19}NO_3Na^+$ 344.1257; Found: 344.1261.

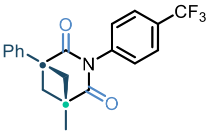

1-methyl-5-phenyl-3-(4-(trifluoromethyl)phenyl)-3-azabicyclo[3.1.1]heptane-2,4-dione (8c)
15.7 mg, 44% yield, white solid. Eluent: pentane/ethyl acetate = 15:1, R_f = 0.2, r.r.> 19:1.
^{1}H NMR (300 MHz, $CDCl_3$) δ 7.72 (d, *J* = 8.1 Hz, 2H), 7.46 – 7.29 (m, 5H), 7.20 – 7.12 (m, 2H), 3.13 – 3.00 (m, 2H), 2.75 – 2.60 (m, 2H), 1.48 (s, 3H).
^{13}C NMR (75 MHz, $CDCl_3$) δ 177.0, 175.9, 138.9, 137.0, 130.9, 130.5, 129.2, 128.7, 127.9, 126.6, 126.2 (q, *J* = 3.7 Hz), 50.8, 44.5, 43.3, 19.7.
^{19}F NMR (282 MHz, $CDCl_3$) δ -62.62 (s).
HRMS (ESI-TOF): m/z calcd. for $[M + Na]^+$ $C_{20}H_{16}F_3NO_2Na^+$ 382.1025; Found: 382.1032.

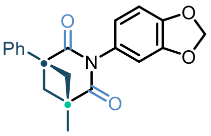

3-(benzo[d][1,3]dioxol-5-yl)-1-methyl-5-phenyl-3-azabicyclo[3.1.1]heptane-2,4-dione (8d)
19.7 mg, 59% yield, white solid. Eluent: pentane/ethyl acetate = 7:1, R_f = 0.2, r.r.> 19:1.
^{1}H NMR (300 MHz, $CDCl_3$) δ 7.44 – 7.36 (m, 2H), 7.34 – 7.28 (m, 1H), 7.20 – 7.11 (m, 2H), 6.94 – 6.81 (m, 1H), 6.72 – 6.55 (m, 2H), 6.00 (s, 2H), 3.07 – 2.95 (m, 2H), 2.67 – 2.55 (m, 2H), 1.46 (s, 3H).
^{13}C NMR (75 MHz, $CDCl_3$) δ 177.6, 176.4, 148.3, 147.9, 139.2, 128.6, 127.7, 127.3, 126.6, 122.0, 109.5, 108.4, 101.9, 50.7, 44.6, 43.1, 19.8.
HRMS (ESI-TOF): m/z calcd. for $[M + Na]^+$ $C_{20}H_{17}NO_4Na^+$ 358.1049; Found: 358.1054.

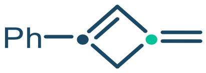

(3-methylenecyclobut-1-en-1-yl)benzene (9)

752.6 mg, 53% yield, colorless oil. Eluent: pentane, R_f = 0.3.

^{1}H NMR (300 MHz, CDCl$_3$) δ 7.47 – 7.42 (m, 2H), 7.38 – 7.28 (m, 3H), 6.59 (s, 1H), 4.81 (t, *J* = 1.4 Hz, 1H), 4.61 (d, *J* = 0.9 Hz, 1H), 3.21 (s, 2H).

^{13}C NMR (75 MHz, CDCl$_3$) δ 150.8, 144.7, 133.8, 128.6, 128.6, 128.5, 125.7, 98.9, 36.4.

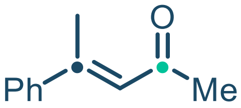

4-phenylpent-3-en-2-one (10)

13.5 mg, 85% yield, colorless oil. Eluent: pentane/ethyl acetate = 20:1, R_f = 0.2.

^{1}H NMR (300 MHz, CDCl$_3$) δ 7.51 – 7.45 (m, 2H), 7.40 – 7.34 (m, 3H), 6.52 – 6.50 (m, 1H), 2.54 (d, *J* = 1.3 Hz, 3H), 2.29 (s, 3H).

^{13}C NMR (75 MHz, CDCl$_3$) δ 199.0, 153.9, 142.6, 129.2, 128.6, 126.5, 124.6, 32.3, 18.4.

8. NMR Spectra of the Products

^{1}H NMR spectrum of **3a** (300 MHz, $CDCl_3$)

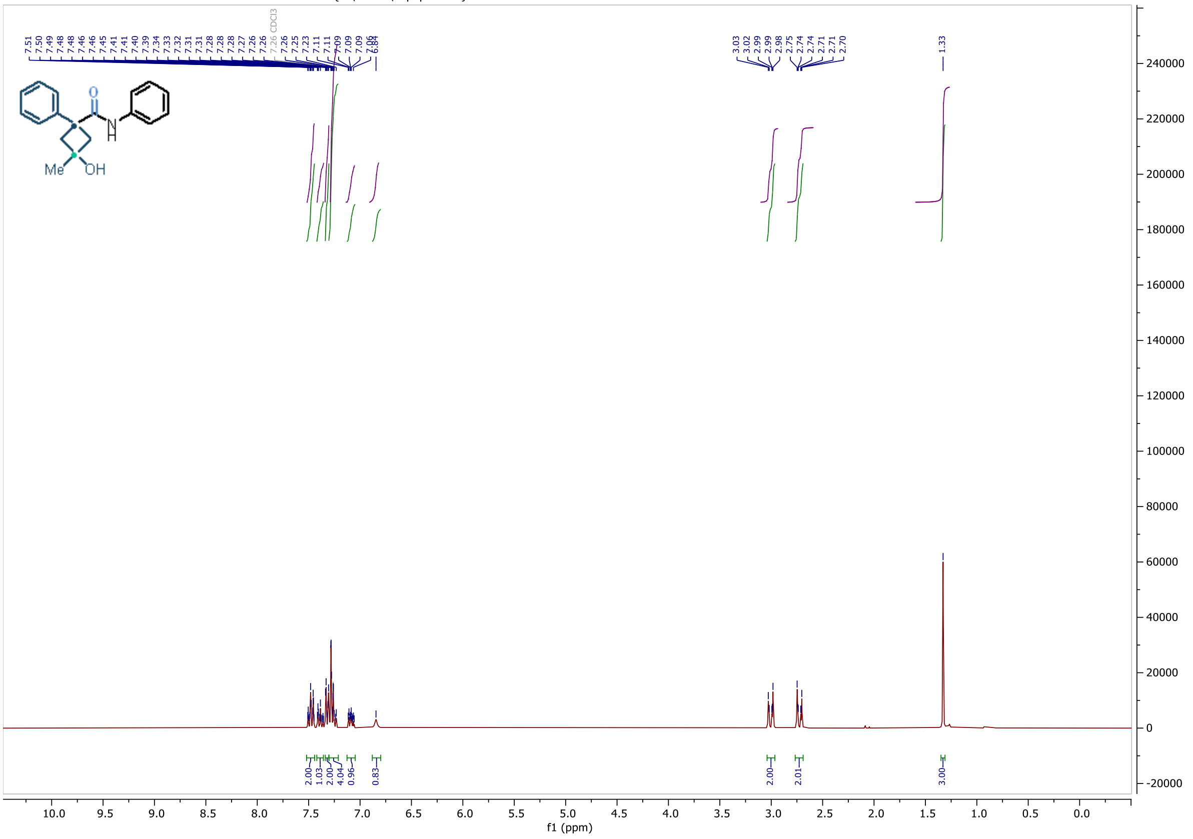

^{13}C NMR spectrum of **3a** (75 MHz, $CDCl_3$)

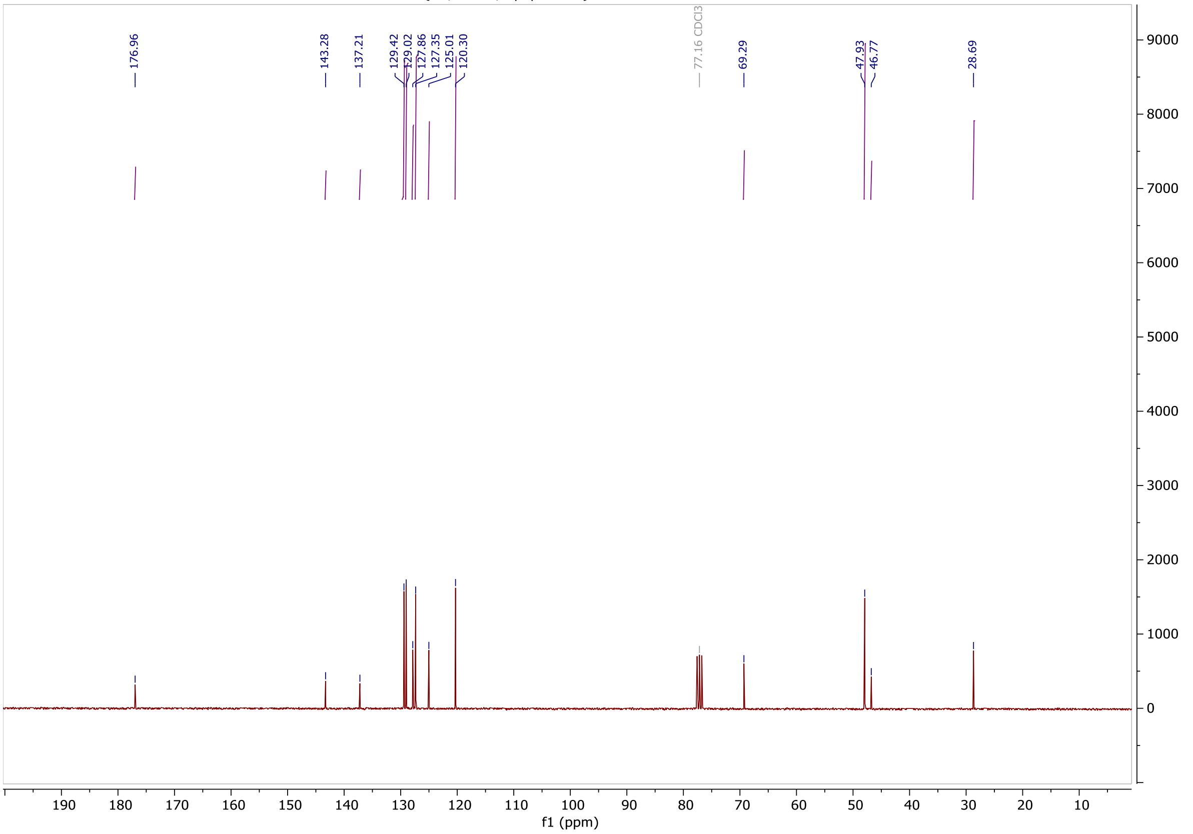

^{1}H NMR spectrum of **3b** (300 MHz, $CDCl_3$)

260317.322.10.fid — Yu-kun Liu LYK-24-102-re — Au1H CDCl3 {C:\Bruker\TopSpin3.6.2} 2603 22

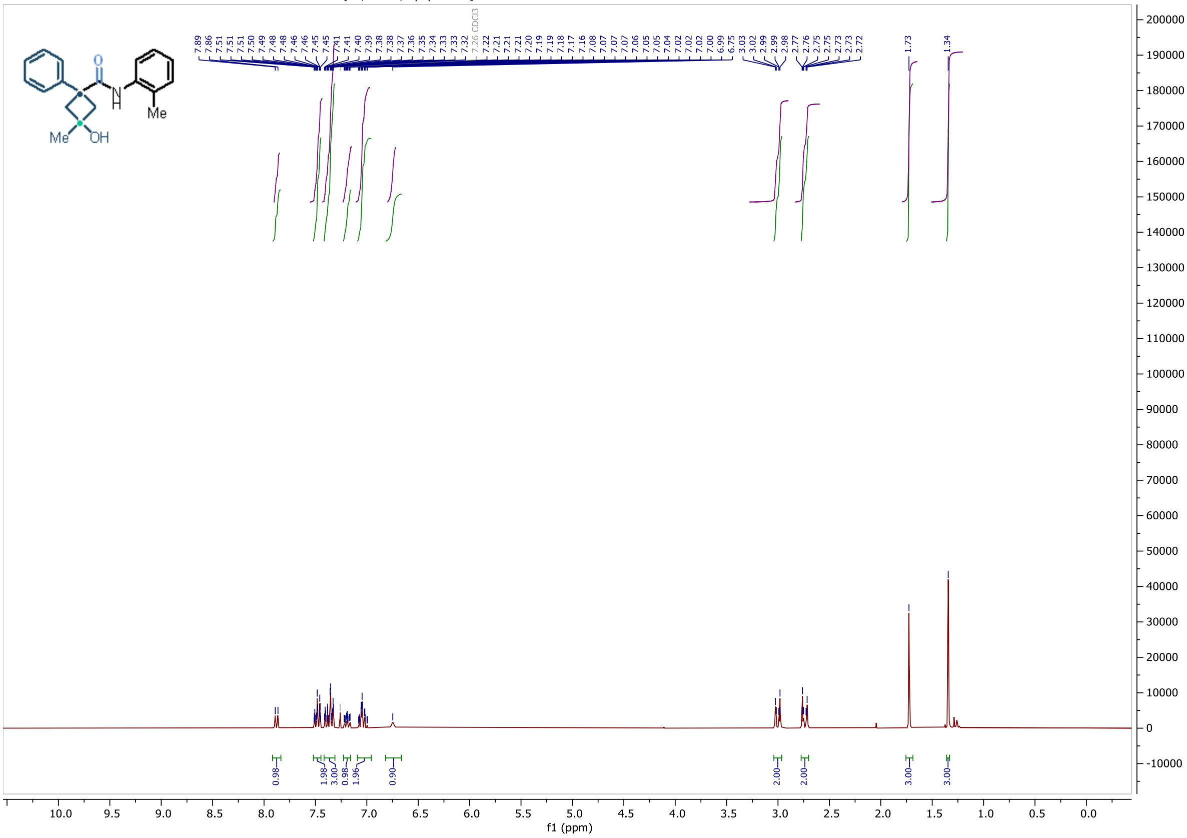

^{13}C NMR spectrum of **3b** (75 MHz, $CDCl_3$)

260317.322.11.fid — Yu-kun Liu LYK-24-102-re — Au13C CDCl3 {C:\Bruker\TopSpin3.6.2} 2603 22

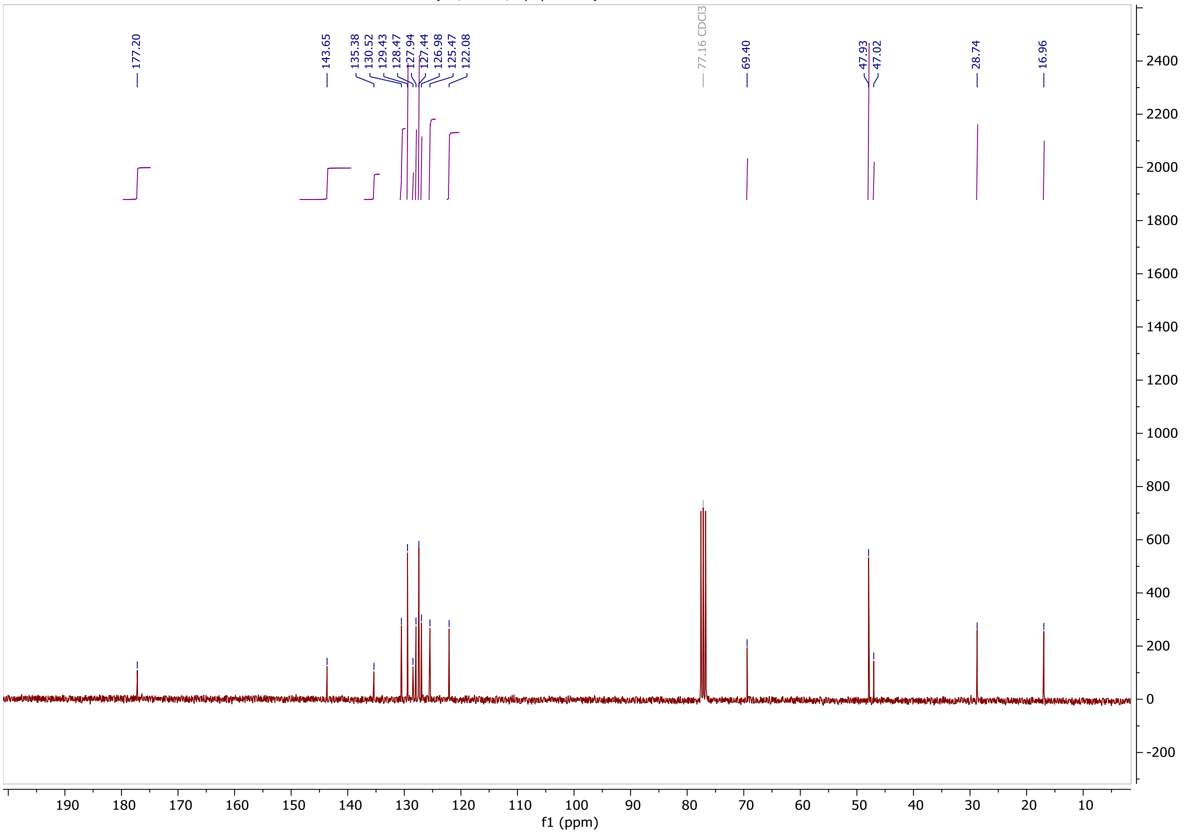

^{1}H NMR spectrum of **3c** (300 MHz, $CDCl_3$)

260317.321.10.fid — Yu-kun Liu LYK-24-101-re — Au1H CDCl3 {C:\Bruker\TopSpin3.6.2} 2603 21

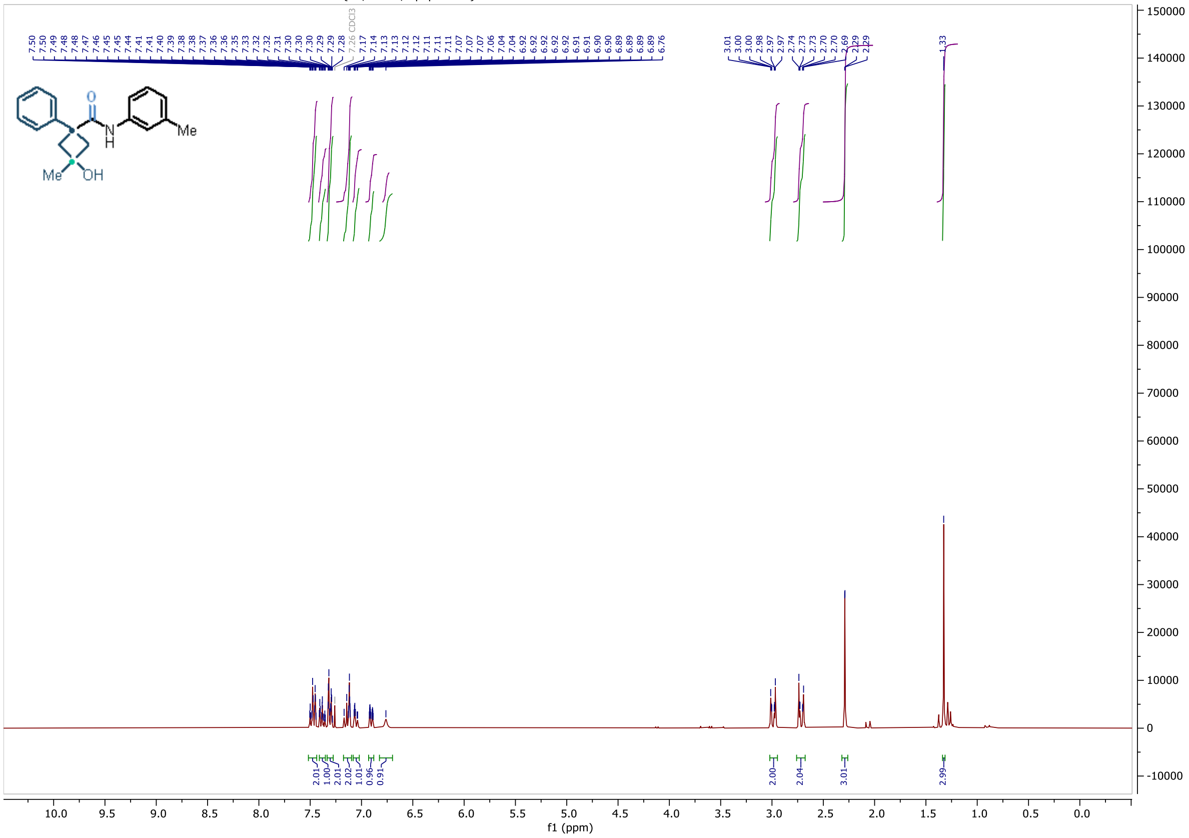

^{13}C NMR spectrum of **3c** (75 MHz, $CDCl_3$)

260317.321.11.fid — Yu-kun Liu LYK-24-101-re — Au13C CDCl3 {C:\Bruker\TopSpin3.6.2} 2603 21

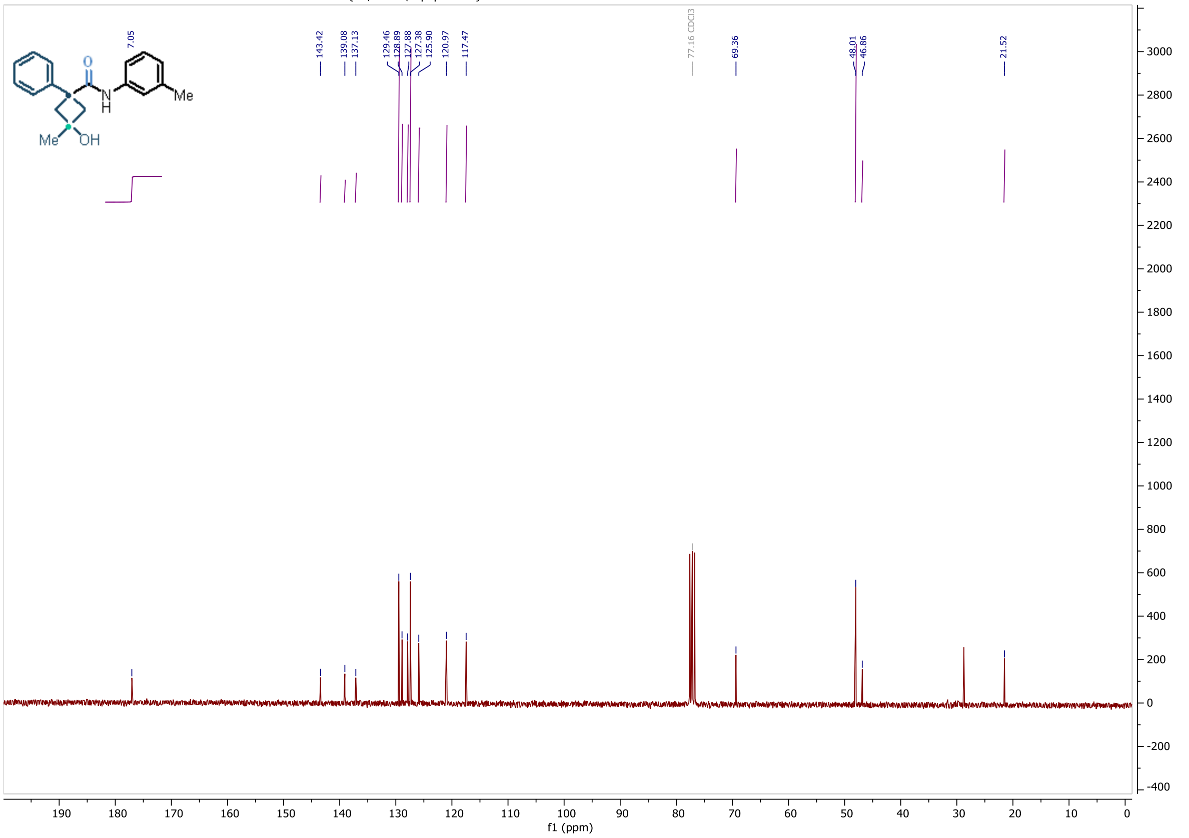

^{1}H NMR spectrum of **3d** (300 MHz, $CDCl_3$)

260320.303.10.fid — Yu-kun Liu LYK-24-123 — Au1H CDCl3 {C:\Bruker\TopSpin3.6.2} 2603 3

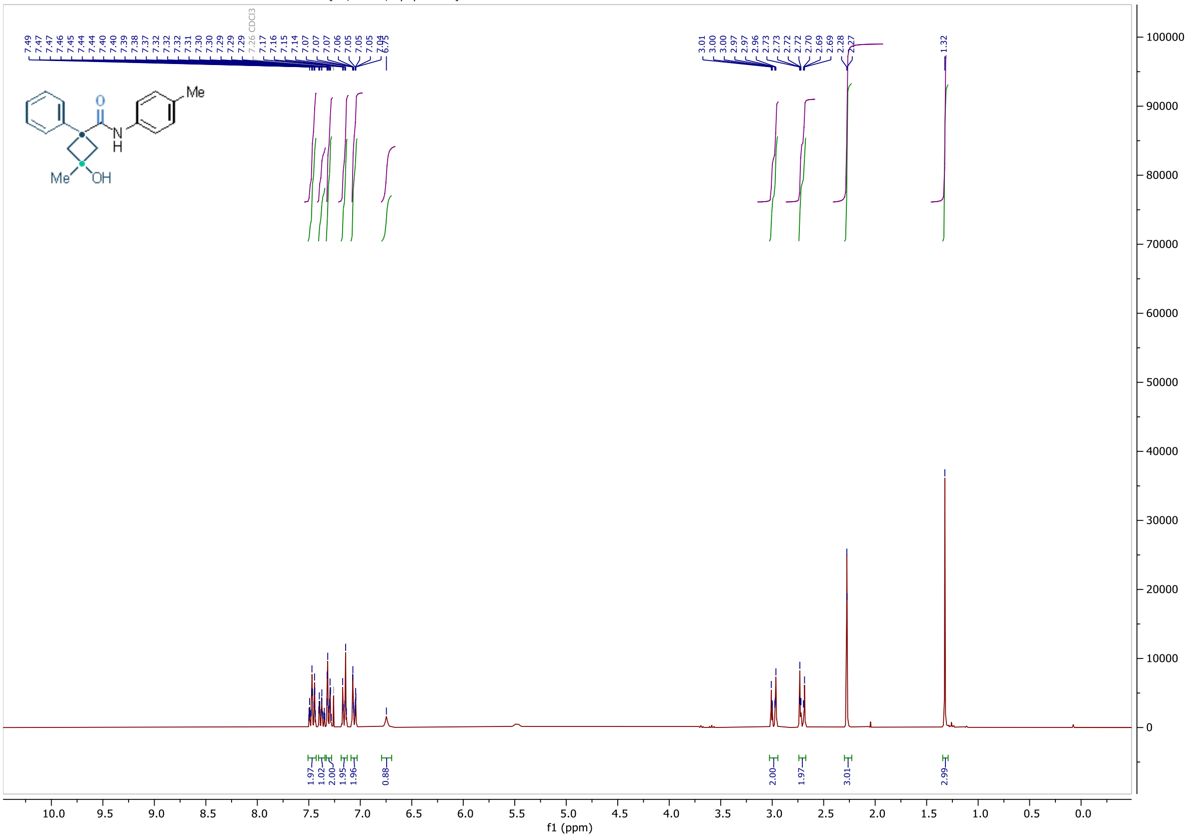

^{13}C NMR spectrum of **3d** (75 MHz, $CDCl_3$)

260320.303.11.fid — Yu-kun Liu LYK-24-123 — Au13C CDCl3 {C:\Bruker\TopSpin3.6.2} 2603 3

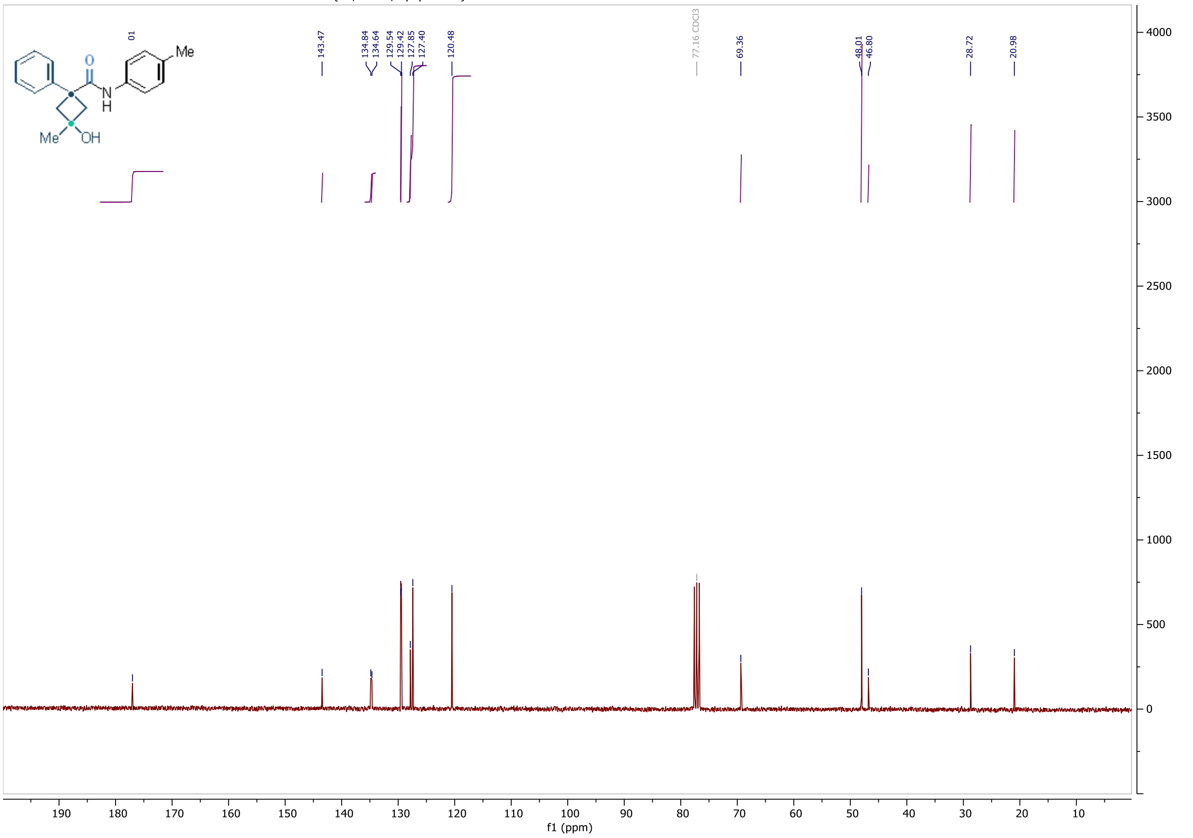

^{1}H NMR spectrum of **3e** (300 MHz, $CDCl_3$)

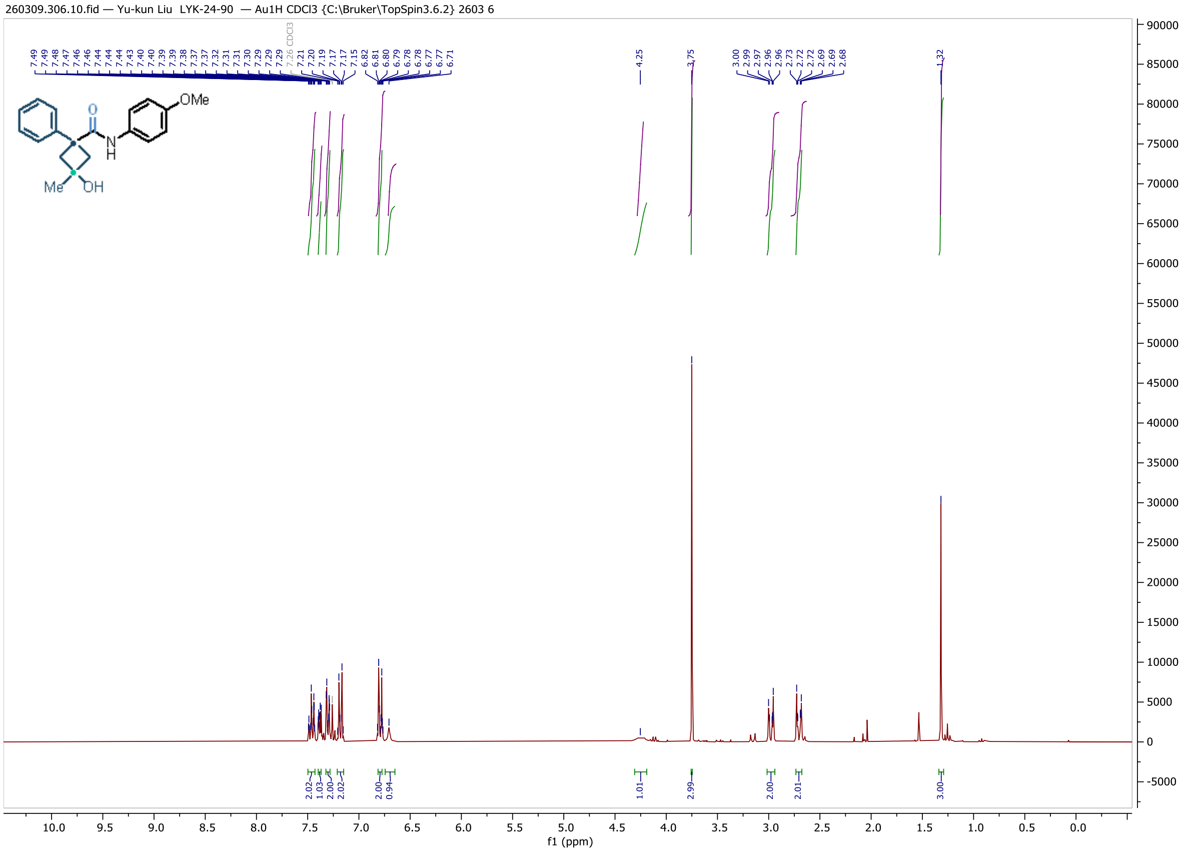

^{13}C NMR spectrum of **3e** (75 MHz, $CDCl_3$)

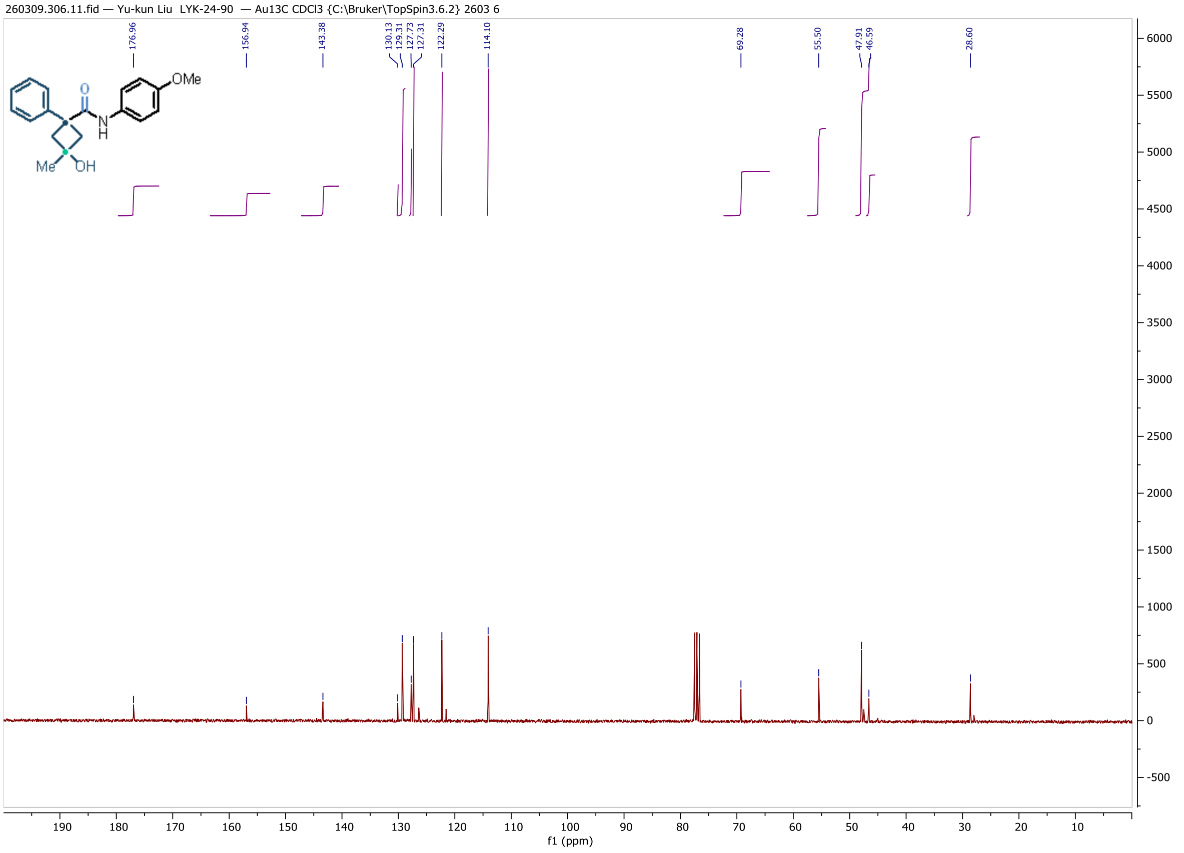

^{1}H NMR spectrum of **3f** (300 MHz, $CDCl_3$)

260319.312.10.fid — Yu-kun Liu LYK-24-88-re — Au1H CDCl3 {C:\Bruker\TopSpin3.6.2} 2603 12

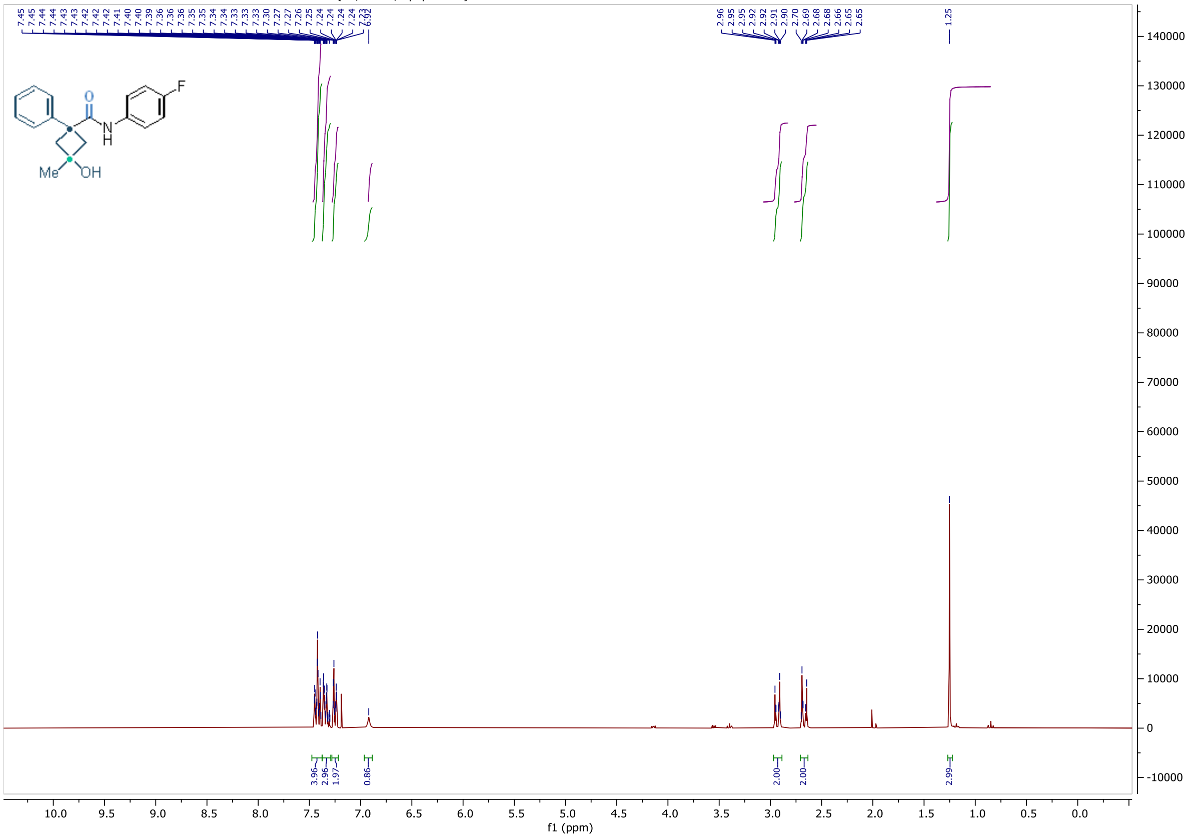

^{13}C NMR spectrum of **3f** (75 MHz, $CDCl_3$)

260319.312.11.fid — Yu-kun Liu LYK-24-88-re — Au13C CDCl3 {C:\Bruker\TopSpin3.6.2} 2603 12

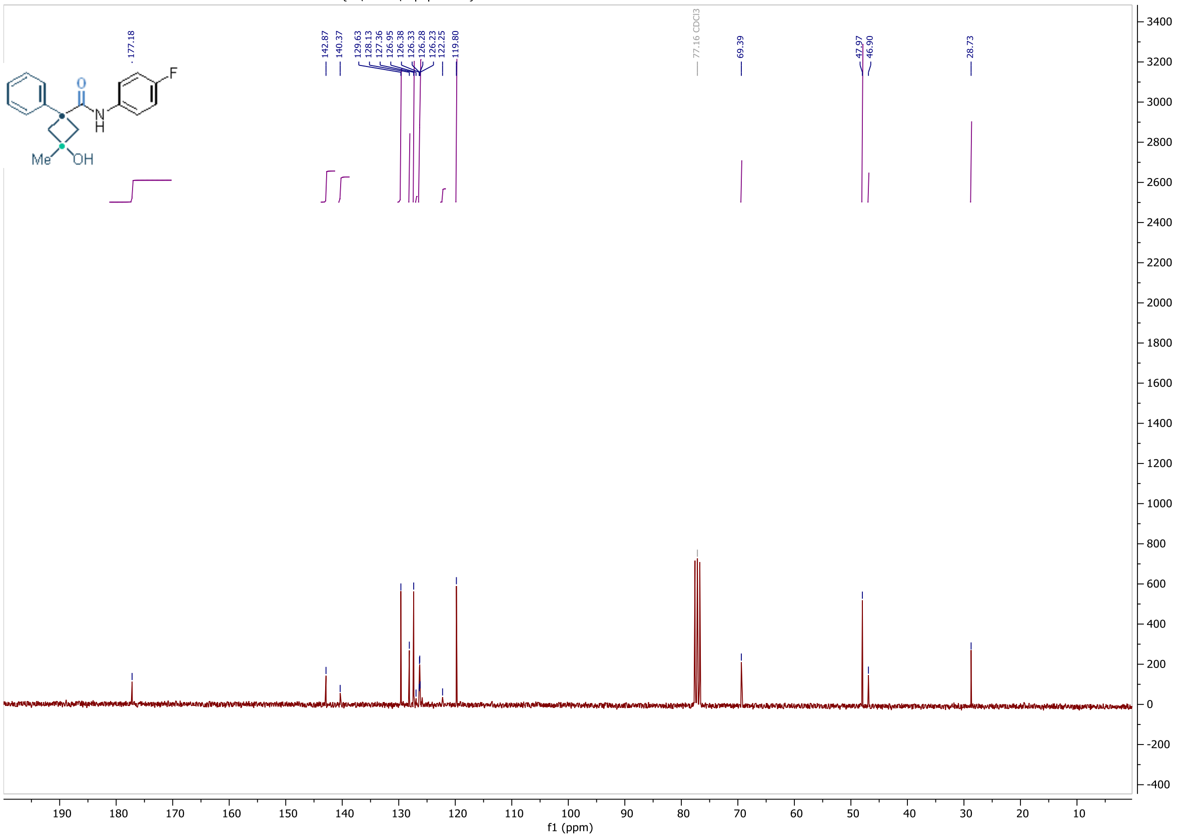

^{19}F NMR spectrum of **3f** (282 MHz, $CDCl_3$)

260319.312.12.fid — Yu-kun Liu LYK-24-88-re — Au19F CDCl3 {C:\Bruker\TopSpin3.6.2} 2603 12

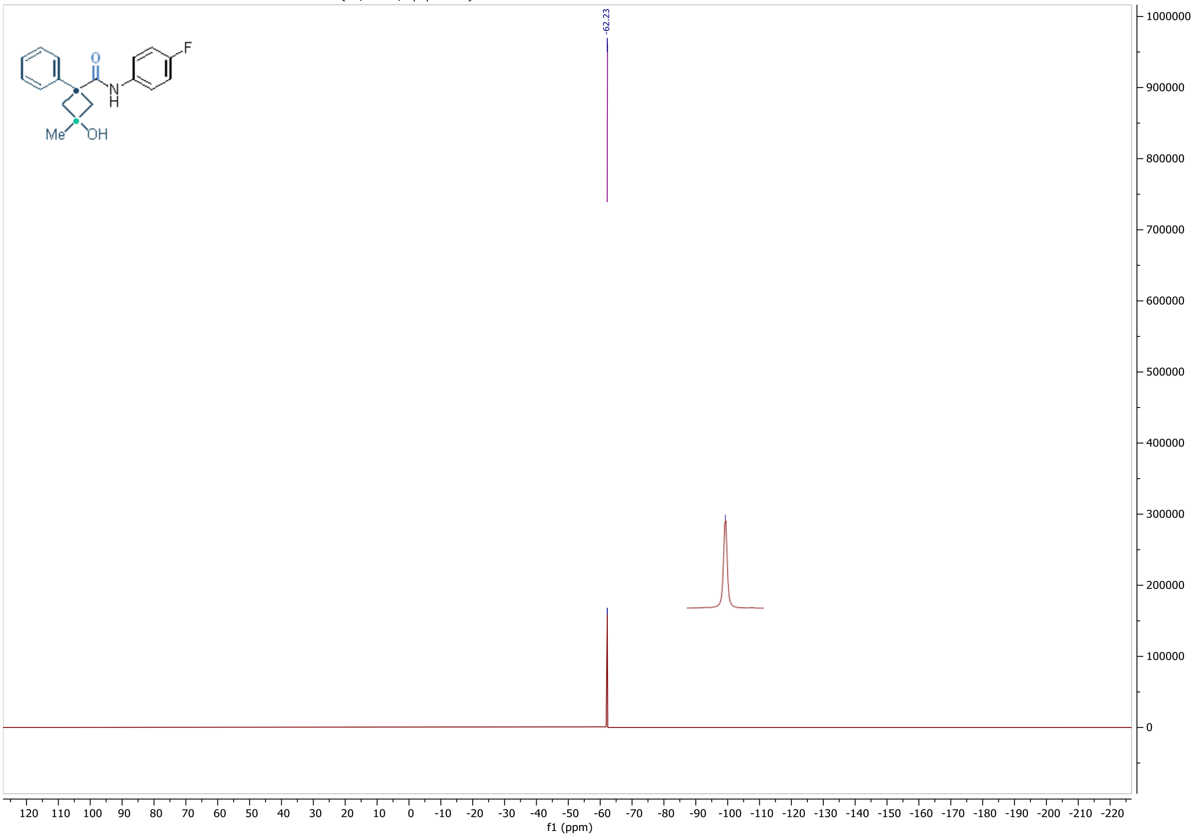

^{1}H NMR spectrum of **3g** (400 MHz, $CDCl_3$)

260310.423.10.fid — Yu-kun Liu LYK-24-93 — Au1H CDCl3 {C:\Bruker\TopSpin3.6.2} 2603 23

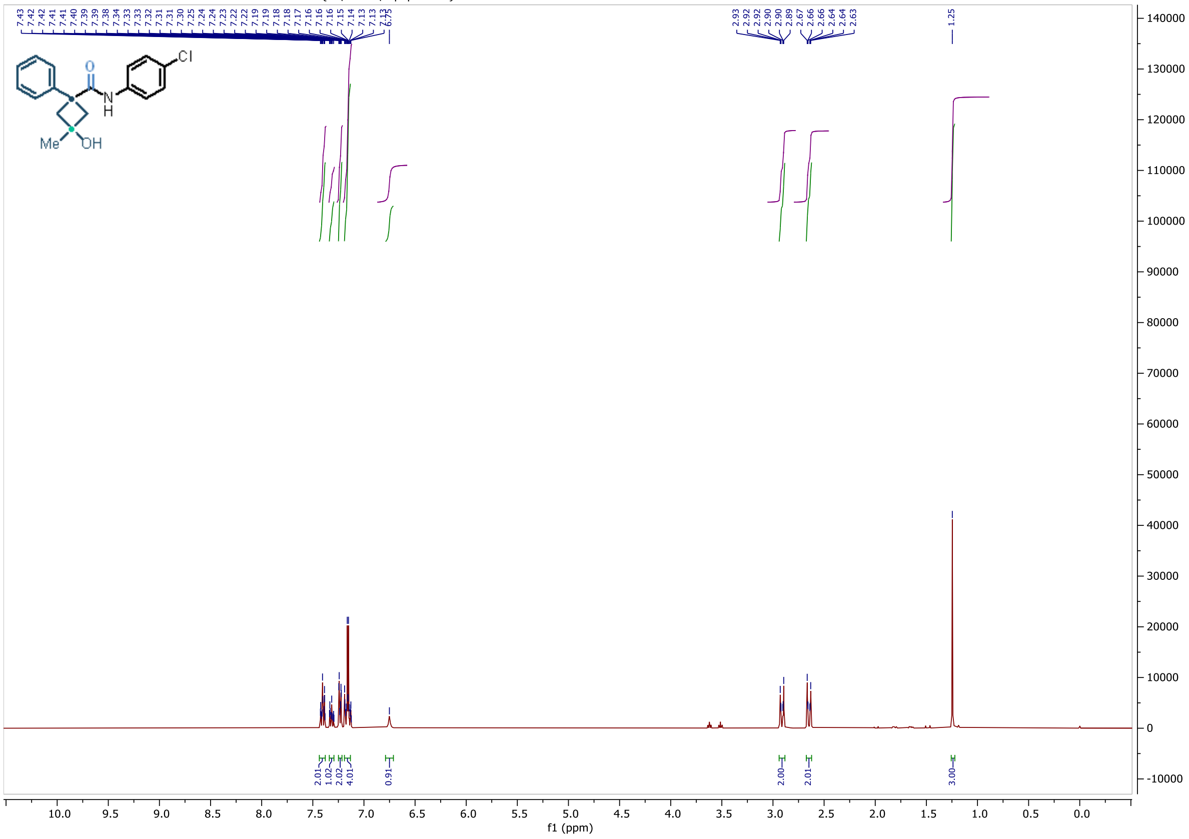

^{13}C NMR spectrum of **3g** (101 MHz, $CDCl_3$)

260310.423.11.fid — Yu-kun Liu LYK-24-93 — Au13C CDCl3 {C:\Bruker\TopSpin3.6.2} 2603 23

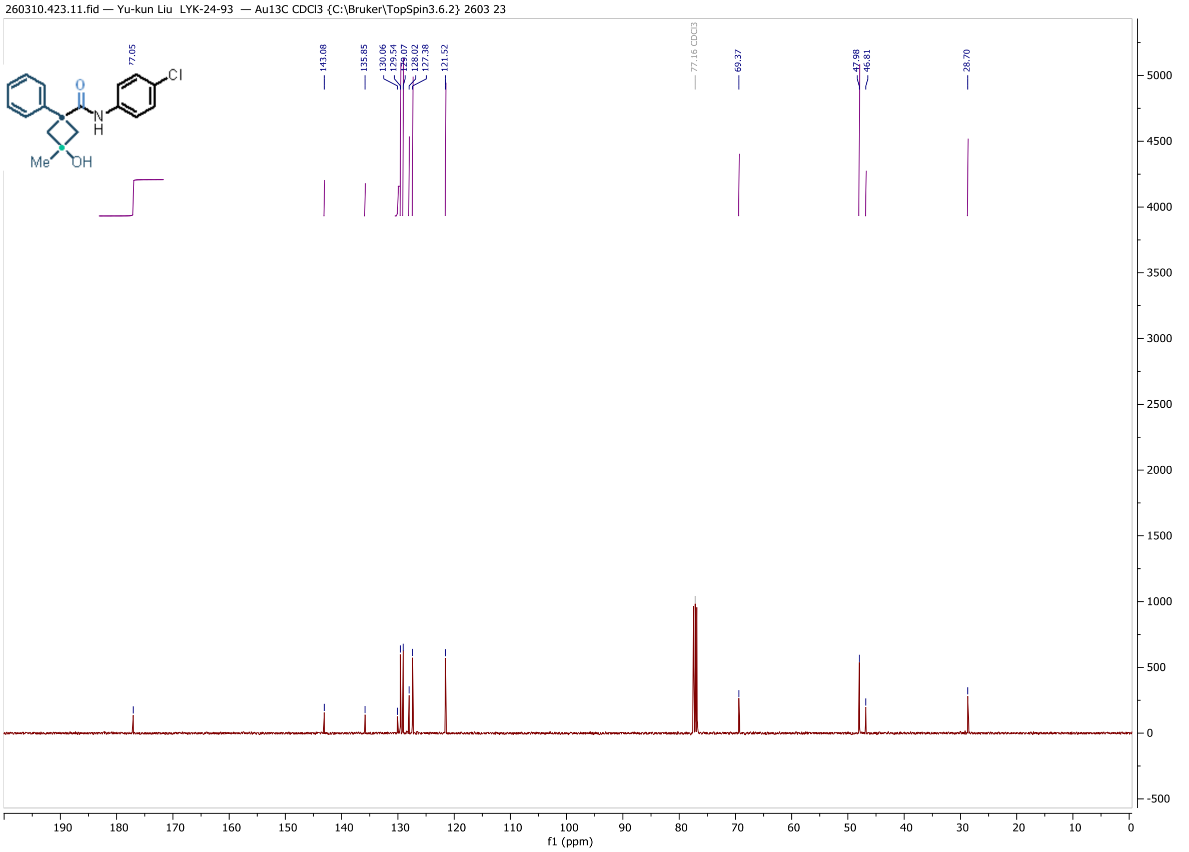

^{1}H NMR spectrum of **3h** (400 MHz, $CDCl_3$)

260310.424.10.fid — Yu-kun Liu LYK-24-94 — Au1H CDCl3 {C:\Bruker\TopSpin3.6.2} 2603 24

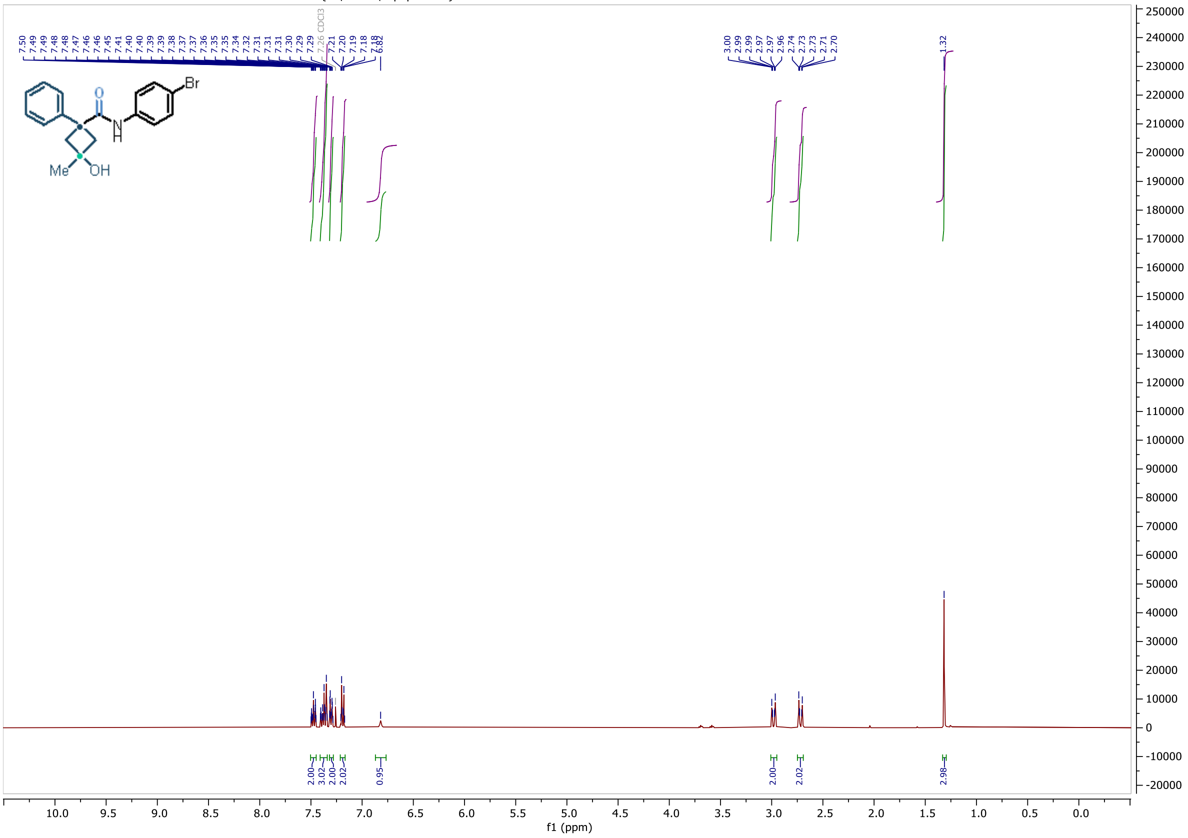

^{13}C NMR spectrum of **3h** (101 MHz, $CDCl_3$)

260310.424.11.fid — Yu-kun Liu LYK-24-94 — Au13C CDCl3 {C:\Bruker\TopSpin3.6.2} 2603 24

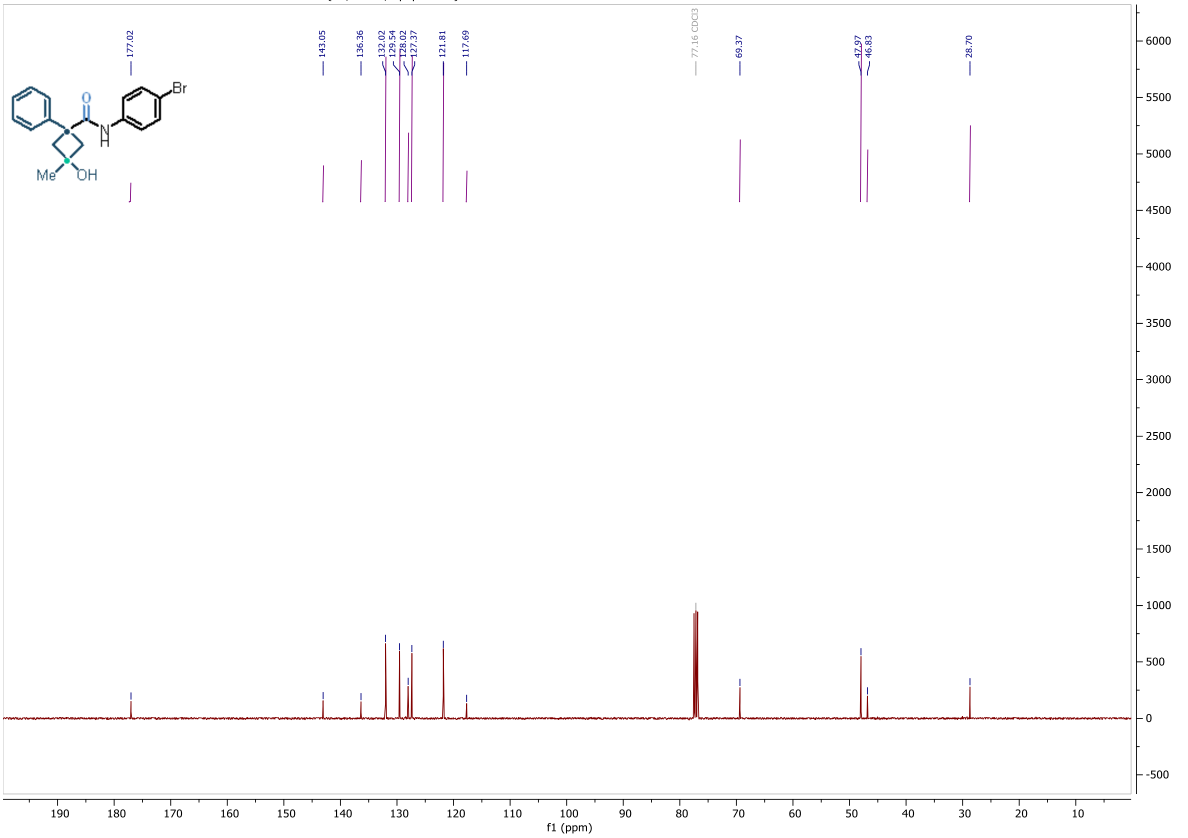

^{1}H NMR spectrum of **3i** (300 MHz, $CDCl_3$)

260320.301.10.fid — Yu-kun Liu LYK-24-99-re-3 — Au1H CDCl3 {C:\Bruker\TopSpin3.6.2} 2603 1

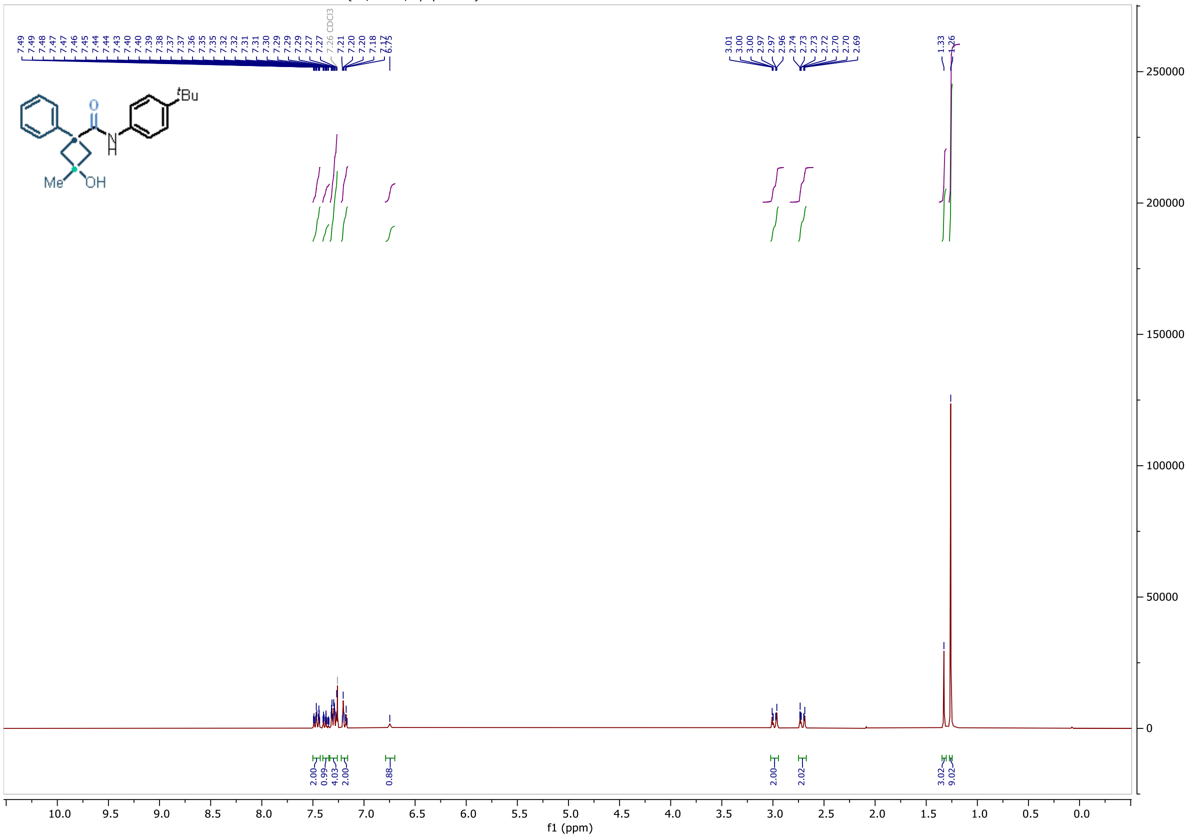

^{13}C NMR spectrum of **3i** (75 MHz, $CDCl_3$)

260320.301.11.fid — Yu-kun Liu LYK-24-99-re-3 — Au13C CDCl3 {C:\Bruker\TopSpin3.6.2} 2603 1

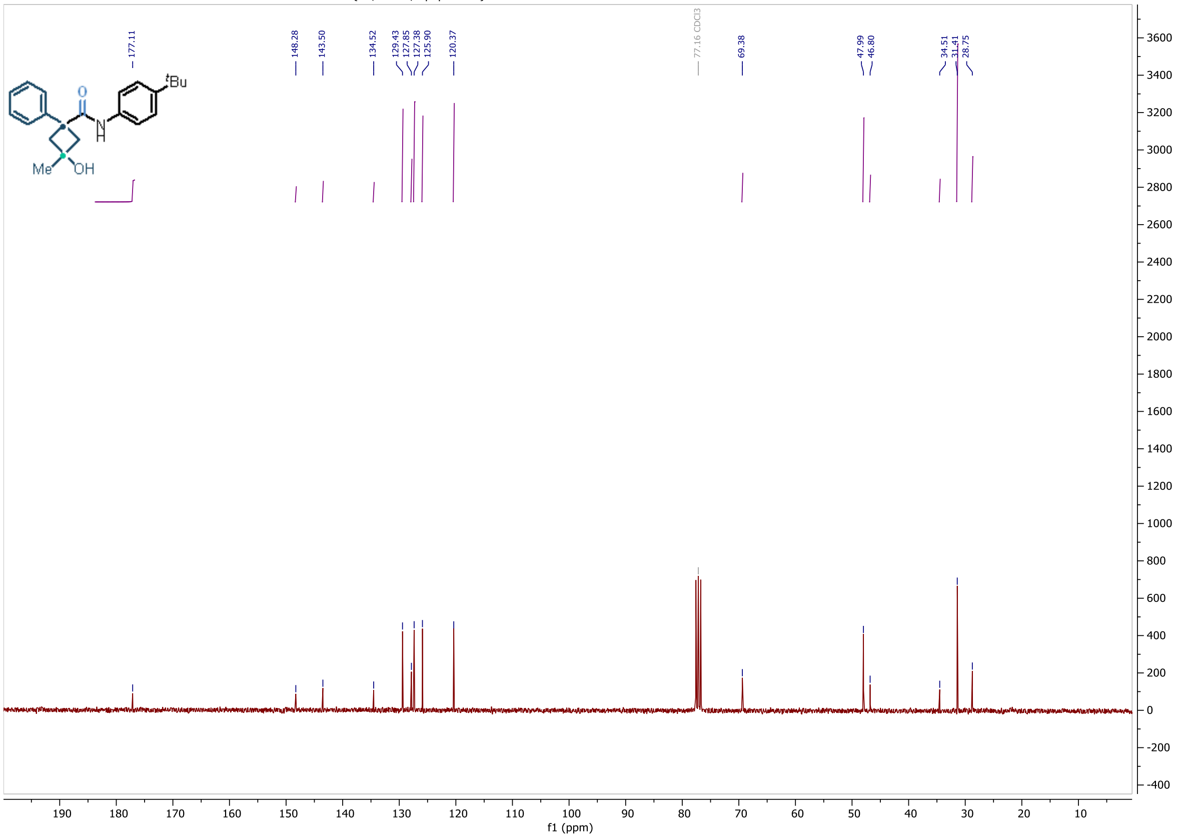

^{1}H NMR spectrum of **3j** (300 MHz, $CDCl_3$)

260319.337.10.fid — Yu-ku Liu, LYK-24-106-re-2 — Au1H CDCl3 {C:\Bruker\TopSpin3.6.2} 2603 37

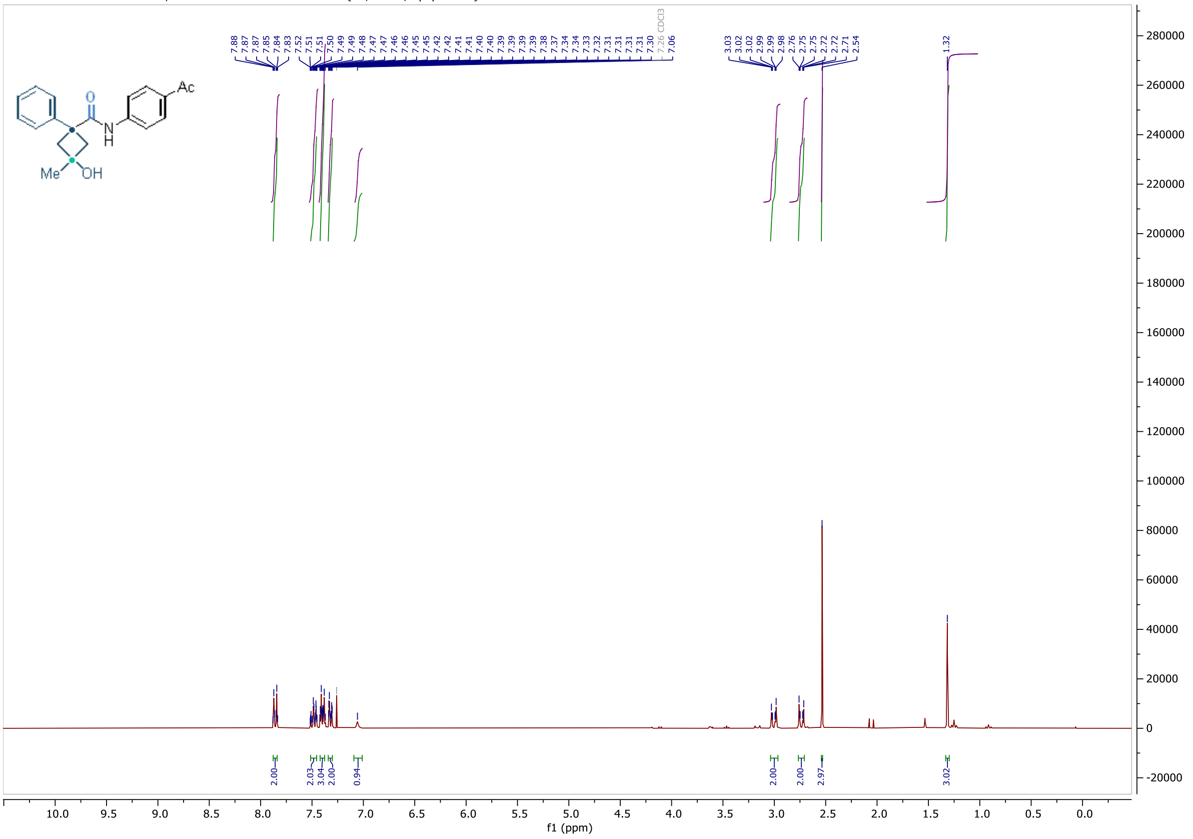

^{13}C NMR spectrum of **3j** (75 MHz, $CDCl_3$)

260319.337.11.fid — Yu-ku Liu, LYK-24-106-re-2 — Au13C CDCl3 {C:\Bruker\TopSpin3.6.2} 2603 37

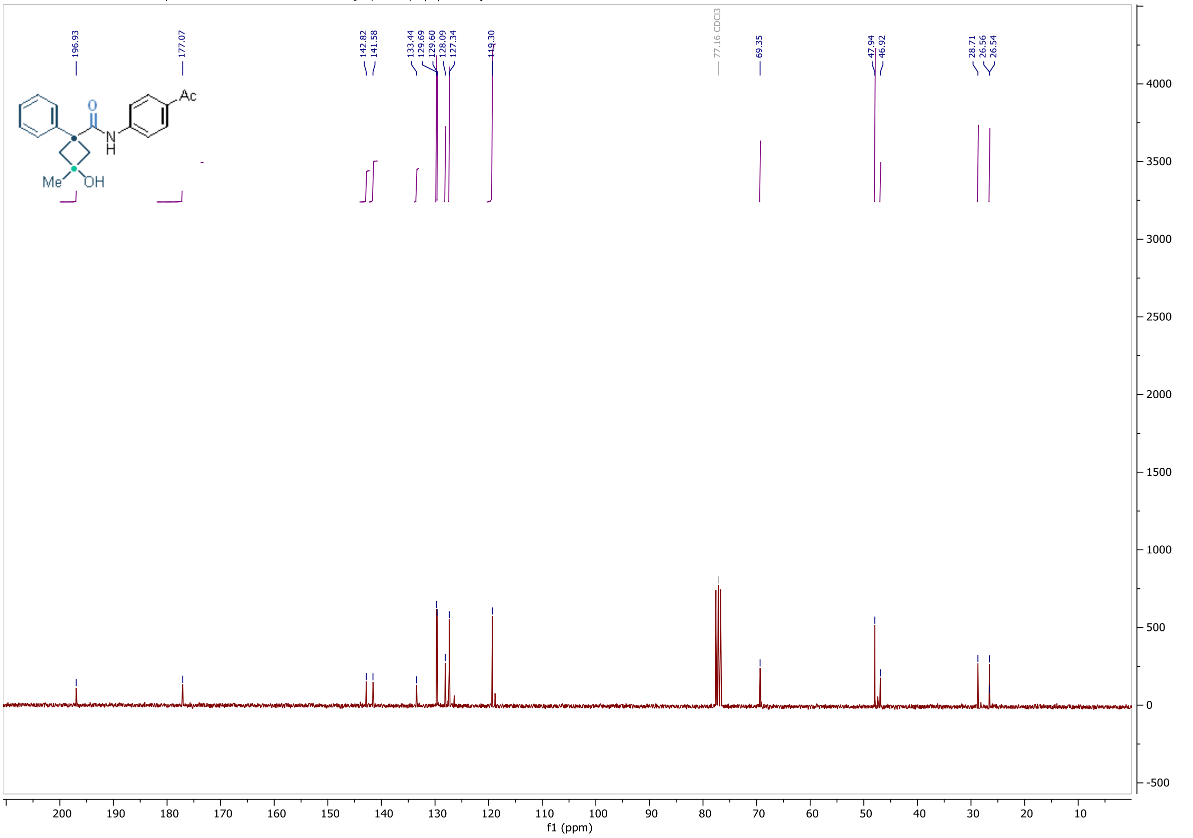

^{1}H NMR spectrum of **3k** (400 MHz, $CDCl_3$)

260310.422.10.fid — Yu-kun Liu LYK-24-92 — Au1H CDCl3 {C:\Bruker\TopSpin3.6.2} 2603 22

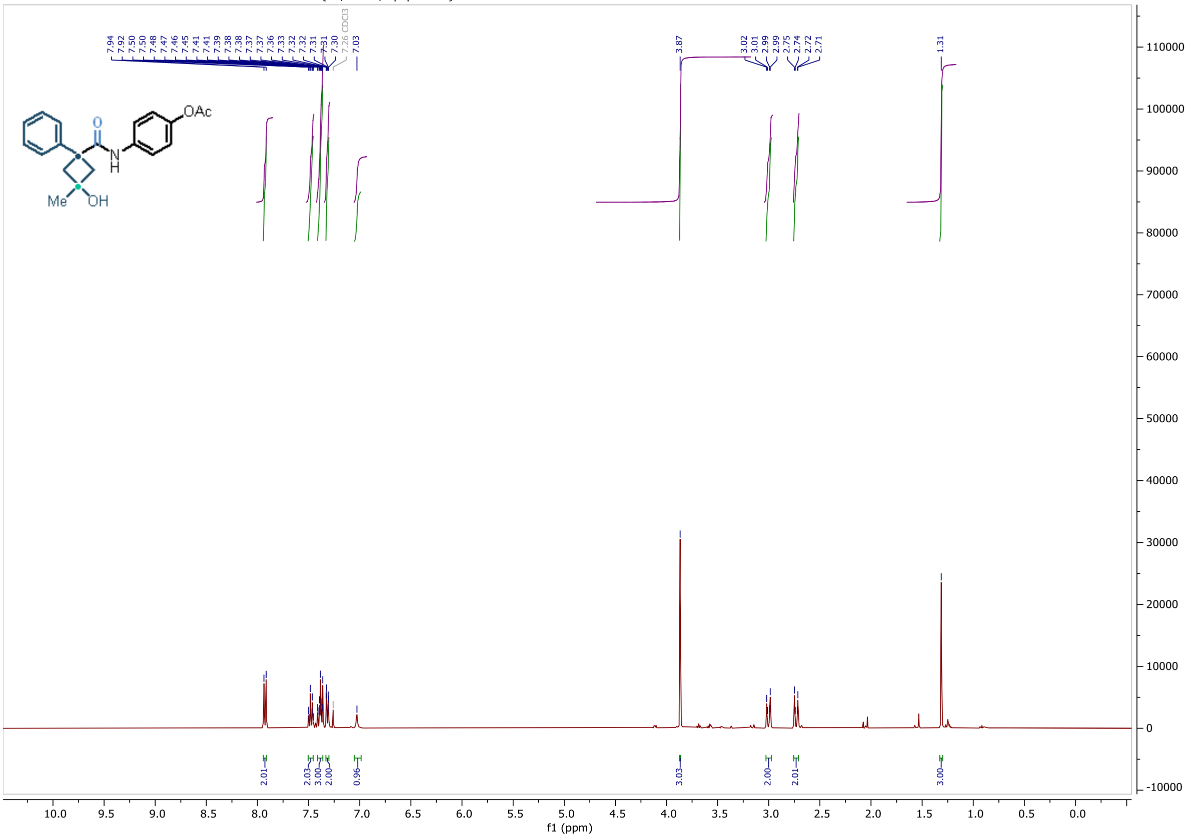

^{13}C NMR spectrum of **3k** (101 MHz, $CDCl_3$)

260310.422.11.fid — Yu-kun Liu LYK-24-92 — Au13C CDCl3 {C:\Bruker\TopSpin3.6.2} 2603 22

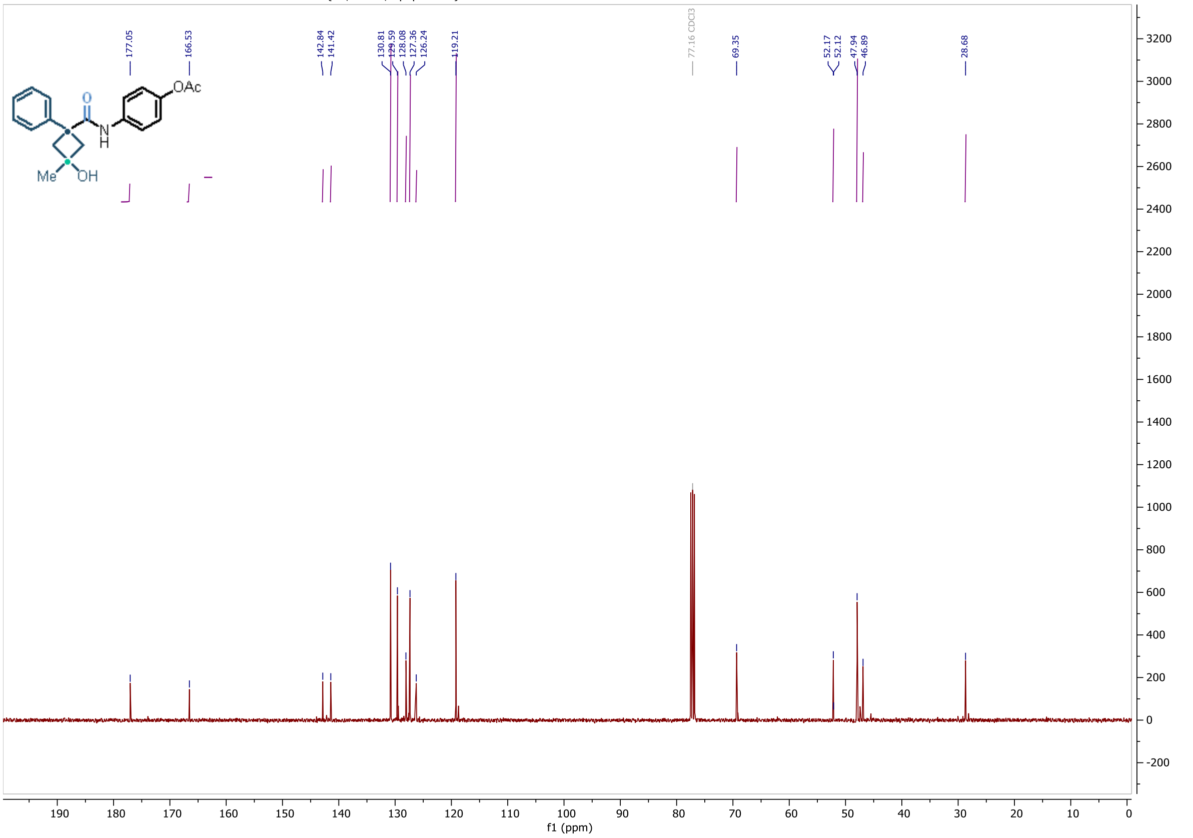

^{1}H NMR spectrum of **3l** (300 MHz, $CDCl_3$)

260319.313.10.fid — Yu-kun Liu LYK-24-89-re — Au1H CDCl3 {C:\Bruker\TopSpin3.6.2} 2603 13

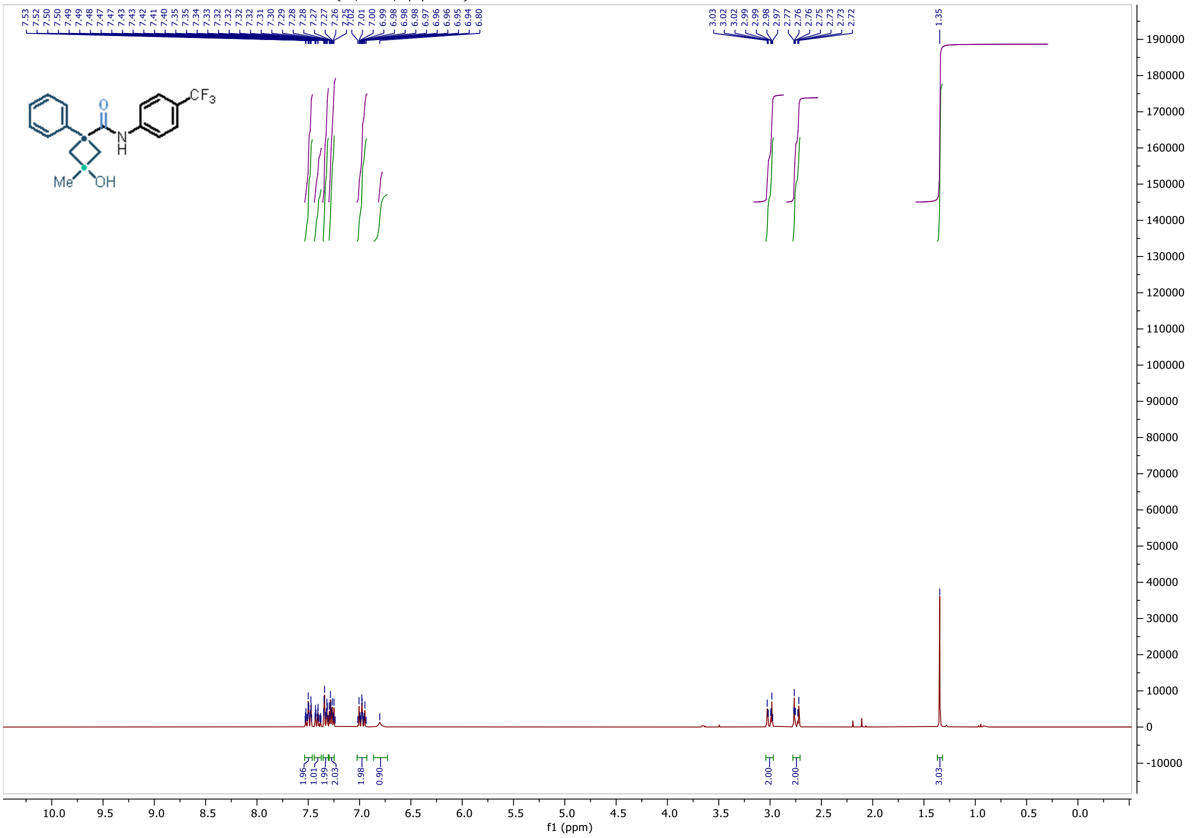

^{13}C NMR spectrum of **3l** (75 MHz, $CDCl_3$)

260319.313.11.fid — Yu-kun Liu LYK-24-89-re — Au13C CDCl3 {C:\Bruker\TopSpin3.6.2} 2603 13

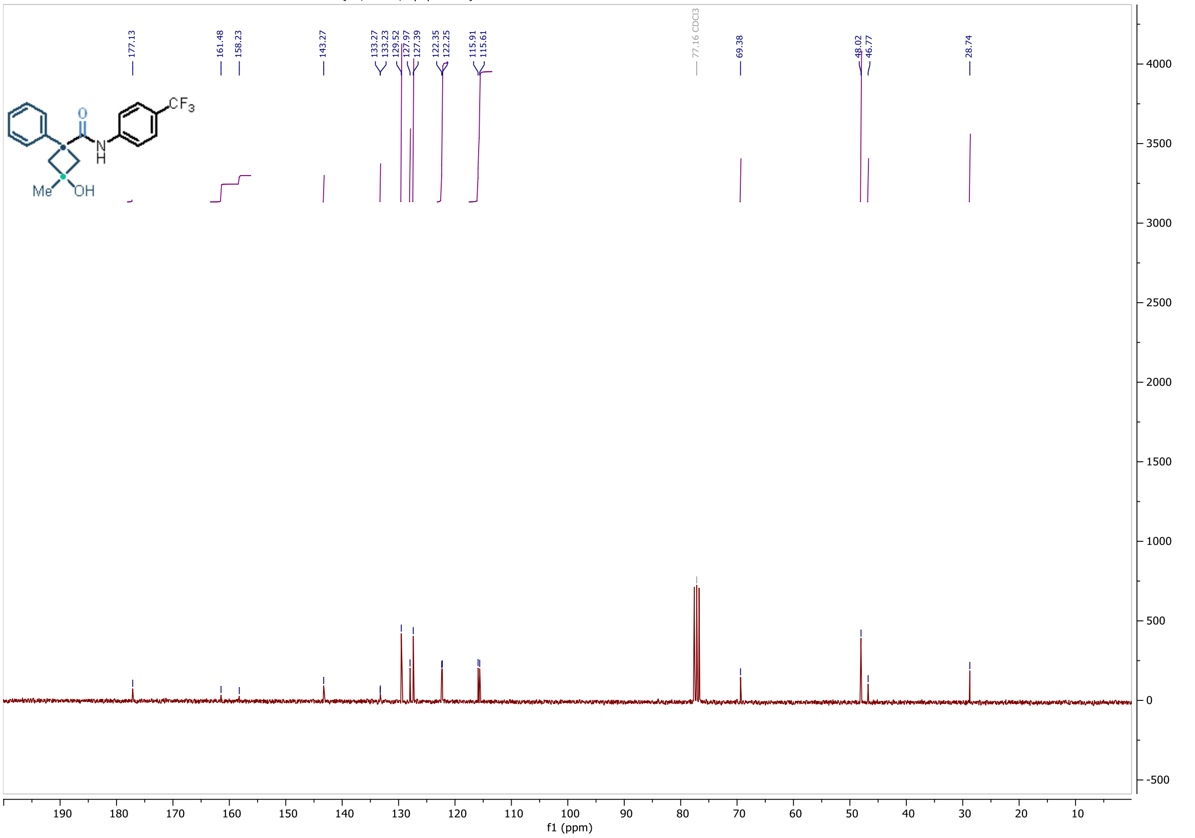

^{19}F NMR spectrum of **3l** (282 MHz, $CDCl_3$)

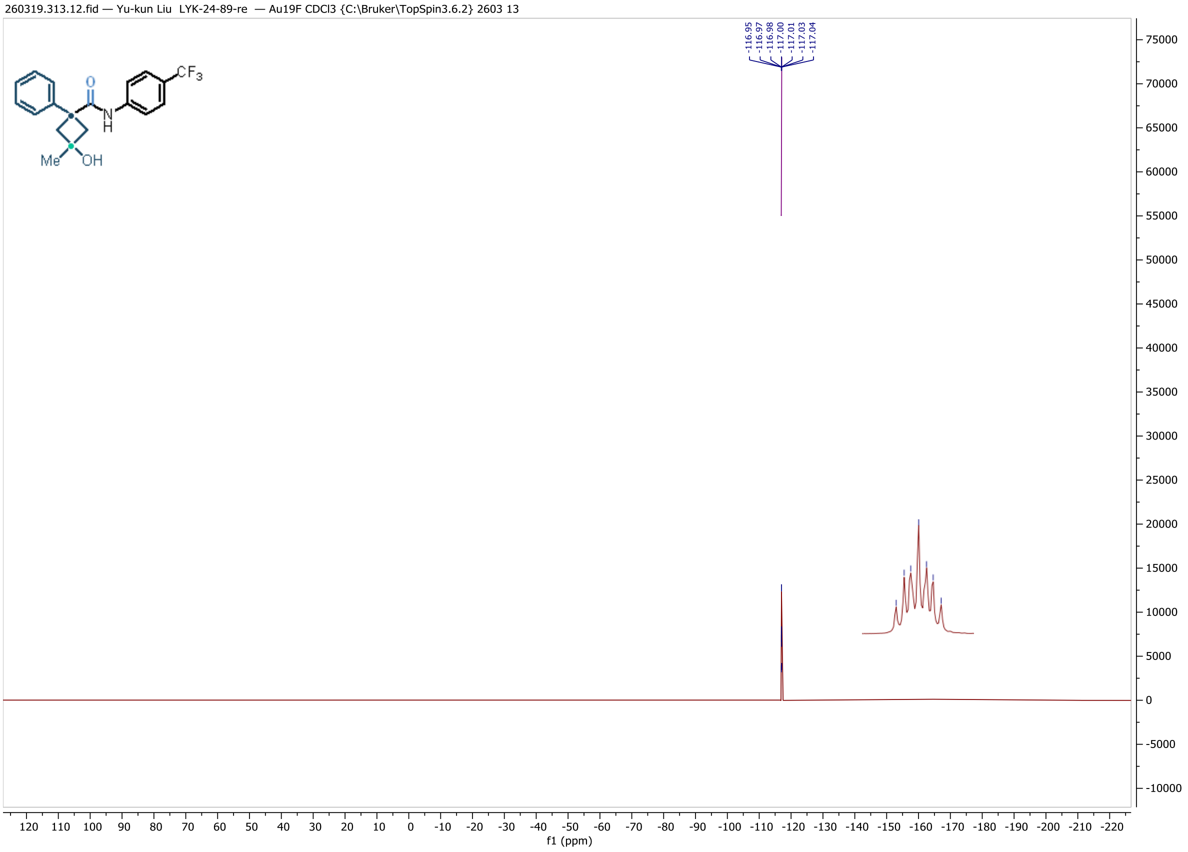

^{1}H NMR spectrum of **3m** (300 MHz, $CDCl_3$)

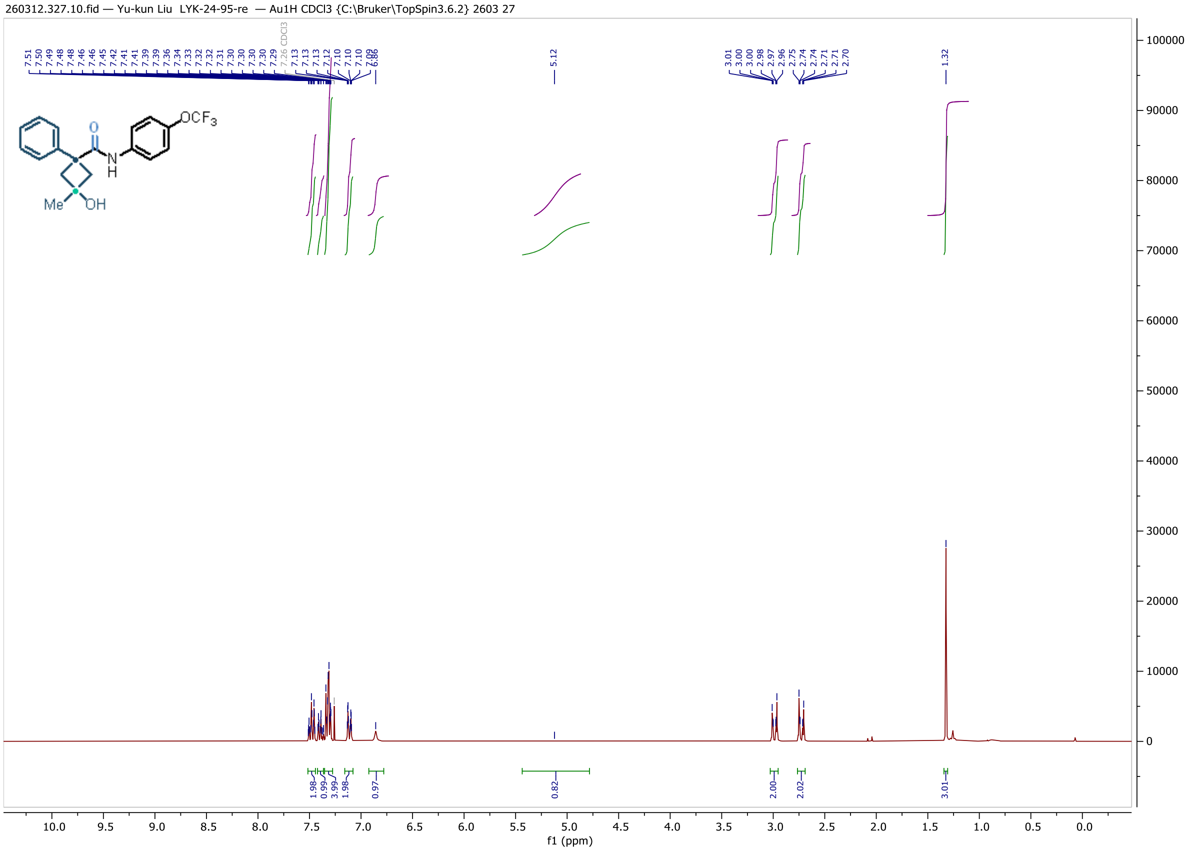

^{13}C NMR spectrum of **3m** (75 MHz, $CDCl_3$)

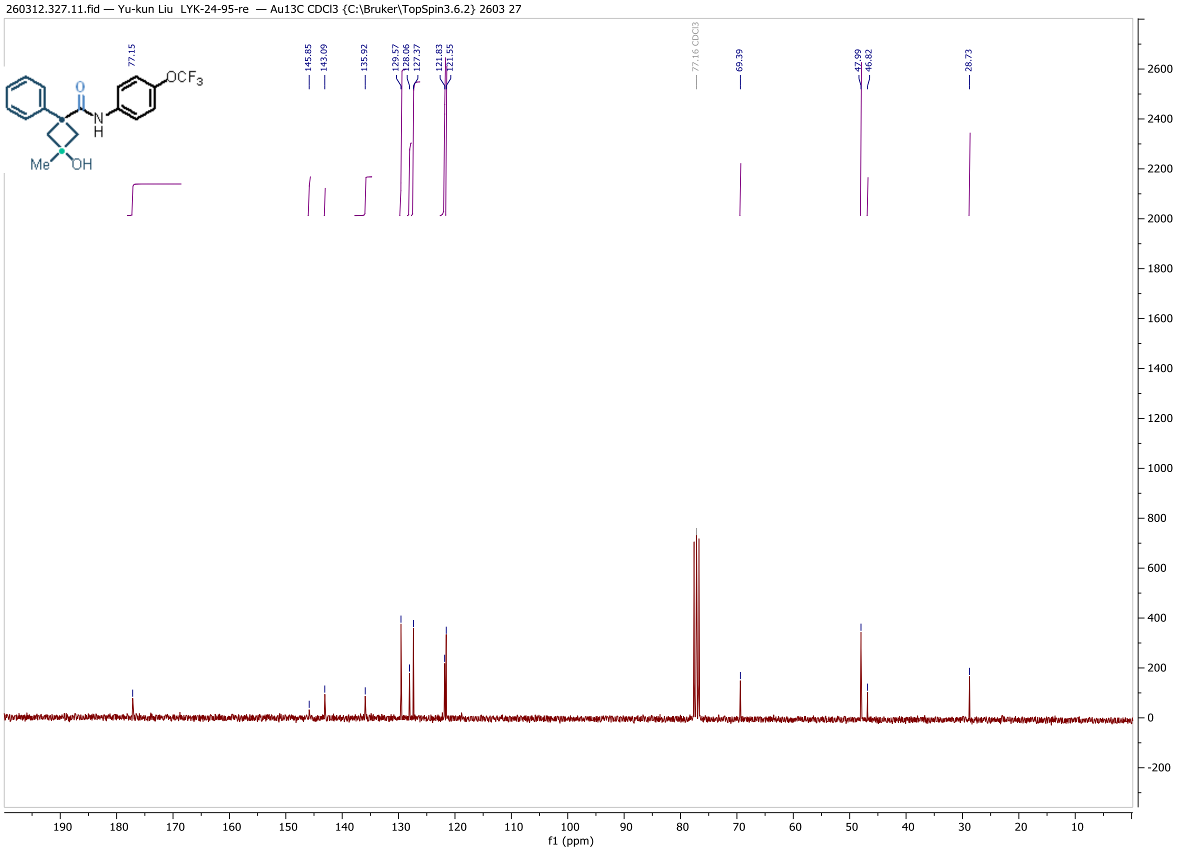

^{19}F NMR spectrum of **3m** (282 MHz, $CDCl_3$)

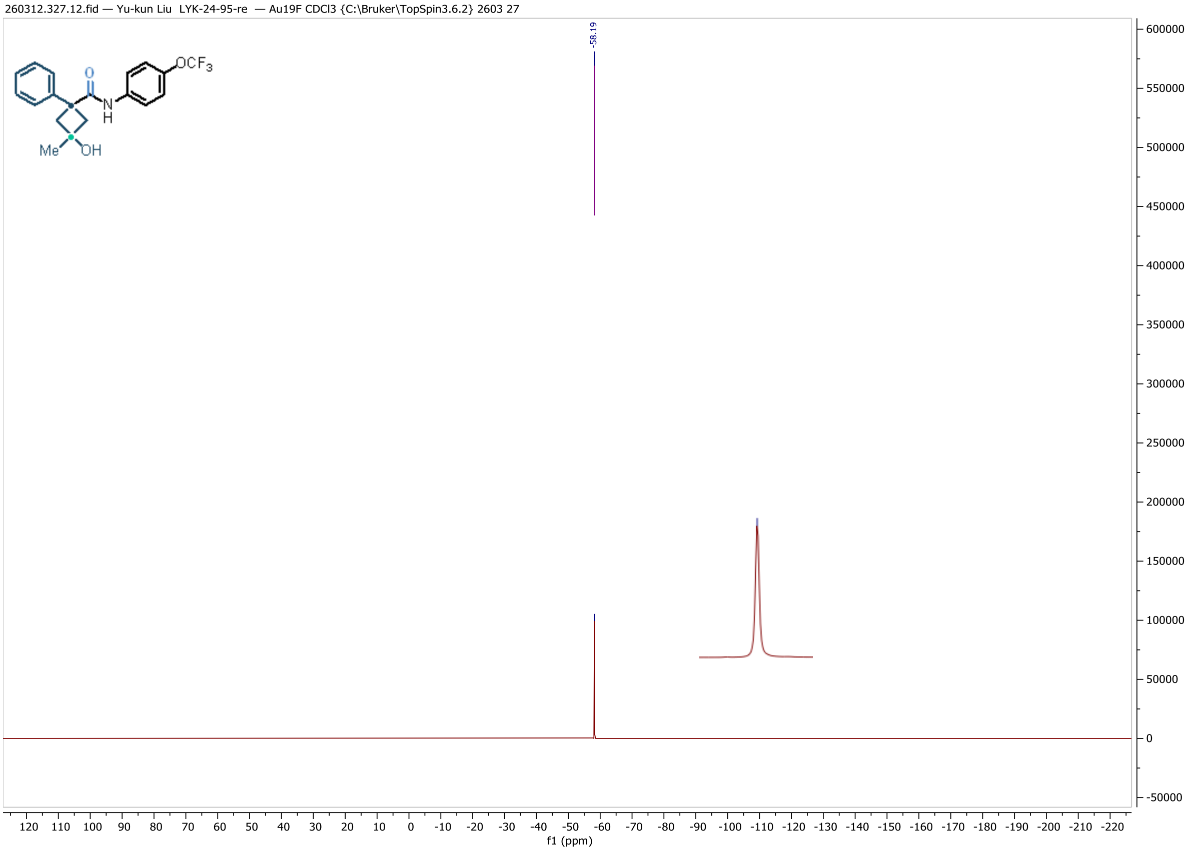

^{1}H NMR spectrum of **3n** (300 MHz, $CDCl_3$)

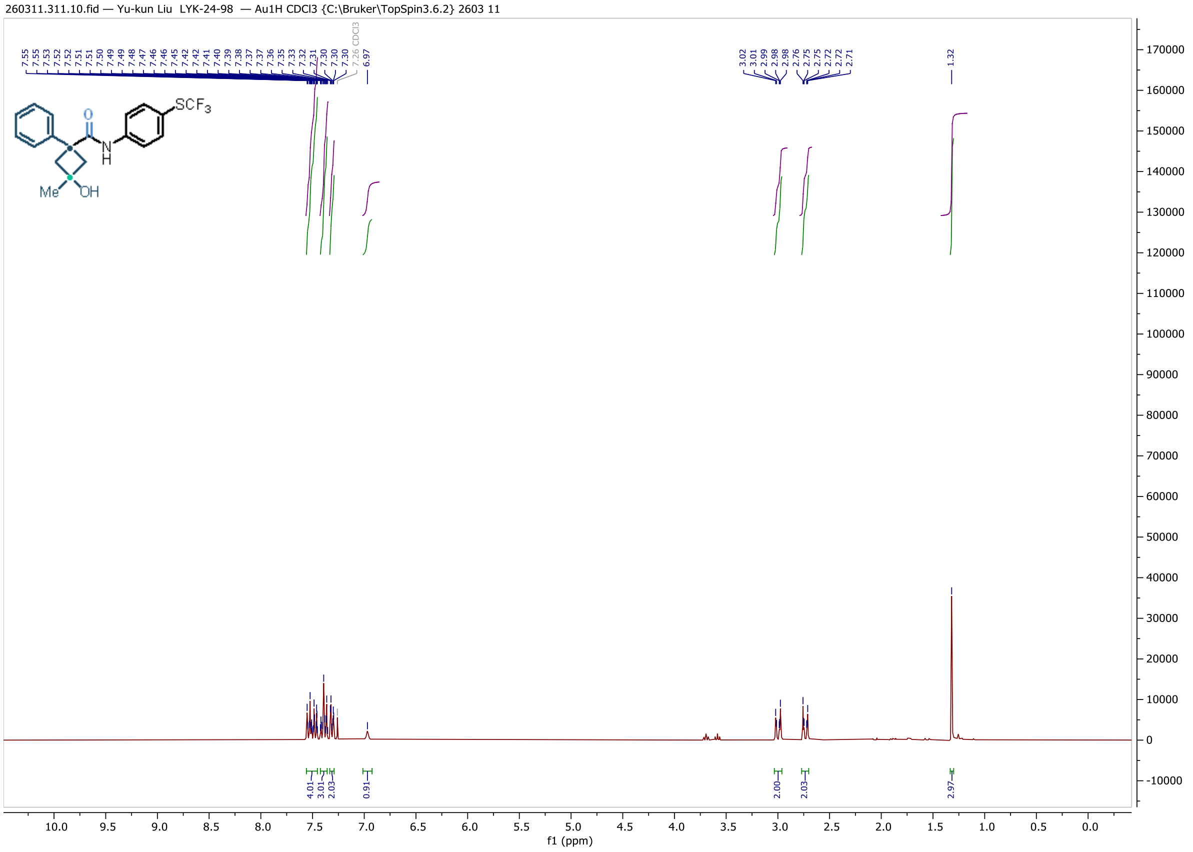

^{13}C NMR spectrum of **3n** (75 MHz, $CDCl_3$)

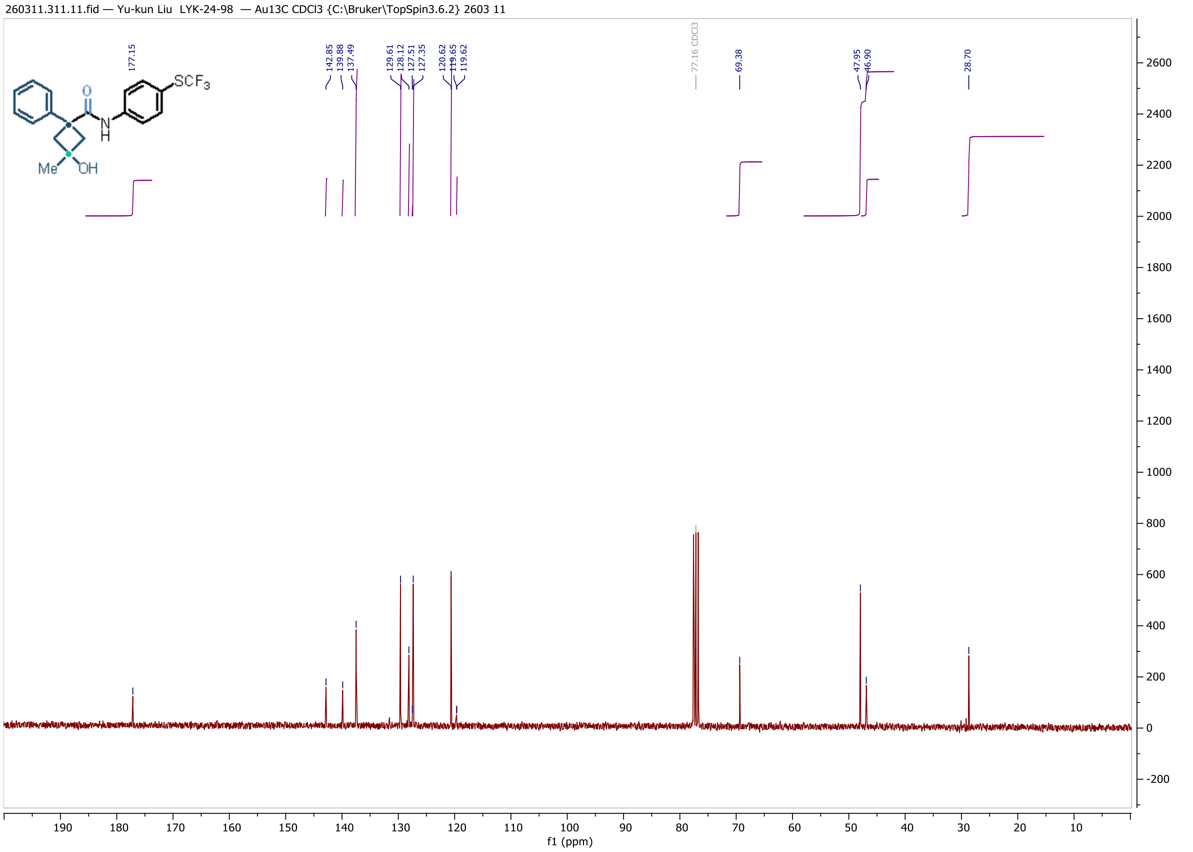

^{19}F NMR spectrum of **3n** (282 MHz, $CDCl_3$)

260311.311.12.fid — Yu-kun Liu LYK-24-98 — Au19F CDCl3 {C:\Bruker\TopSpin3.6.2} 2603 11

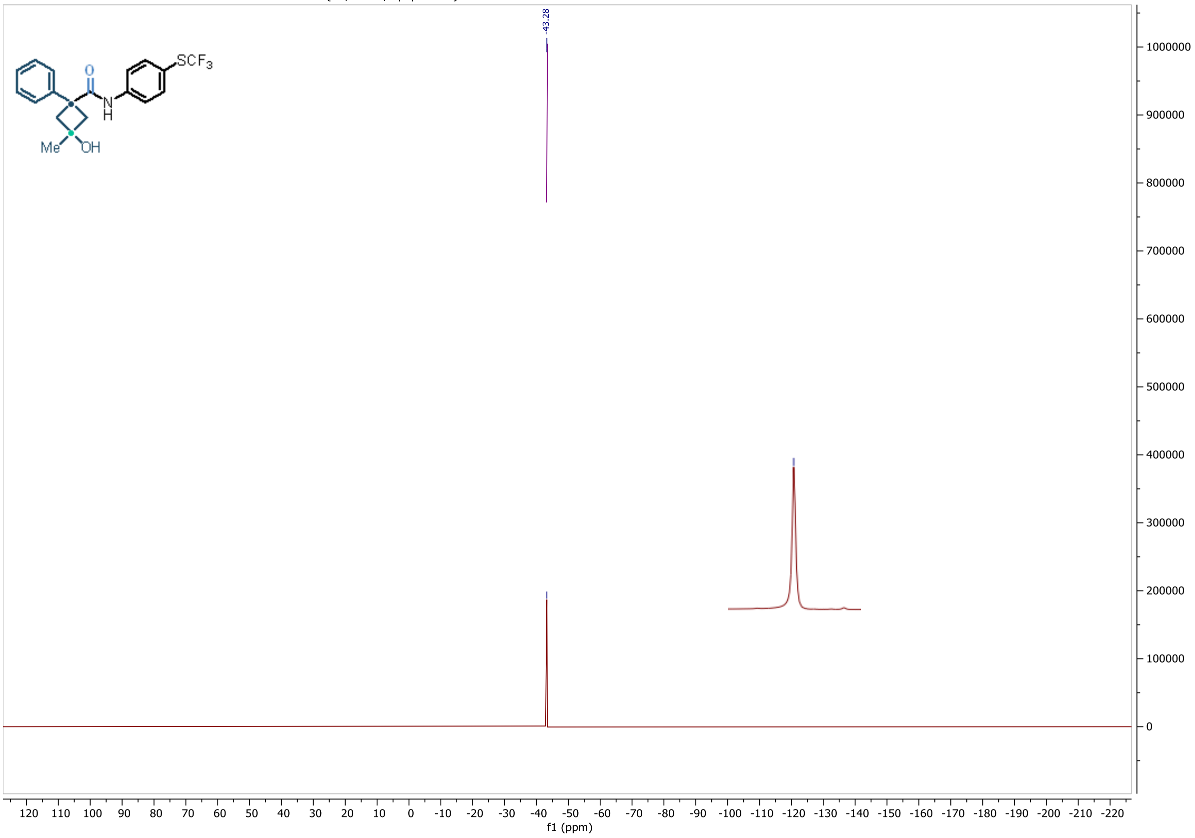

^{1}H NMR spectrum of **3o** (300 MHz, $CDCl_3$)

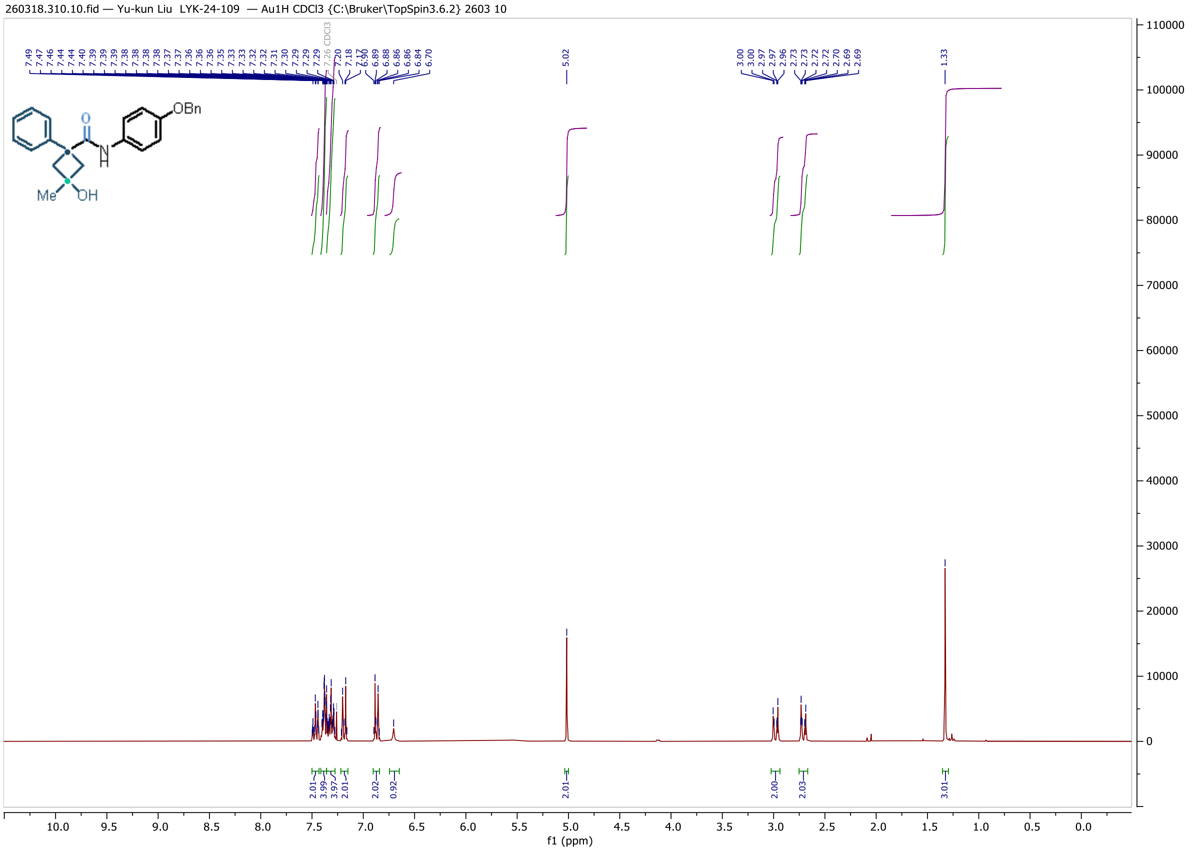

^{13}C NMR spectrum of **3o** (75 MHz, $CDCl_3$)

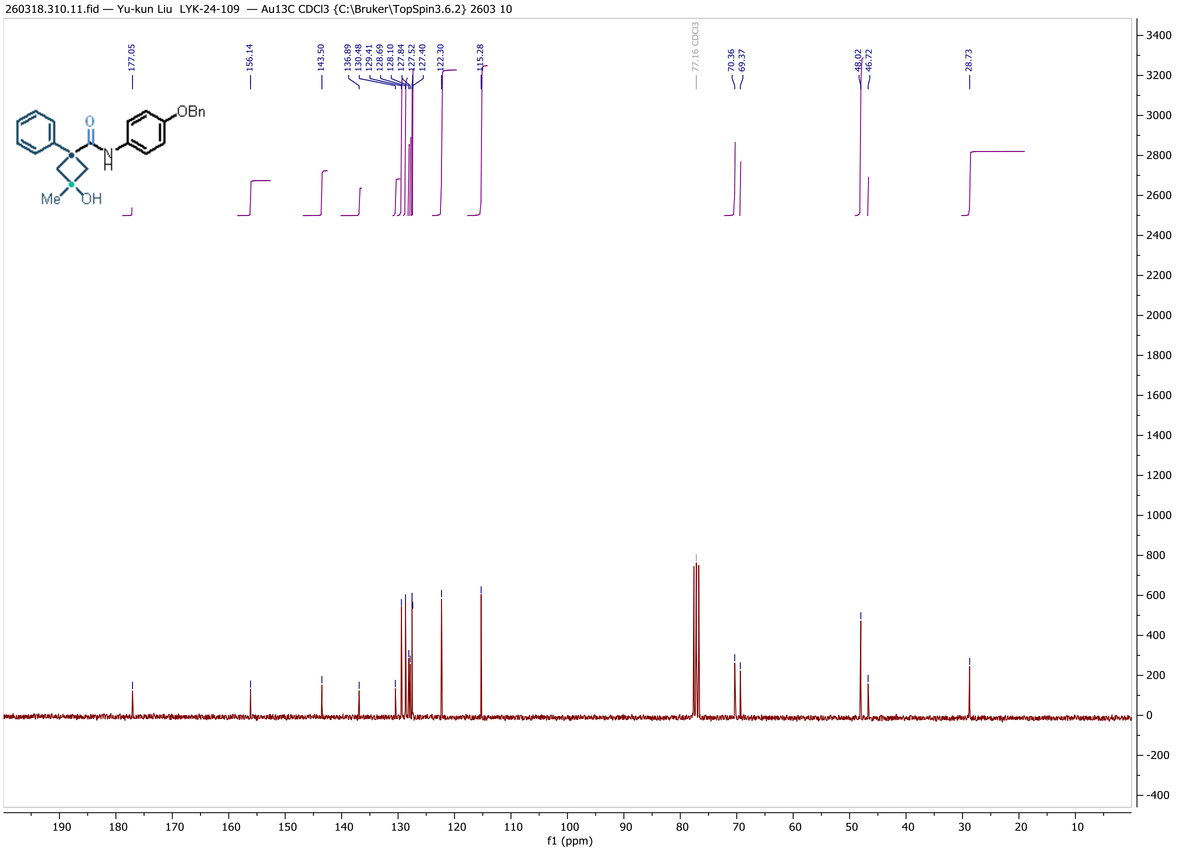

^{1}H NMR spectrum of **3p** (300 MHz, $CDCl_3$)

260326.f304.10.fid — Yu-kun Liu LYK-24-143 — Au1H CDCl3 {C:\Bruker\TopSpin3.6.2} 2603 4

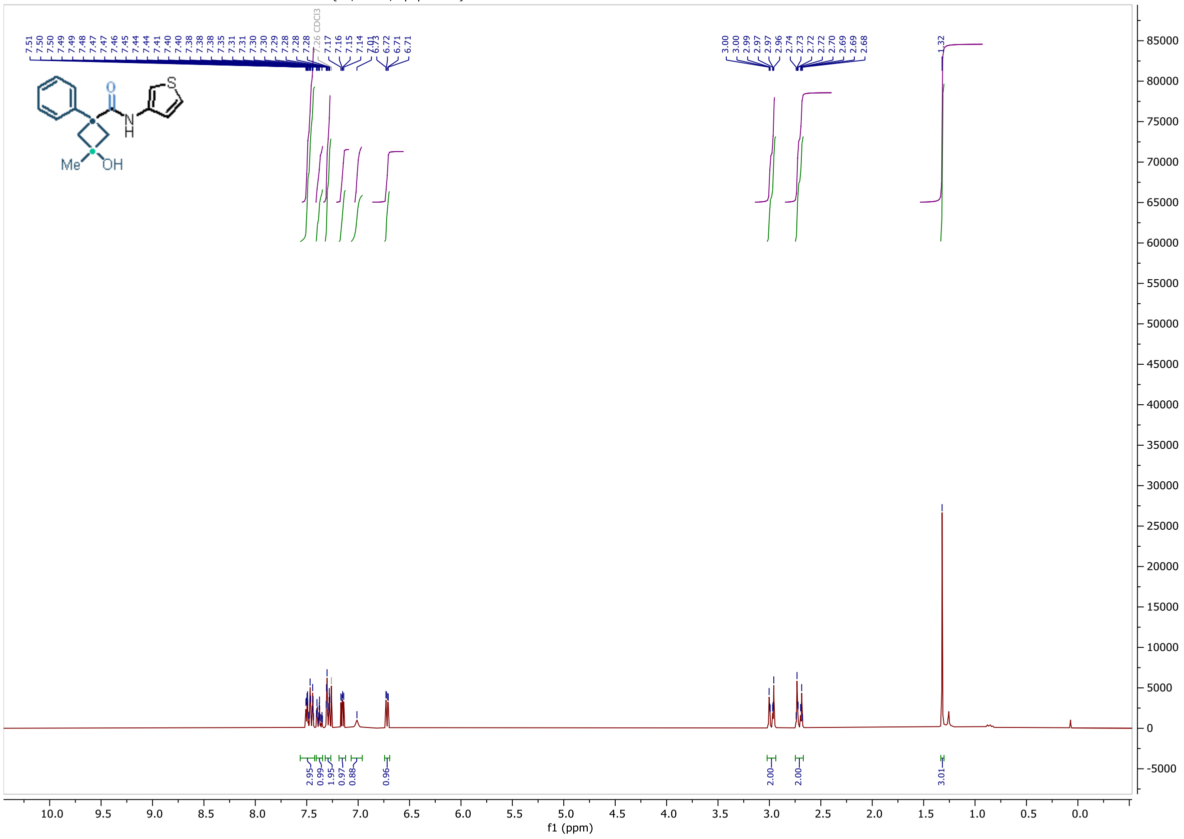

^{13}C NMR spectrum of **3p** (75 MHz, $CDCl_3$)

260326.f304.11.fid — Yu-kun Liu LYK-24-143 — Au13C CDCl3 {C:\Bruker\TopSpin3.6.2} 2603 4

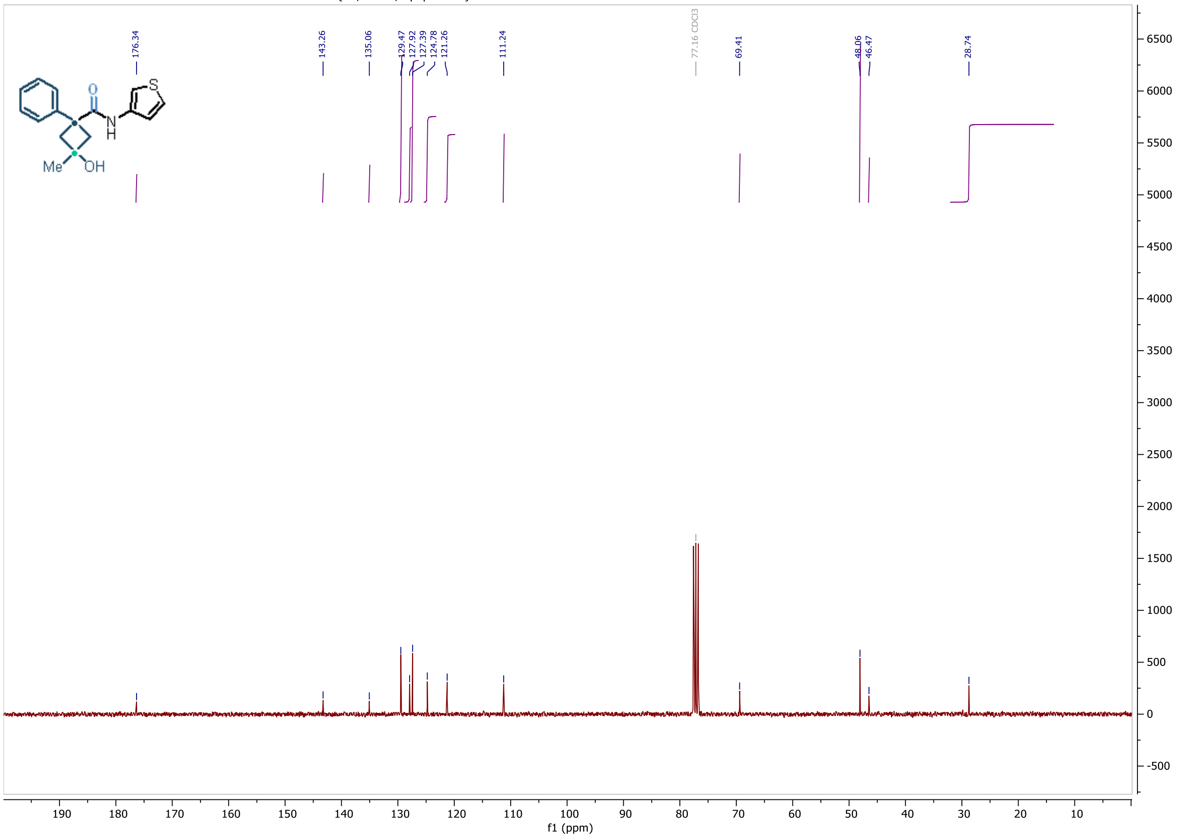

^{1}H NMR spectrum of **3q** (300 MHz, $CDCl_3$)

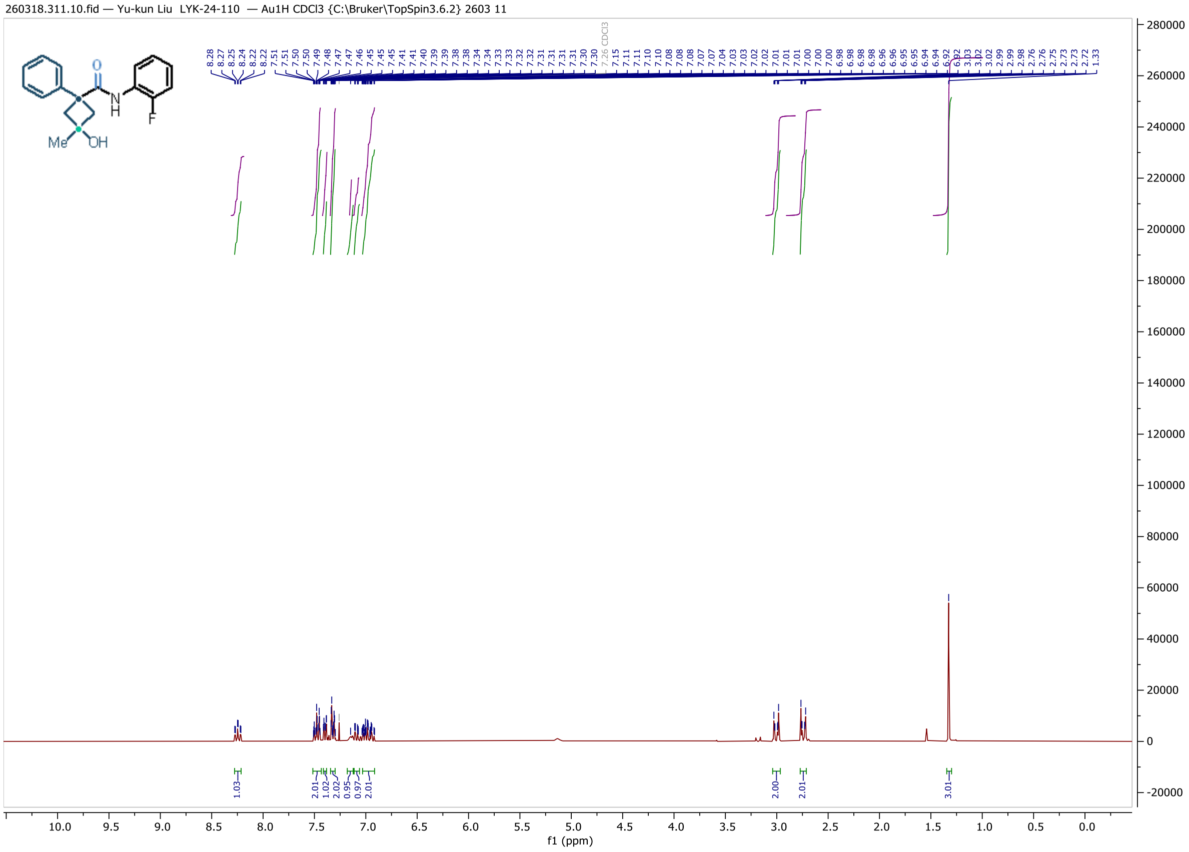

^{13}C NMR spectrum of **3q** (75 MHz, $CDCl_3$)

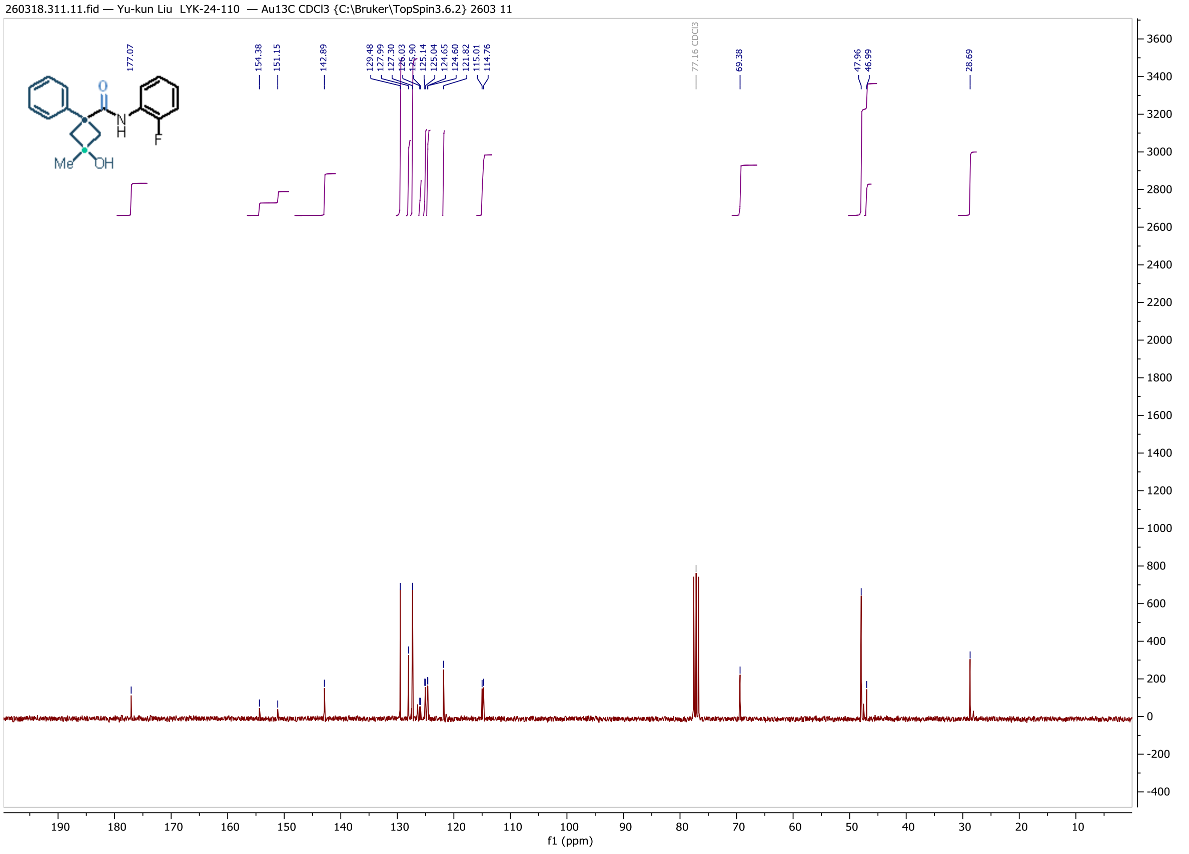

^{19}F NMR spectrum of **3q** (282 MHz, $CDCl_3$)

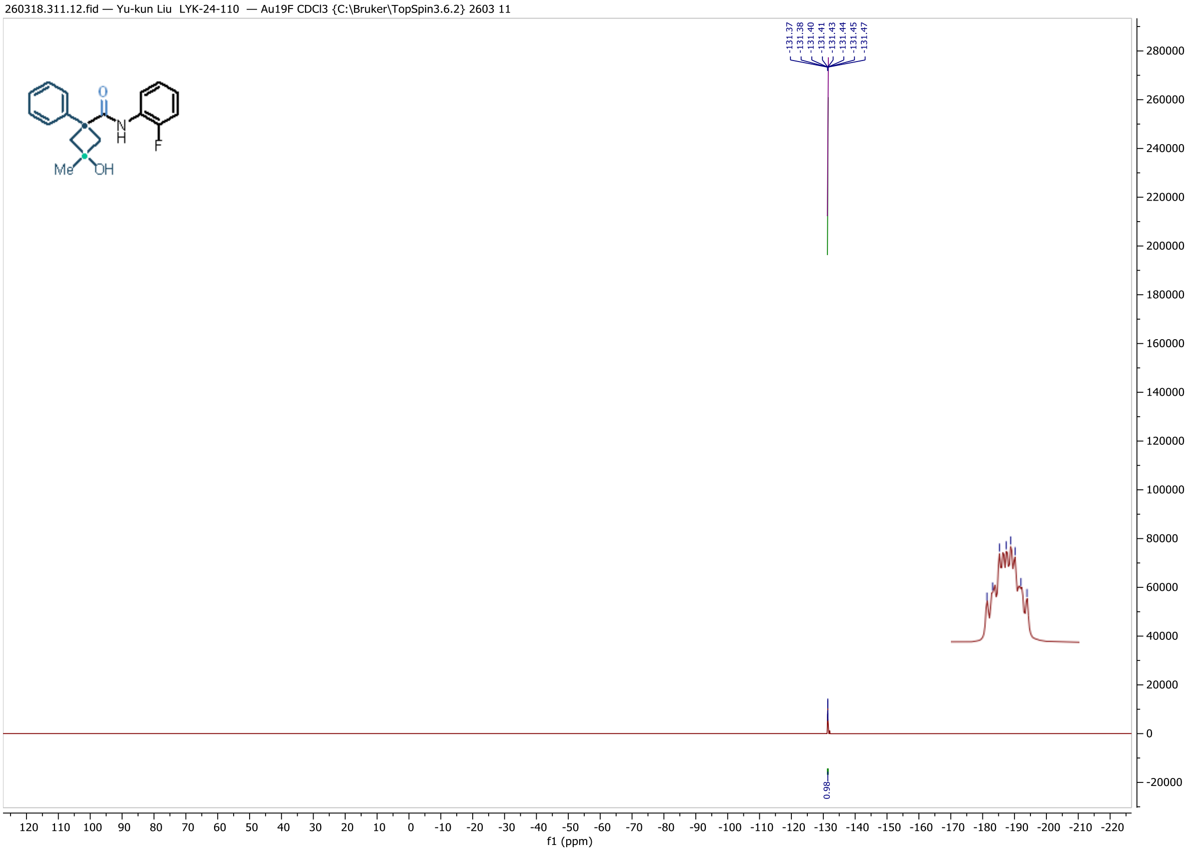

^{1}H NMR spectrum of **3r** (300 MHz, $CDCl_3$)

260320.304.10.fid — Yu-kun Liu LYK-24-128 — Au1H CDCl3 {C:\Bruker\TopSpin3.6.2} 2603 4

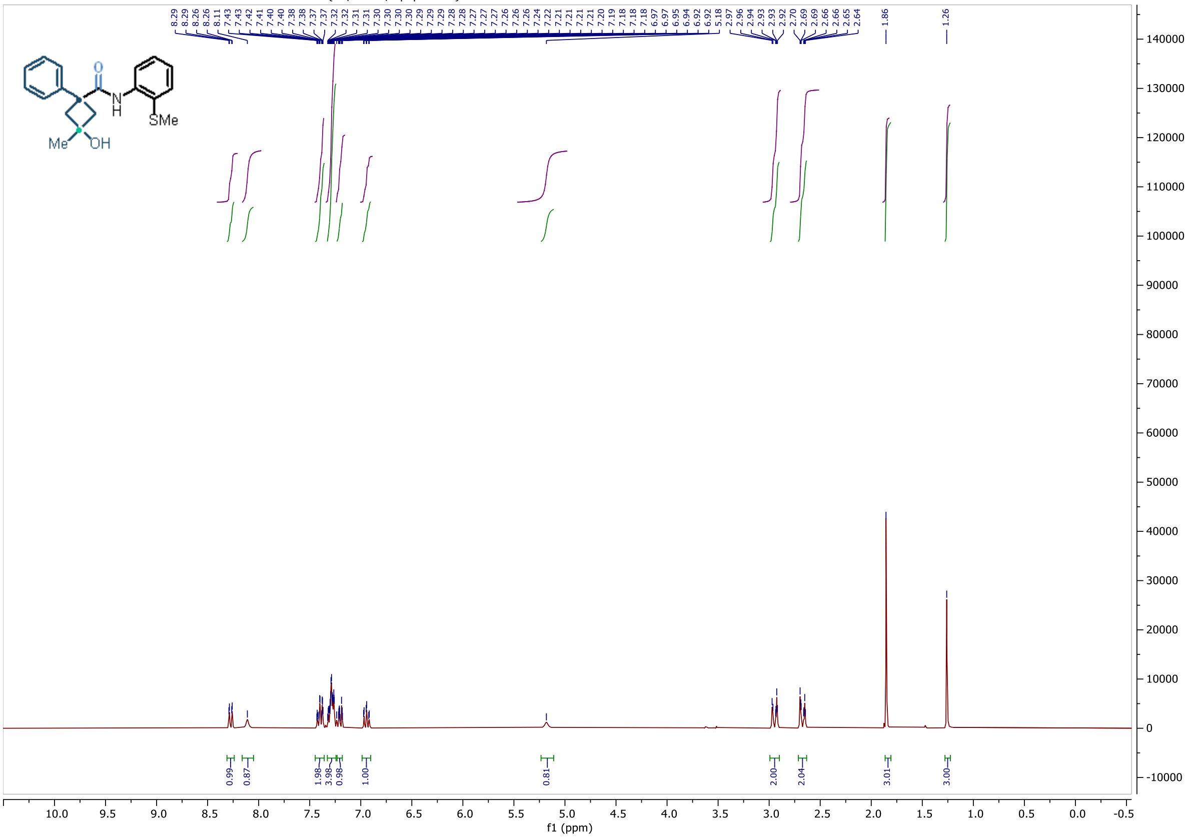

^{13}C NMR spectrum of **3r** (75 MHz, $CDCl_3$)

260320.304.11.fid — Yu-kun Liu LYK-24-128 — Au13C CDCl3 {C:\Bruker\TopSpin3.6.2} 2603 4

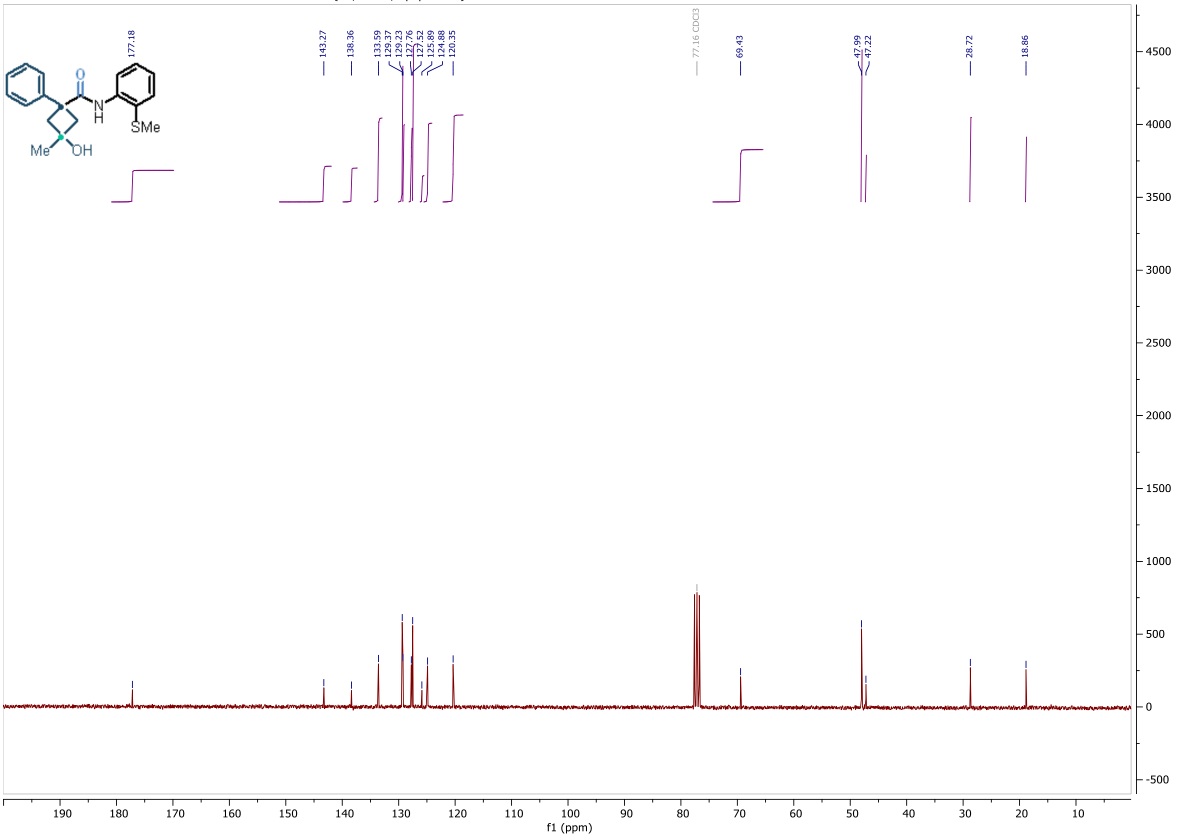

^{1}H NMR spectrum of **3s** (300 MHz, $CDCl_3$)

260320.306.10.fid — Yu-kun Liu LYK-24-130 — Au1H CDCl3 {C:\Bruker\TopSpin3.6.2} 2603 6

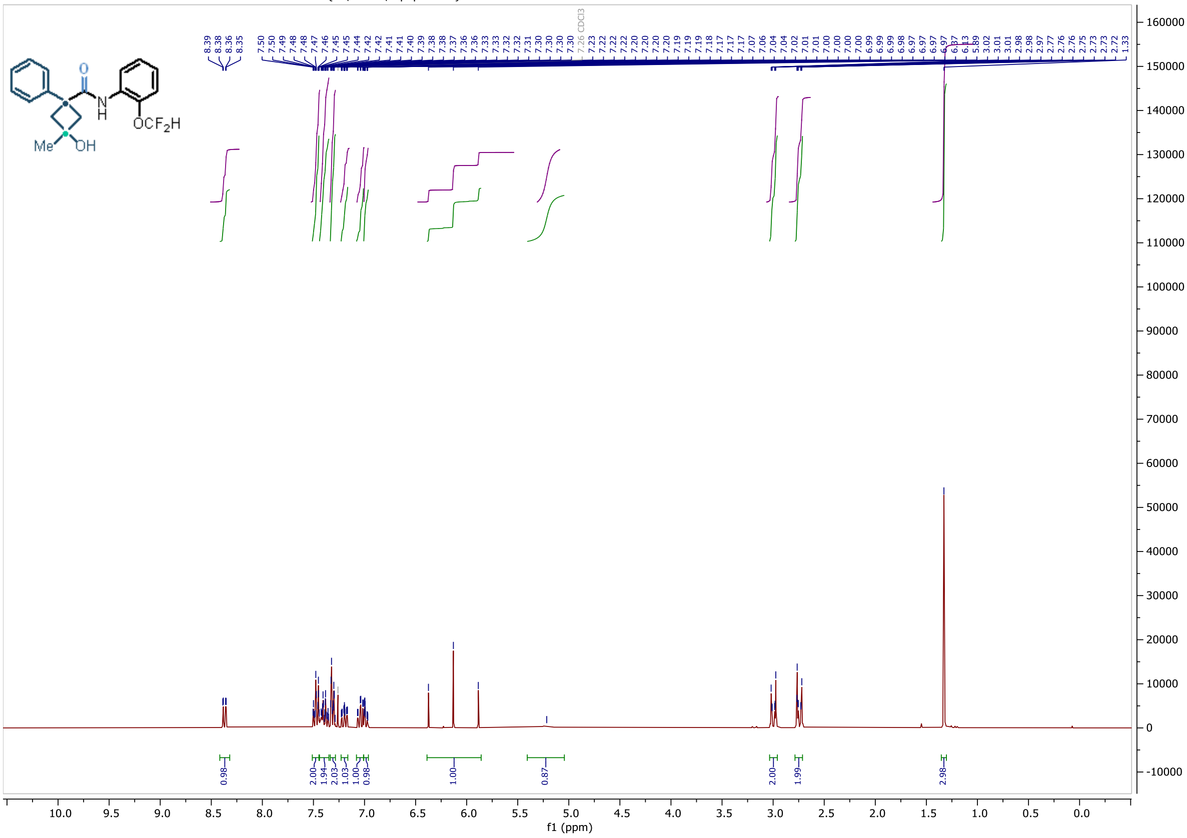

^{13}C NMR spectrum of **3s** (75 MHz, $CDCl_3$)

260320.306.11.fid — Yu-kun Liu LYK-24-130 — Au13C CDCl3 {C:\Bruker\TopSpin3.6.2} 2603 6

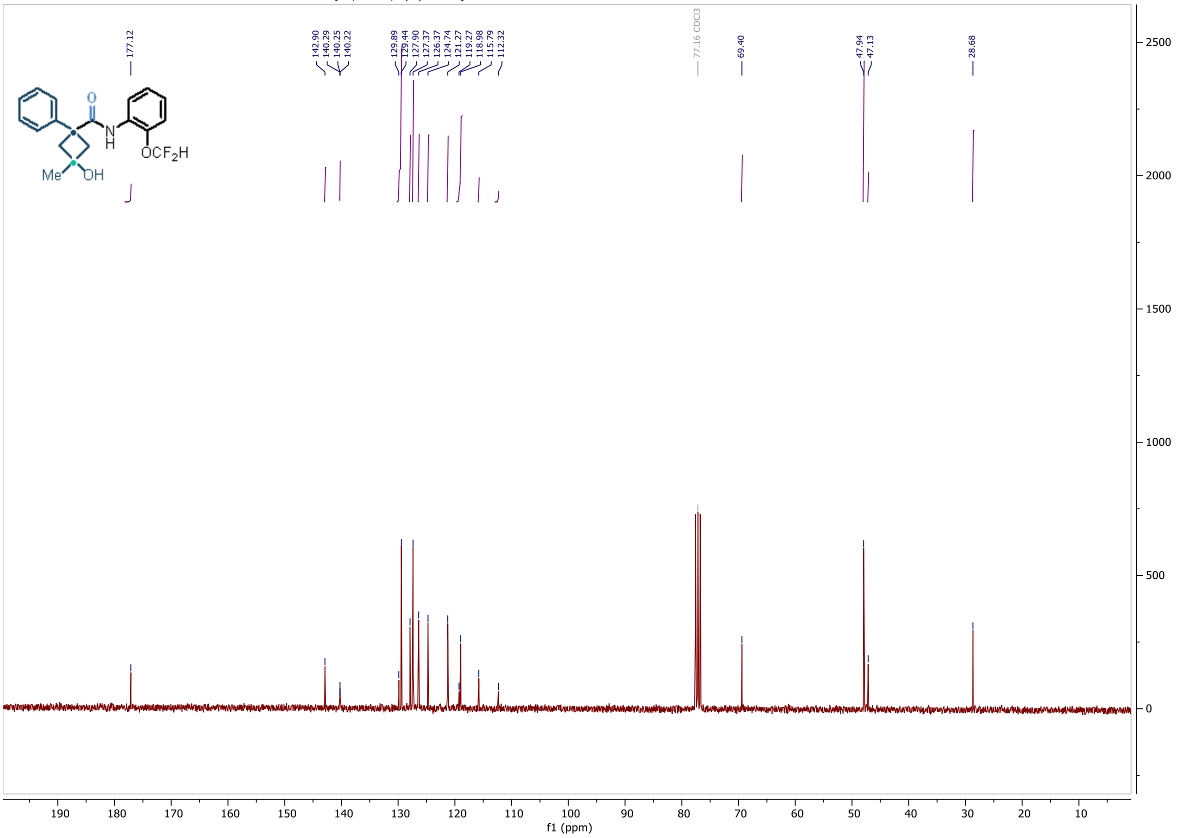

^{19}F NMR spectrum of **3s** (282 MHz, $CDCl_3$)

260320.306.12.fid — Yu-kun Liu LYK-24-130 — Au19F CDCl3 {C:\Bruker\TopSpin3.6.2} 2603 6

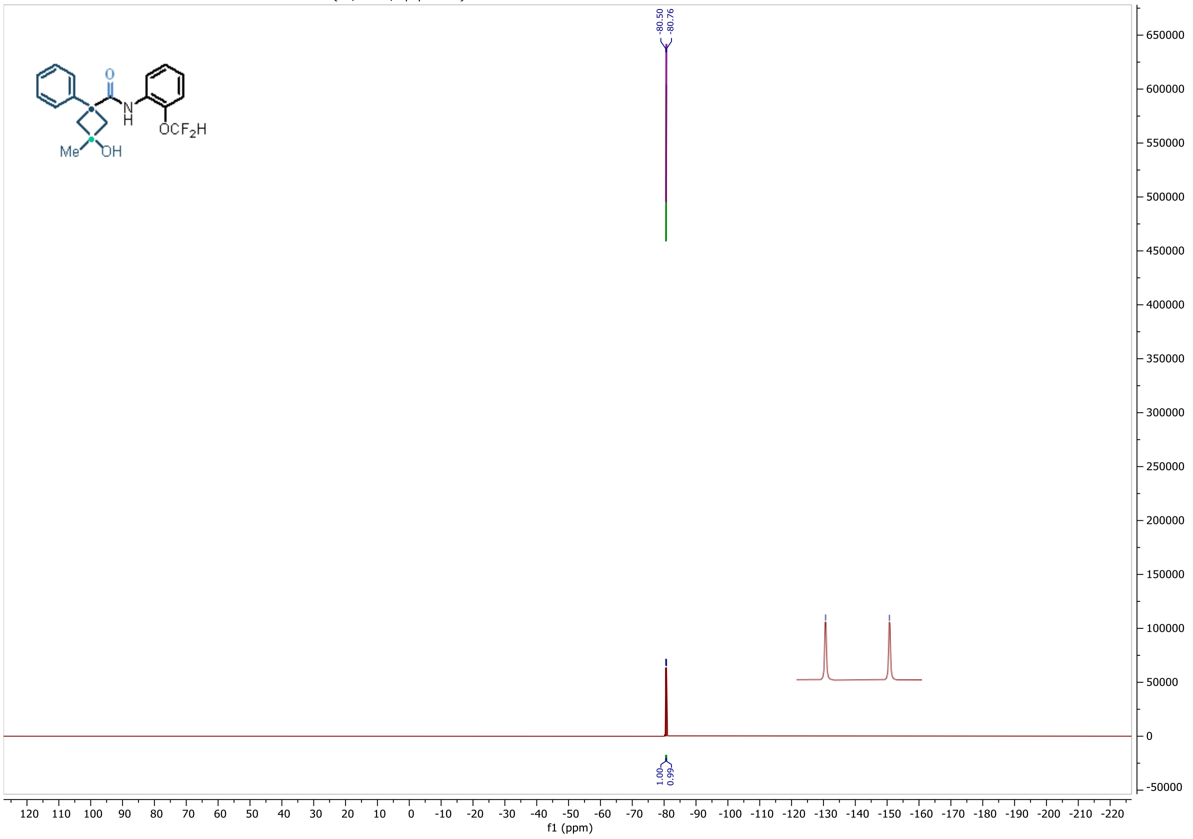

^{1}H NMR spectrum of **3t** (300 MHz, $CDCl_3$)

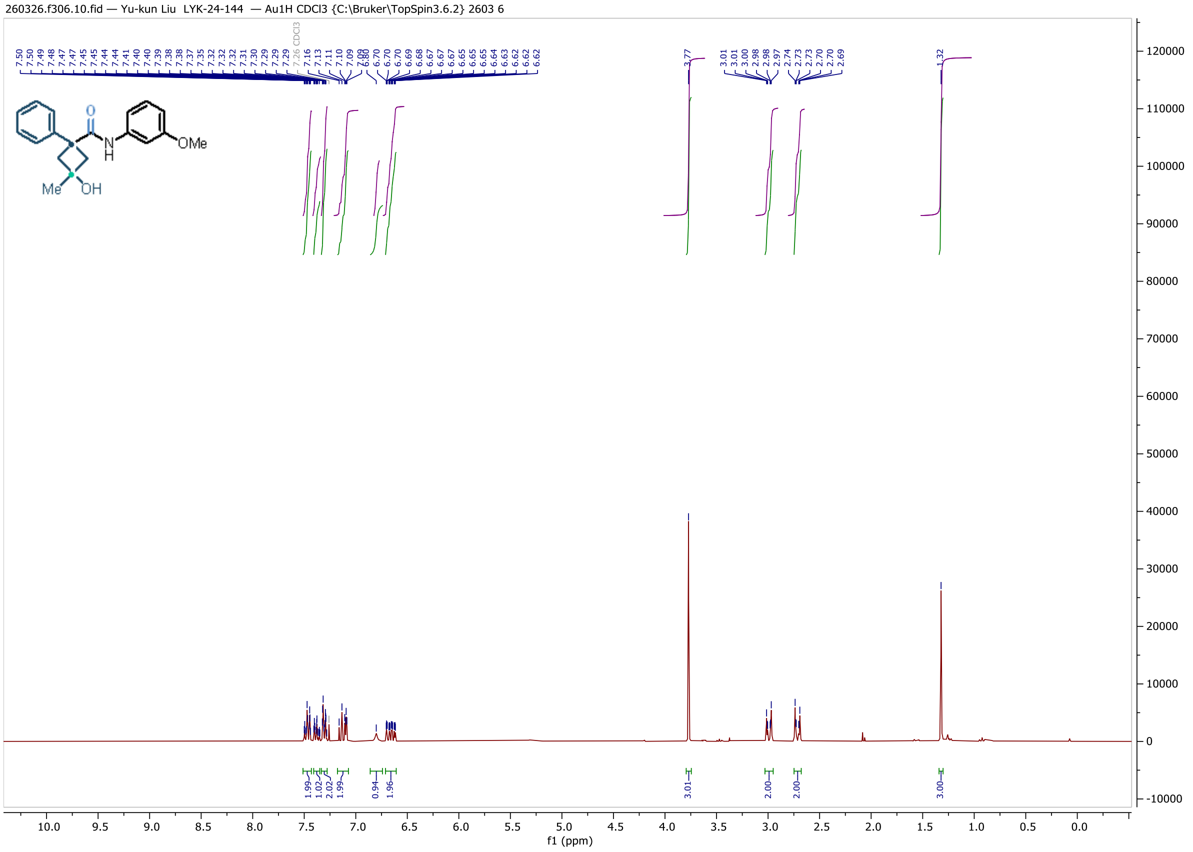

^{13}C NMR spectrum of **3t** (75 MHz, $CDCl_3$)

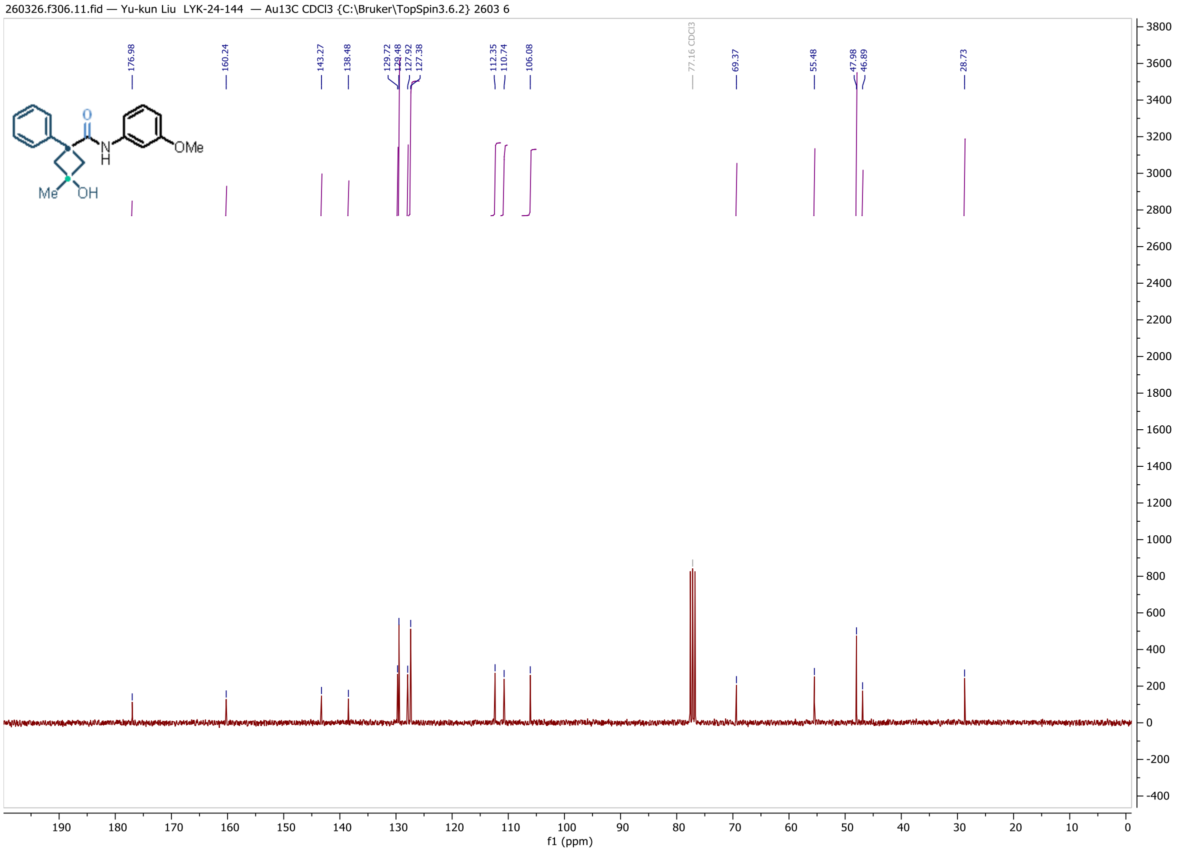

^{1}H NMR spectrum of **3u** (300 MHz, $CDCl_3$)

260312.330.10.fid — Yu-kun Liu LYK-24-103 — Au1H CDCl3 {C:\Bruker\TopSpin3.6.2} 2603 30

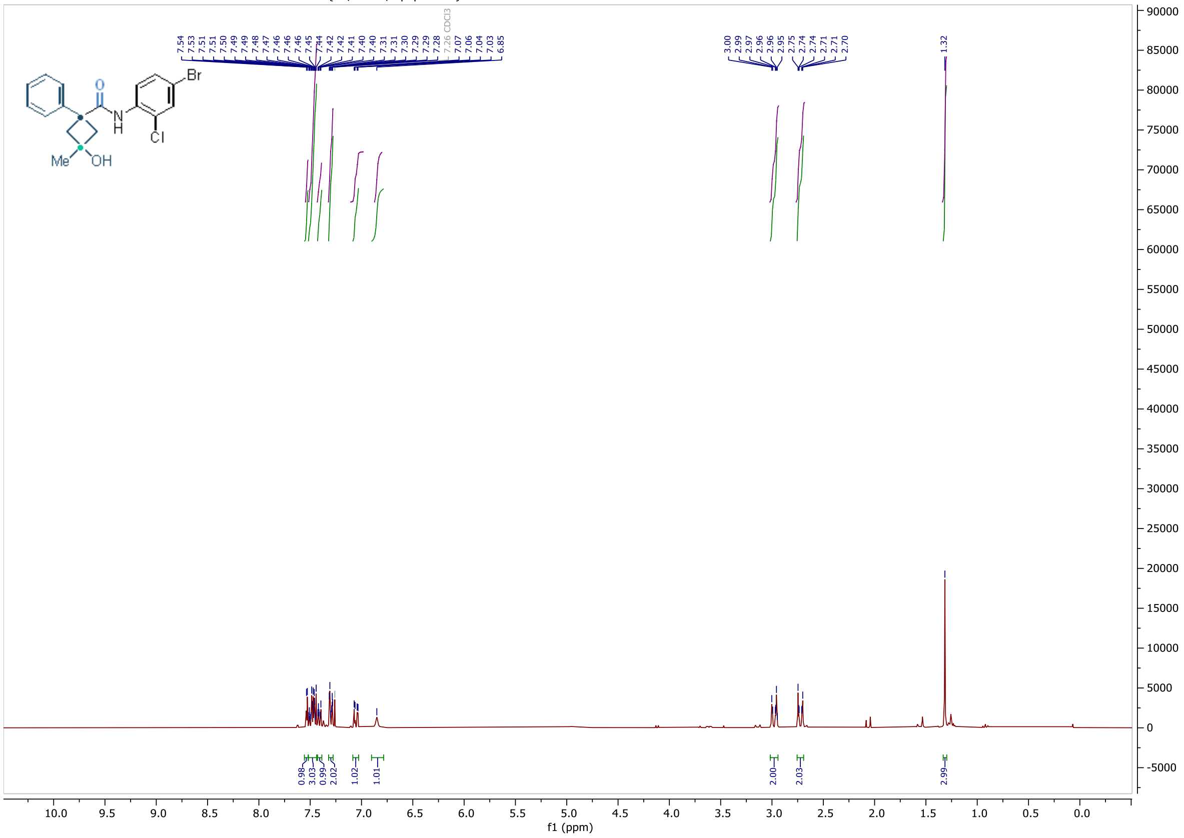

^{13}C NMR spectrum of **3u** (75 MHz, $CDCl_3$)

260312.330.11.fid — Yu-kun Liu LYK-24-103 — Au13C CDCl3 {C:\Bruker\TopSpin3.6.2} 2603 30

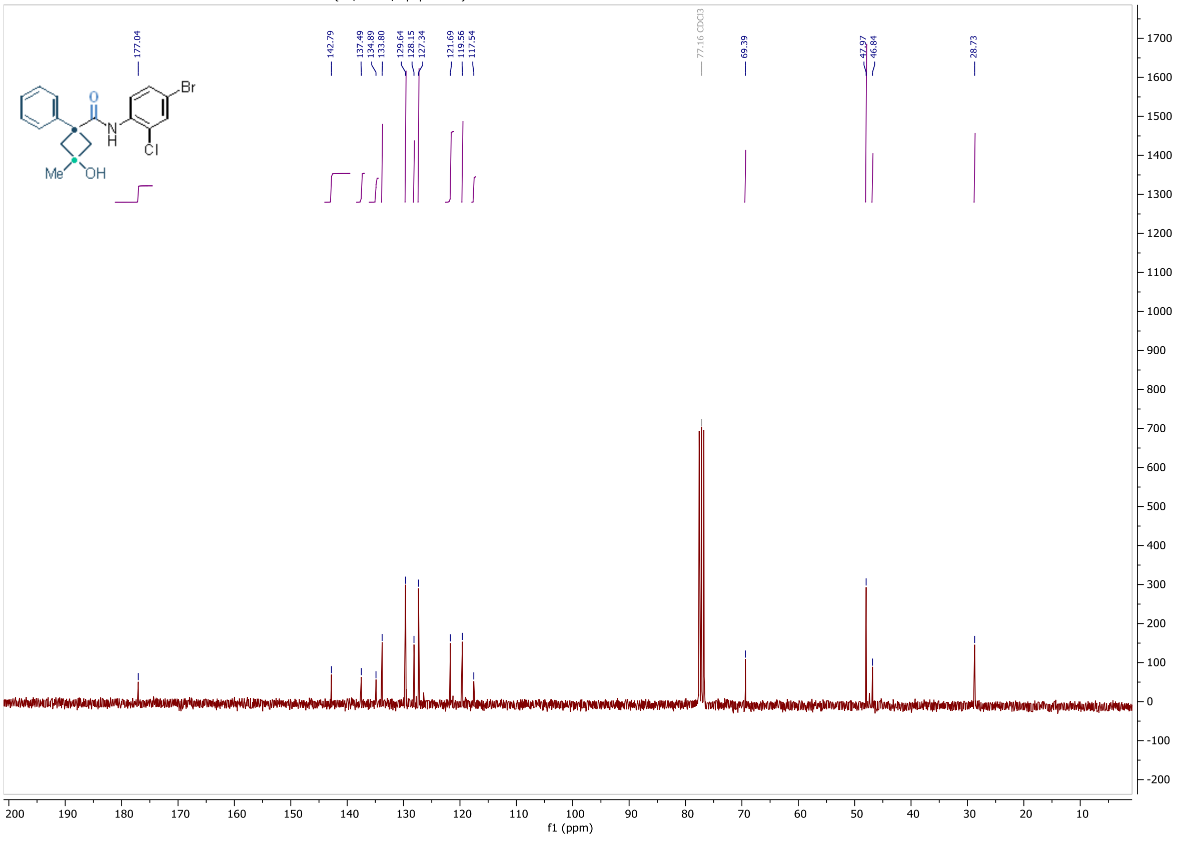

^{1}H NMR spectrum of **3v** (300 MHz, $CDCl_3$)

260331.310.10.fid — Yu-kun Liu LYK-24-150-H — Au1H CDCl3 {C:\Bruker\TopSpin3.6.2} 2603 10

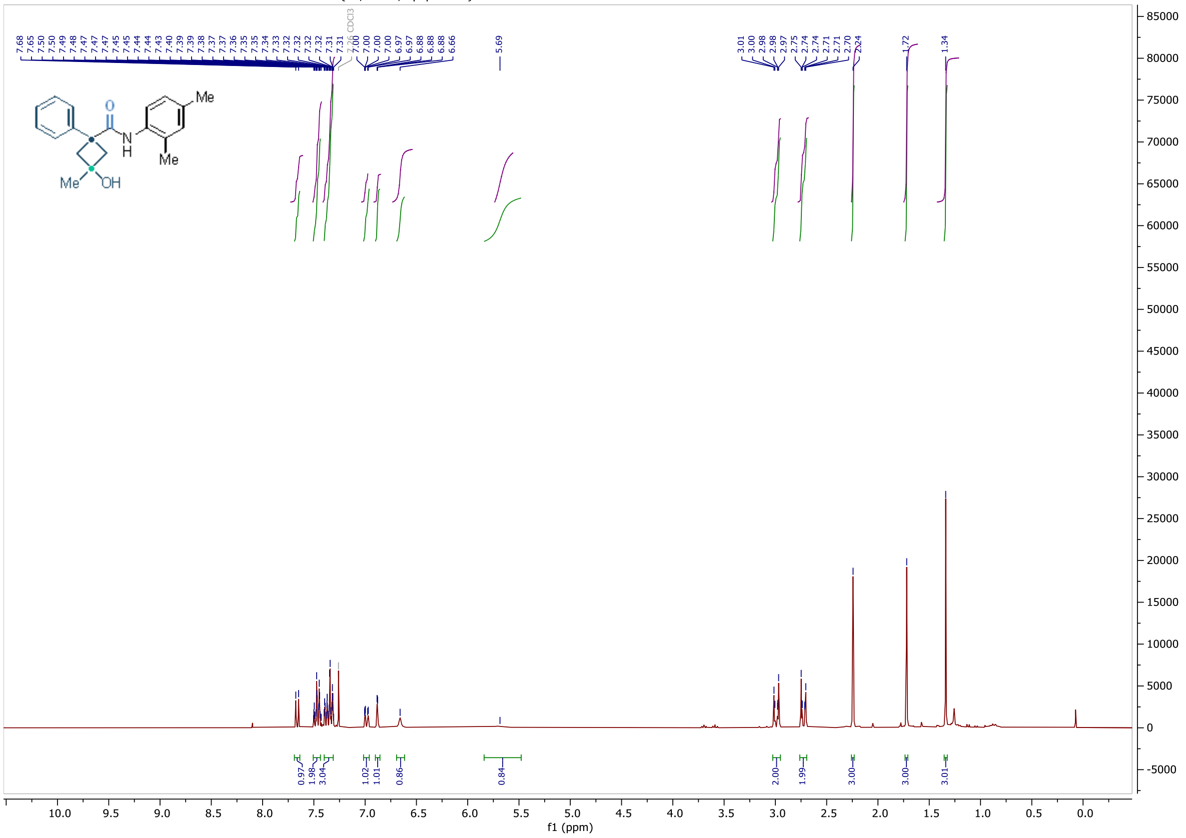

^{13}C NMR spectrum of **3v** (75 MHz, $CDCl_3$)

260331.407.10.fid — Yu-Kun Liu LYK-24-150 — Au13C CDCl3 {C:\Bruker\TopSpin3.6.2} 2603 7

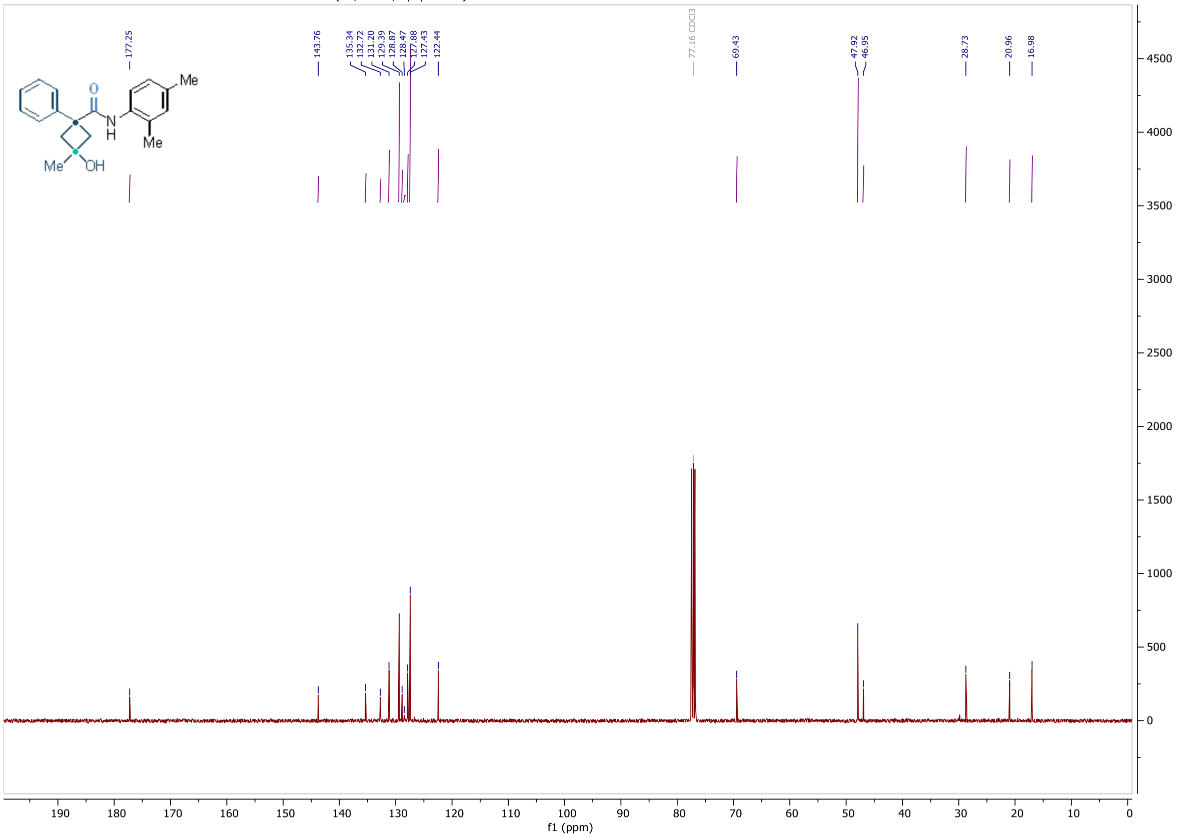

^{1}H NMR spectrum of **3w** (300 MHz, $CDCl_3$)

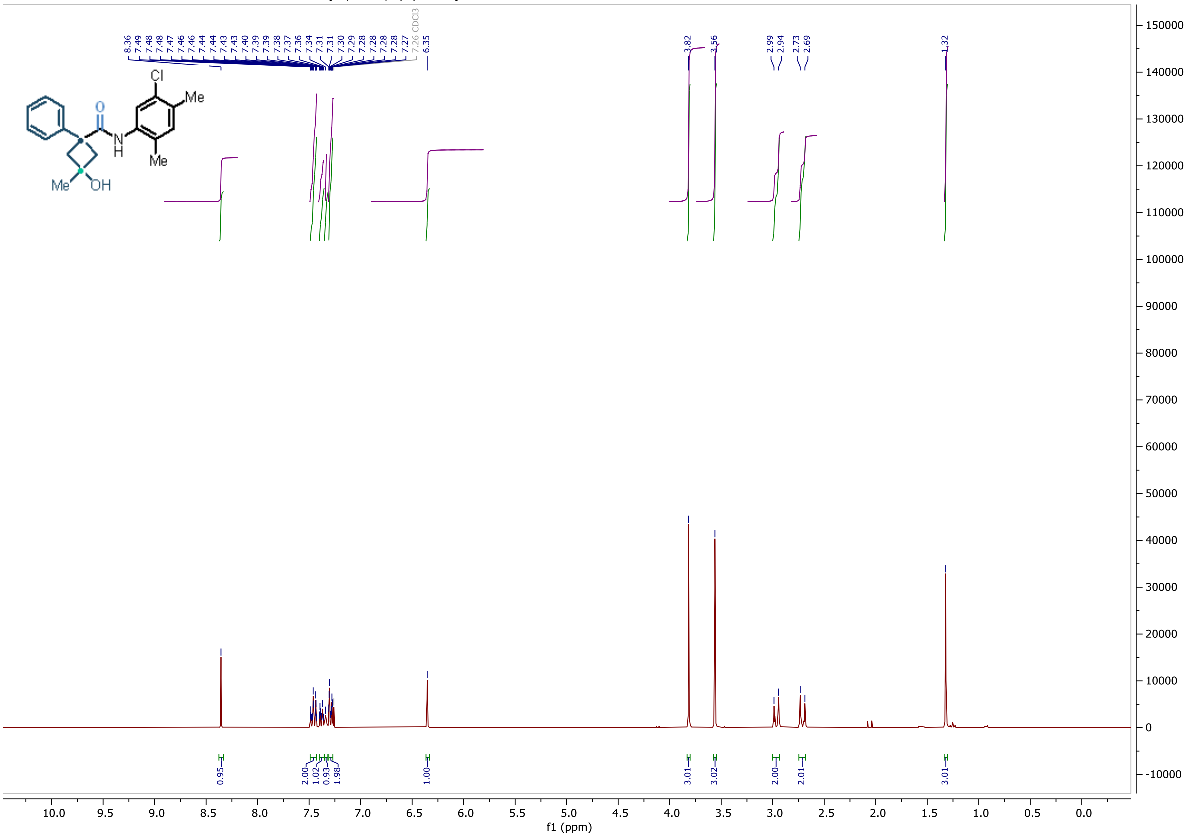

^{13}C NMR spectrum of **3w** (75 MHz, $CDCl_3$)

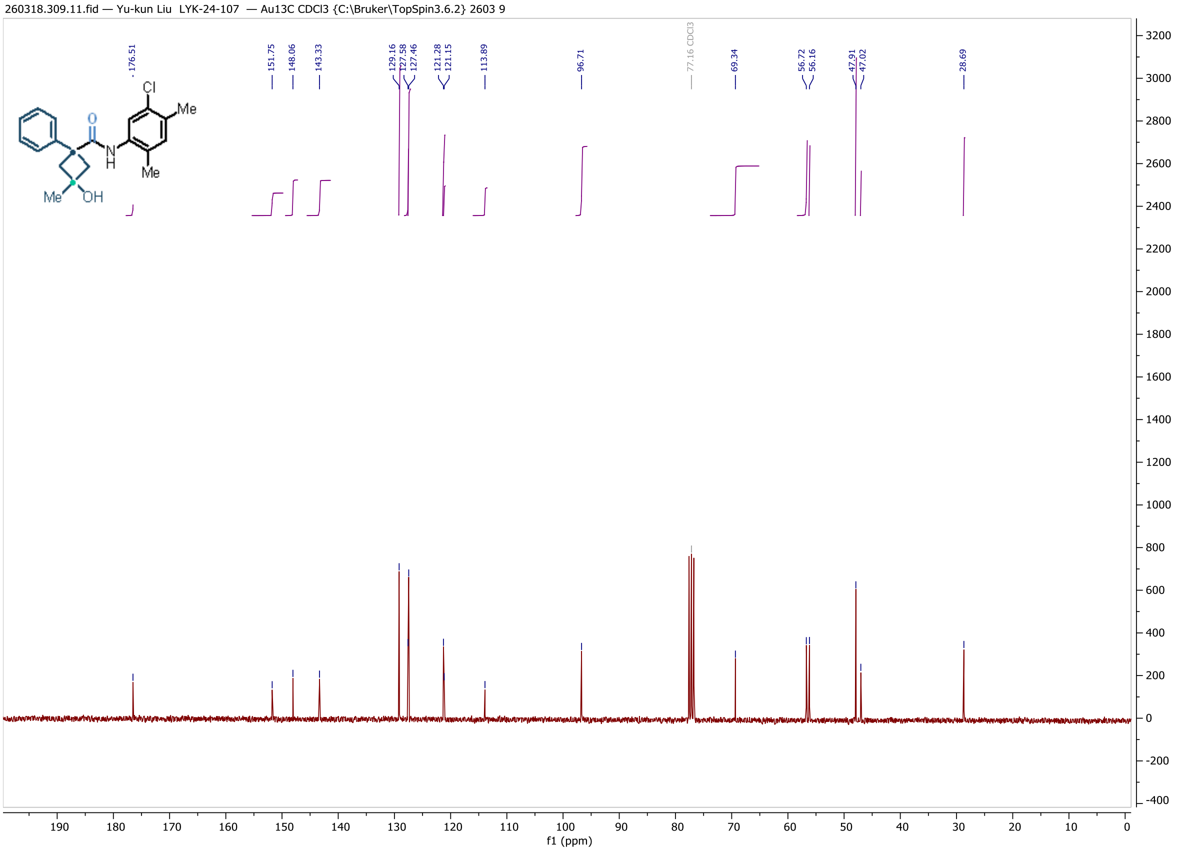

^{1}H NMR spectrum of **3x** (300 MHz, $CDCl_3$)

260319.336.10.fid — Yu-ku Liu, LYK-24-104-re-2 — Au1H CDCl3 {C:\Bruker\TopSpin3.6.2} 2603 36

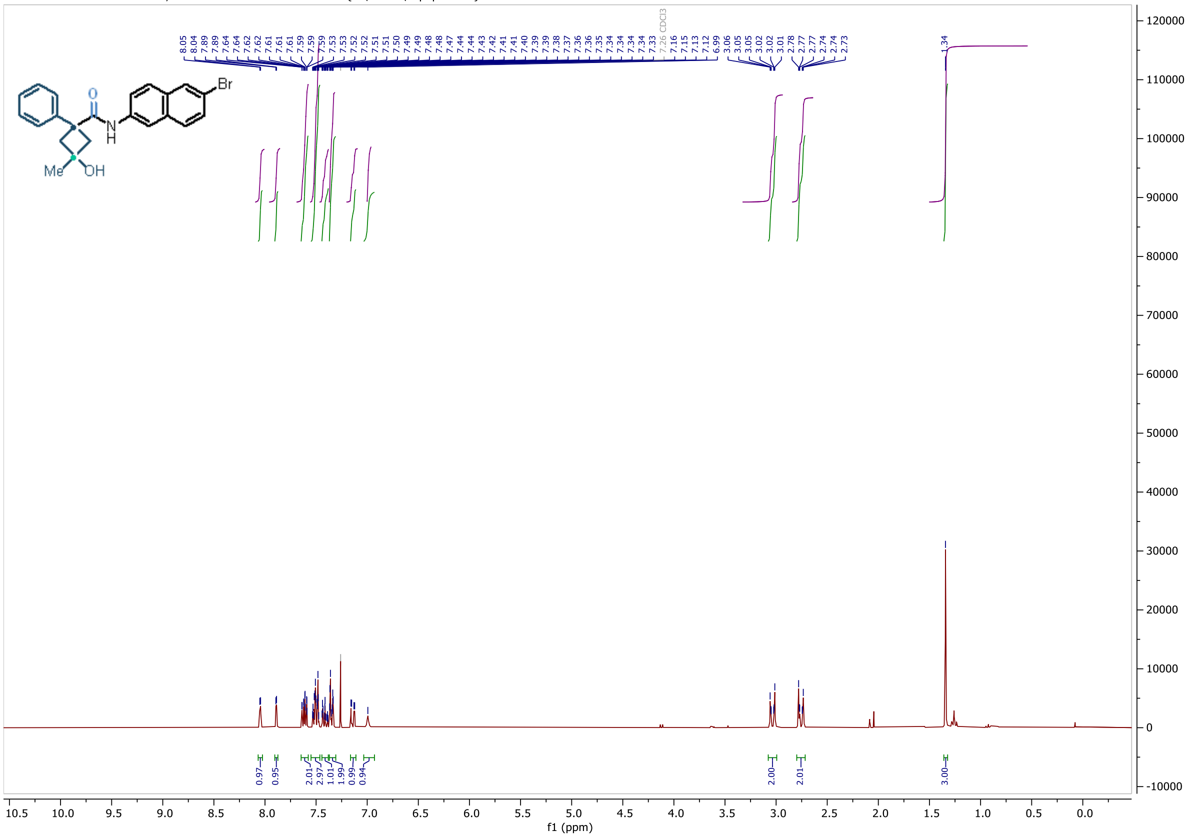

^{13}C NMR spectrum of **3x** (75 MHz, $CDCl_3$)

260319.336.11.fid — Yu-ku Liu, LYK-24-104-re-2 — Au13C CDCl3 {C:\Bruker\TopSpin3.6.2} 2603 36

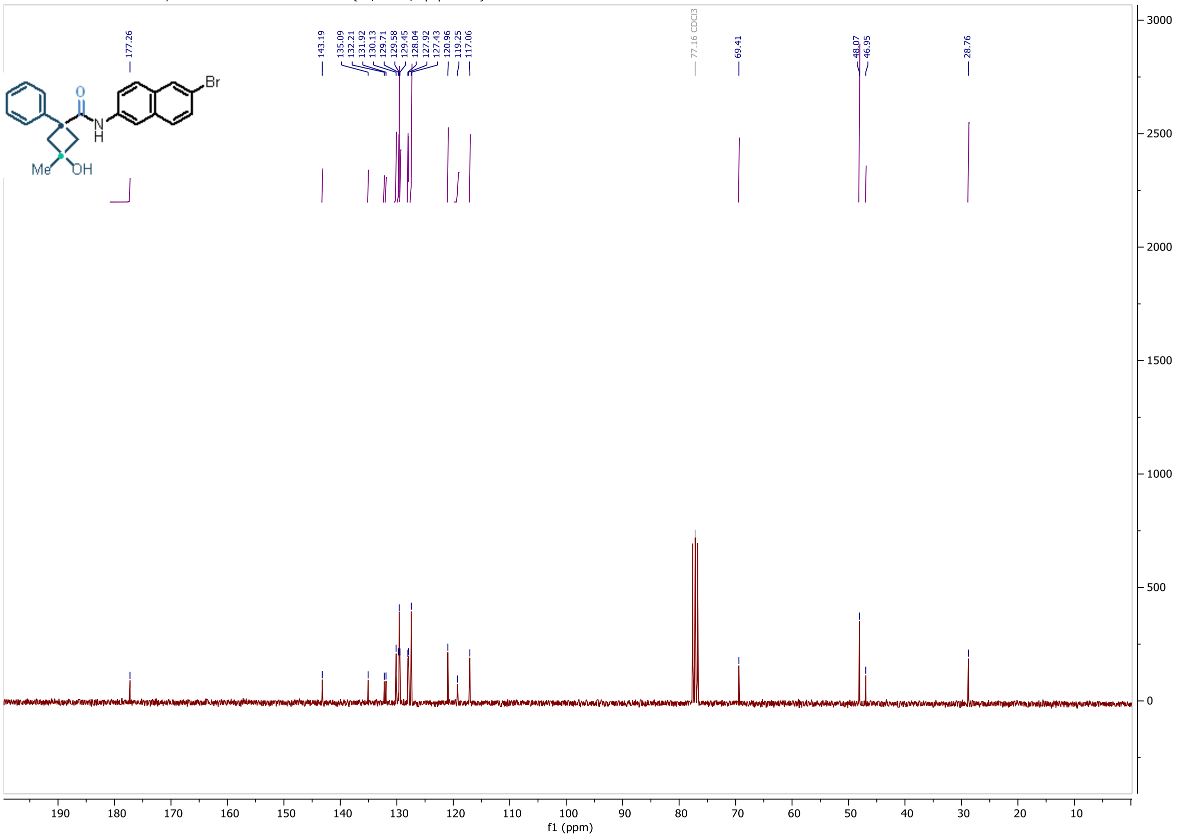

^{1}H NMR spectrum of **3y** (300 MHz, $CDCl_3$)

260317.320.10.fid — Yu-kun Liu LYK-24-100-re — Au1H CDCl3 {C:\Bruker\TopSpin3.6.2} 2603 20

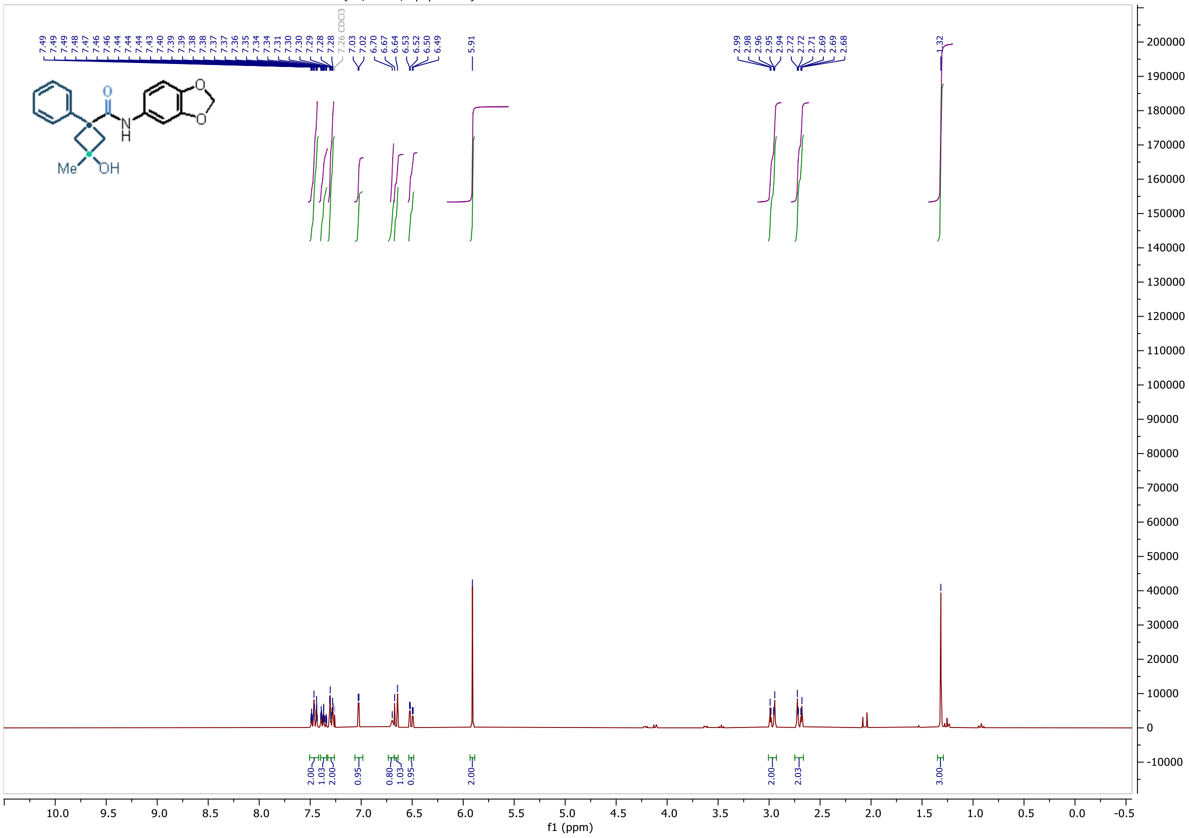

^{13}C NMR spectrum of **3y** (75 MHz, $CDCl_3$)

260317.320.11.fid — Yu-kun Liu LYK-24-100-re — Au13C CDCl3 {C:\Bruker\TopSpin3.6.2} 2603 20

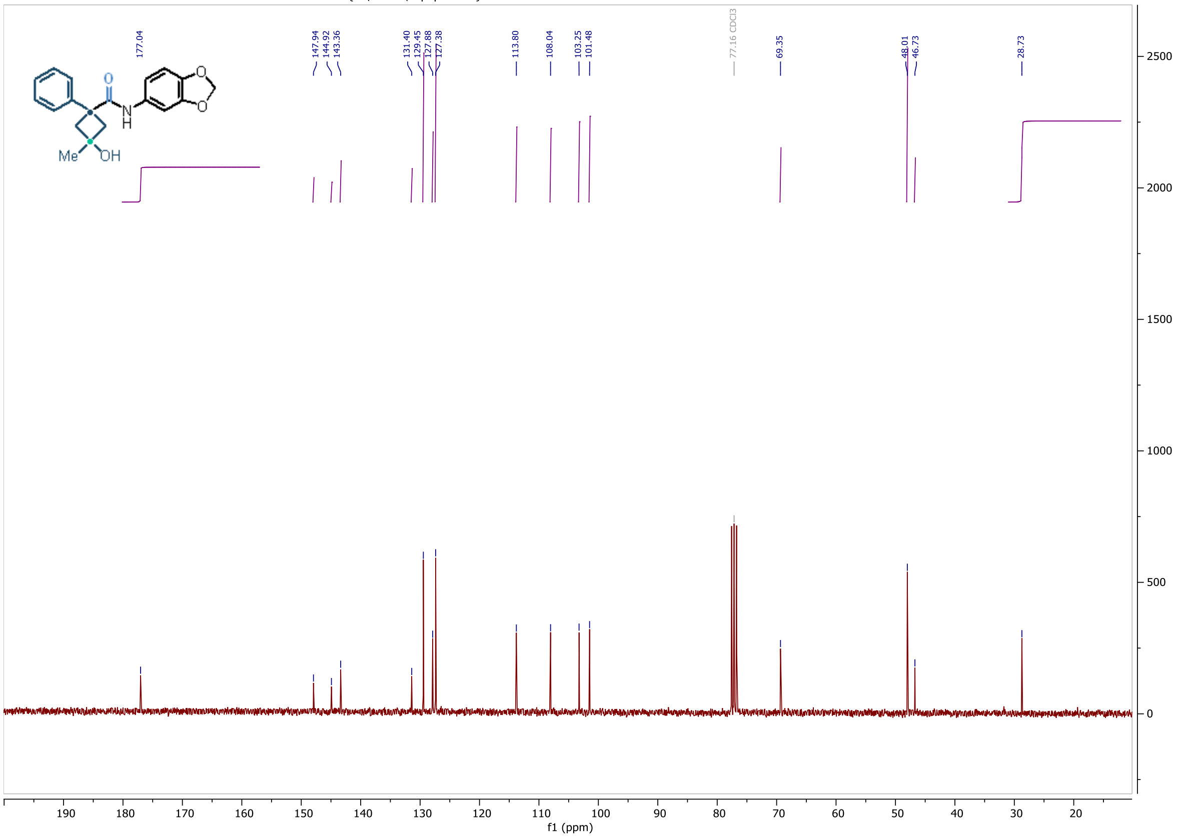

^{1}H NMR spectrum of **3z** (300 MHz, $CDCl_3$)

260312.328.10.fid — Yu-kun Liu LYK-24-96-re — Au1H CDCl3 {C:\Bruker\TopSpin3.6.2} 2603 28

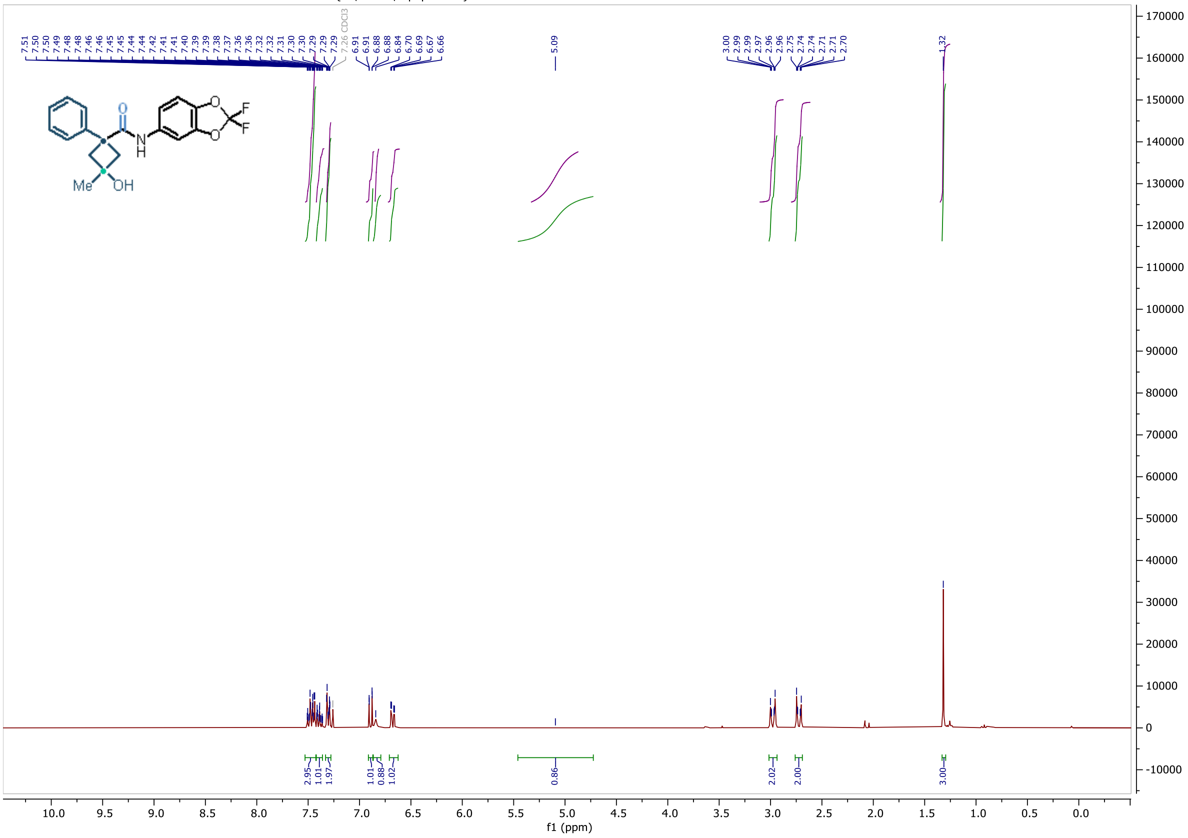

^{13}C NMR spectrum of **3z** (75 MHz, $CDCl_3$)

260312.328.11.fid — Yu-kun Liu LYK-24-96-re — Au13C CDCl3 {C:\Bruker\TopSpin3.6.2} 2603 28

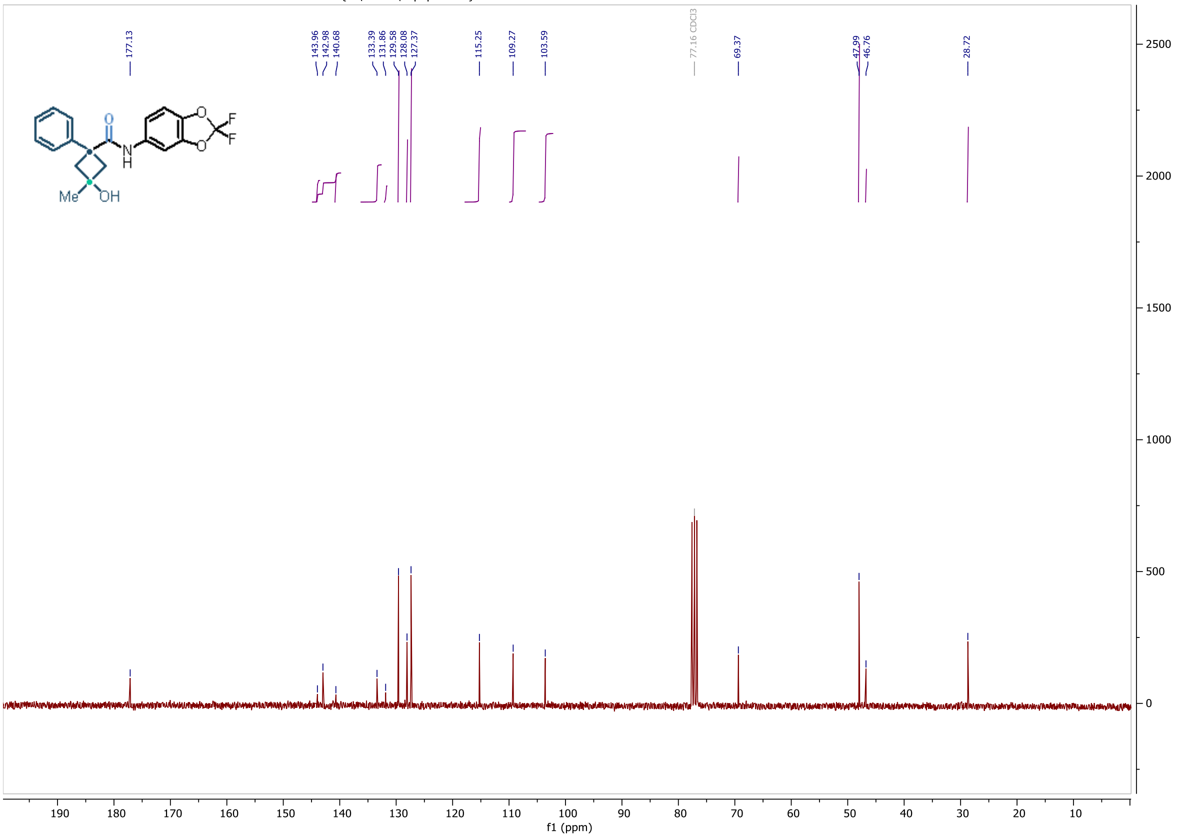

^{19}F NMR spectrum of **3z** (282 MHz, $CDCl_3$)

260312.328.12.fid — Yu-kun Liu LYK-24-96-re — Au19F CDCl3 {C:\Bruker\TopSpin3.6.2} 2603 28

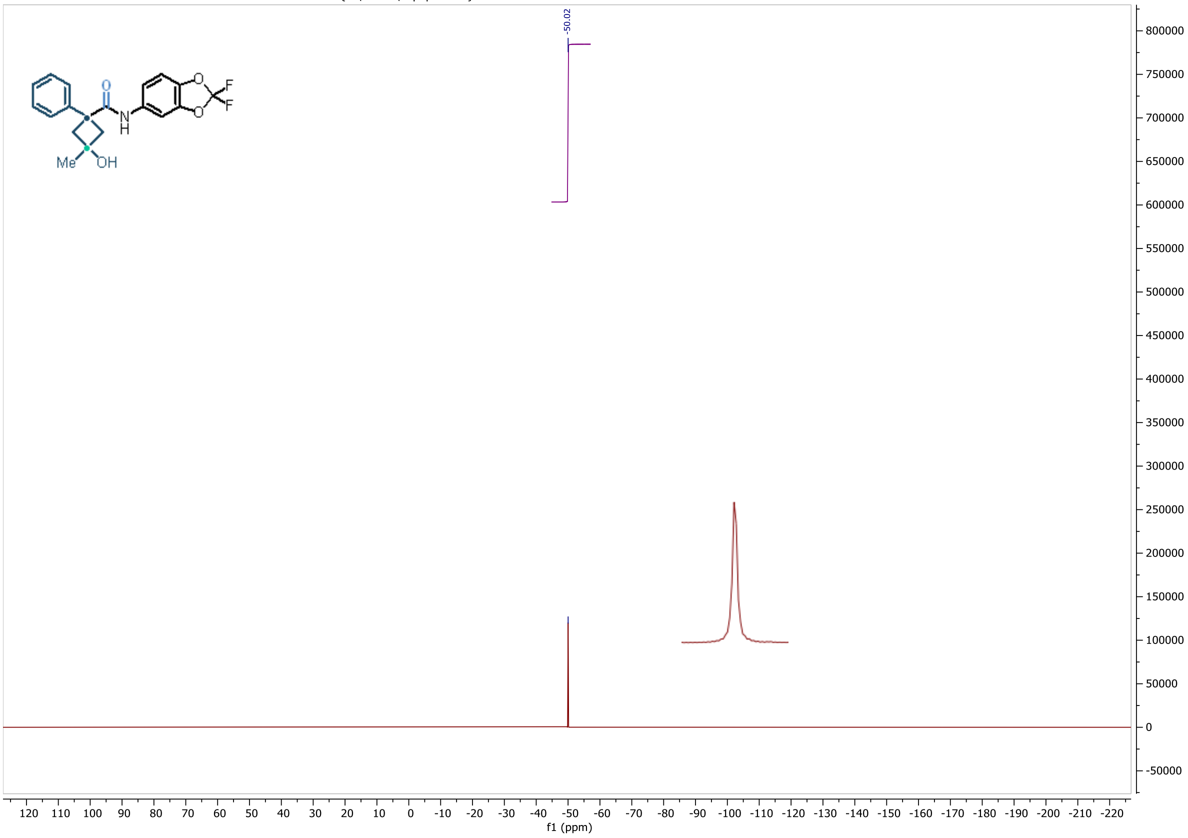

^{1}H NMR spectrum of **3ab** (300 MHz, $CDCl_3$)

260323.312.10.fid — Yu-kun Liu LYK-24-129-re — Au1H CDCl3 {C:\Bruker\TopSpin3.6.2} 2603 12

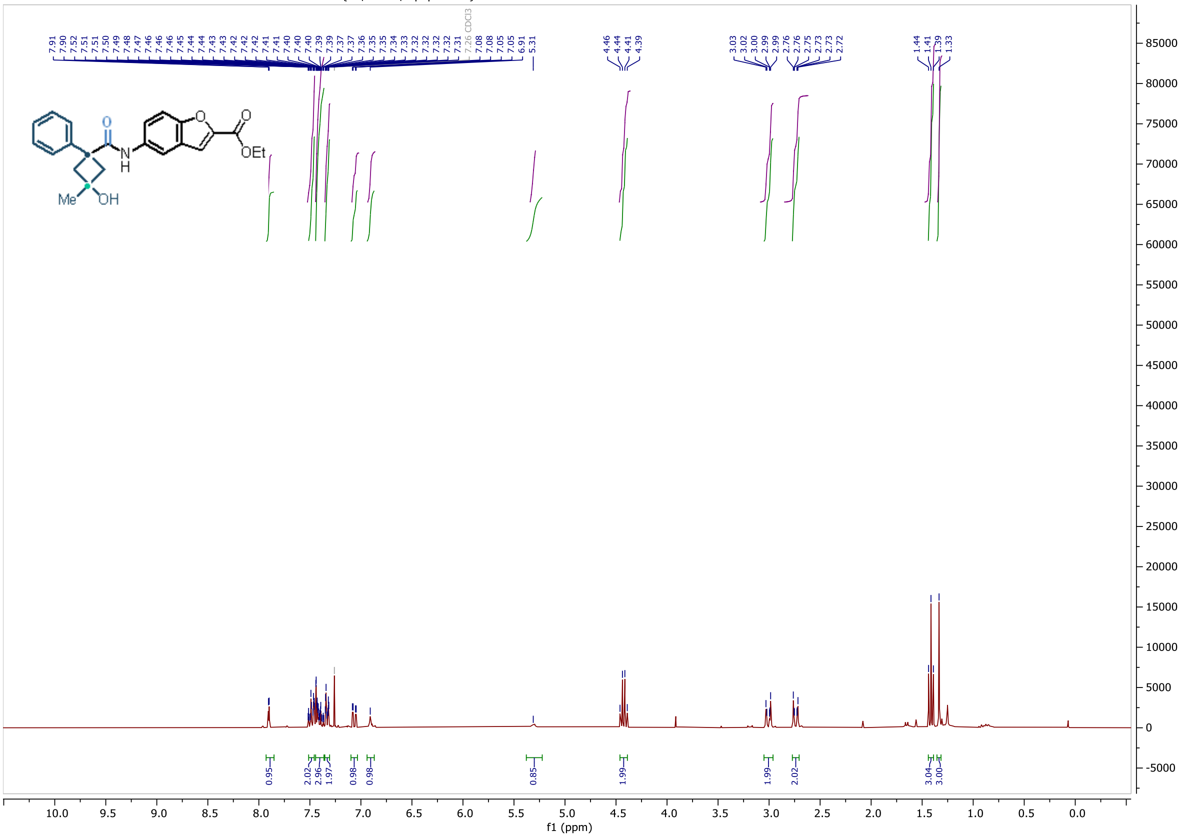

^{13}C NMR spectrum of **3ab** (75 MHz, $CDCl_3$)

260323.312.11.fid — Yu-kun Liu LYK-24-129-re — Au13C CDCl3 {C:\Bruker\TopSpin3.6.2} 2603 12

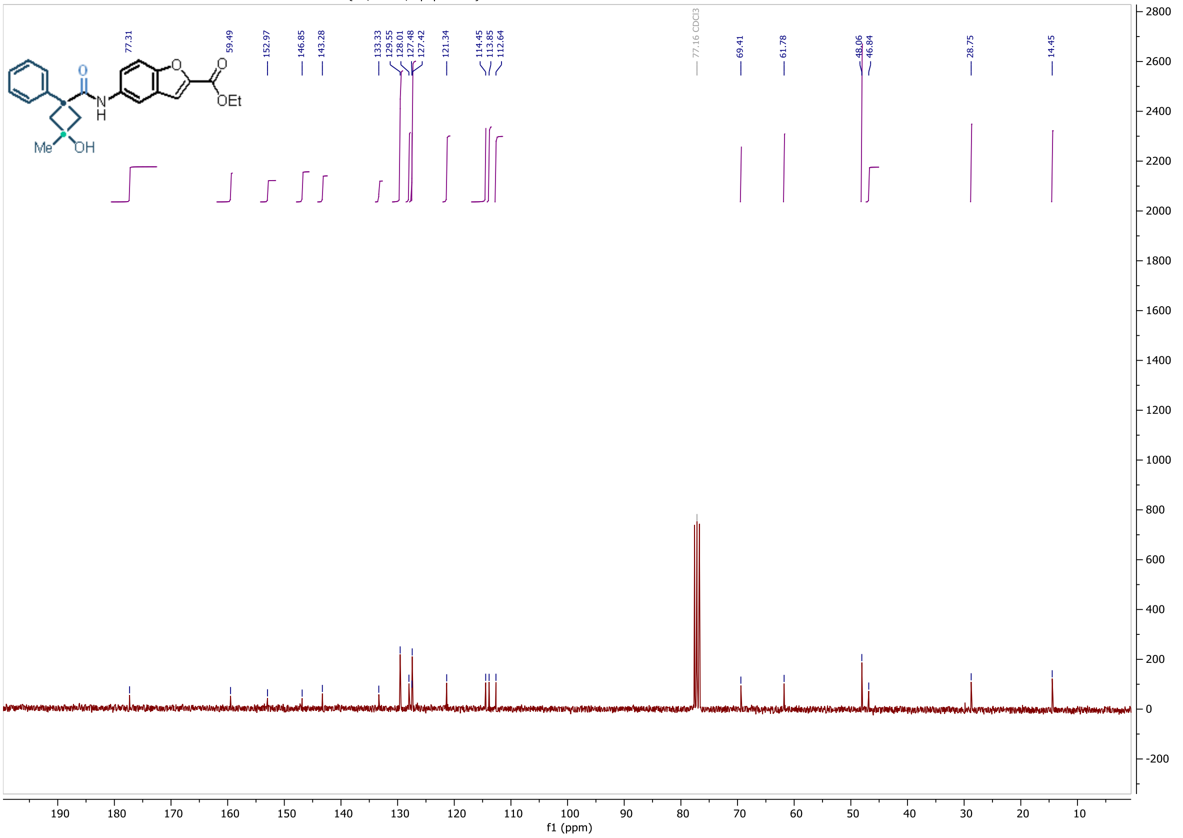

^{1}H NMR spectrum of **3ae** (300 MHz, $CDCl_3$)

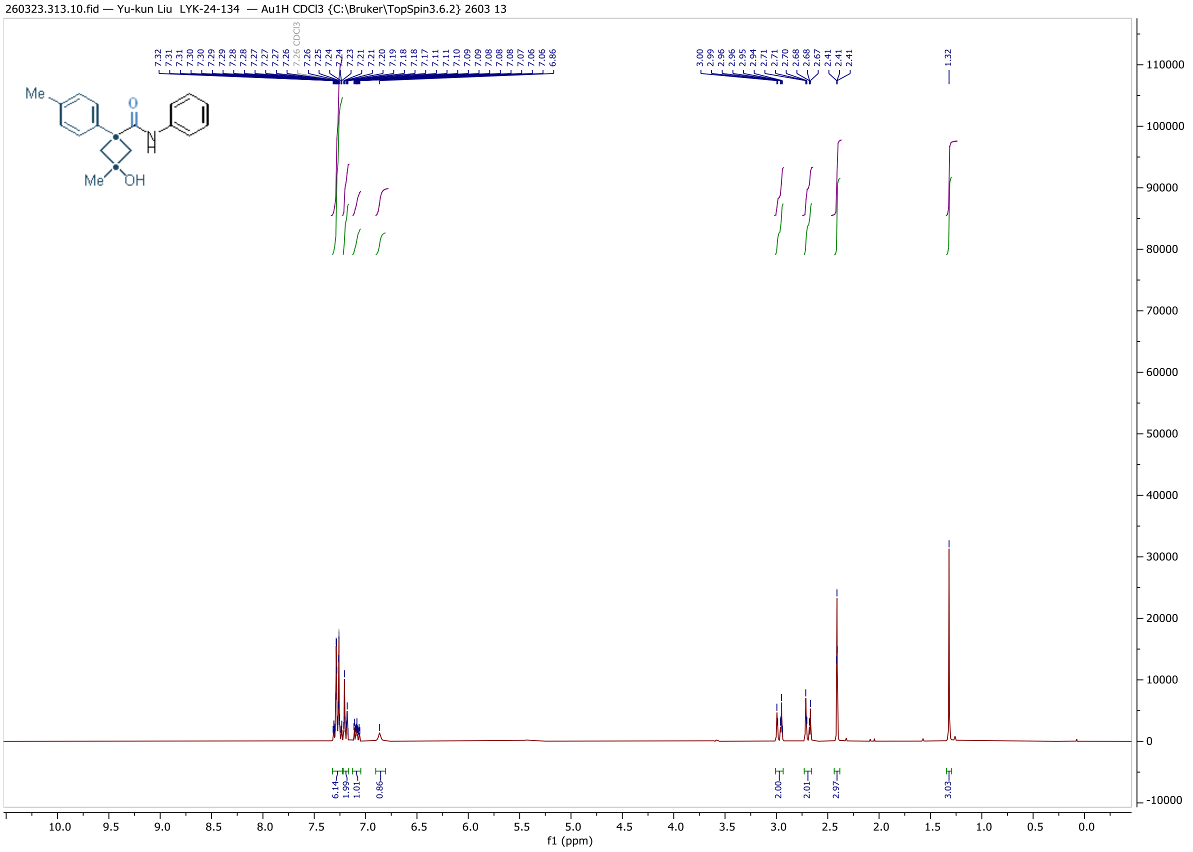

^{13}C NMR spectrum of **3ae** (75 MHz, $CDCl_3$)

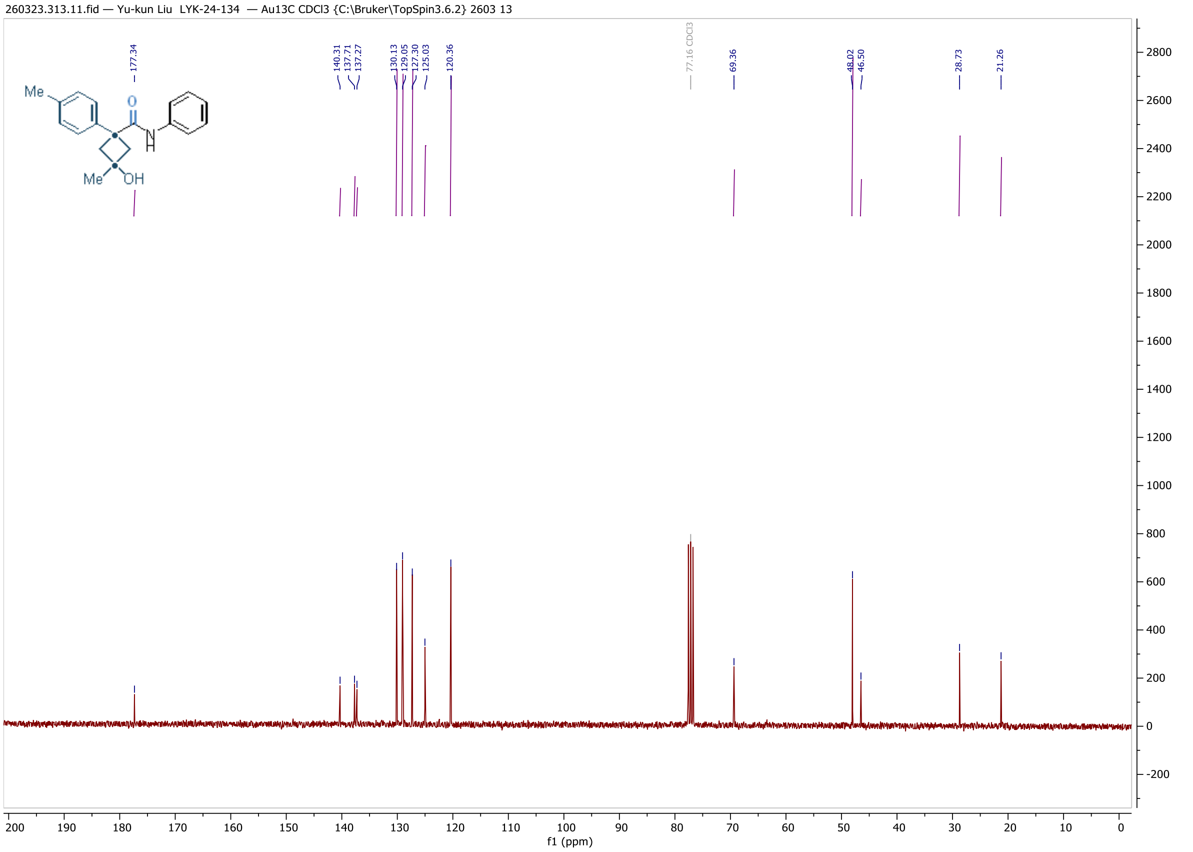

^{1}H NMR spectrum of **3af** (300 MHz, $CDCl_3$)

260407.305.10.fid — Yu-kun Liu LYK-24-162 — Au1H CDCl3 {C:\Bruker\TopSpin3.6.2} 2604 5

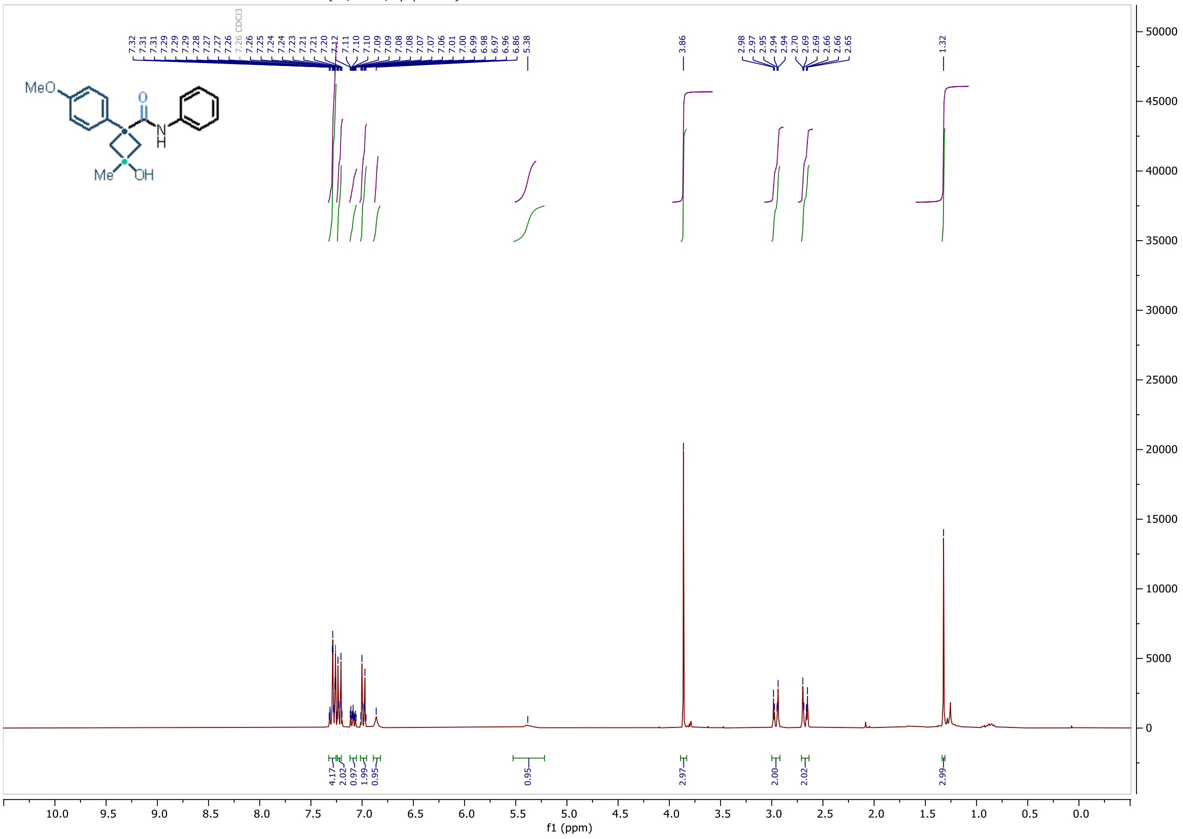

^{13}C NMR spectrum of **3af** (75 MHz, $CDCl_3$)

260407.305.11.fid — Yu-kun Liu LYK-24-162 — Au13C CDCl3 {C:\Bruker\TopSpin3.6.2} 2604 5

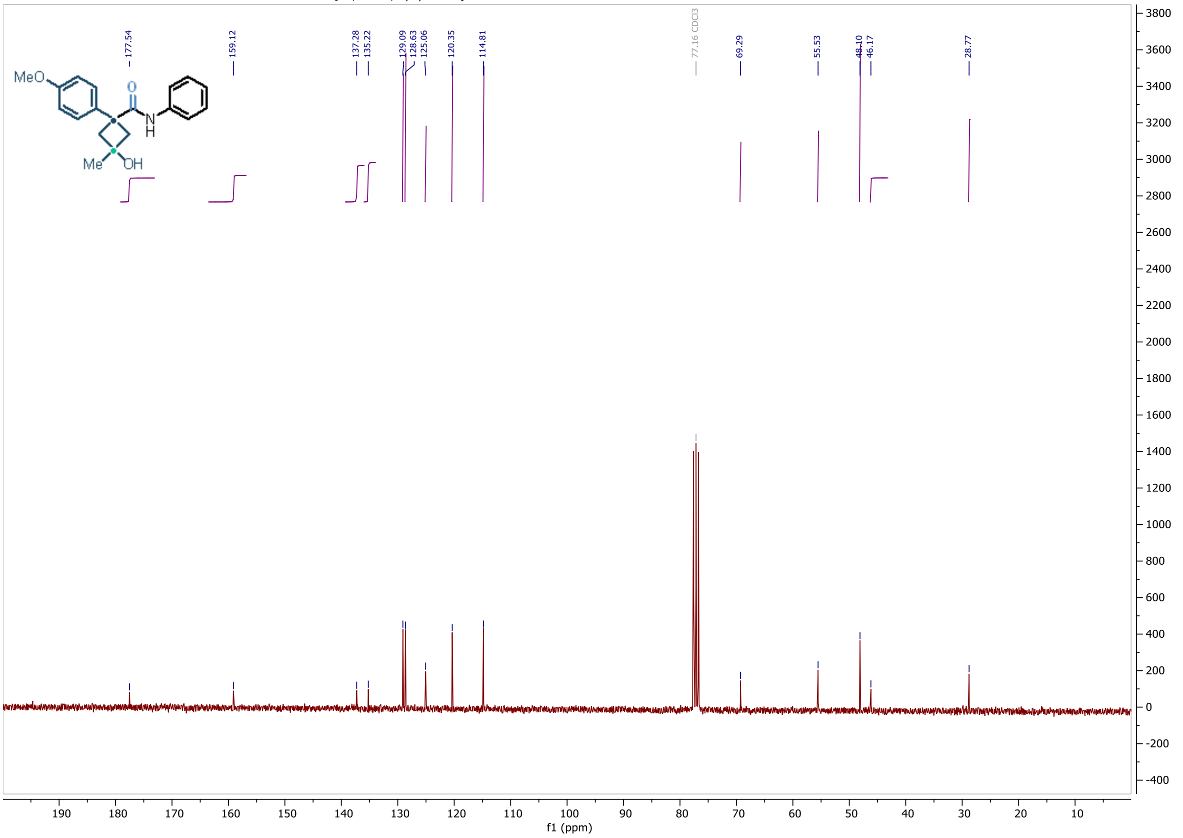

^{1}H NMR spectrum of **3ag** (300 MHz, $CDCl_3$)

260407.304.10.fid — Yu-kun Liu LYK-24-161 — Au1H CDCl3 {C:\Bruker\TopSpin3.6.2} 2604 4

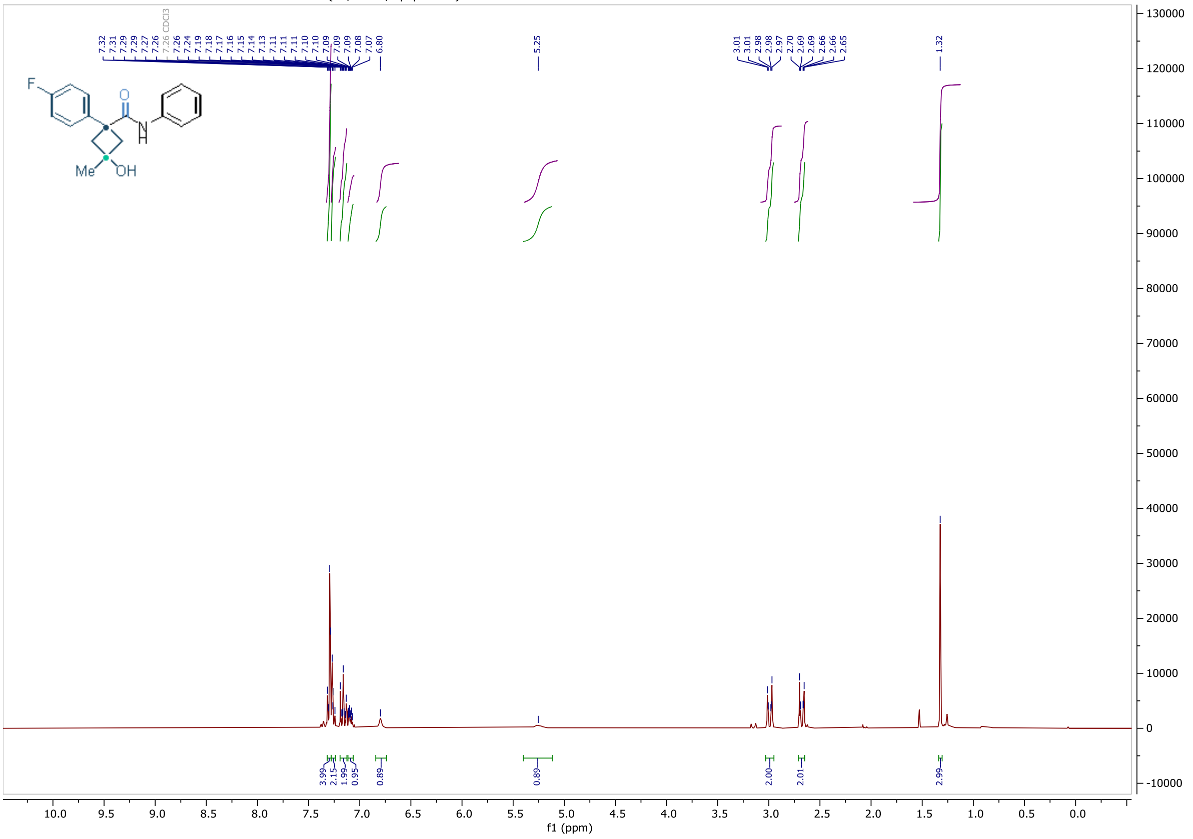

^{13}C NMR spectrum of **3ag** (75 MHz, $CDCl_3$)

260407.304.11.fid — Yu-kun Liu LYK-24-161 — Au13C CDCl3 {C:\Bruker\TopSpin3.6.2} 2604 4

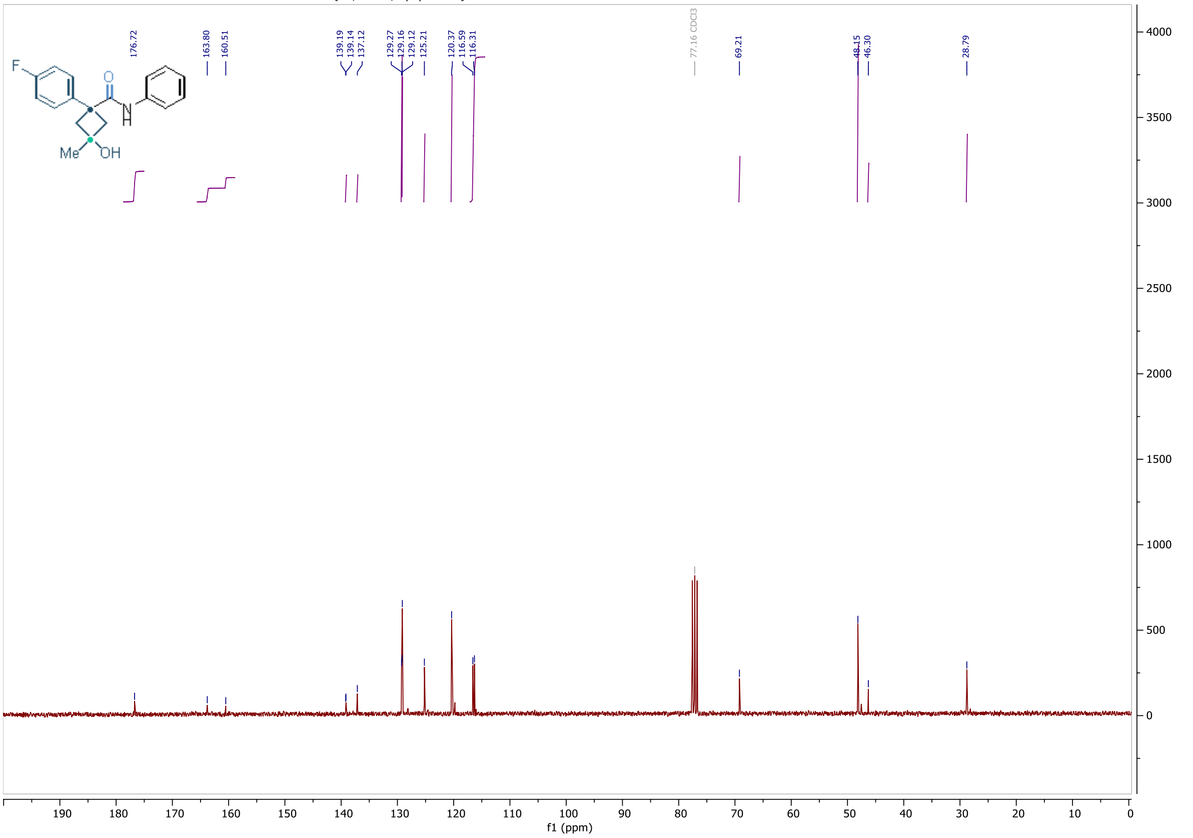

^{19}F NMR spectrum of **3ah** (282 MHz, $CDCl_3$)

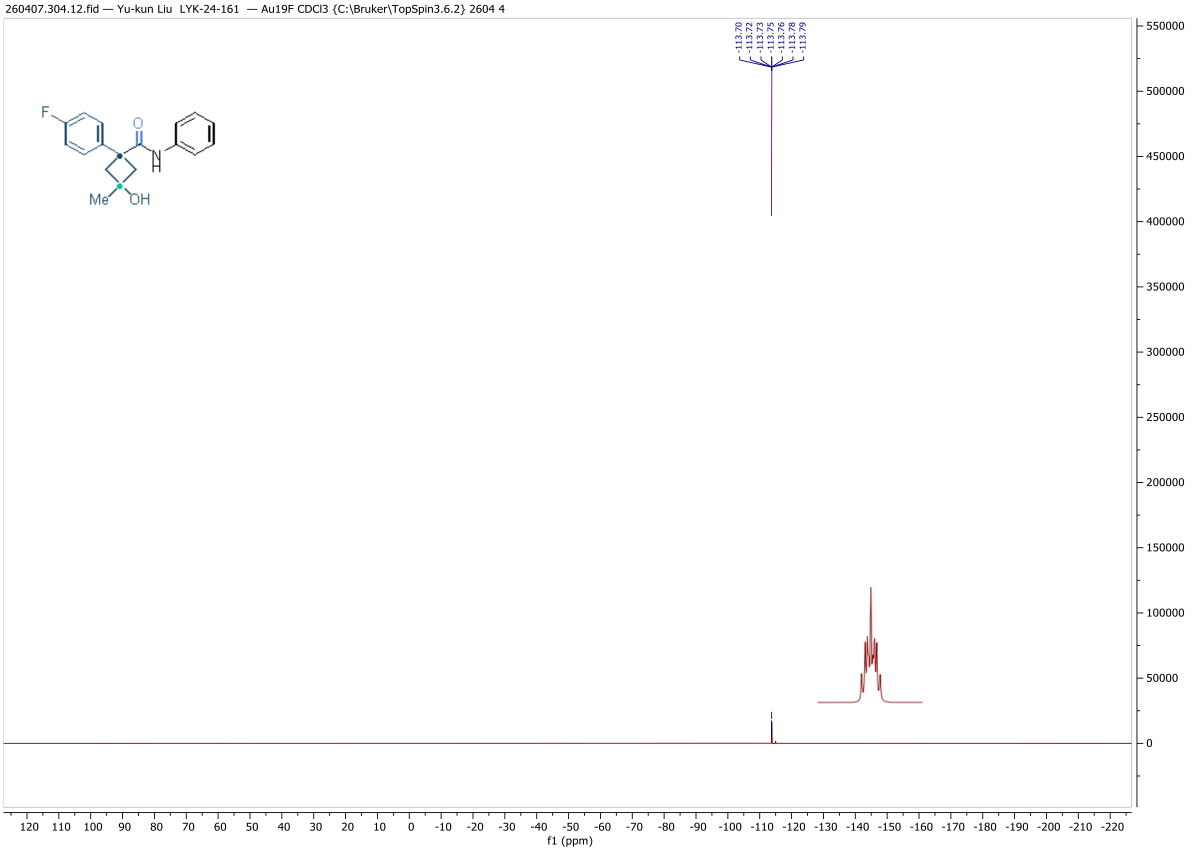

^{1}H NMR spectrum of **3ah** (300 MHz, $CDCl_3$)

260413.307.10.fid — Yu-kun Liu LYK-24-164 — Au1H CDCl3 {C:\Bruker\TopSpin3.6.2} 2604 7

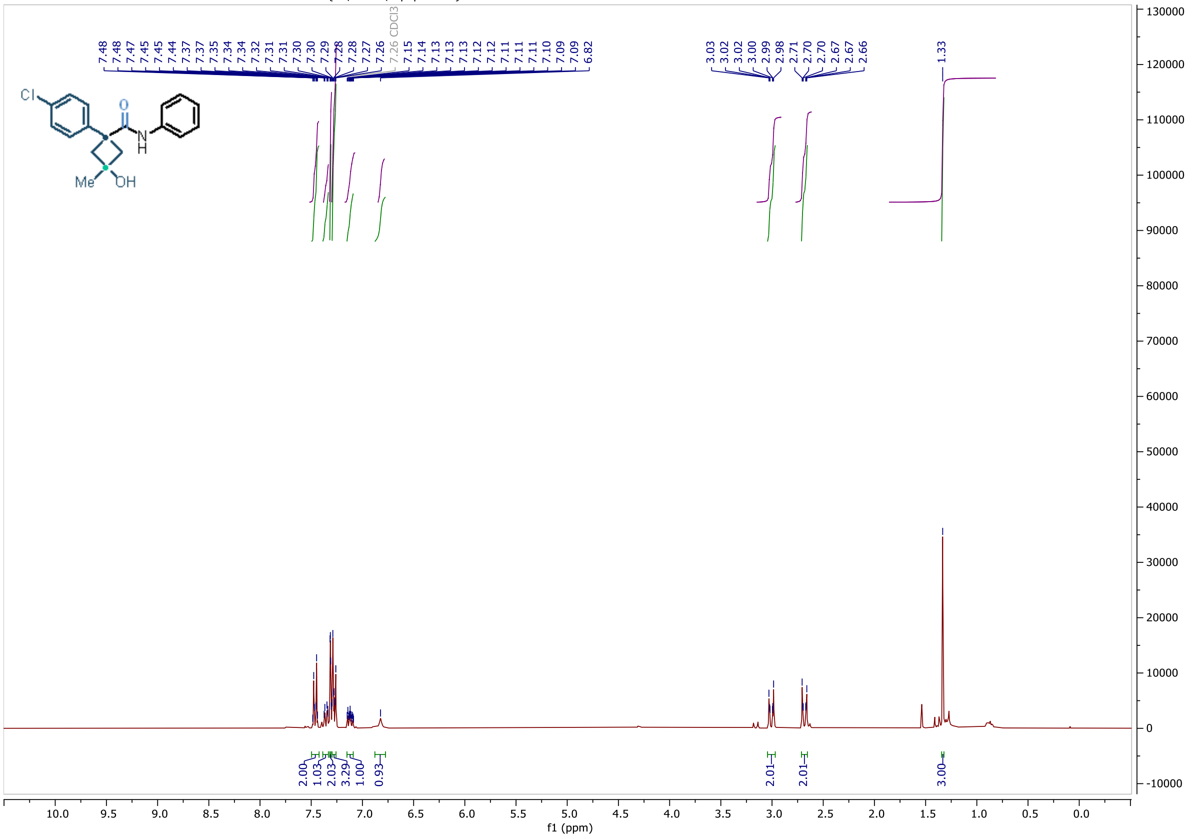

^{13}C NMR spectrum of **3ah** (75 MHz, $CDCl_3$)

260413.307.11.fid — Yu-kun Liu LYK-24-164 — Au13C CDCl3 {C:\Bruker\TopSpin3.6.2} 2604 7

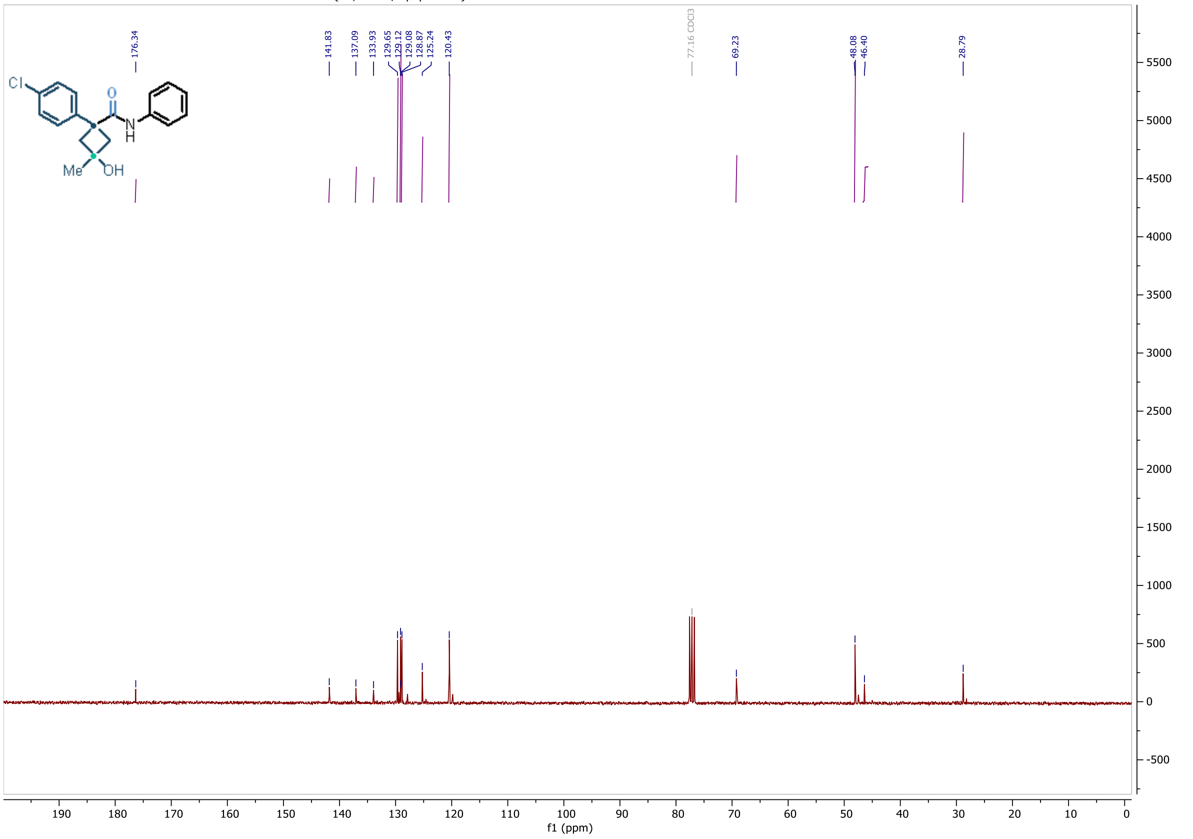

^{1}H NMR spectrum of **3ai** (300 MHz, $CDCl_3$)

260415.f322.10.fid — Yu-kun Liu LYK-24-165-re — Au1H CDCl3 {C:\Bruker\TopSpin3.6.2} 2604 22

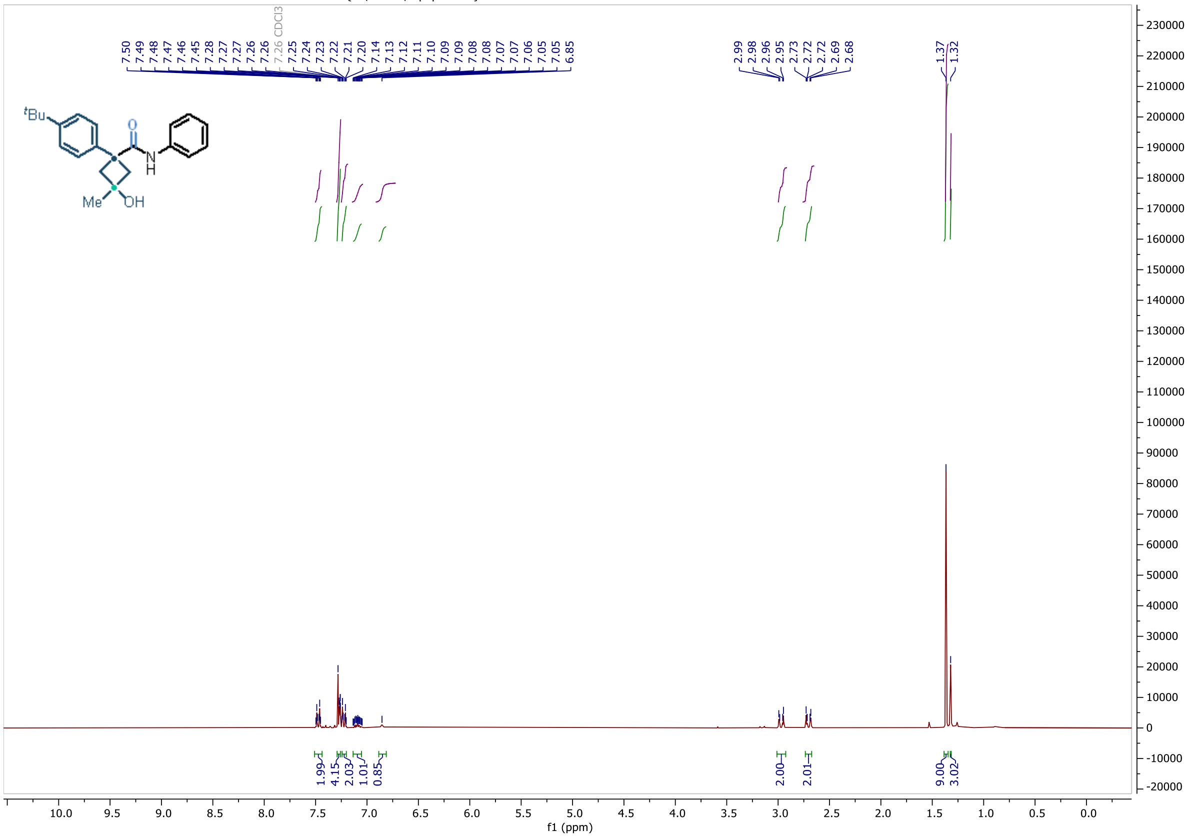

^{13}C NMR spectrum of **3ai** (75 MHz, $CDCl_3$)

260415.f322.11.fid — Yu-kun Liu LYK-24-165-re — Au13C CDCl3 {C:\Bruker\TopSpin3.6.2} 2604 22

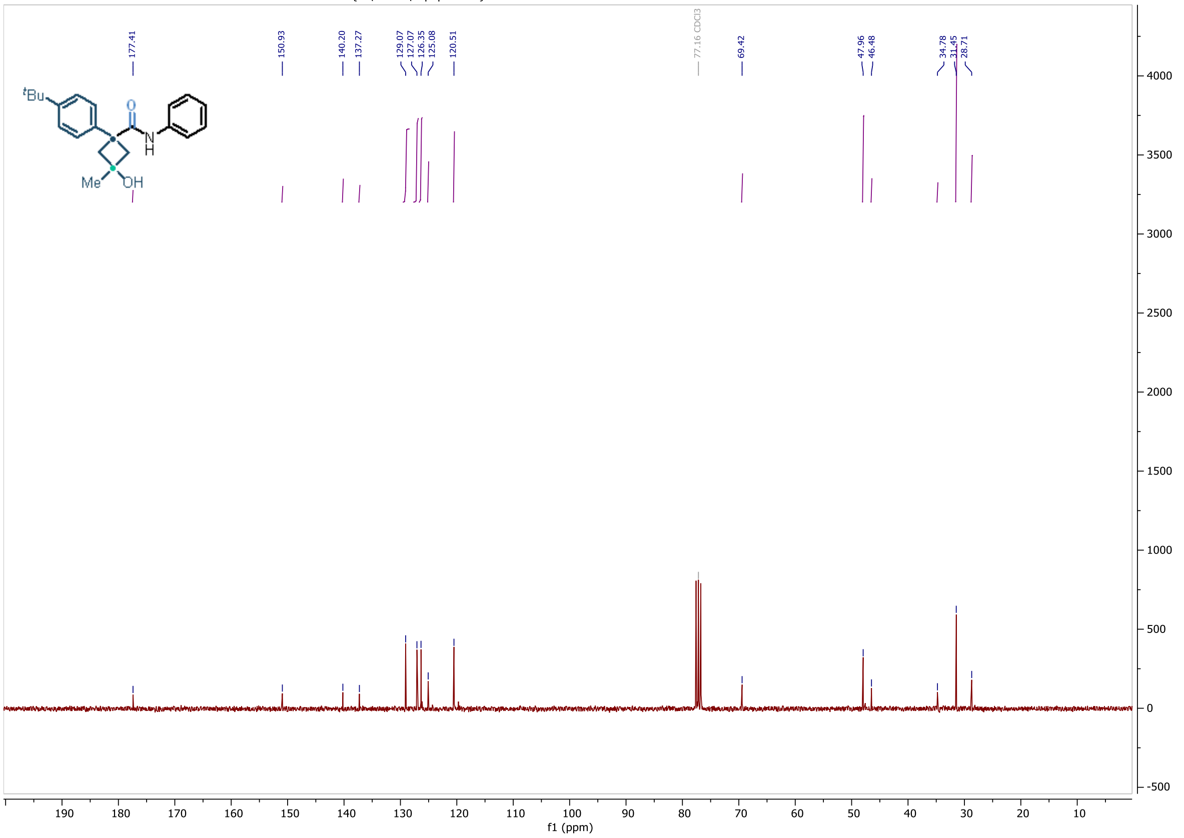

^{1}H NMR spectrum of **3aj** (300 MHz, $CDCl_3$)

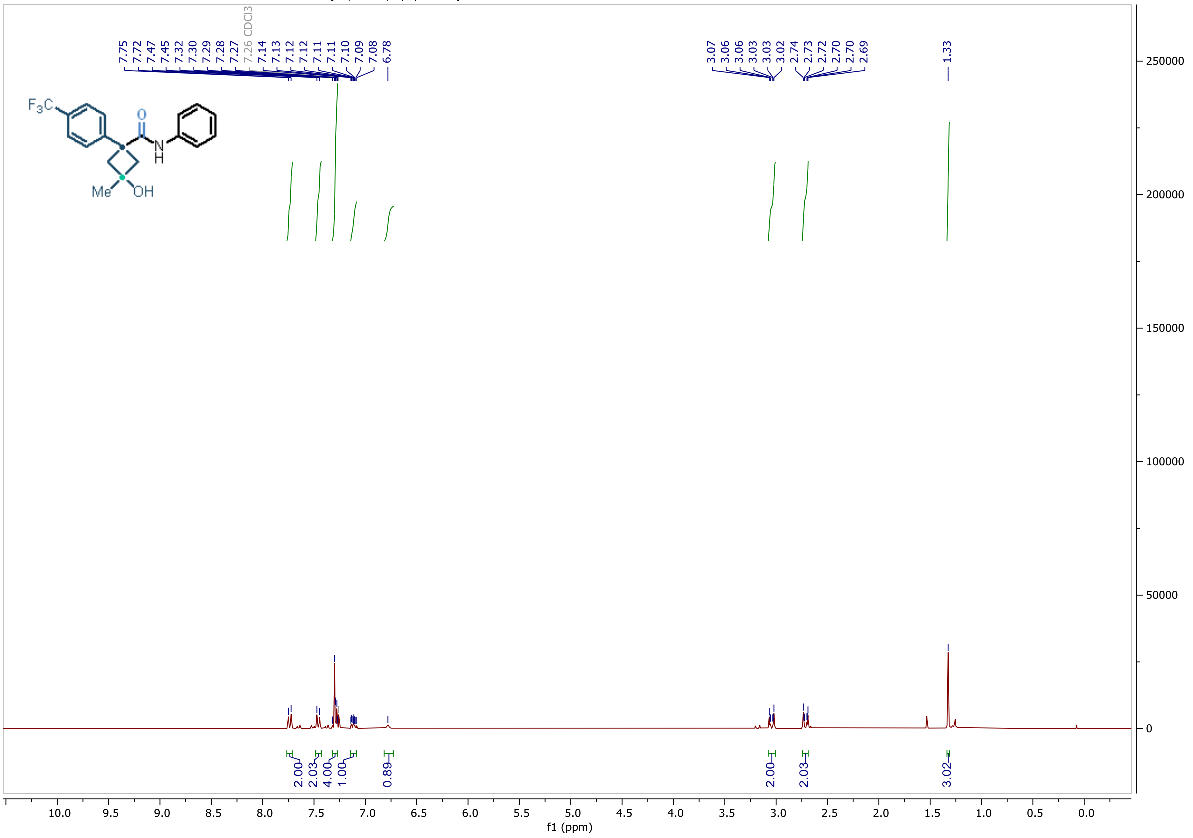

^{13}C NMR spectrum of **3aj** (75 MHz, $CDCl_3$)

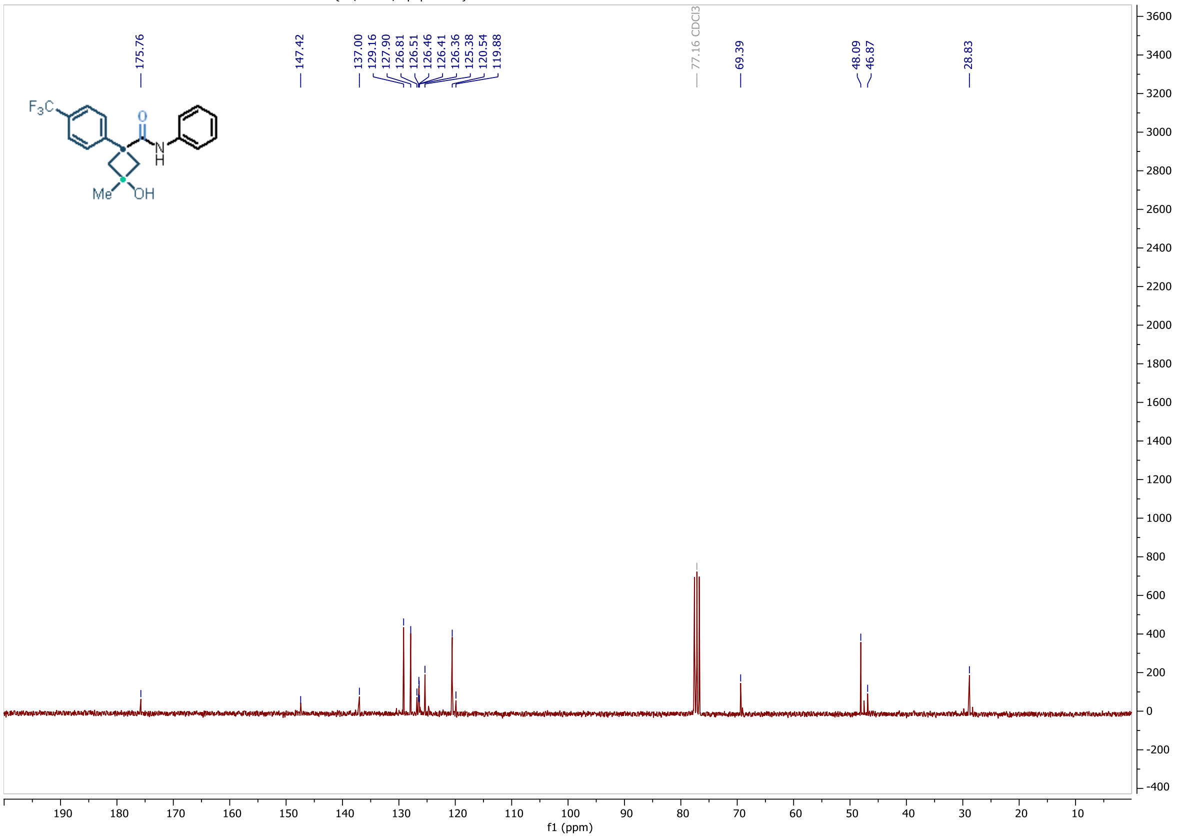

^{19}F NMR spectrum of **3aj** (282 MHz, $CDCl_3$)

260424.315.12.fid — Yu-Kun Liu LYK-24-173 — Au19F CDCl3 {C:\Bruker\TopSpin3.6.2} 2604 15

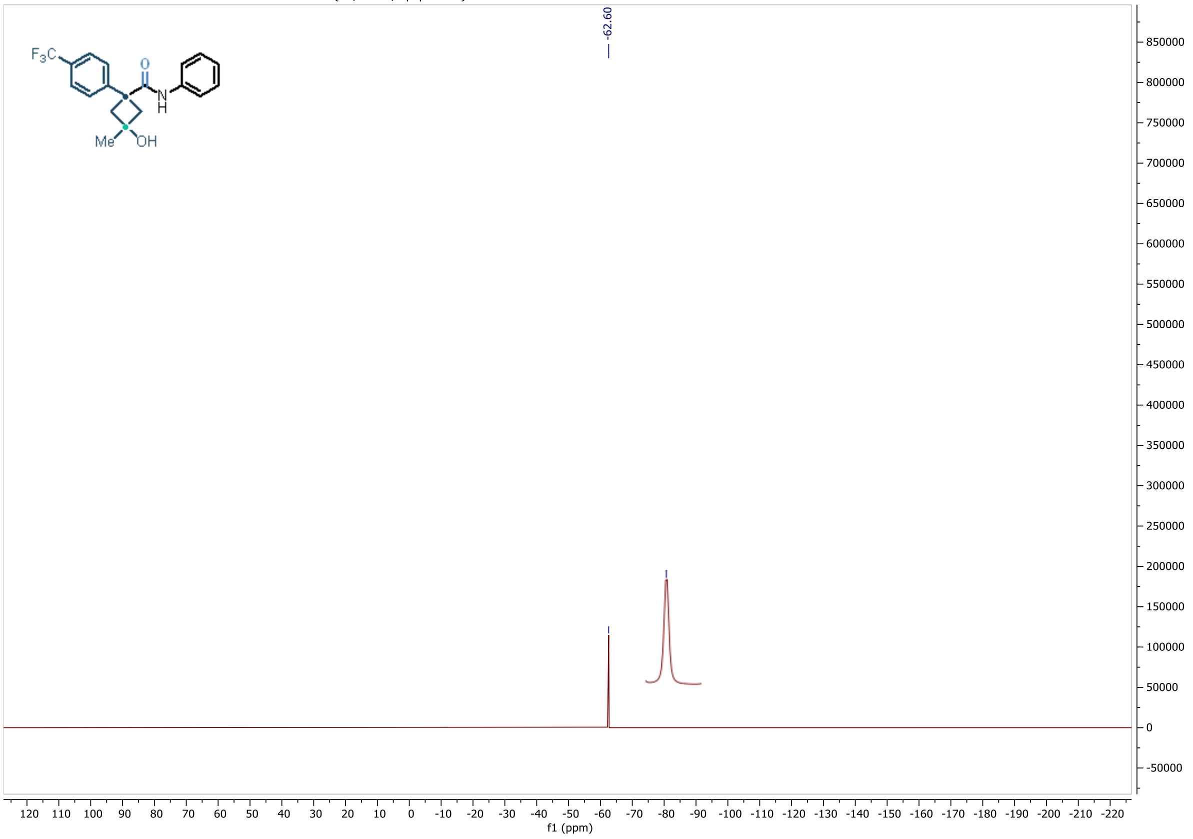

^{1}H NMR spectrum of **3ak** (300 MHz, $CDCl_3$)

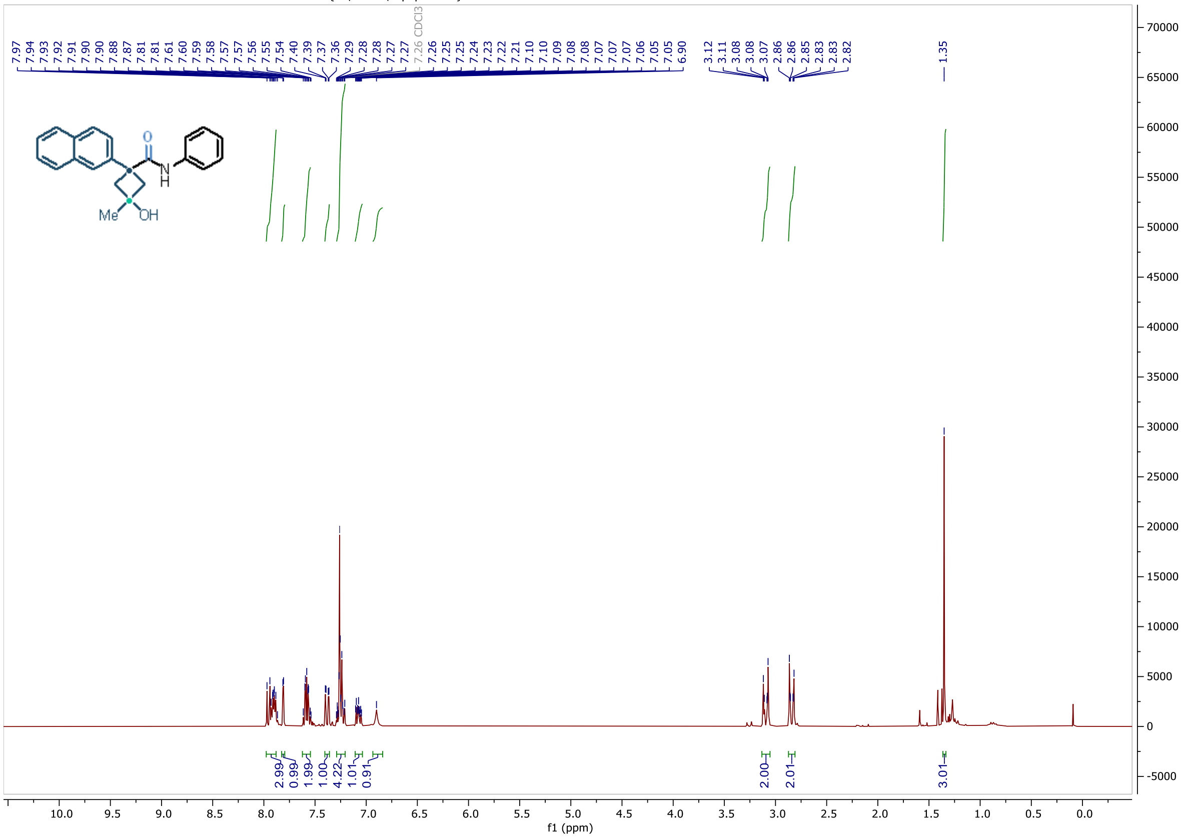

^{13}C NMR spectrum of **3ak** (75 MHz, $CDCl_3$)

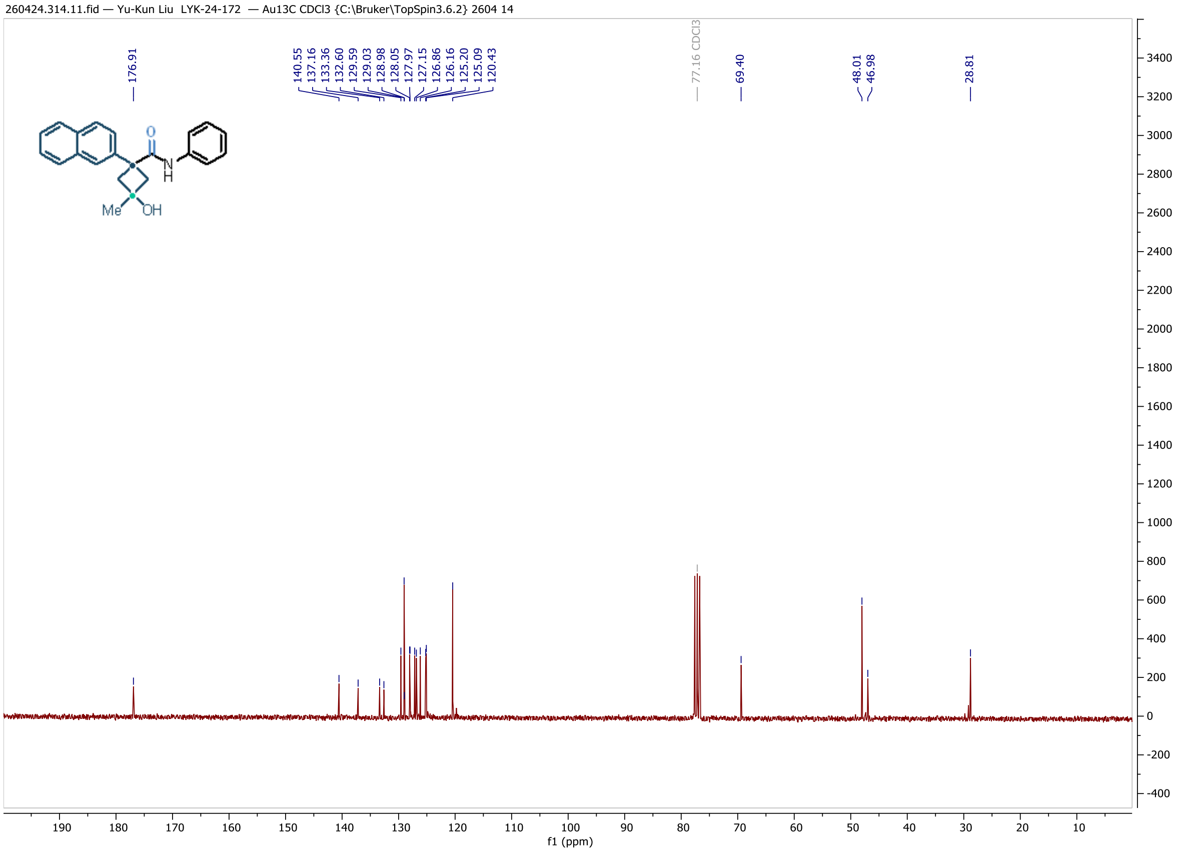

^{1}H NMR spectrum of **3al** (300 MHz, $CDCl_3$)

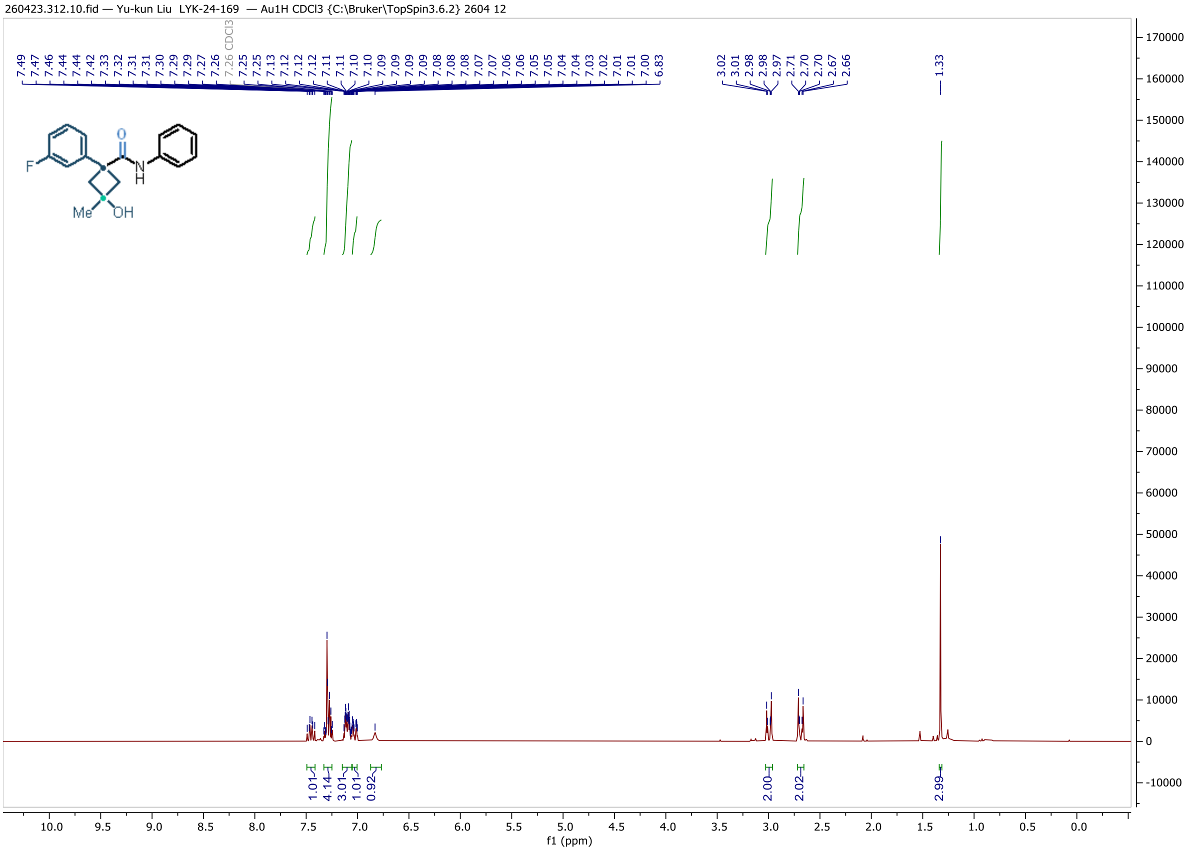

^{13}C NMR spectrum of **3al** (75 MHz, $CDCl_3$)

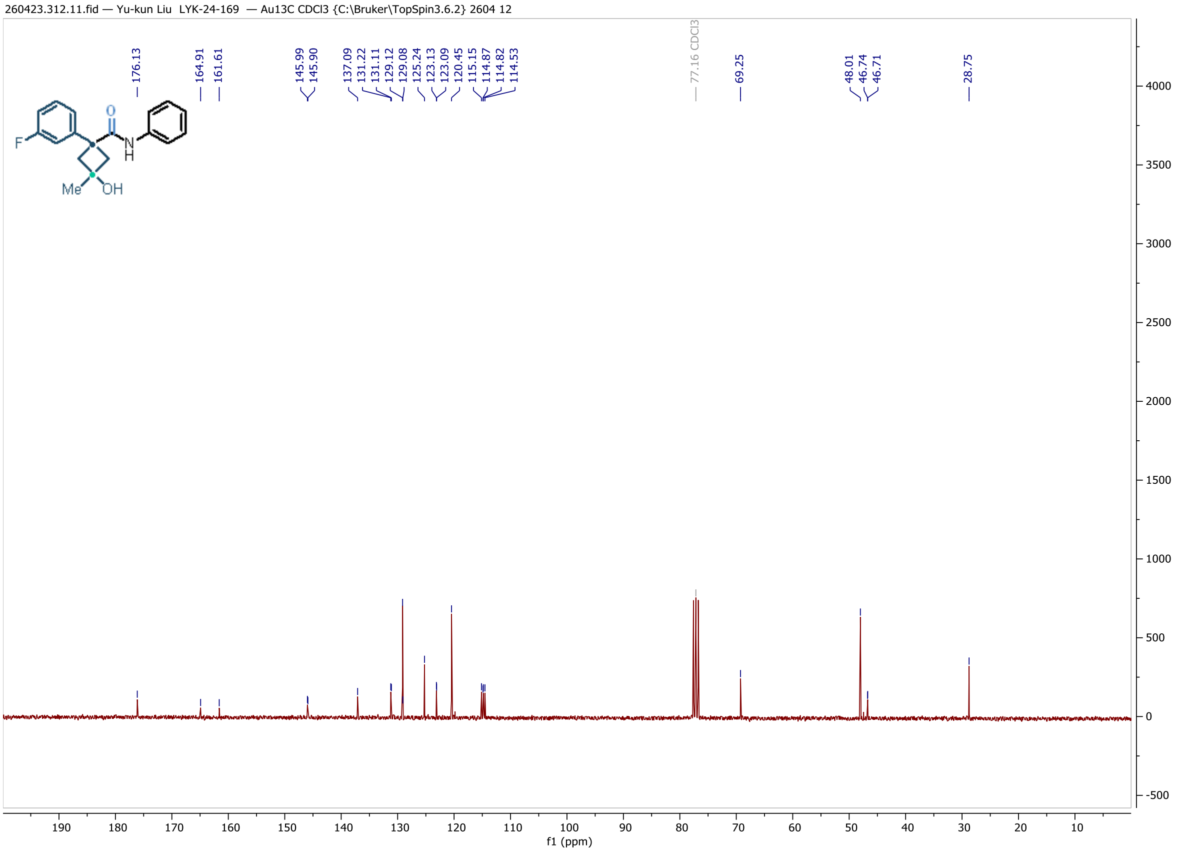

^{19}F NMR spectrum of **3al** (282 MHz, $CDCl_3$)

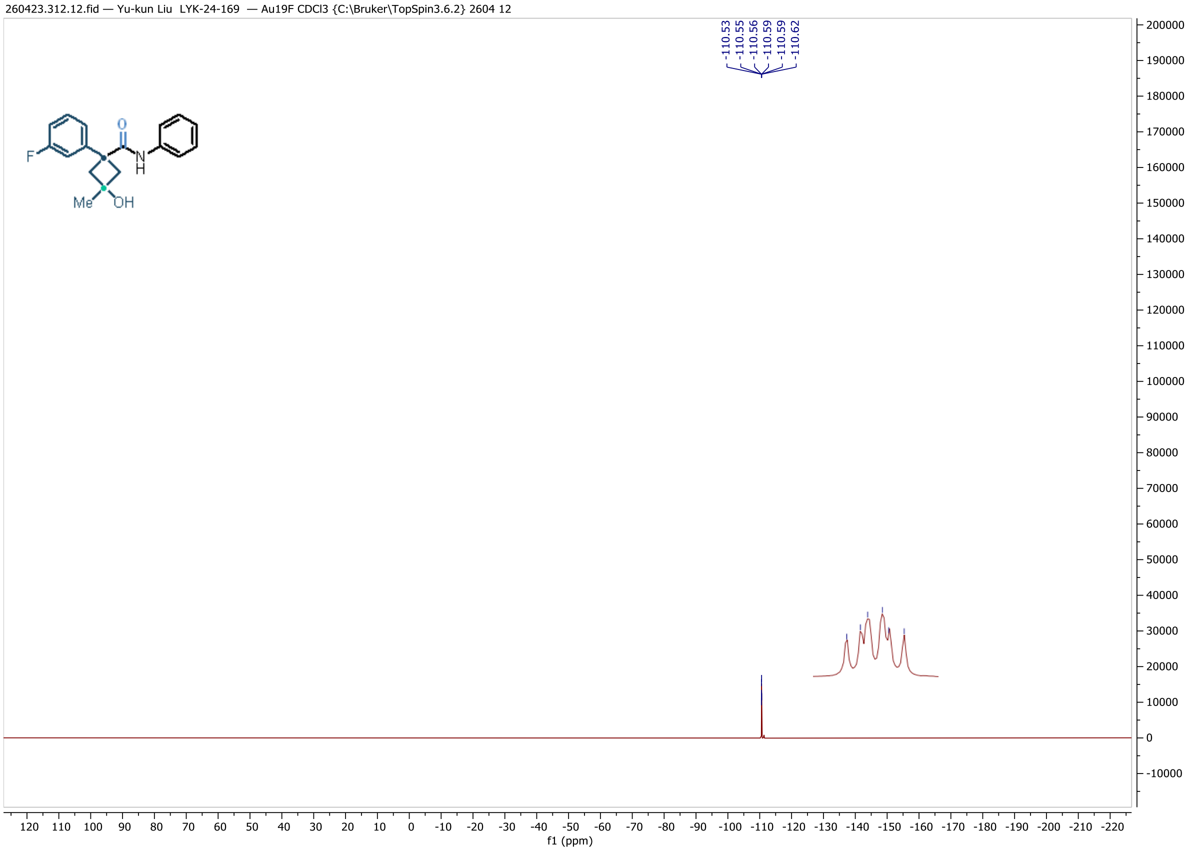

^{1}H NMR spectrum of **3am** (300 MHz, $CDCl_3$)

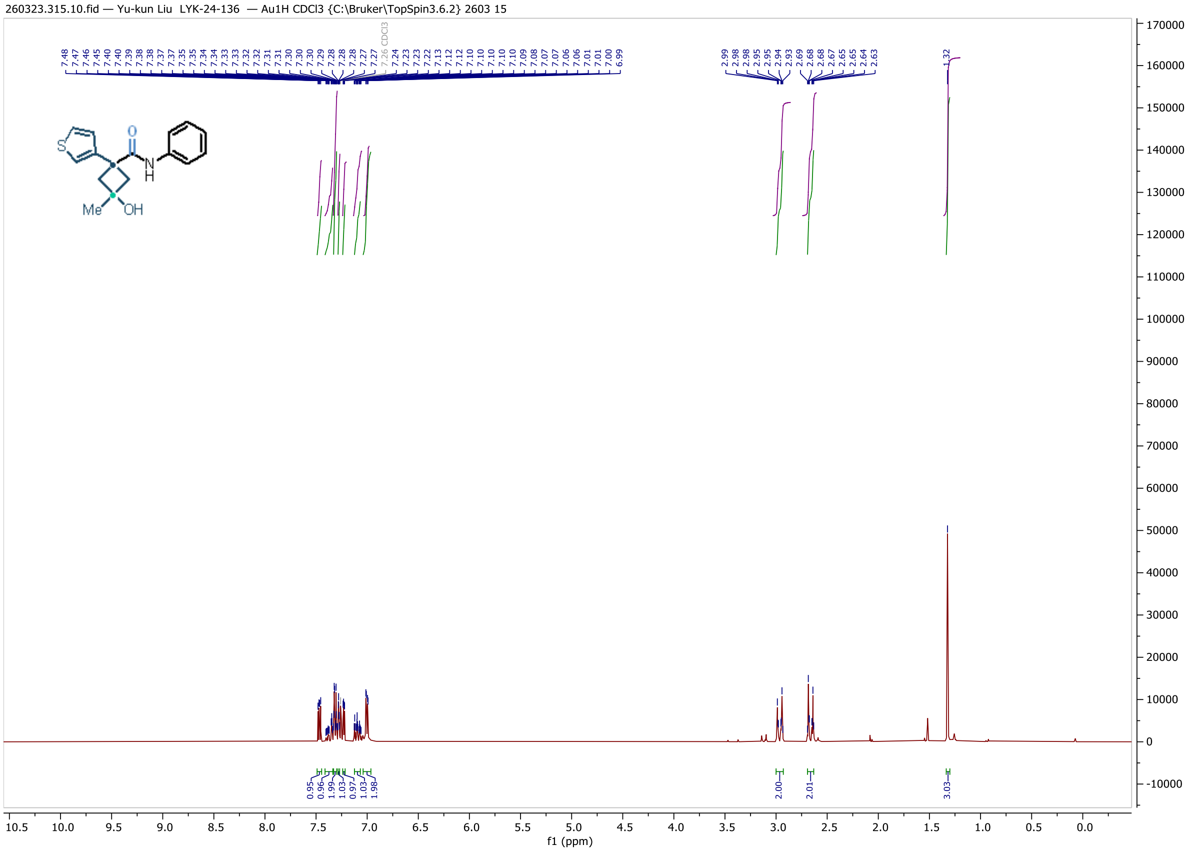

^{13}C NMR spectrum of **3am** (75 MHz, $CDCl_3$)

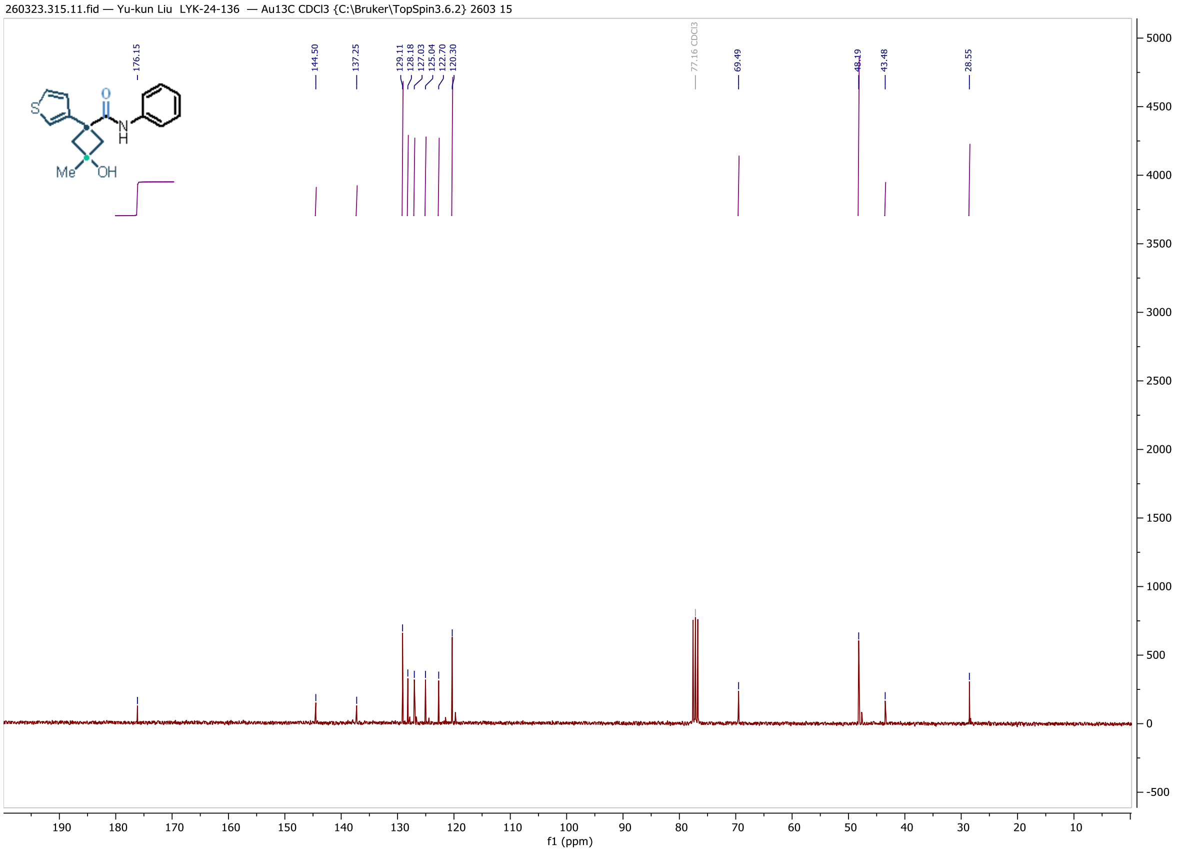

^{1}H NMR spectrum of **3an** (300 MHz, $CDCl_3$)

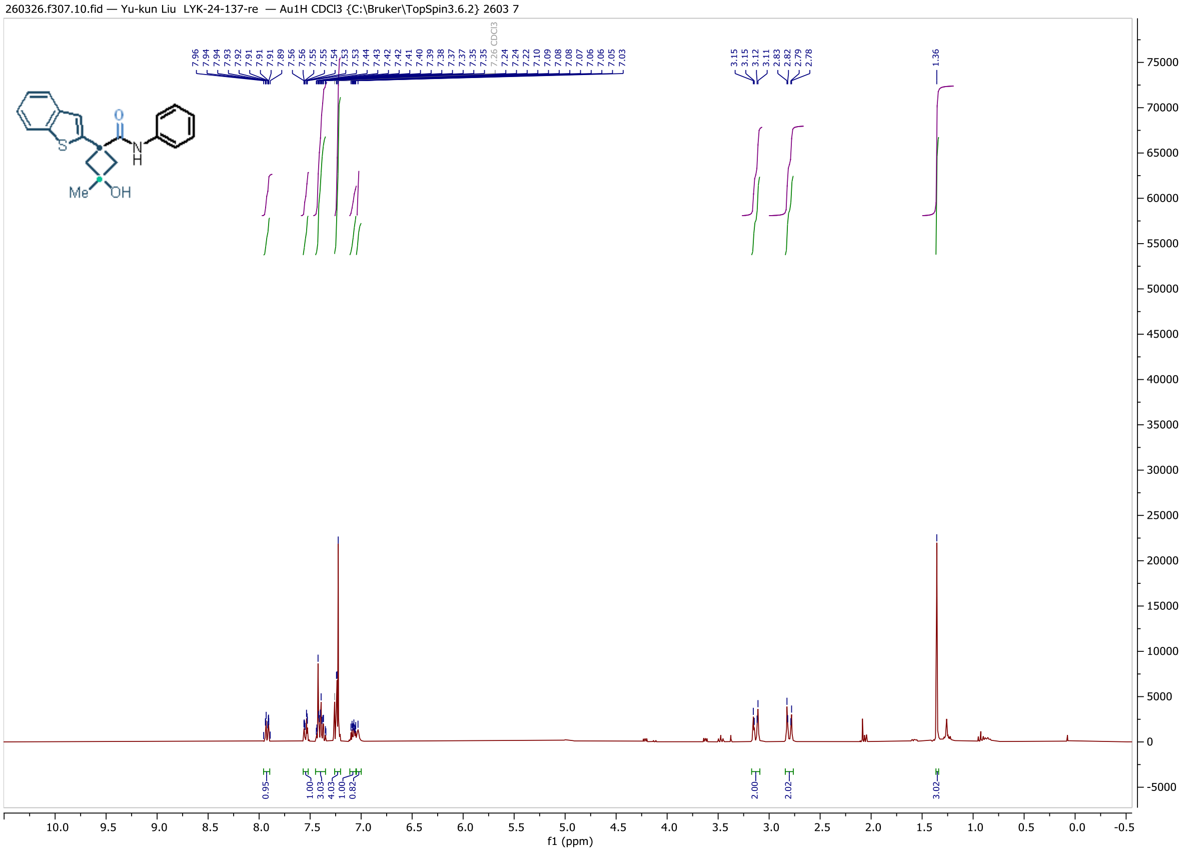

^{13}C NMR spectrum of **3an** (75 MHz, $CDCl_3$)

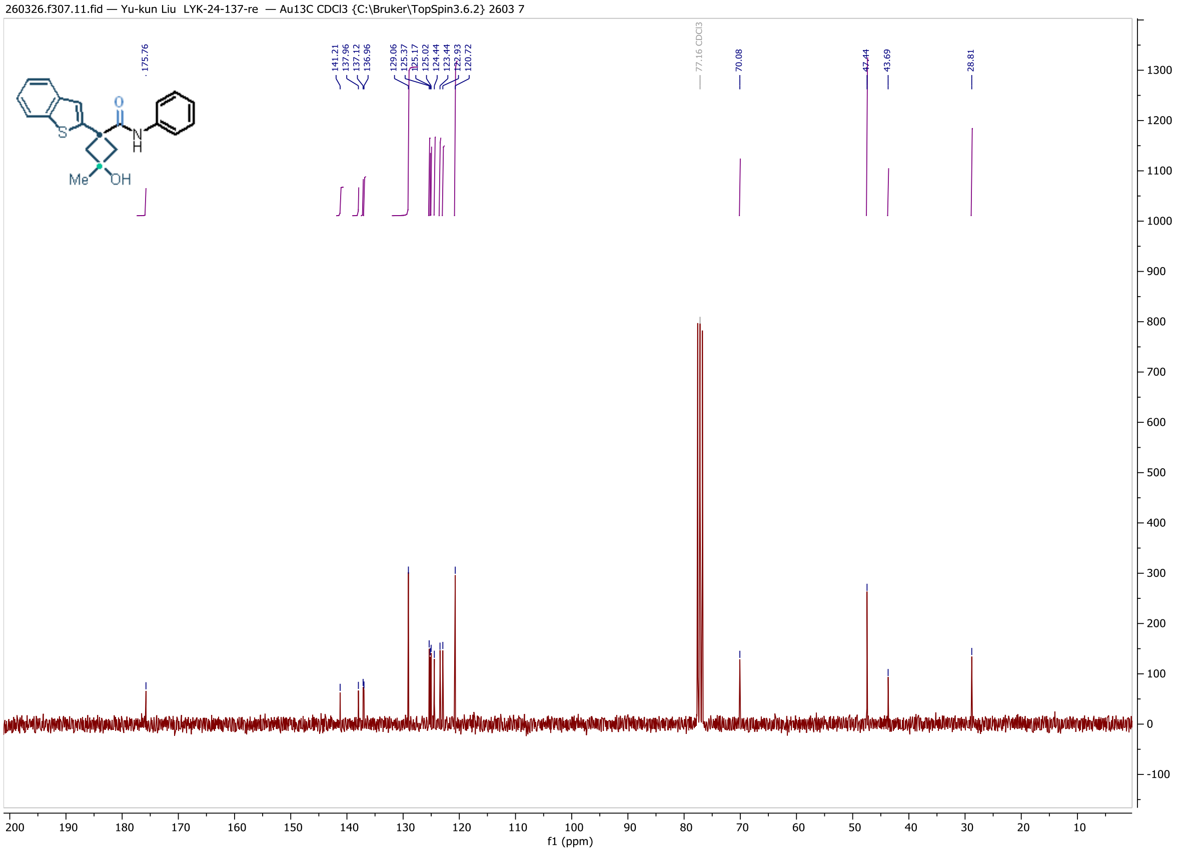

^{1}H NMR spectrum of **3ao** (300 MHz, $CDCl_3$)

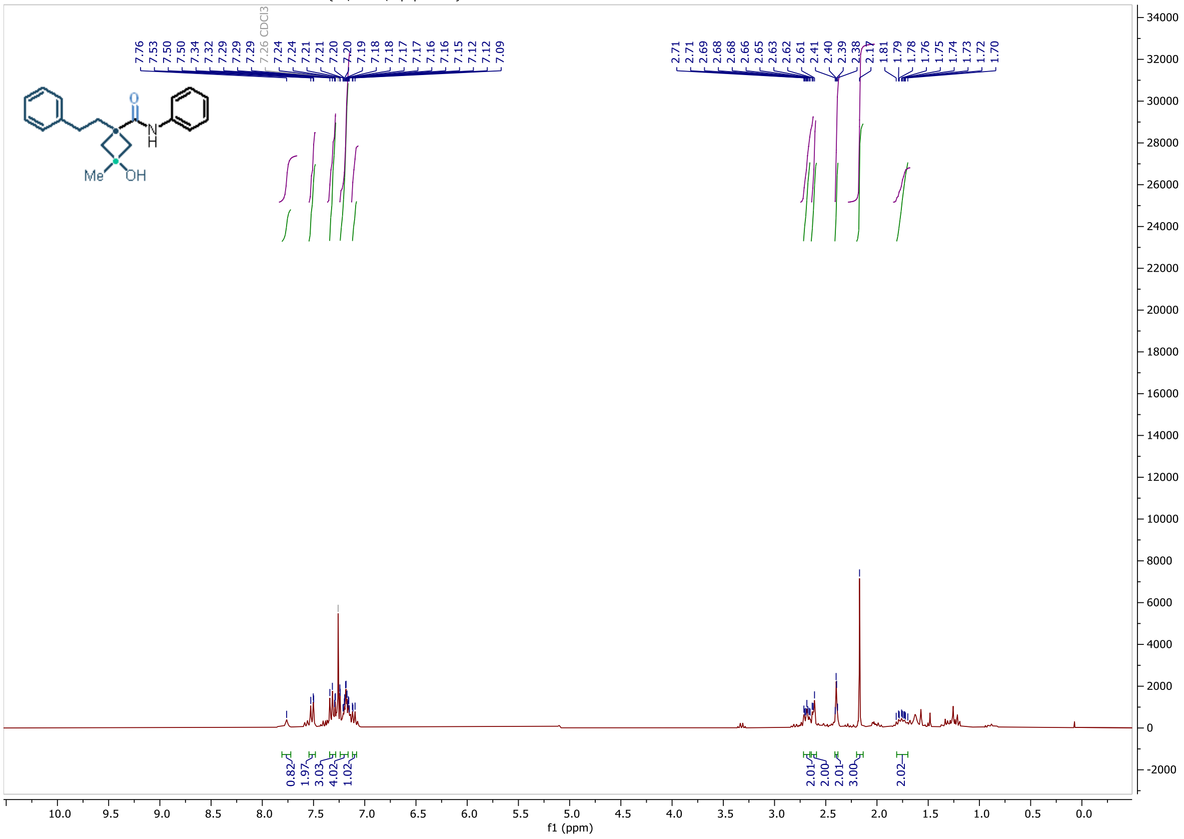

^{13}C NMR spectrum of **3ao** (75 MHz, $CDCl_3$)

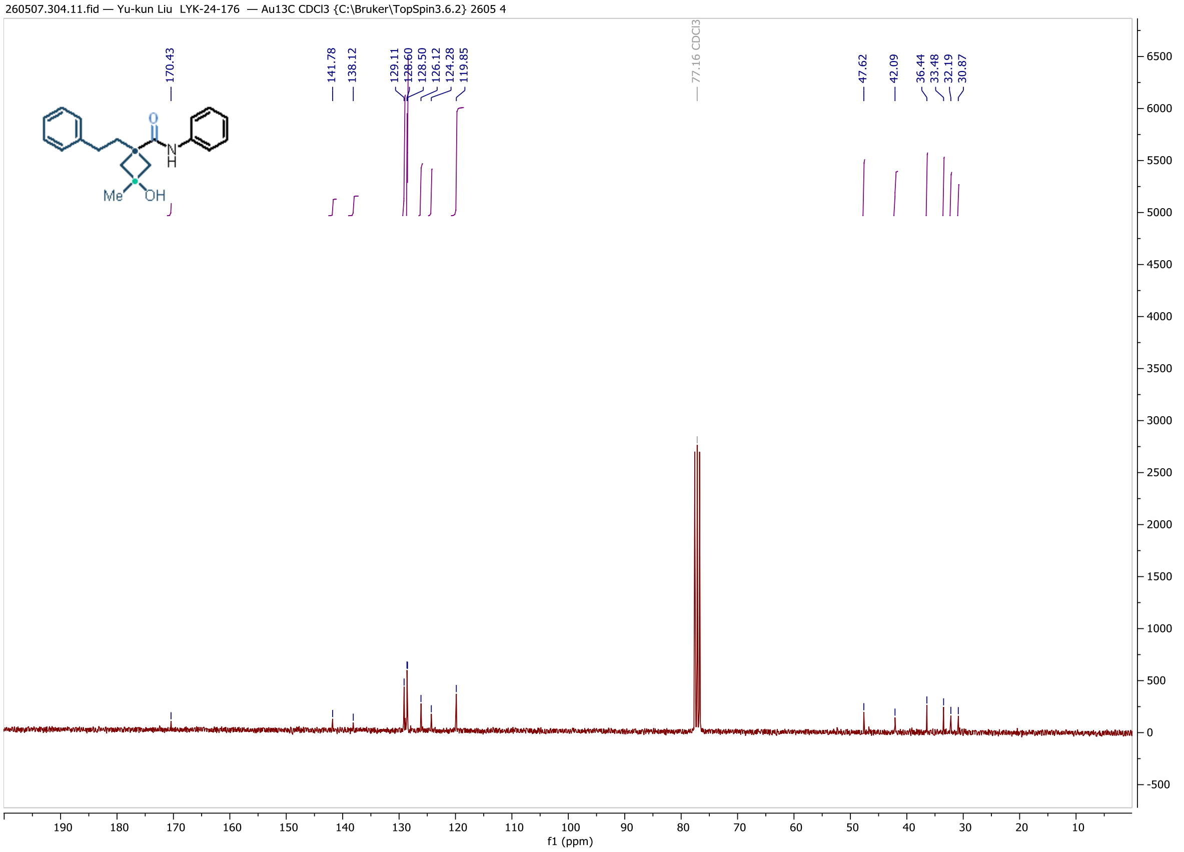

^{1}H NMR spectrum of **3ap** (400 MHz, $CDCl_3$)

260324.417.10.fid — Yu-kun Liu LYK-24-139 — Au1H CDCl3 {C:\Bruker\TopSpin3.6.2} 2603 17

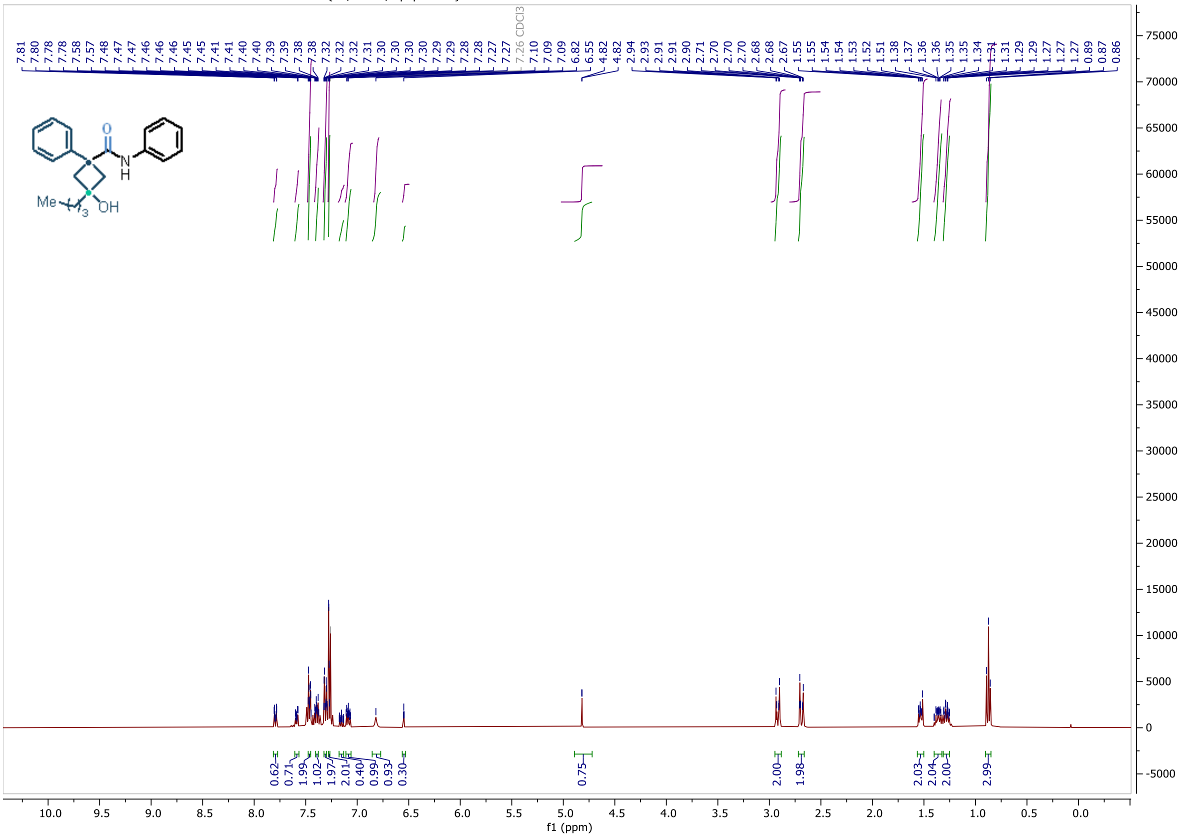

^{13}C NMR spectrum of **3ap** (101 MHz, $CDCl_3$)

260324.417.11.fid — Yu-kun Liu LYK-24-139 — Au13C CDCl3 {C:\Bruker\TopSpin3.6.2} 2603 17

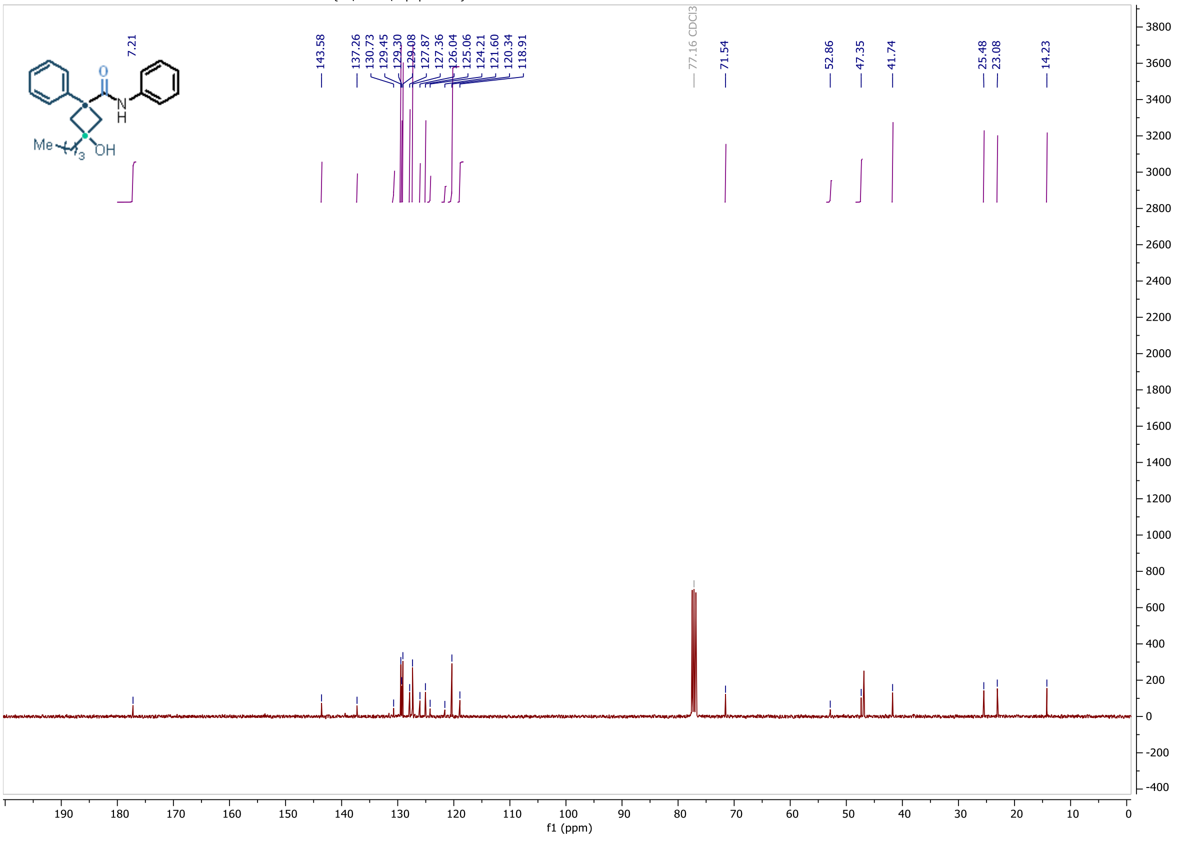

^{1}H NMR spectrum of **3aq** (300 MHz, $CDCl_3$)

260326.f308.10.fid — Yu-kun Liu LYK-24-147 — Au1H CDCl3 {C:\Bruker\TopSpin3.6.2} 2603 8

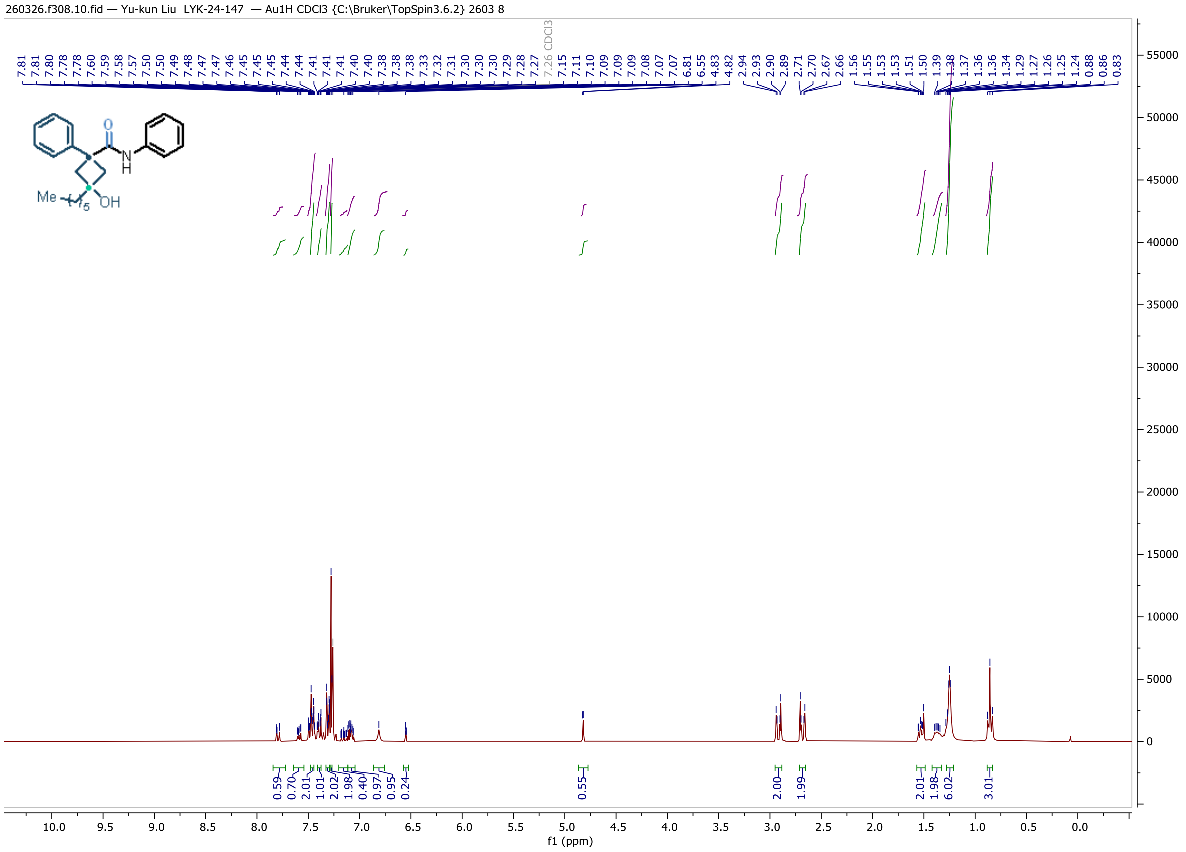

^{13}C NMR spectrum of **3aq** (75 MHz, $CDCl_3$)

260326.f308.11.fid — Yu-kun Liu LYK-24-147 — Au13C CDCl3 {C:\Bruker\TopSpin3.6.2} 2603 8

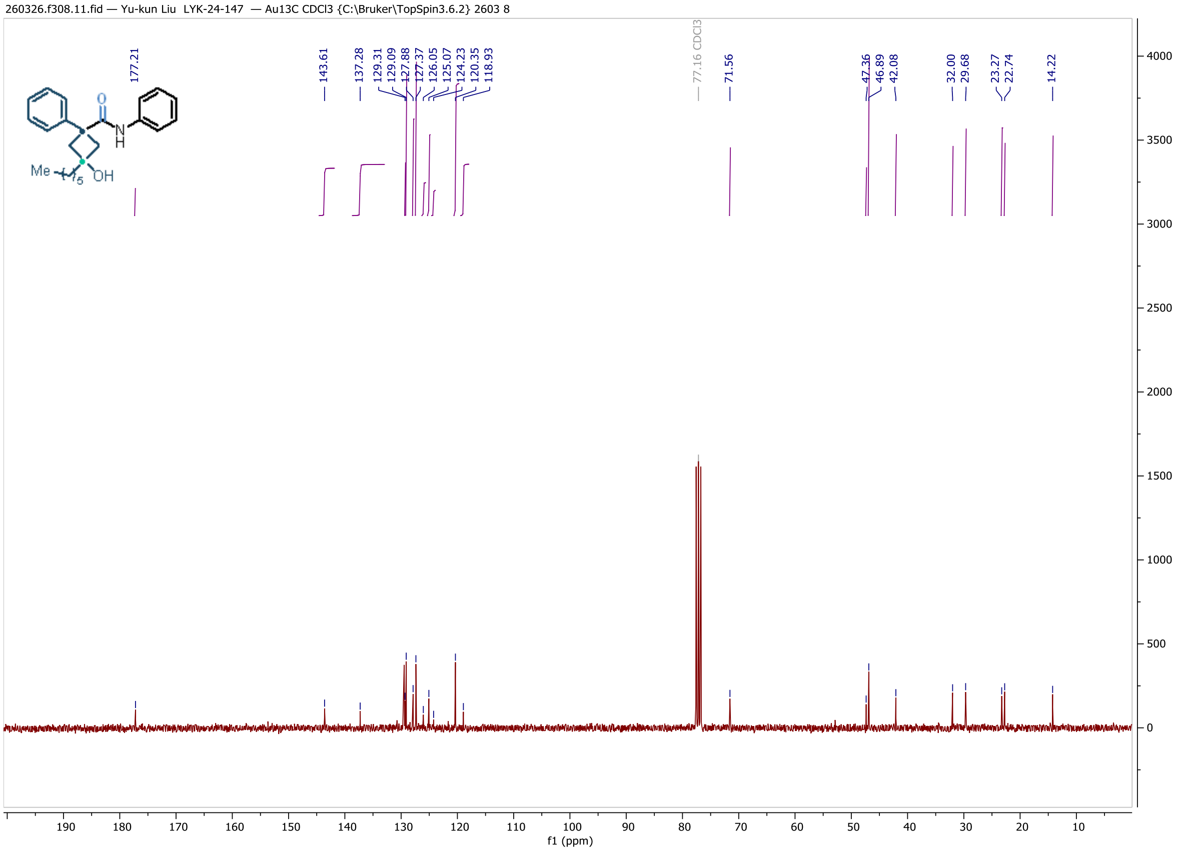

^{1}H NMR spectrum of **3at** (300 MHz, $CDCl_3$)

260323.310.10.fid — Yu-kun Liu LYK-24-119-re — Au1H CDCl3 {C:\Bruker\TopSpin3.6.2} 2603 10

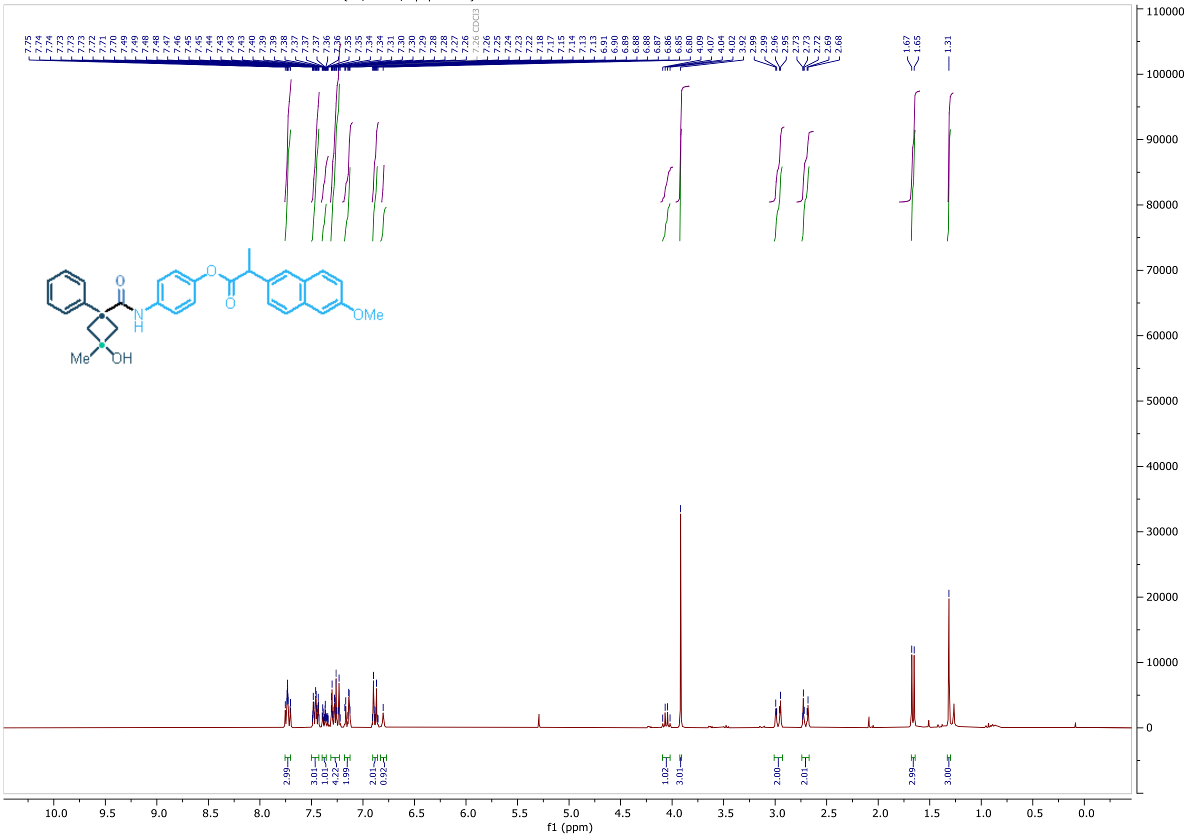

^{13}C NMR spectrum of **3at** (75 MHz, $CDCl_3$)

260323.310.11.fid — Yu-kun Liu LYK-24-119-re — Au13C CDCl3 {C:\Bruker\TopSpin3.6.2} 2603 10

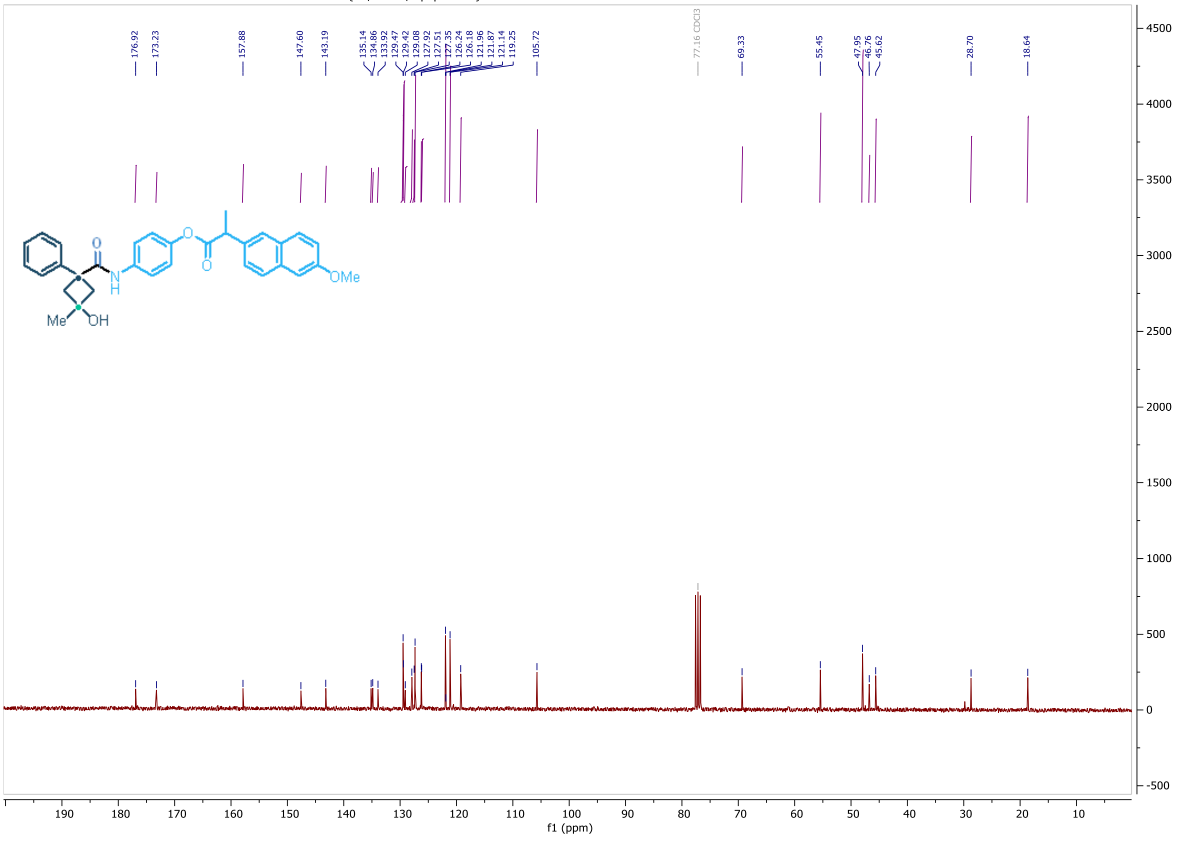

^{1}H NMR spectrum of **3au** (300 MHz, $CDCl_3$)

260402.320.10.fid — Yu-kun Liu, LYK-24-157 — Au1H CDCl3 {C:\Bruker\TopSpin3.6.2} 2604 20

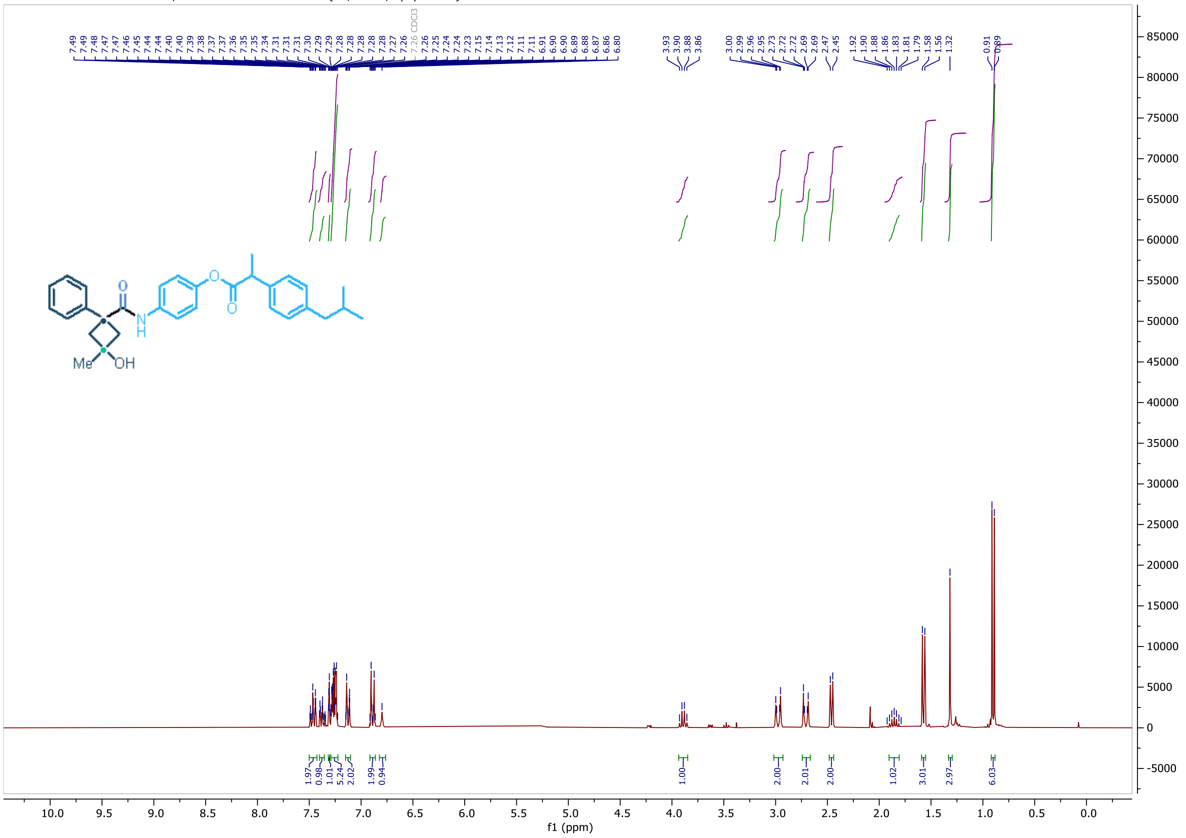

^{13}C NMR spectrum of **3au** (75 MHz, $CDCl_3$)

260402.320.11.fid — Yu-kun Liu, LYK-24-157 — Au13C CDCl3 {C:\Bruker\TopSpin3.6.2} 2604 20

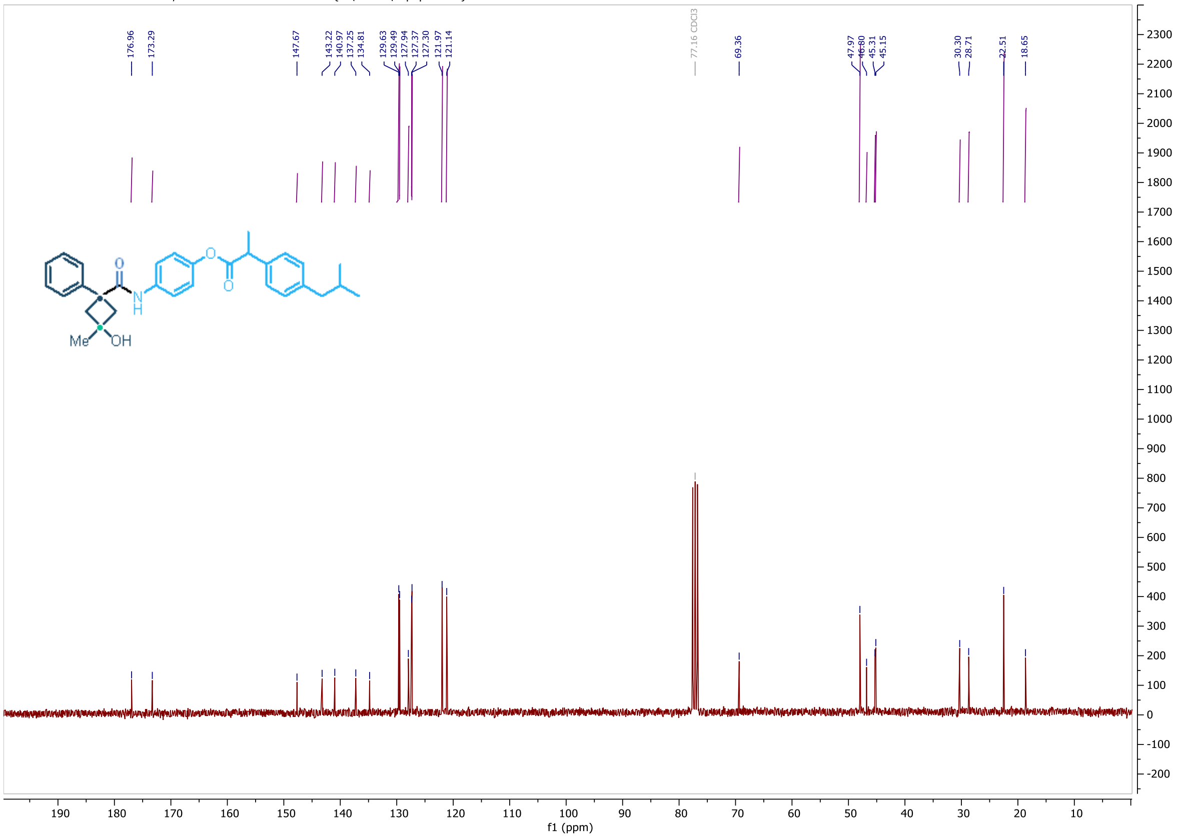

^{1}H NMR spectrum of **3av** (300 MHz, $CDCl_3$)

260319.339.10.fid — Yu-ku Liu, LYK-24-121 — Au1H CDCl3 {C:\Bruker\TopSpin3.6.2} 2603 39

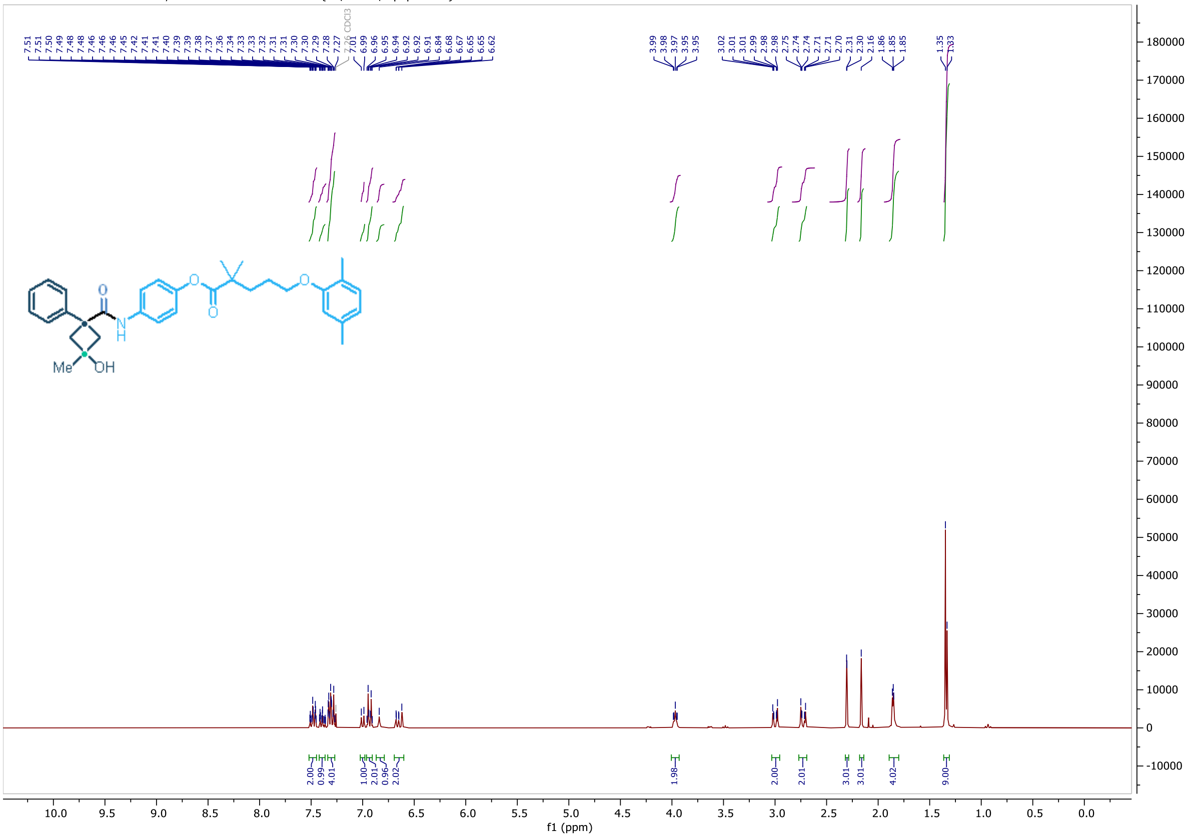

^{13}C NMR spectrum of **3av** (75 MHz, $CDCl_3$)

260319.339.11.fid — Yu-ku Liu, LYK-24-121 — Au13C CDCl3 {C:\Bruker\TopSpin3.6.2} 2603 39

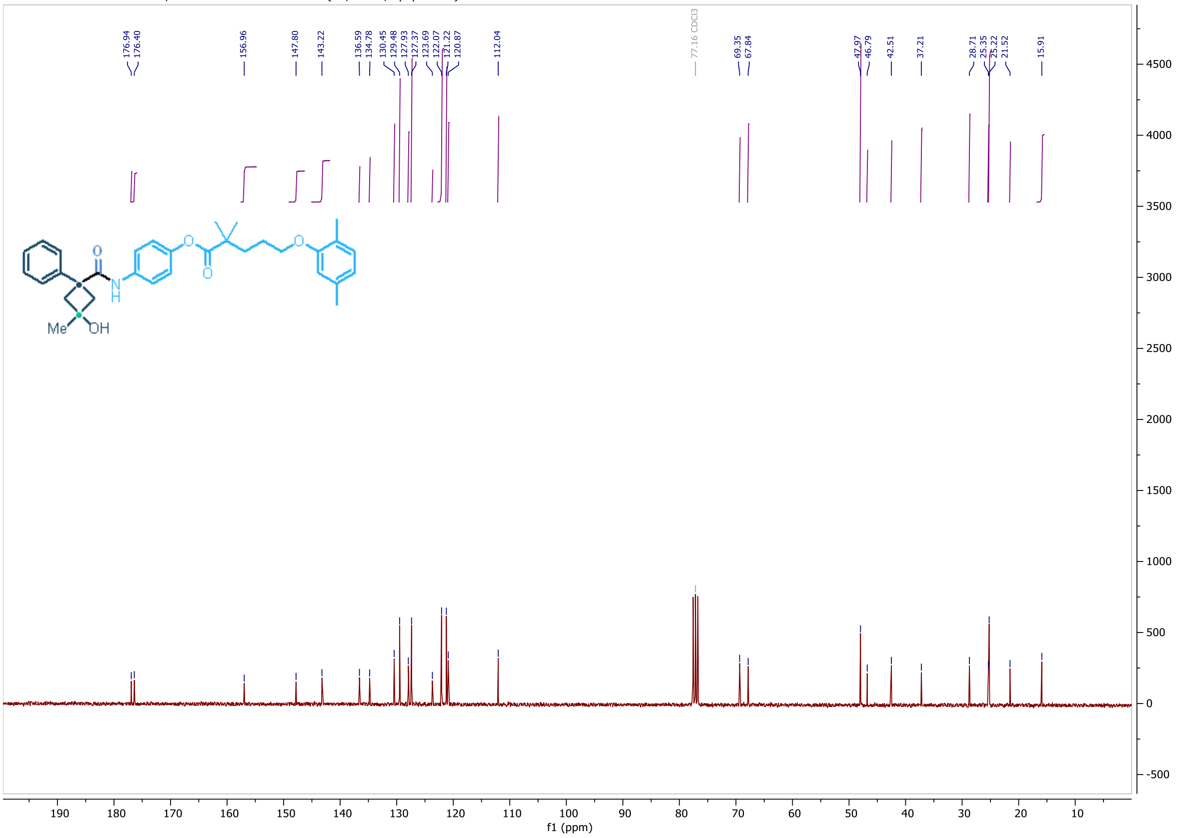

^{1}H NMR spectrum of **3aw** (300 MHz, $CDCl_3$)

260402.319.10.fid — Yu-kun Liu, LYK-24-160 — Au1H CDCl3 {C:\Bruker\TopSpin3.6.2} 2604 19

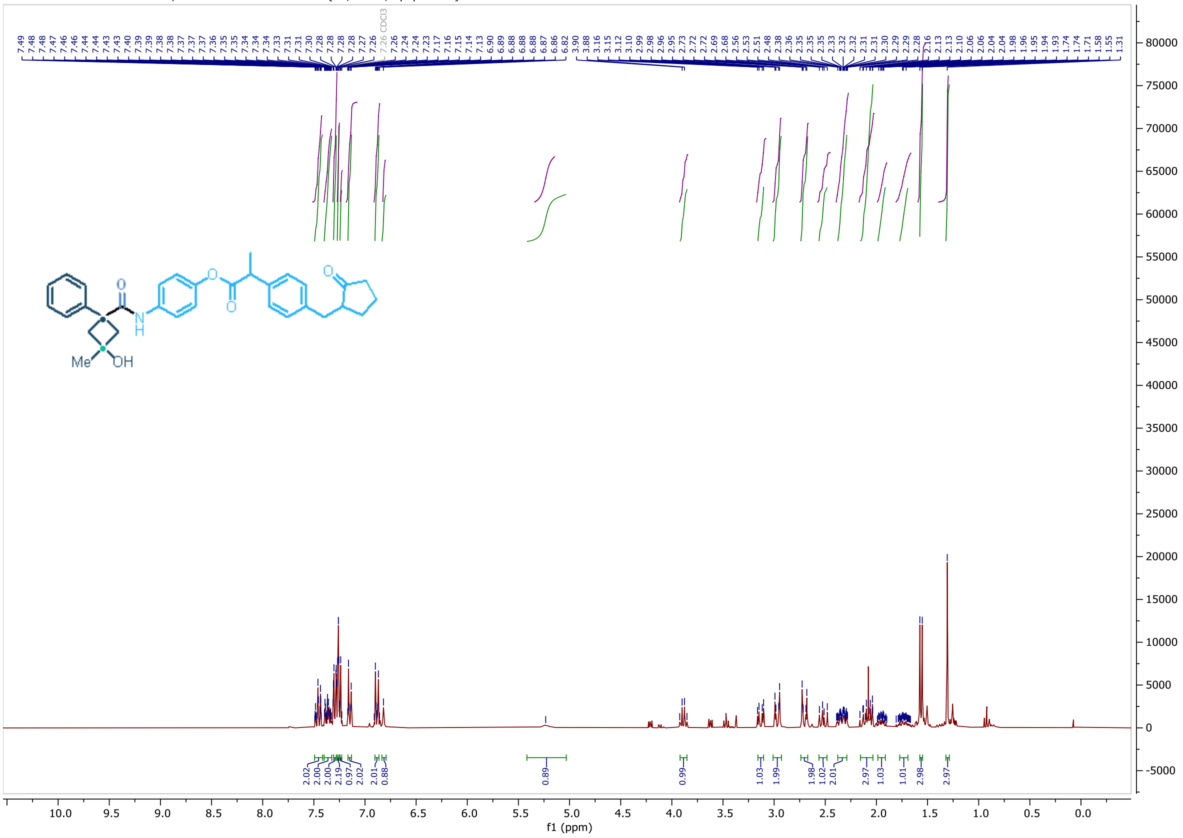

^{13}C NMR spectrum of **3aw** (75 MHz, $CDCl_3$)

260402.319.11.fid — Yu-kun Liu, LYK-24-160 — Au13C CDCl3 {C:\Bruker\TopSpin3.6.2} 2604 19

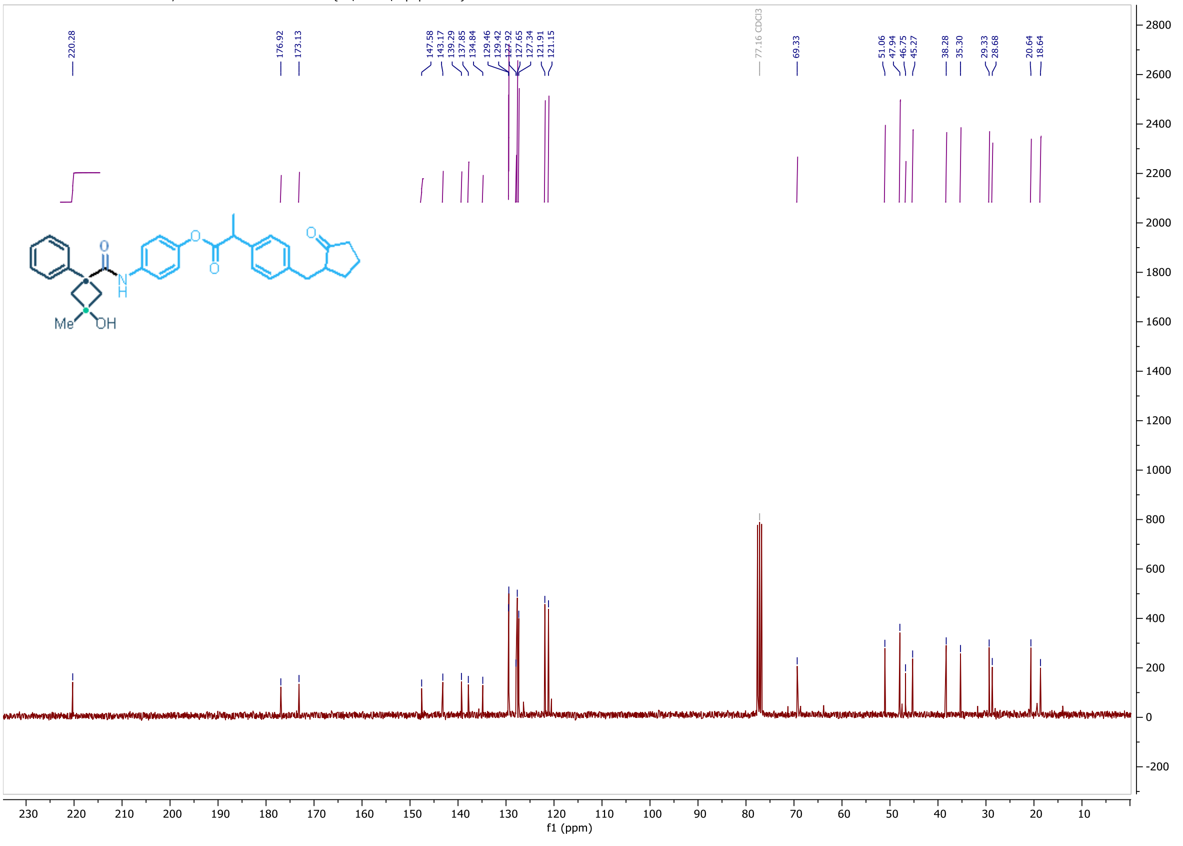

^{1}H NMR spectrum of **3ax** (300 MHz, $CDCl_3$)

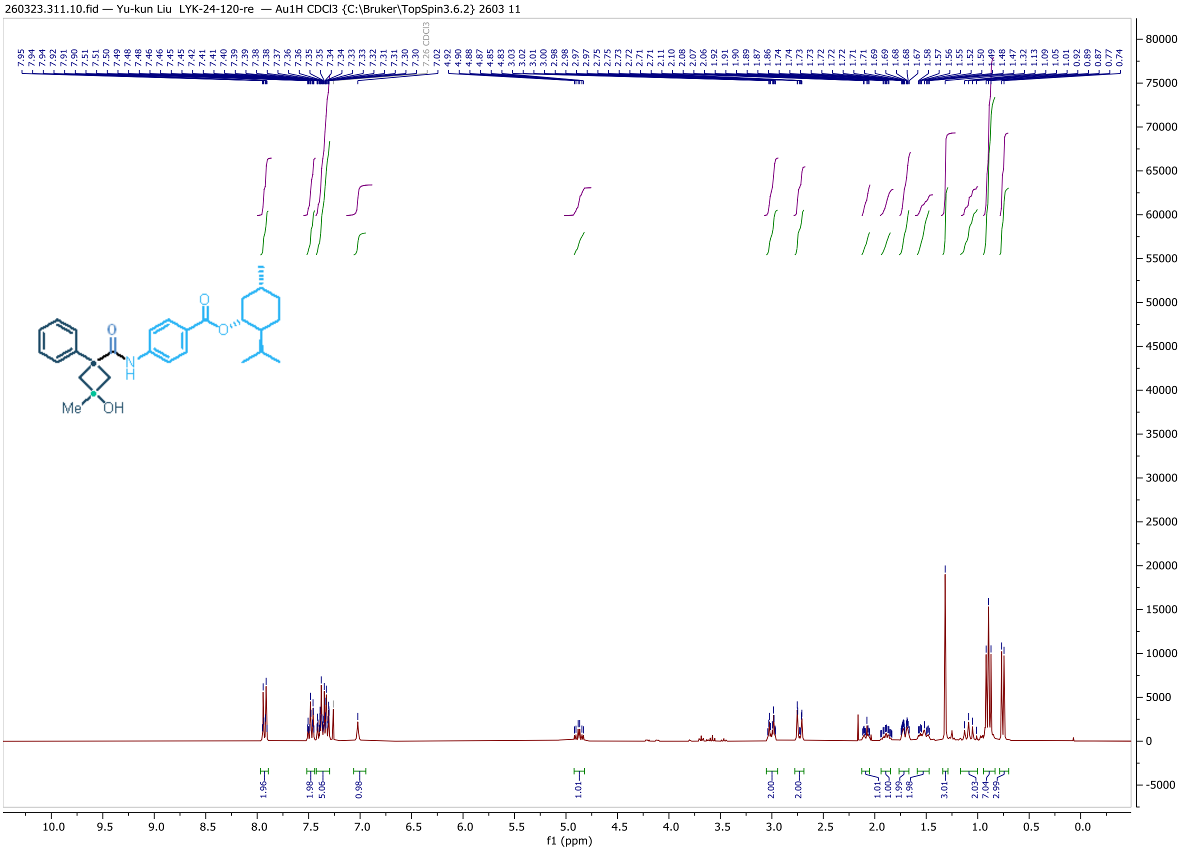

^{13}C NMR spectrum of **3ax** (75 MHz, $CDCl_3$)

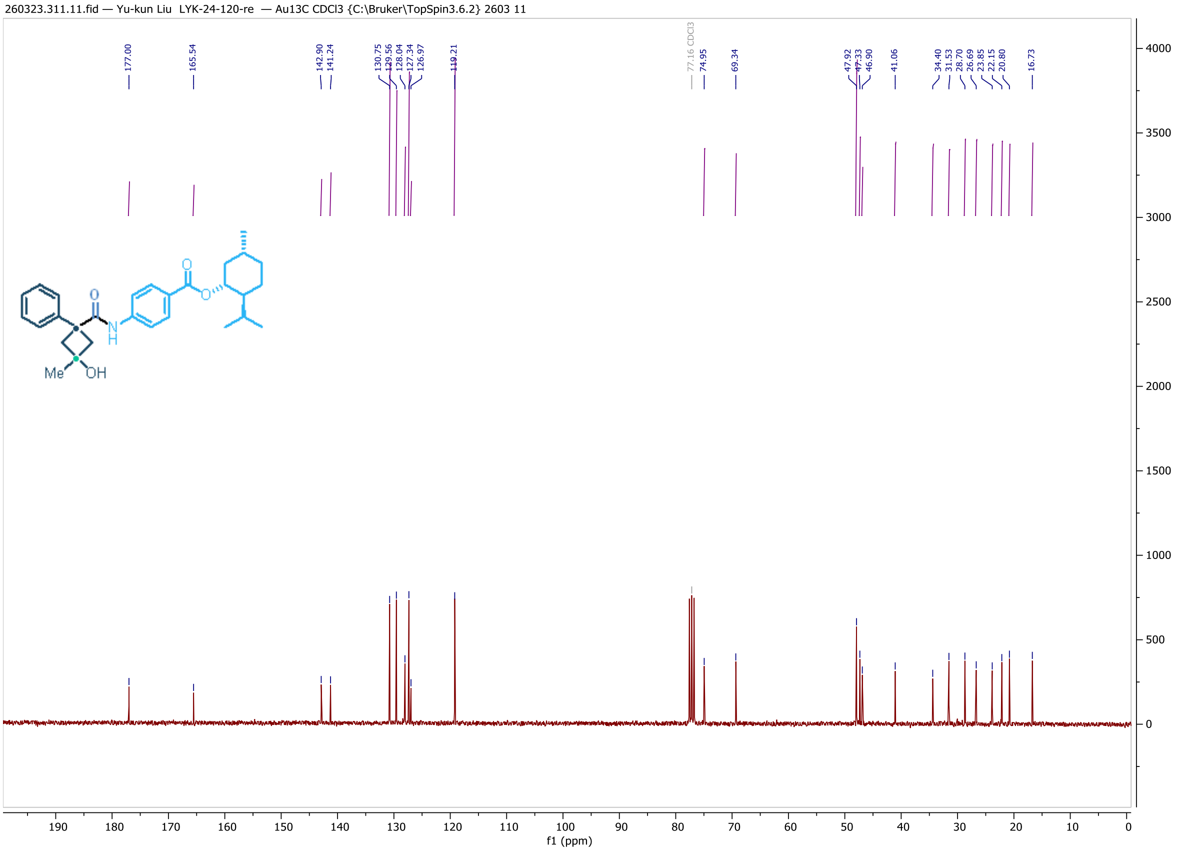

^{1}H NMR spectrum of 4**a** (300 MHz, $CDCl_3$)

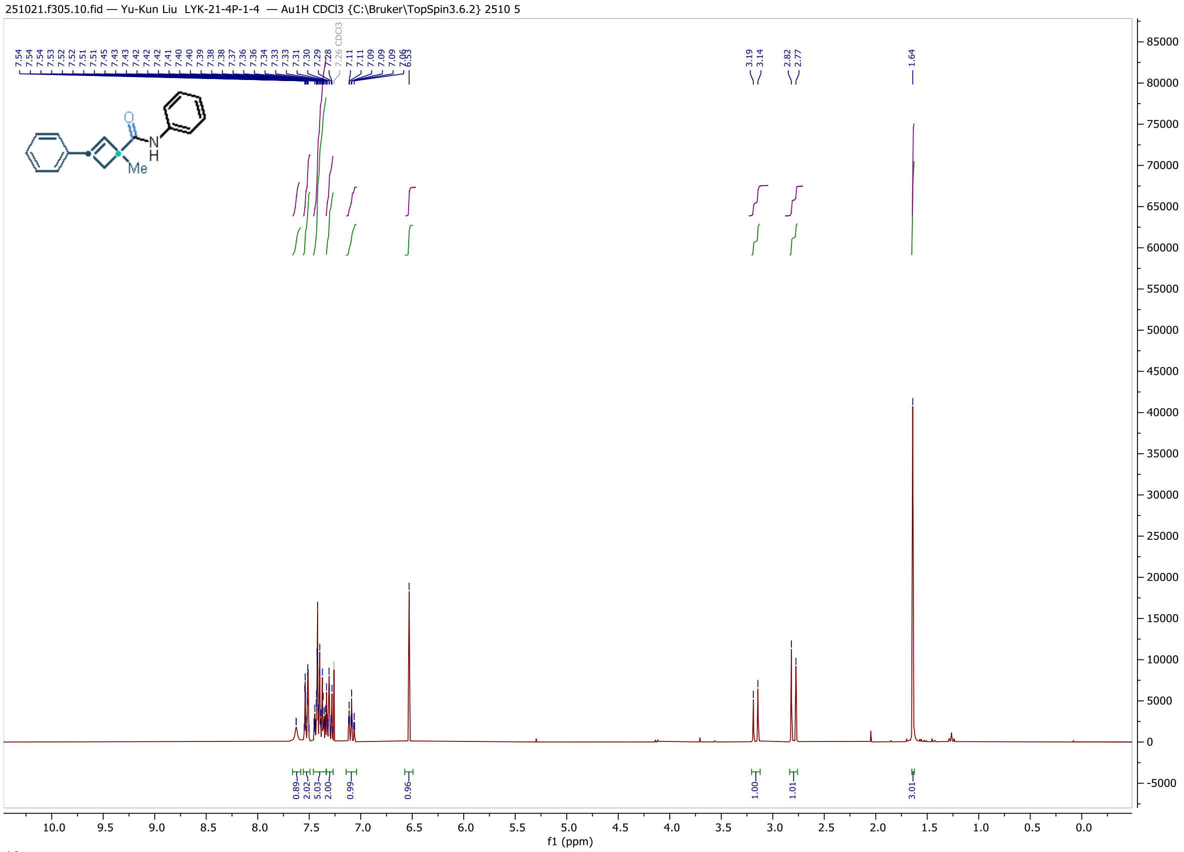

^{13}C NMR spectrum of **4a** (75 MHz, $CDCl_3$)

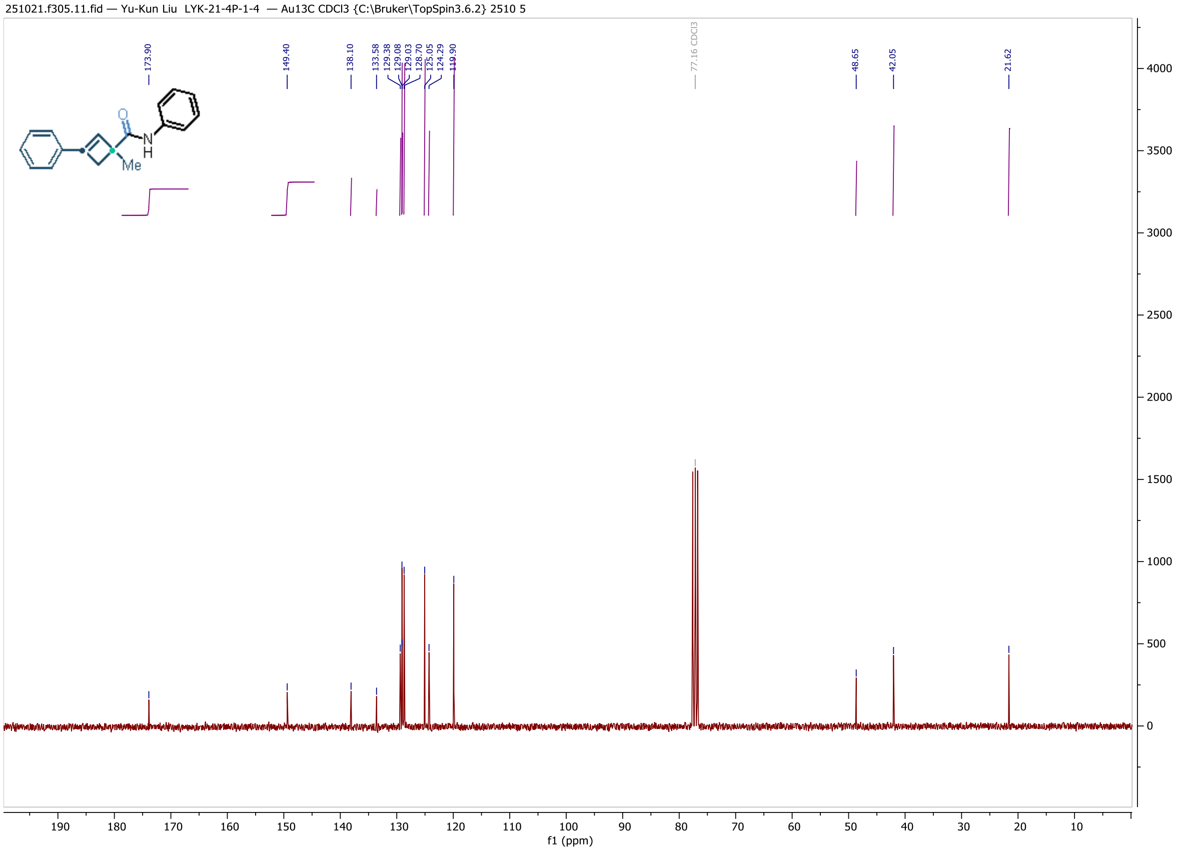

^{1}H NMR spectrum of 4**b** (300 MHz, $CDCl_3$)

260223.324.10.fid — Yu-Kun Liu LYK-24-2-80 — Au1H CDCl3 {C:\Bruker\TopSpin3.6.2} 2602 24

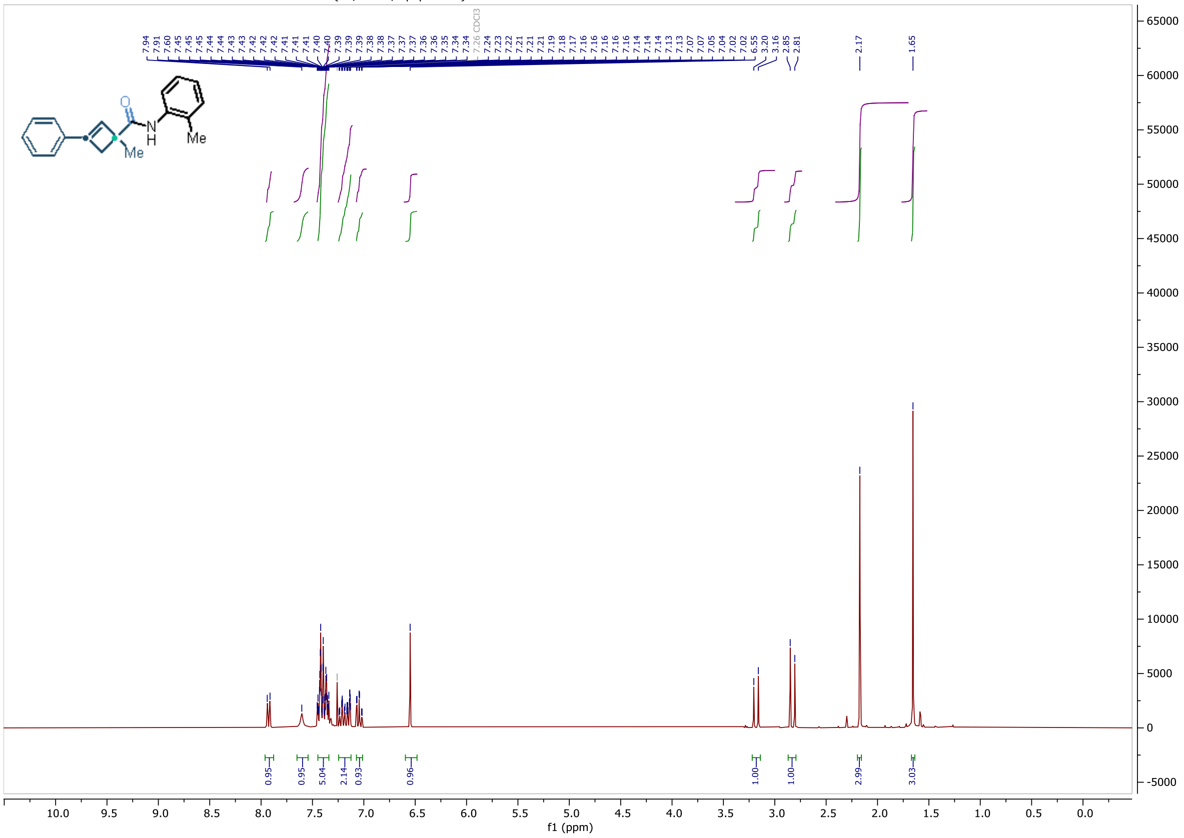

^{13}C NMR spectrum of **4b** (75 MHz, $CDCl_3$)

260223.324.12.fid — Yu-Kun Liu LYK-24-2-80 — Au13C CDCl3 {C:\Bruker\TopSpin3.6.2} 2602 24

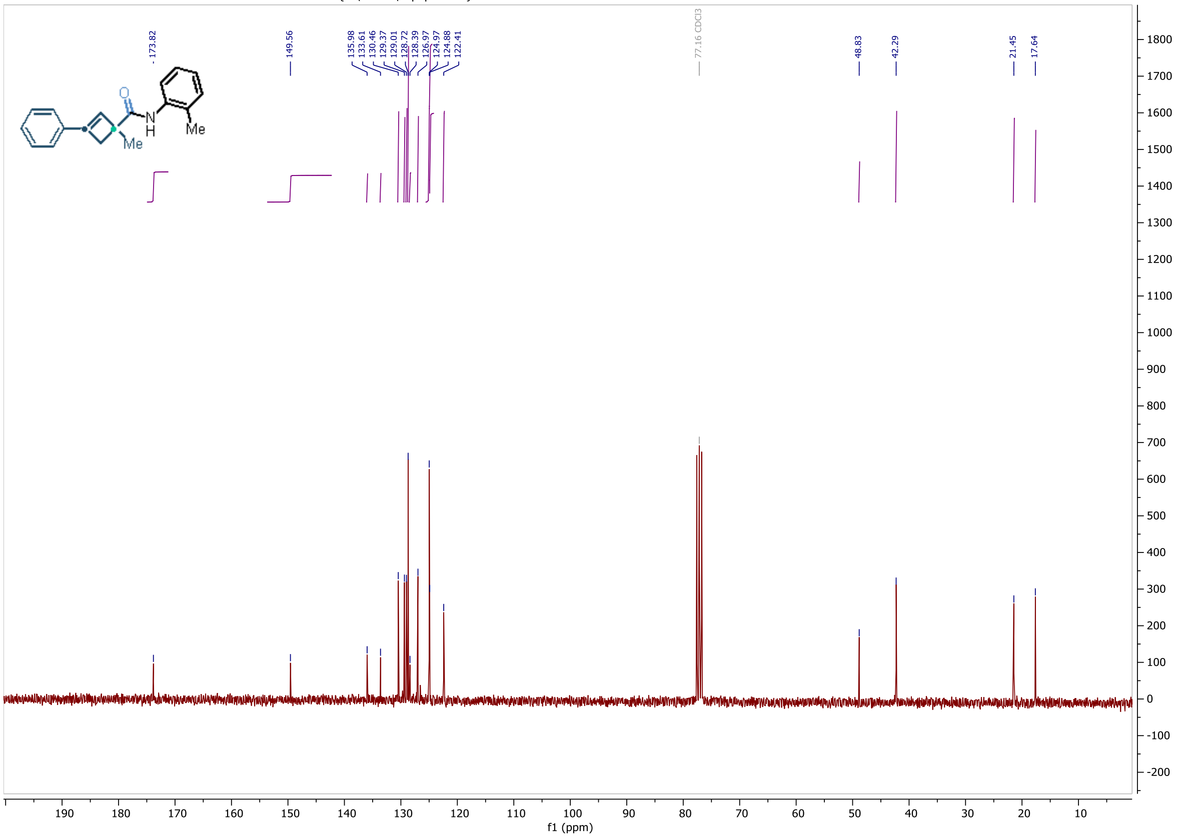

^{1}H NMR spectrum of 4**c** (300 MHz, $CDCl_3$)

260220.312.10.fid — Yu-kun Liu LYK-24-2-79 — Au1H CDCl3 {C:\Bruker\TopSpin3.6.2} 2602 12

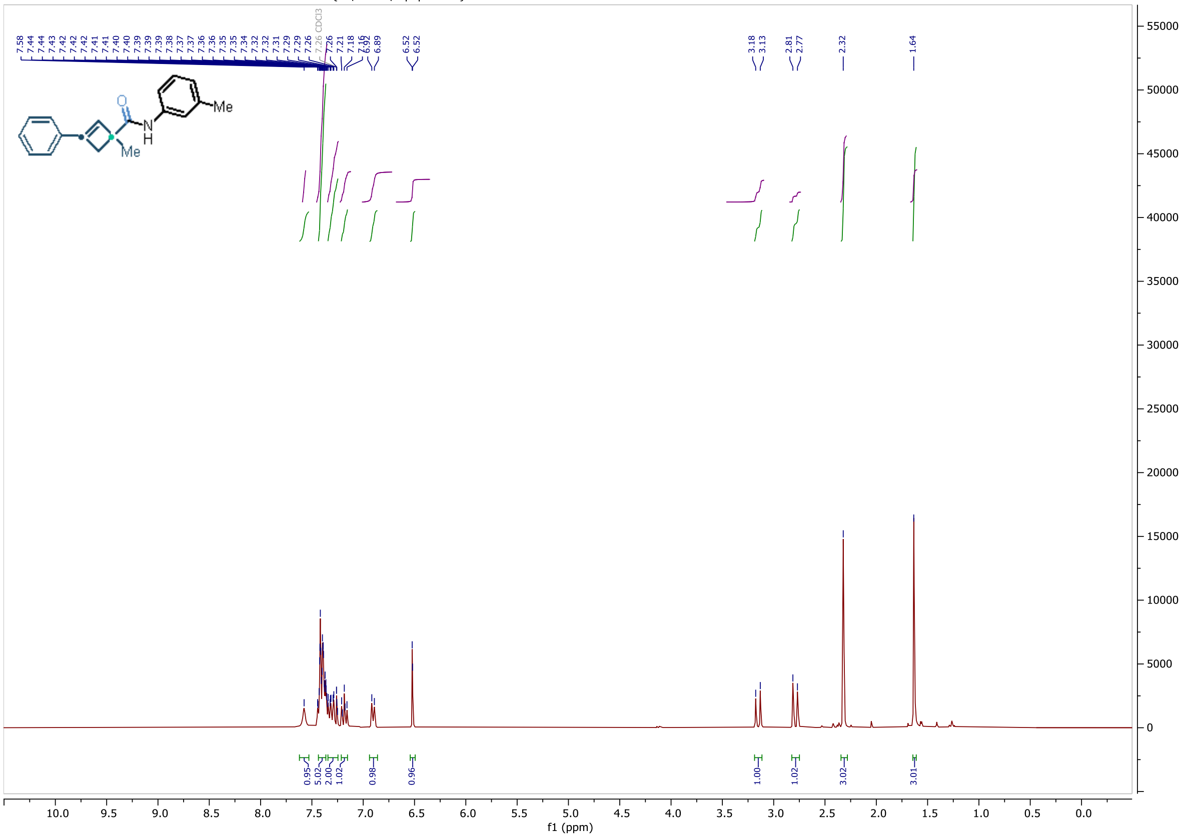

^{13}C NMR spectrum of **4c** (75 MHz, $CDCl_3$)

260220.312.11.fid — Yu-kun Liu LYK-24-2-79 — Au13C CDCl3 {C:\Bruker\TopSpin3.6.2} 2602 12

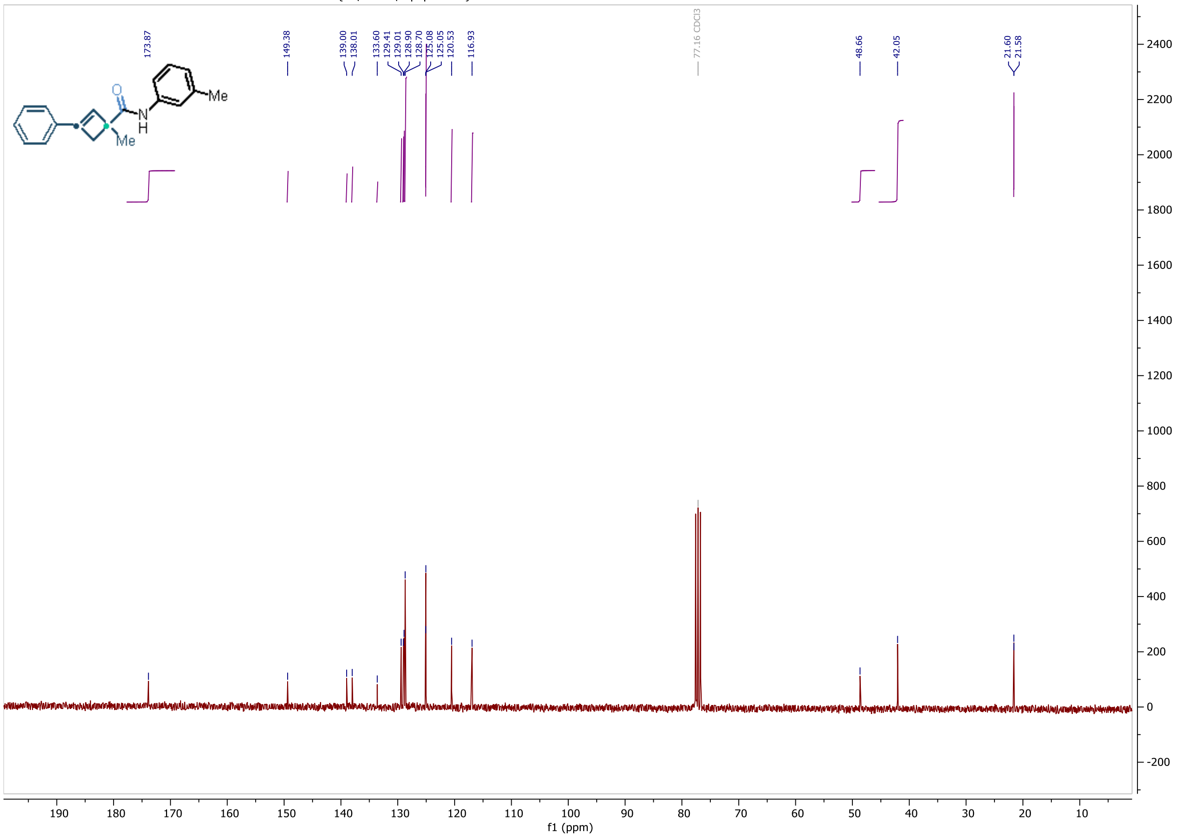

^{1}H NMR spectrum of 4**d** (300 MHz, $CDCl_3$)

260326.343.10.fid — Yu-kun Liu, LYK-24-2-116 — Au1H CDCl3 {C:\Bruker\TopSpin3.6.2} 2603 43

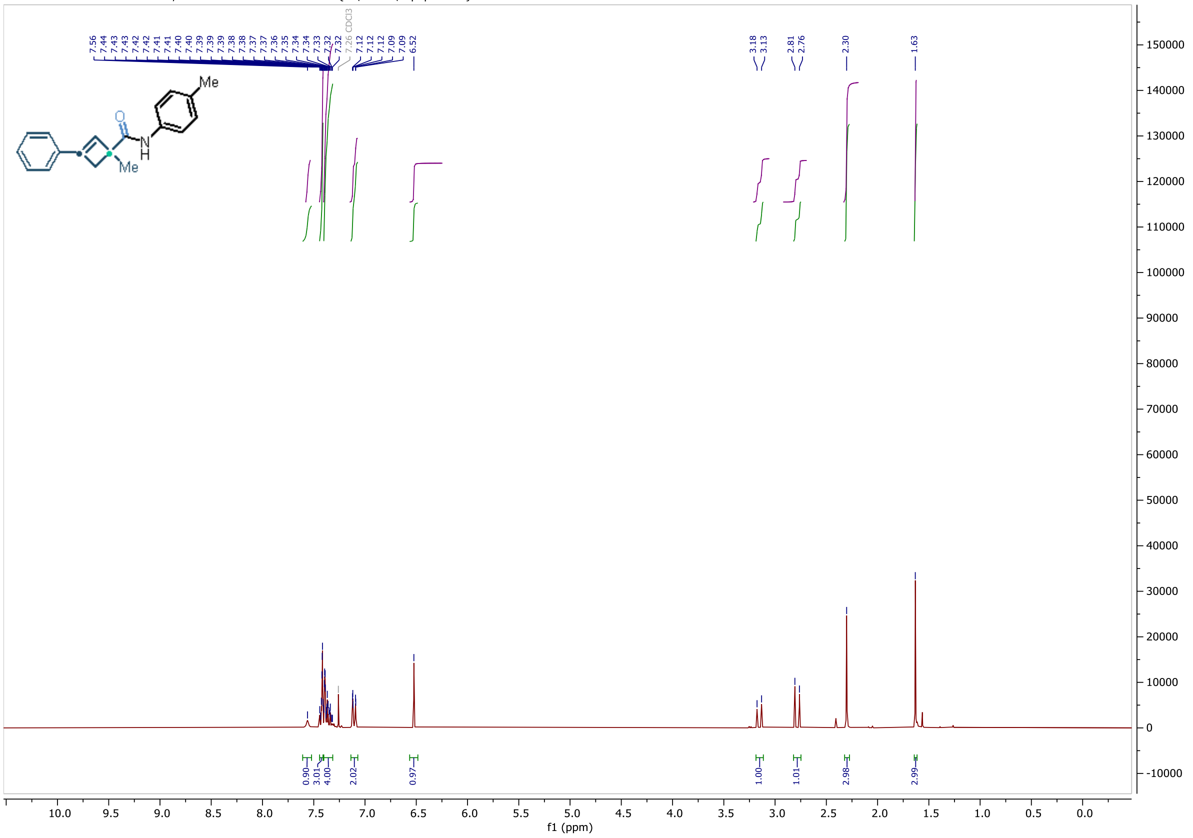

^{13}C NMR spectrum of **4d** (75 MHz, $CDCl_3$)

260326.343.11.fid — Yu-kun Liu, LYK-24-2-116 — Au13C CDCl3 {C:\Bruker\TopSpin3.6.2} 2603 43

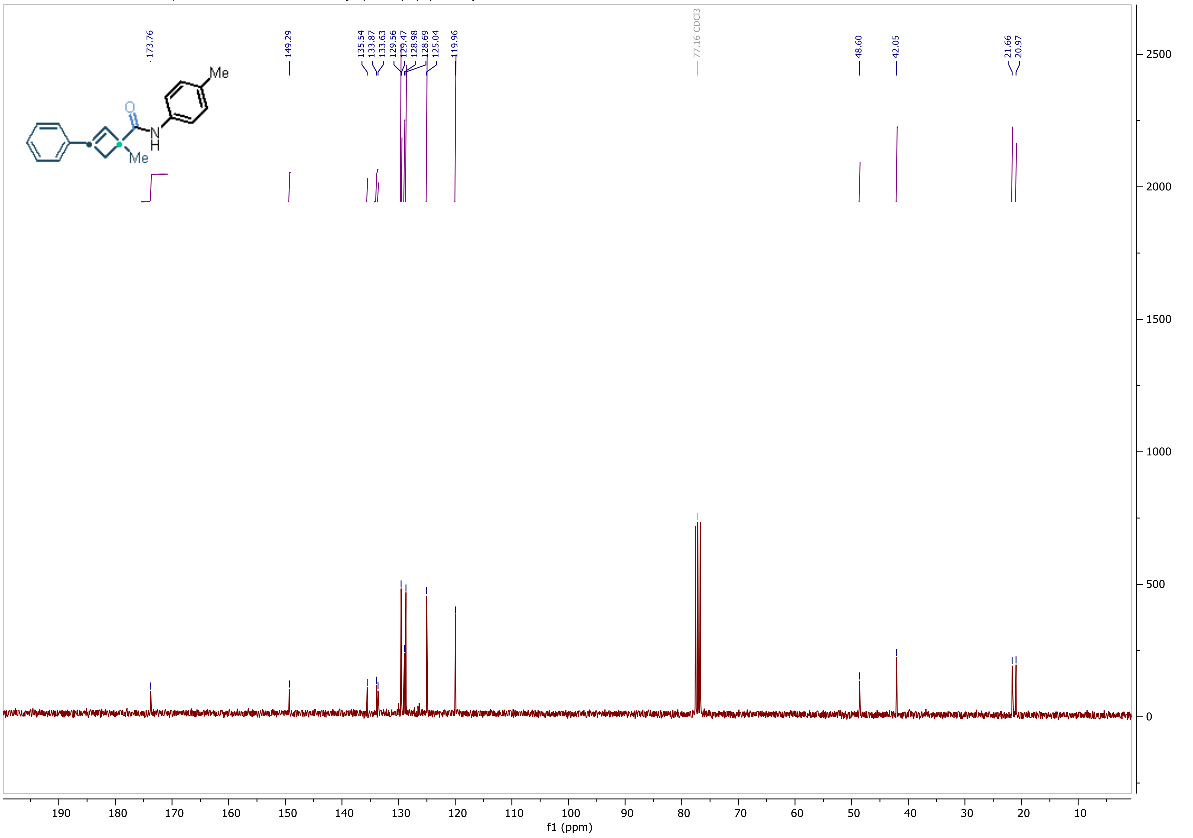

^{1}H NMR spectrum of 4**e** (300 MHz, $CDCl_3$)

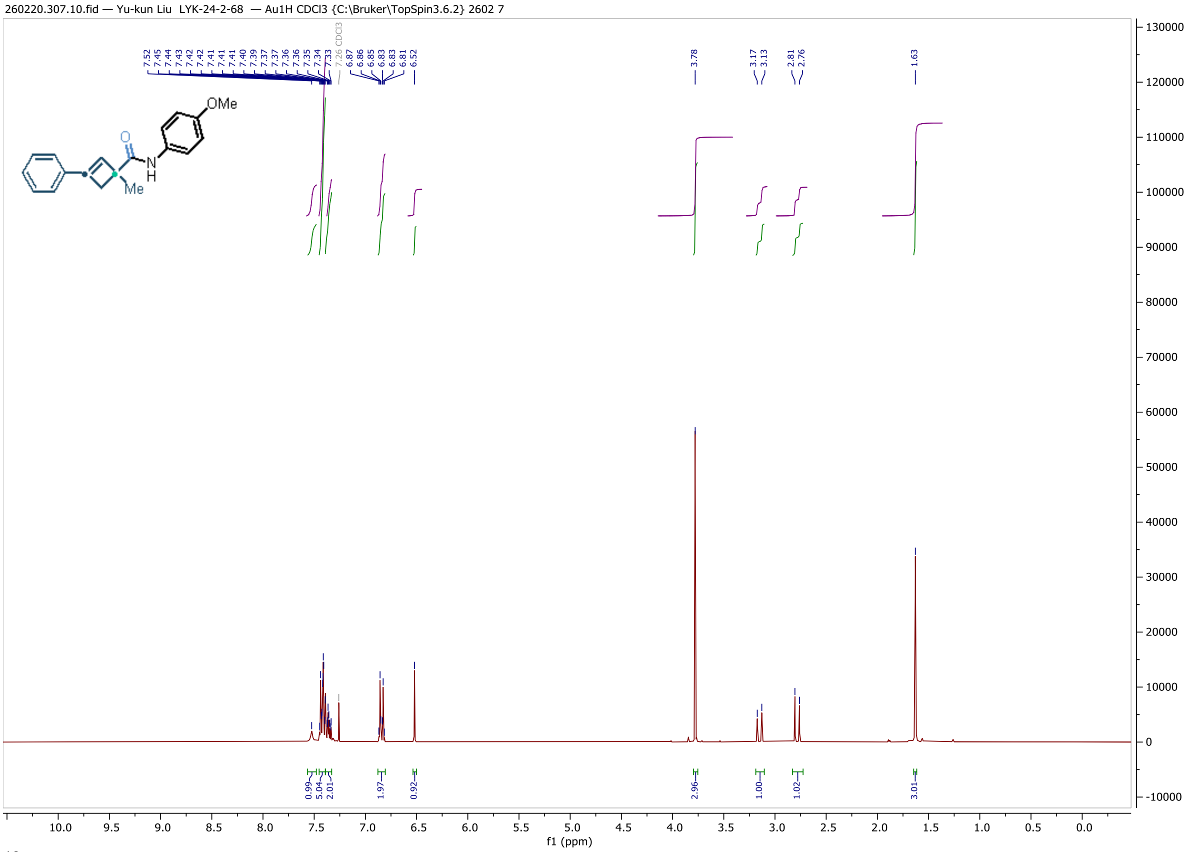

^{13}C NMR spectrum of **4e** (75 MHz, $CDCl_3$)

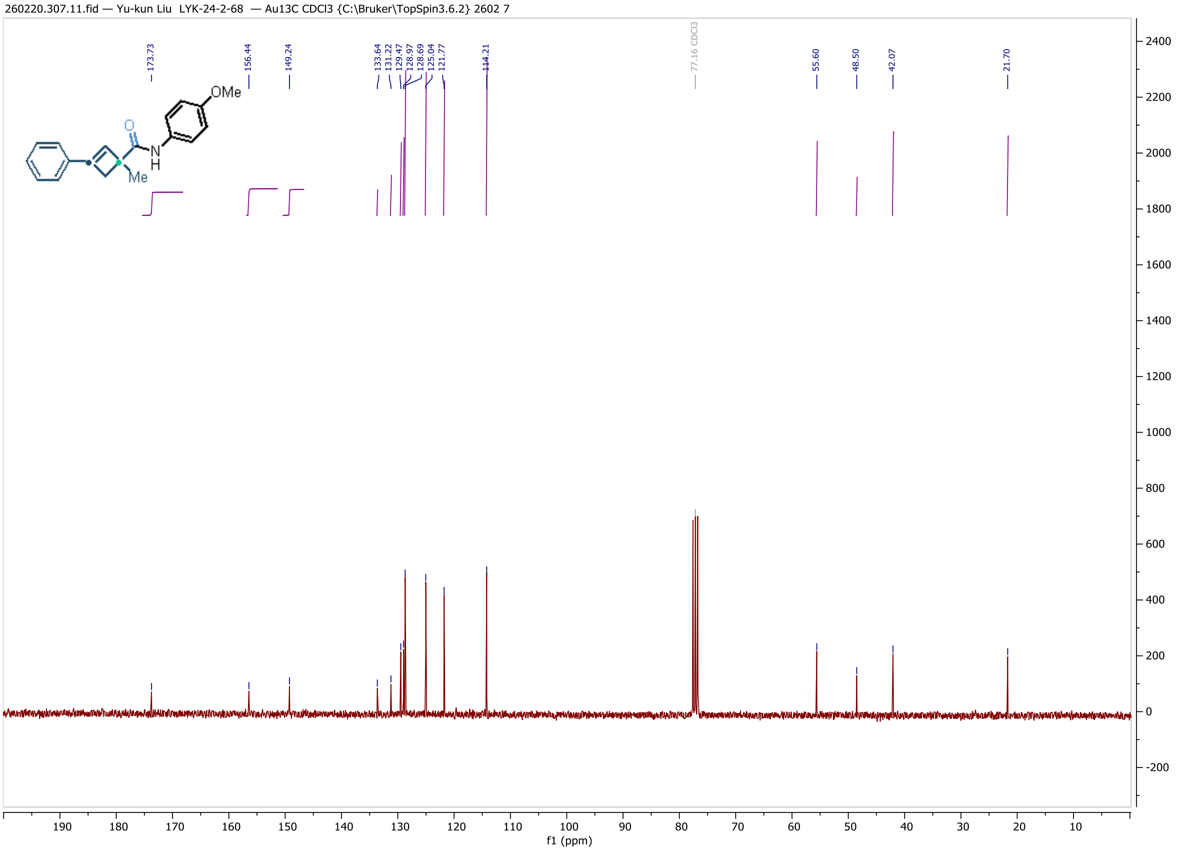

^{1}H NMR spectrum of 4**f** (300 MHz, $CDCl_3$)

260211.304.10.fid — Yu-kun Liu LYK-24-2-62 — Au1H CDCl3 {C:\Bruker\TopSpin3.6.2} 2602 4

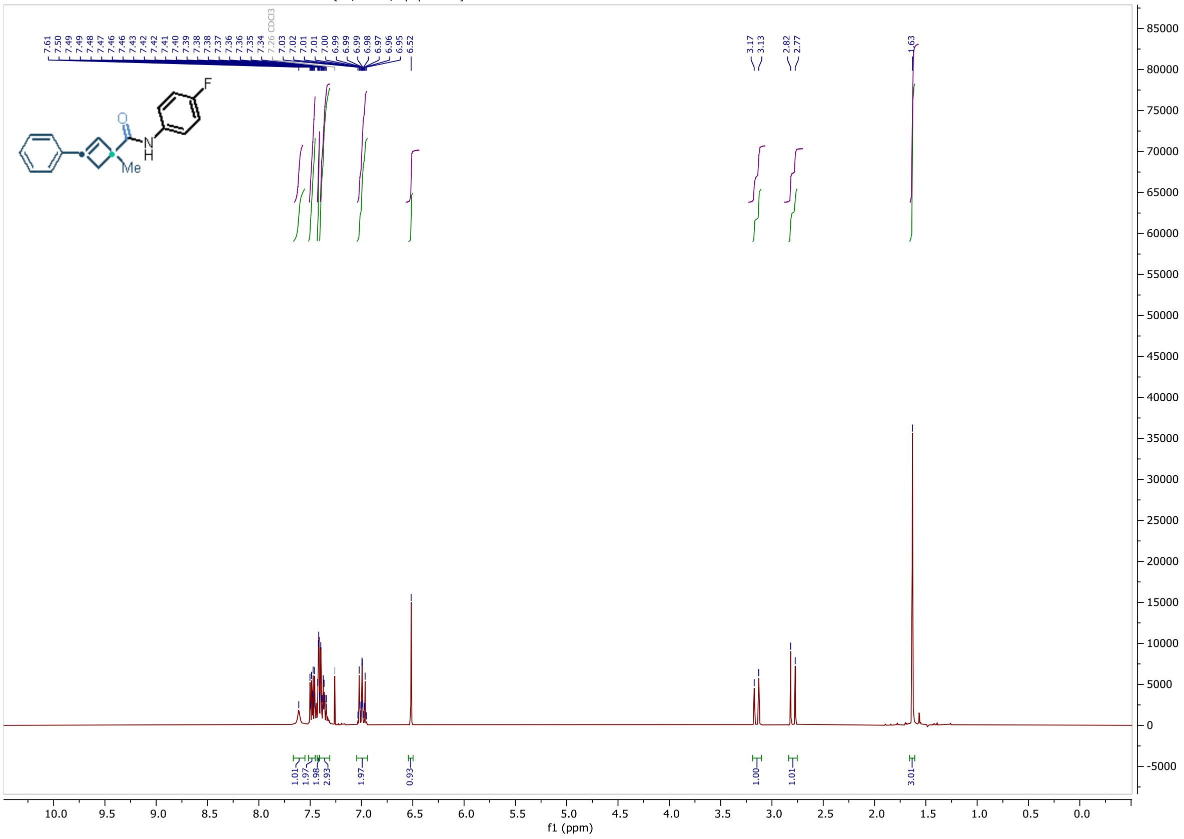

^{13}C NMR spectrum of **4f** (75 MHz, $CDCl_3$)

260211.304.11.fid — Yu-kun Liu LYK-24-2-62 — Au13C CDCl3 {C:\Bruker\TopSpin3.6.2} 2602 4

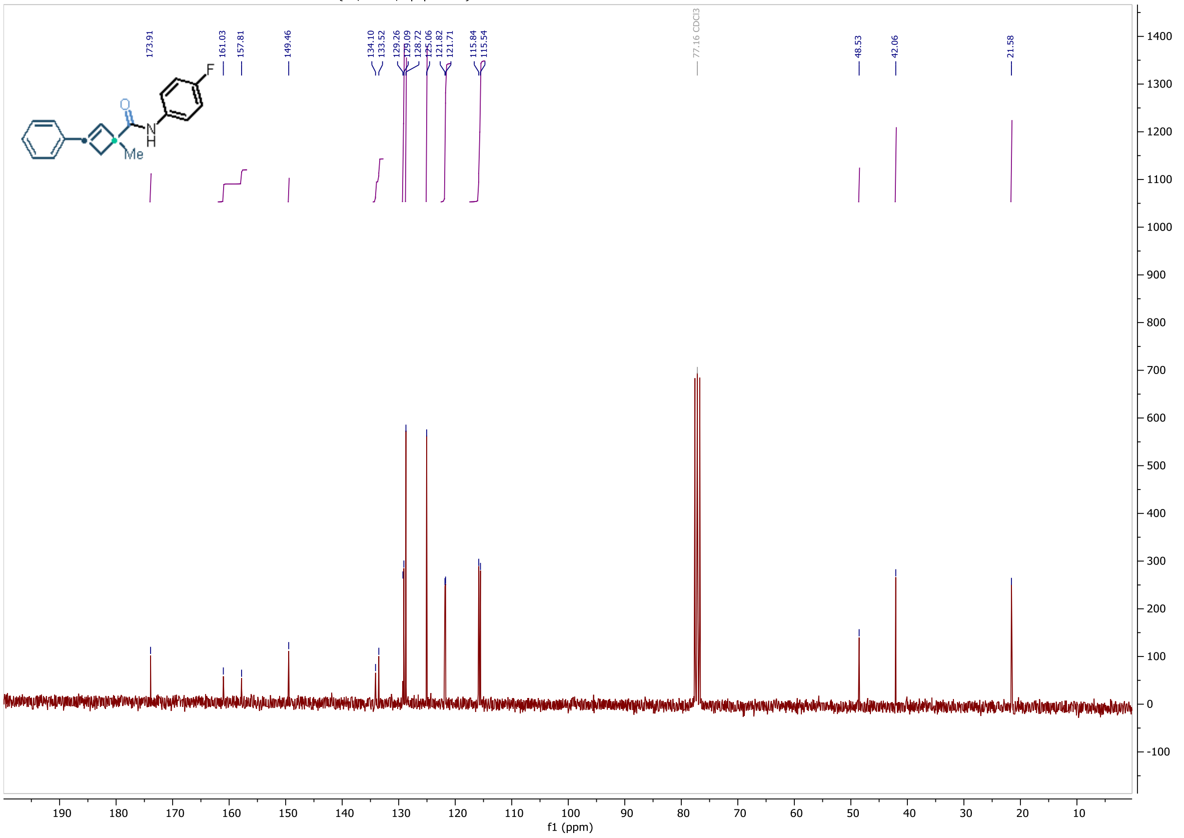

^{19}F NMR spectrum of **4e** (282 MHz, $CDCl_3$)

260211.304.12.fid — Yu-kun Liu LYK-24-2-62 — Au19F CDCl3 {C:\Bruker\TopSpin3.6.2} 2602 4

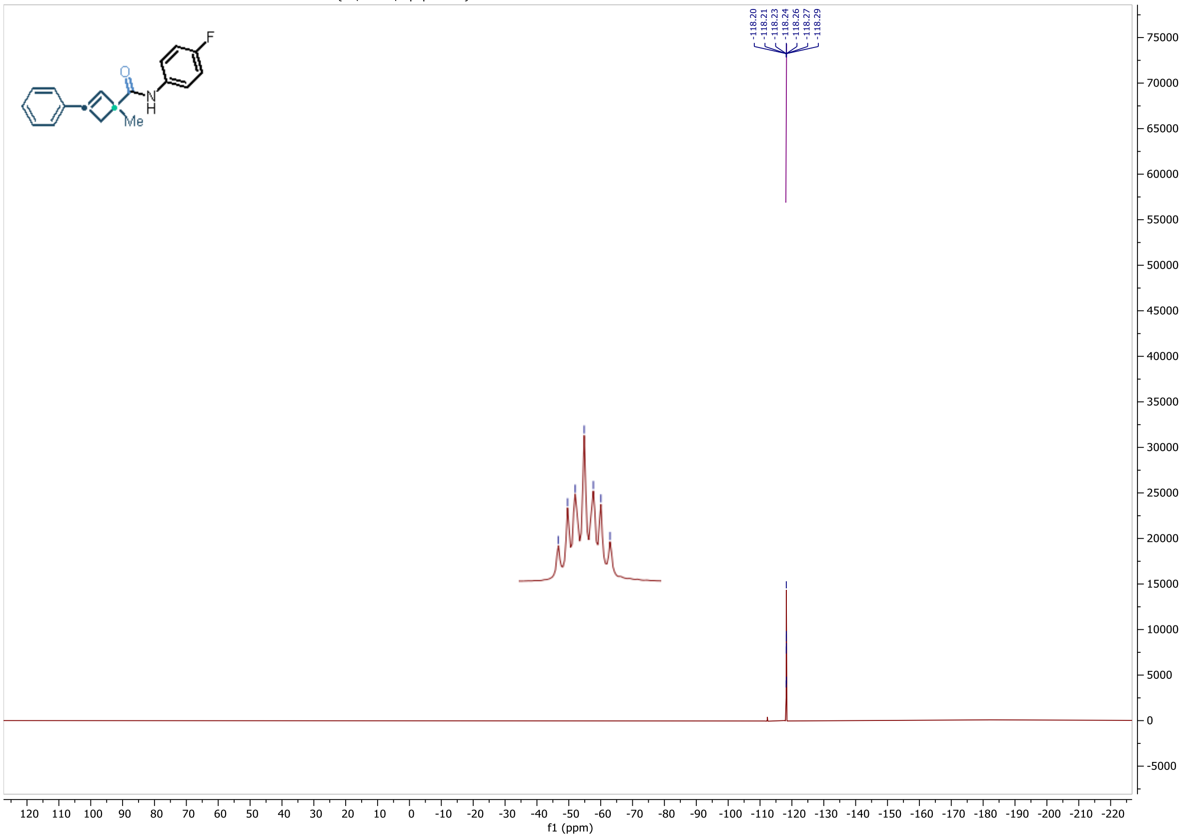

^{1}H NMR spectrum of 4**g** (400 MHz, $CDCl_3$)

260217.405.10.fid — Yu-kun Liu LYK-24-2-69 — Au1H CDCl3 {C:\Bruker\TopSpin3.6.2} 2602 5

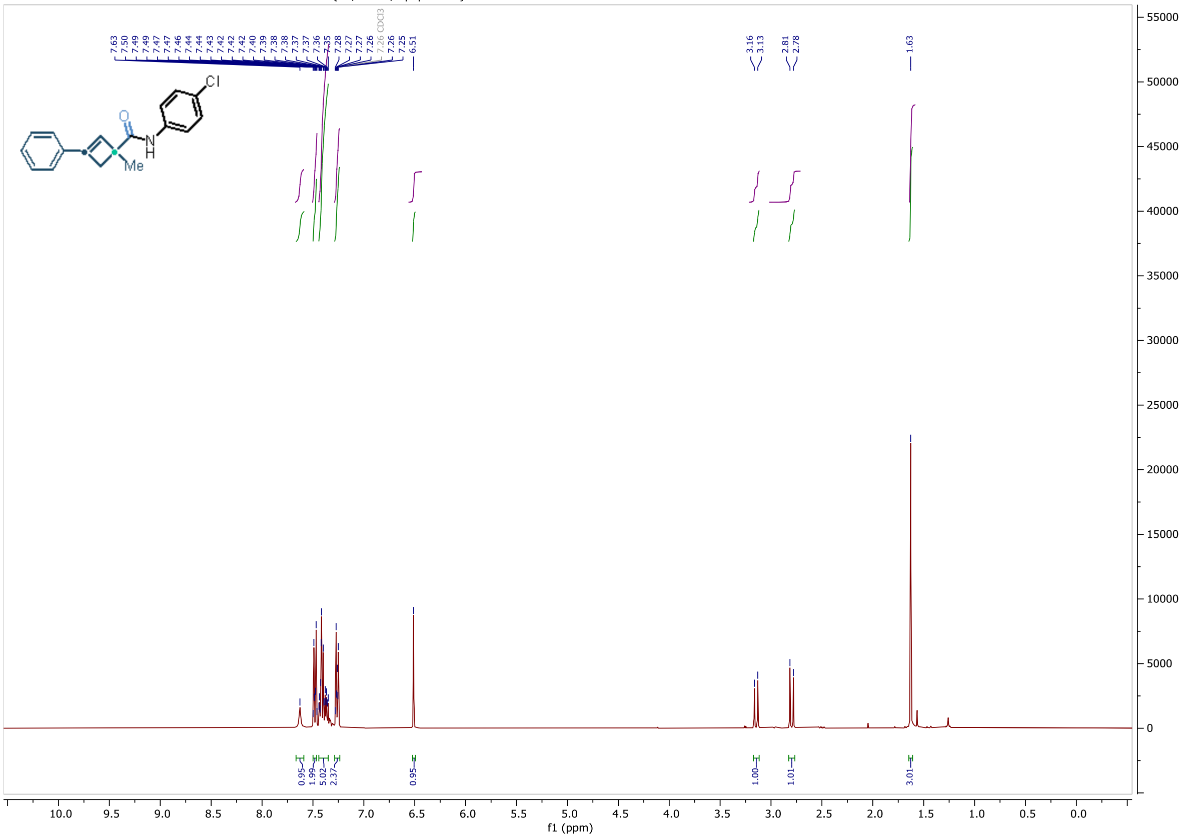

^{13}C NMR spectrum of **4g** (101 MHz, $CDCl_3$)

260217.405.11.fid — Yu-kun Liu LYK-24-2-69 — Au13C CDCl3 {C:\Bruker\TopSpin3.6.2} 2602 5

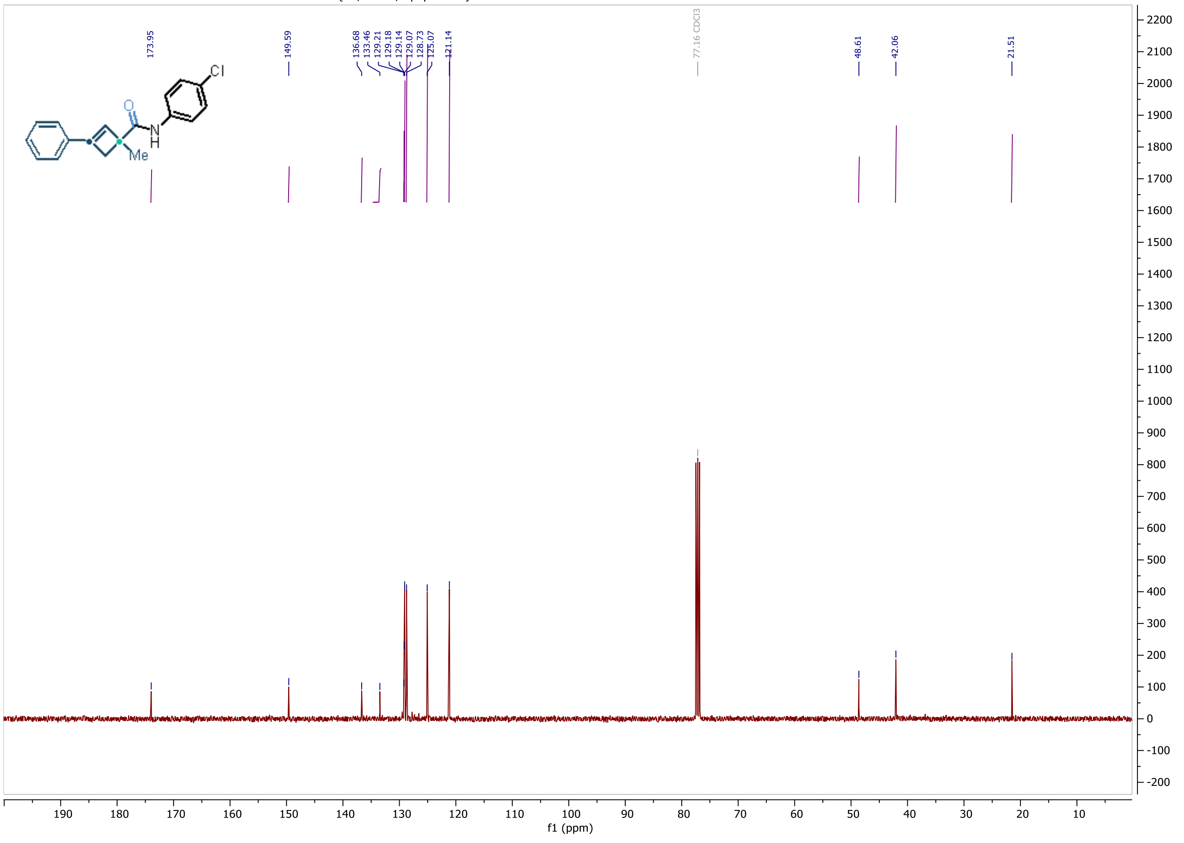

^{1}H NMR spectrum of 4**h** (400 MHz, $CDCl_3$)

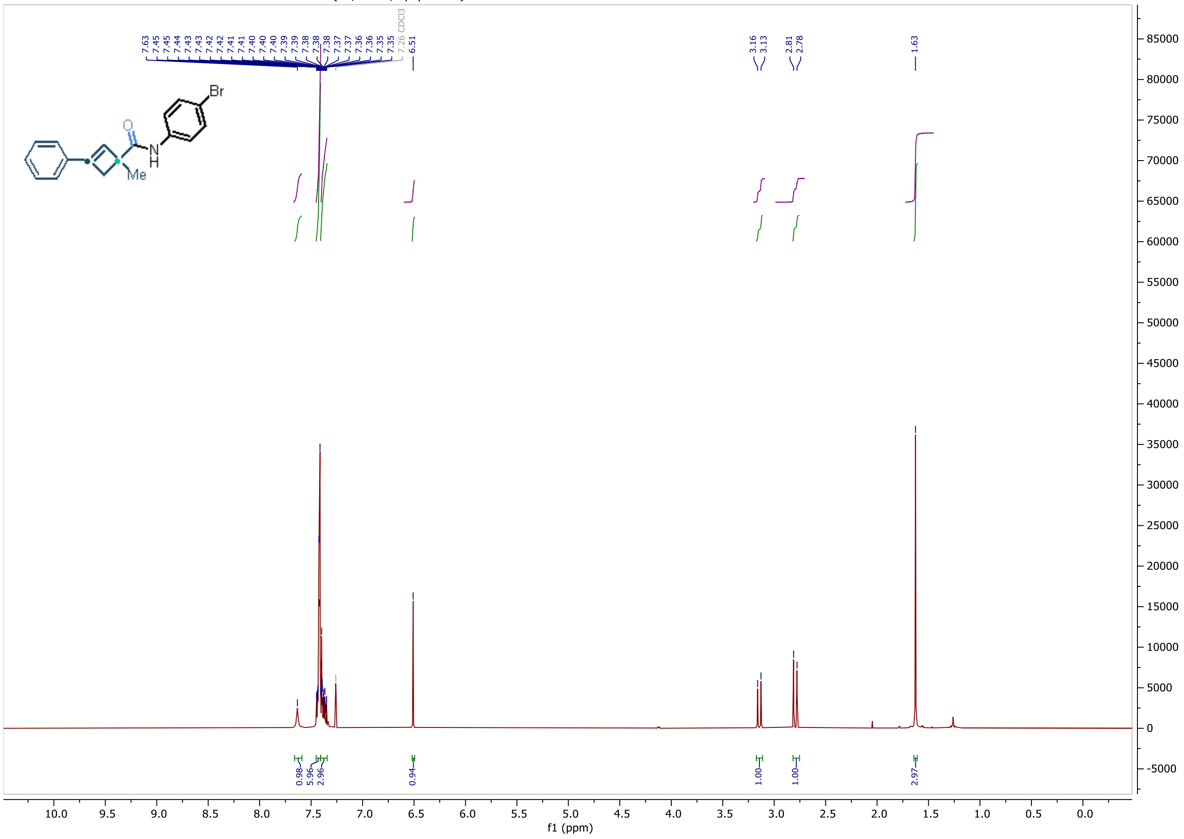

^{13}C NMR spectrum of **4h** (101 MHz, $CDCl_3$)

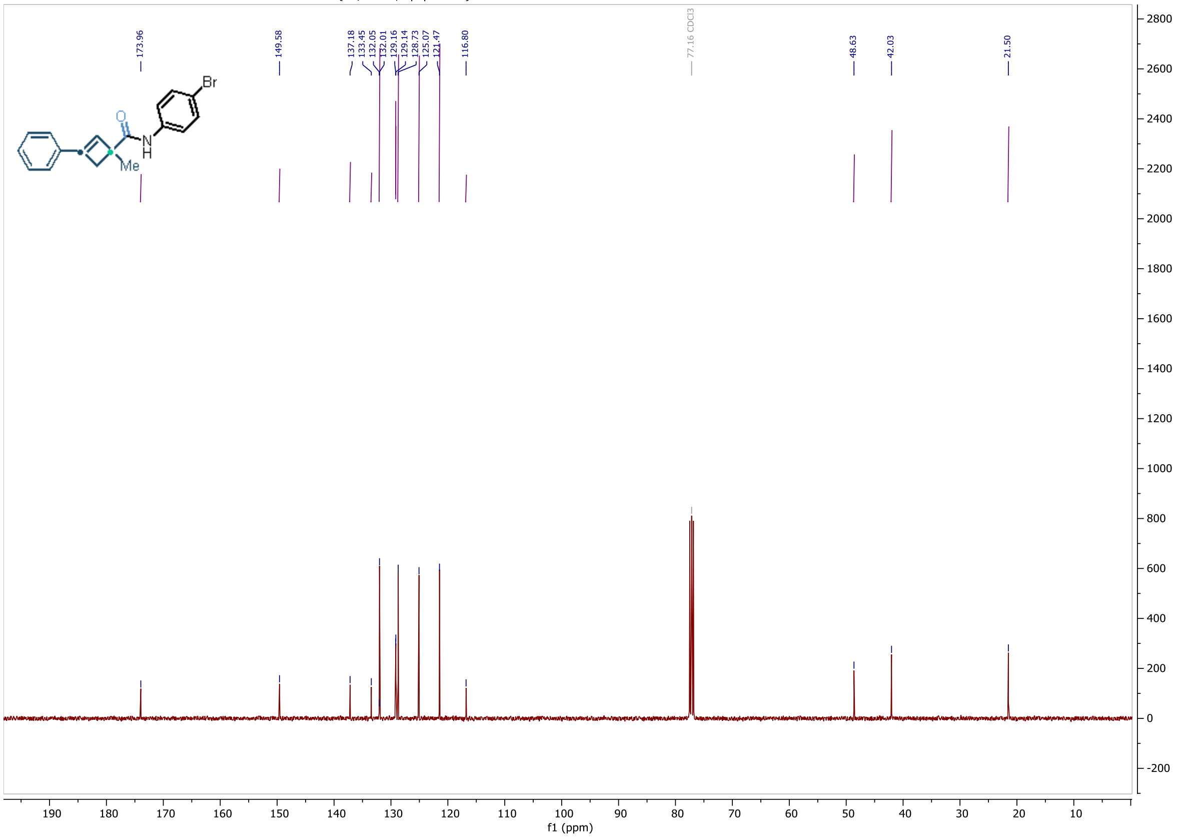

^{1}H NMR spectrum of 4**i** (300 MHz, $CDCl_3$)

260220.310.10.fid — Yu-kun Liu LYK-24-2-77 — Au1H CDCl3 {C:\Bruker\TopSpin3.6.2} 2602 10

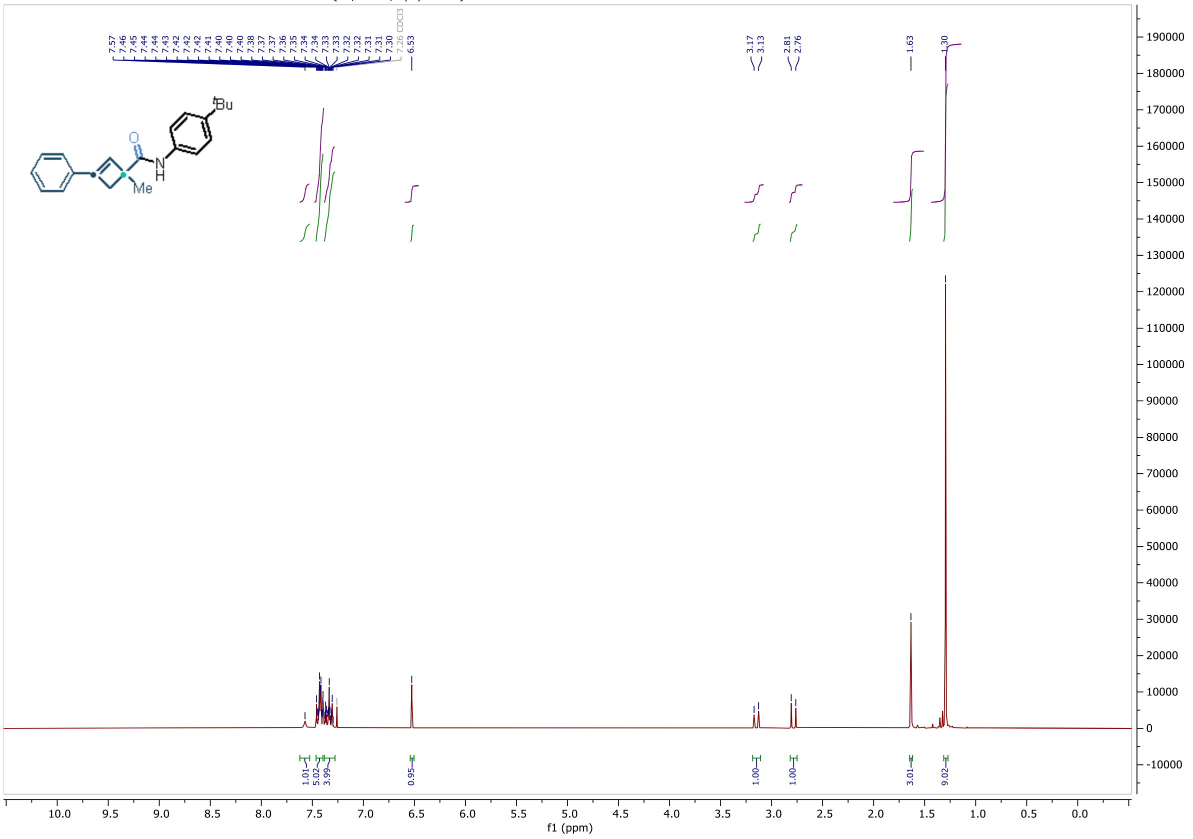

^{13}C NMR spectrum of **4i** (75 MHz, $CDCl_3$)

260220.310.11.fid — Yu-kun Liu LYK-24-2-77 — Au13C CDCl3 {C:\Bruker\TopSpin3.6.2} 2602 10

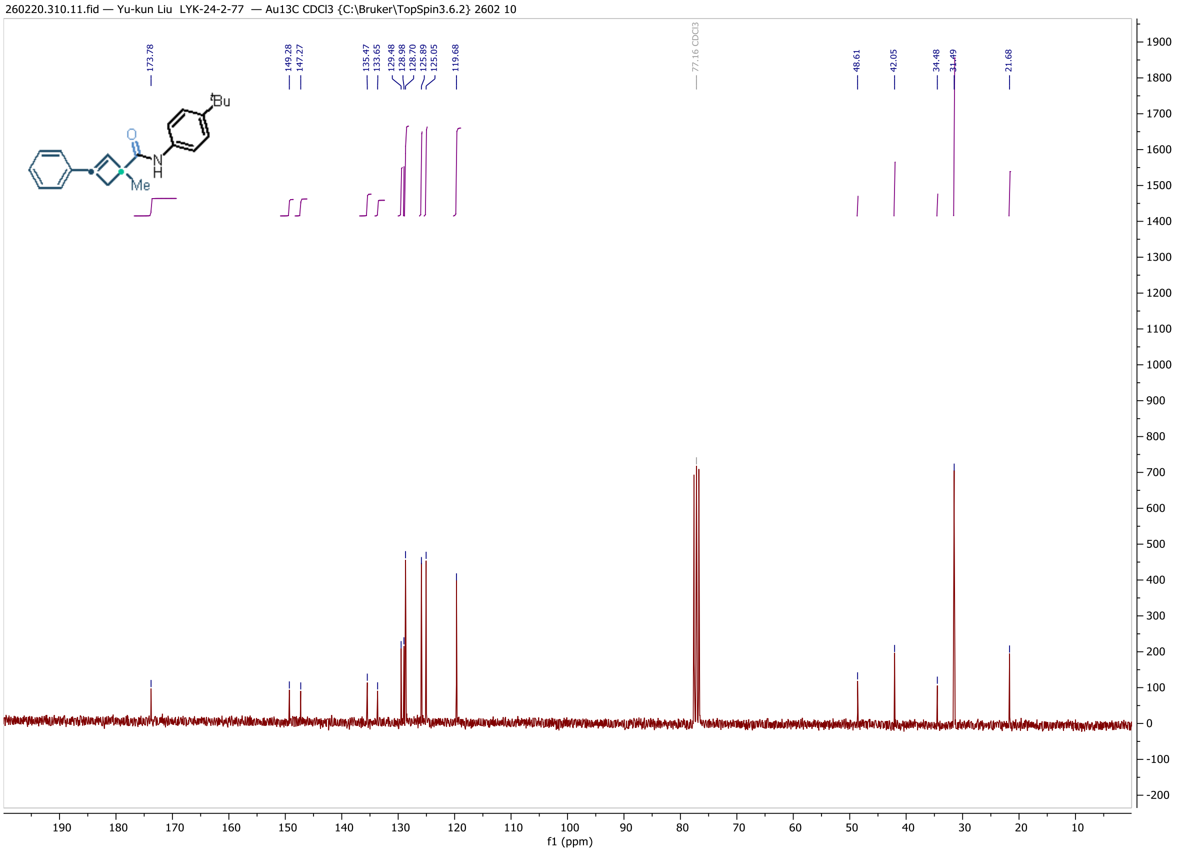

^{1}H NMR spectrum of **4j** (300 MHz, $CDCl_3$)

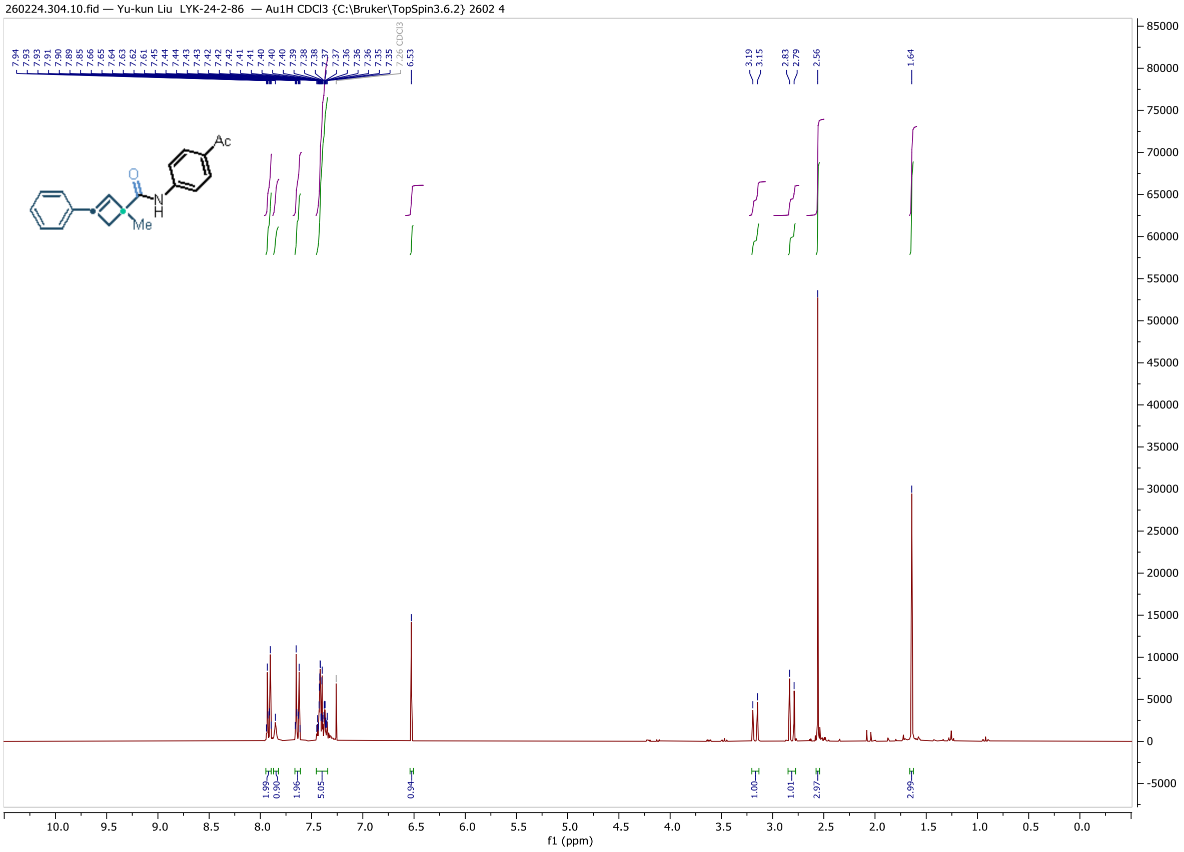

^{13}C NMR spectrum of **4j** (75 MHz, $CDCl_3$)

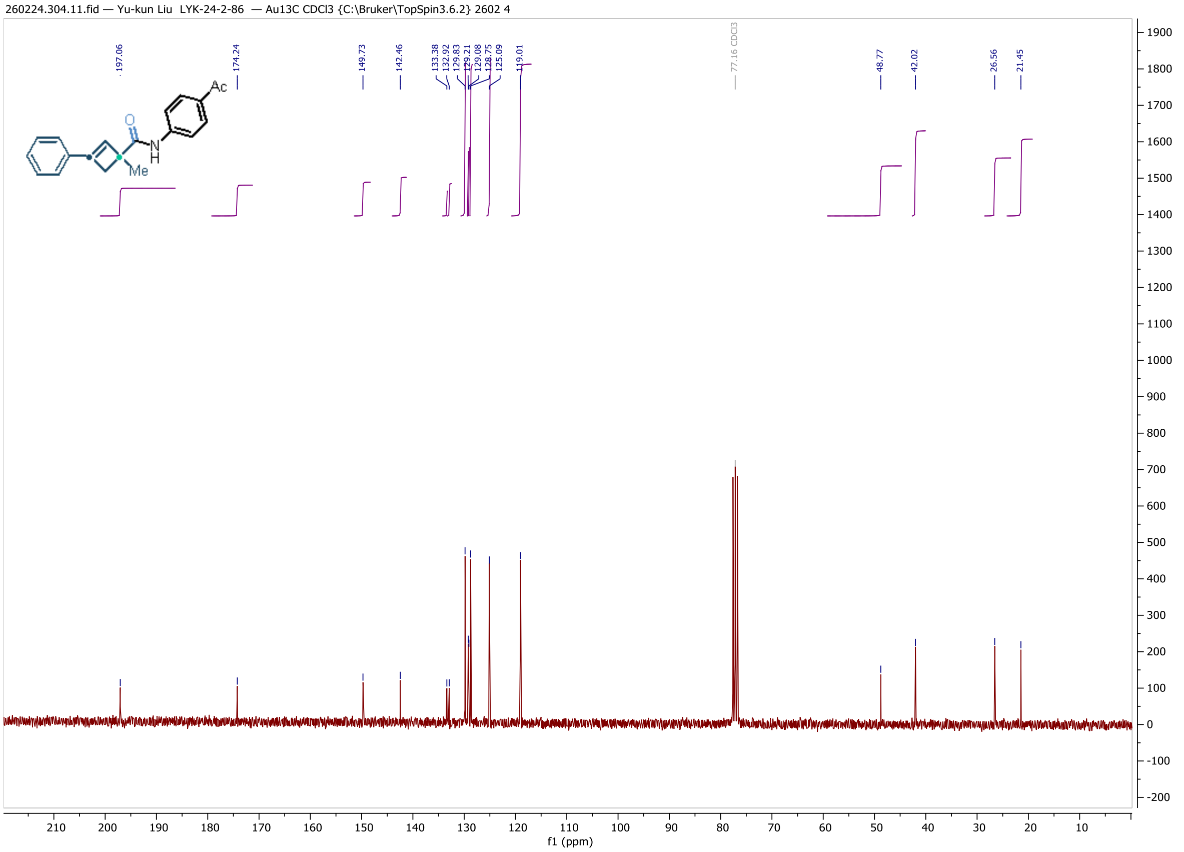

^{1}H NMR spectrum of 4**k** (300 MHz, $CDCl_3$)

260224.305.10.fid — Yu-kun Liu LYK-24-2-87 — Au1H CDCl3 {C:\Bruker\TopSpin3.6.2} 2602 5

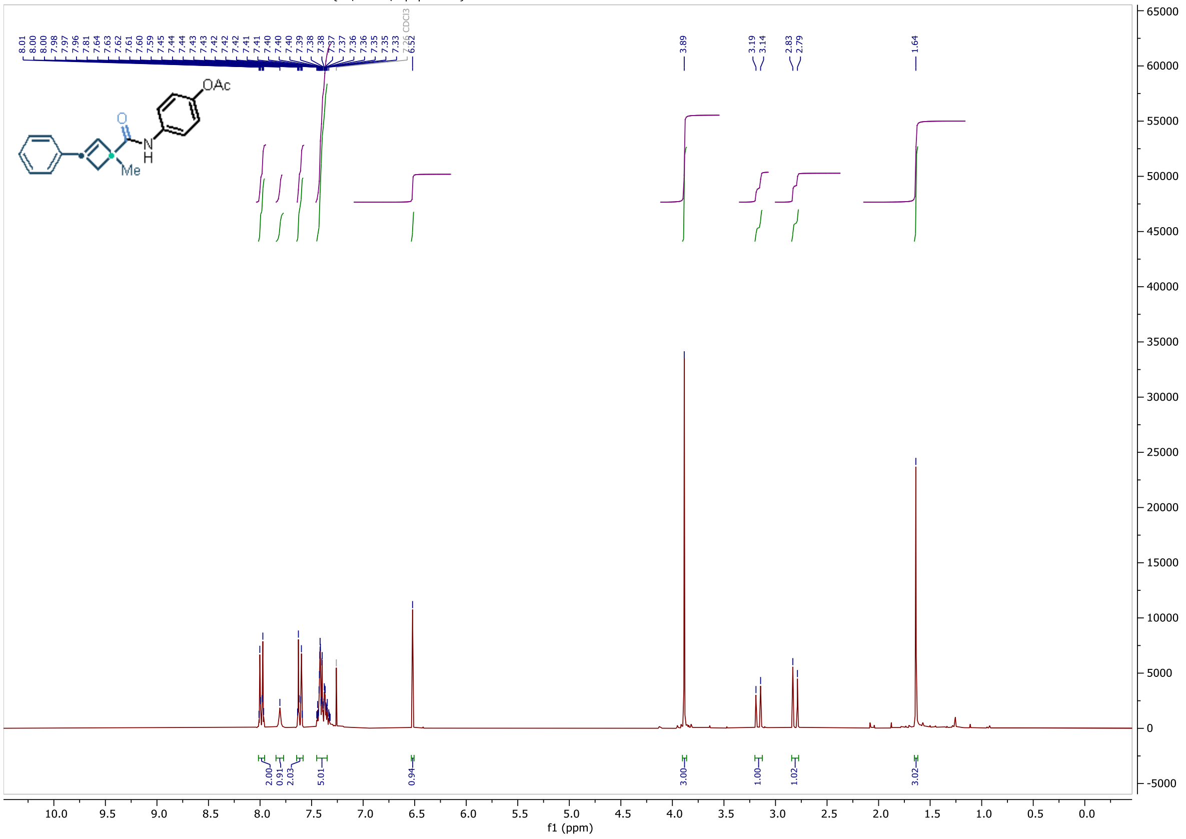

^{13}C NMR spectrum of **4k** (75 MHz, $CDCl_3$)

260224.305.11.fid — Yu-kun Liu LYK-24-2-87 — Au13C CDCl3 {C:\Bruker\TopSpin3.6.2} 2602 5

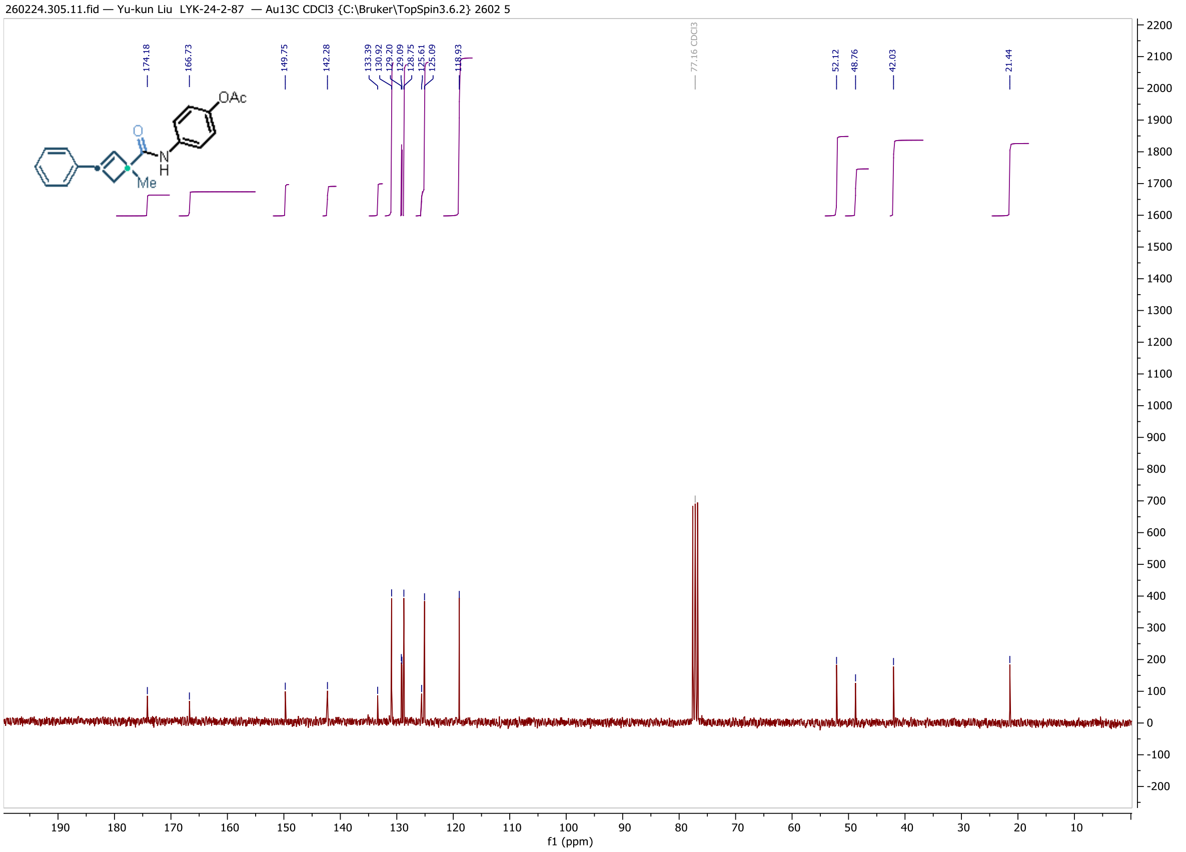

^{1}H NMR spectrum of 4**l** (300 MHz, $CDCl_3$)

260211.305.10.fid — Yu-kun Liu LYK-24-2-63 — Au1H CDCl3 {C:\Bruker\TopSpin3.6.2} 2602 5

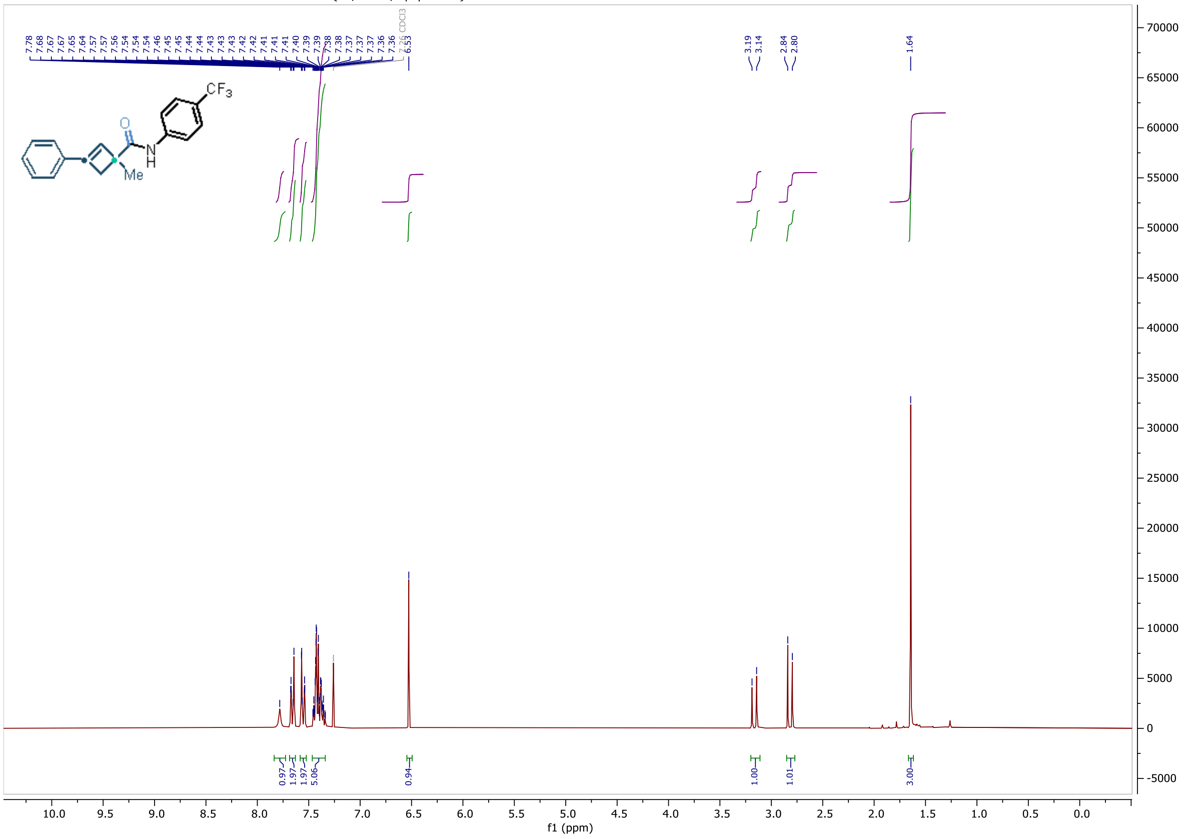

^{13}C NMR spectrum of **4l** (75 MHz, $CDCl_3$)

260211.305.11.fid — Yu-kun Liu LYK-24-2-63 — Au13C CDCl3 {C:\Bruker\TopSpin3.6.2} 2602 5

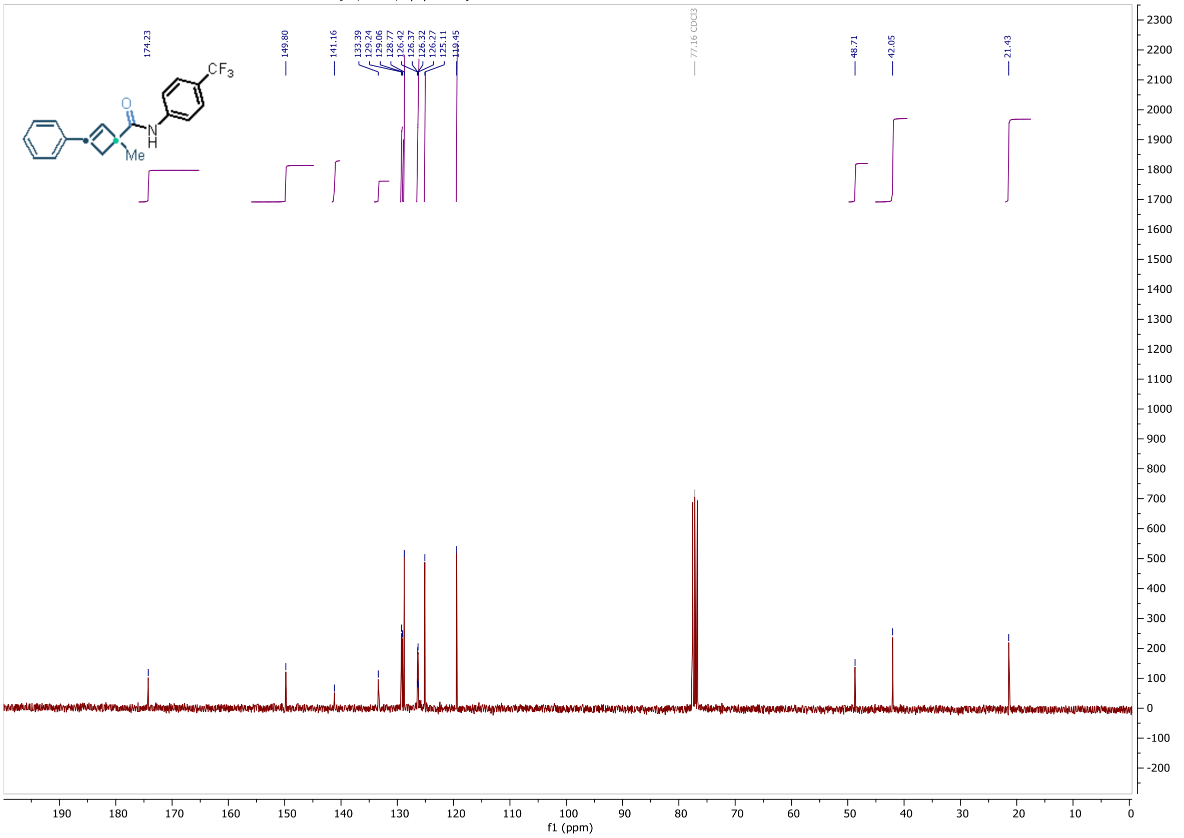

^{19}F NMR spectrum of **4l** (282 MHz, $CDCl_3$)

260211.305.12.fid — Yu-kun Liu LYK-24-2-63 — Au19F CDCl3 {C:\Bruker\TopSpin3.6.2} 2602 5

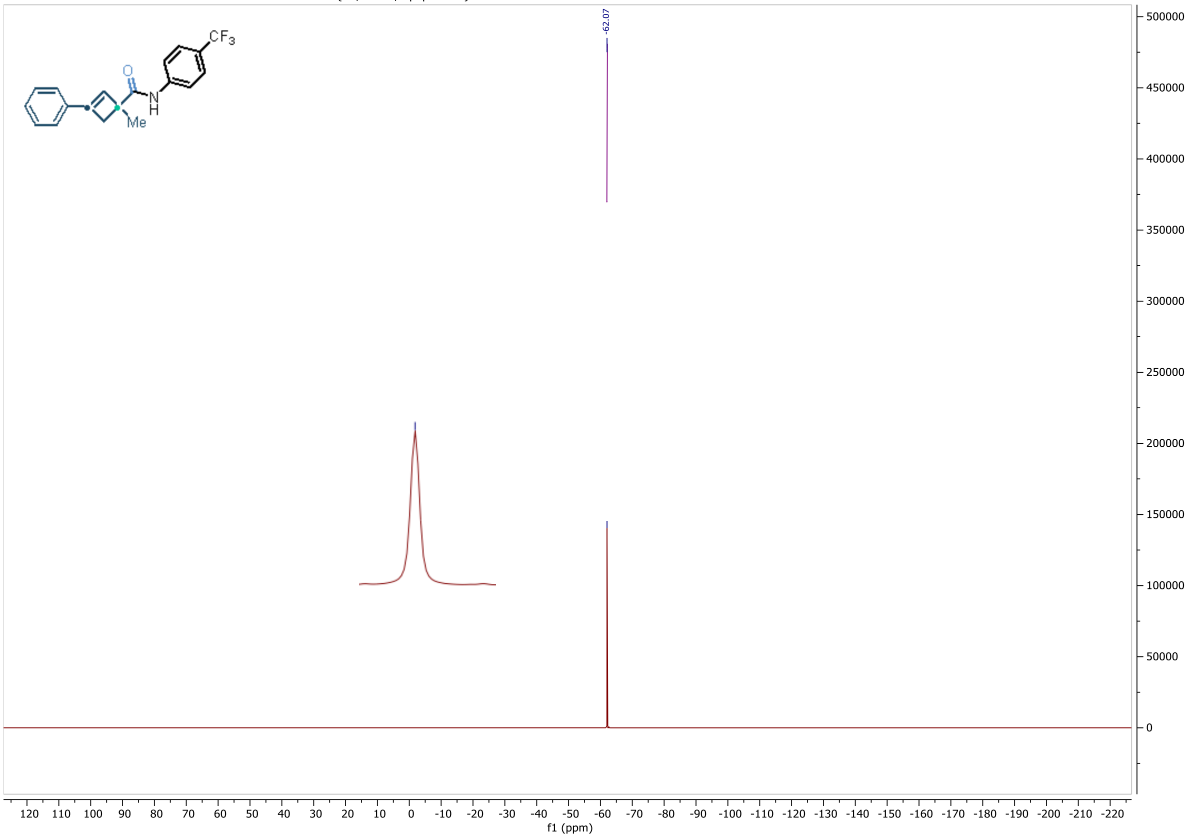

^{1}H NMR spectrum of 4**m** (300 MHz, $CDCl_3$)

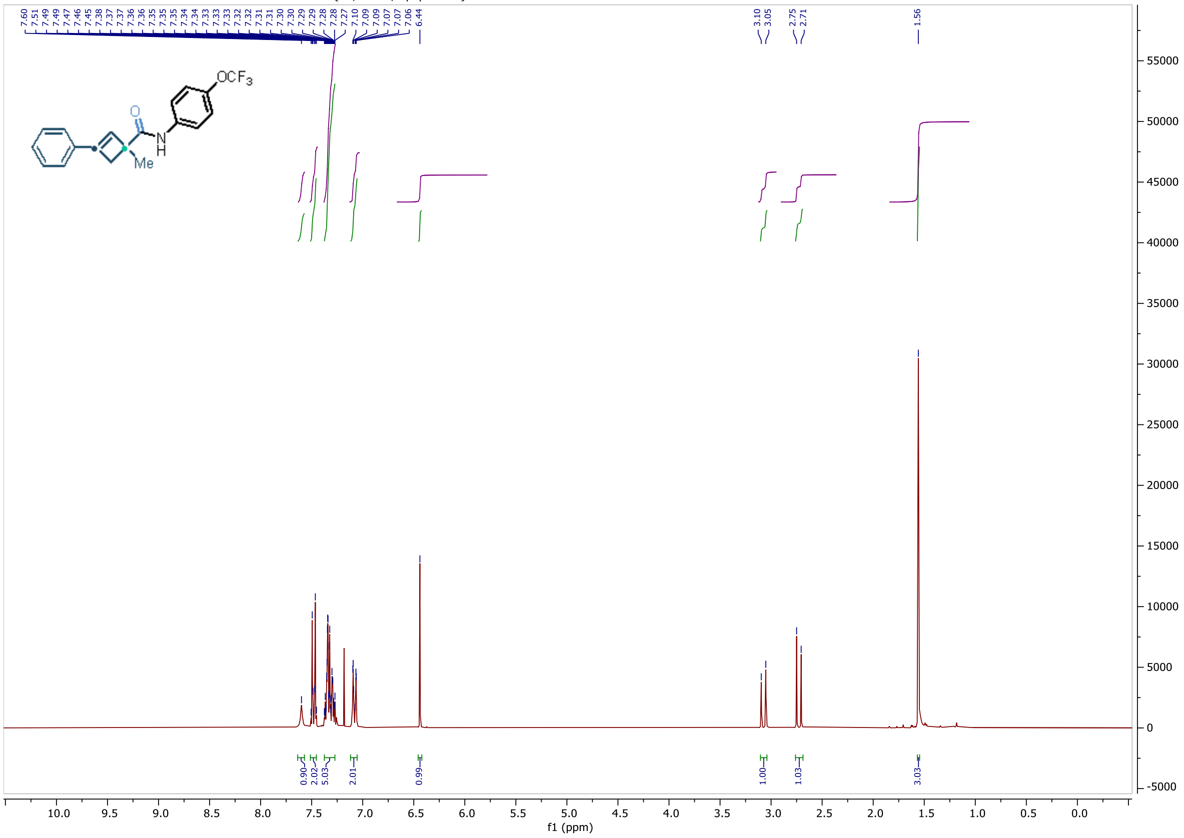

^{13}C NMR spectrum of **4m** (75 MHz, $CDCl_3$)

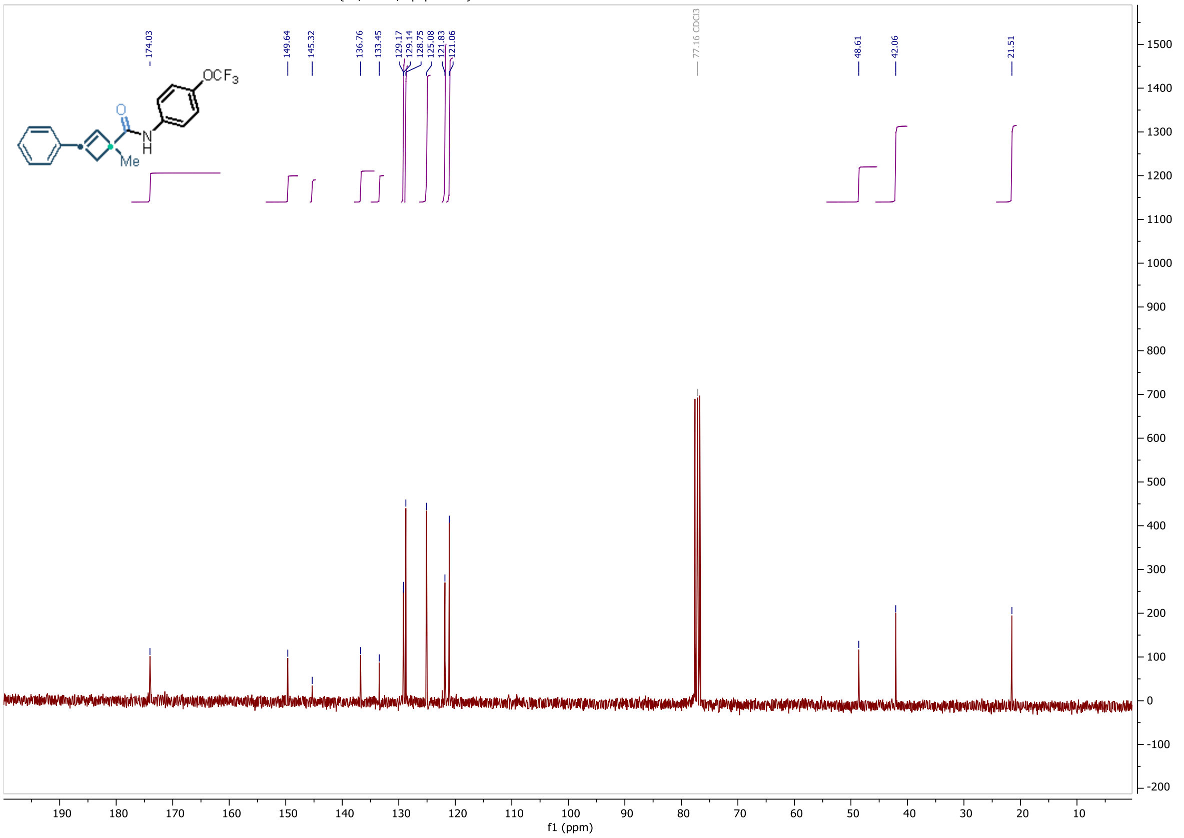

^{19}F NMR spectrum of **4m** (282 MHz, $CDCl_3$)

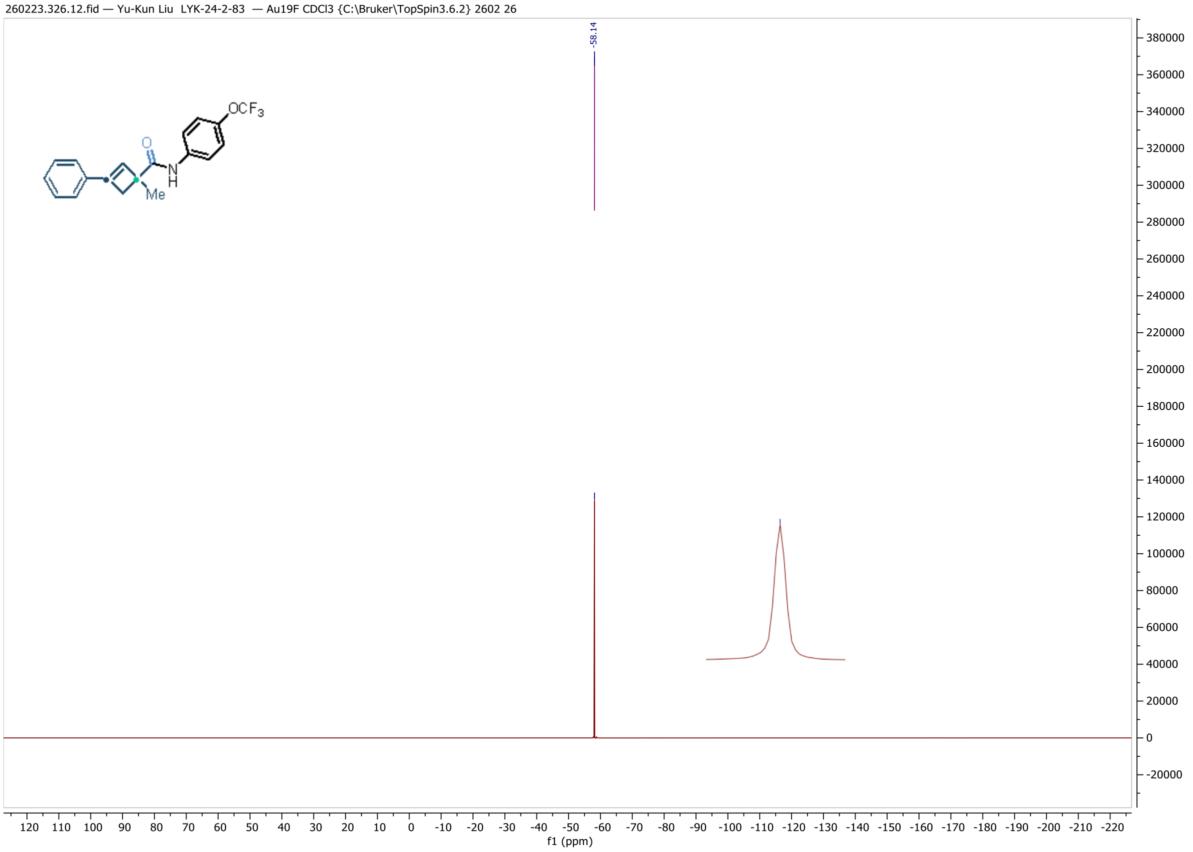

^{1}H NMR spectrum of 4**n** (300 MHz, $CDCl_3$)

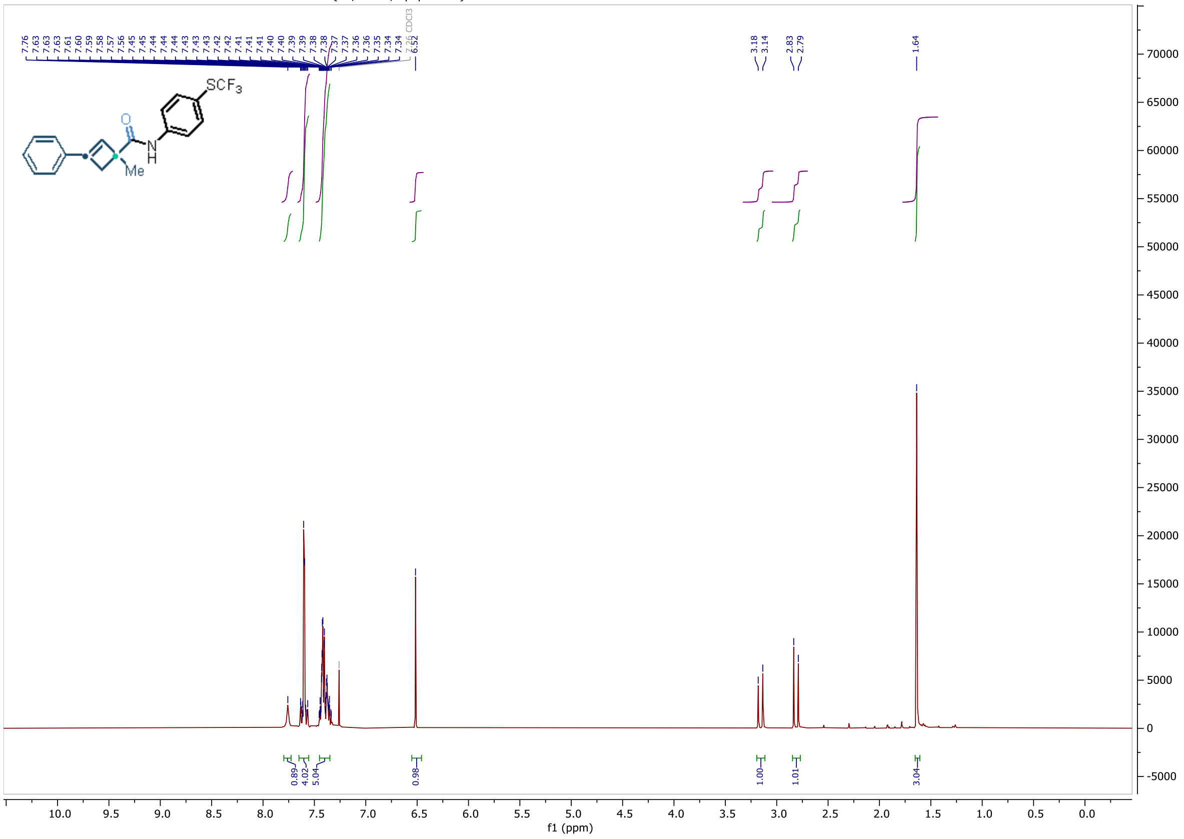

^{13}C NMR spectrum of **4n** (75 MHz, $CDCl_3$)

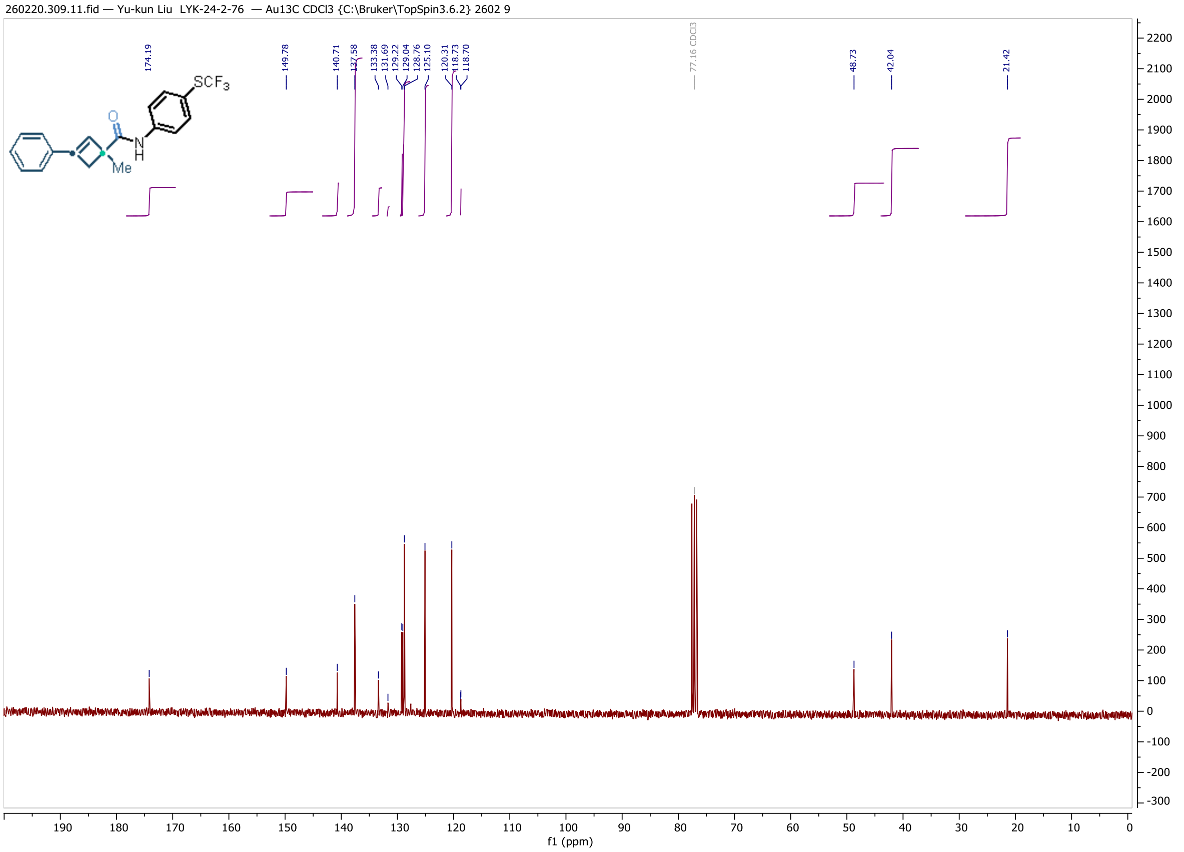

^{19}F NMR spectrum of **4n** (282 MHz, $CDCl_3$)

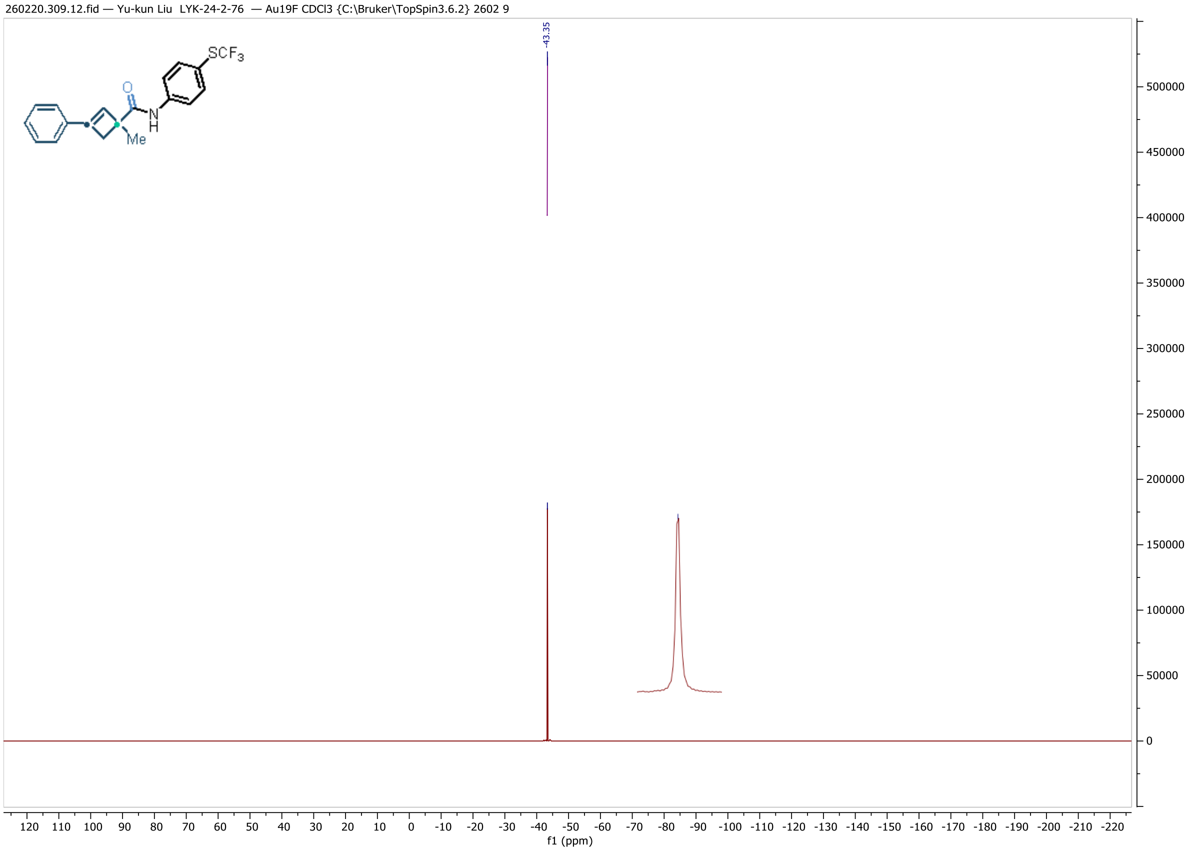

^{1}H NMR spectrum of **4o** (300 MHz, $CDCl_3$)

260225.f311.10.fid — Yu-Kun Liu LYK-24-2-91 — Au1H CDCl3 {C:\Bruker\TopSpin3.6.2} 2602 11

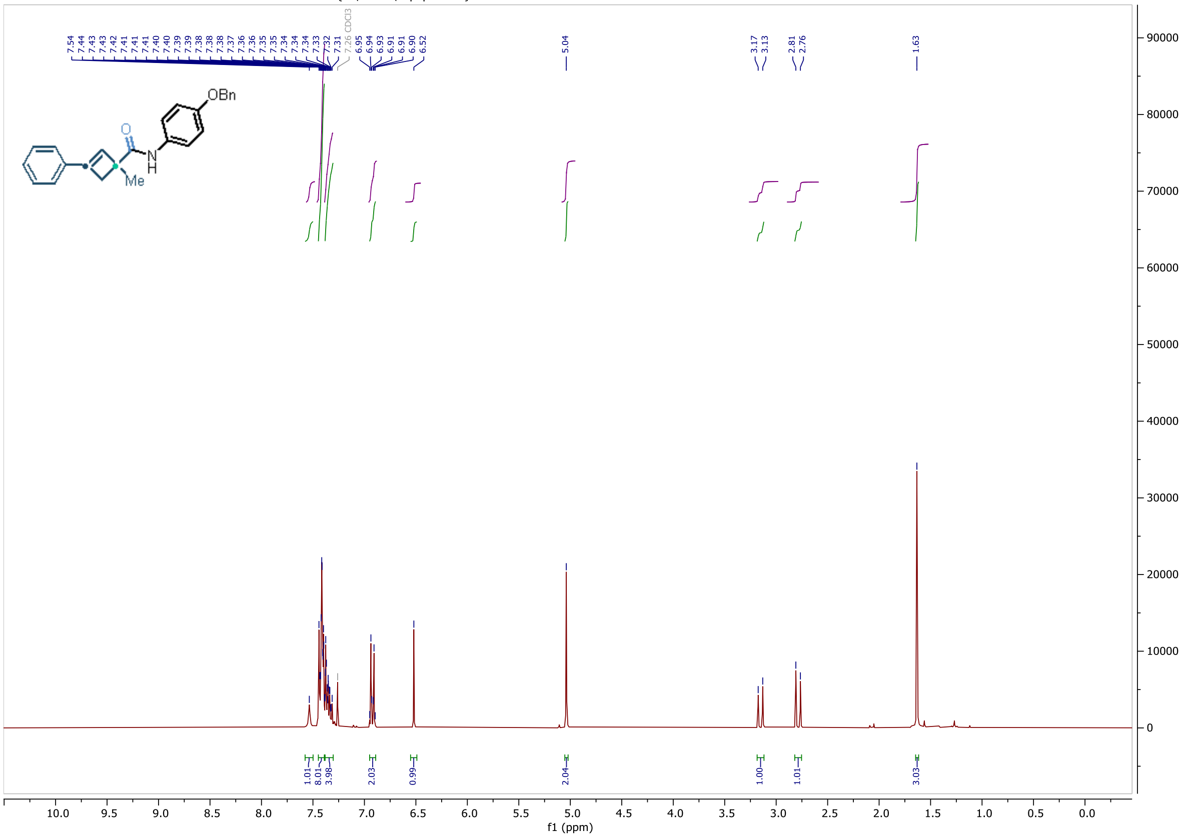

^{13}C NMR spectrum of **4o** (75 MHz, $CDCl_3$)

260225.f311.11.fid — Yu-Kun Liu LYK-24-2-91 — Au13C CDCl3 {C:\Bruker\TopSpin3.6.2} 2602 11

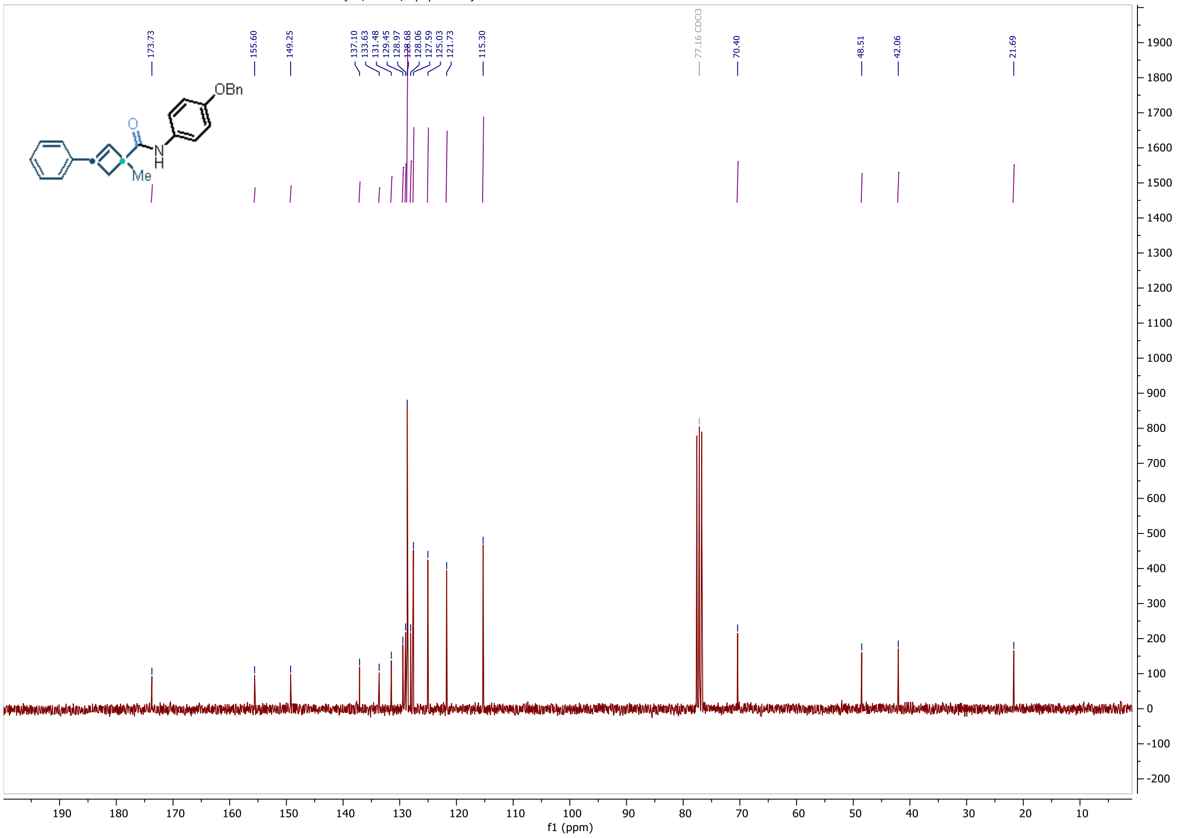

^{1}H NMR spectrum of **4p** (300 MHz, $CDCl_3$)

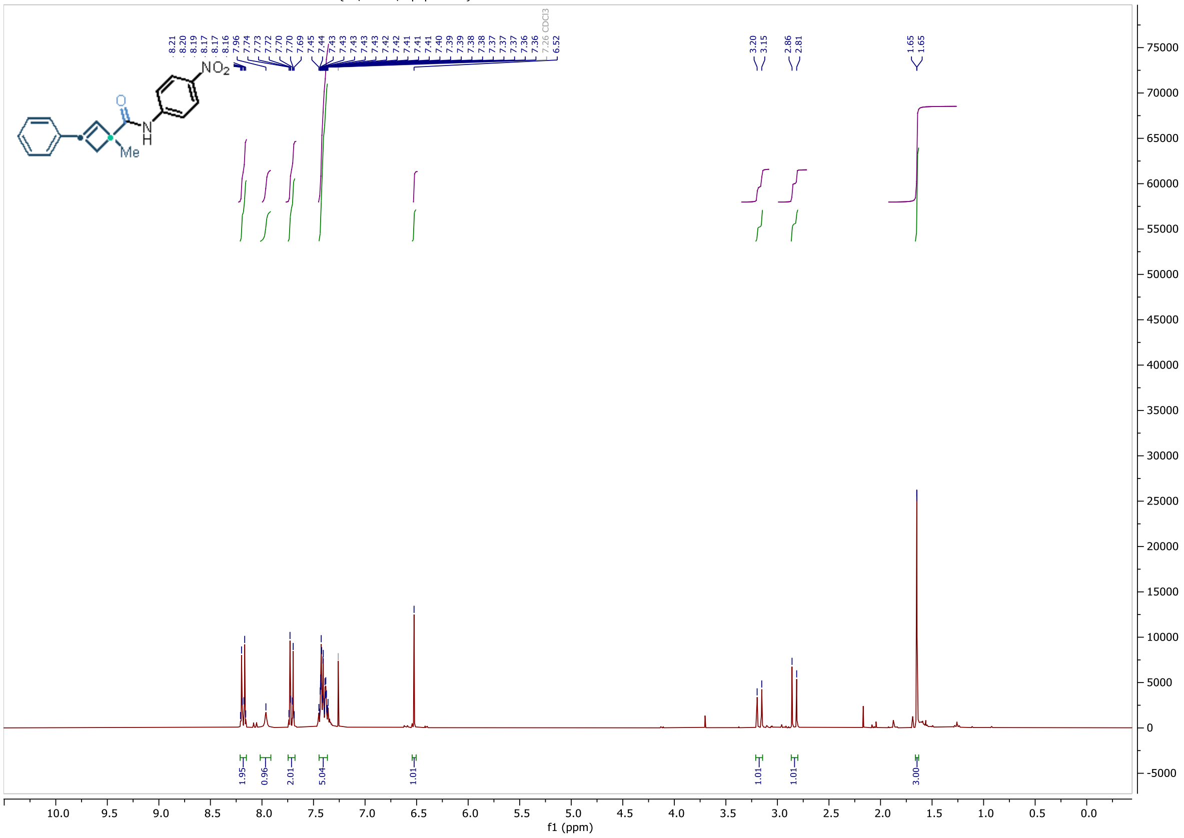

^{13}C NMR spectrum of **4p** (75 MHz, $CDCl_3$)

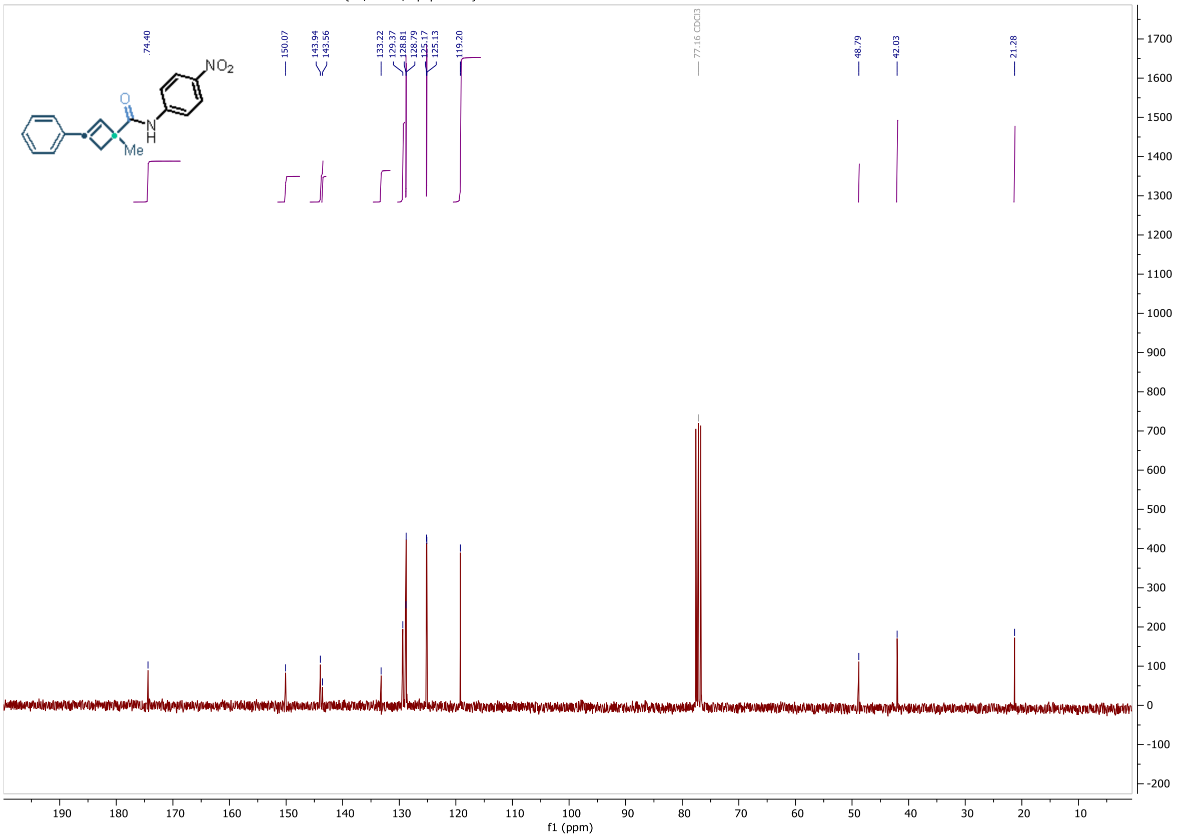

^{1}H NMR spectrum of **4q** (300 MHz, $CDCl_3$)

260220.308.10.fid — Yu-kun Liu LYK-24-2-75 — Au1H CDCl3 {C:\Bruker\TopSpin3.6.2} 2602 8

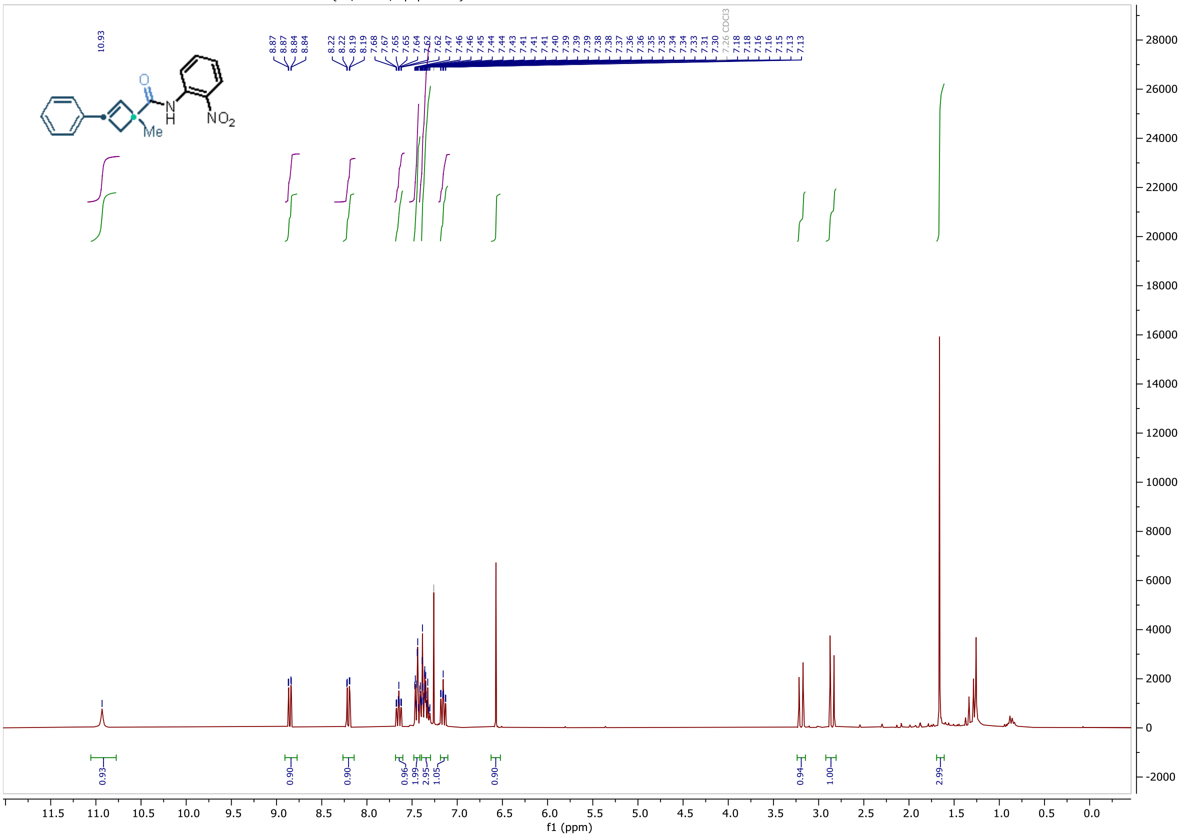

^{13}C NMR spectrum of **4q** (75 MHz, $CDCl_3$)

260220.308.11.fid — Yu-kun Liu LYK-24-2-75 — Au13C CDCl3 {C:\Bruker\TopSpin3.6.2} 2602 8

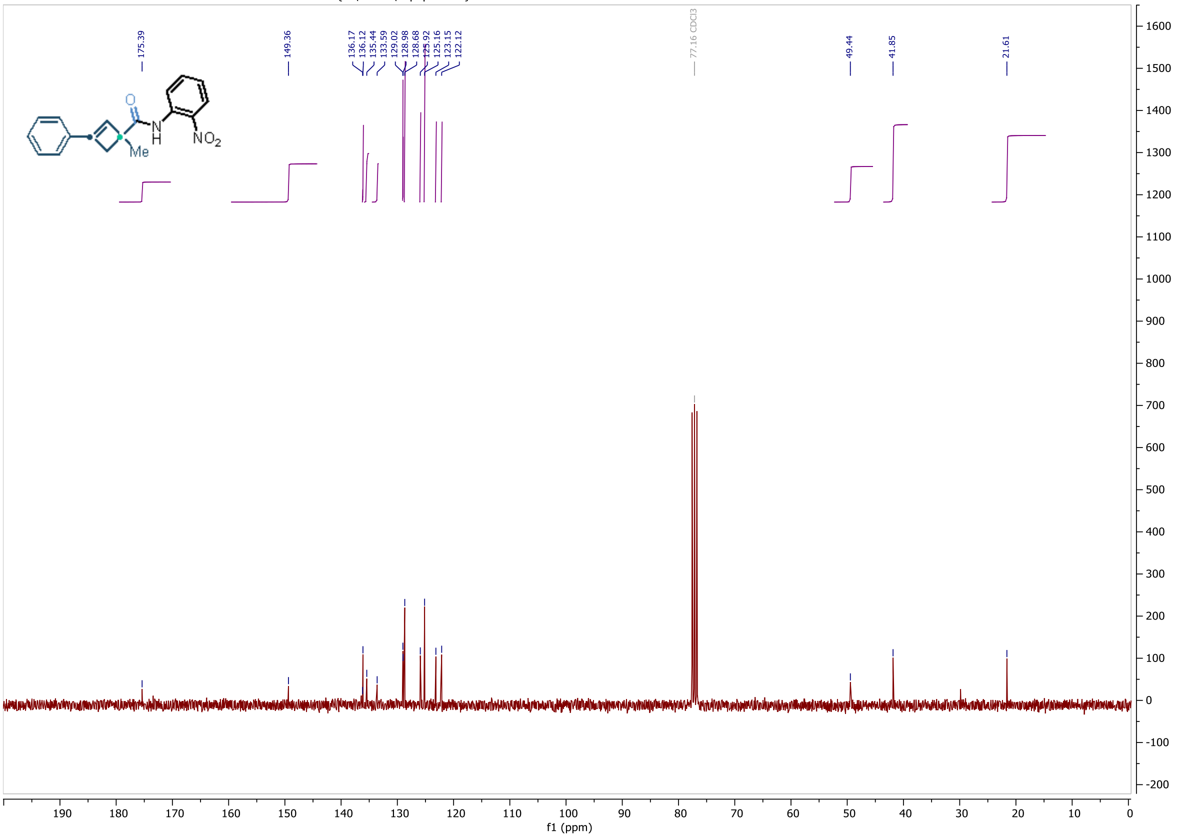

^{1}H NMR spectrum of **4r** (300 MHz, $CDCl_3$)

260226.318.10.fid — Yu-kun Liu LYK-24-2-92 — Au1H CDCl3 {C:\Bruker\TopSpin3.6.2} 2602 18

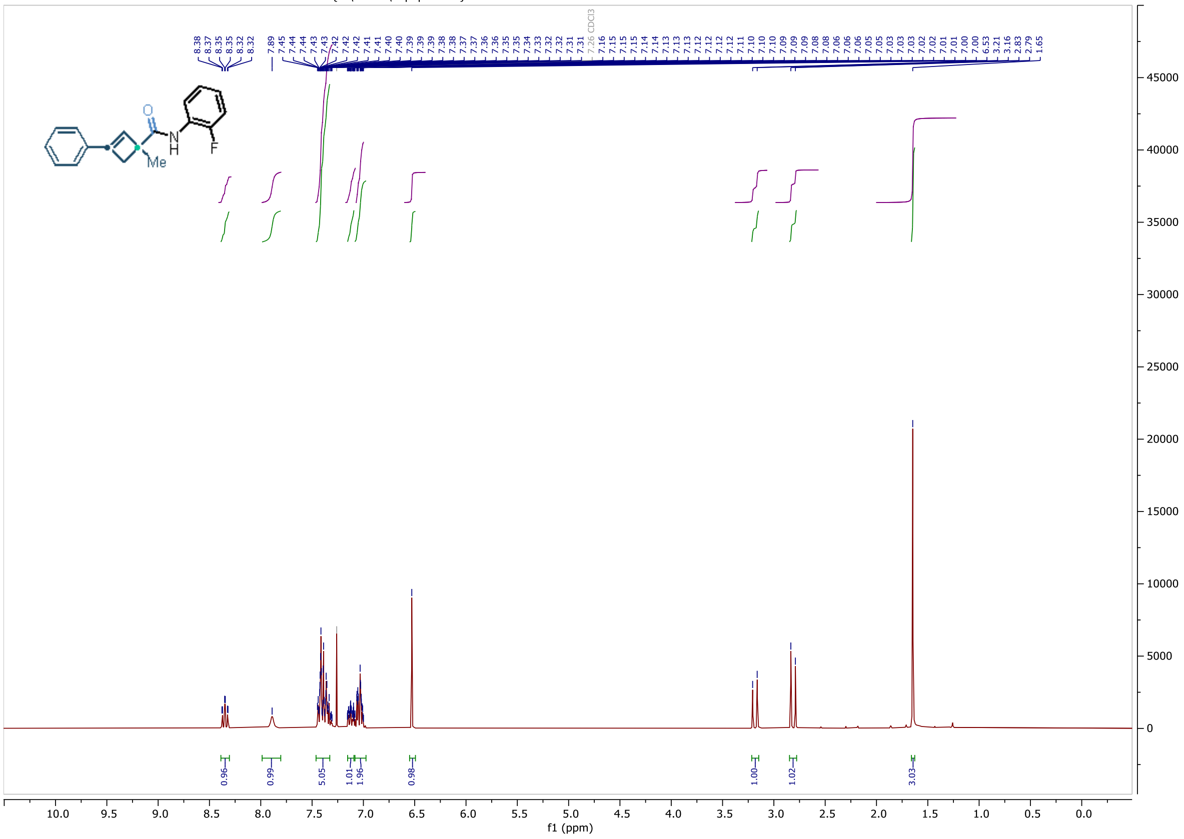

^{13}C NMR spectrum of **4r** (75 MHz, $CDCl_3$)

260226.318.11.fid — Yu-kun Liu LYK-24-2-92 — Au13C CDCl3 {C:\Bruker\TopSpin3.6.2} 2602 18

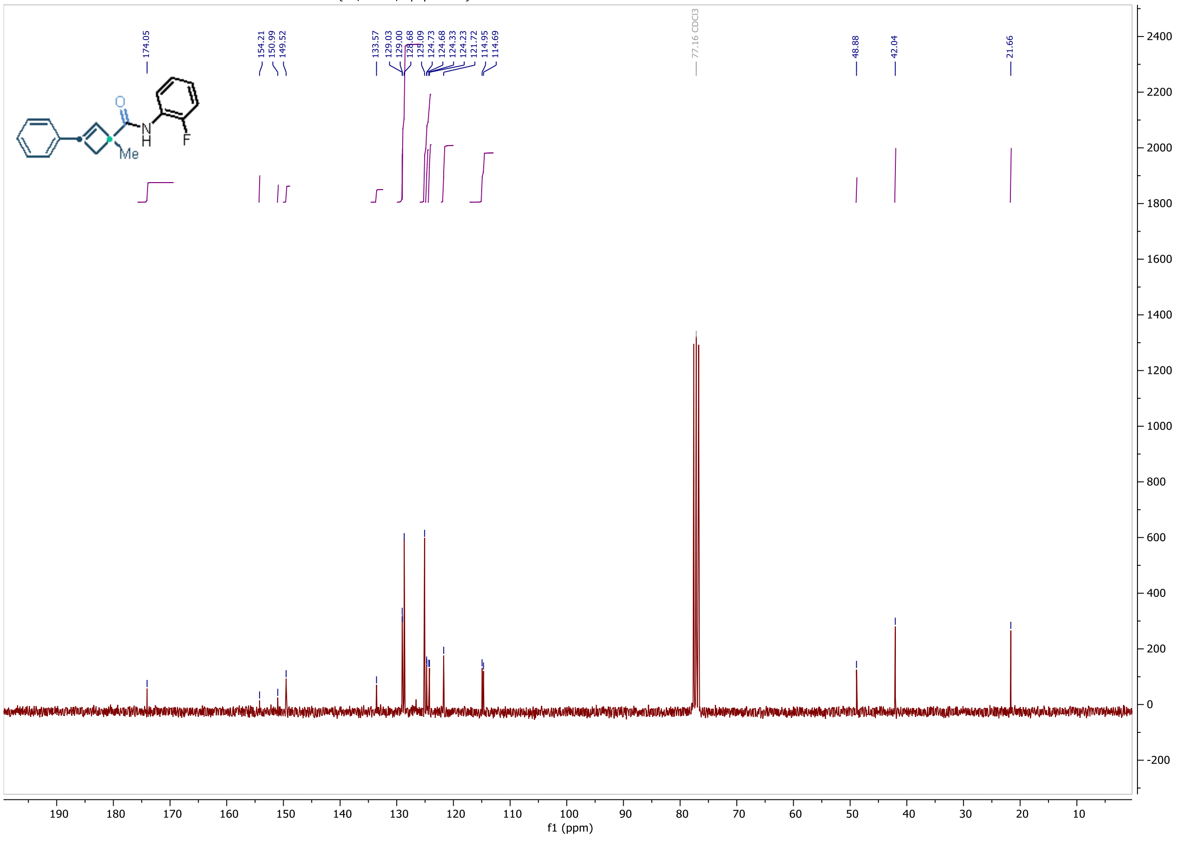

^{19}F NMR spectrum of **4r** (282 MHz, $CDCl_3$)

260226.318.12.fid — Yu-kun Liu LYK-24-2-92 — Au19F CDCl3 {C:\Bruker\TopSpin3.6.2} 2602 18

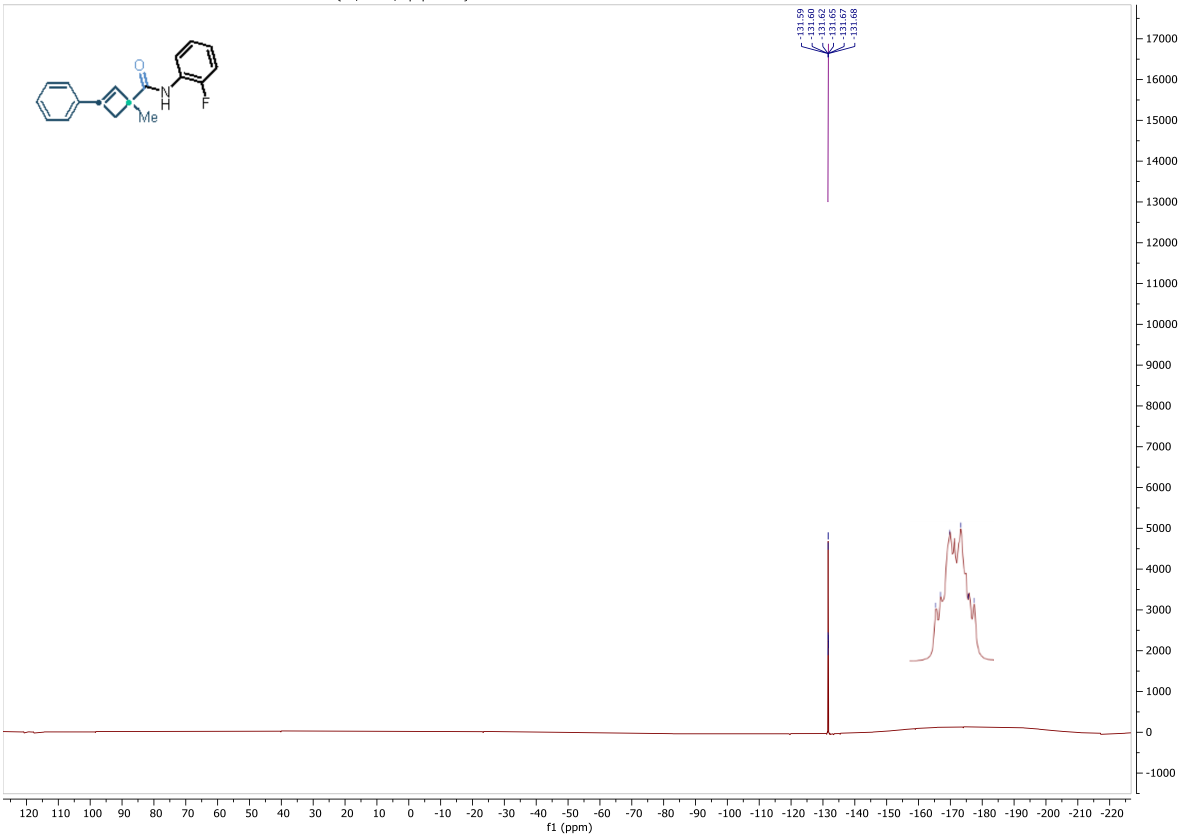

^{1}H NMR spectrum of **4s** (300 MHz, $CDCl_3$)

260326.344.10.fid — Yu-kun Liu, LYK-24-2-118 — Au1H CDCl3 {C:\Bruker\TopSpin3.6.2} 2603 44

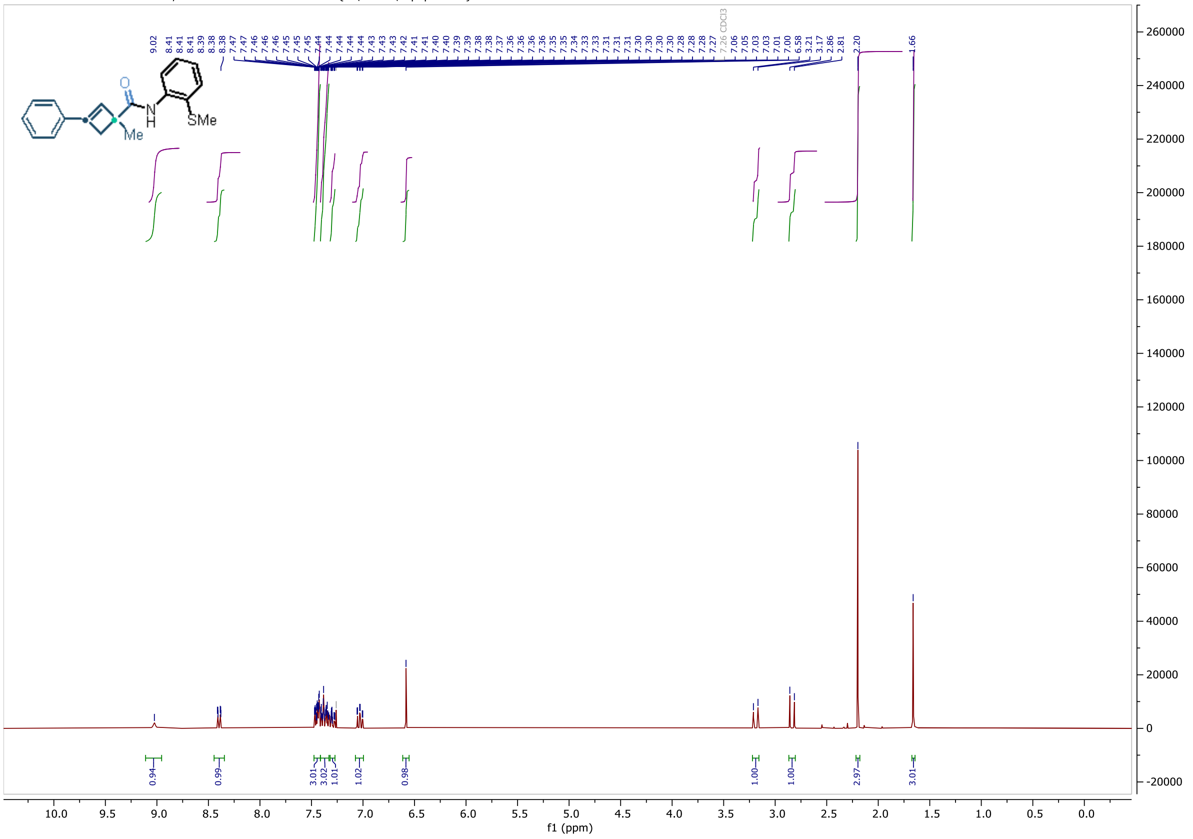

^{13}C NMR spectrum of **4s** (75 MHz, $CDCl_3$)

260326.344.11.fid — Yu-kun Liu, LYK-24-2-118 — Au13C CDCl3 {C:\Bruker\TopSpin3.6.2} 2603 44

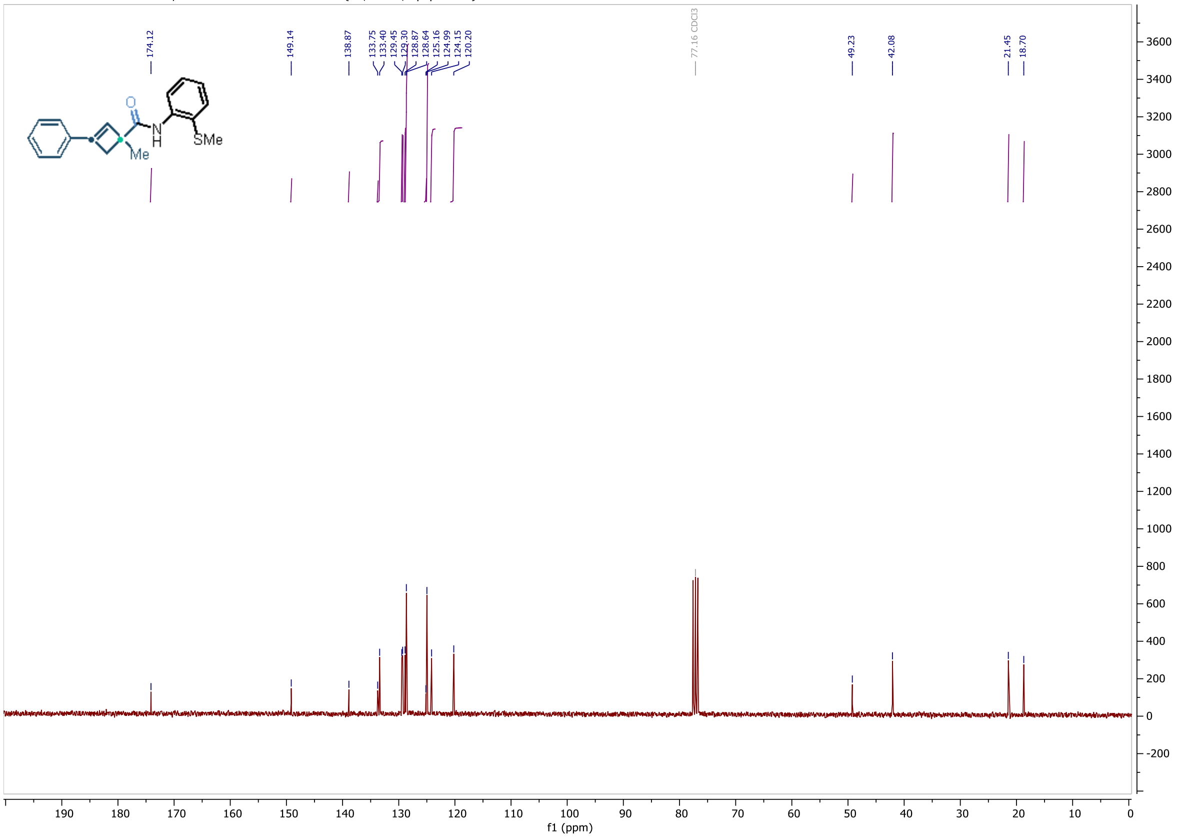

^{1}H NMR spectrum of **4t** (300 MHz, $CDCl_3$)

260326.345.10.fid — Yu-kun Liu, LYK-24-2-119 — Au1H CDCl3 {C:\Bruker\TopSpin3.6.2} 2603 45

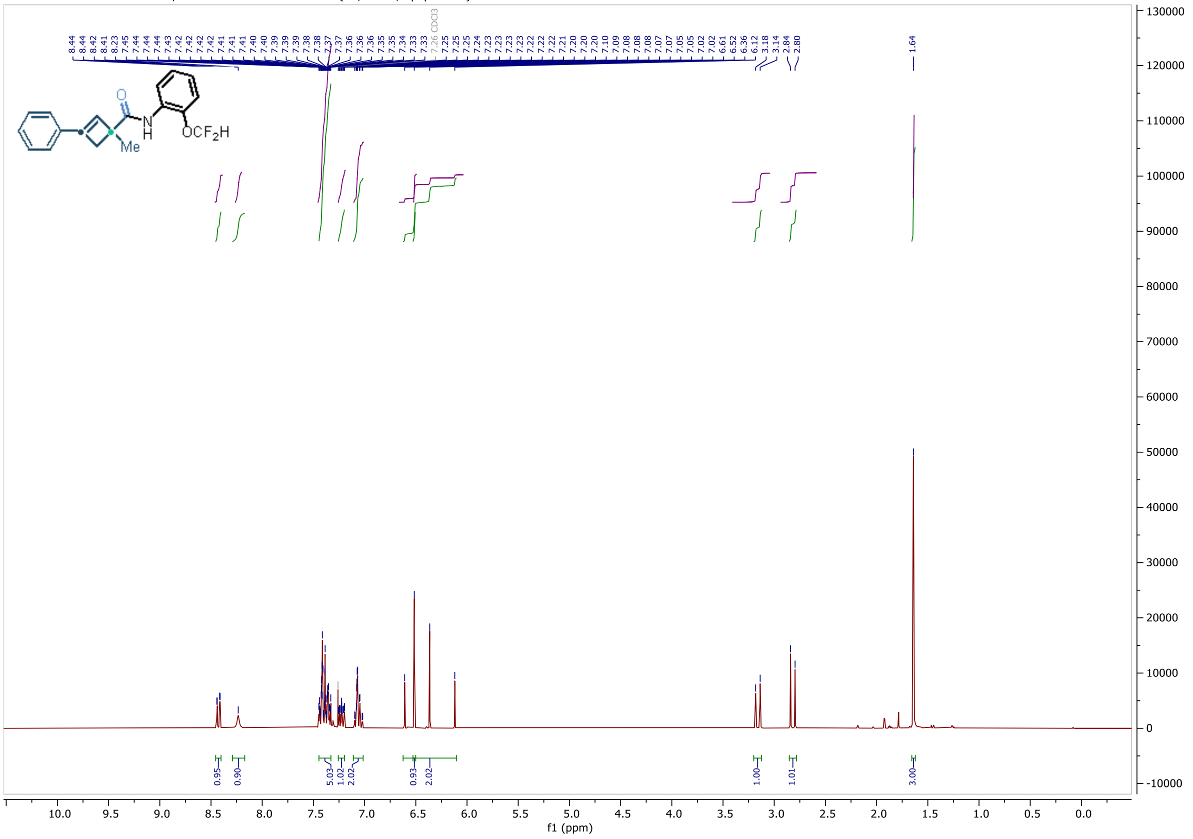

^{13}C NMR spectrum of **4t** (75 MHz, $CDCl_3$)

260326.345.11.fid — Yu-kun Liu, LYK-24-2-119 — Au13C CDCl3 {C:\Bruker\TopSpin3.6.2} 2603 45

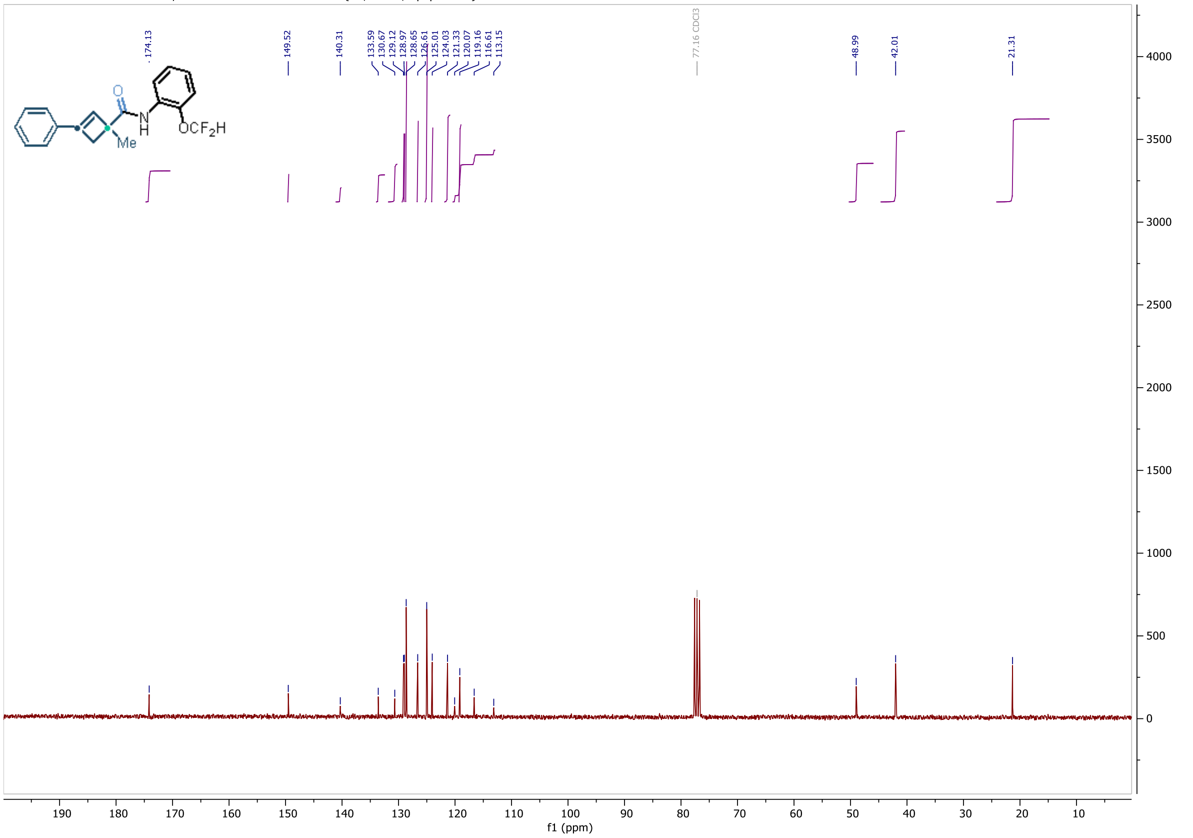

^{19}F NMR spectrum of **4u** (282 MHz, $CDCl_3$)

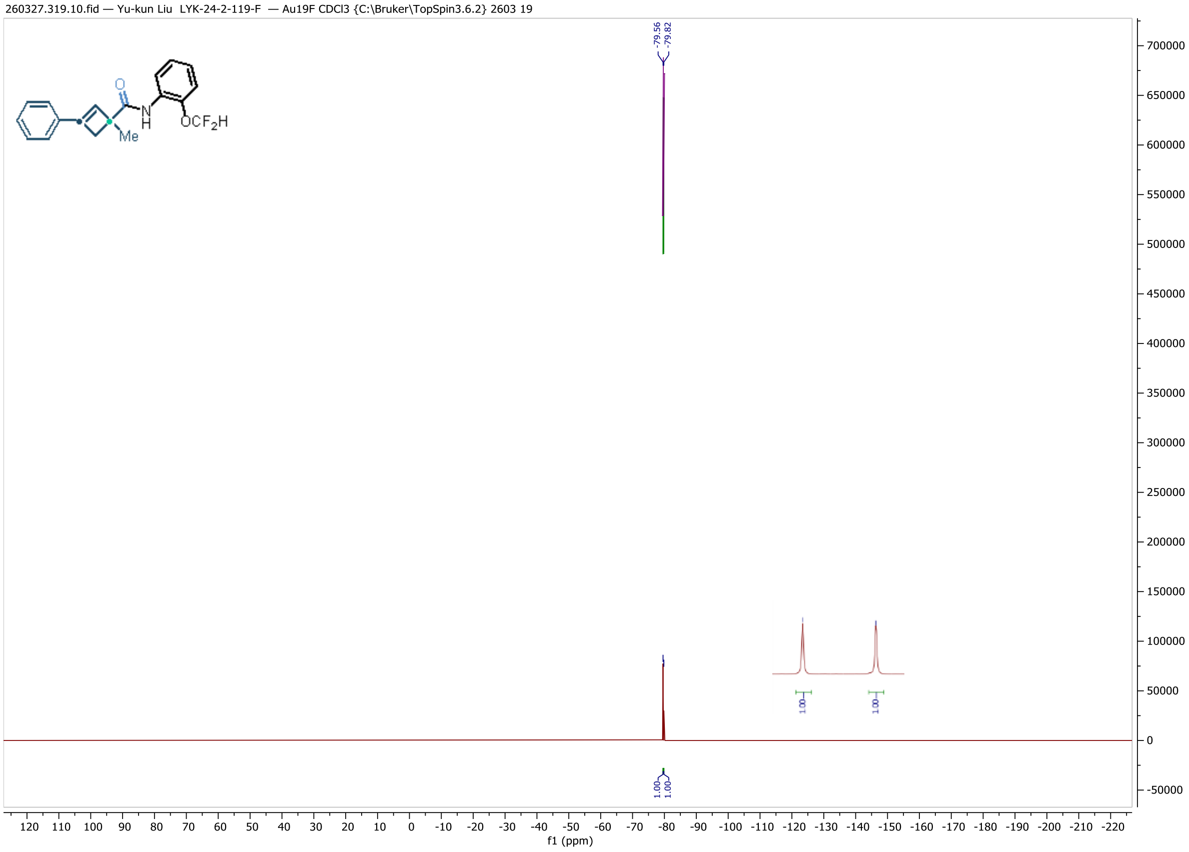

^{1}H NMR spectrum of **4u** (300 MHz, $CDCl_3$)

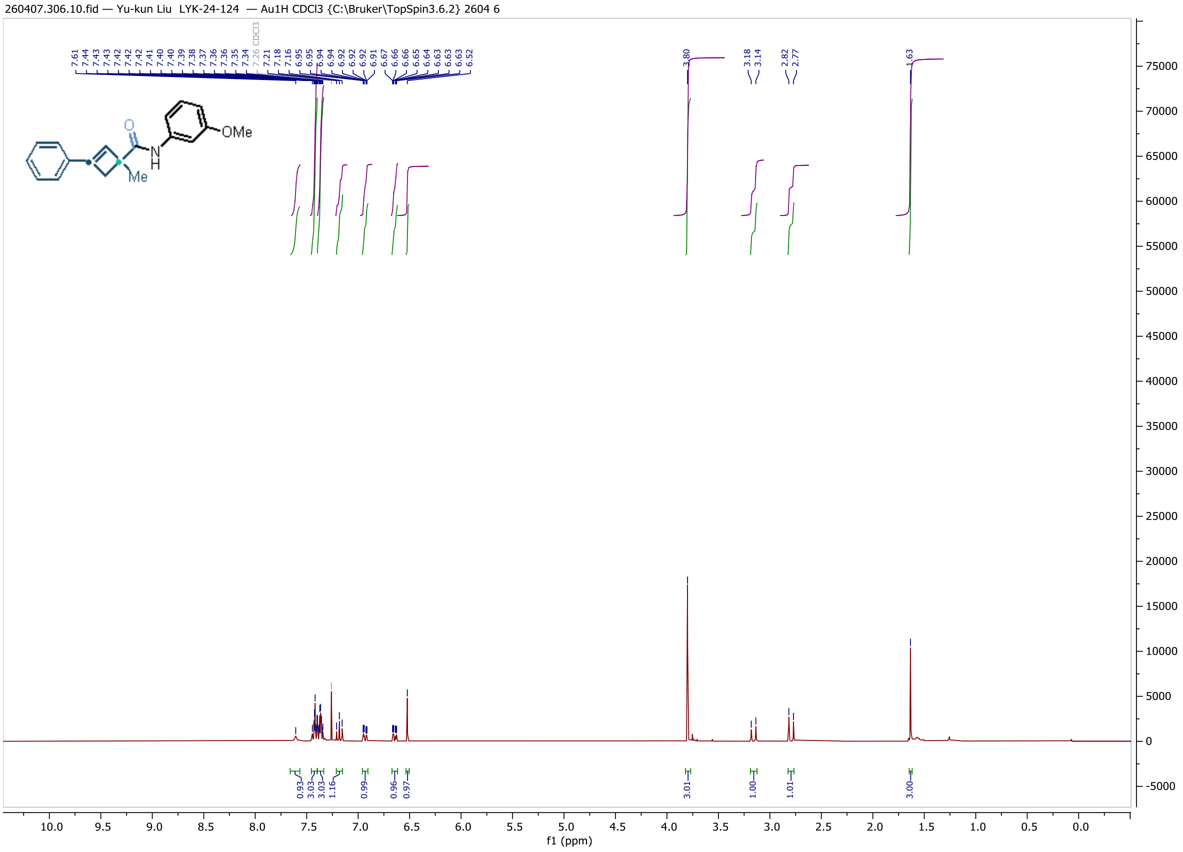

^{13}C NMR spectrum of **4u** (75 MHz, $CDCl_3$)

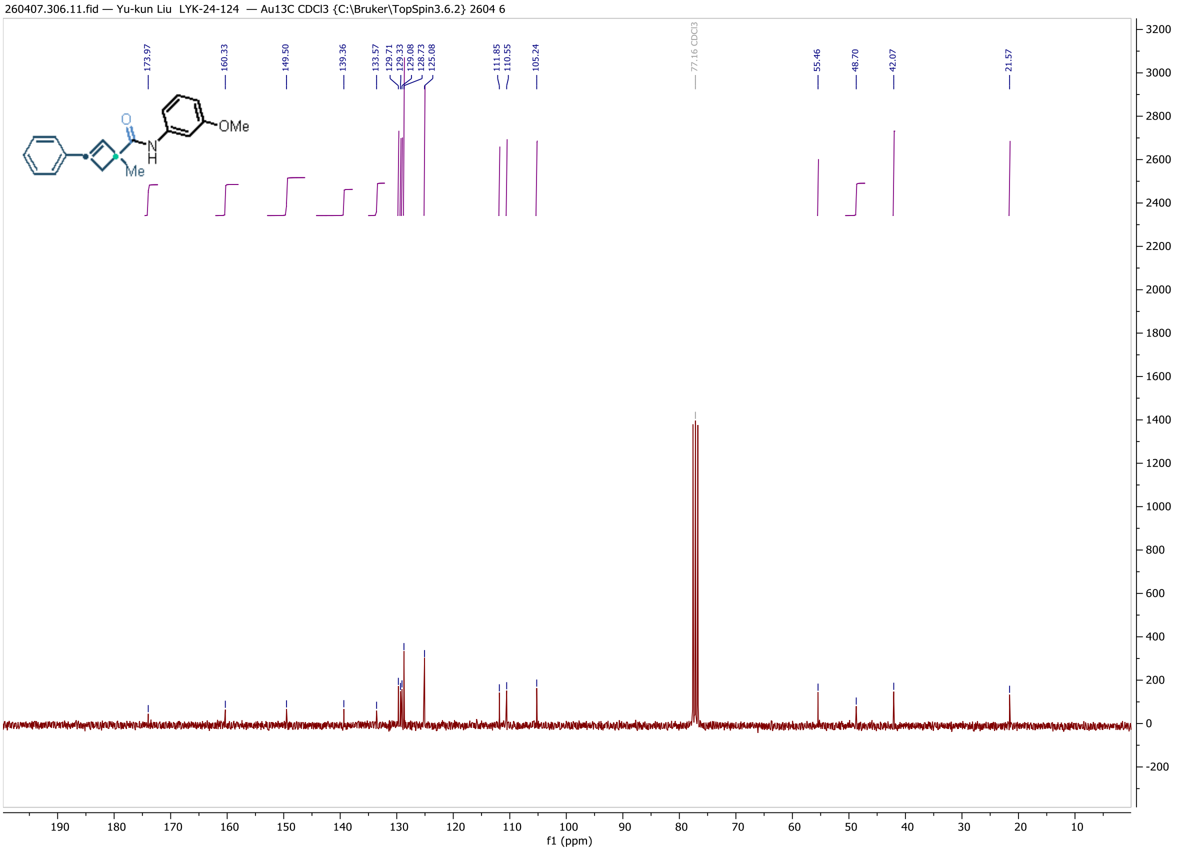

^{1}H NMR spectrum of **4v** (300 MHz, $CDCl_3$)

260223.325.10.fid — Yu-Kun Liu LYK-24-2-81 — Au1H CDCl3 {C:\Bruker\TopSpin3.6.2} 2602 25

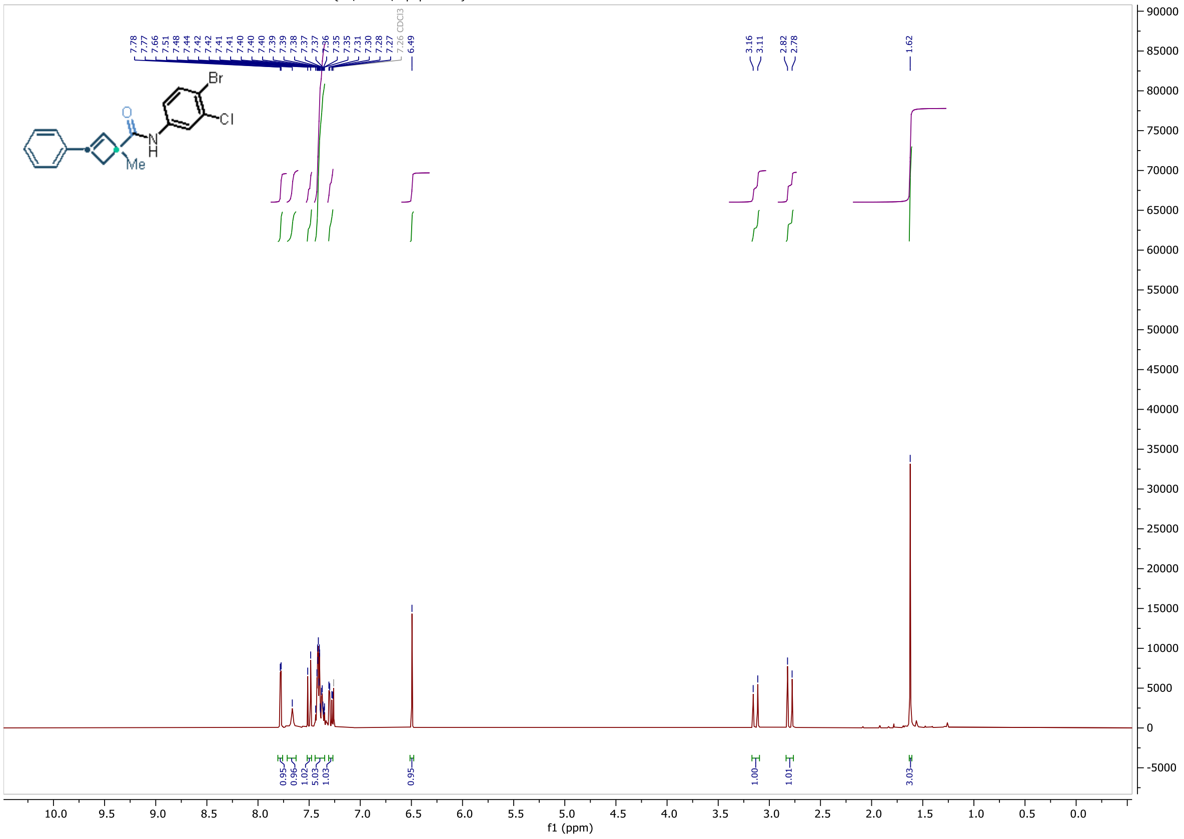

^{13}C NMR spectrum of **4v** (75 MHz, $CDCl_3$)

260223.325.12.fid — Yu-Kun Liu LYK-24-2-81 — Au13C CDCl3 {C:\Bruker\TopSpin3.6.2} 2602 25

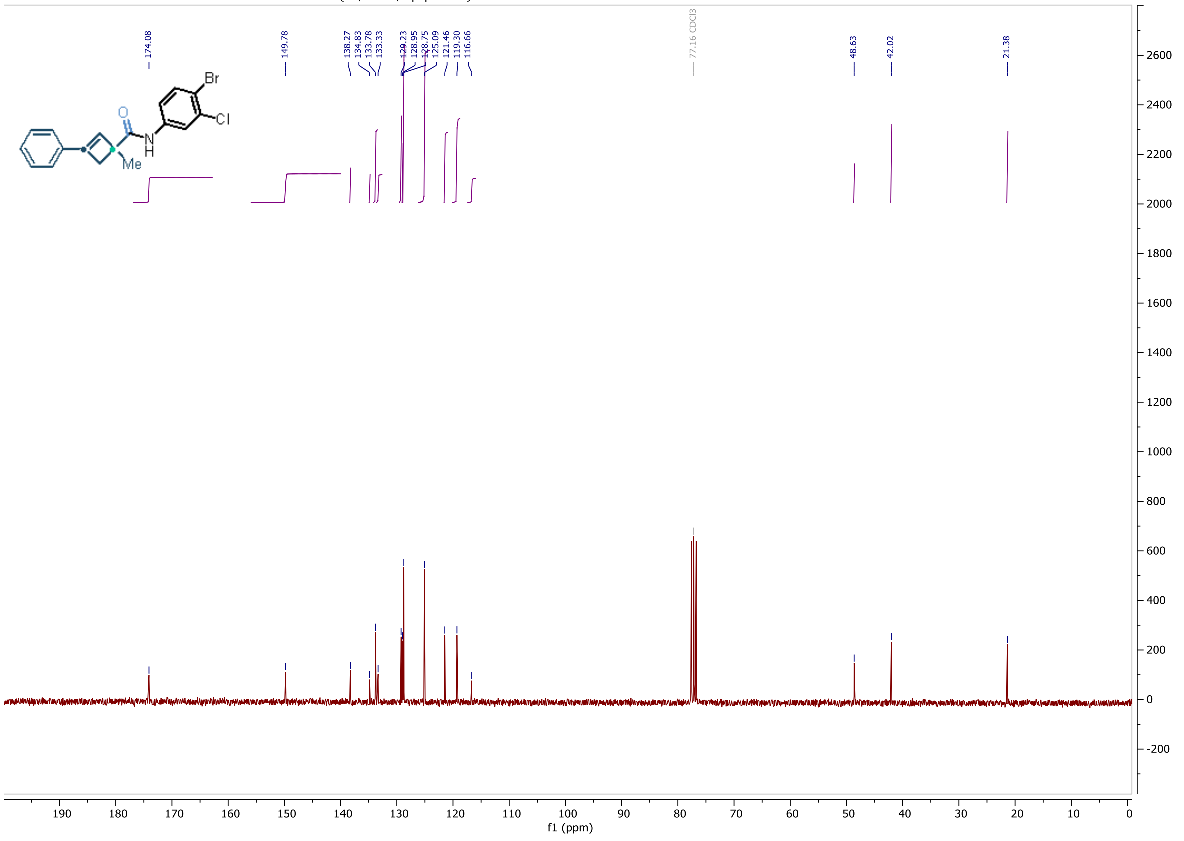

^1H NMR spectrum of **4w** (300 MHz, $CDCl_3$)

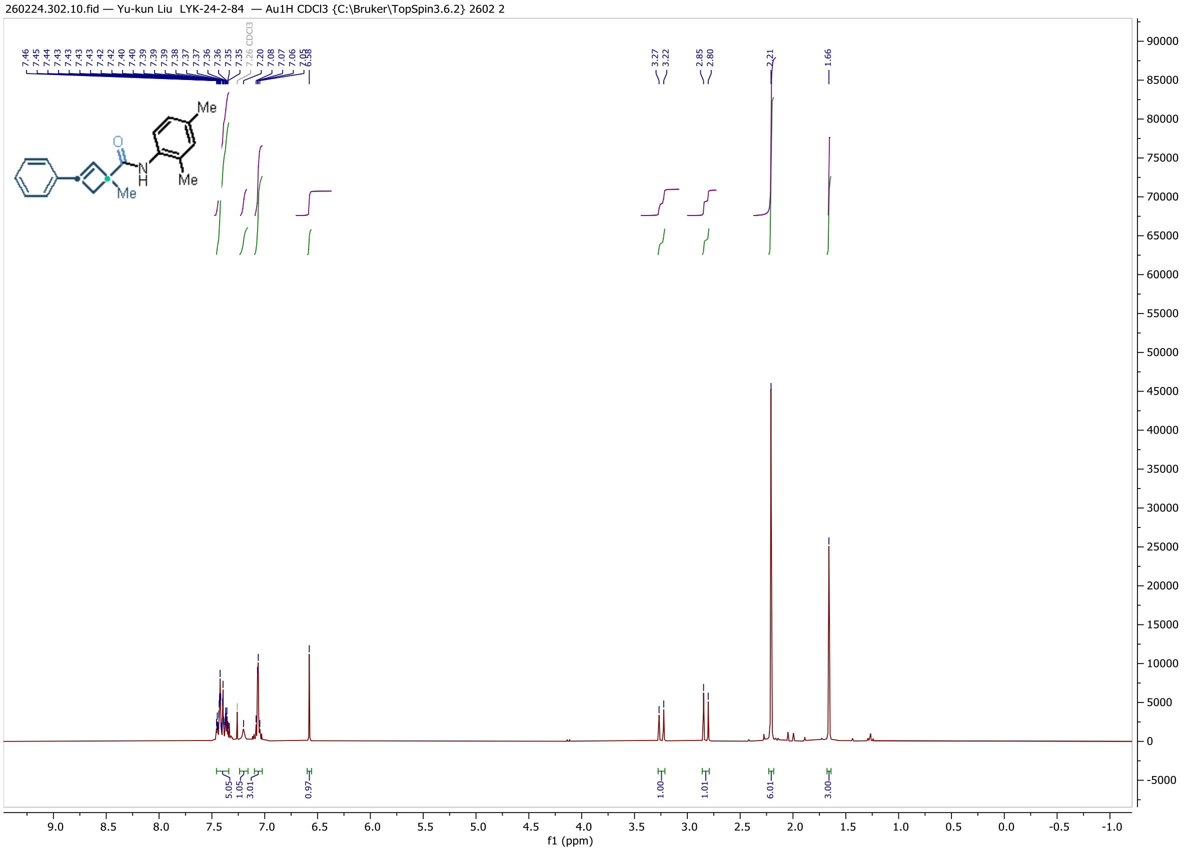

^{13}C NMR spectrum of **4w** (75 MHz, $CDCl_3$)

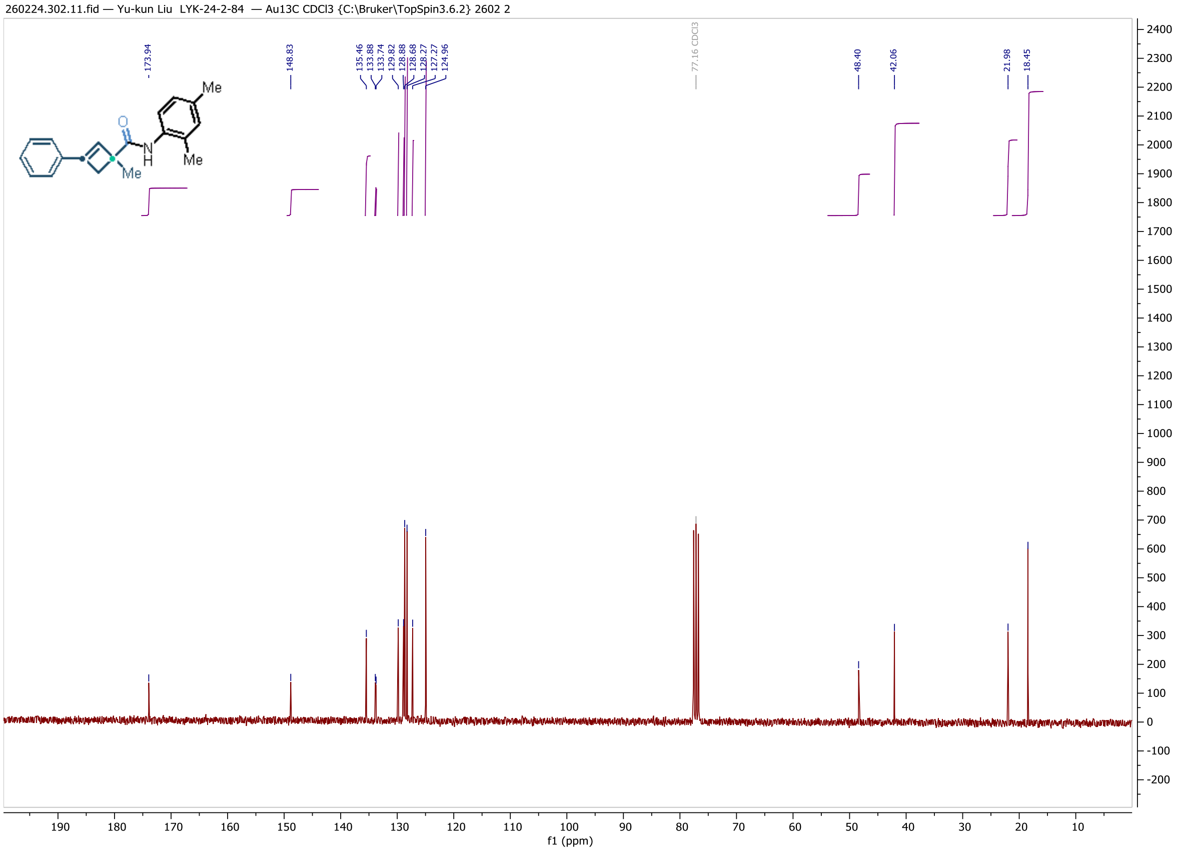

^{1}H NMR spectrum of **4x** (300 MHz, $CDCl_3$)

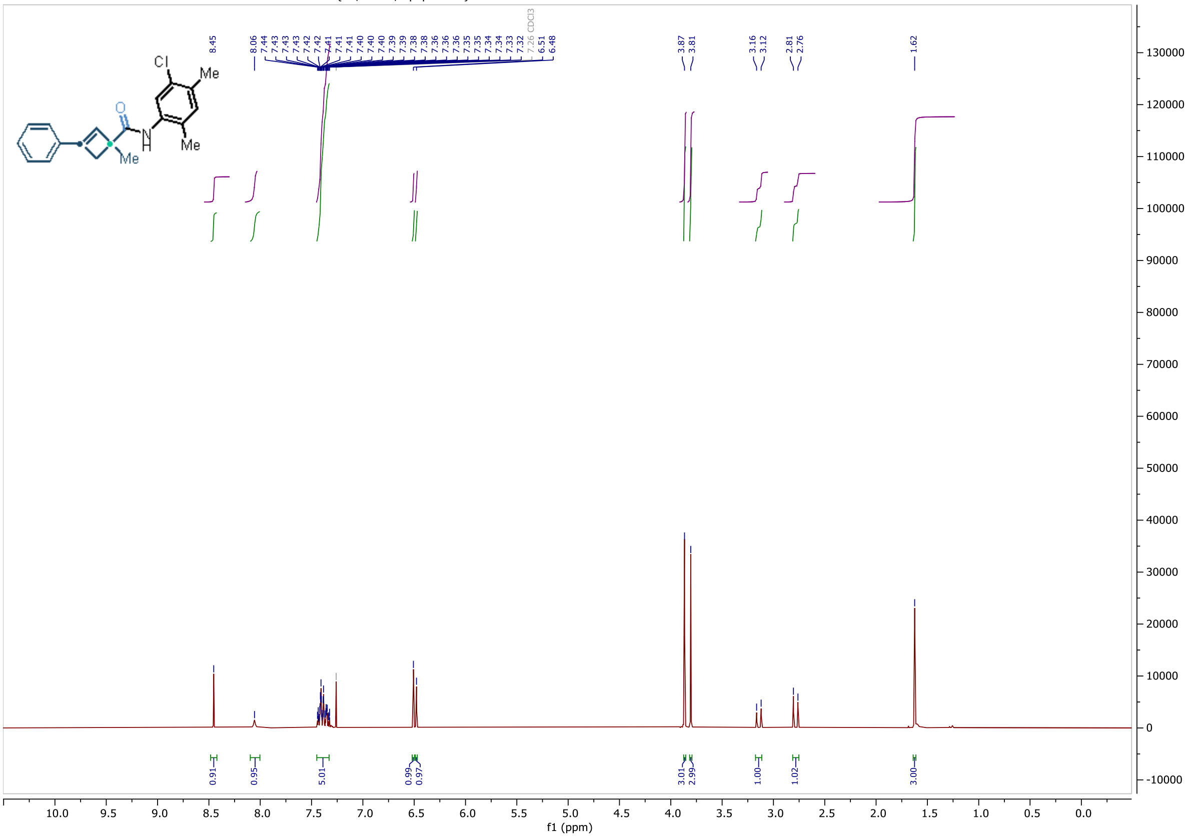

^{13}C NMR spectrum of **4x** (75 MHz, $CDCl_3$)

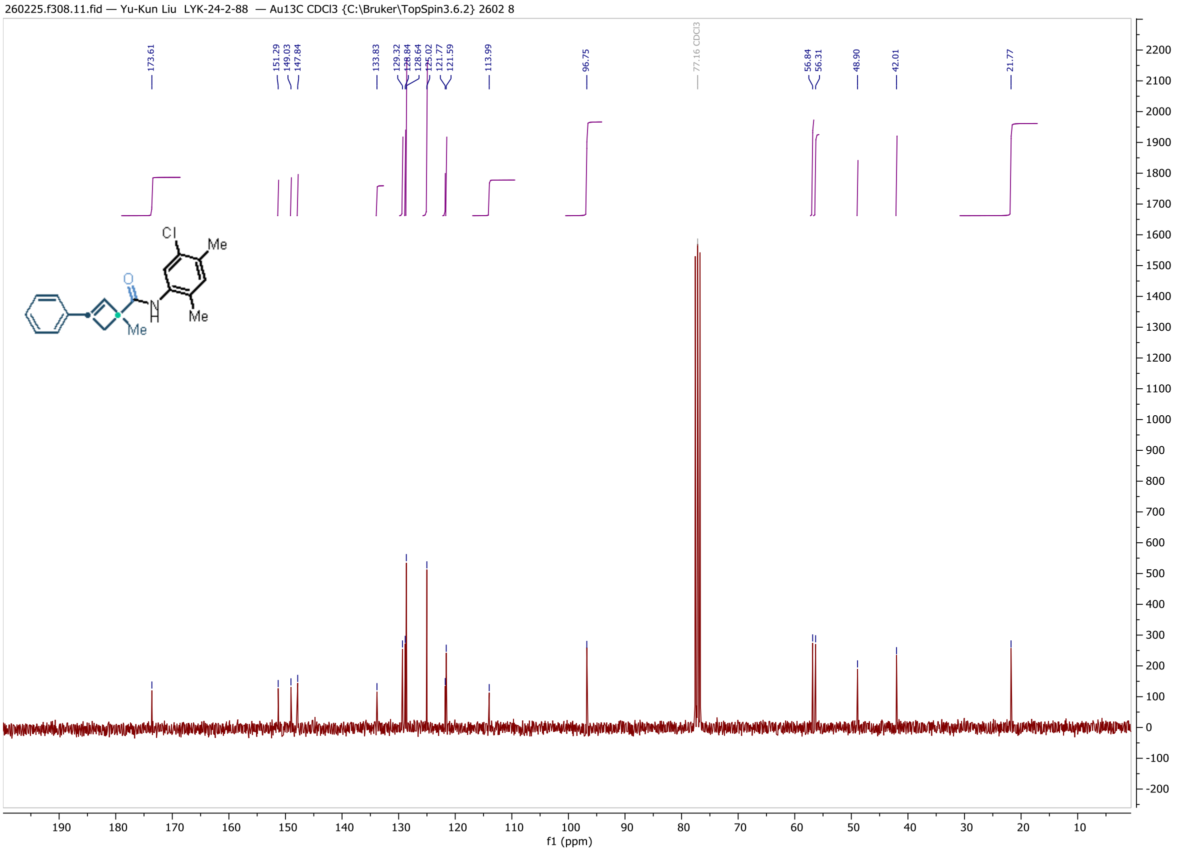

^{1}H NMR spectrum of **4y** (300 MHz, $CDCl_3$)

260224.303.10.fid — Yu-kun Liu LYK-24-2-85 — Au1H CDCl3 {C:\Bruker\TopSpin3.6.2} 2602 3

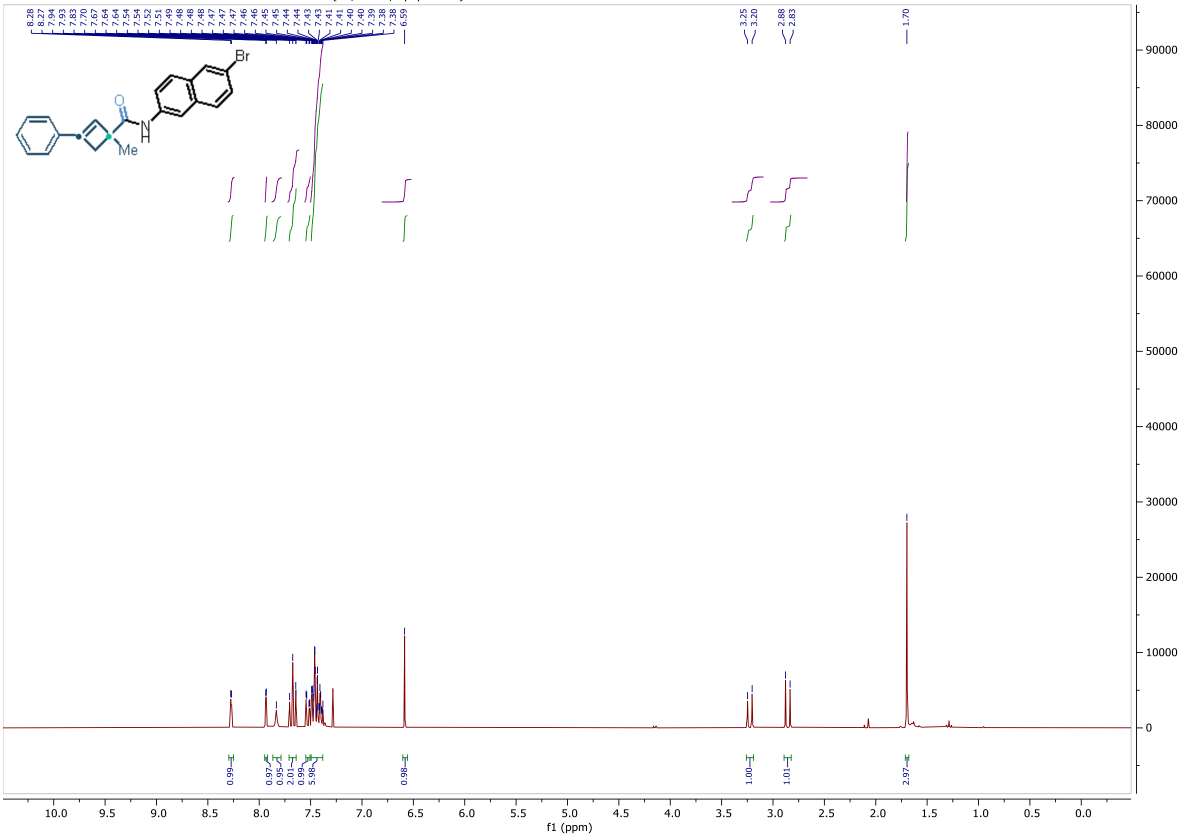

^{13}C NMR spectrum of **4y** (75 MHz, $CDCl_3$)

260224.303.11.fid — Yu-kun Liu LYK-24-2-85 — Au13C CDCl3 {C:\Bruker\TopSpin3.6.2} 2602 3

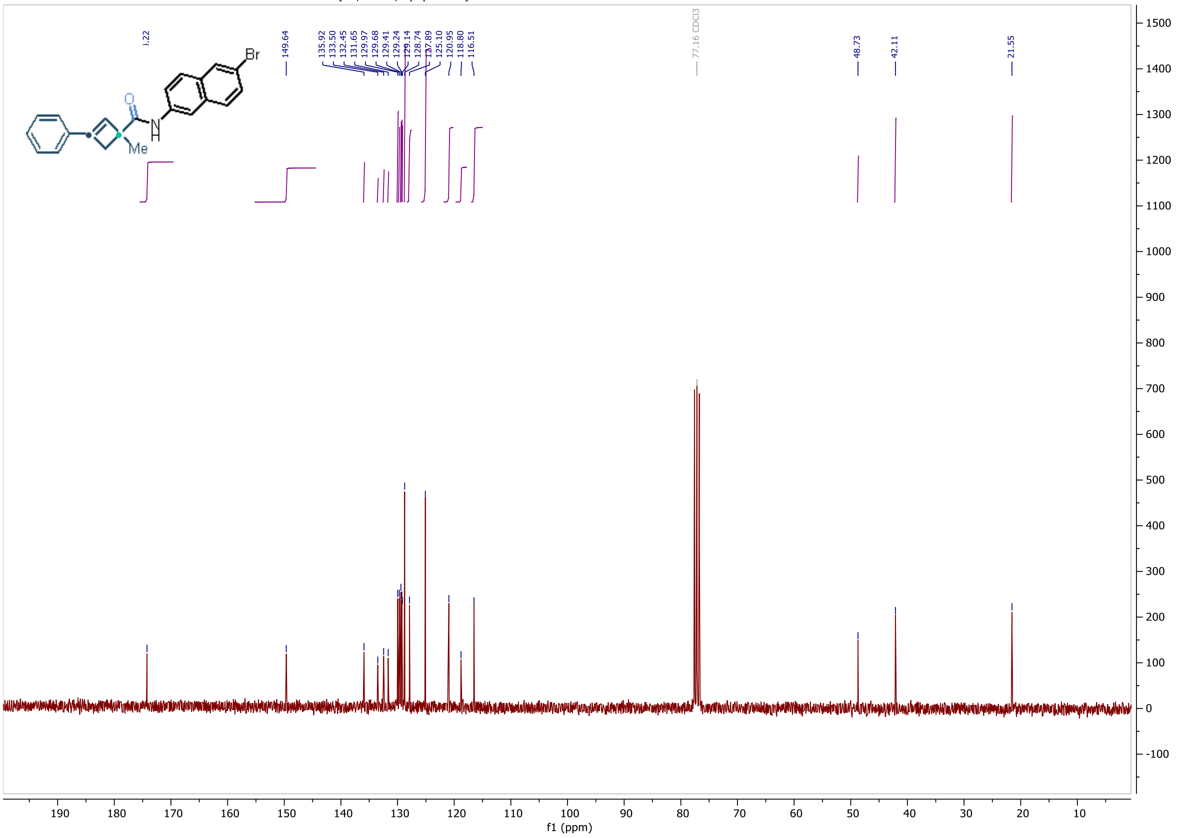

^{1}H NMR spectrum of **4z** (300 MHz, $CDCl_3$)

260220.311.10.fid — Yu-kun Liu LYK-24-2-78 — Au1H CDCl3 {C:\Bruker\TopSpin3.6.2} 2602 11

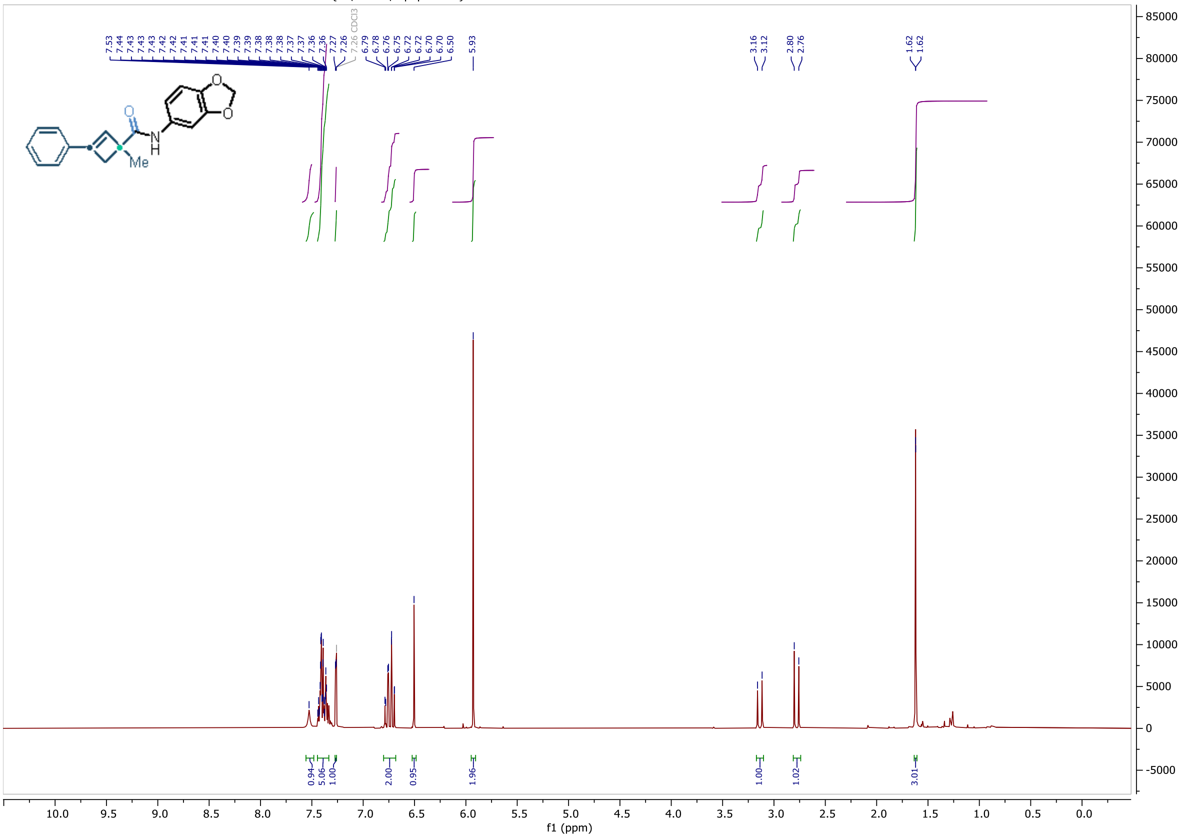

^{13}C NMR spectrum of **4z** (75 MHz, $CDCl_3$)

260220.311.11.fid — Yu-kun Liu LYK-24-2-78 — Au13C CDCl3 {C:\Bruker\TopSpin3.6.2} 2602 11

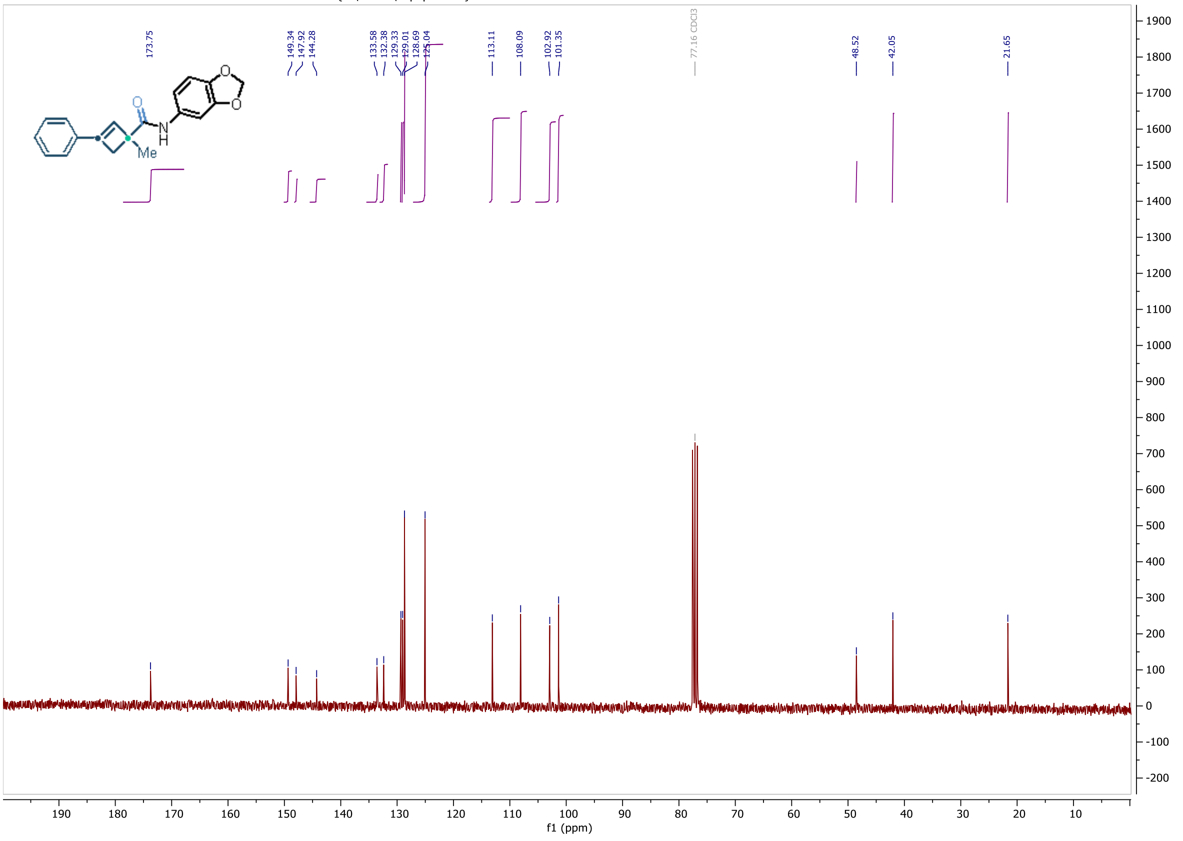

^{1}H NMR spectrum of **4ab** (300 MHz, $CDCl_3$)

260217.407.10.fid — Yu-kun Liu LYK-24-2-73 — Au1H CDCl3 {C:\Bruker\TopSpin3.6.2} 2602 7

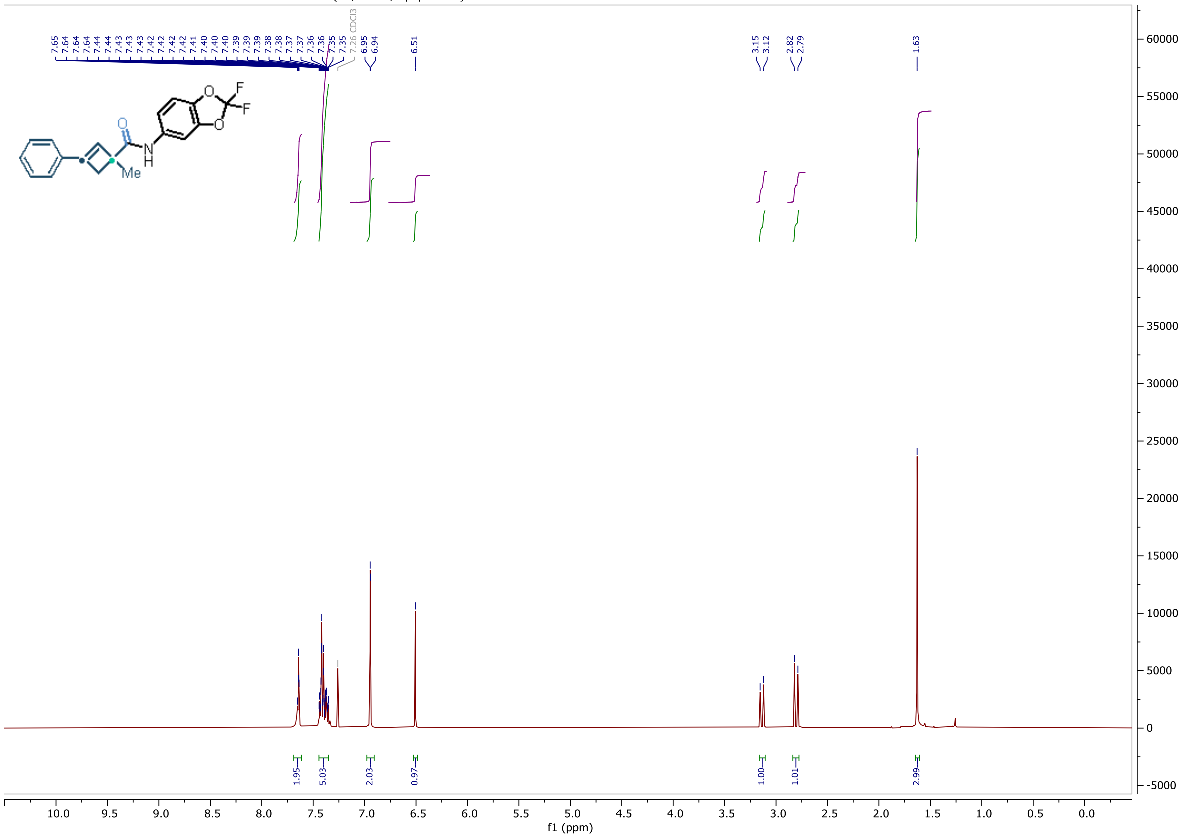

^{13}C NMR spectrum of **4ab** (75 MHz, $CDCl_3$)

260217.407.11.fid — Yu-kun Liu LYK-24-2-73 — Au13C CDCl3 {C:\Bruker\TopSpin3.6.2} 2602 7

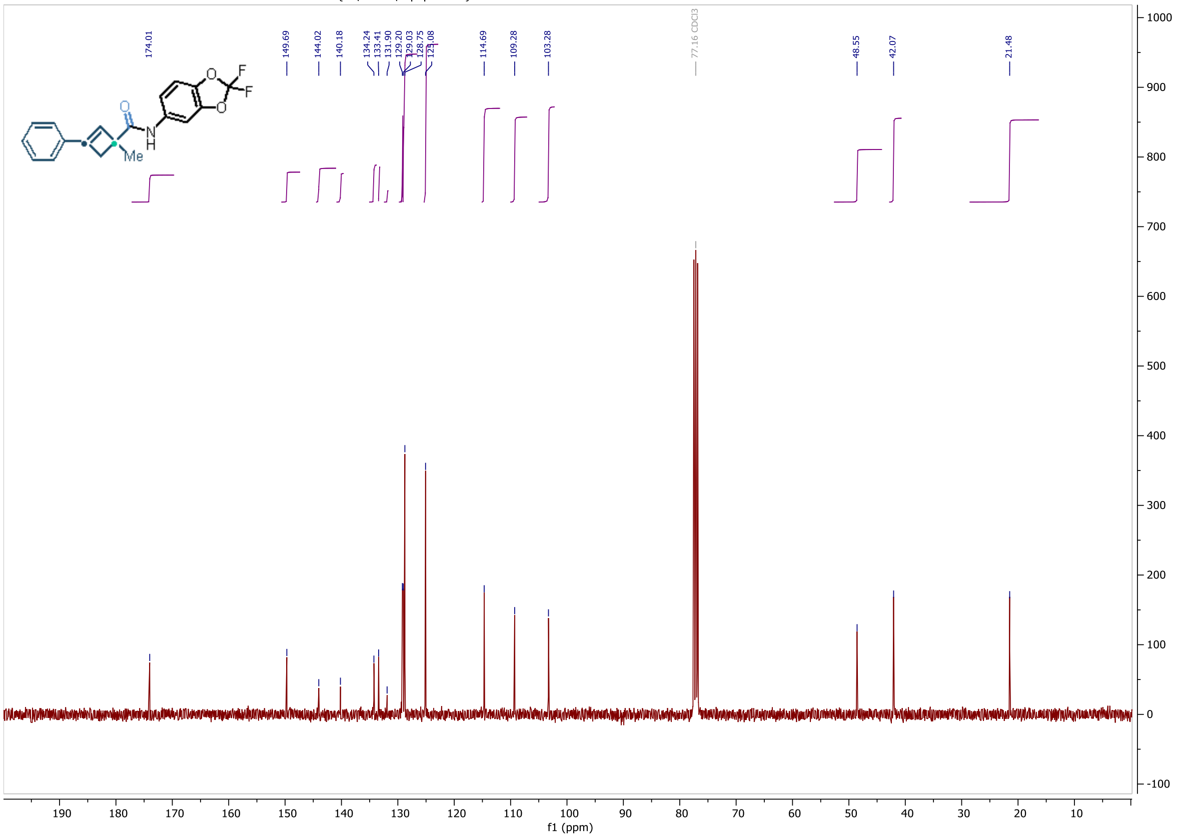

^{19}F NMR spectrum of **4ab** (282 MHz, $CDCl_3$)

260217.407.12.fid — Yu-kun Liu LYK-24-2-73 — Au19F CDCl3 {C:\Bruker\TopSpin3.6.2} 2602 7

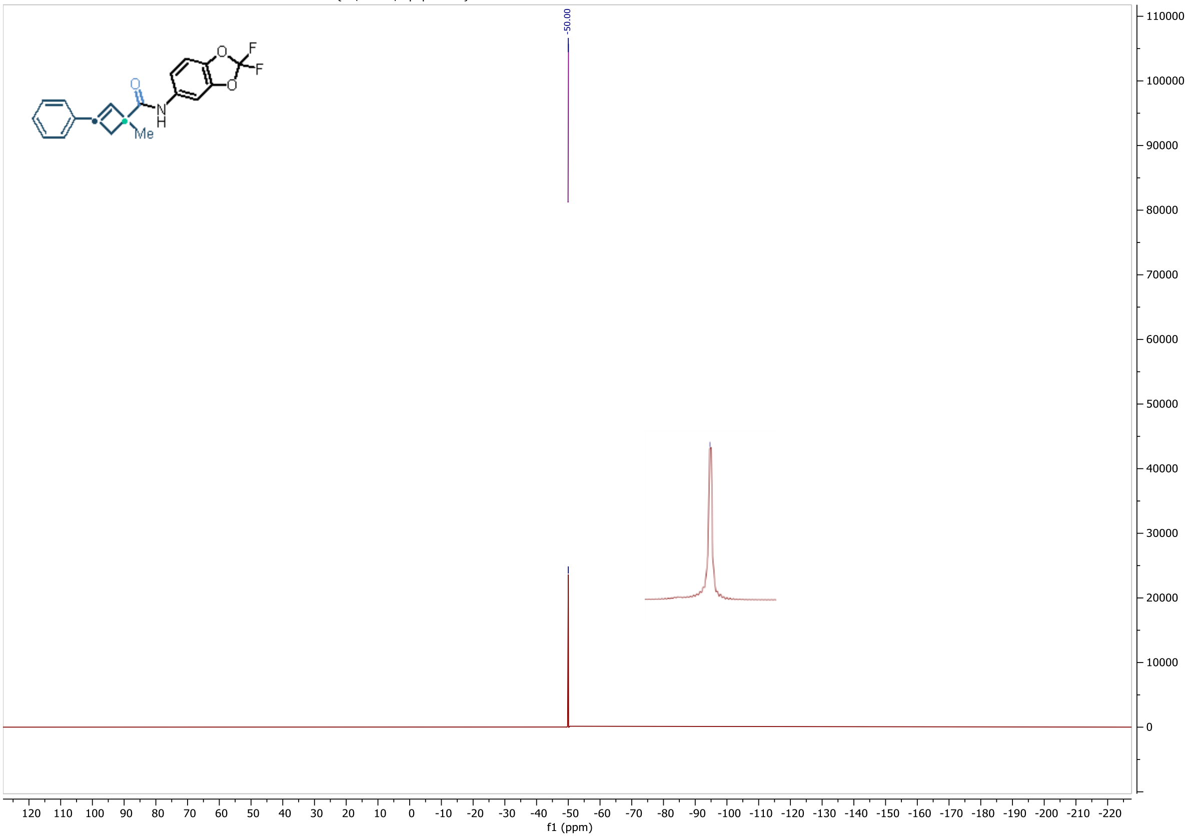

^{1}H NMR spectrum of **4ac** (400 MHz, $CDCl_3$)

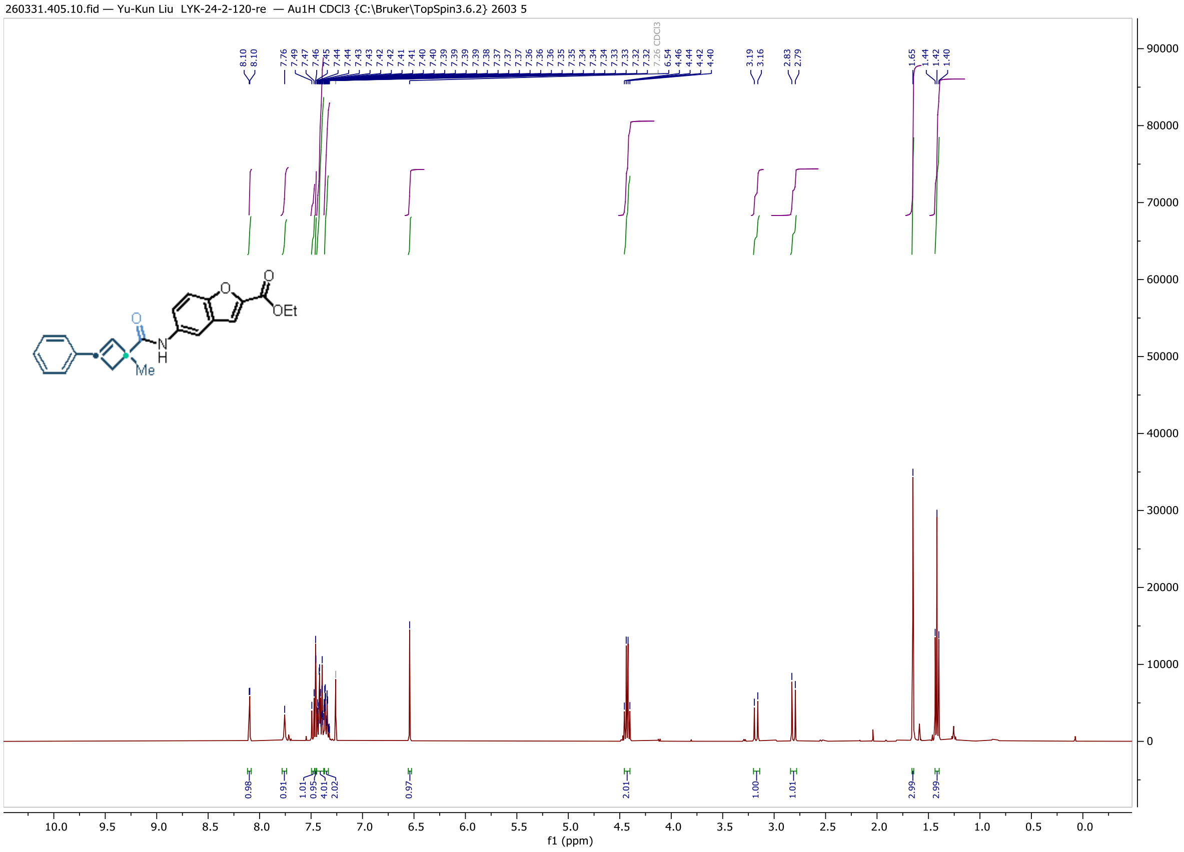

^{13}C NMR spectrum of **4ac** (101 MHz, $CDCl_3$)

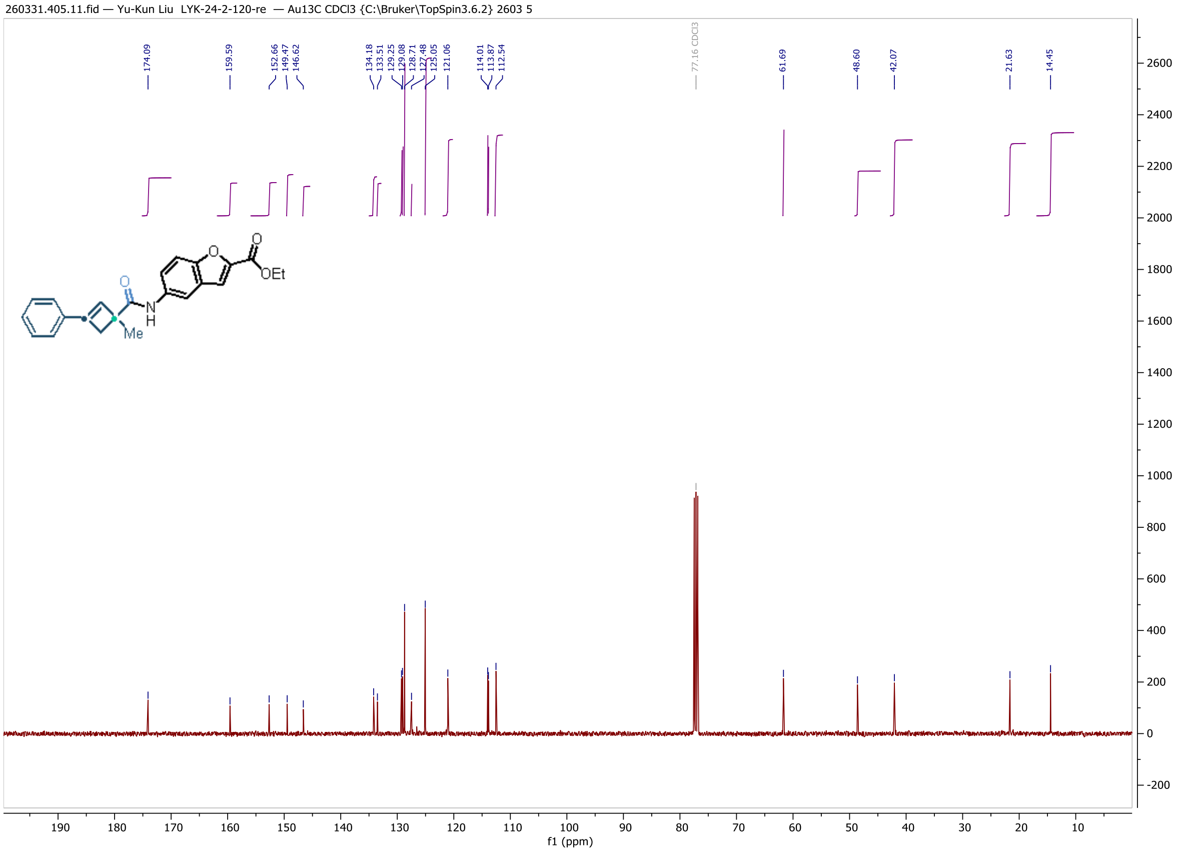

^{1}H NMR spectrum of **4ad** (300 MHz, $CDCl_3$)

260303.f306.10.fid — Yu-kun Liu LYK-24-2-99 — Au1H CDCl3 {C:\Bruker\TopSpin3.6.2} 2603 6

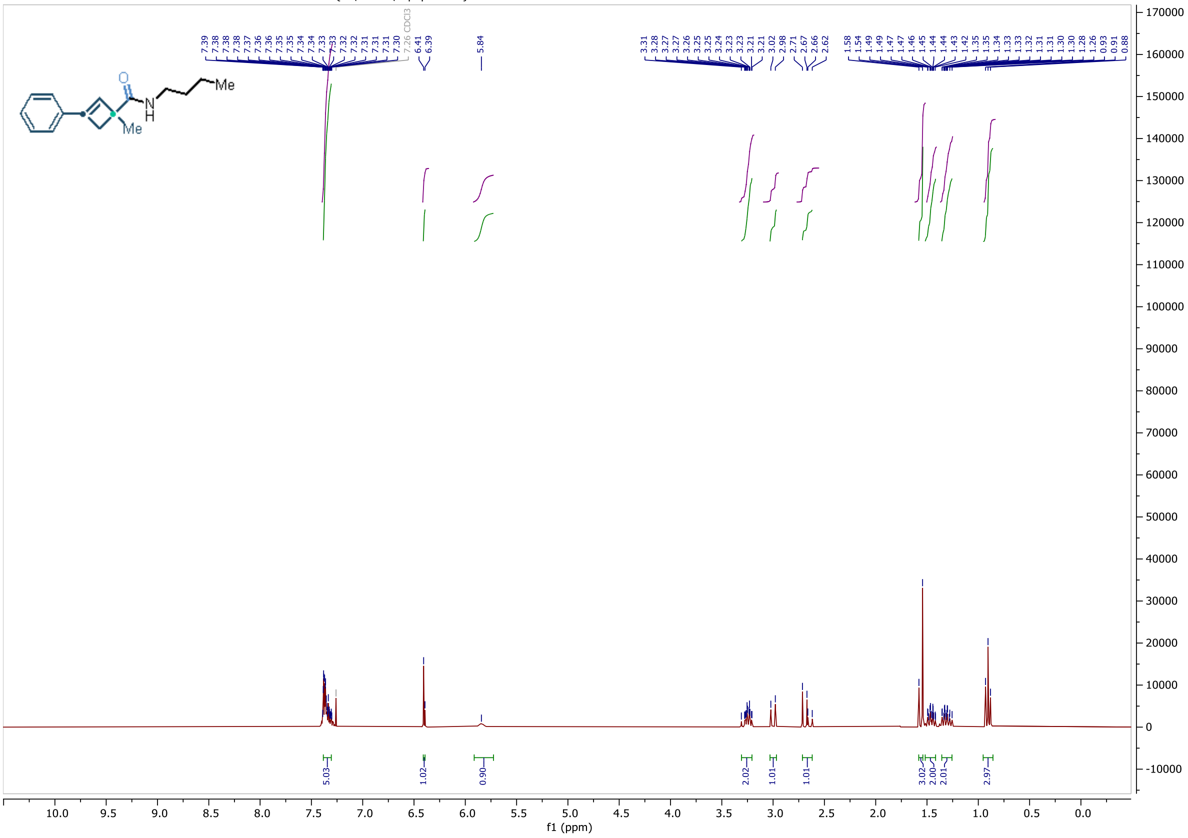

^{13}C NMR spectrum of **4ad** (75 MHz, $CDCl_3$)

260303.f306.11.fid — Yu-kun Liu LYK-24-2-99 — Au13C CDCl3 {C:\Bruker\TopSpin3.6.2} 2603 6

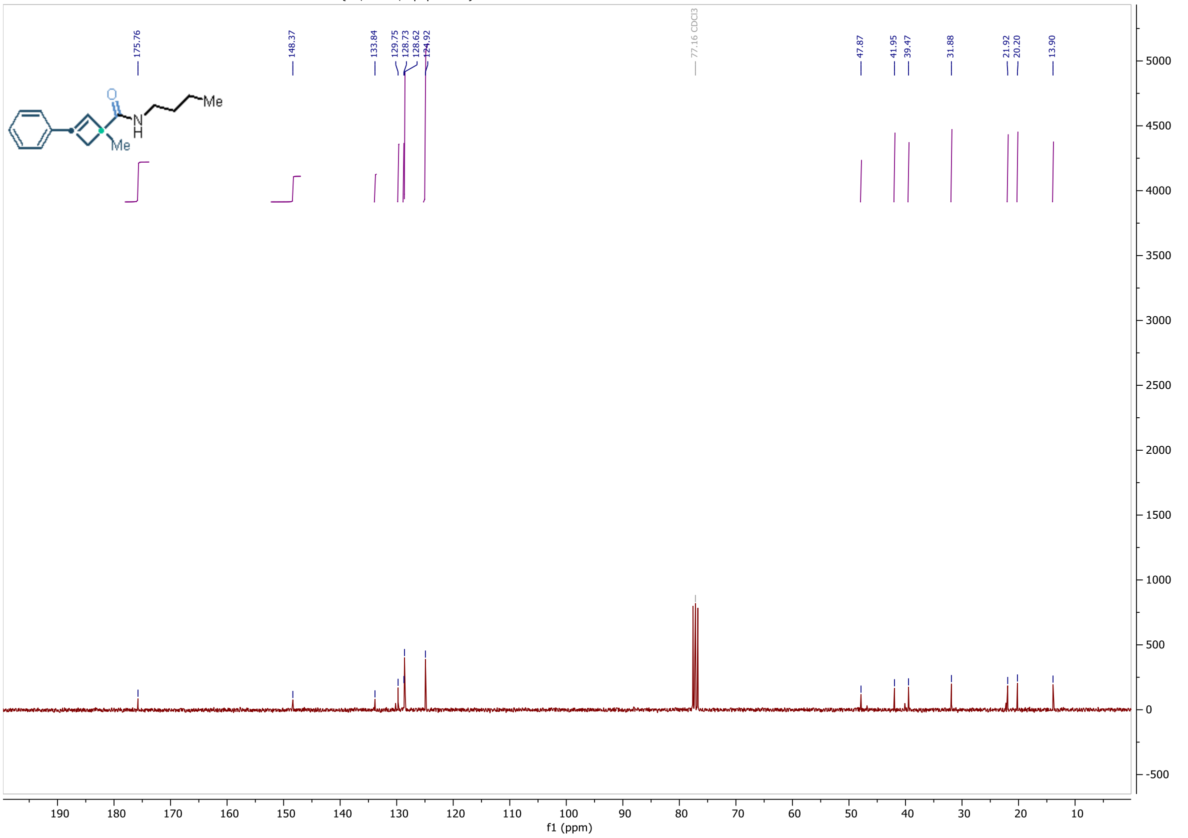

^{1}H NMR spectrum of **4ae** (300 MHz, $CDCl_3$)

260225.f309.10.fid — Yu-Kun Liu LYK-24-2-89 — Au1H CDCl3 {C:\Bruker\TopSpin3.6.2} 2602 9

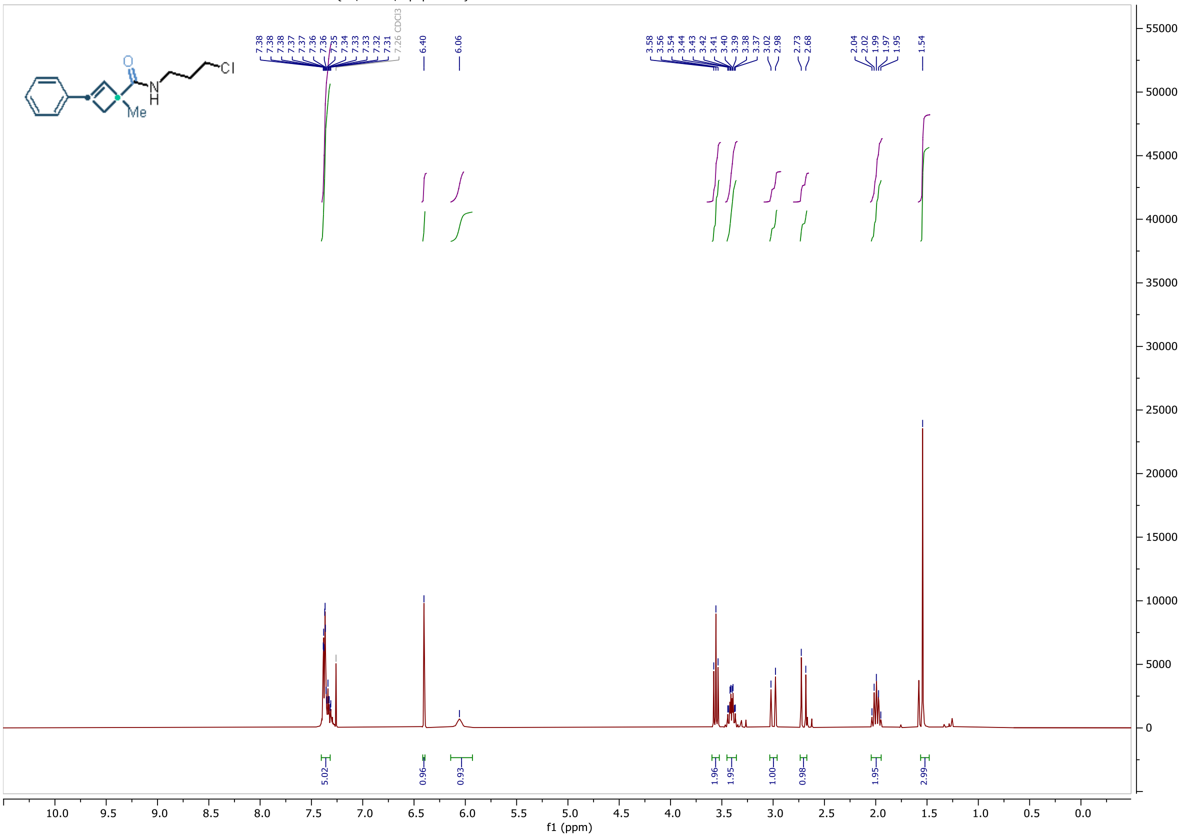

^{13}C NMR spectrum of **4ae** (75 MHz, $CDCl_3$)

260225.f309.11.fid — Yu-Kun Liu LYK-24-2-89 — Au13C CDCl3 {C:\Bruker\TopSpin3.6.2} 2602 9

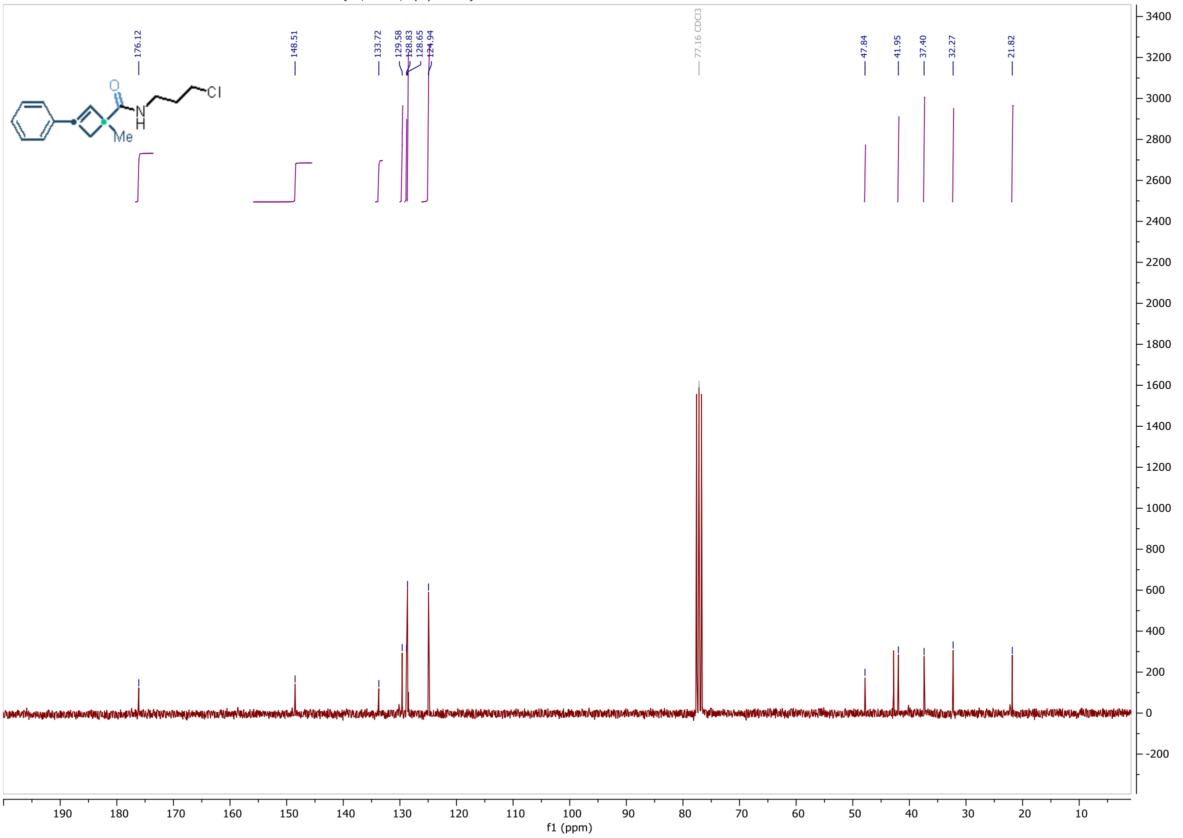

^{1}H NMR spectrum of **4af** (300 MHz, $CDCl_3$)

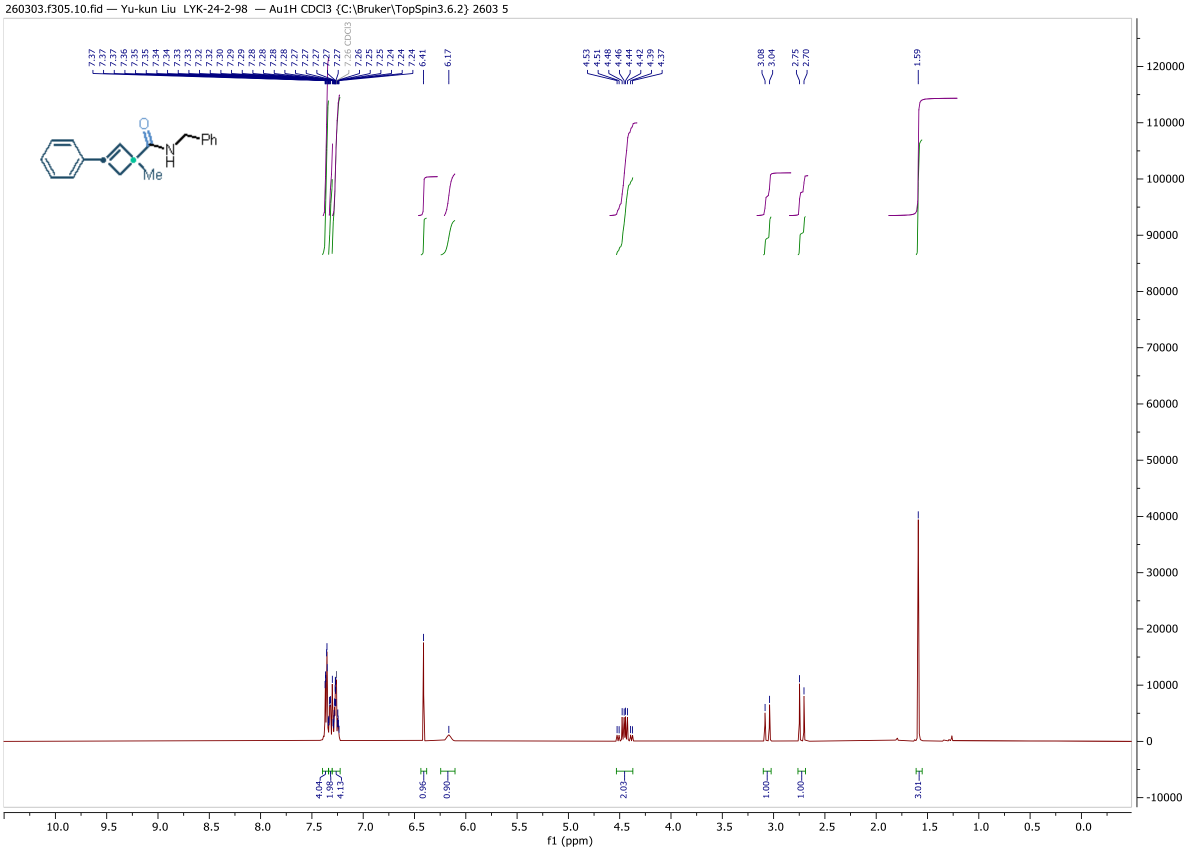

^{13}C NMR spectrum of **4af** (75 MHz, $CDCl_3$)

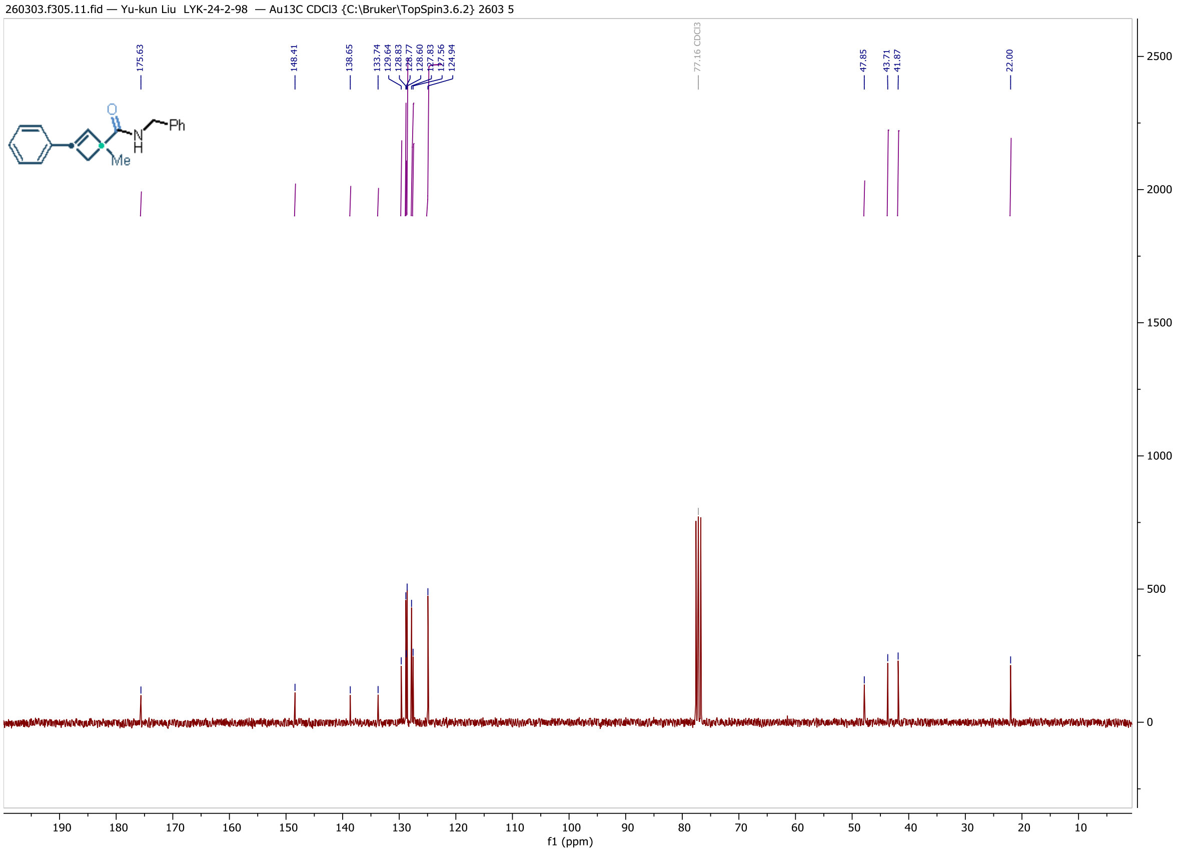

^{1}H NMR spectrum of **4ag** (300 MHz, $CDCl_3$)

260304.309.10.fid — Yu-kun Liu LYK-24-2-106 — Au1H CDCl3 {C:\Bruker\TopSpin3.6.2} 2603 9

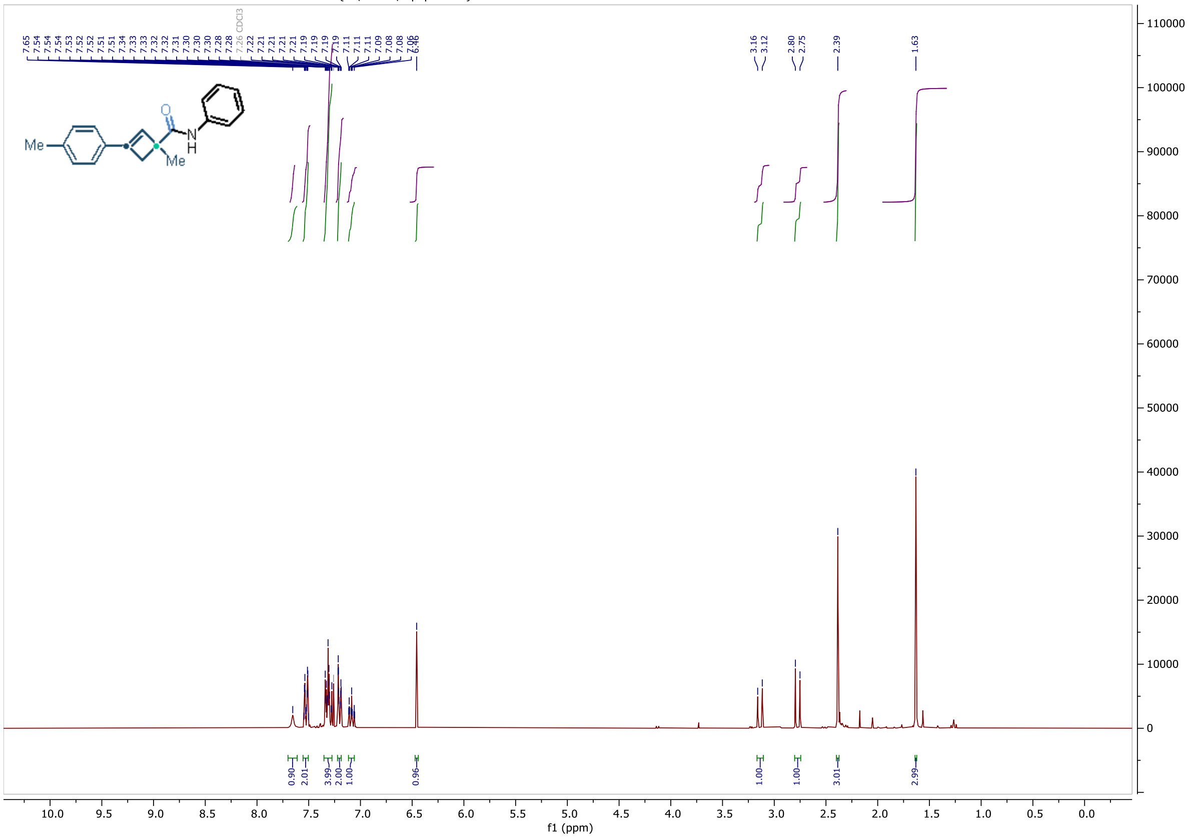

^{13}C NMR spectrum of **4ag** (75 MHz, $CDCl_3$)

260304.309.11.fid — Yu-kun Liu LYK-24-2-106 — Au13C CDCl3 {C:\Bruker\TopSpin3.6.2} 2603 9

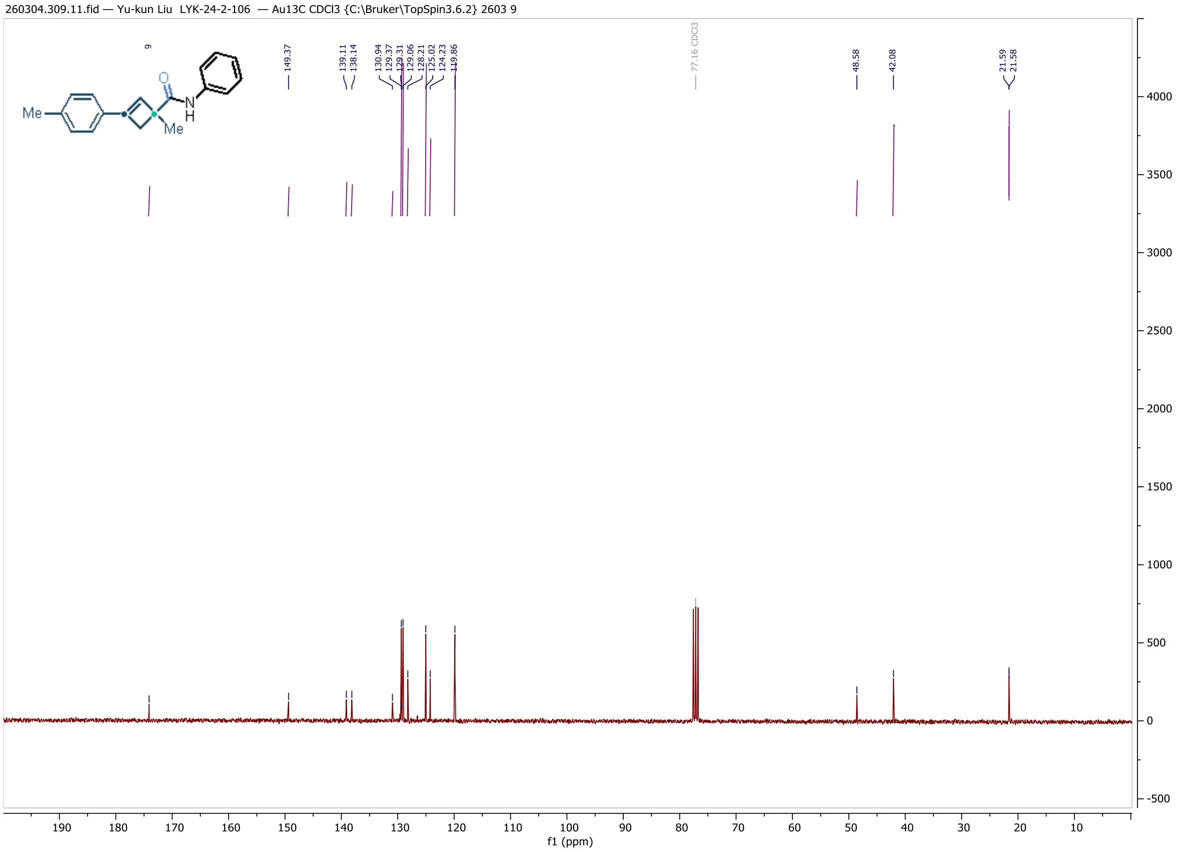

^{1}H NMR spectrum of **4ah** (300 MHz, $CDCl_3$)

260304.312.10.fid — Yu-kun Liu LYK-24-2-109 — Au1H CDCl3 {C:\Bruker\TopSpin3.6.2} 2603 12

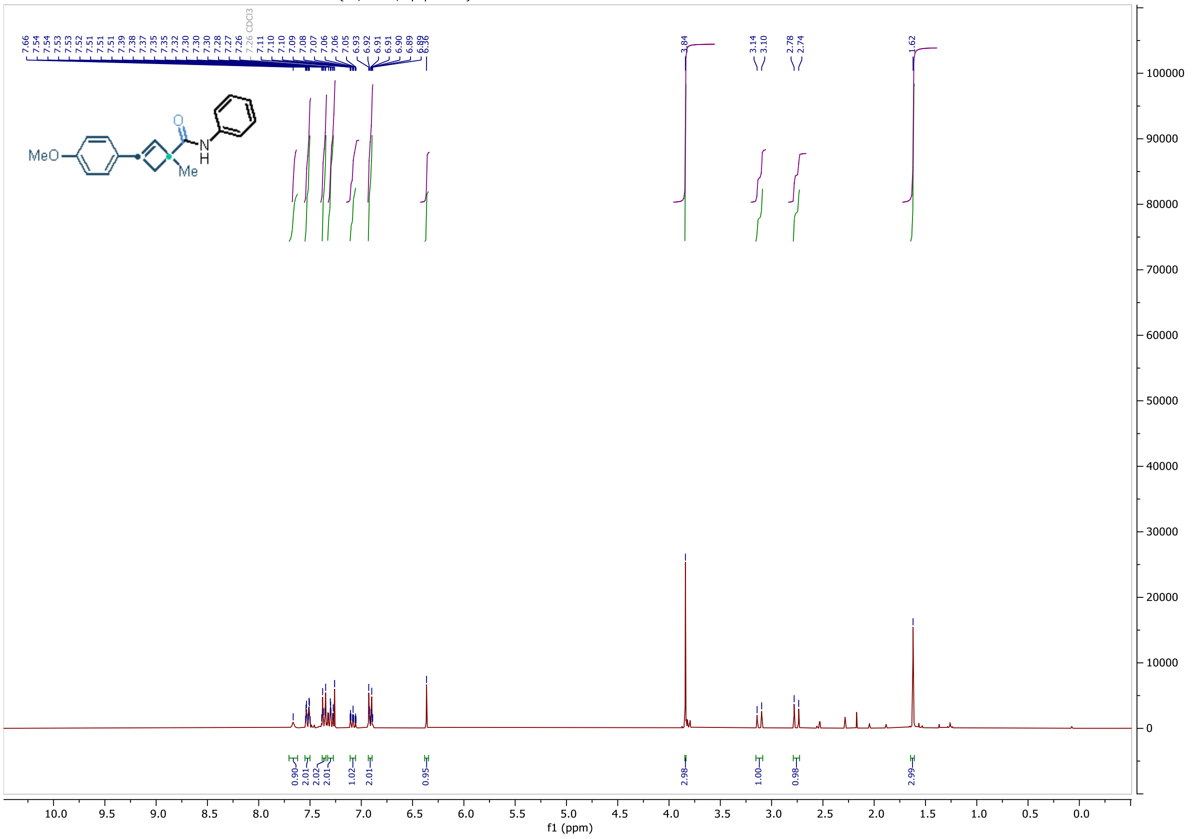

^{13}C NMR spectrum of **4ah** (75 MHz, $CDCl_3$)

260304.312.11.fid — Yu-kun Liu LYK-24-2-109 — Au13C CDCl3 {C:\Bruker\TopSpin3.6.2} 2603 12

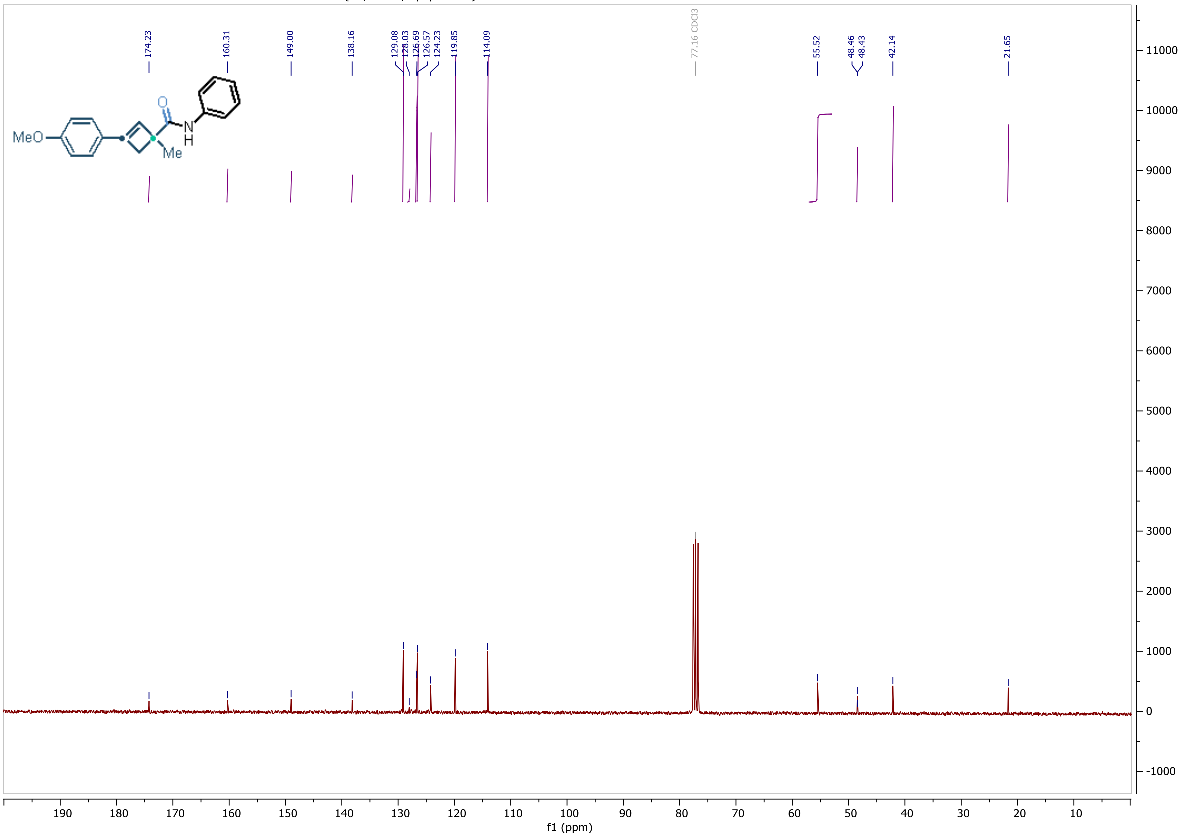

^{1}H NMR spectrum of **4ai** (300 MHz, $CDCl_3$)

260407.308.10.fid — Yu-kun Liu LYK-24-126 — Au1H CDCl3 {C:\Bruker\TopSpin3.6.2} 2604 8

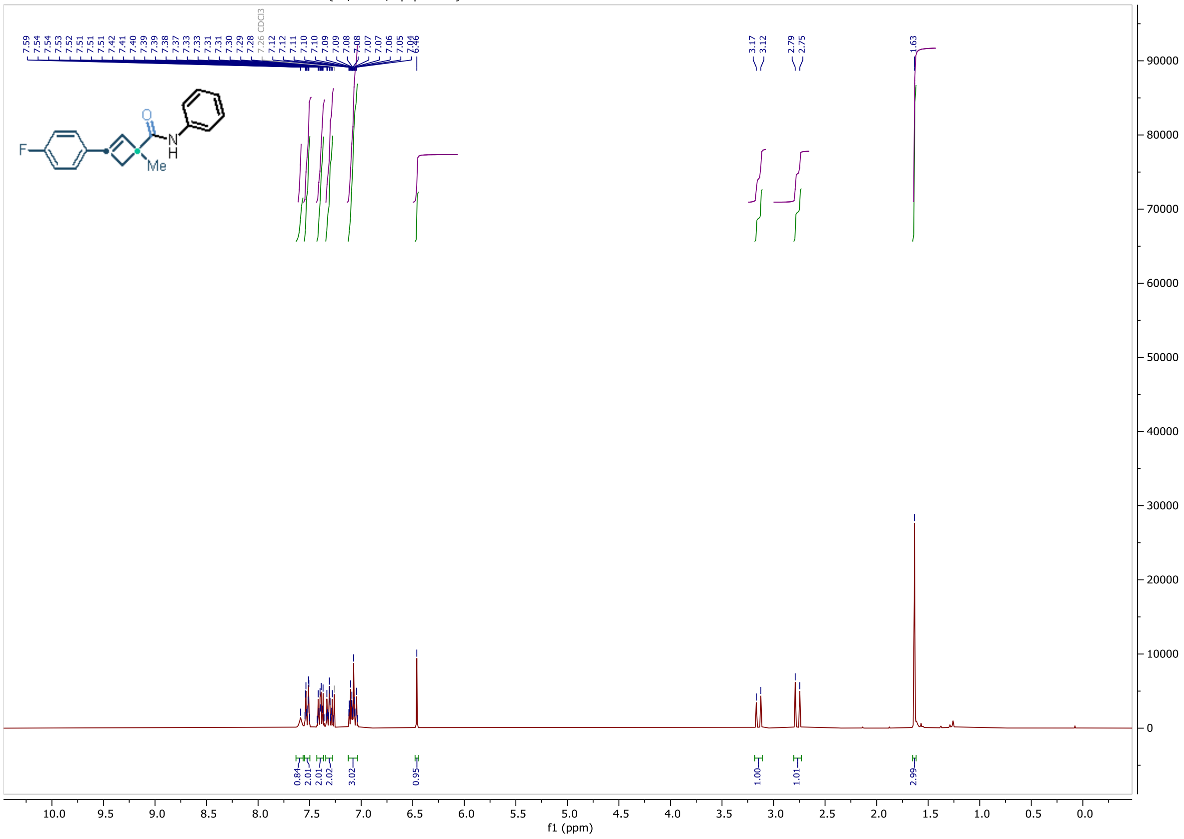

^{13}C NMR spectrum of **4ai** (75 MHz, $CDCl_3$)

260407.308.11.fid — Yu-kun Liu LYK-24-126 — Au13C CDCl3 {C:\Bruker\TopSpin3.6.2} 2604 8

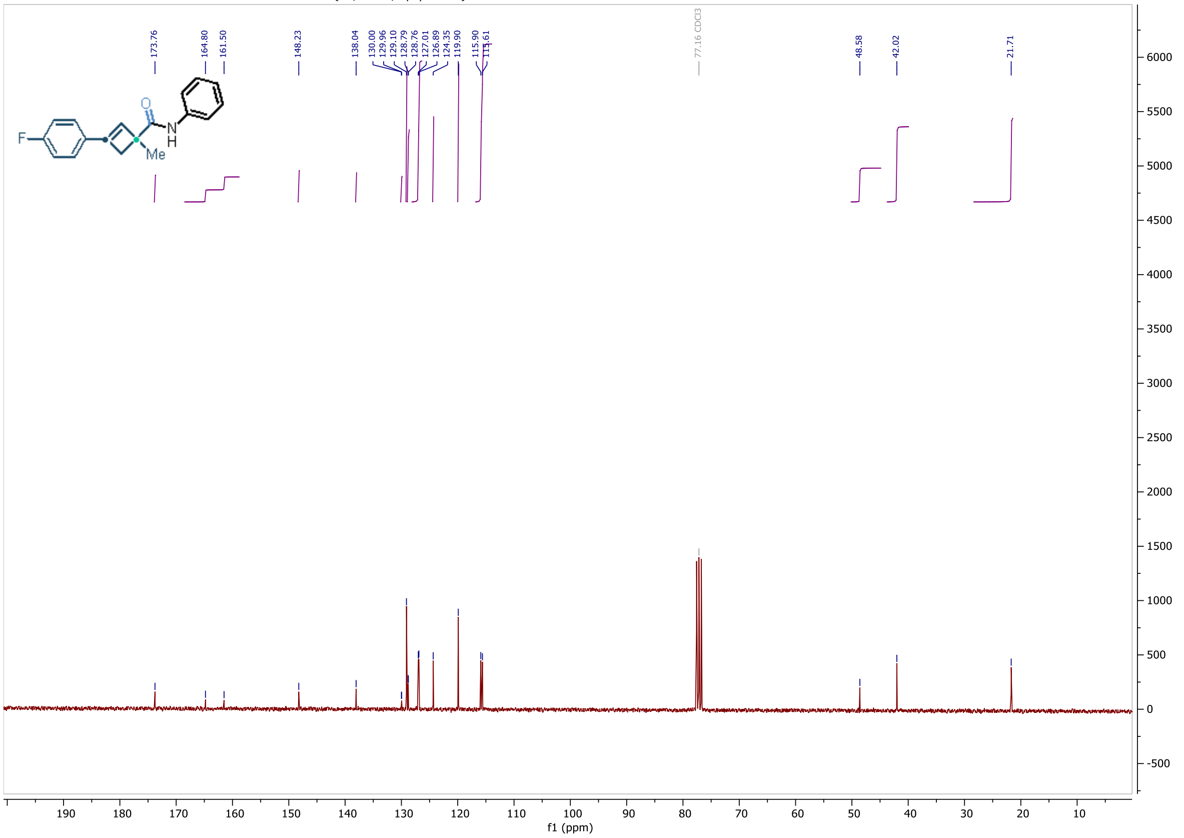

^{19}F NMR spectrum of **4ai** (282 MHz, $CDCl_3$)

260407.308.12.fid — Yu-kun Liu LYK-24-126 — Au19F CDCl3 {C:\Bruker\TopSpin3.6.2} 2604 8

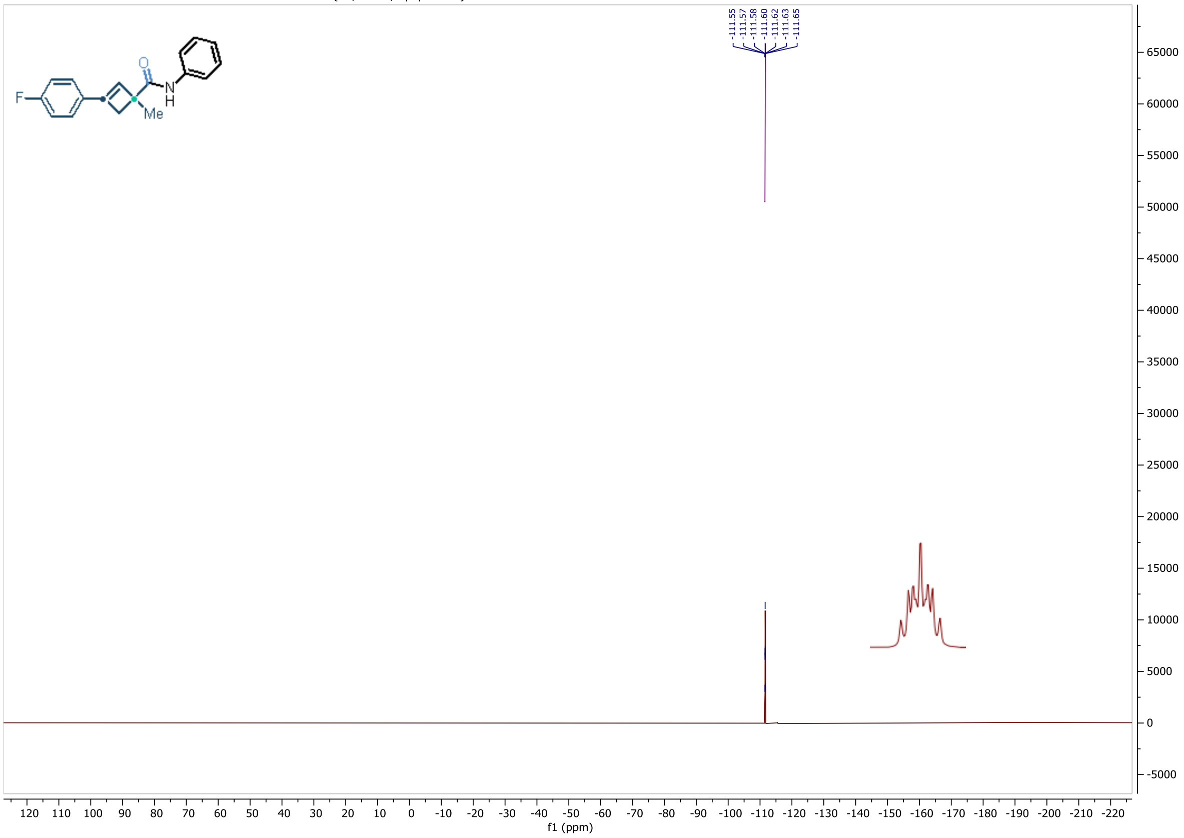

^{1}H NMR spectrum of **4aj** (300 MHz, $CDCl_3$)

260304.310.10.fid — Yu-kun Liu LYK-24-2-107 — Au1H CDCl3 {C:\Bruker\TopSpin3.6.2} 2603 10

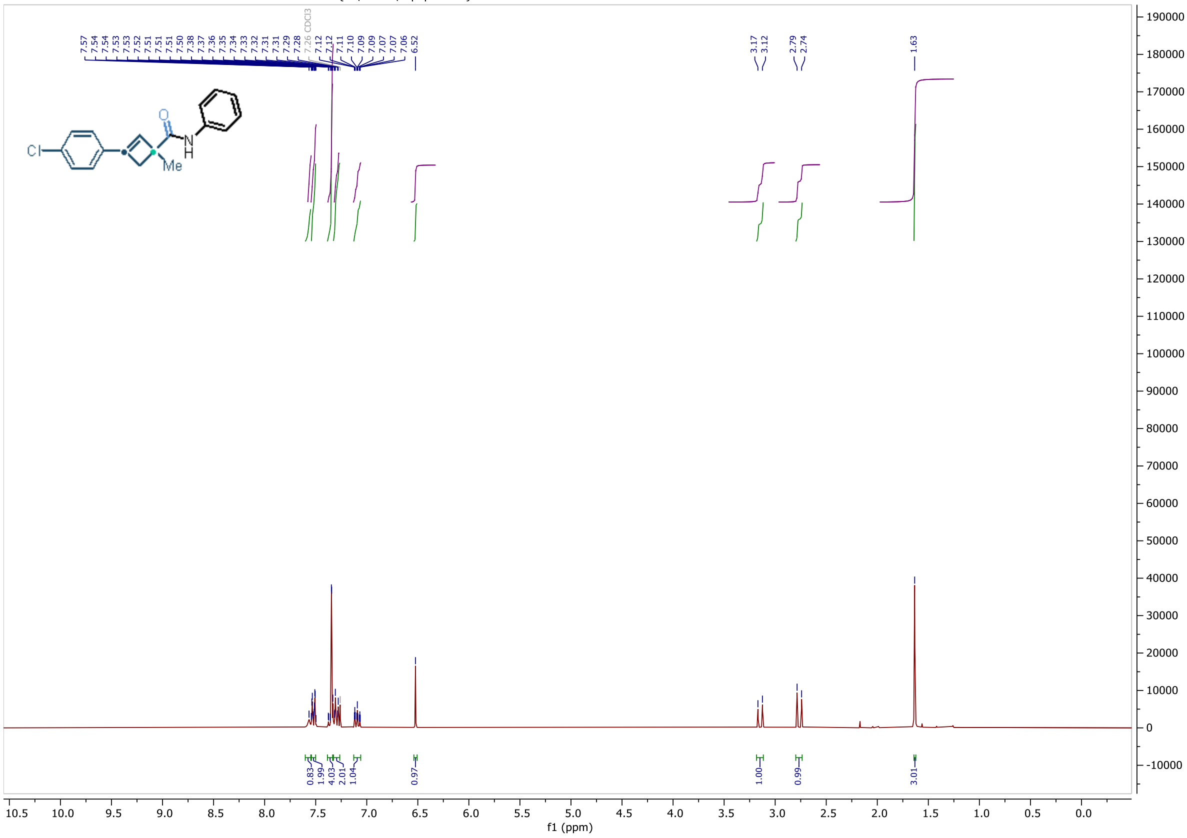

^{13}C NMR spectrum of **4aj** (75 MHz, $CDCl_3$)

260304.310.11.fid — Yu-kun Liu LYK-24-2-107 — Au13C CDCl3 {C:\Bruker\TopSpin3.6.2} 2603 10

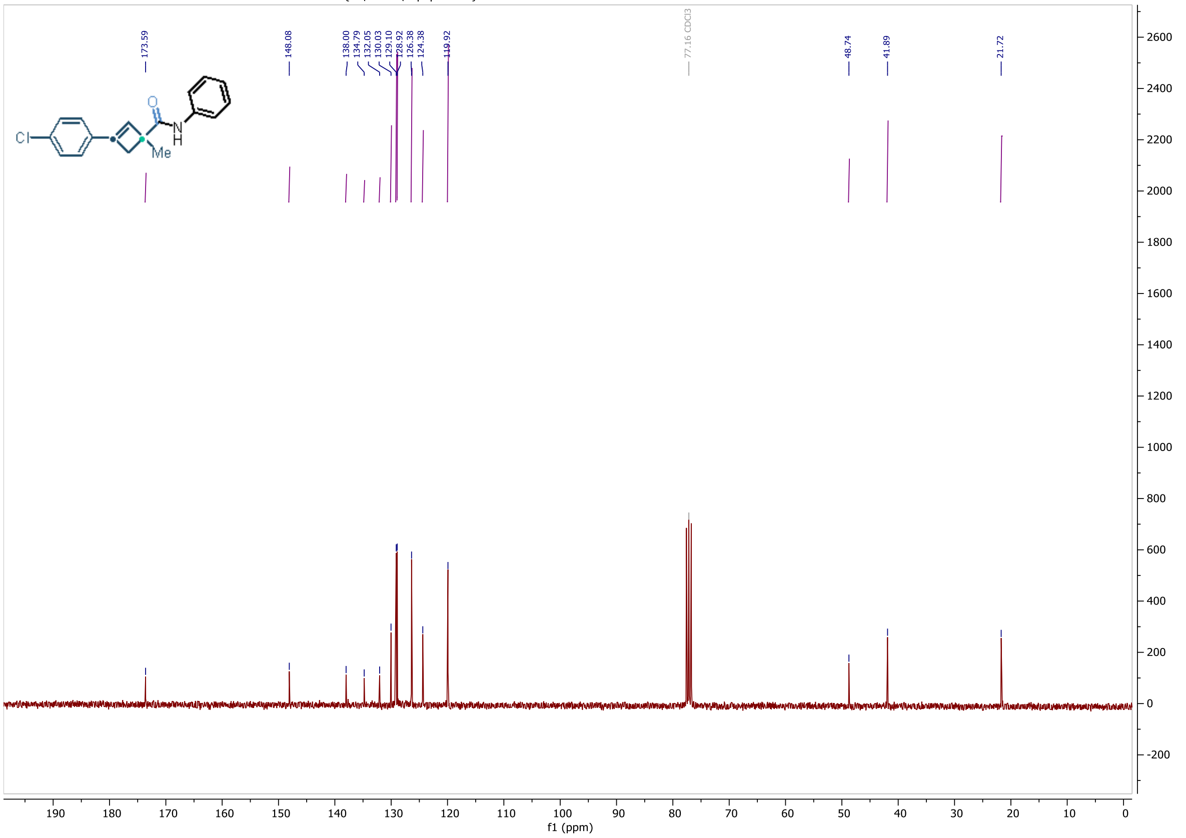

^{1}H NMR spectrum of **4ak** (300 MHz, $CDCl_3$)

260304.311.10.fid — Yu-kun Liu LYK-24-2-108 — Au1H CDCl3 {C:\Bruker\TopSpin3.6.2} 2603 11

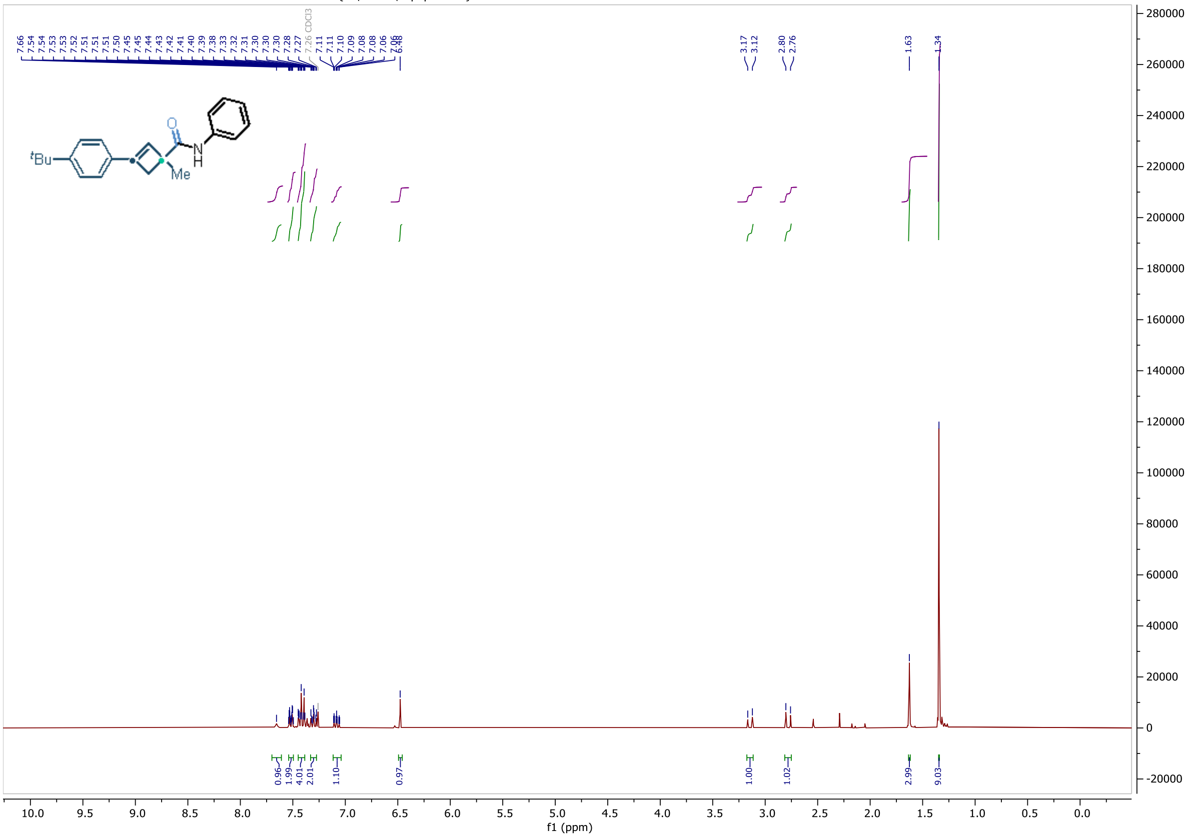

^{13}C NMR spectrum of **4ak** (75 MHz, $CDCl_3$)

260304.311.11.fid — Yu-kun Liu LYK-24-2-108 — Au13C CDCl3 {C:\Bruker\TopSpin3.6.2} 2603 11

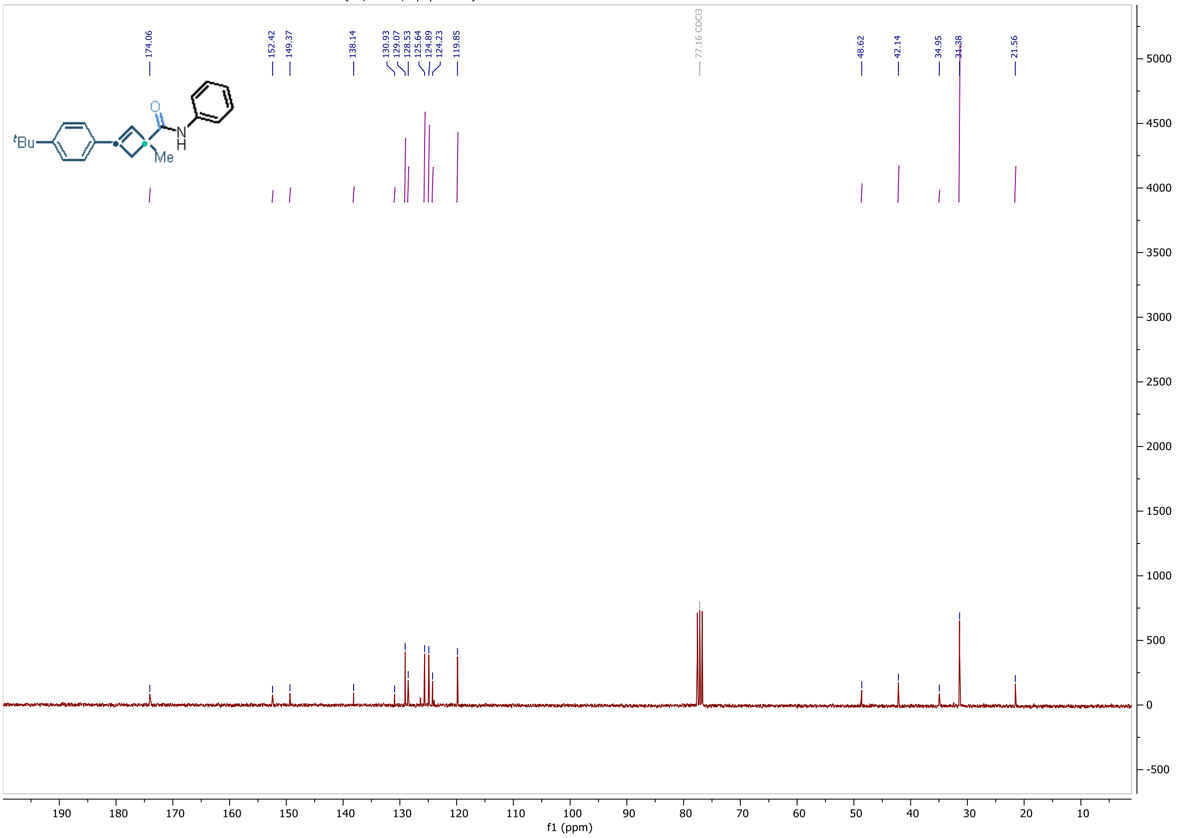

^{1}H NMR spectrum of **4al** (300 MHz, $CDCl_3$)

260424.313.10.fid — Yu-Kun Liu LYK-24-2-136 — Au1H CDCl3 {C:\Bruker\TopSpin3.6.2} 2604 13

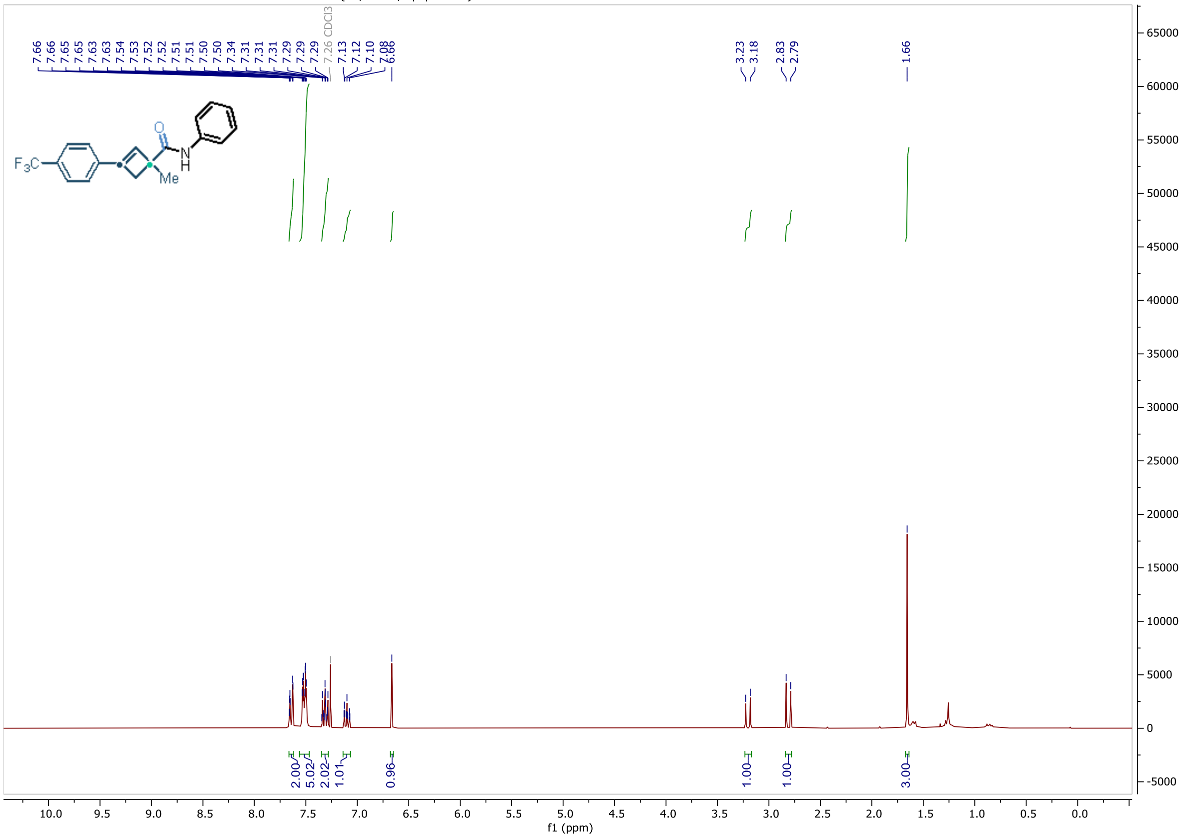

^{13}C NMR spectrum of **4al** (75 MHz, $CDCl_3$)

260424.313.11.fid — Yu-Kun Liu LYK-24-2-136 — Au13C CDCl3 {C:\Bruker\TopSpin3.6.2} 2604 13

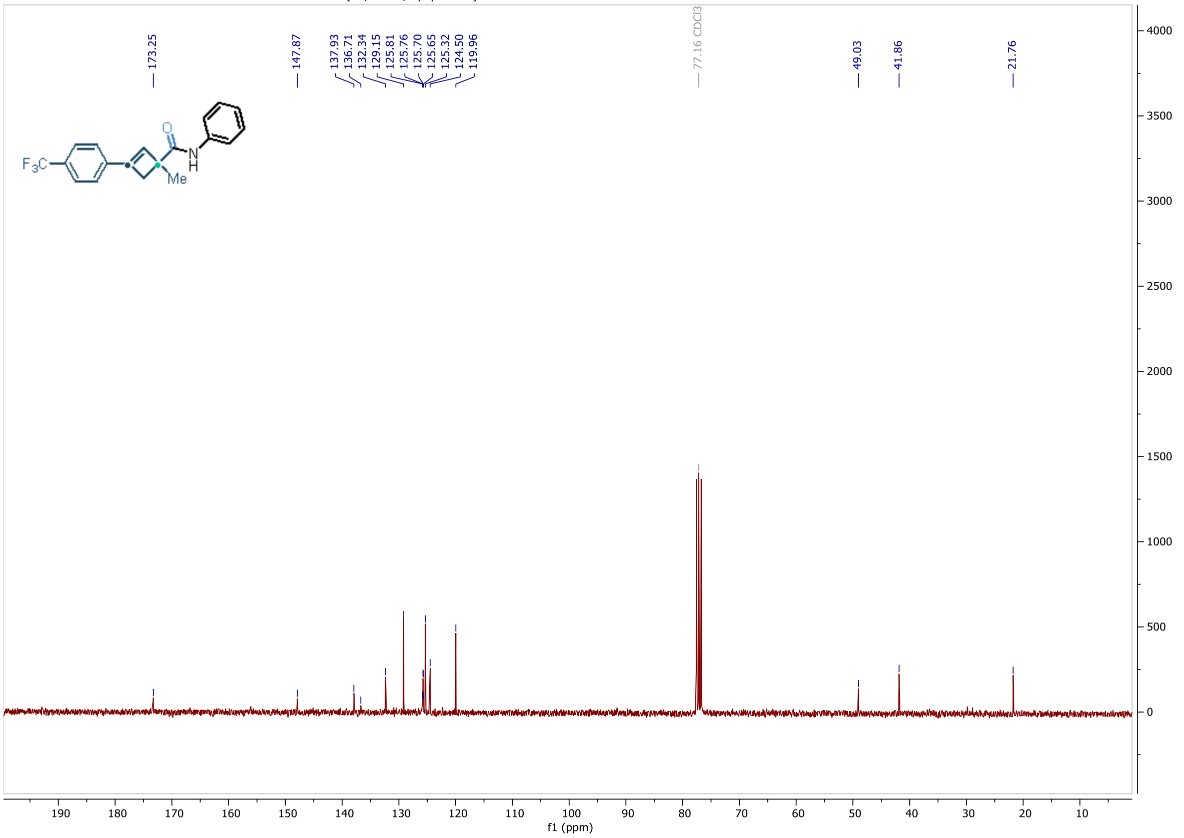

^{19}F NMR spectrum of **4al** (282 MHz, $CDCl_3$)

260424.313.12.fid — Yu-Kun Liu LYK-24-2-136 — Au19F CDCl3 {C:\Bruker\TopSpin3.6.2} 2604 13

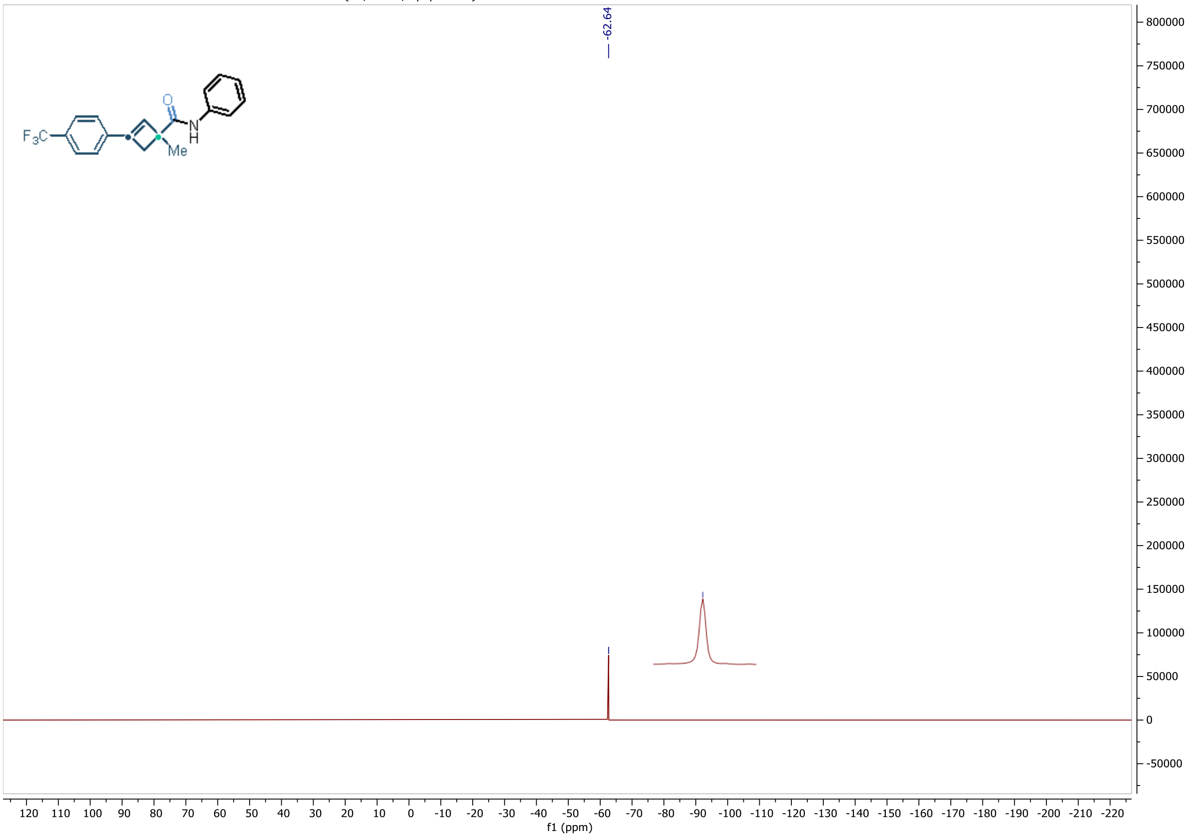

^{1}H NMR spectrum of **4am** (300 MHz, $CDCl_3$)

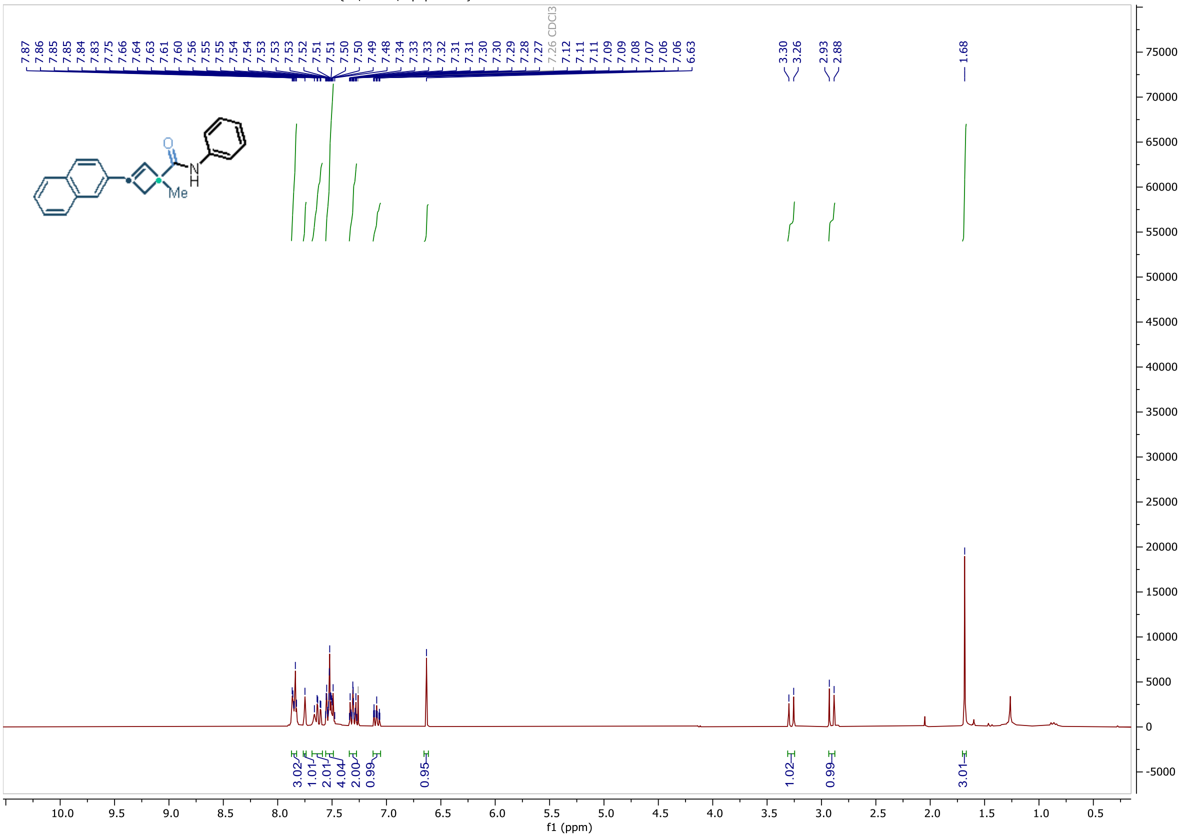

^{13}C NMR spectrum of **4am** (75 MHz, $CDCl_3$)

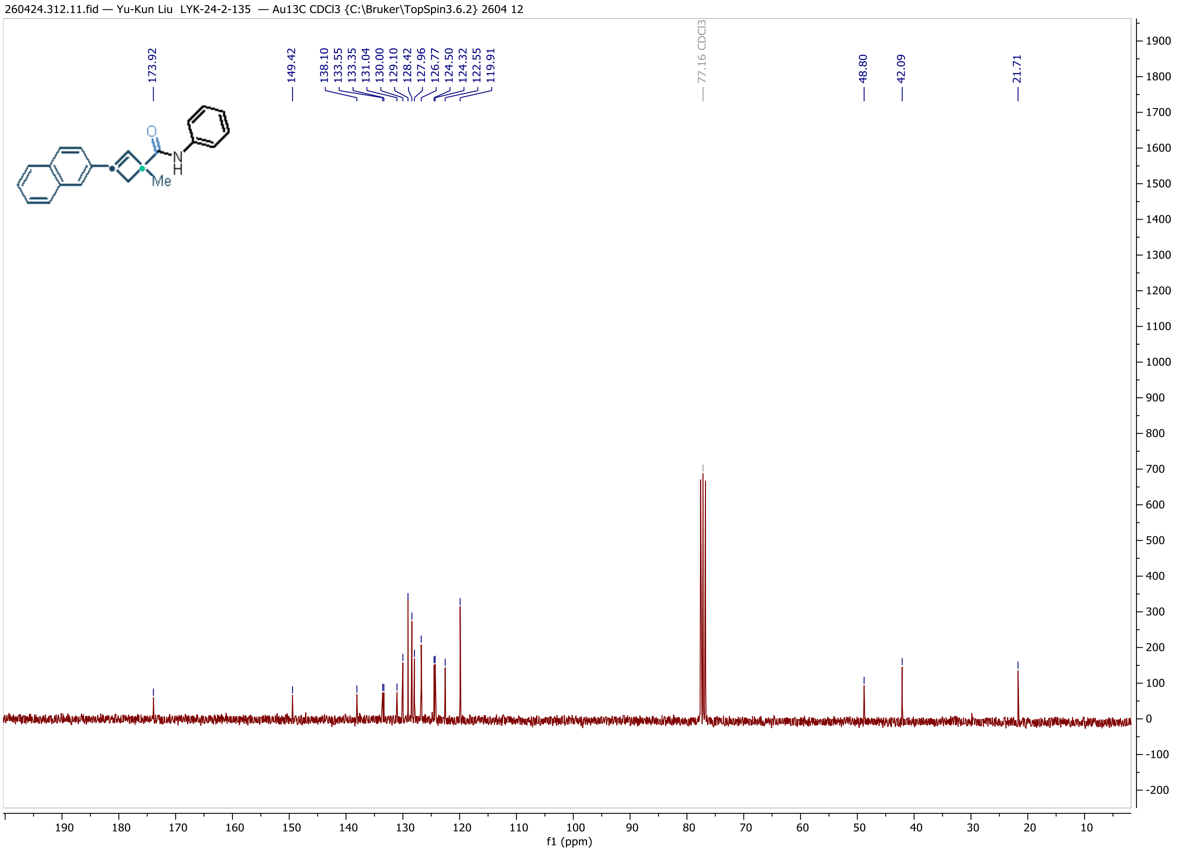

^{1}H NMR spectrum of **4an** (300 MHz, $CDCl_3$)

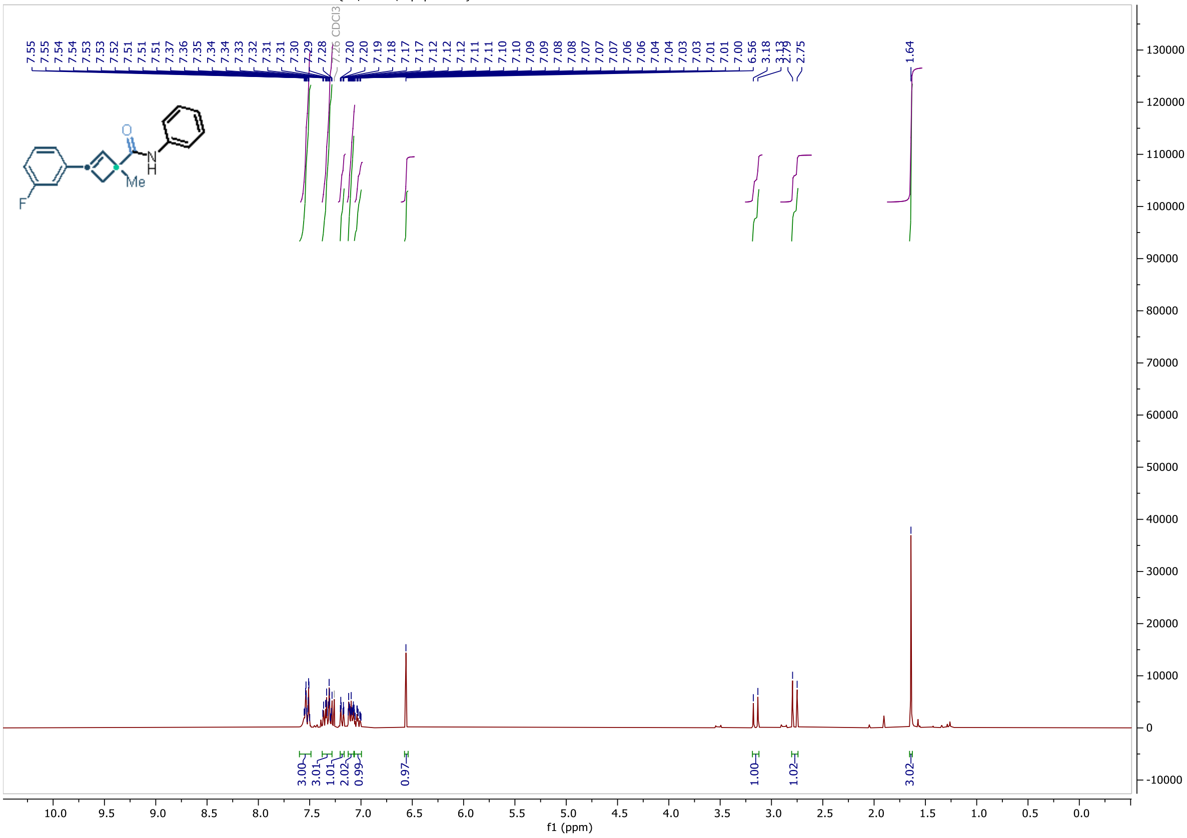

^{13}C NMR spectrum of **4an** (75 MHz, $CDCl_3$)

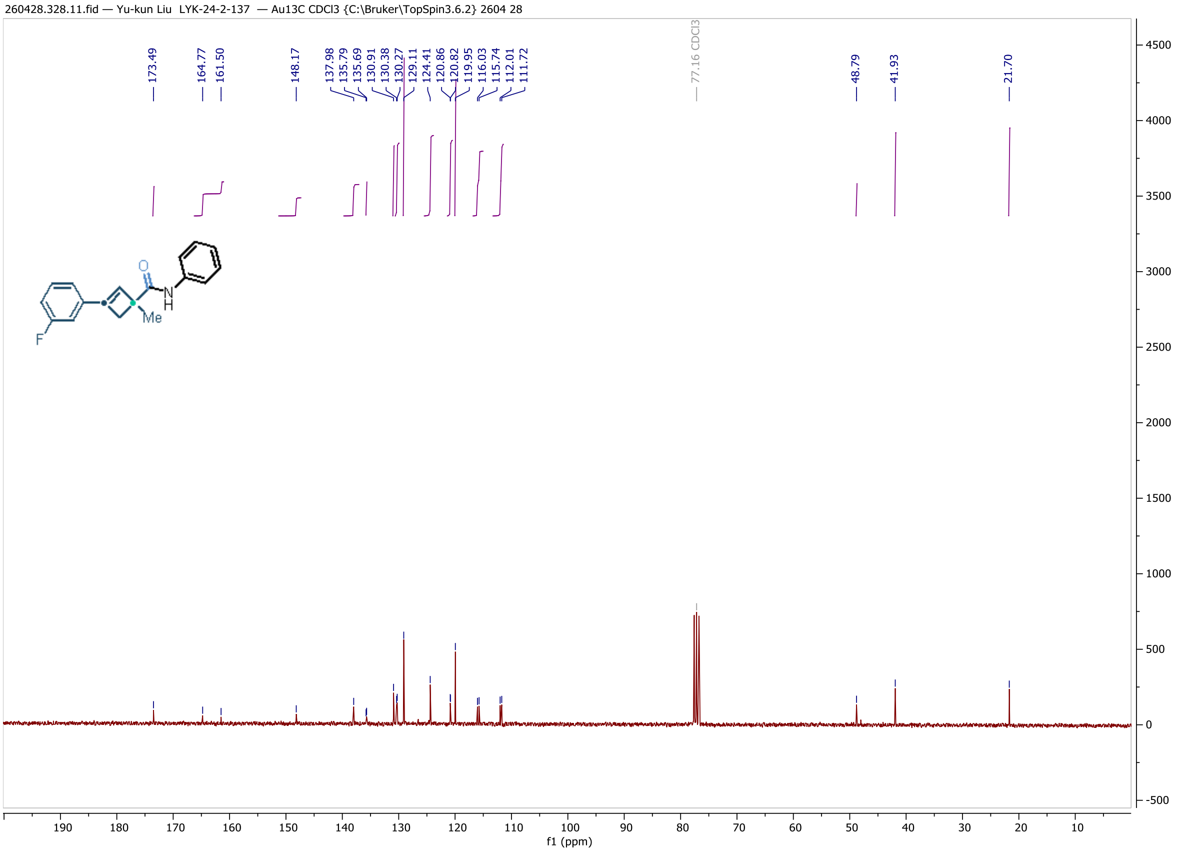

^{19}F NMR spectrum of **4an** (282 MHz, $CDCl_3$)

260428.328.12.fid — Yu-kun Liu LYK-24-2-137 — Au19F CDCl3 {C:\Bruker\TopSpin3.6.2} 2604 28

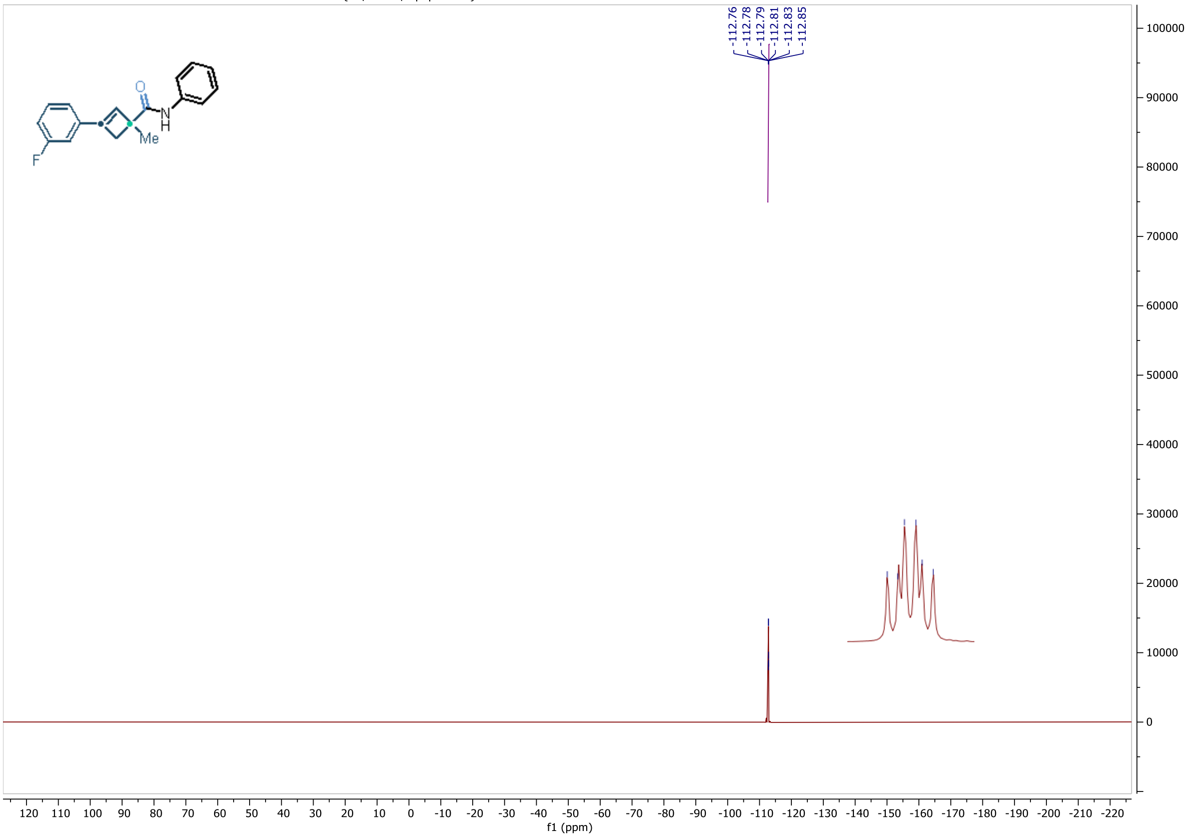

^{1}H NMR spectrum of **4ao** (300 MHz, $CDCl_3$)

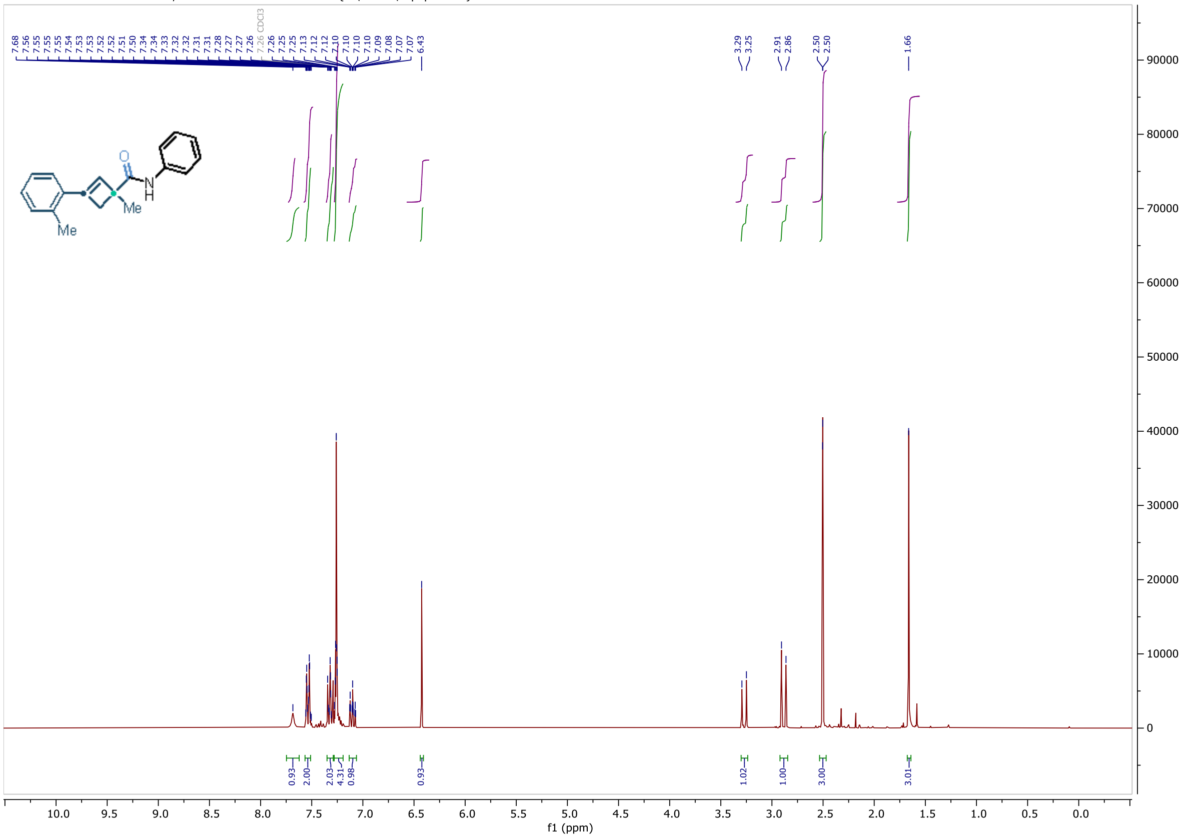

^{13}C NMR spectrum of **4ao** (75 MHz, $CDCl_3$)

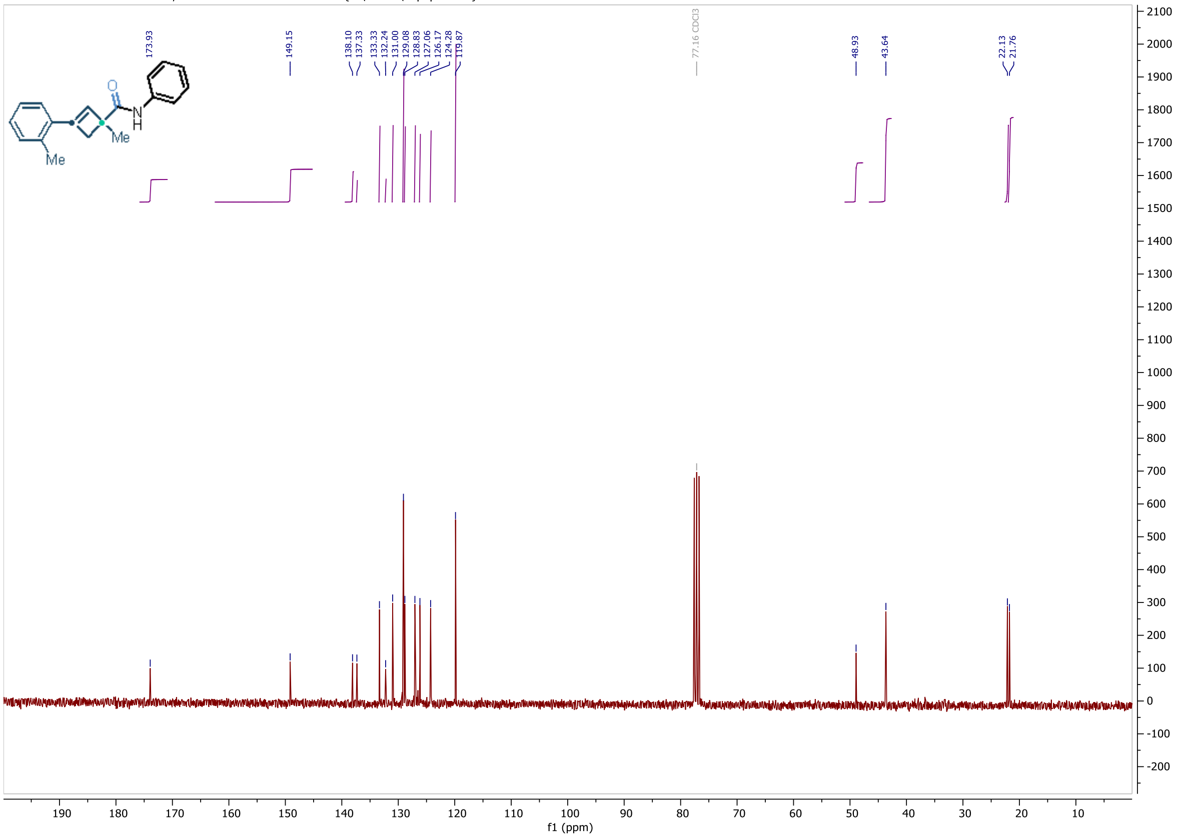

^{1}H NMR spectrum of **4ap** (300 MHz, $CDCl_3$)

260305.304.10.fid — Yu-kun Liu LYK-24-2-111 — Au1H CDCl3 {C:\Bruker\TopSpin3.6.2} 2603 4

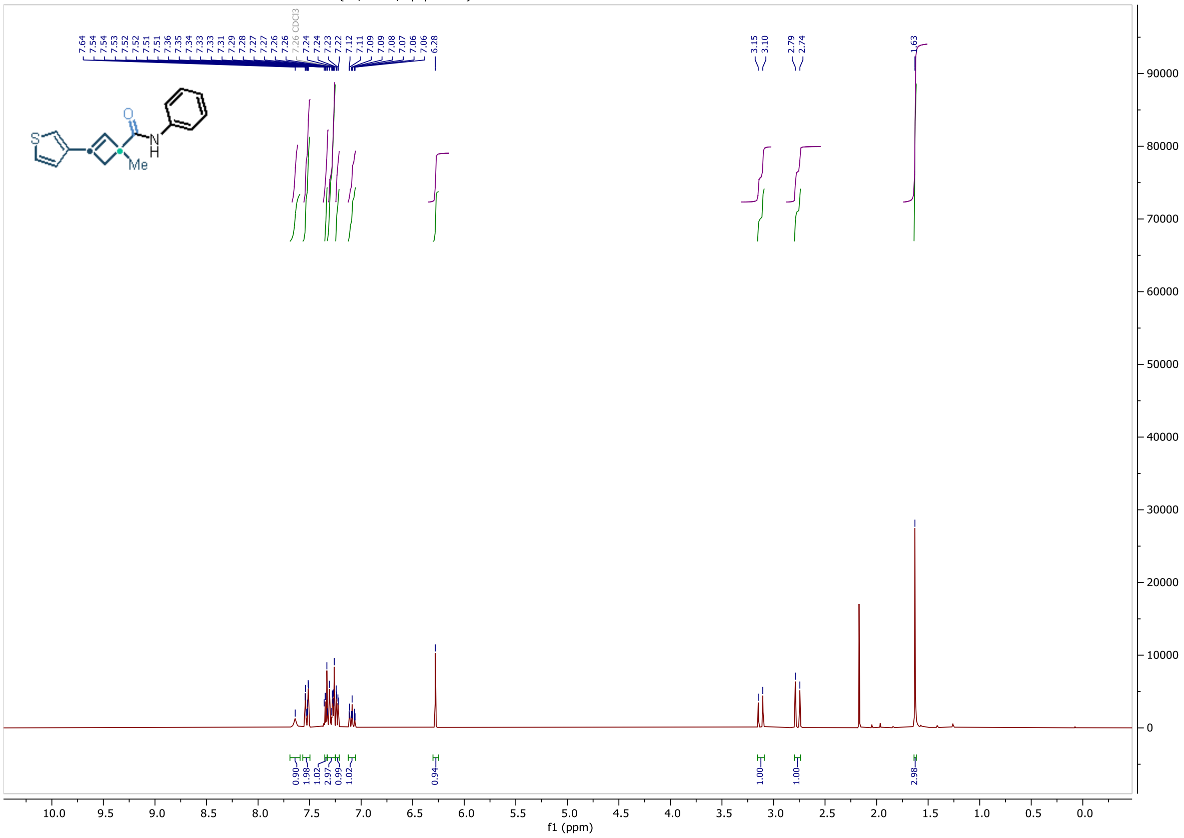

^{13}C NMR spectrum of **4ap** (75 MHz, $CDCl_3$)

260305.304.11.fid — Yu-kun Liu LYK-24-2-111 — Au13C CDCl3 {C:\Bruker\TopSpin3.6.2} 2603 4

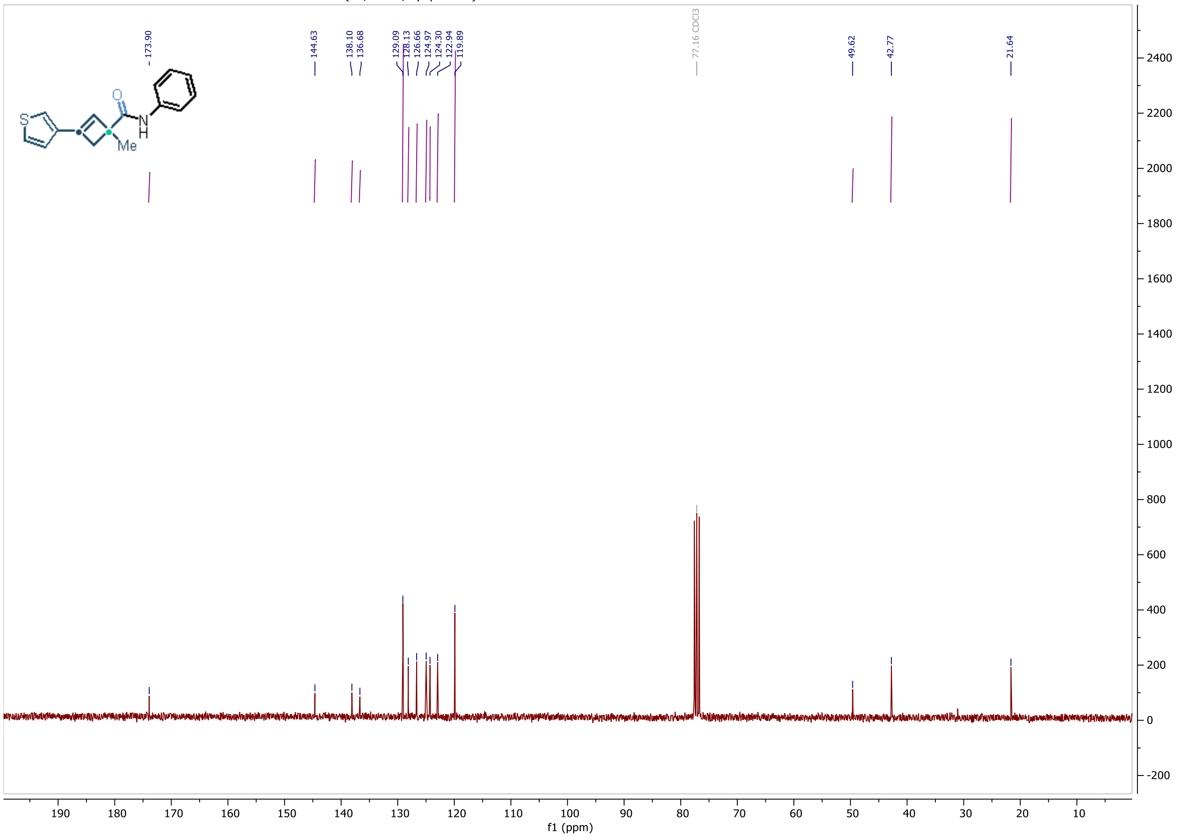

^{1}H NMR spectrum of **4aq** (300 MHz, $CDCl_3$)

260305.305.10.fid — Yu-kun Liu LYK-24-2-112 — Au1H CDCl3 {C:\Bruker\TopSpin3.6.2} 2603 5

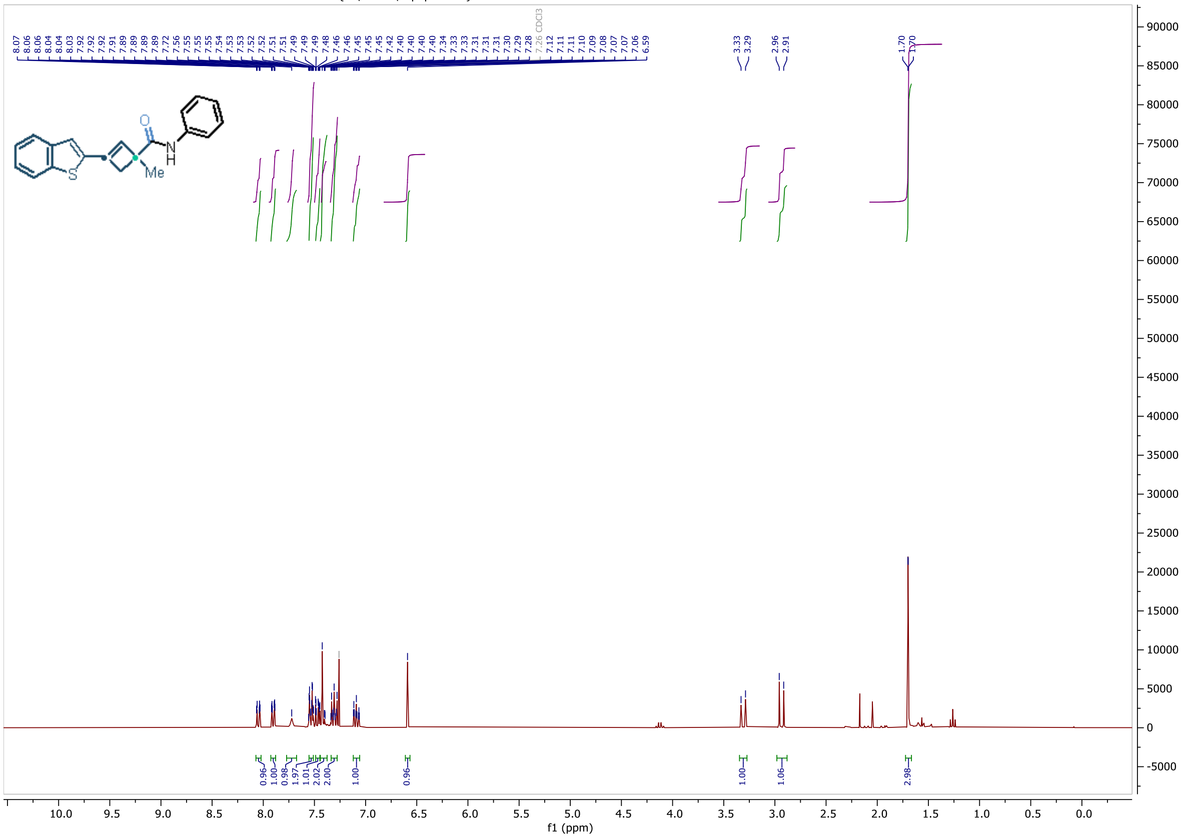

^{13}C NMR spectrum of **4aq** (75 MHz, $CDCl_3$)

260305.305.11.fid — Yu-kun Liu LYK-24-2-112 — Au13C CDCl3 {C:\Bruker\TopSpin3.6.2} 2603 5

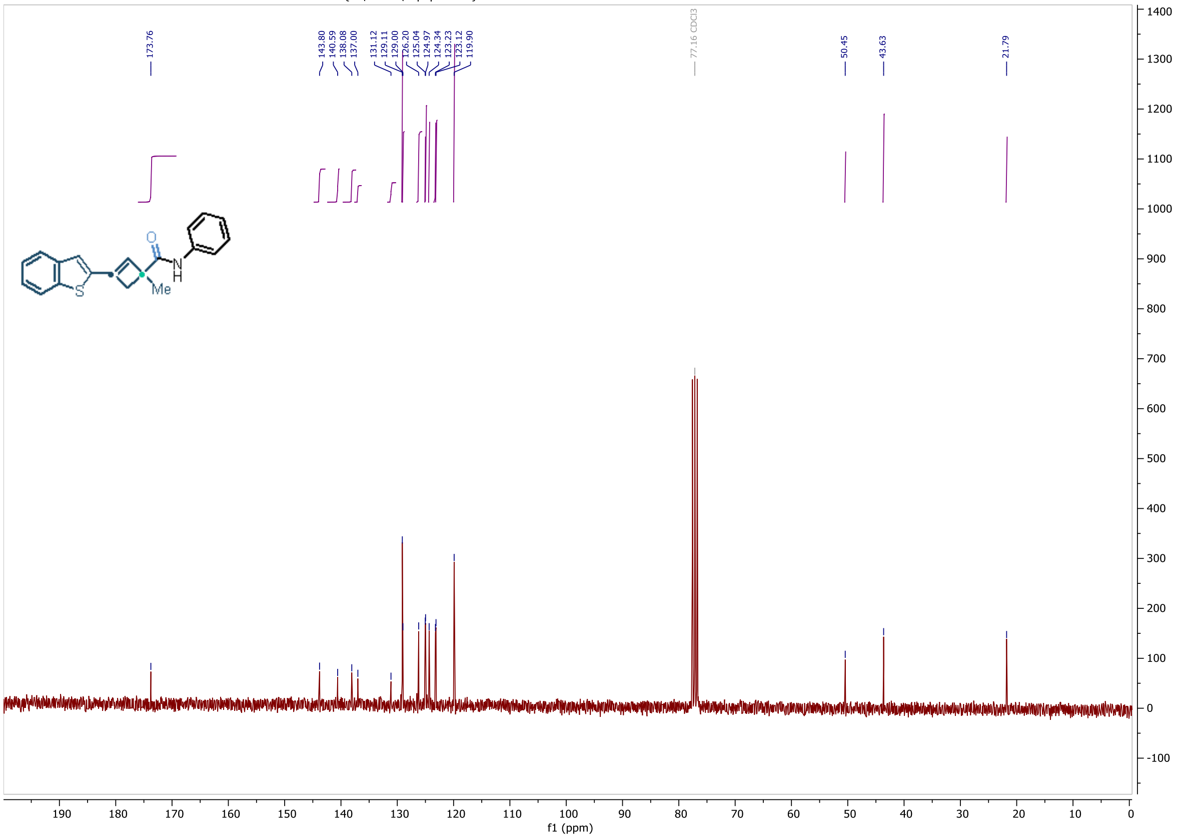

^{1}H NMR spectrum of **4ar** (300 MHz, $CDCl_3$)

260305.303.10.fid — Yu-kun Liu LYK-24-2-110 — Au1H CDCl3 {C:\Bruker\TopSpin3.6.2} 2603 3

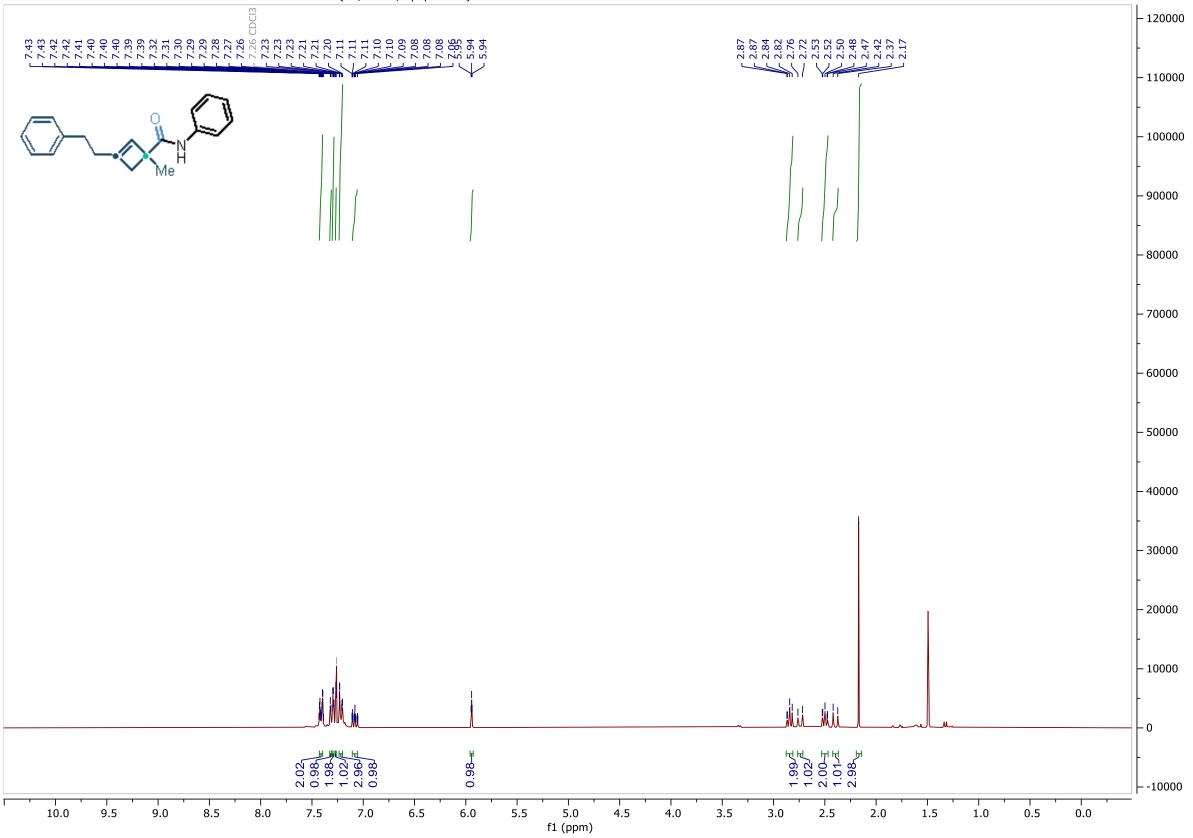

^{13}C NMR spectrum of **4ar** (75 MHz, $CDCl_3$)

260305.303.11.fid — Yu-kun Liu LYK-24-2-110 — Au13C CDCl3 {C:\Bruker\TopSpin3.6.2} 2603 3

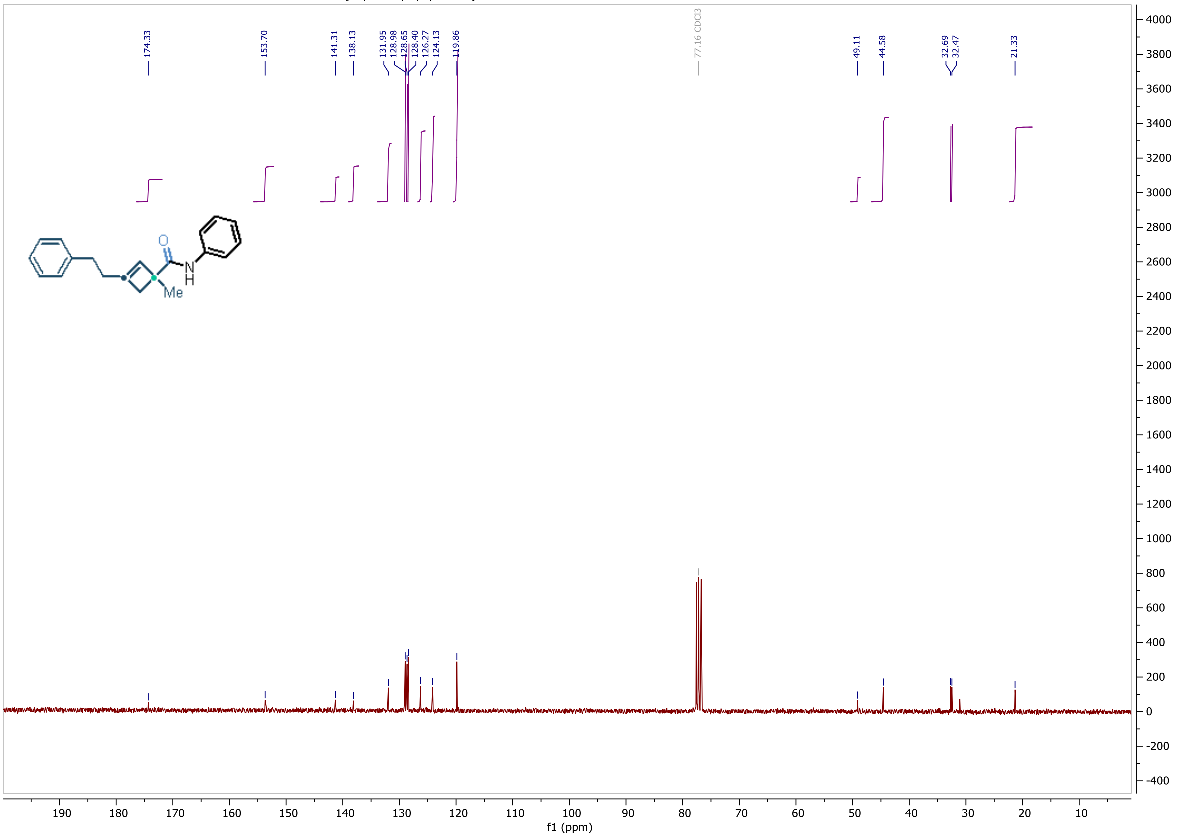

^{1}H NMR spectrum of **4as** (300 MHz, CDCl$_3$)

260428.329.10.fid — Yu-kun Liu LYK-24-2-138 — Au1H CDCl3 {C:\Bruker\TopSpin3.6.2} 2604 29

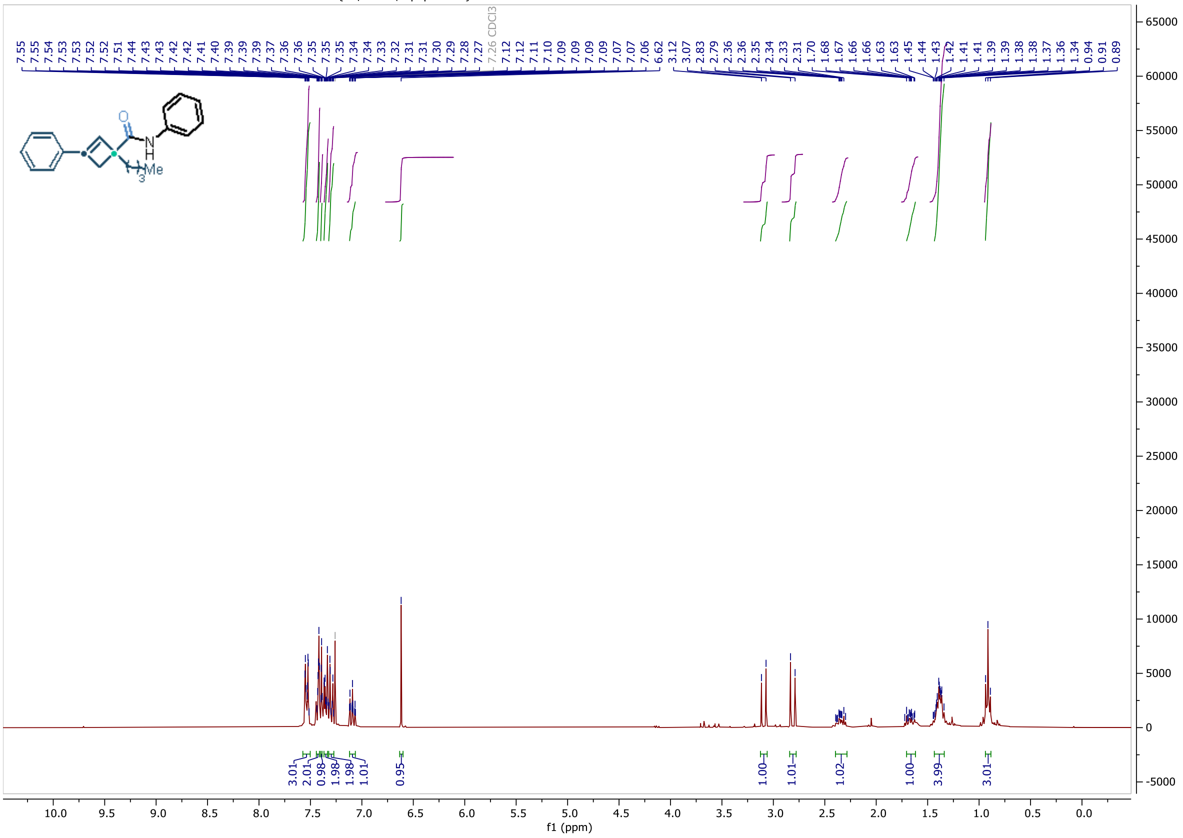

^{13}C NMR spectrum of **4as** (75 MHz, CDCl$_3$)

260428.329.11.fid — Yu-kun Liu LYK-24-2-138 — Au13C CDCl3 {C:\Bruker\TopSpin3.6.2} 2604 29

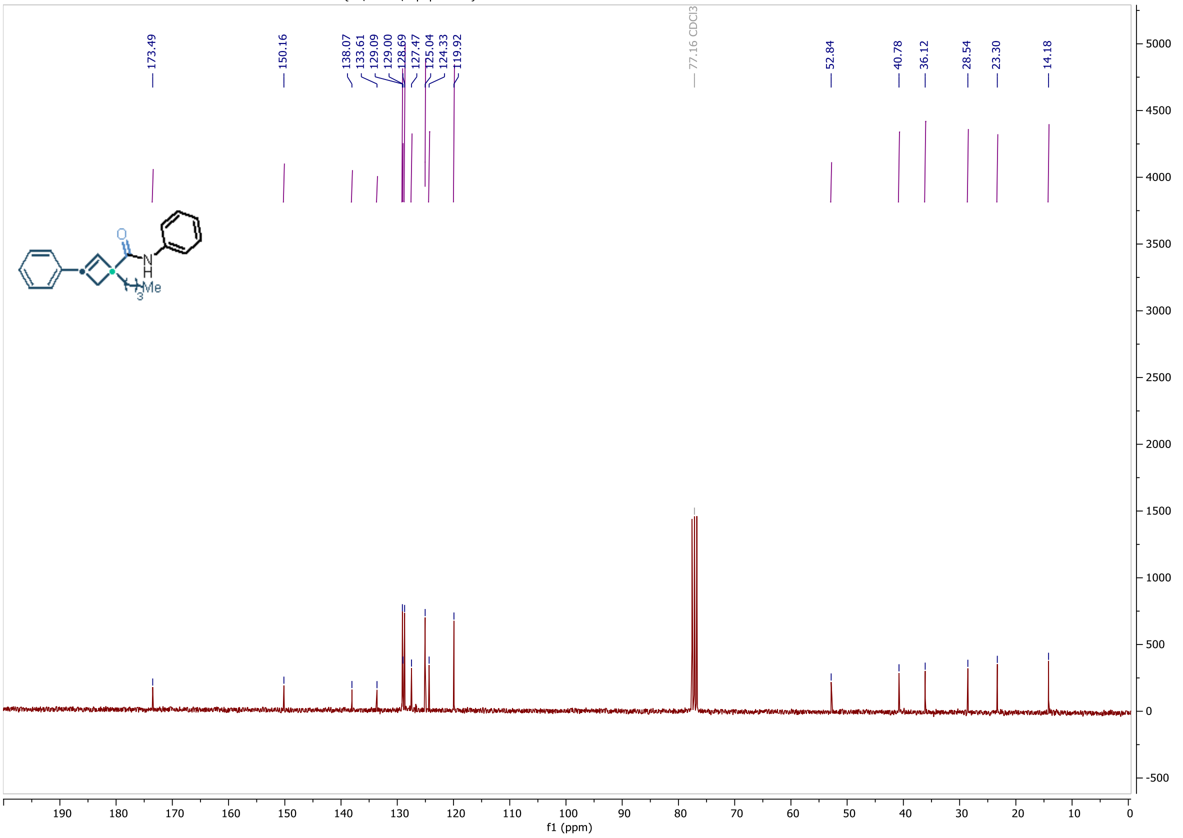

^{1}H NMR spectrum of **4at** (300 MHz, $CDCl_3$)

260428.330.10.fid — Yu-kun Liu LYK-24-2-139 — Au1H CDCl3 {C:\Bruker\TopSpin3.6.2} 2604 30

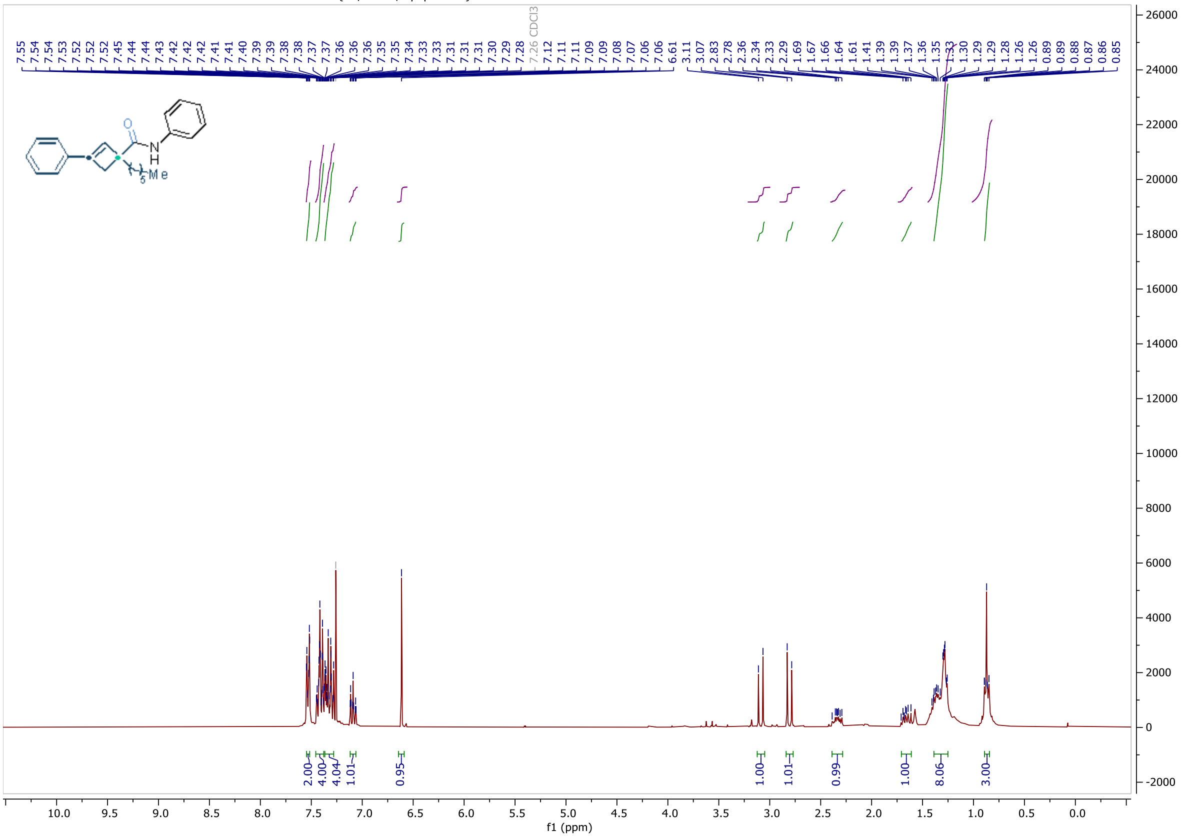

^{13}C NMR spectrum of **4at** (75 MHz, $CDCl_3$)

260428.330.11.fid — Yu-kun Liu LYK-24-2-139 — Au13C CDCl3 {C:\Bruker\TopSpin3.6.2} 2604 30

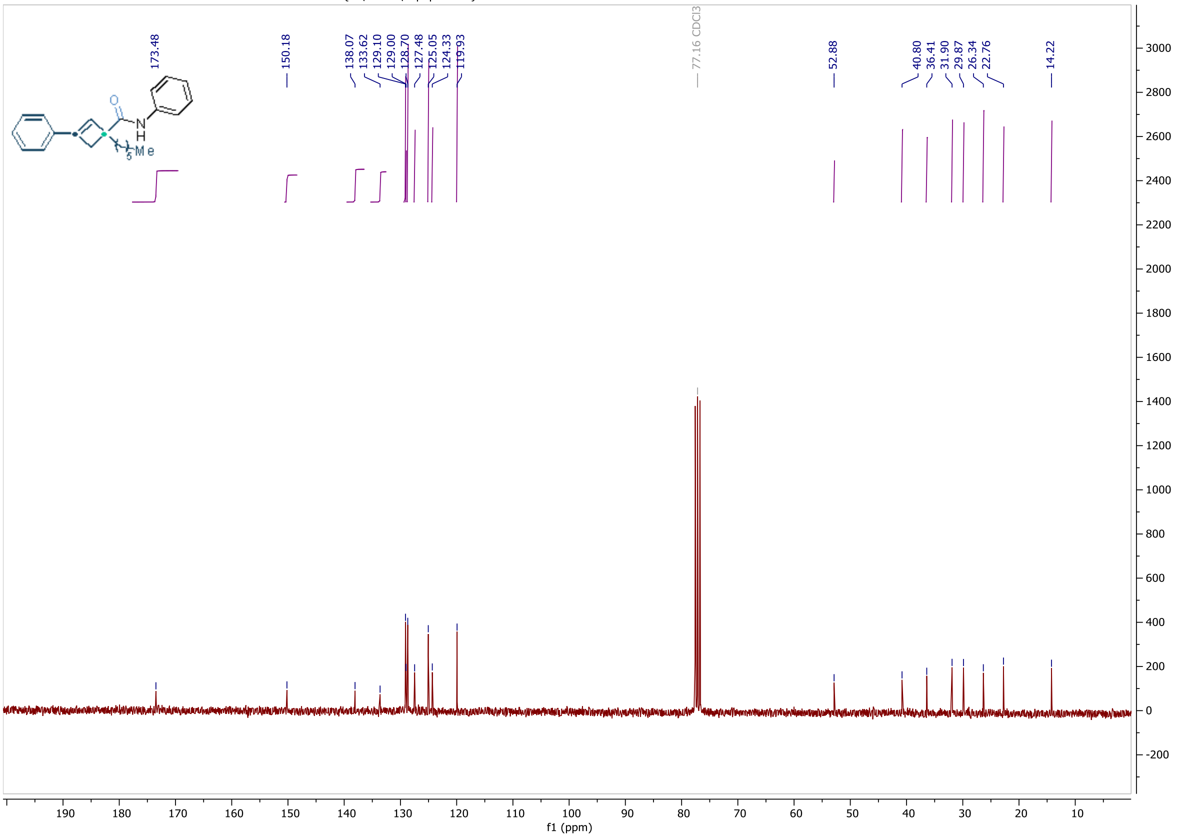

^{1}H NMR spectrum of **4aw** (300 MHz, $CDCl_3$)

260413.301.10.fid — Yu-kun Liu LYK-24-2-100-re-2 — Au1H CDCl3 {C:\Bruker\TopSpin3.6.2} 2604 1

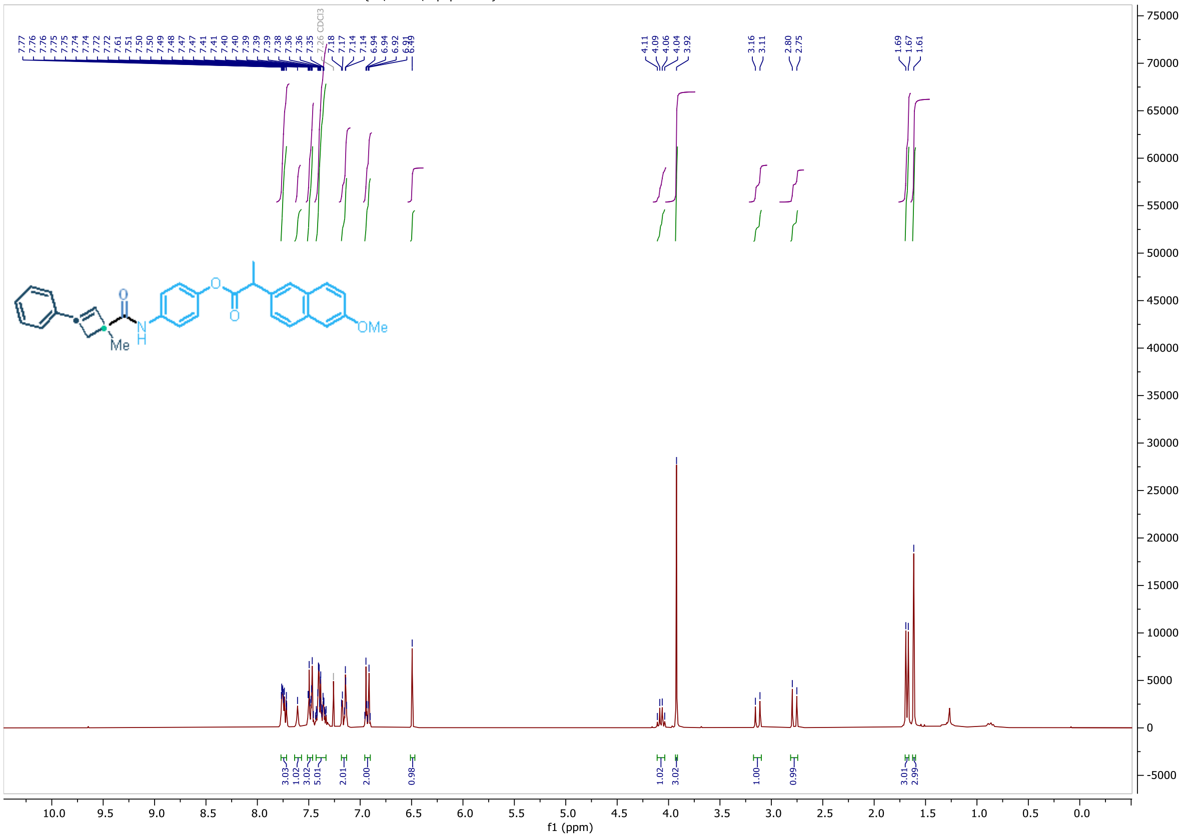

^{13}C NMR spectrum of **4aw** (75 MHz, $CDCl_3$)

260413.301.11.fid — Yu-kun Liu LYK-24-2-100-re-2 — Au13C CDCl3 {C:\Bruker\TopSpin3.6.2} 2604 1

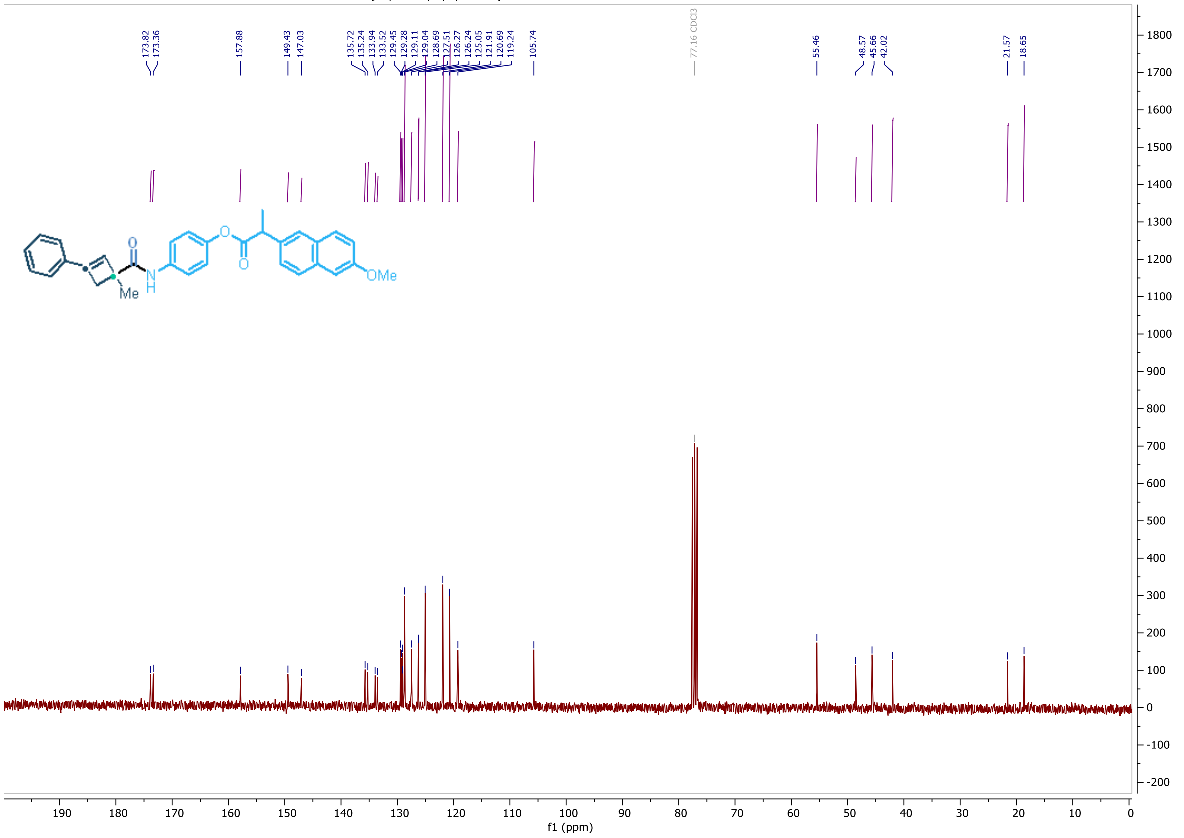

^{1}H NMR spectrum of **4ax** (300 MHz, $CDCl_3$)

260303.f308.10.fid — Yu-kun Liu LYK-24-2-101 — Au1H CDCl3 {C:\Bruker\TopSpin3.6.2} 2603 8

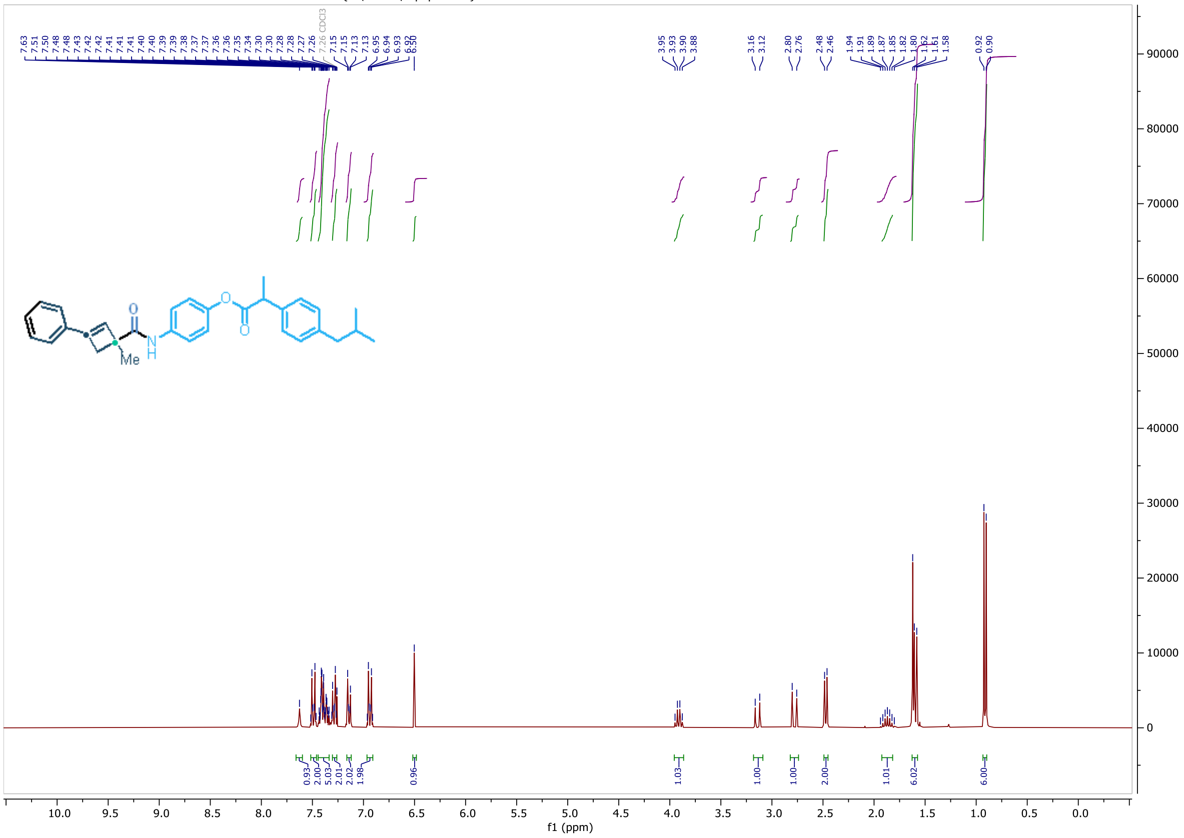

^{13}C NMR spectrum of **4ax** (75 MHz, $CDCl_3$)

260303.f308.11.fid — Yu-kun Liu LYK-24-2-101 — Au13C CDCl3 {C:\Bruker\TopSpin3.6.2} 2603 8

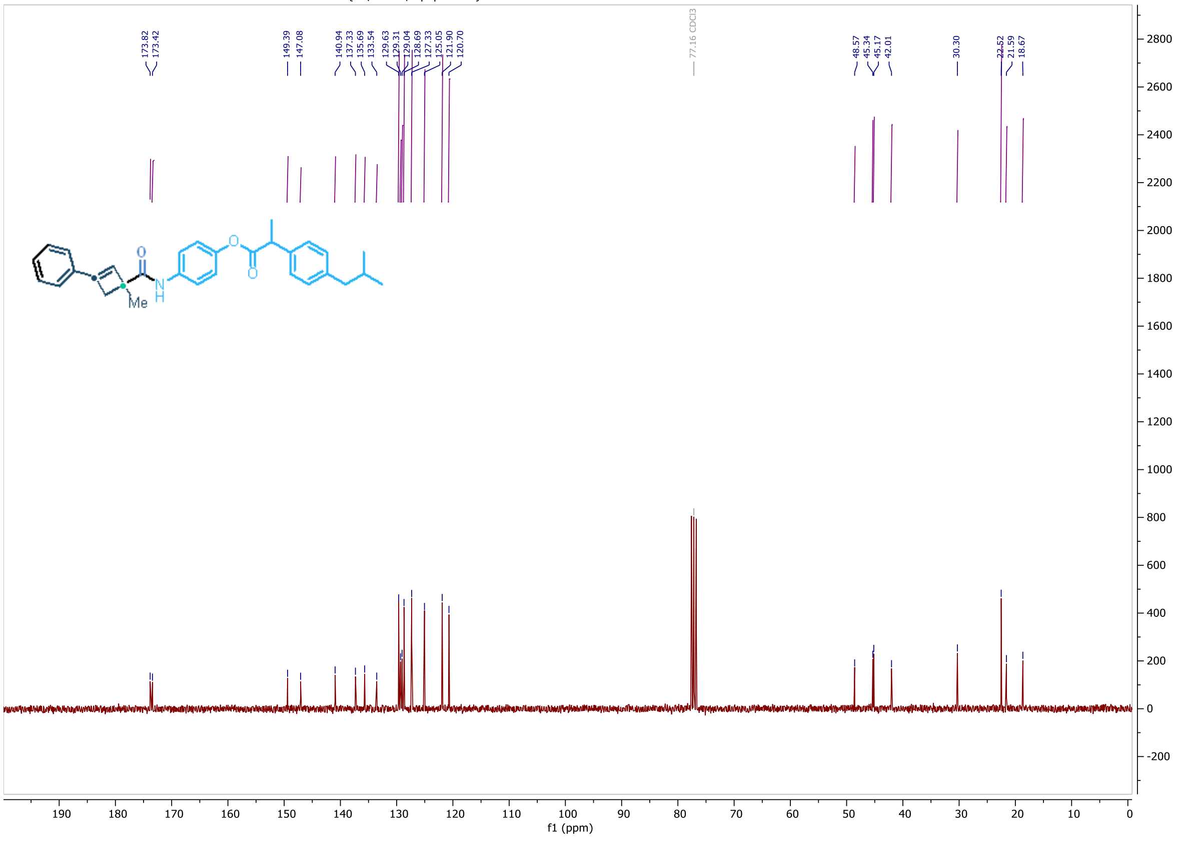

^{1}H NMR spectrum of **4ay** (300 MHz, $CDCl_3$)

260303.f309.10.fid — Yu-kun Liu LYK-24-2-102 — Au1H CDCl3 {C:\Bruker\TopSpin3.6.2} 2603 9

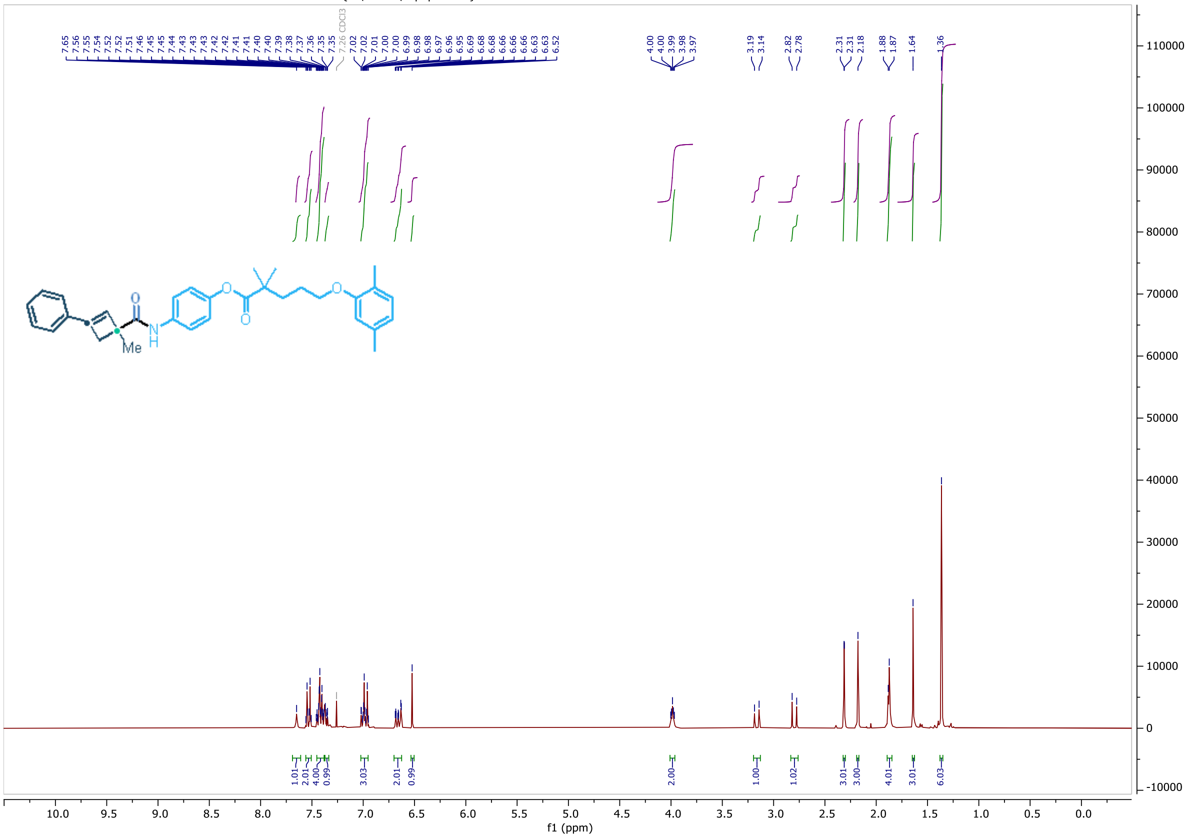

^{13}C NMR spectrum of **4ay** (75 MHz, $CDCl_3$)

260303.f309.11.fid — Yu-kun Liu LYK-24-2-102 — Au13C CDCl3 {C:\Bruker\TopSpin3.6.2} 2603 9

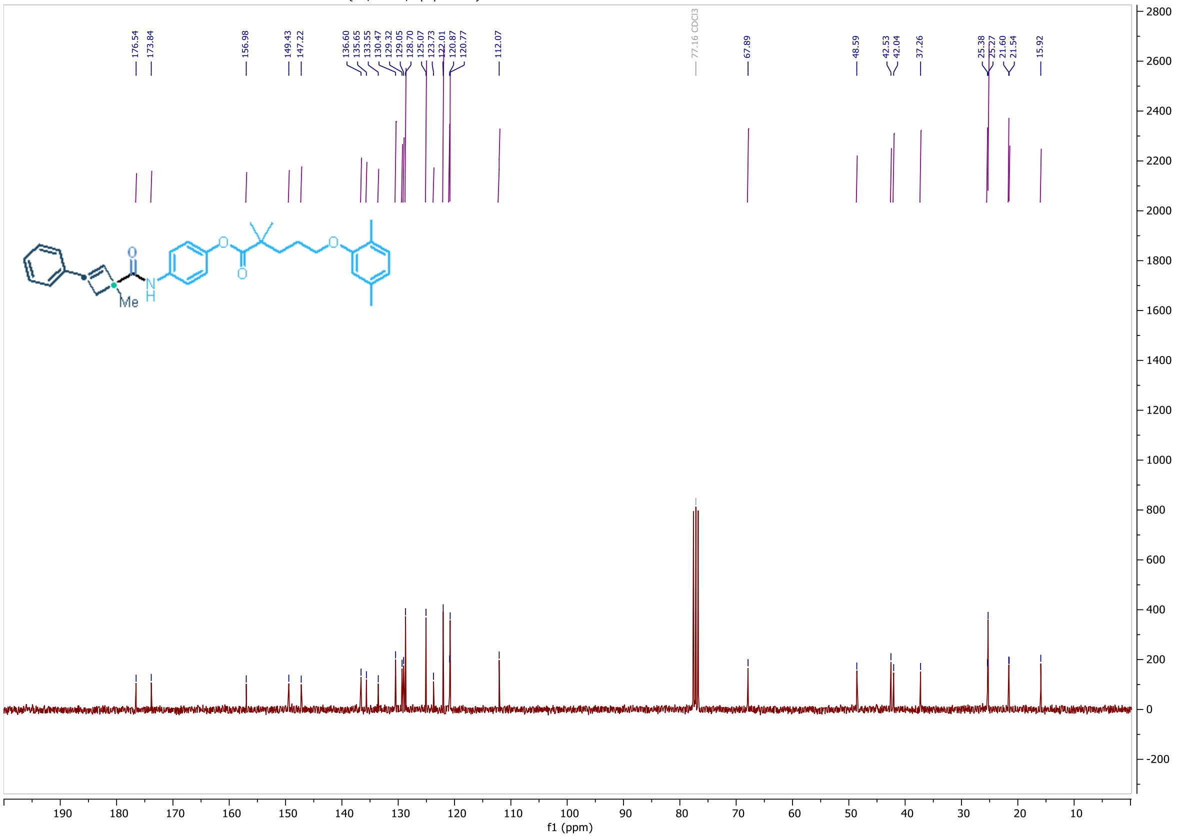

^1H NMR spectrum of **4az** (300 MHz, $CDCl_3$)

260303.f310.10.fid — Yu-kun Liu LYK-24-2-103 — Au1H CDCl3 {C:\Bruker\TopSpin3.6.2} 2603 10

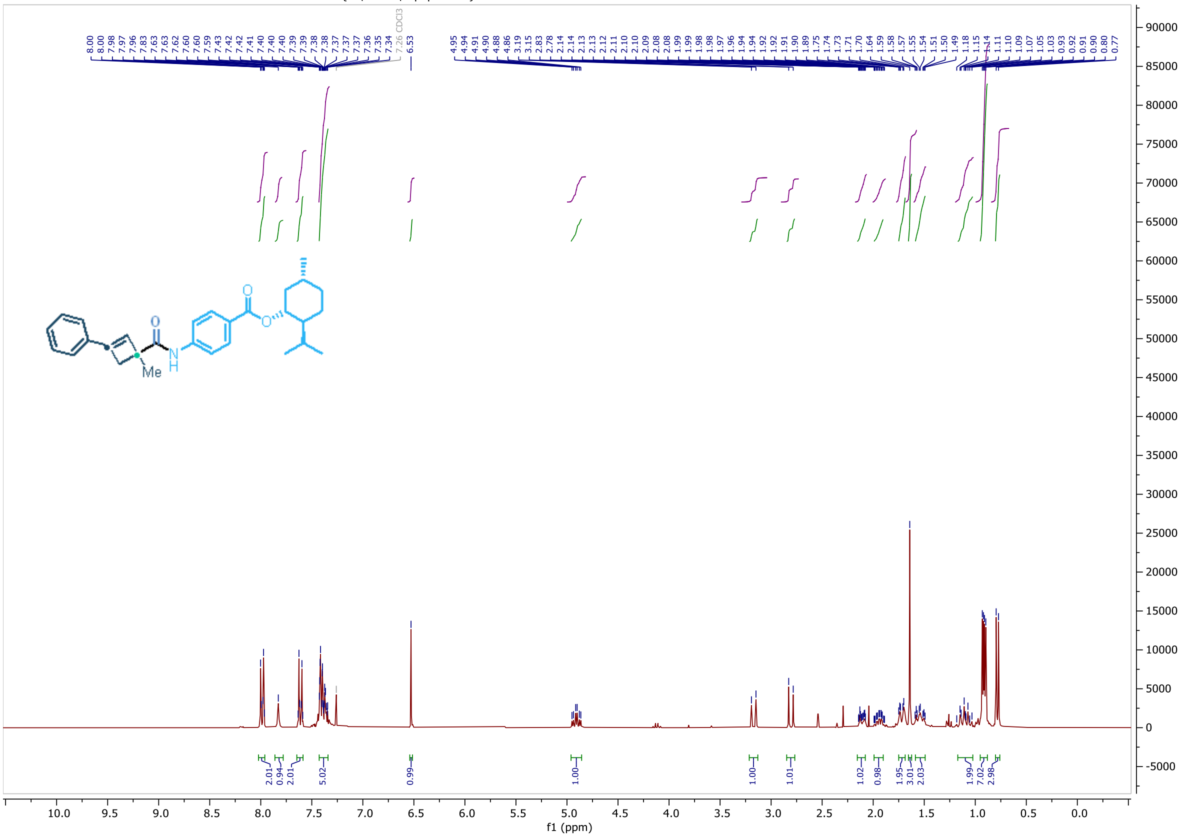

^{13}C NMR spectrum of **4az** (75 MHz, $CDCl_3$)

260303.f310.11.fid — Yu-kun Liu LYK-24-2-103 — Au13C CDCl3 {C:\Bruker\TopSpin3.6.2} 2603 10

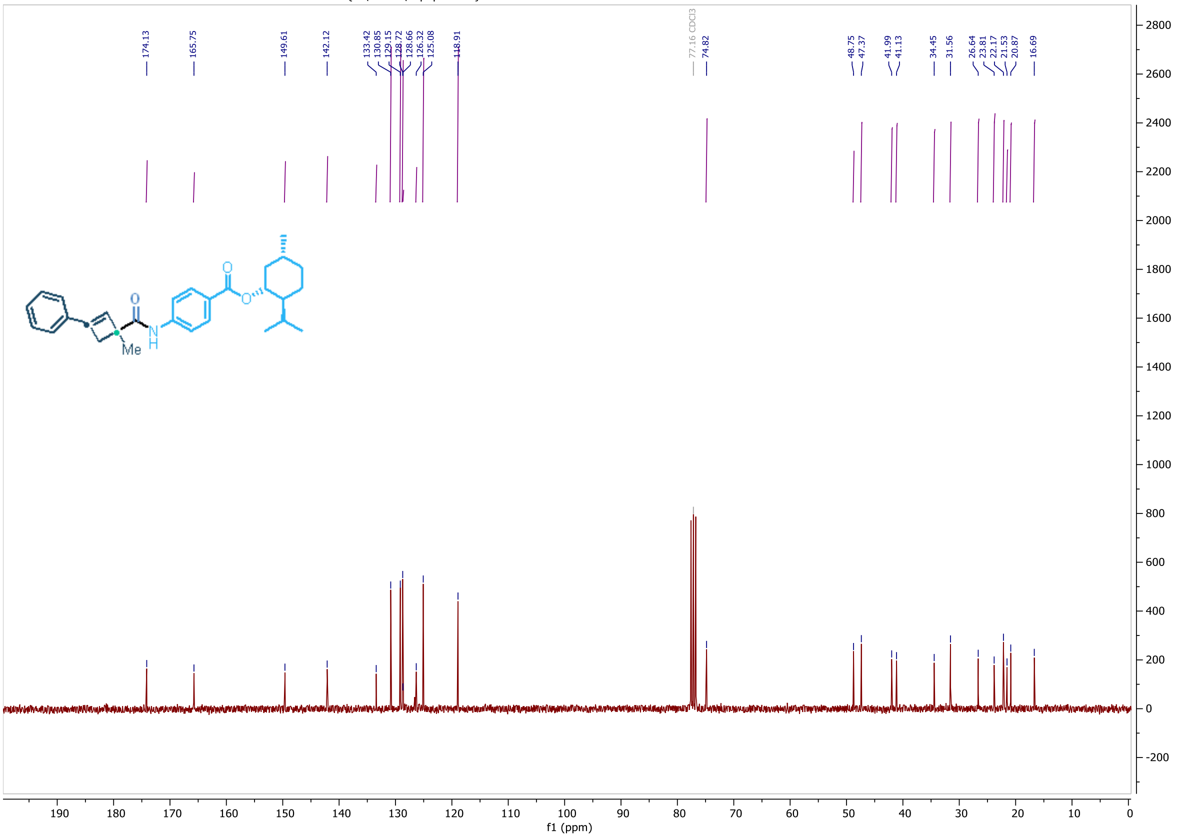

^{1}H NMR spectrum of **4bb** (300 MHz, $CDCl_3$)

260407.307.10.fid — Yu-kun Liu LYK-24-125 — Au1H CDCl3 {C:\Bruker\TopSpin3.6.2} 2604 7

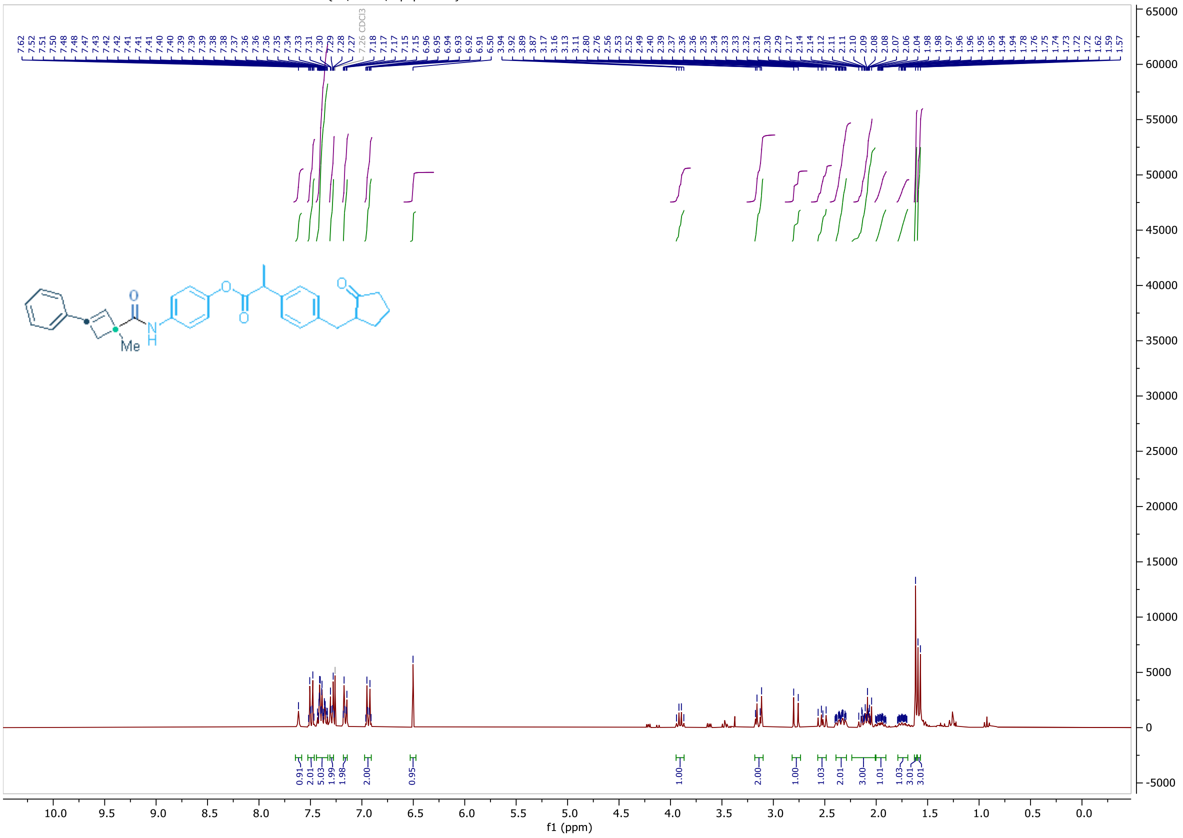

^{13}C NMR spectrum of **4bb** (75 MHz, $CDCl_3$)

260407.307.11.fid — Yu-kun Liu LYK-24-125 — Au13C CDCl3 {C:\Bruker\TopSpin3.6.2} 2604 7

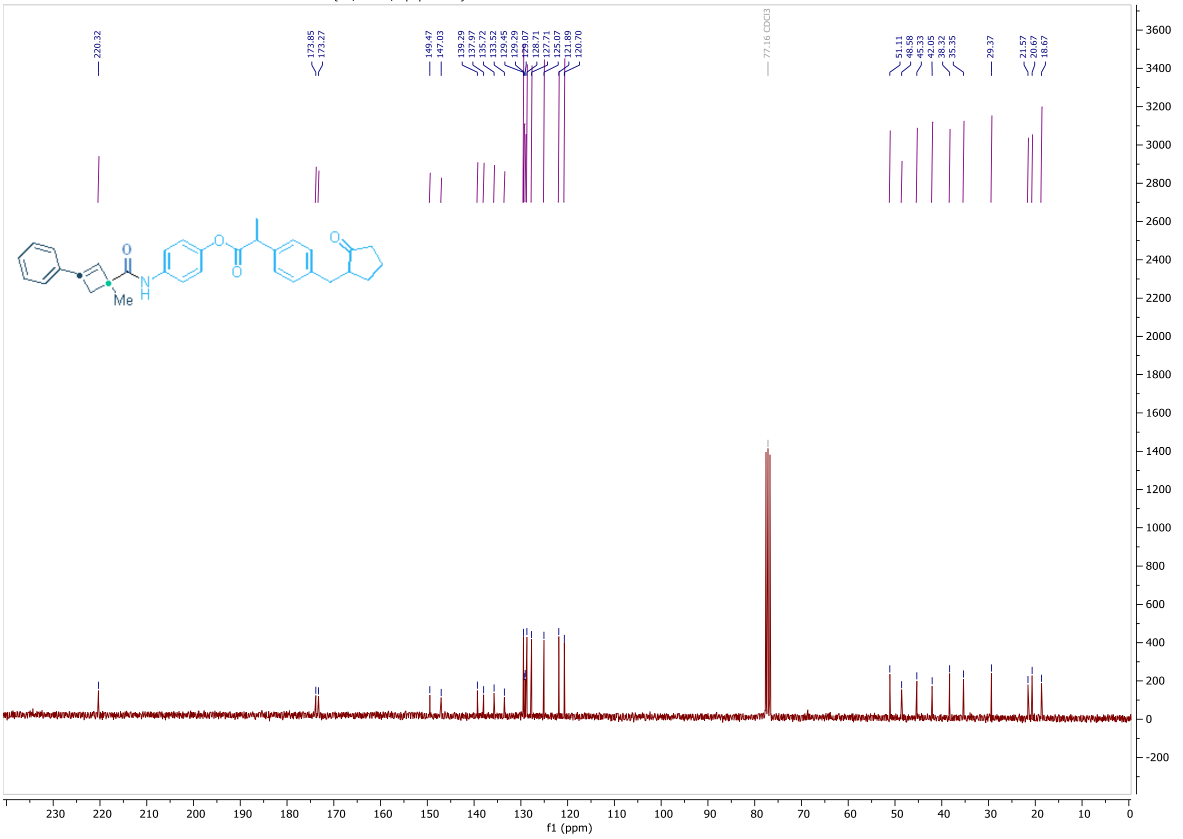

^{1}H NMR spectrum of **4bc** (300 MHz, $CDCl_3$)

260226.319.10.fid — Yu-kun Liu LYK-24-2-94 — Au1H CDCl3 {C:\Bruker\TopSpin3.6.2} 2602 19

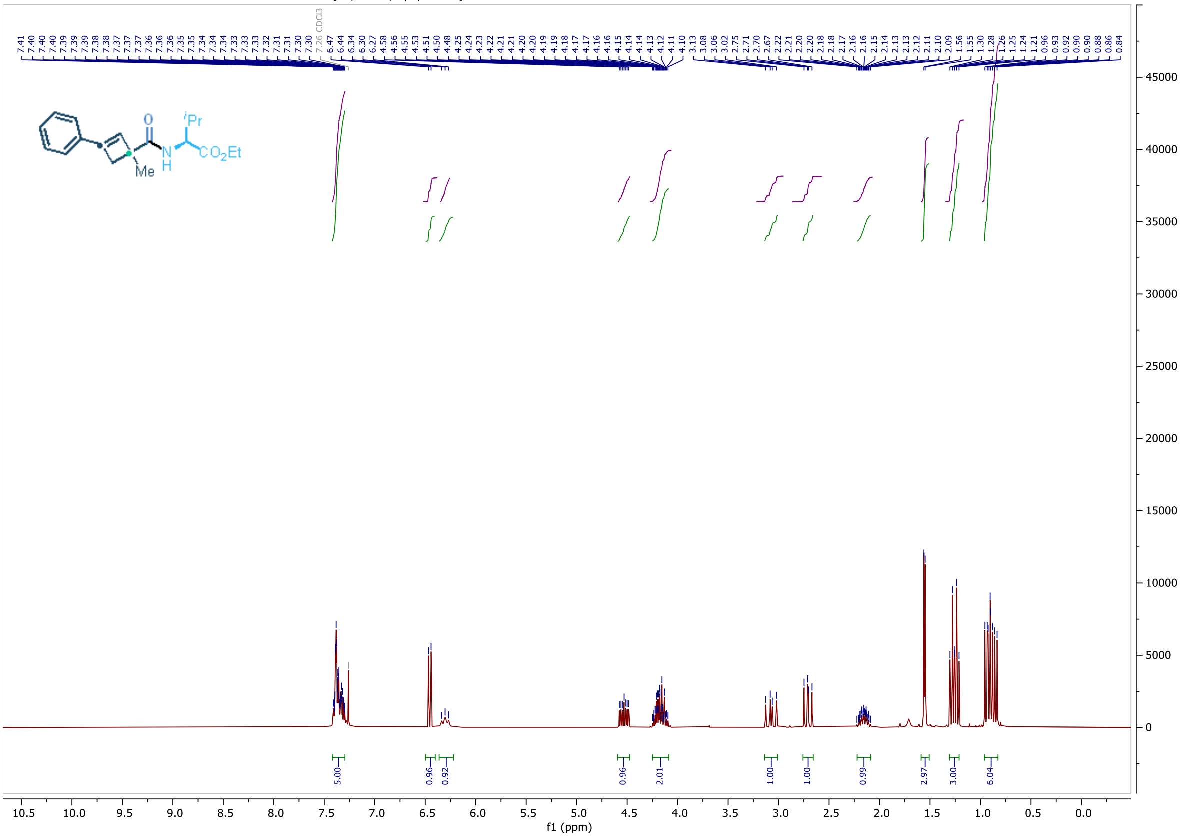

^{13}C NMR spectrum of **4bc** (75 MHz, $CDCl_3$)

260226.319.11.fid — Yu-kun Liu LYK-24-2-94 — Au13C CDCl3 {C:\Bruker\TopSpin3.6.2} 2602 19

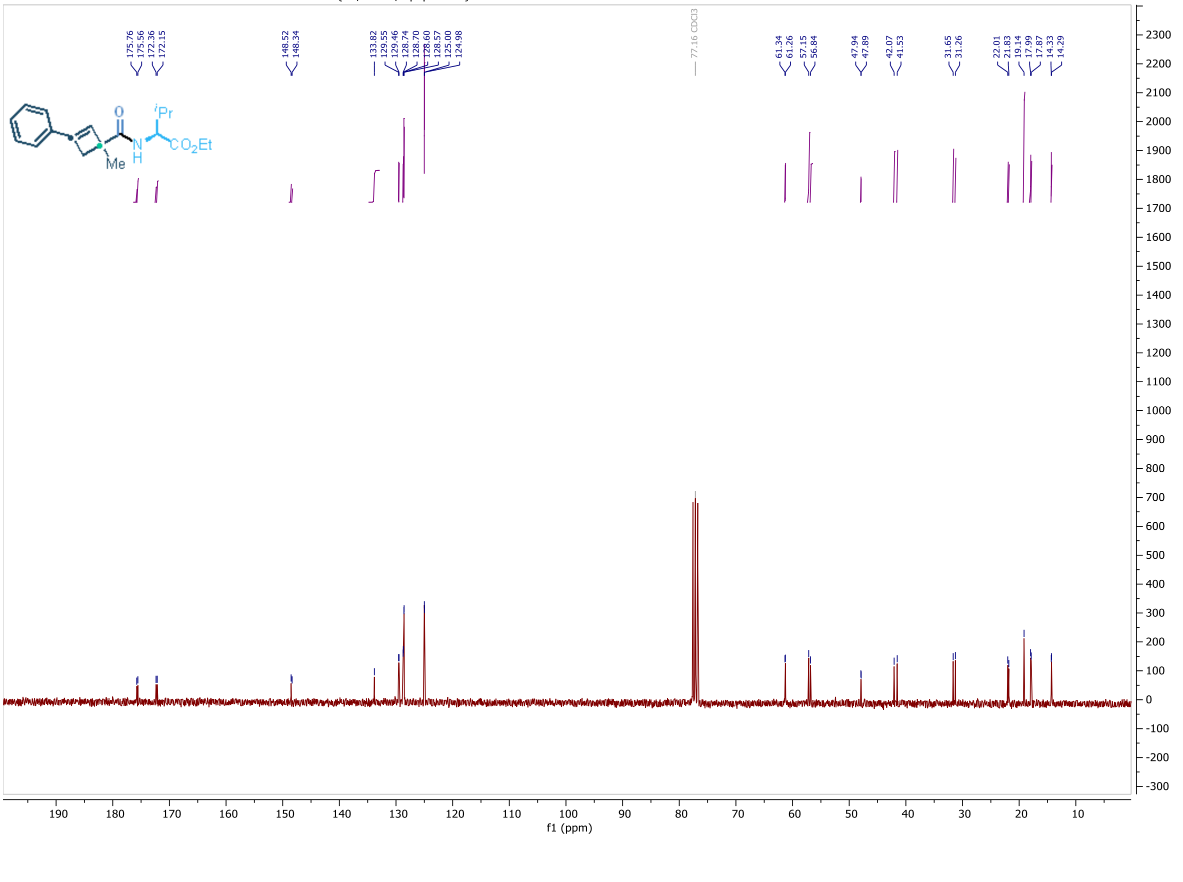

^{1}H NMR spectrum of **4bd** (300 MHz, $CDCl_3$)

260226.320.10.fid — Yu-kun Liu LYK-24-2-95 — Au1H CDCl3 {C:\Bruker\TopSpin3.6.2} 2602 20

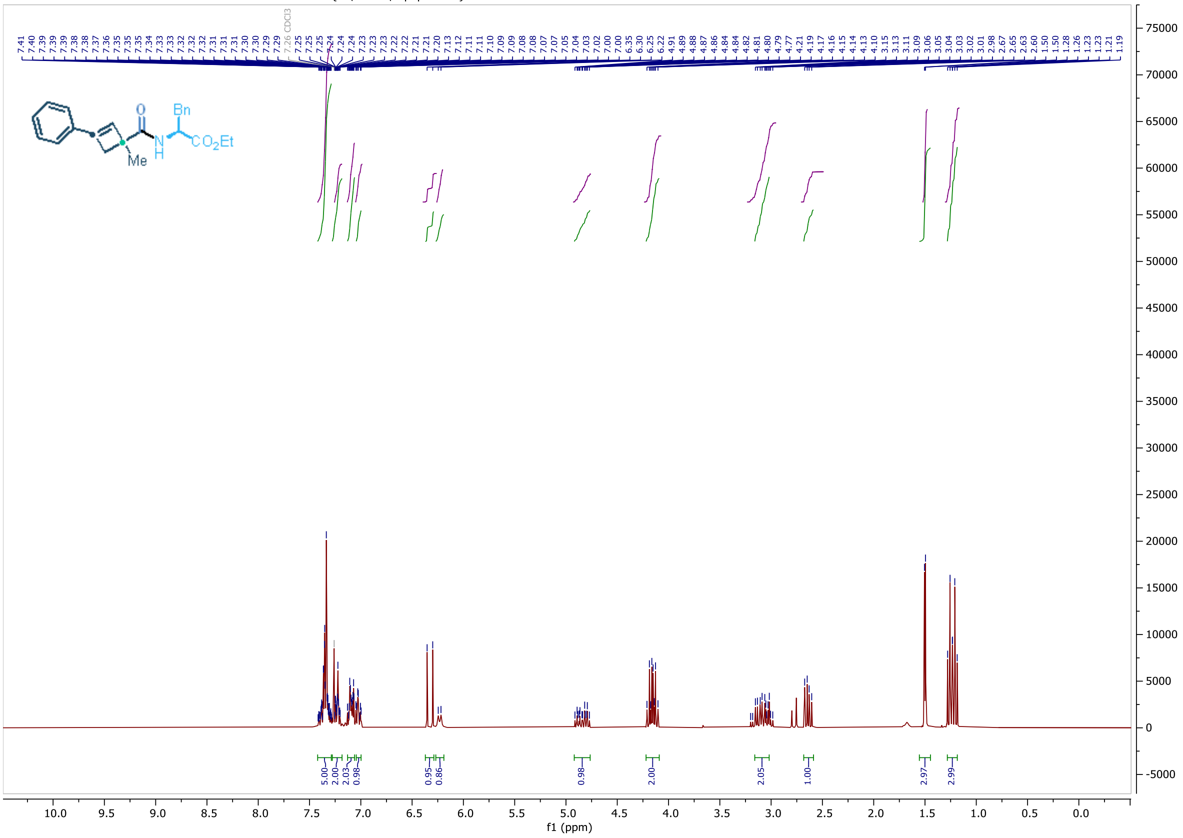

^{13}C NMR spectrum of **4bd** (75 MHz, $CDCl_3$)

260226.320.11.fid — Yu-kun Liu LYK-24-2-95 — Au13C CDCl3 {C:\Bruker\TopSpin3.6.2} 2602 20

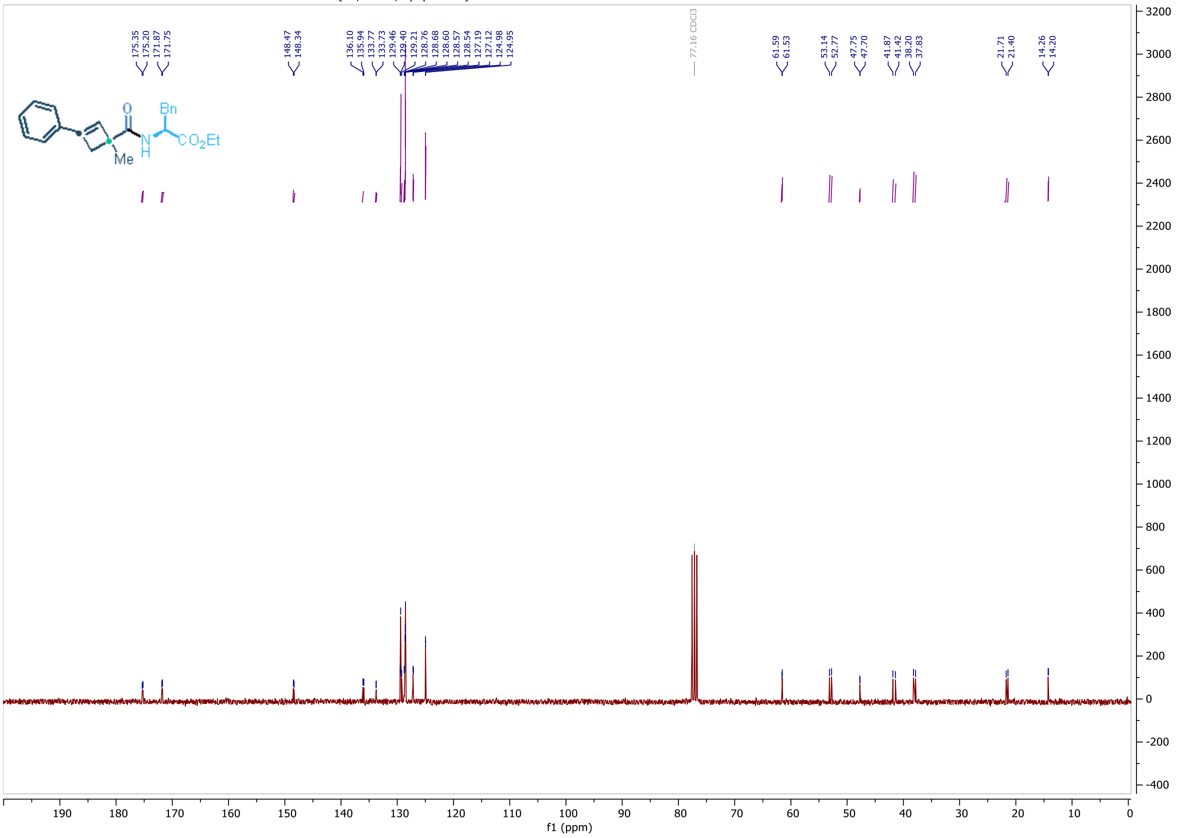

^{1}H NMR spectrum of **4be** (300 MHz, $CDCl_3$)

260226.321.10.fid — Yu-kun Liu LYK-24-2-96 — Au1H CDCl3 {C:\Bruker\TopSpin3.6.2} 2602 21

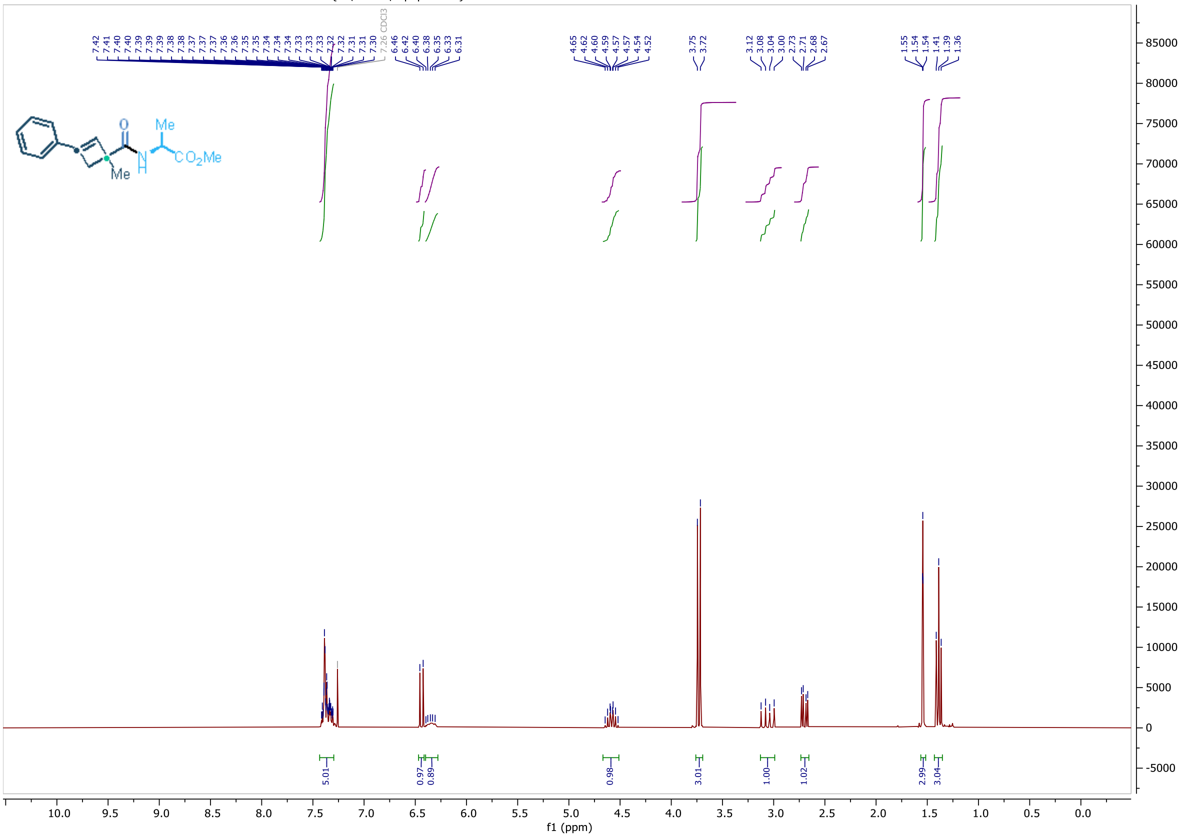

^{13}C NMR spectrum of **4be** (75 MHz, $CDCl_3$)

260226.321.11.fid — Yu-kun Liu LYK-24-2-96 — Au13C CDCl3 {C:\Bruker\TopSpin3.6.2} 2602 21

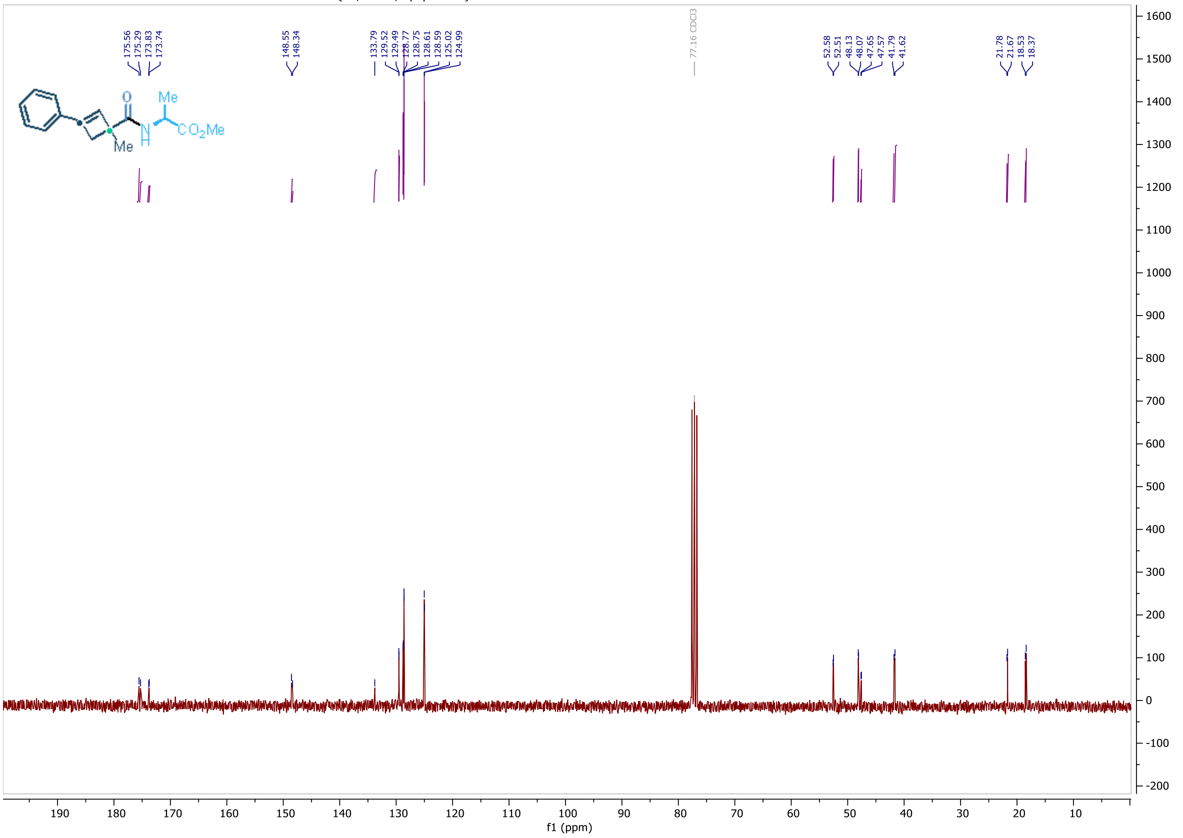

^{1}H NMR spectrum of **4bf** (300 MHz, $CDCl_3$)

260226.322.10.fid — Yu-kun Liu LYK-24-2-97 — Au1H CDCl3 {C:\Bruker\TopSpin3.6.2} 2602 22

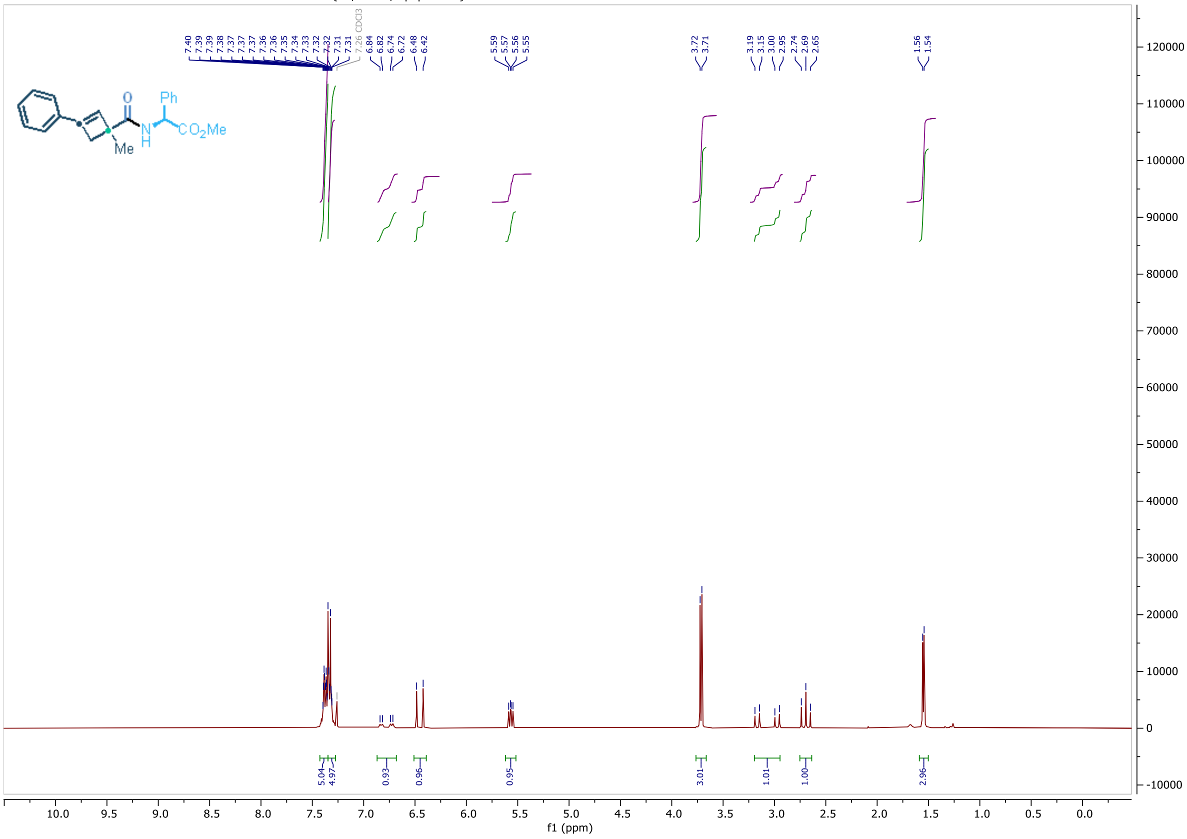

^{13}C NMR spectrum of **4bf** (75 MHz, $CDCl_3$)

260226.322.11.fid — Yu-kun Liu LYK-24-2-97 — Au13C CDCl3 {C:\Bruker\TopSpin3.6.2} 2602 22

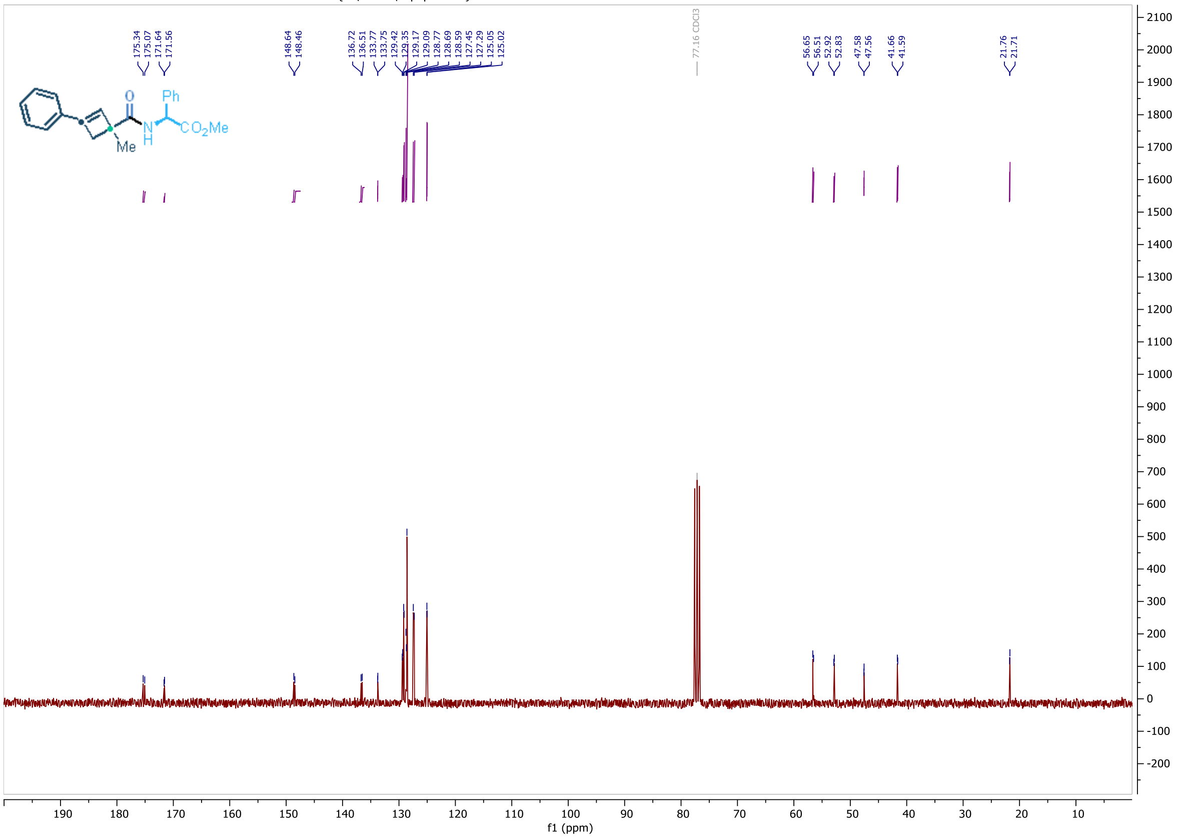

^{1}H NMR spectrum of **5** (300 MHz, $CDCl_3$)

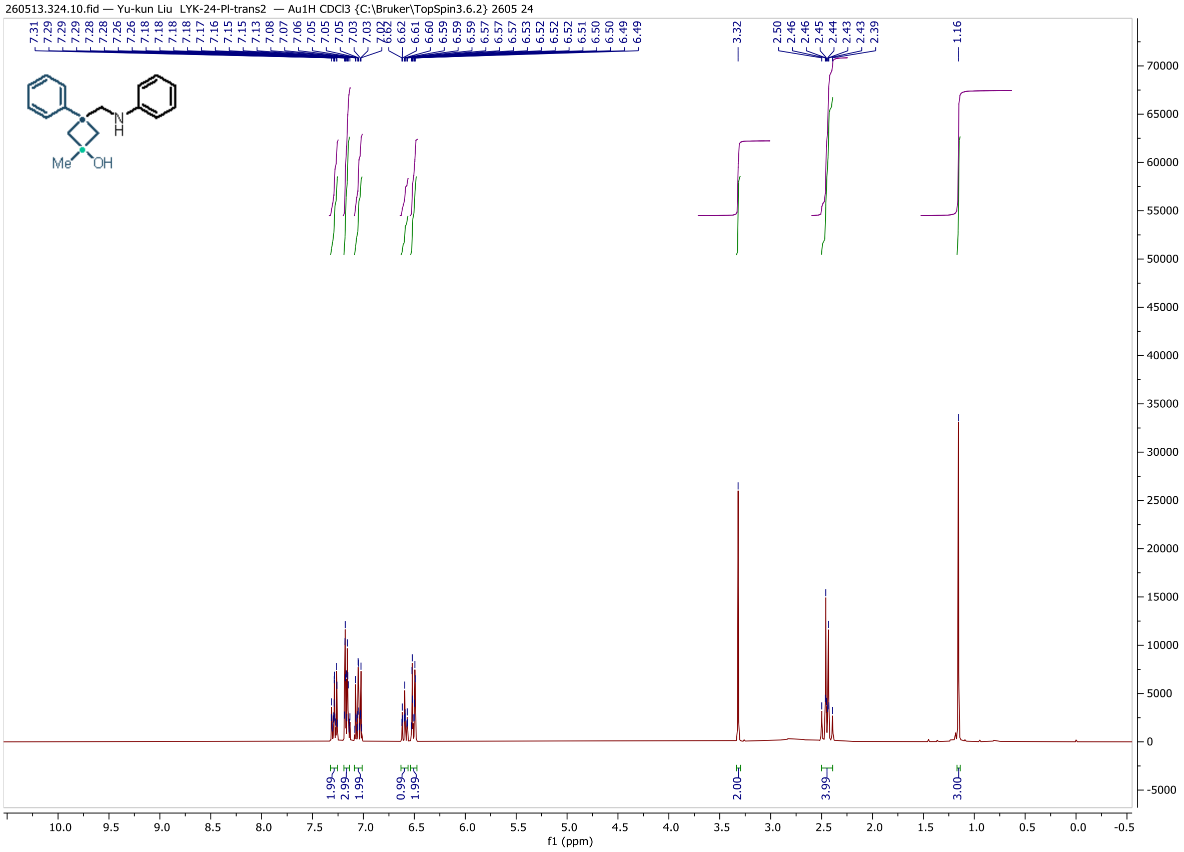

^{13}C NMR spectrum of **5** (75 MHz, $CDCl_3$)

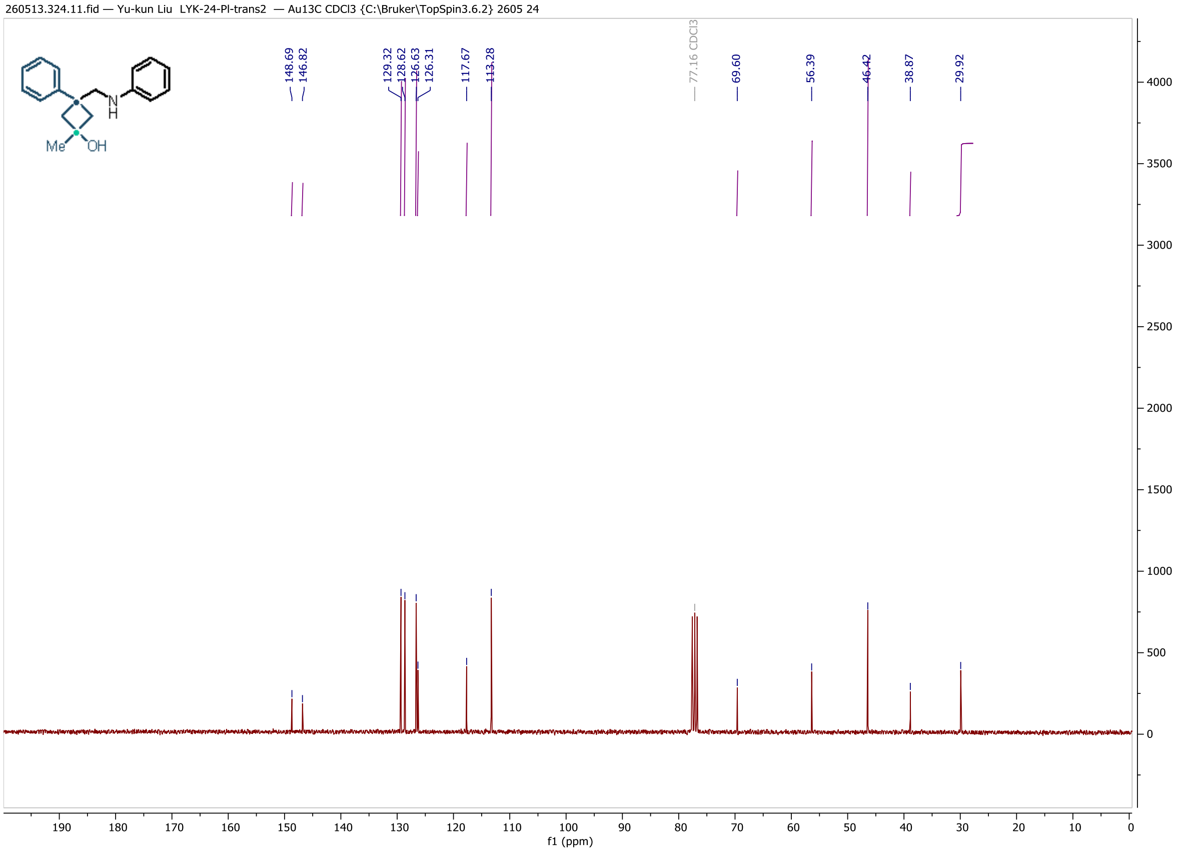

^{1}H NMR spectrum of **6** (300 MHz, $CDCl_3$)

260518.f305.10.fid — Yu-kun Liu LYK-24-IP-TFA — Au1H CDCl3 {C:\Bruker\TopSpin3.6.2} 2605 5

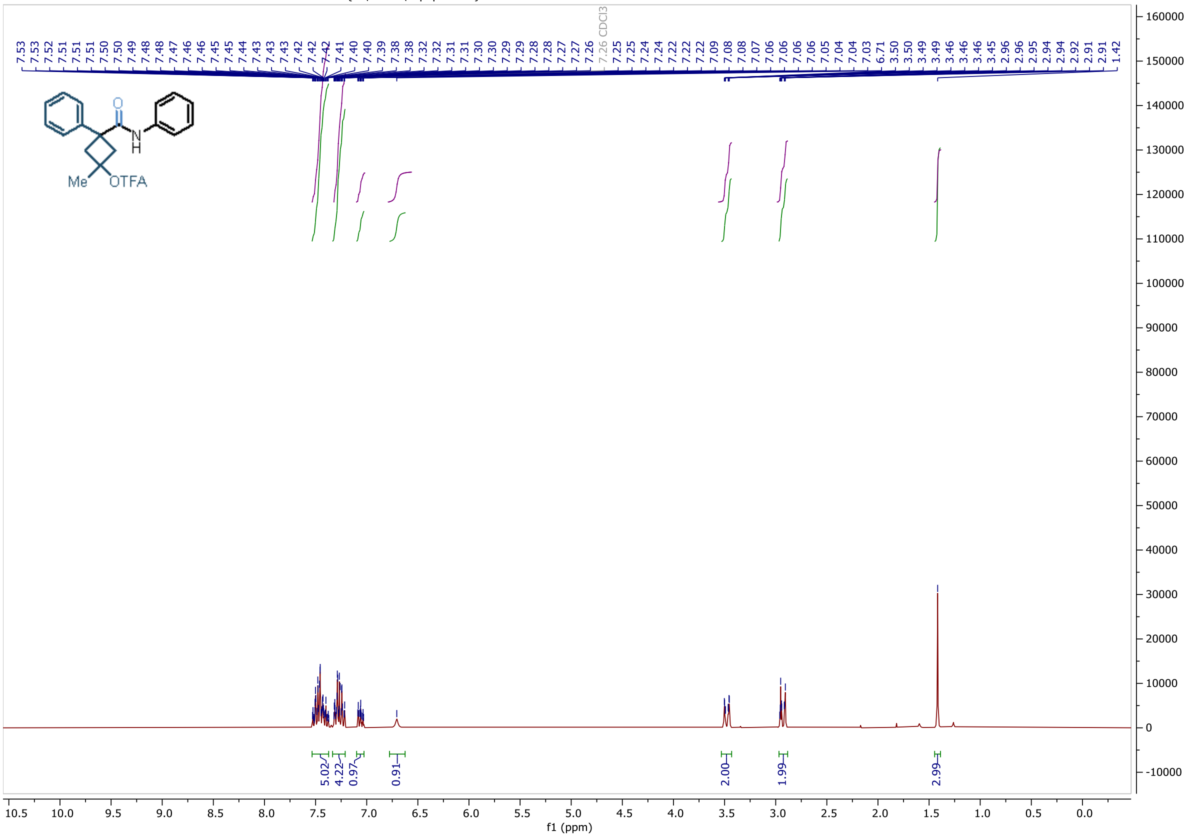

^{13}C NMR spectrum of **6** (75 MHz, $CDCl_3$)

260518.f305.11.fid — Yu-kun Liu LYK-24-IP-TFA — Au13C CDCl3 {C:\Bruker\TopSpin3.6.2} 2605 5

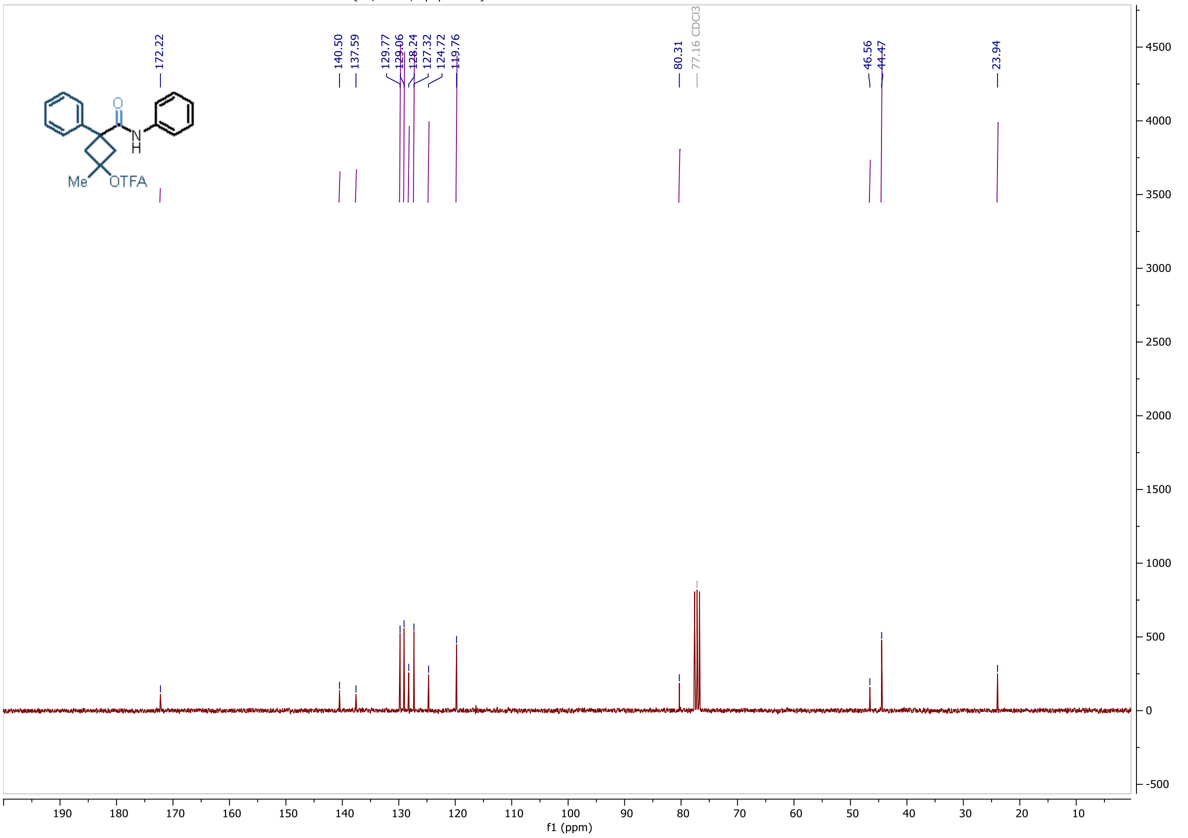

^{19}F NMR spectrum of **6** (282 MHz, $CDCl_3$)

260518.f305.12.fid — Yu-kun Liu LYK-24-IP-TFA — Au19F(H-gek) CDCl3 {C:\Bruker\TopSpin3.6.2} 2605 5

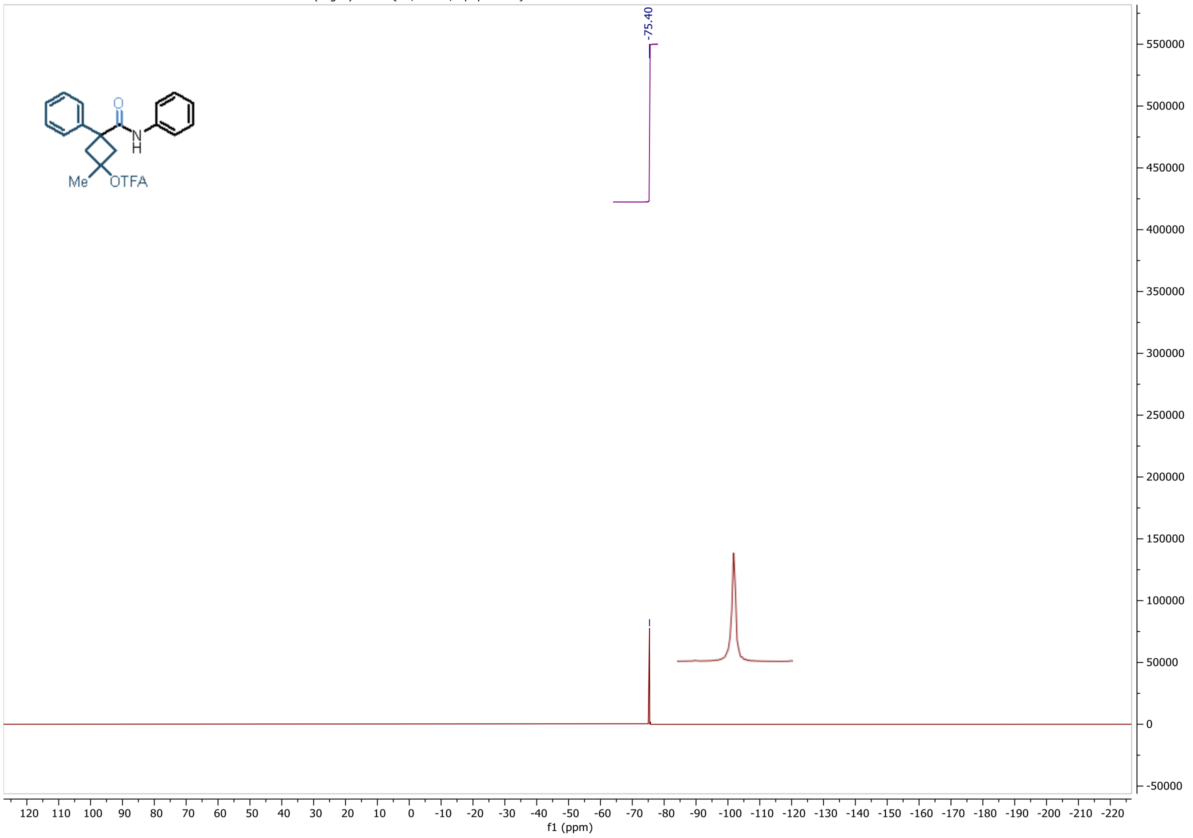

^{1}H NMR spectrum of **7a** (300 MHz, $CDCl_3$)

260507.303.10.fid — Yu-kun Liu LYK-4-1-1P — Au1H CDCl3 {C:\Bruker\TopSpin3.6.2} 2605 3

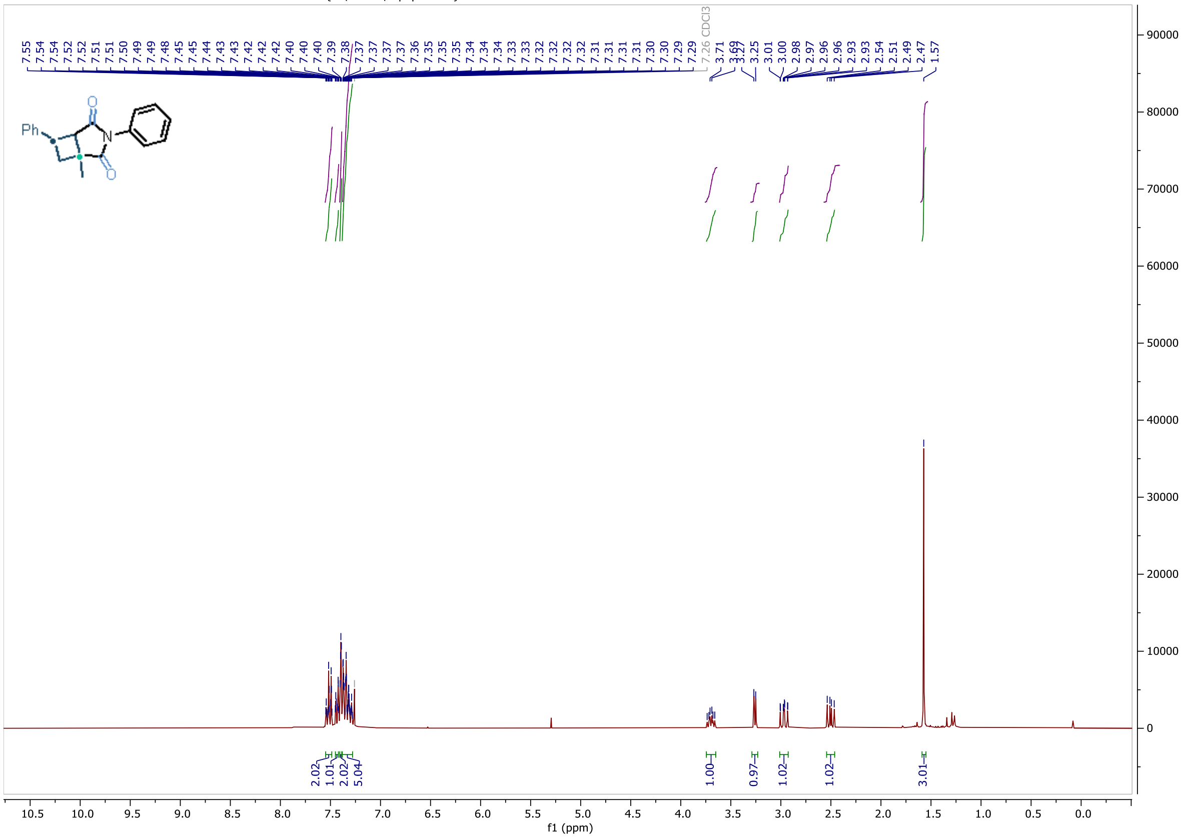

^{13}C NMR spectrum of **7a** (75 MHz, $CDCl_3$)

260507.303.11.fid — Yu-kun Liu LYK-4-1-1P — Au13C CDCl3 {C:\Bruker\TopSpin3.6.2} 2605 3

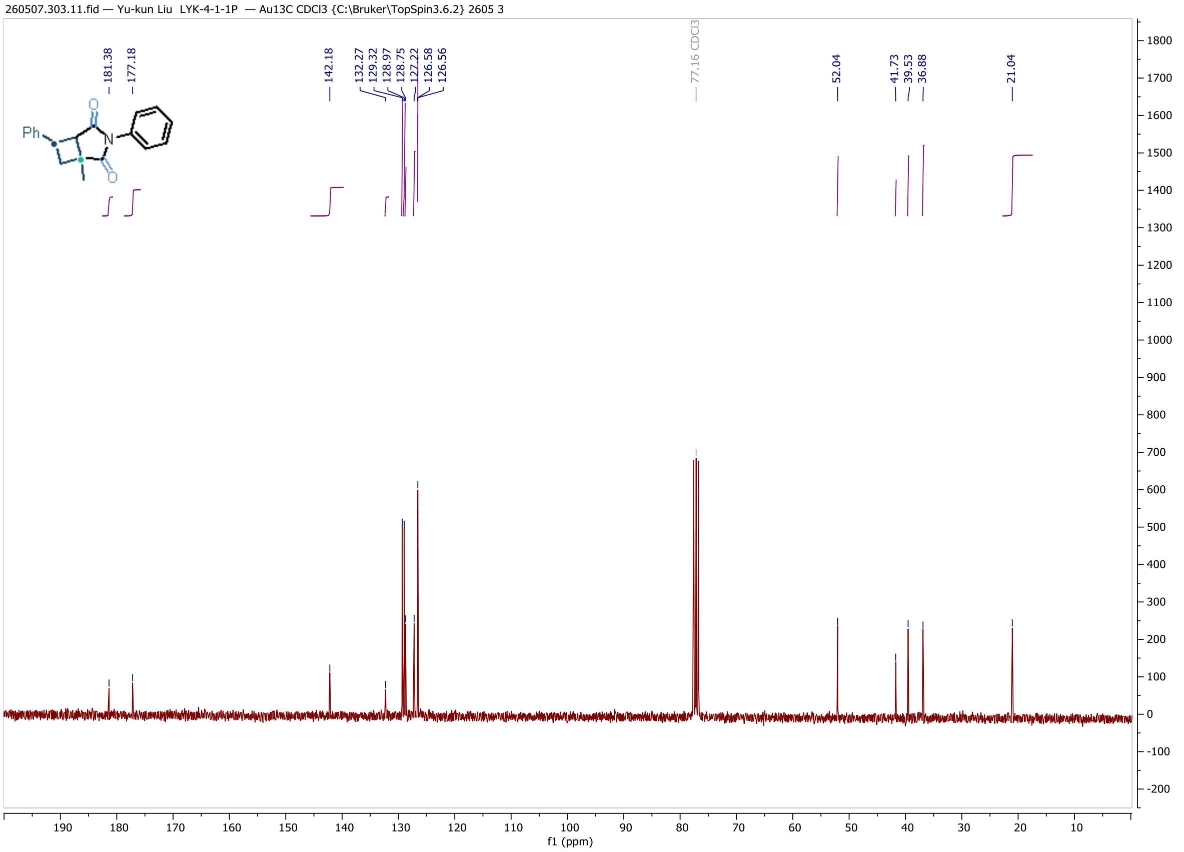

^{1}H NMR spectrum of **7b** (300 MHz, $CDCl_3$)

260505.311.10.fid — Yu-kun Liu LYK-4-2-1P — Au1H CDCl3 {C:\Bruker\TopSpin3.6.2} 2605 11

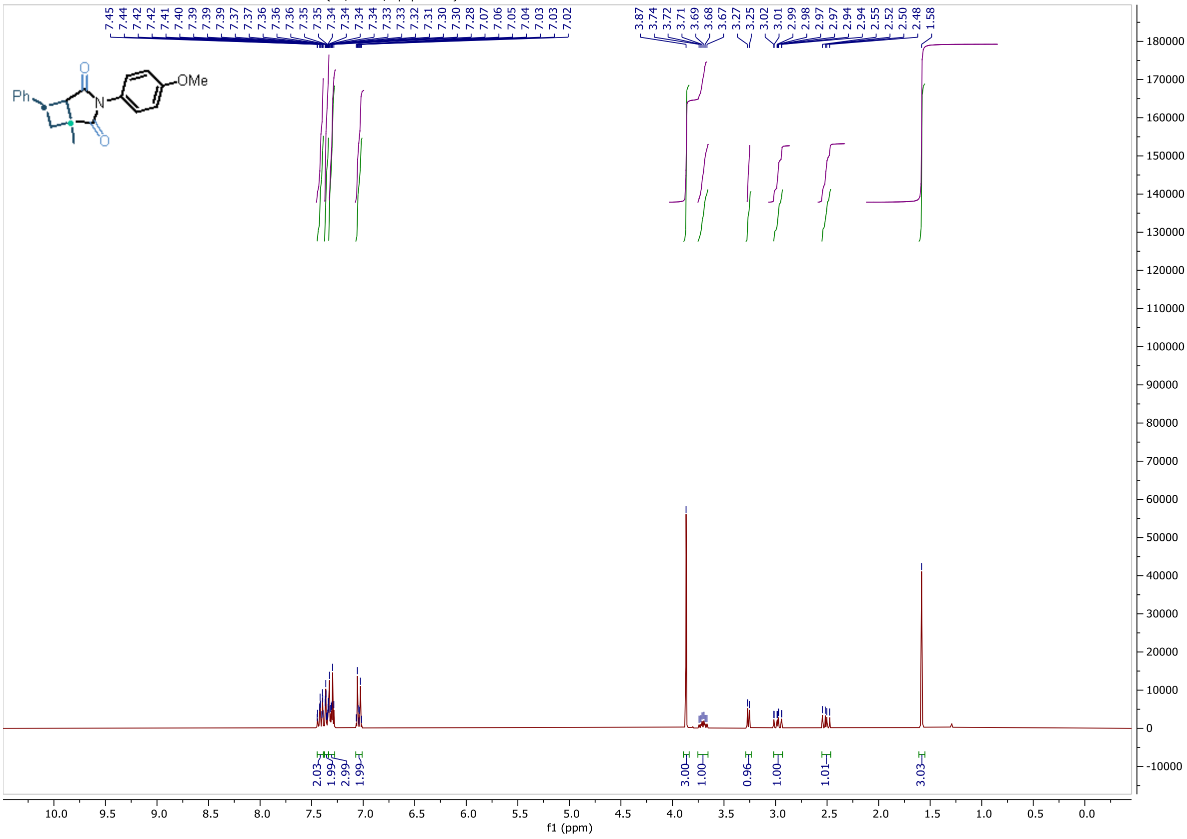

^{13}C NMR spectrum of **7b** (75 MHz, $CDCl_3$)

260505.311.11.fid — Yu-kun Liu LYK-4-2-1P — Au13C CDCl3 {C:\Bruker\TopSpin3.6.2} 2605 11

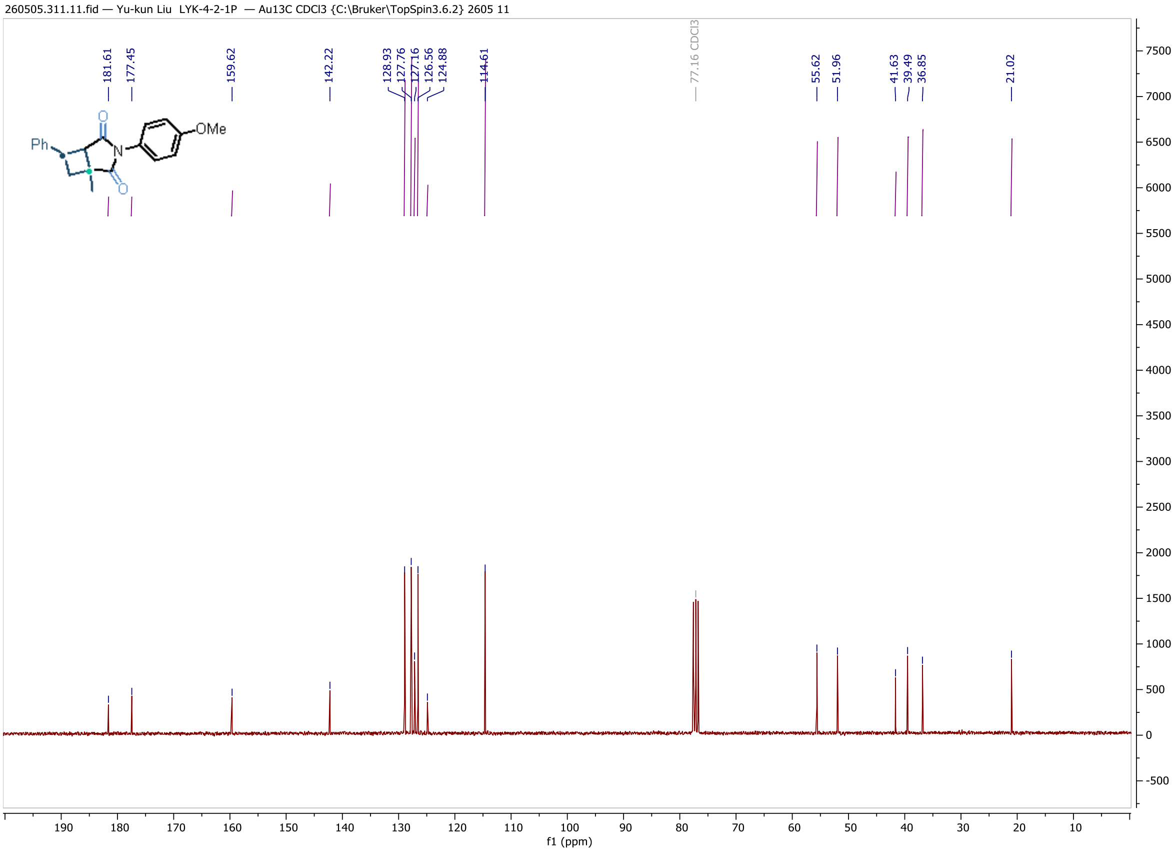

^{1}H NMR spectrum of **7c** (300 MHz, $CDCl_3$)

260506.302.10.fid — Yu-kun Liu LYK-4-3-1P — Au1H CDCl3 {C:\Bruker\TopSpin3.6.2} 2605 2

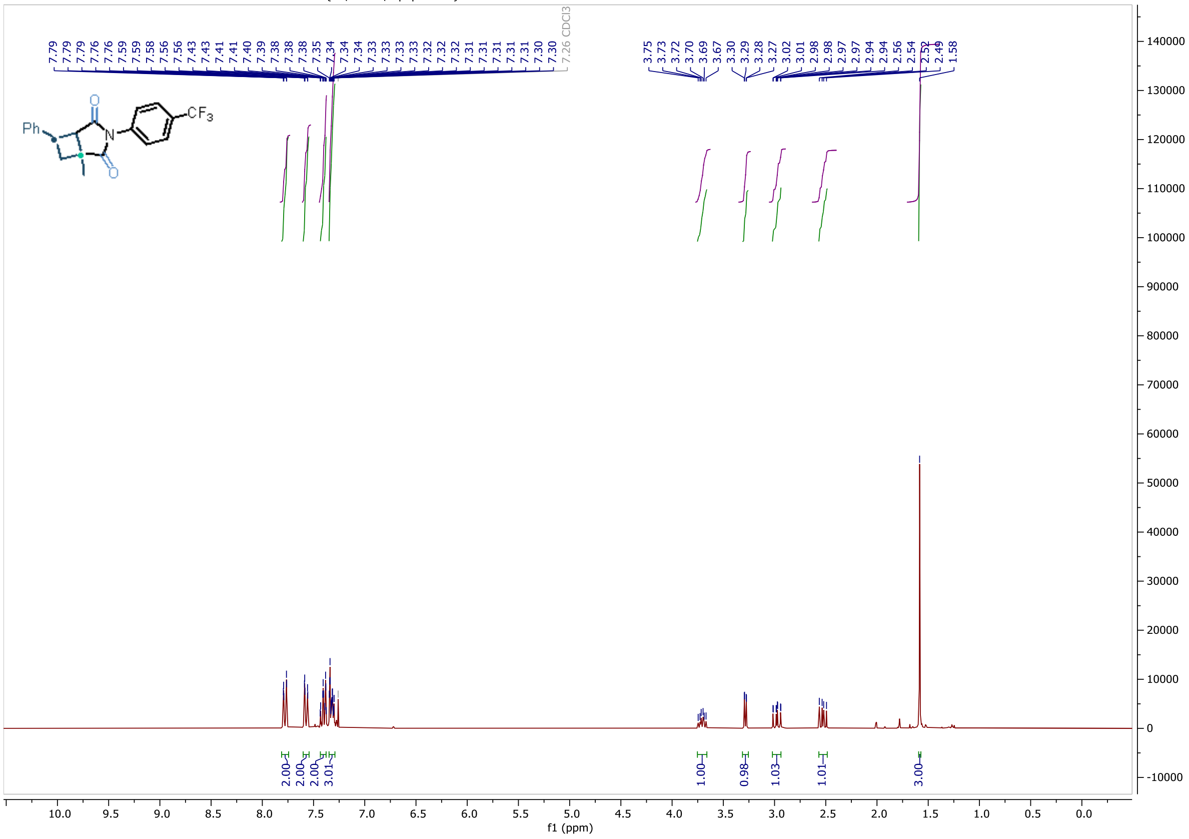

^{13}C NMR spectrum of **7c** (75 MHz, $CDCl_3$)

260506.302.11.fid — Yu-kun Liu LYK-4-3-1P — Au13C CDCl3 {C:\Bruker\TopSpin3.6.2} 2605 2

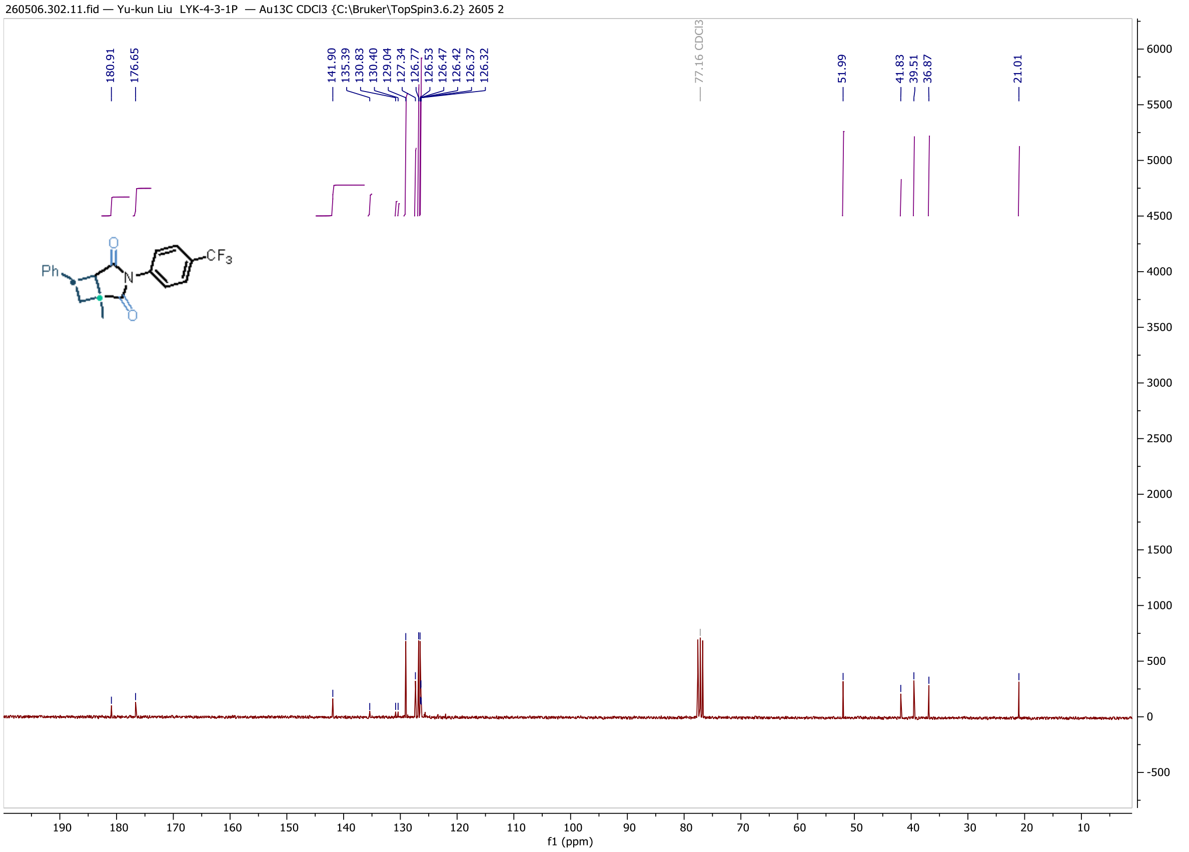

^{19}F NMR spectrum of **7c** (282 MHz, $CDCl_3$)

260506.302.12.fid — Yu-kun Liu LYK-4-3-1P — Au19F CDCl3 {C:\Bruker\TopSpin3.6.2} 2605 2

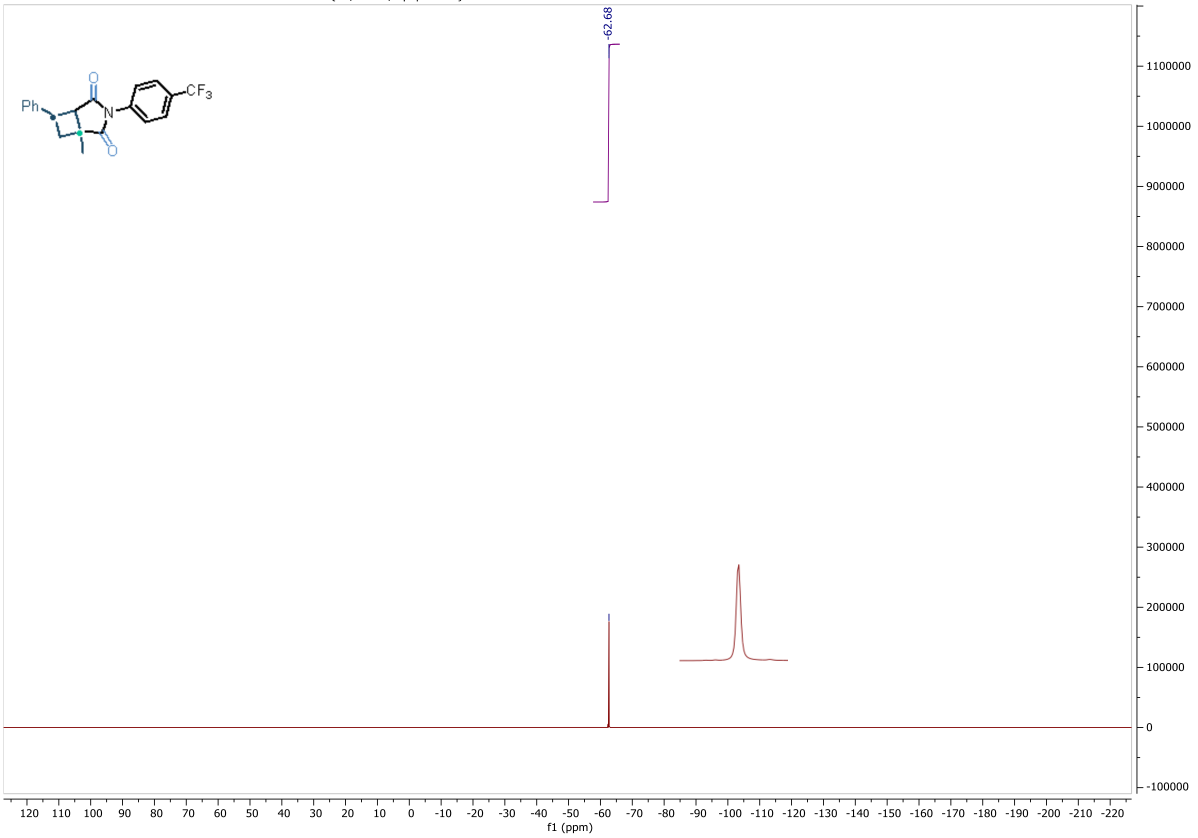

^{1}H NMR spectrum of **7d** (300 MHz, $CDCl_3$)

260508.309.10.fid — Yu-kun Liu LYK-4-5-1P — Au1H CDCl3 {C:\Bruker\TopSpin3.6.2} 2605 9

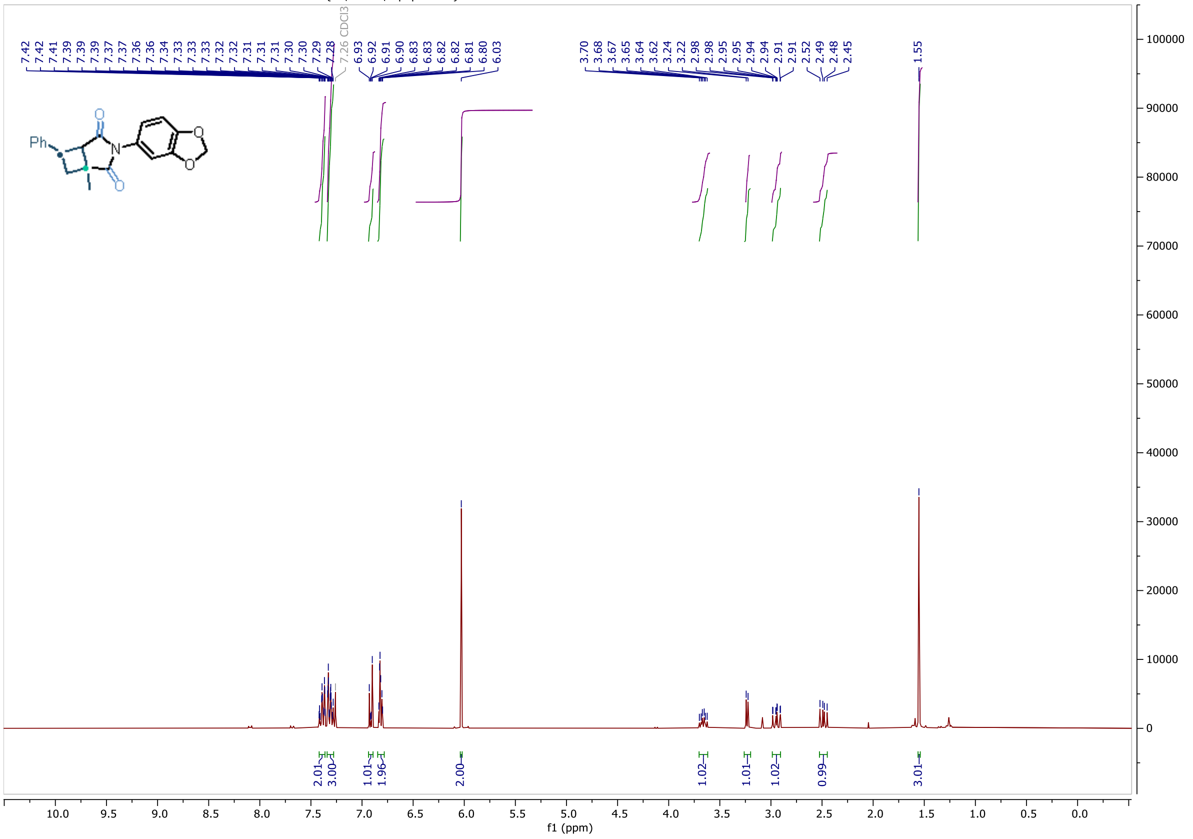

^{13}C NMR spectrum of **7d** (75 MHz, $CDCl_3$)

260508.309.11.fid — Yu-kun Liu LYK-4-5-1P — Au13C CDCl3 {C:\Bruker\TopSpin3.6.2} 2605 9

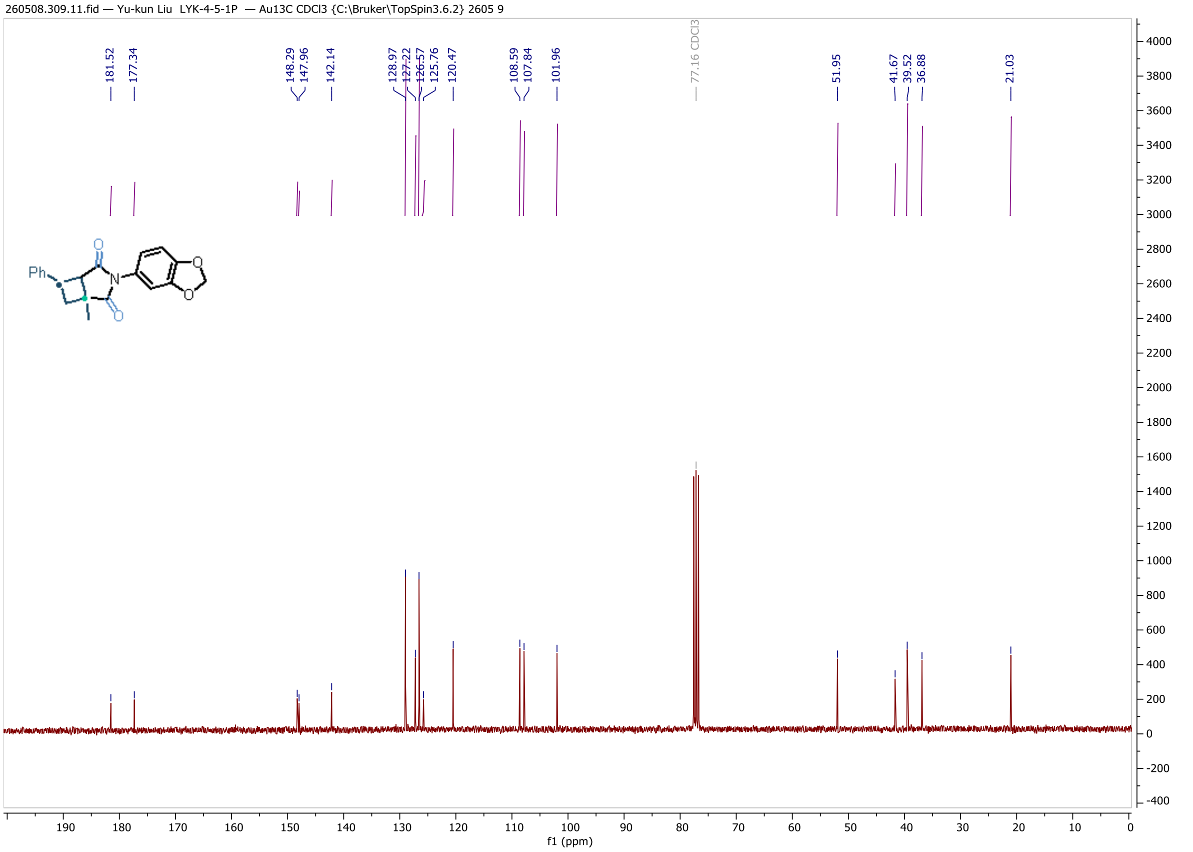

^{1}H NMR spectrum of **8a** (300 MHz, $CDCl_3$)

260505.310.10.fid — Yu-kun Liu LYK-4-1-2P — Au1H CDCl3 {C:\Bruker\TopSpin3.6.2} 2605 10

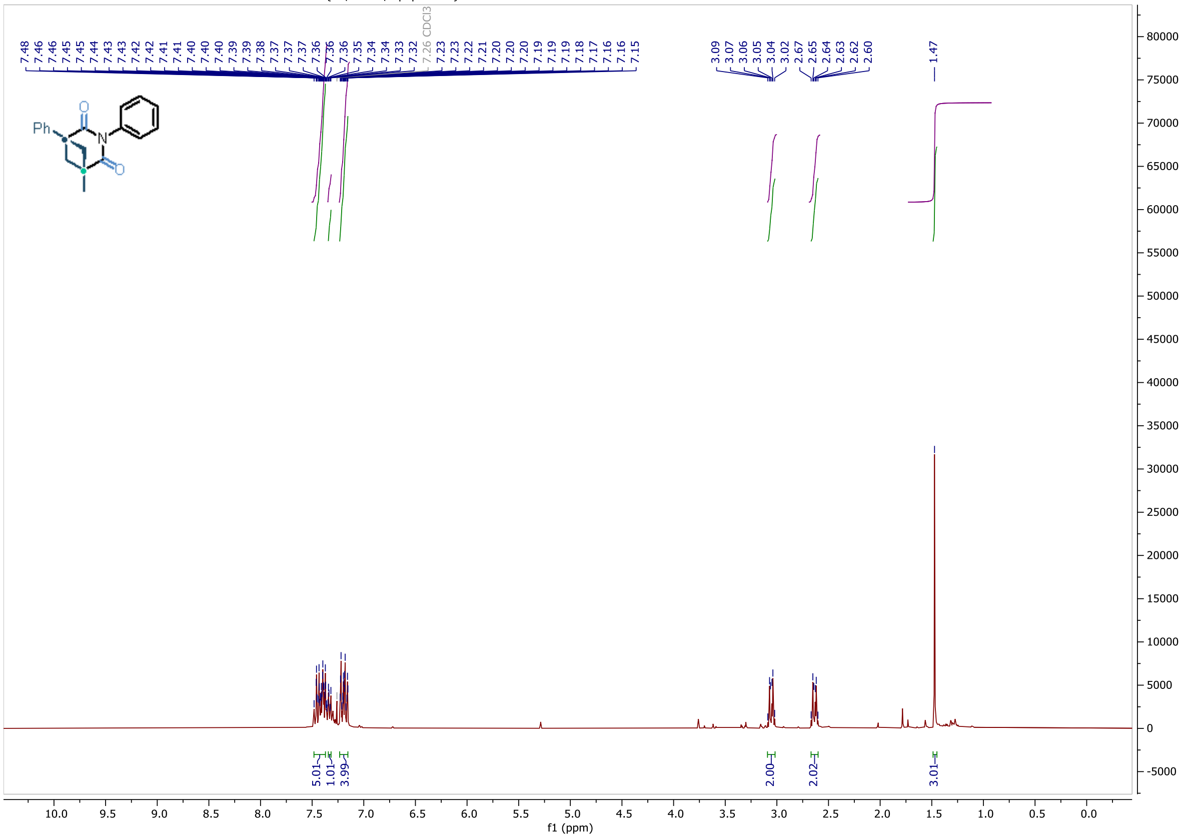

^{13}C NMR spectrum of **8a** (75 MHz, $CDCl_3$)

260505.310.11.fid — Yu-kun Liu LYK-4-1-2P — Au13C CDCl3 {C:\Bruker\TopSpin3.6.2} 2605 10

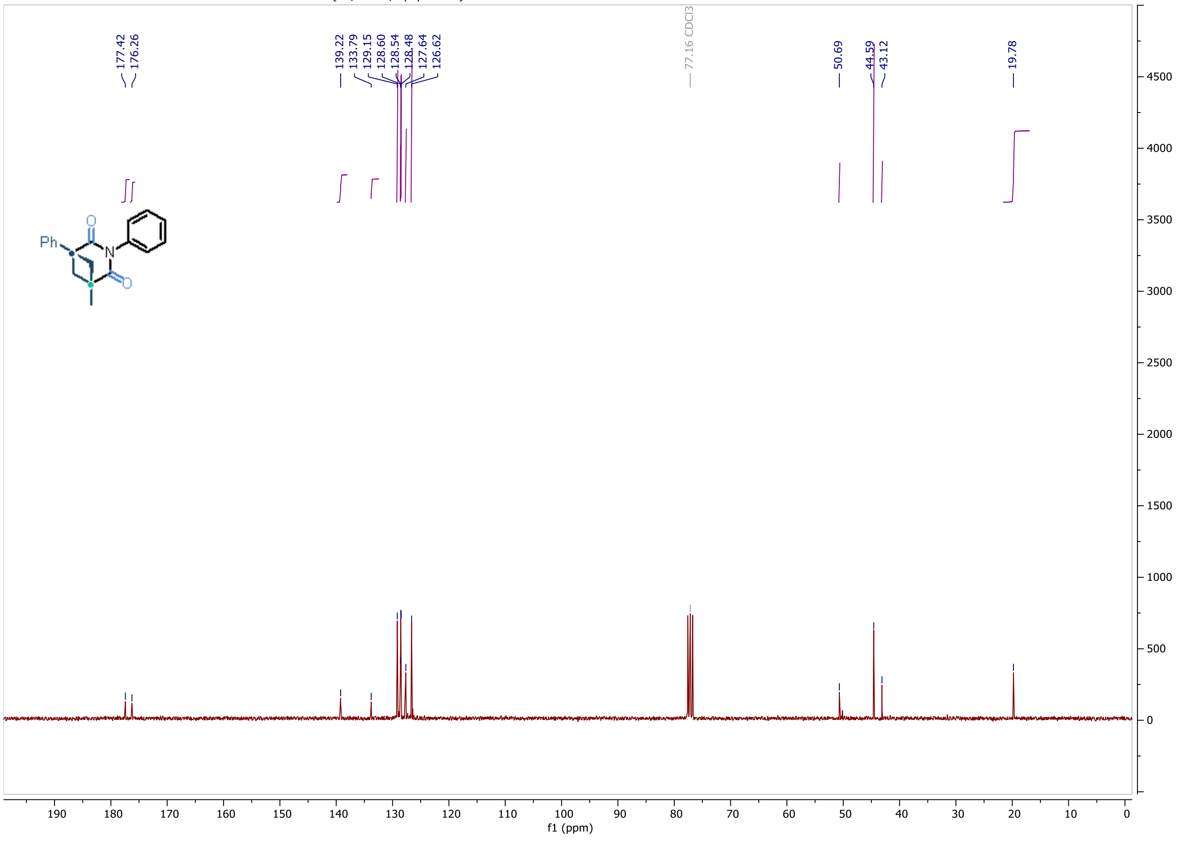

^{1}H NMR spectrum of **8b** (300 MHz, $CDCl_3$)

260505.312.10.fid — Yu-kun Liu LYK-4-2-2P — Au1H CDCl3 {C:\Bruker\TopSpin3.6.2} 2605 12

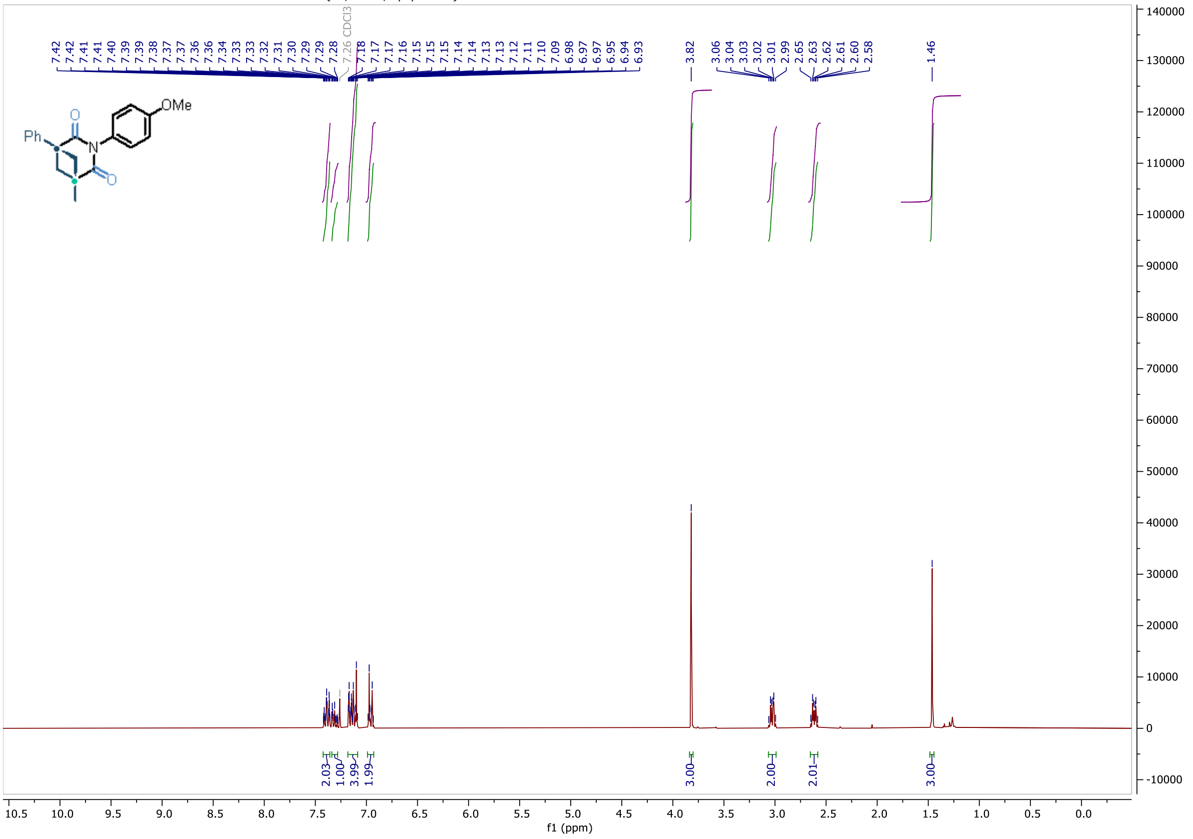

^{13}C NMR spectrum of **8b** (75 MHz, $CDCl_3$)

260505.312.11.fid — Yu-kun Liu LYK-4-2-2P — Au13C CDCl3 {C:\Bruker\TopSpin3.6.2} 2605 12

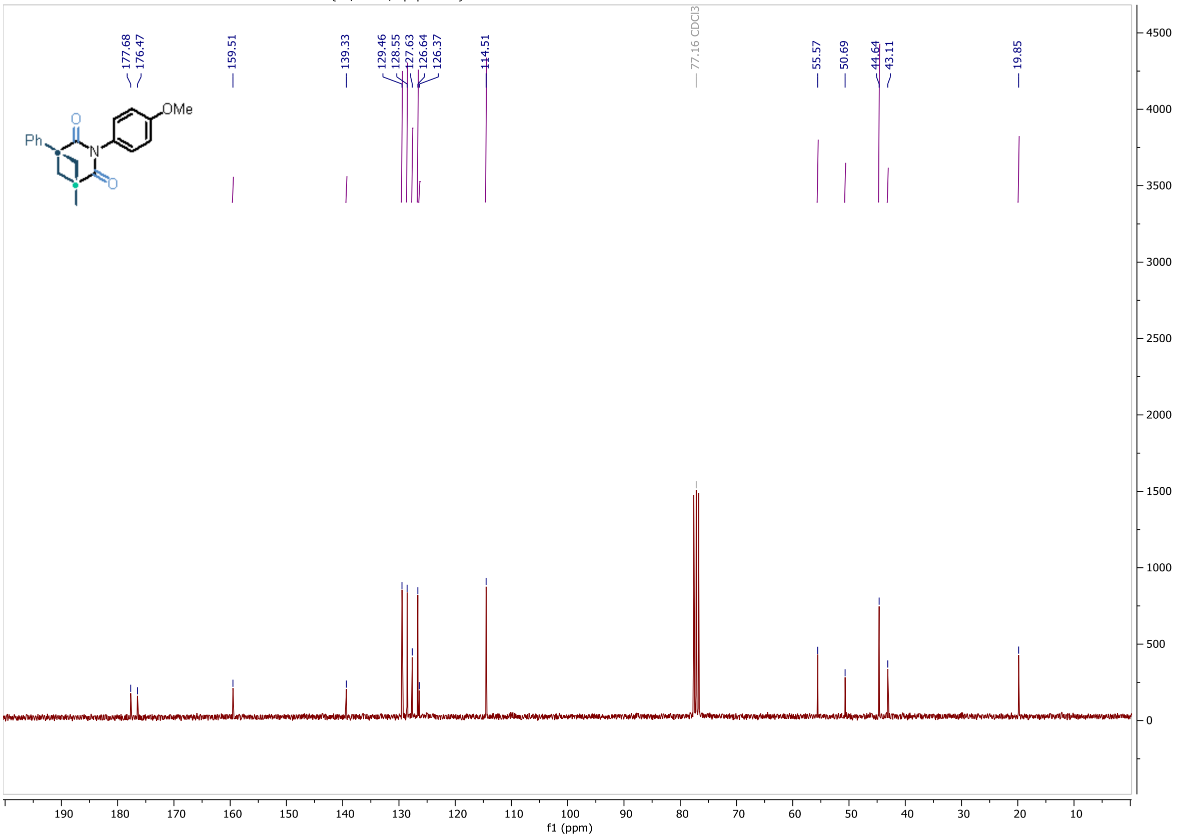

^{1}H NMR spectrum of **8c** (300 MHz, $CDCl_3$)

260505.313.10.fid — Yu-kun Liu LYK-4-3-2P — Au1H CDCl3 {C:\Bruker\TopSpin3.6.2} 2605 13

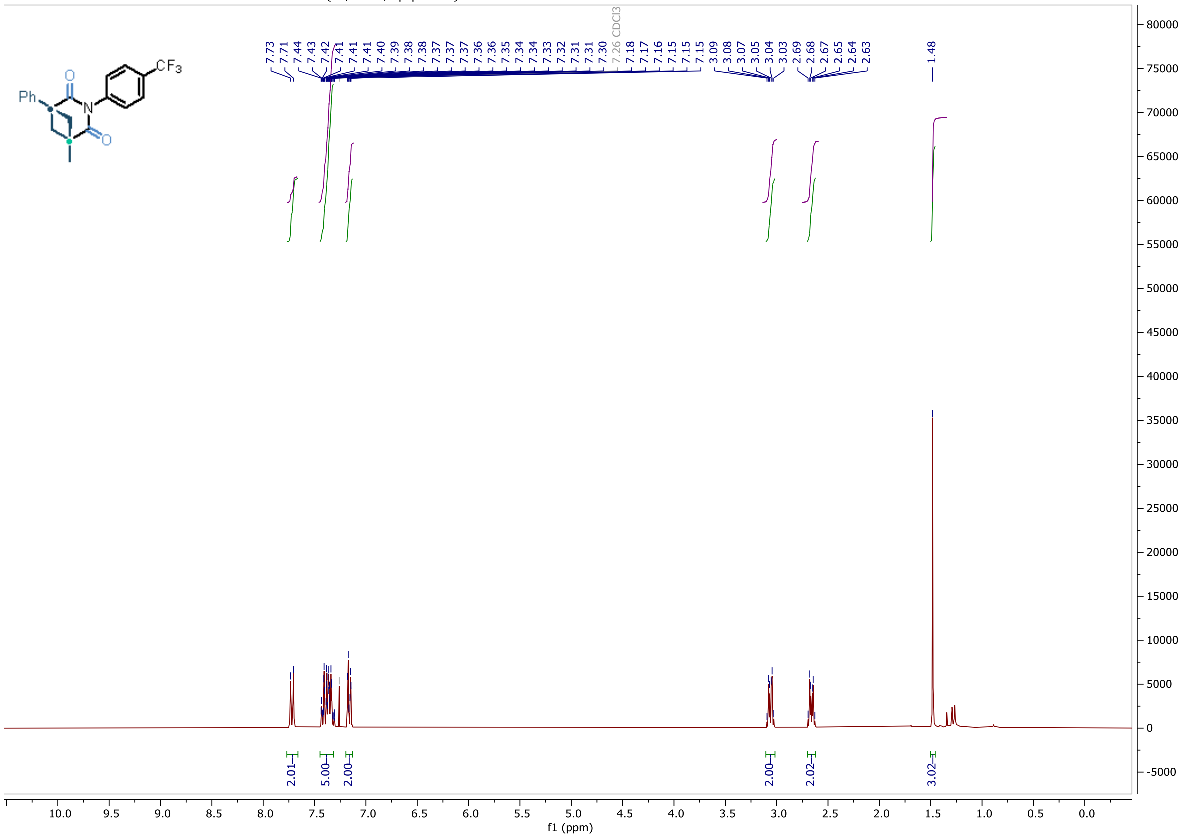

^{13}C NMR spectrum of **8c** (75 MHz, $CDCl_3$)

260505.313.11.fid — Yu-kun Liu LYK-4-3-2P — Au13C CDCl3 {C:\Bruker\TopSpin3.6.2} 2605 13

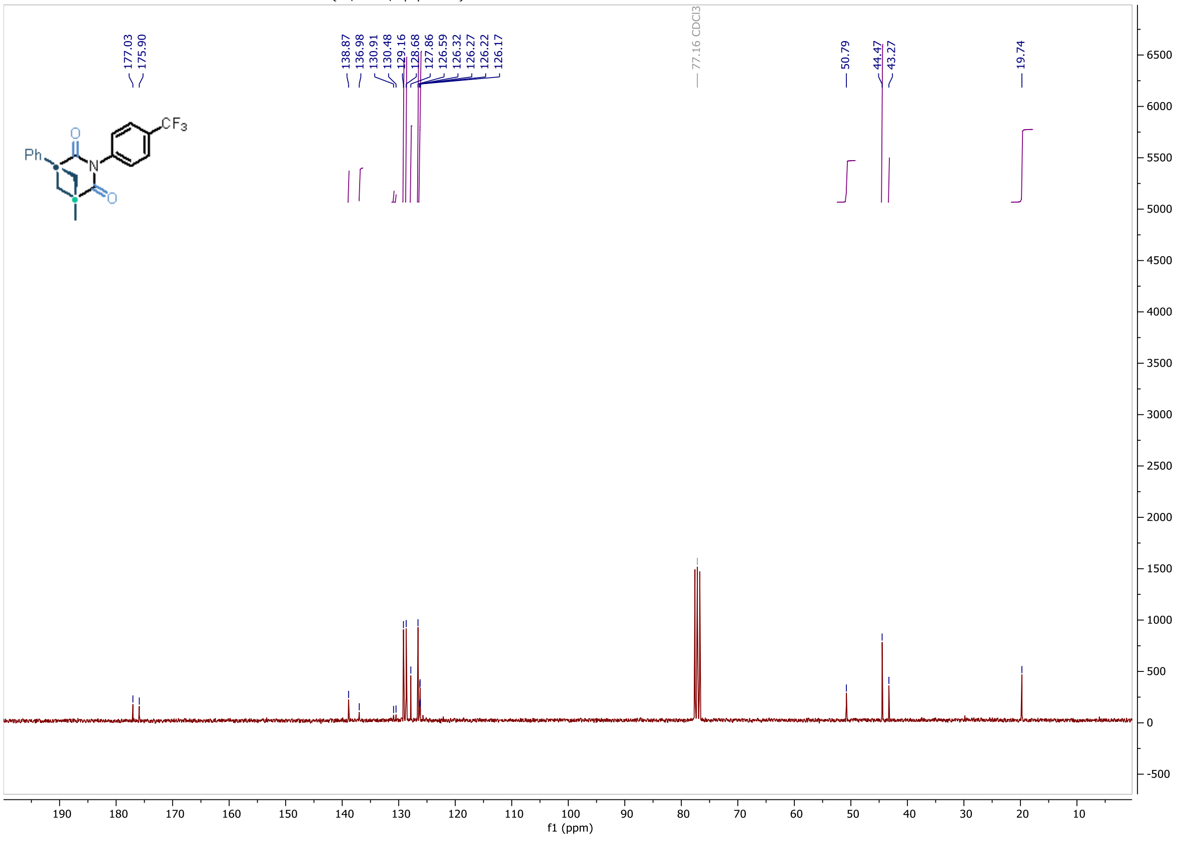

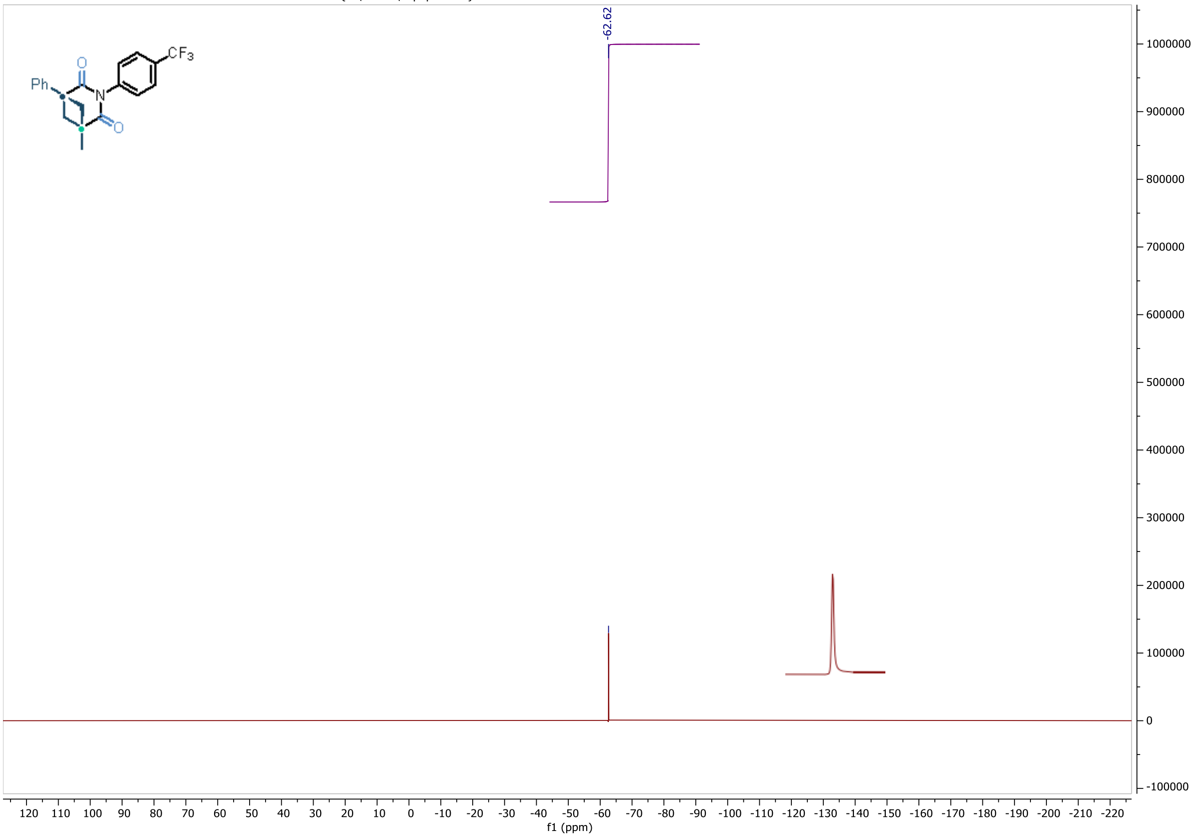
Ph
O
N
O
CF3
-62.62
1000000
900000
800000
700000
600000
500000
400000
300000
200000
100000
0
-100000
120 110 100 90 80 70 60 50 40 30 20 10 0 -10 -20 -30 -40 -50 -60 -70 -80 -90 -100 -110 -120 -130 -140 -150 -160 -170 -180 -190 -200 -210 -220
f1 (ppm)

^{1}H NMR spectrum of **8d** (300 MHz, $CDCl_3$)

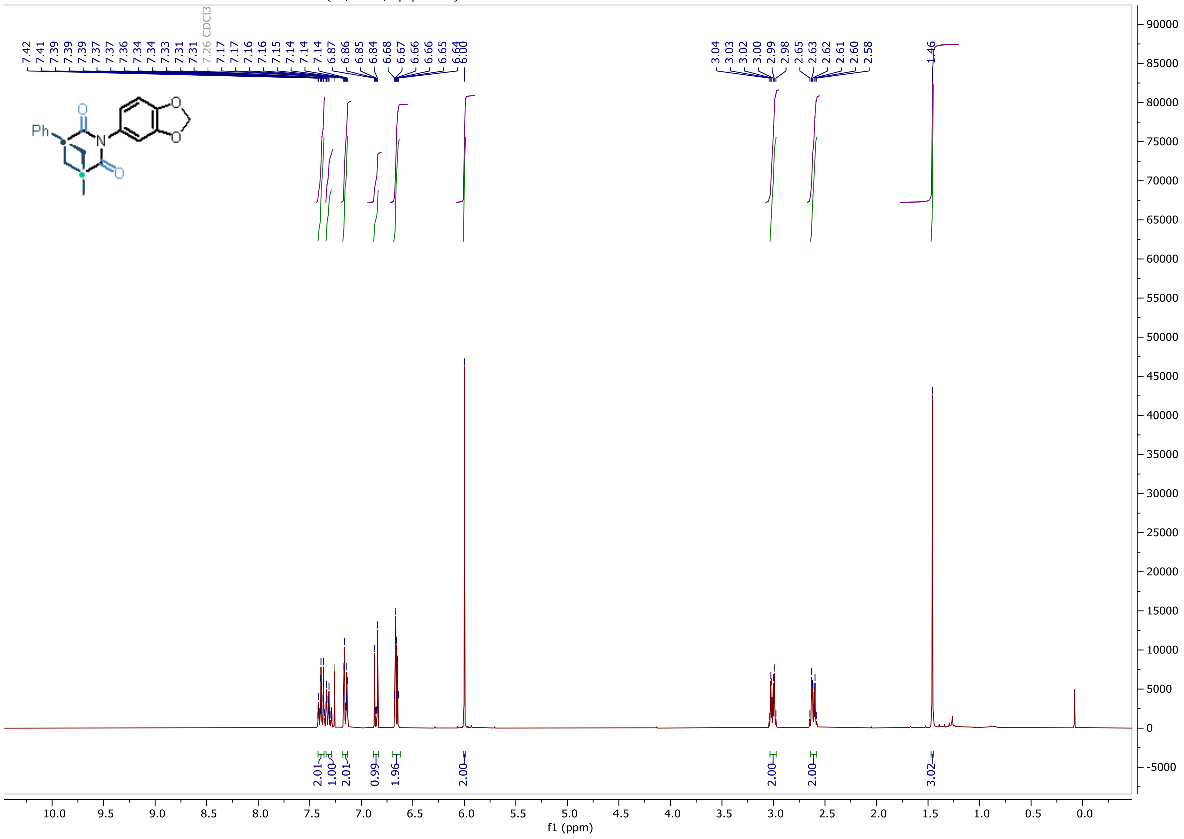

^{13}C NMR spectrum of **8d** (75 MHz, $CDCl_3$)

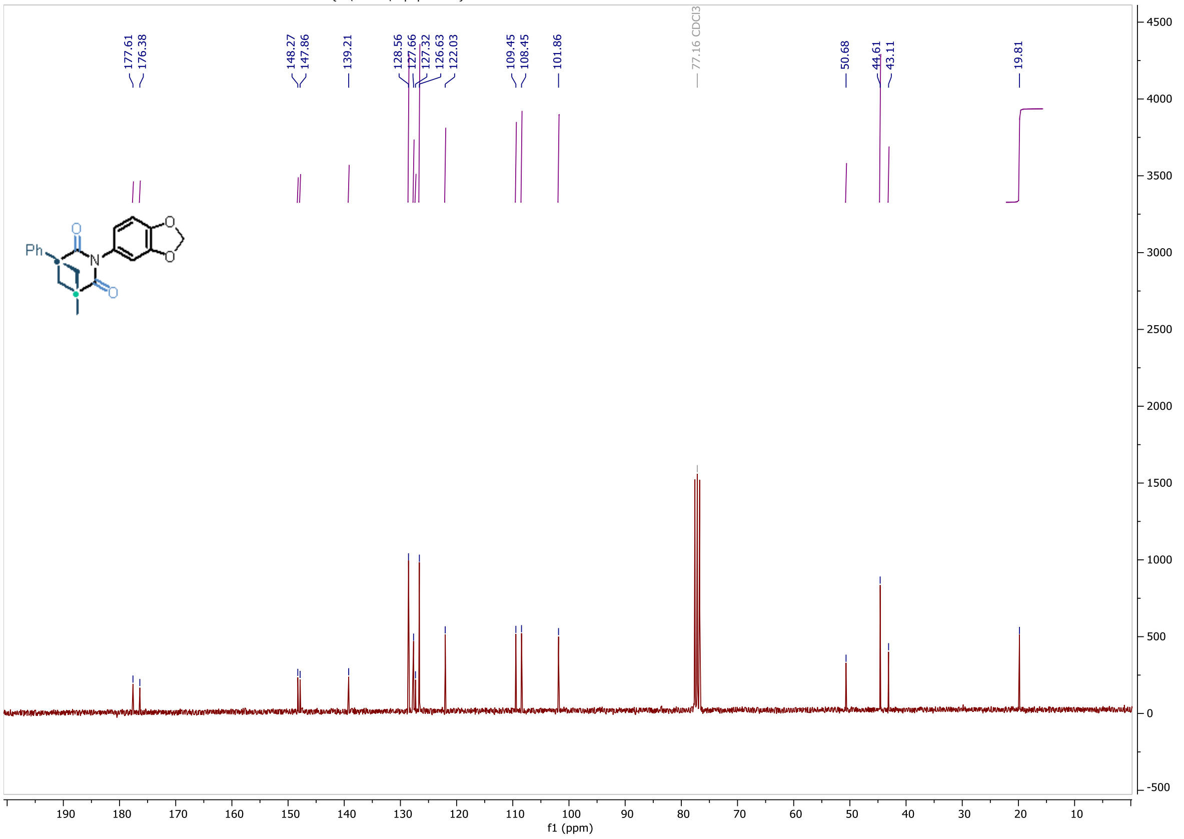

^{1}H NMR spectrum of **9** (300 MHz, $CDCl_3$)

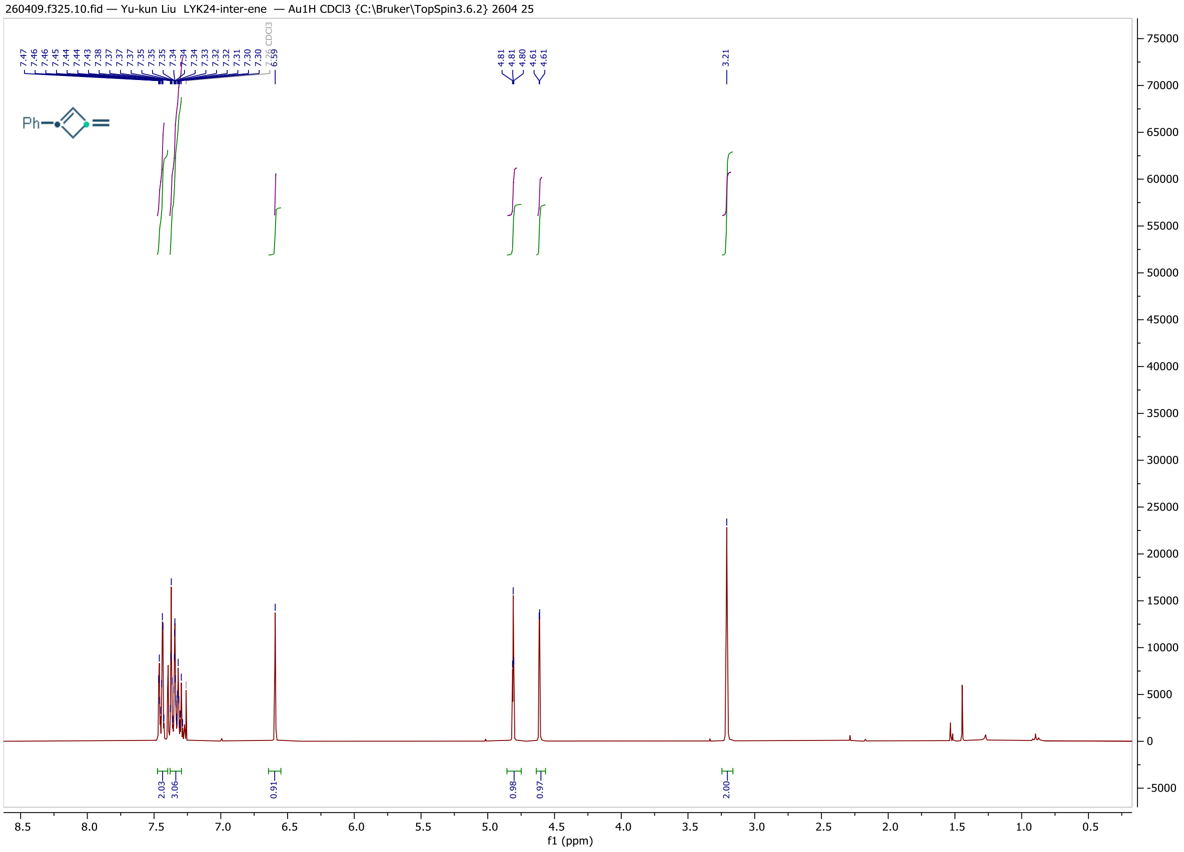

^{13}C NMR spectrum of **9** (75 MHz, $CDCl_3$)

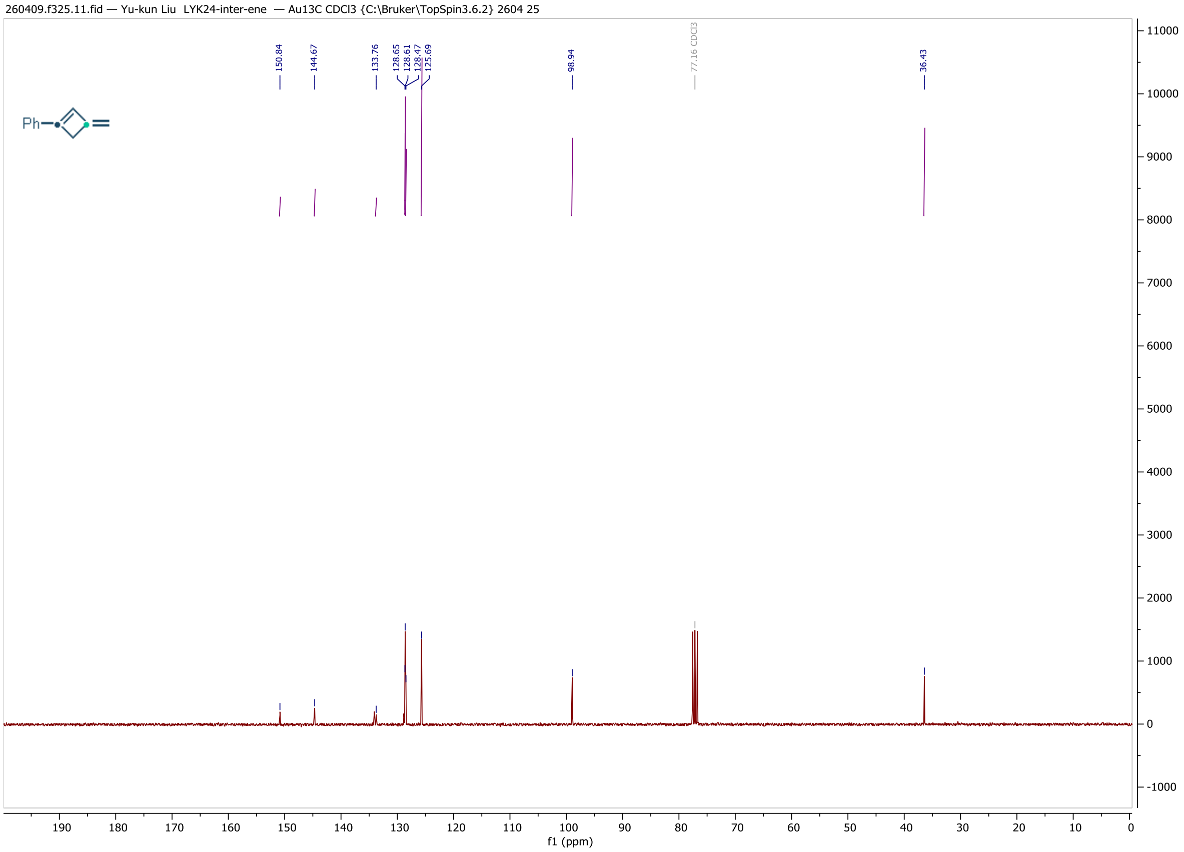

^{1}H NMR spectrum of **10** (300 MHz, $CDCl_3$)

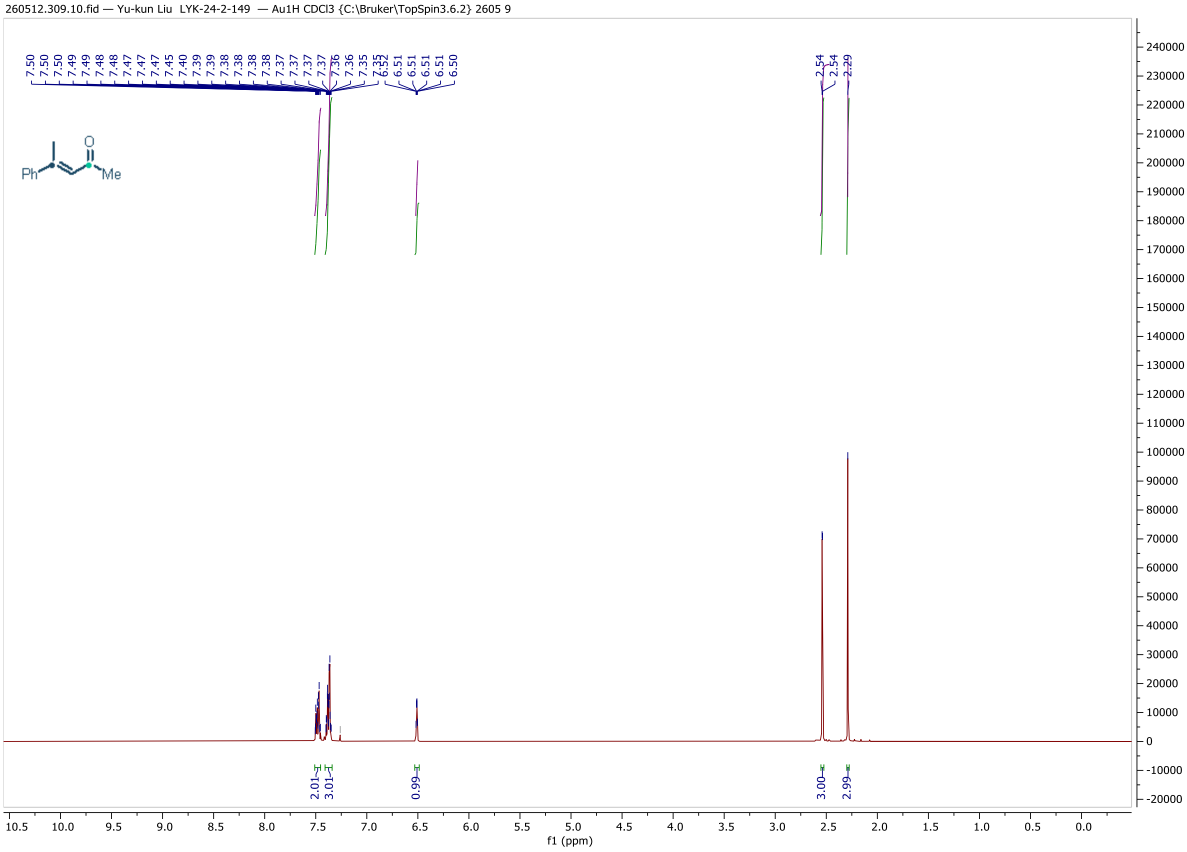

^{13}C NMR spectrum of **10** (75 MHz, $CDCl_3$)

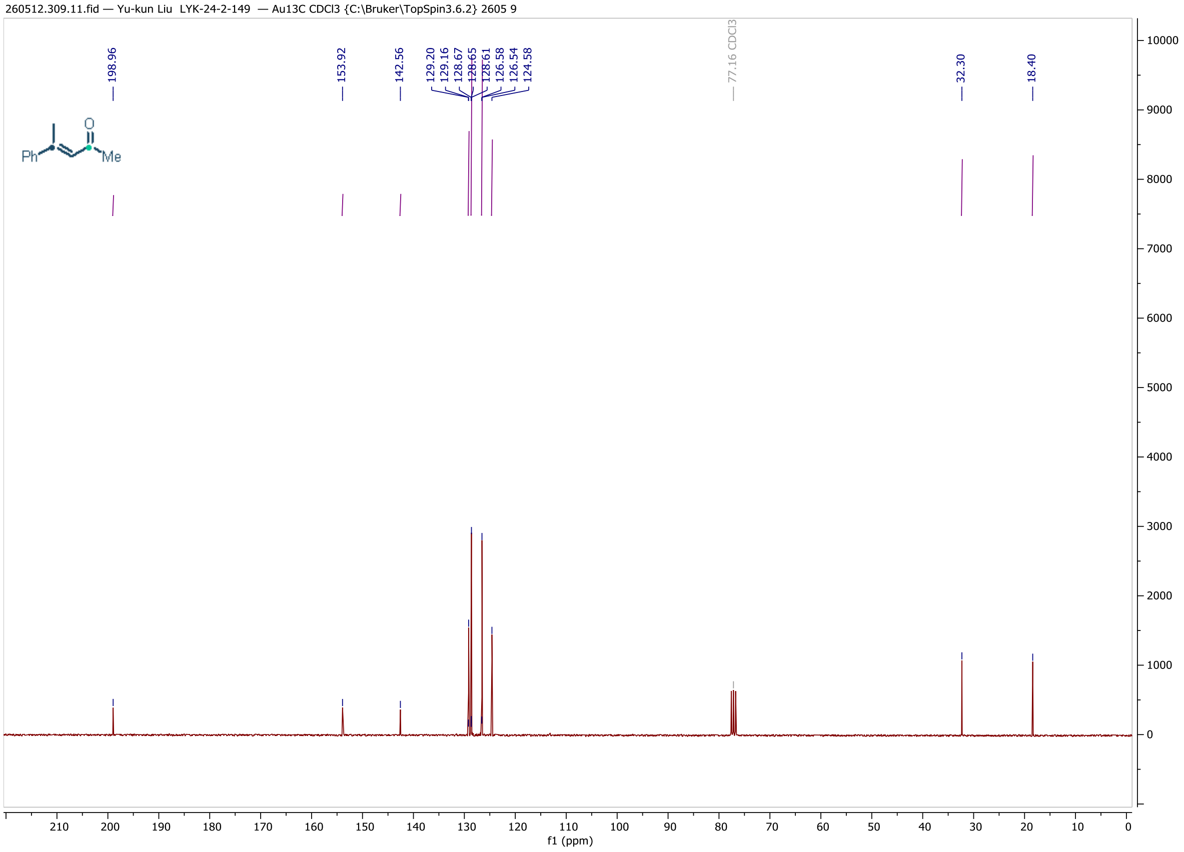

9. X-ray Crystallographic Data

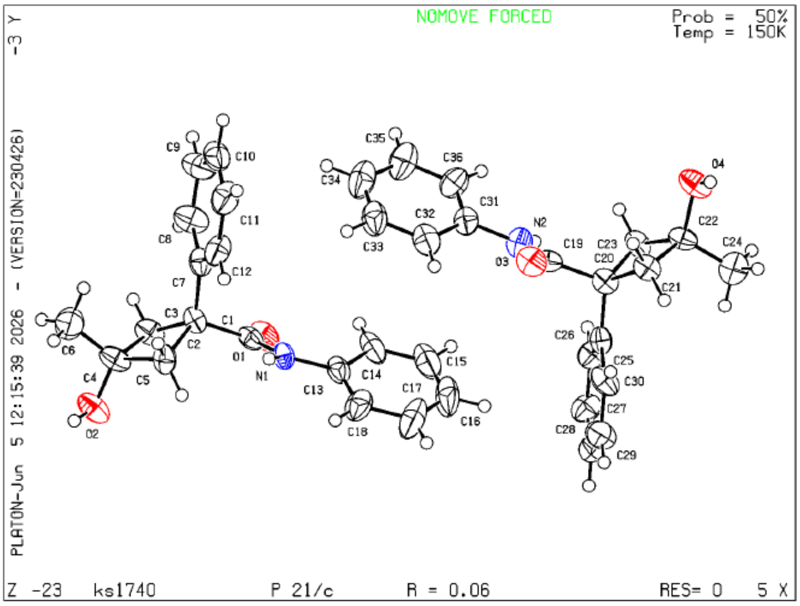

Figure 1. ORTEP drawing of product **3a** (CCDC 2554488)

Bond precision: C-C = 0.0053 A Wavelength=0.71073

Cell:	a=17.404(3)	b=18.2355(16)	c=9.9016(12)
	alpha=90	beta=101.182(11)	gamma=90

Temperature: 150 K

	Calculated	Reported
Volume	3082.8(7)	3082.9(7)
Space group	P 21/c	P 21/c
Hall group	-P 2ybc	-P 2ybc
Moiety formula	C18 H19 N O2	?
Sum formula	C18 H19 N O2	C18 H19 N O2
Mr	281.34	281.34
Dx,g cm-3	1.212	1.212
Z	8	8
Mu (mm-1)	0.079	0.079
F000	1200.0	1200.0
F000'	1200.52	
h,k,lmax	22,24,13	22,24,13
Nref	7436	7436
Tmin,Tmax	0.988,0.995	0.980,0.990
Tmin'	0.984	

Cor.r.ection method= # Reported T Limits: Tmin=0.980 Tmax=0.990
AbsCor.r. = MULTI-SCAN

Data completeness= 4.115 Theta(max)= 28.000

R(reflections)= 0.0638(13965) wR2(reflections)=0.1787(30602)

S = 0.904 Npar= 380

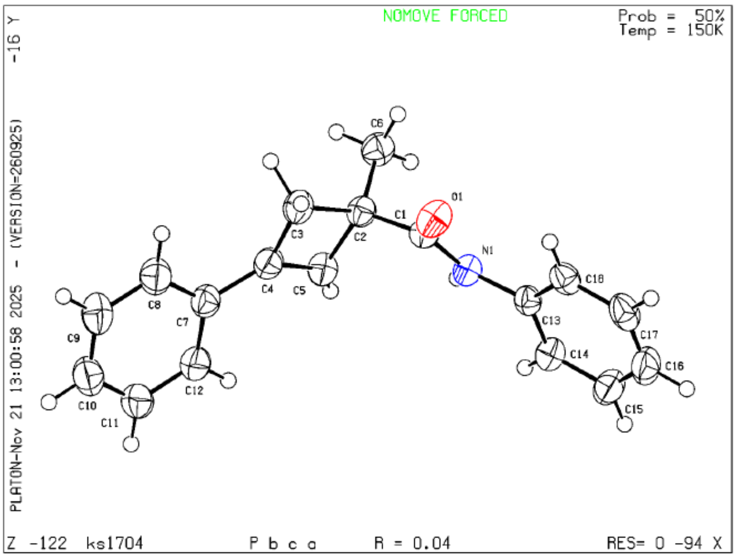

Figure 3. ORTEP drawing of product **4a** (CCDC 2501737)

Bond precision: C-C = 0.0017 A Wavelength=0.71073

Cell:	a=9.7630(2)	b=9.9422(2)	c=29.7893(6)
	alpha=90	beta=90	gamma=90

Temperature: 150 K

	Calculated	Reported
Volume	2891.52(10)	2891.52(10)
Space group	P b c a	P b c a
Hall group	-P 2ac 2ab	-P 2ac 2ab
Moiety formula	C18 H17 N O	?
Sum formula	C18 H17 N O	C18 H17 N O
Mr	263.33	263.32
Dx,g cm-3	1.210	1.210
Z	8	8
Mu (mm-1)	0.075	0.075
F000	1120.0	1120.0
F000‘	1120.44	
h,k,lmax	12,13,39	12,13,39
Nref	3490	3488
Tmin,Tmax	0.987,0.993	0.980,0.990
Tmin‘	0.983	

Cor.r.ection method= # Reported T Limits: Tmin=0.980 Tmax=0.990
AbsCor.r. = MULTI-SCAN

Data completeness= 0.999 Theta(max)= 28.000

R(reflections)= 0.0409(3049) wR2(reflections)= 0.1158(3488)

S = 1.040 Npar= 195

10. Reference

(1) Yan, X.; Zhu, Y.; Xia, Y. Nickel-Catalyzed Tunable Enantioconvergence and Kinetic Resolution in the Coupling of Tertiary Cyclobutenols with Arylboroxines. *Angew. Chem. Int. Ed*. **2023**, 62 (25), e202304462.
(2) Dubarle-Offner, J.; Marrot, J.; Rager, M.-N.; Le Bideau, F.; Jaouen, G. New Access to a Tricyclo [3.2. 1.02, 7] oct-3-ene Structure. Synlett **2007**, 2007 (05), 0800–0802.
(3) Wang, Z.; Chen, G. M.; Li, Z.; et al. Methylene cycloalkene as a reactive 1,3-diene for enantioselective hydroamination. J. Am. Chem. Soc. **2026**, 148, 13463–13472.
